# Health effects associated with smoking: a Burden of Proof study

**DOI:** 10.1038/s41591-022-01978-x

**Published:** 2022-10-10

**Authors:** Xiaochen Dai, Gabriela F. Gil, Marissa B. Reitsma, Noah S. Ahmad, Jason A. Anderson, Catherine Bisignano, Sinclair Carr, Rachel Feldman, Simon I. Hay, Jiawei He, Vincent Iannucci, Hilary R. Lawlor, Matthew J. Malloy, Laurie B. Marczak, Susan A. McLaughlin, Larissa Morikawa, Erin C. Mullany, Sneha I. Nicholson, Erin M. O’Connell, Chukwuma Okereke, Reed J. D. Sorensen, Joanna Whisnant, Aleksandr Y. Aravkin, Peng Zheng, Christopher J. L. Murray, Emmanuela Gakidou

**Affiliations:** 1 Institute for Health Metrics and Evaluation, University of Washington, Seattle, WA USA; 2 Department of Health Metrics Sciences, School of Medicine, University of Washington, Seattle, WA USA; 3 Department of Applied Mathematics, University of Washington, Seattle, WA USA

**Keywords:** Risk factors, Cancer

## Abstract

As a leading behavioral risk factor for numerous health outcomes, smoking is a major ongoing public health challenge. Although evidence on the health effects of smoking has been widely reported, few attempts have evaluated the dose–response relationship between smoking and a diverse range of health outcomes systematically and comprehensively. In the present study, we re-estimated the dose–response relationships between current smoking and 36 health outcomes by conducting systematic reviews up to 31 May 2022, employing a meta-analytic method that incorporates between-study heterogeneity into estimates of uncertainty. Among the 36 selected outcomes, 8 had strong-to-very-strong evidence of an association with smoking, 21 had weak-to-moderate evidence of association and 7 had no evidence of association. By overcoming many of the limitations of traditional meta-analyses, our approach provides comprehensive, up-to-date and easy-to-use estimates of the evidence on the health effects of smoking. These estimates provide important information for tobacco control advocates, policy makers, researchers, physicians, smokers and the public.

## Abstract (web summary)

A meta-analysis using the Burden of proof method reported consistent evidence supporting harmful associations between smoking and 28 different health outcomes.

## Main

Among both the public and the health experts, smoking is recognized as a major behavioral risk factor with a leading attributable health burden worldwide. The health risks of smoking were clearly outlined in a canonical study of disease rates (including lung cancer) and smoking habits in British doctors in 1950 and have been further elaborated in detail over the following seven decades^1,2^. In 2005, evidence of the health consequences of smoking galvanized the adoption of the first World Health Organization (WHO) treaty, the Framework Convention on Tobacco Control, in an attempt to drive reductions in global tobacco use and second-hand smoke exposure^3^. However, as of 2020, an estimated 1.18 billion individuals globally were current smokers and 7 million deaths and 177 million disability-adjusted life-years were attributed to smoking, reflecting a persistent public health challenge^4^. Quantifying the relationship between smoking and various important health outcomes—in particular, highlighting any significant dose–response relationships—is crucial to understanding the attributable health risk experienced by these individuals and informing responsive public policy.

Existing literature on the relationship between smoking and specific health outcomes is prolific, including meta-analyses, cohort studies and case–control studies analyzing the risk of outcomes such as lung cancer^5–7^, chronic obstructive pulmonary disease (COPD)^8–10^ and ischemic heart disease^11–14^ due to smoking. There are few if any attempts, however, to systematically and comprehensively evaluate the landscape of evidence on smoking risk across a diverse range of health outcomes, with most current research focusing on risk or attributable burden of smoking for a specific condition^7,15^, thereby missing the opportunity to provide a comprehensive picture of the health risk experienced by smokers. Furthermore, although evidence surrounding specific health outcomes, such as lung cancer, has generated widespread consensus, findings about the attributable risk of other outcomes are much more heterogeneous and inconclusive^16–18^. These studies also vary in their risk definitions, with many comparing dichotomous exposure measures of ever smokers versus nonsmokers^19,20^. Others examine the distinct risks of current smokers and former smokers compared with never smokers^21–23^. Among the studies that do analyze dose–response relationships, there is large variation in the units and dose categories used in reporting their findings (for example, the use of pack-years or cigarettes per day)^24,25^, which complicates the comparability and consolidation of evidence. This, in turn, can obscure data that could inform personal health choices, public health practices and policy measures. Guidance on the health risks of smoking, such as the *Surgeon General’s Reports* on smoking^26,27^, is often based on experts’ evaluation of heterogenous evidence, which, although extremely useful and well suited to carefully consider nuances in the evidence, is fundamentally subjective.

The present study, as part of the Global Burden of Diseases, Risk Factors, and Injuries Study (GBD) 2020, re-estimated the continuous dose–response relationships (the mean risk functions and associated uncertainty estimates) between current smoking and 36 health outcomes (Supplementary Table 1) by identifying input studies using a systematic review approach and employing a meta-analytic method^28^. The 36 health outcomes that were selected based on existing evidence of a relationship included 16 cancers (lung cancer, esophageal cancer, stomach cancer, leukemia, liver cancer, laryngeal cancer, breast cancer, cervical cancer, colorectal cancer, lip and oral cavity cancer, nasopharyngeal cancer, other pharynx cancer (excluding nasopharynx cancer), pancreatic cancer, bladder cancer, kidney cancer and prostate cancer), 5 cardiovascular diseases (CVDs: ischemic heart disease, stroke, atrial fibrillation and flutter, aortic aneurysm and peripheral artery disease) and 15 other diseases (COPD, lower respiratory tract infections, tuberculosis, asthma, type 2 diabetes, Alzheimer’s disease and related dementias, Parkinson’s disease, multiple sclerosis, cataracts, gallbladder diseases, low back pain, peptic ulcer disease, rheumatoid arthritis, macular degeneration and fractures). Definitions of the outcomes are described in Supplementary Table 1. We conducted a separate systematic review for each risk–outcome pair with the exception of cancers, which were done together in a single systematic review. This approach allowed us to systematically identify all relevant studies indexed in PubMed up to 31 May 2022, and we extracted relevant data on risk of smoking, including study characteristics, following a pre-specified template (Supplementary Table 2). The meta-analytic tool overcomes many of the limitations of traditional meta-analyses by incorporating between-study heterogeneity into the uncertainty of risk estimates, accounting for small numbers of studies, relaxing the assumption of log(linearity) applied to the risk functions, handling differences in exposure ranges between comparison groups, and systematically testing and adjusting for bias due to study designs and characteristics. We then estimated the burden-of-proof risk function (BPRF) for each risk–outcome pair, as proposed by Zheng et al.^29^; the BPRF is a conservative risk function defined as the 5th quantile curve (for harmful risks) that reflects the smallest harmful effect at each level of exposure consistent with the available evidence. Given all available data for each outcome, the risk of smoking is at least as harmful as the BPRF indicates.

We used the BPRF for each risk–outcome pair to calculate risk–outcome scores (ROSs) and categorize the strength of evidence for the association between smoking and each health outcome using a star rating from 1 to 5. The interpretation of the star ratings is as follows: 1 star (*) indicates no evidence of association; 2 stars (**) correspond to a 0–15% increase in risk across average range of exposures for harmful risks; 3 stars (***) represent a 15–50% increase in risk; 4 stars (****) refer to >50–85% increase in risk; and 5 stars (*****) equal >85% increase in risk. The thresholds for each star rating were developed in consultation with collaborators and other stakeholders.

The increasing disease burden attributable to current smoking, particularly in low- and middle-income countries^4^, demonstrates the relevance of the present study, which quantifies the strength of the evidence using an objective, quantitative, comprehensive and comparative framework. Findings from the present study can be used to support policy makers in making informed smoking recommendations and regulations focusing on the associations for which the evidence is strongest (that is, the 4- and 5-star associations). However, associations with a lower star rating cannot be ignored, especially when the outcome has high prevalence or severity. A summary of the main findings, limitations and policy implications of the study is presented in Table 1.

**Table 1:** Policy summary

Background	There is widespread evidence that smoking is a leading behavioral risk factor for numerous health outcomes; despite this evidence, smoking remains a persistent public health challenge. Although there is substantial research on the subject, few attempts have been made to evaluate the dose–response relationships between smoking and its many potential health outcomes in a systematic and comprehensive way or to assess the strength of the evidence for these relationships. Due to limited assessments of this kind, current guidance on and regulation in response to the risks of smoking are often based on expert evaluation of heterogeneous evidence. Expert committees are valuable in large part because they are able to consider the nuances of the available evidence, but they are also inherently subjective. In the present study, we used a systematic review approach and employed a meta-analytic framework^29^ to assess all available evidence in a way that did not force log(linearity), more fully incorporated between-study heterogeneity into estimates of uncertainty and addressed many of the other limitations of existing meta-analytic approaches.
Main findings and limitations	Even when between-study heterogeneity and other forms of uncertainty were incorporated into our assessment of risk—leading to a very conservative interpretation of the available data—smoking had a strong-to-very-strong (>50% increase in risk) association with 8 of the 36 outcomes selected for this analysis. A further 24 outcomes had weak-to-moderate evidence of an association (including 1 outcome for which smoking was protective), whereas 4 had no evidence of an association once between-study heterogeneity had been incorporated. On a 5-star scale with 1 suggesting no evidence of association (no increase in risk) and 5 very strong evidence of association (>85% increase in risk), the 8 highest pairs received 4–5 stars, the next 24 received 2–3 stars and the final 4 received just 1 star. These findings confirm that smoking is irrefutably highly harmful to human health.
	Limitations of the present study include the fact that the bias covariates we used were based on observable study characteristics and thus may not fully capture characteristics such as study quality or context; our approach to selecting which risk estimates from a given study should be included, if multiple estimates with different adjustment levels were reported, limited our ability to make full use of all available risk estimates in the literature, and we did not test for additional forms of bias such as whether studies are more consistent with each other than expected by chance.
Policy implications	The available evidence demonstrates that smoking is a highly harmful risk factor for a wide array of serious health outcomes, most notably laryngeal cancer, aortic aneurysm, bladder cancer, lung cancer and other pharynx cancer (excluding nasopharynx cancer). Policy makers should pay particular attention to the 5- and 4-star smoking–outcome pairs, for which the evidence of an association is strongest, but should not ignore lower starred pairs, particularly those with a high prevalence or severity of outcome. Our findings further validate previous conclusions about the high risk of smoking and, using our meta-analytic methods, we offer the added value of minimizing the chance that risk has been overestimated. Policy makers can therefore be confident that smoking recommendations and regulations made based on the findings from the present study are not unnecessarily restrictive.

## Results

### Overview

**Extended Data Fig. 1:**
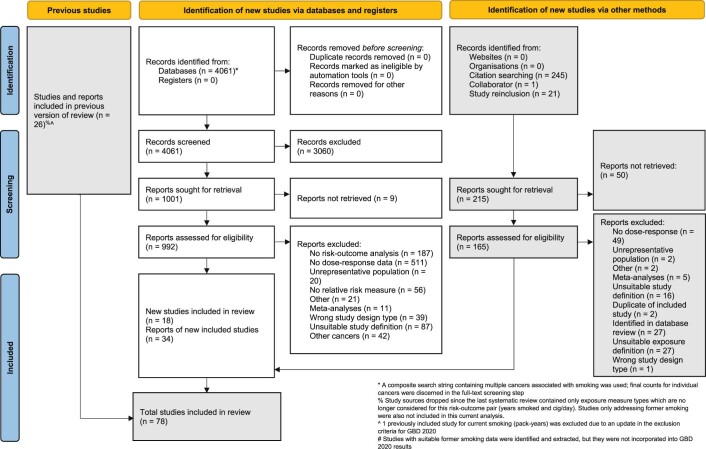
PRISMA 2020 flow diagram for an updated systematic review of the Smoking and Tracheal, bronchus, and lung cancer risk-outcome pair. The PRISMA flow diagram of an updated systematic review on the relationship between smoking and lung cancer conducted on PubMed to update historical review from previous cycles of the Global Burden of Disease Study. Template is from: Page MJ, McKenzie JE, Bossuyt PM, Boutron I, Hoffmann TC, Mulrow CD, et al. The PRISMA 2020 statement: an updated guideline for reporting systematic reviews. BMJ 2021;372:n71. doi: 10.1136/bmj.n71. For more information, visit: http://www.prisma-statement.org/.

**Extended Data Fig. 2:**
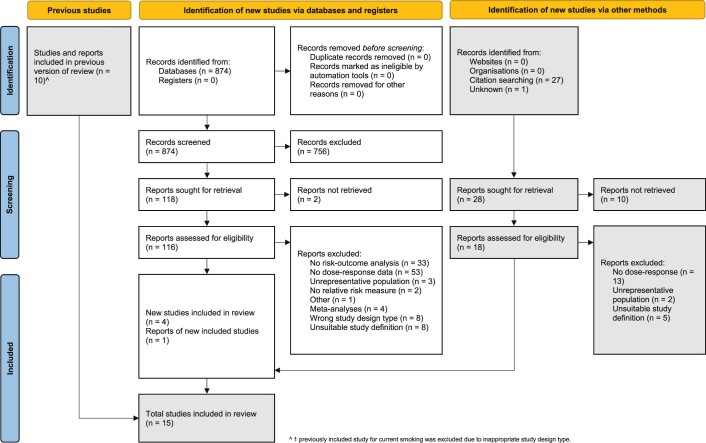
PRISMA 2020 flow diagram for an updated systematic review of the Smoking and Chronic obstructive pulmonary disease risk-outcome pair. The PRISMA flow diagram of an updated systematic review on the relationship between smoking and chronic obstructive pulmonary disease conducted on PubMed to update historical review from previous cycles of the Global Burden of Disease Study. Template is from: Page MJ, McKenzie JE, Bossuyt PM, Boutron I, Hoffmann TC, Mulrow CD, et al. The PRISMA 2020 statement: an updated guideline for reporting systematic reviews. BMJ 2021;372:n71. doi: 10.1136/bmj.n71. For more information, visit: http://www.prisma-statement.org/.

**Extended Data Fig. 3:**
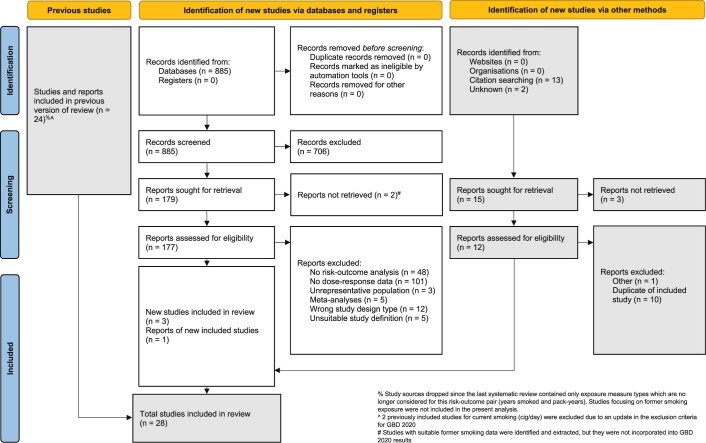
PRISMA 2020 flow diagram for an updated systematic review of the Smoking and Diabetes mellitus type 2 risk- outcome pair. The PRISMA flow diagram of an updated systematic review on the relationship between smoking and type 2 diabetes conducted on PubMed to update historical review from previous cycles of the Global Burden of Disease Study. Template is from: Page MJ, McKenzie JE, Bossuyt PM, Boutron I, Hoffmann TC, Mulrow CD, et al. The PRISMA 2020 statement: an updated guideline for reporting systematic reviews. BMJ 2021;372:n71. doi: 10.1136/bmj.n71. For more information, visit: http://www.prisma-statement.org/.

**Extended Data Fig. 4:**
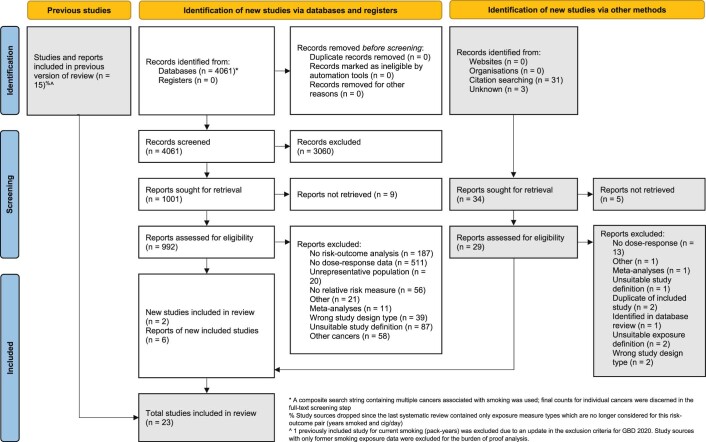
PRISMA 2020 flow diagram for an updated systematic review of the Smoking and Breast cancer risk-outcome pair. The PRISMA flow diagram of an updated systematic review on the relationship between smoking and breast cancer conducted on PubMed to update historical review from previous cycles of the Global Burden of Disease Study. Template is from: Page MJ, McKenzie JE, Bossuyt PM, Boutron I, Hoffmann TC, Mulrow CD, et al. The PRISMA 2020 statement: an updated guideline for reporting systematic reviews. BMJ 2021;372:n71. doi: 10.1136/bmj.n71. For more information, visit: http://www.prisma-statement.org/.

**Extended Data Fig. 5:**
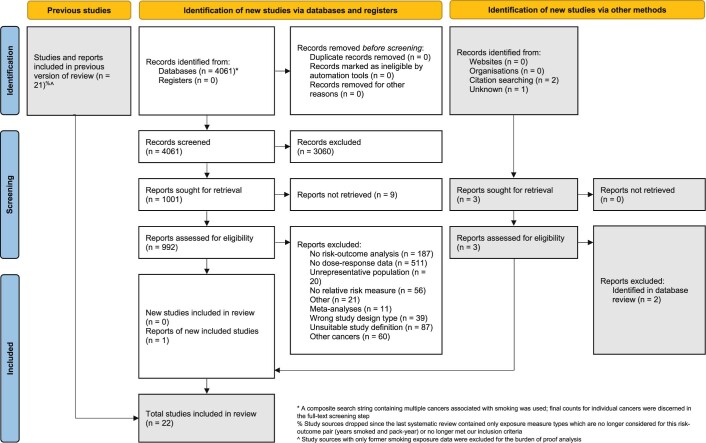
PRISMA 2020 flow diagram for an updated systematic review of the Smoking and Prostate cancer risk-outcome pair. The PRISMA flow diagram of an updated systematic review on the relationship between smoking and prostate cancer conducted on PubMed to update historical review from previous cycles of the Global Burden of Disease Study. Template is from: Page MJ, McKenzie JE, Bossuyt PM, Boutron I, Hoffmann TC, Mulrow CD, et al. The PRISMA 2020 statement: an updated guideline for reporting systematic reviews. BMJ 2021;372:n71. doi: 10.1136/bmj.n71. For more information, visit: http://www.prisma-statement.org/.

We evaluated the mean risk functions and the BPRFs for 36 health outcomes that are associated with current smoking^30^ (Table 2). Following the Preferred Reporting Items for Systematic Reviews and Meta-Analyses (PRISMA) guidelines^31^ for each of our systematic reviews, we identified studies reporting relative risk (RR) of incidence or mortality from each of the 36 selected outcomes for smokers compared with nonsmokers. We reviewed 21,108 records, which were identified to have been published between 1 May 2018 and 31 May 2022; this represents the most recent time period since the last systematic review of the available evidence for the GBD at the time of publication. The meta-analyses reported in the present study for each of the 36 health outcomes are based on evidence from a total of 793 studies published between 1970 and 2022 (Extended Data Fig. 1–5 and Supplementary Information 1.5 show the PRISMA diagrams for each outcome). Only prospective cohort and case–control studies were included for estimating dose–response risk curves, but cross-sectional studies were also included for estimating the age pattern of smoking risk on cardiovascular and circulatory disease (CVD) outcomes. Details on each, including the study’s design, data sources, number of participants, length of follow-up, confounders adjusted for in the input data and bias covariates included in the dose–response risk model, can be found in Supplementary Information 2 and 3. The theoretical minimum risk exposure level used for current smoking was never smoking or zero^30^.

**Table 2:** Strength of the evidence for the relationship between current smoking and the 36 health outcomes analyzed

Risk–outcome pair	Risk unit	Mean risk at different exposure levels				85th percentile risk level	Mean risk at 85th percentile risk level	ROSs	Average BPRF	Average increased risk (%)	Star rating	Pub. bias	No. of studies
		5	10	20	40								
Laryngeal cancer	Pack-years	2.30 (1.88, 2.84)	3.77 (2.73, 5.30)	7.25 (4.48, 12.05)	14.62 (7.62, 29.11)	50.50	17.73 (8.82, 37.07)	1.56	4.75	374.95	5	0	5
Aortic aneurism (ref. age: 55–59 years)	Cigarettes per day	2.52 (1.79, 3.60)	3.78 (2.31, 6.33)	5.39 (2.89, 10.36)	6.22 (3.17, 12.64)	30.00	6.08 (3.13, 12.27)	0.92	2.50	149.73	5	0	14
Peripheral artery disease (ref. age: 60–64 years)	Cigarettes per day	2.52 (1.67, 3.90)	3.80 (2.10, 7.14)	5.69 (2.62, 12.94)	7.82 (3.13, 20.68)	31.25	7.16 (2.98, 18.16)	0.86	2.37	136.53	5	0	6
Lung cancer	Pack-years	1.58 (1.19, 2.15)	2.48 (1.40, 4.53)	5.11 (1.84, 14.99)	11.62 (2.49, 58.73)	50.88	13.42 (2.63, 74.59)	0.73	2.07	106.66	5	1	78
Other pharynx cancer	Pack-years	1.65 (1.30, 2.13)	2.20 (1.51, 3.30)	3.02 (1.77, 5.30)	3.89 (2.02, 7.78)	63.75	4.72 (2.24, 10.45)	0.65	1.92	92.26	5	0	8
COPD	Pack-years	1.61 (1.21, 2.17)	2.16 (1.37, 3.51)	3.17 (1.60, 6.55)	5.05 (1.94, 13.97)	49.75	6.01 (2.08, 18.58)	0.54	1.72	72.11	4	1	13
Lower respiratory infection	Cigarettes per day	1.46 (1.23, 1.76)	1.83 (1.38, 2.46)	2.63 (1.68, 4.23)	2.97 (1.79, 5.06)	31.25	2.97 (1.79, 5.06)	0.43	1.54	54.45	4	0	7
Pancreatic cancer	Pack-years	1.26 (1.22, 1.31)	1.47 (1.38, 1.57)	1.72 (1.57, 1.88)	1.80 (1.63, 1.98)	51.25	1.87 (1.69, 2.08)	0.42	1.52	51.66	4	0	19
Bladder cancer	Pack-years	1.21 (1.08, 1.36)	1.43 (1.16, 1.77)	1.90 (1.31, 2.81)	2.92 (1.57, 5.63)	50.65	3.29 (1.65, 6.83)	0.34	1.40	40.18	3	1	30
Tuberculosis	Cigarettes per day	1.46 (1.13, 1.92)	1.88 (1.22, 2.97)	2.71 (1.38, 5.56)	3.47 (1.49, 8.50)	26.56	3.24 (1.46, 7.56)	0.27	1.31	31.04	3	0	19
Esophageal cancer	Pack-years	1.24 (1.06, 1.46)	1.48 (1.11, 2.00)	1.96 (1.20, 3.32)	3.12 (1.36, 7.57)	50.00	4.79 (1.53, 16.26)	0.26	1.29	29.36	3	1	14
Cervical cancer	Pack-years	1.60 (1.12, 2.35)	1.99 (1.18, 3.48)	2.37 (1.23, 4.79)	2.62 (1.26, 5.72)	25.50	2.53 (1.25, 5.36)	0.21	1.24	23.53	3	0	4
Multiple sclerosis	Cigarettes per day	1.14 (1.10, 1.17)	1.31 (1.23, 1.40)	1.78 (1.55, 2.06)	2.77 (2.17, 3.60)	20.00	1.78 (1.55, 2.06)	0.21	1.23	23.36	3	0	6
Rheumatoid arthritis	Cigarettes per day	1.20 (1.10, 1.31)	1.40 (1.20, 1.66)	1.67 (1.31, 2.14)	1.73 (1.34, 2.26)	26.25	1.72 (1.34, 2.25)	0.21	1.23	23.32	3	1	6
Lower back pain	Cigarettes per day	1.58 (1.11, 2.28)	1.96 (1.16, 3.39)	2.26 (1.20, 4.40)	2.29 (1.21, 4.50)	26.25	2.29 (1.21, 4.49)	0.20	1.22	21.84	3	0	6
Ischemic heart disease (ref. age: 55–59 years)	Cigarettes per day	1.66 (1.09, 2.57)	2.12 (1.14, 4.1)	2.43 (1.17, 5.27)	4.20 (1.28, 14.77)	31.25	3.13 (1.22, 8.50)	0.19	1.20	20.39	3	1	60
Peptic ulcer	Cigarettes per day	1.47 (1.11, 1.97)	1.79 (1.17, 2.80)	2.10 (1.22, 3.73)	2.16 (1.23, 3.92)	21.94	2.12 (1.22, 3.80)	0.18	1.20	19.84	3	0	7
Macular degeneration	Cigarettes per day	1.33 (1.07, 1.69)	1.67 (1.12, 2.54)	2.23 (1.20, 4.31)	2.42 (1.22, 5.01)	27.50	2.41 (1.22, 4.99)	0.18	1.19	19.44	3	0	2
Parkinson's disease (protective risk)	Cigarettes per day	0.79 (0.66, 0.93)	0.65 (0.47, 0.88)	0.54 (0.34, 0.84)	0.49 (0.29, 0.81)	26.25	0.51 (0.31, 0.83)	0.16	0.85	−14.88	3	1	14
Stomach cancer	Pack-years	1.10 (1.06, 1.15)	1.18 (1.10, 1.27)	1.29 (1.16, 1.44)	1.39 (1.22, 1.60)	51.13	1.61 (1.32, 1.97)	0.16	1.17	17.39	3	1	13
Stroke (ref. age: 55–59)	Cigarettes per day	1.40 (1.07, 1.88)	1.75 (1.11, 2.82)	2.23 (1.16, 4.45)	2.43 (1.18, 5.22)	29.50	2.42 (1.18, 5.19)	0.16	1.17	16.89	3	0	67
Type 2 diabetes	Cigarettes per day	1.23 (1.09, 1.39)	1.38 (1.15, 1.68)	1.49 (1.18, 1.90)	1.67 (1.24, 2.29)	26.25	1.54 (1.20, 2.01)	0.15	1.16	15.76	3	1	28
Cataracts	Cigarettes per day	1.10 (1.08, 1.12)	1.16 (1.13, 1.19)	1.29 (1.23, 1.35)	1.70 (1.54, 1.88)	25.00	1.43 (1.34, 1.53)	0.14	1.15	15.47	3	0	10
Nasopharyngeal cancer	Pack-years	1.16 (1.04, 1.31)	1.28 (1.06, 1.56)	1.39 (1.08, 1.83)	1.97 (1.17, 3.43)	50.00	2.67 (1.26, 5.94)	0.13	1.14	14.29	2	1	12
Alzheimer’s and other dementia	Cigarettes per day	1.16 (1.04, 1.30)	1.29 (1.06, 1.58)	1.46 (1.10, 1.96)	1.74 (1.14, 2.70)	30.00	1.54 (1.11, 2.18)	0.09	1.10	9.70	2	1	8
Gallbladder diseases	Cigarettes per day	1.15 (1.03, 1.30)	1.24 (1.05, 1.49)	1.29 (1.05, 1.61)	1.51 (1.09, 2.14)	27.93	1.41 (1.07, 1.88)	0.06	1.06	6.34	2	0	4
Atrial fibrillation and flutter (ref. age: 55–59 years)	Cigarettes per day	1.34 (1.03, 1.78)	1.40 (1.03, 1.93)	1.40 (1.03, 1.93)	1.40 (1.03, 1.93)	25.00	1.40 (1.03, 1.93)	0.06	1.06	5.67	2	0	5
Lip and oral cavity cancer	Pack-years	1.15 (1.00, 1.34)	1.37 (0.99, 1.94)	2.05 (0.98, 4.50)	3.50 (0.97, 13.93)	49.68	3.91 (0.96, 17.56)	0.05	1.05	4.81	2	0	10
Breast cancer	Pack-years	1.08 (1.02, 1.14)	1.13 (1.04, 1.24)	1.17 (1.04, 1.31)	1.17 (1.04, 1.31)	34.10	1.17 (1.04, 1.31)	0.04	1.04	4.46	2	0	23
Colon and rectum cancer	Pack-years	1.07 (0.99, 1.16)	1.12 (0.98, 1.29)	1.19 (0.97, 1.46)	1.20 (0.97,1.50)	50.00	1.20 (0.97, 1.50)	−0.01	0.99	N/A	1	0	16
Kidney cancer	Pack-years	1.01 (1.00, 1.02)	1.04 (0.99, 1.09)	1.15 (0.98, 1.36)	1.52 (0.94, 2.53)	45.86	1.59 (0.93, 2.79)	−0.01	0.99	N/A	1	1	18
Leukemia	Pack-years	1.04 (0.98, 1.11)	1.07 (0.97, 1.19)	1.10 (0.95, 1.29)	1.62 (0.79, 3.47)	37.50	1.50 (0.82, 2.80)	−0.04	0.96	N/A	1	0	7
Fracture	Binary	N/A	N/A	N/A	N/A	1 (binary)	1.34 (0.84, 1.97)	−0.05	0.95	N/A	1	0	59
Prostate cancer	Cigarettes per day	1.03 (0.98, 1.10)	1.07 (0.95, 1.21)	1.16 (0.89, 1.53)	1.30 (0.82, 2.10)	29.73	1.25 (0.85, 1.89)	−0.06	0.94	N/A	1	1	22
Liver cancer	Pack-years	1.15 (0.84, 1.59)	1.26 (0.75, 2.19)	1.39 (0.65, 3.10)	1.42 (0.64, 3.30)	62.50	1.42 (0.64, 3.30)	−0.32	0.72	N/A	1	0	11
Asthma	Cigarettes per day	1.41 (0.54, 3.96)	1.59 (0.43, 6.39)	1.61 (0.42, 6.74)	1.64 (0.41, 7.23)	26.25	1.63 (0.41, 7.04)	−0.64	0.53	N/A	1	0	7

The ROS represents the signed value of the log(BPRF) averaged across the 15th–85th percentiles of exposure. The BPRF corresponds to the lower (if harmful) or higher (if protective) UI—inclusive of between-study heterogeneity—for each risk–outcome pair’s RR curve. ROSs are directly comparable across outcomes and each risk–outcome pair receives an ROS based on the final formulation of the risk curve. For Parkinson’s disease, the ROS reflects a protective effect of smoking, whereas for the other outcomes it reflects a harmful effect. Negative ROSs indicate that a conservative interpretation of the available evidence suggests that there may be no association between risk and outcome. For ease of interpretation, we have transformed the ROS and BPRF into a star rating (1–5), with a higher rating representing a larger effect and stronger evidence. Average BPRF, which is the exponential ROS for harmful effects (or exponential negative ROS for protective effects), is the conservative exposure-averaged RR consistent with all the available data. Average increased risk, which equates to (average BPRF − 1) × 100% for harmful effects or (1 − average BPRF) × 100% for protective effects, refers to the percentage increase in RR based on a conservative interpretation of the evidence. For harmful risks, this percentage is positive and, for protective risks, negative, indicating the percentage decrease in RR. The average increased risk is not applicable for pairs with negative ROSs. N/A, not available; Pub., Publication; ref., reference.

### Five-star associations

When the most conservative interpretation of the evidence, that is, the BPRF, suggests that the average exposure (15th–85th percentiles of exposure) of smoking increases the risk of a health outcome by >85% (that is, ROS > 0.62), smoking and that outcome are categorized as a 5-star pair. Among the 36 outcomes, there are 5 that have a 5-star association with current smoking: laryngeal cancer (375% increase in risk based on the BPRF, 1.56 ROS), aortic aneurysm (150%, 0.92), peripheral artery disease (137%, 0.86), lung cancer (107%, 0.73) and other pharynx cancer (excluding nasopharynx cancer) (92%, 0.65).

**Fig. 1:**
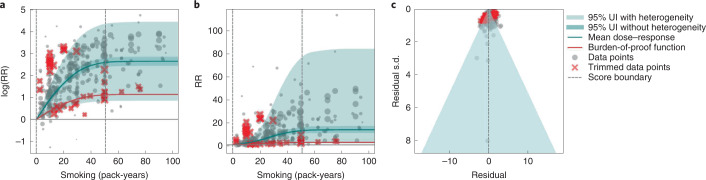
Smoking and lung cancer. **a**, The log(RR) function. **b**, RR function. **c**, A modified funnel plot showing the residuals (relative to 0) on the *x* axis and the estimated s.d. that includes reported s.d. and between-study heterogeneity on the *y* axis.

Results for all 5-star risk–outcome pairs are available in Table 2 and Supplementary Information 4.1. In the present study, we provide detailed results for one example 5-star association: current smoking and lung cancer. We extracted 371 observations from 25 prospective cohort studies and 53 case–control studies across 25 locations (Supplementary Table 3)^5,6,32–107^. Exposure ranged from 1 pack-year to >112 pack-years, with the 85th percentile of exposure being 50.88 pack-years (Fig. 1a).

We found a very strong and significant harmful relationship between pack-years of current smoking and the RR of lung cancer (Fig. 1b). The mean RR of lung cancer at 20 pack-years of smoking was 5.11 (95% uncertainty interval (UI) inclusive of between-study heterogeneity = 1.84–14.99). At 50.88 pack-years (85th percentile of exposure), the mean RR of lung cancer was 13.42 (2.63–74.59). See Table 2 for mean RRs at other exposure levels. The BPRF, which represents the most conservative interpretation of the evidence (Fig. 1a), suggests that smoking in the 15th–85th percentiles of exposure increases the risk of lung cancer by an average of 107%, yielding an ROS of 0.73.

The relationship between pack-years of current smoking and RR of lung cancer is nonlinear, with diminishing impact of further pack-years of smoking, particularly for middle-to-high exposure levels (Fig. 1b). To reduce the effect of bias, we adjusted observations that did not account for more than five confounders, including age and sex, because they were the significant bias covariates identified by the bias covariate selection algorithm^29^ (Supplementary Table 7). The reported RRs across studies were very heterogeneous. Our meta-analytic method, which accounts for the reported uncertainty in both the data and between-study heterogeneity, fit the data and covered the estimated residuals well (Fig. 1c). After trimming 10% of outliers, we still detected publication bias in the results for lung cancer. See Supplementary Tables 4 and 7 for study bias characteristics and selected bias covariates, Supplementary Fig. 5 for results without 10% trimming and Supplementary Table 8 for observed RR data and alternative exposures across studies for the remaining 5-star pairs.

### Four-star associations

When the BPRF suggests that the average exposure of smoking increases the risk of a health outcome by 50–85% (that is, ROS > 0.41–0.62), smoking is categorized as having a 4-star association with that outcome. We identified three outcomes with a 4-star association with smoking: COPD (72% increase in risk based on the BPRF, 0.54 ROS), lower respiratory tract infection (54%, 0.43) and pancreatic cancer (52%, 0.42).

In the present study, we provide detailed results for one example 4-star association: current smoking and COPD. We extracted 51 observations from 11 prospective cohort studies and 4 case–control studies across 36 locations (Supplementary Table 3)^6,8–10,78,108–116^. Exposure ranged from 1 pack-year to 100 pack-years, with the 85th percentile of exposure in the exposed group being 49.75 pack-years.

**Fig. 2:**
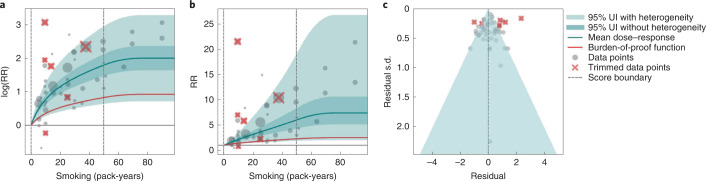
Smoking and COPD. **a**, The log(RR) function. **b**, RR function. **c**, A modified funnel plot showing the residuals (relative to 0) on th*e* x axis and the estimated s.d. that includes the reported s.d. and between-study heterogeneity on the *y* axis.

We found a strong and significant harmful relationship between pack-years of current smoking and RR of COPD (Fig. 2b). The mean RR of COPD at 20 pack-years was 3.17 (1.60–6.55; Table 2 reports RRs at other exposure levels). At the 85th percentile of exposure, the mean RR of COPD was 6.01 (2.08–18.58). The BPRF suggests that average smoking exposure raises the risk of COPD by an average of 72%, yielding an ROS of 0.54. The results for the other health outcomes that have an association with smoking rated as 4 stars are shown in Table 2 and Supplementary Information 4.2.

The relationship between smoking and COPD is nonlinear, with diminishing impact of further pack-years of current smoking on risk of COPD, particularly for middle-to-high exposure levels (Fig. 2a). To reduce the effect of bias, we adjusted observations that did not account for age and sex and/or were generated for individuals aged >65 years^116^, because they were the two significant bias covariates identified by the bias covariate selection algorithm (Supplementary Table 7). There was large heterogeneity in the reported RRs across studies, and our meta-analytic method fit the data and covered the estimated residuals well (Fig. 2b). Although we trimmed 10% of outliers, publication bias was still detected in the results for COPD. See Supplementary Tables 4 and 7 for study bias characteristics and selected bias covariates, Supplementary Fig. 5 for results without 10% trimming and Supplementary Table 8 for reported RR data and alternative exposures across studies for the remaining health outcomes that have a 4-star association with smoking.

### Three-star associations

When the BPRF suggests that the average exposure of smoking increases the risk of a health outcome by 15–50% (or, when protective, decreases the risk of an outcome by 13–34%; that is, ROS >0.14–0.41), the association between smoking and that outcome is categorized as having a 3-star rating. We identified 15 outcomes with a 3-star association: bladder cancer (40% increase in risk, 0.34 ROS); tuberculosis (31%, 0.27); esophageal cancer (29%, 0.26); cervical cancer, multiple sclerosis and rheumatoid arthritis (each 23–24%, 0.21); lower back pain (22%, 0.20); ischemic heart disease (20%, 0.19); peptic ulcer and macular degeneration (each 19–20%, 0.18); Parkinson's disease (protective risk, 15% decrease in risk, 0.16); and stomach cancer, stroke, type 2 diabetes and cataracts (each 15–17%, 0.14–0.16).

We present the findings on smoking and type 2 diabetes as an example of a 3-star risk association. We extracted 102 observations from 24 prospective cohort studies and 4 case–control studies across 15 locations (Supplementary Table 3)^117–144^. The exposure ranged from 1 cigarette to 60 cigarettes smoked per day, with the 85th percentile of exposure in the exposed group being 26.25 cigarettes smoked per day.

**Fig. 3:**
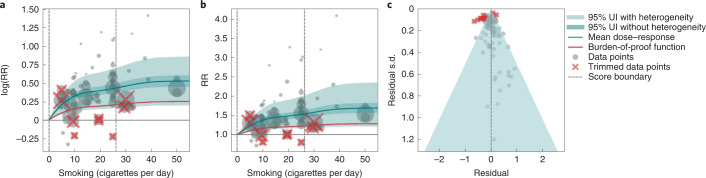
Smoking and type 2 diabetes. **a**, The log(RR) function. **b**, RR function. **c**, A modified funnel plot showing the residuals (relative to 0) on the *x* axis and the estimated s.d. that includes the reported s.d. and between-study heterogeneity on the *y* axis.

We found a moderate and significant harmful relationship between cigarettes smoked per day and the RR of type 2 diabetes (Fig. 3b). The mean RR of type 2 diabetes at 20 cigarettes smoked per day was 1.49 (1.18–1.90; see Table 2 for other exposure levels). At the 85th percentile of exposure, the mean RR of type 2 diabetes was 1.54 (1.20–2.01). The BPRF suggests that average smoking exposure raises the risk of type 2 diabetes by an average of 16%, yielding an ROS of 0.15. See Table 2 and Supplementary Information 4.3 for results for the additional health outcomes with an association with smoking rated as 3 stars.

The relationship between smoking and type 2 diabetes is nonlinear, particularly for high exposure levels where the mean risk curve becomes flat (Fig. 3a). We adjusted observations that were generated in subpopulations, because it was the only significant bias covariate identified by the bias covariate selection algorithm (Supplementary Table 7). There was moderate heterogeneity in the observed RR data across studies and our meta-analytic method fit the data and covered the estimated residuals extremely well (Fig. 3b,c). After trimming 10% of outliers, we still detected publication bias in the results for type 2 diabetes. See Supplementary Tables 4 and 7 for study bias characteristics and selected bias covariates, Supplementary Fig. 5 for results without 10% trimming and Supplementary Table 8 for observed RR data and alternative exposures across studies for the remaining 3-star pairs.

### Two-star associations

When the BPRF suggests that the average exposure of smoking increases the risk of an outcome by 0–15% (that is, ROS 0.0–0.14), the association between smoking and that outcome is categorized as a 2-star rating. We identified six 2-star outcomes: nasopharyngeal cancer (14% increase in risk, 0.13 ROS); Alzheimer’s and other dementia (10%, 0.09); gallbladder diseases and atrial fibrillation and flutter (each 6%, 0.06); lip and oral cavity cancer (5%, 0.05); and breast cancer (4%, 0.04).

We present the findings on smoking and breast cancer as an example of a 2-star association. We extracted 93 observations from 14 prospective cohort studies and 9 case–control studies across 14 locations (Supplementary Table 3)^84,87,145–165^. The exposure ranged from 1 cigarette to >76 cigarettes smoked per day, with the 85th percentile of exposure in the exposed group being 34.10 cigarettes smoked per day.

**Extended Data Fig. 6:**
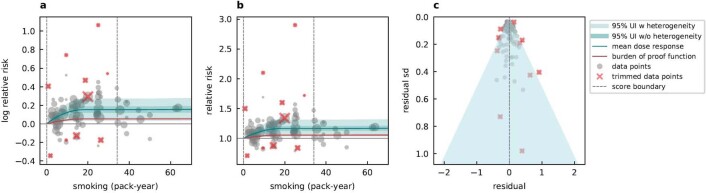
Smoking and Breast Cancer. **a**, log-relative risk function. **b**, relative risk function. **c**, A modified funnel plot showing the residuals (relative to 0) on the x-axis and the estimated standard deviation (SD) that includes reported SD and between-study heterogeneity on the y-axis.

We found a weak but significant relationship between pack-years of current smoking and RR of breast cancer (Extended Data Fig. 6). The mean RR of breast cancer at 20 pack-years was 1.17 (1.04–1.31; Table 2 reports other exposure levels). The BPRF suggests that average smoking exposure raises the risk of breast cancer by an average of 4%, yielding an ROS of 0.04. See Table 2 and Supplementary Information 4.4 for results on the additional health outcomes for which the association with smoking has been categorized as 2 stars.

The relationship between smoking and breast cancer is nonlinear, particularly for high exposure levels where the mean risk curve becomes flat (Extended Data Fig. 6a). To reduce the effect of bias, we adjusted observations that were generated in subpopulations, because it was the only significant bias covariate identified by the bias covariate selection algorithm (Supplementary Table 7). There was heterogeneity in the reported RRs across studies, but our meta-analytic method fit the data and covered the estimated residuals (Extended Data Fig. 6b).﻿ After trimming 10% of outliers, we did not detect publication bias in the results for breast cancer. See Supplementary Tables 4 and 7 for study bias characteristics and selected bias covariates, Supplementary Fig. 5 for results without 10% trimming and Supplementary Table 8 for observed RR data and alternative exposures across studies for the remaining 2-star pairs.

### One-star associations

When average exposure to smoking does not significantly increase (or decrease) the risk of an outcome, once between-study heterogeneity and other sources of uncertainty are accounted for (that is, ROS < 0), the association between smoking and that outcome is categorized as 1 star, indicating that there is not sufficient evidence for the effect of smoking on the outcome to reject the null (that is, there may be no association). There were seven outcomes with an association with smoking that rated as 1 star: colorectal and kidney cancer (each –0.01 ROS); leukemia (−0.04); fractures (−0.05); prostate cancer (−0.06); liver cancer (−0.32); and asthma (−0.64).

We use smoking and prostate cancer as examples of a 1-star association. We extracted 78 observations from 21 prospective cohort studies and 1 nested case–control study across 15 locations (Supplementary Table 3)^157,160,166–185^. The exposure among the exposed group ranged from 1 cigarette to 90 cigarettes smoked per day, with the 85th percentile of exposure in the exposed group being 29.73 cigarettes smoked per day.

**Fig. 4:**
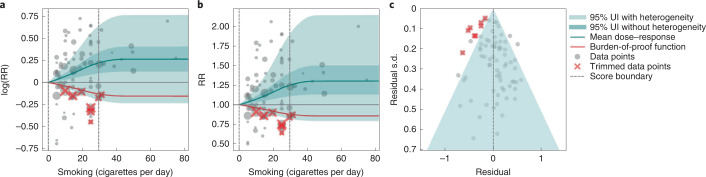
Smoking and prostate cancer. **a**, The log(RR) function. **b**, RR function. **c**, A modified funnel plot showing the residuals (relative to 0) on the *x* axis and the estimated s.d. that includes reported s.d. and between-study heterogeneity on the *y* axis.

Based on our conservative interpretation of the data, we did not find a significant relationship between cigarettes smoked per day and the RR of prostate cancer (Fig. 4B). The exposure-averaged BPRF for prostate cancer was 0.94, which was opposite null from the full range of mean RRs, such as 1.16 (0.89–1.53) at 20 cigarettes smoked per day. The corresponding ROS was −0.06, which is consistent with no evidence of an association between smoking and increased risk of prostate cancer. See Table 2 and Supplementary Information 4.5 for results for the additional outcomes that have a 1-star association with smoking.

The relationship between smoking and prostate cancer is nonlinear, particularly for middle-to-high exposure levels where the mean risk curve becomes flat (Fig. 4a). We did not adjust for any bias covariate because no significant bias covariates were selected by the algorithm (Supplementary Table 7). The RRs reported across studies were very heterogeneous, but our meta-analytic method fit the data and covered the estimated residuals well (Fig. 4b,c). The ROS associated with the BPRF is −0.05, suggesting that the most conservative interpretation of all evidence, after accounting for between-study heterogeneity, indicates an inconclusive relationship between smoking exposure and the risk of prostate cancer. After trimming 10% of outliers, we still detected publication bias in the results for prostate cancer, which warrants further studies using sample populations. See Supplementary Tables 4 and 7 for study bias characteristics and selected bias covariates, Supplementary Fig. 5 for results without 10% trimming and Supplementary Table 8 for observed RR data and alternative exposures across studies for the remaining 1-star pairs.

### Age-specific dose–response risk for CVD outcomes

We produced age-specific dose–response risk curves for the five selected CVD outcomes (Methods). The ROS associated with each smoking–CVD pair was calculated based on the reference risk curve estimated using all risk data regardless of age information. Estimation of the BPRF, calculation of the associated ROS and star rating of the smoking–CVD pairs follow the same rules as the other non-CVD smoking–outcome pairs (Table 1 and Supplementary Figs. 2–4). Once we had estimated the reference dose–response risk curve for each CVD outcome, we determined the age group of the reference risk curve. The reference age group is 55–59 years for all CVD outcomes, except for peripheral artery disease, the reference age group for which is 60–64 years. We then estimated the age pattern of smoking on all CVD outcomes (Supplementary Fig. 2) and calculated age attenuation factors of the risk for each age group by comparing the risk of each age group with that of the reference age group, using the estimated age pattern (Supplementary Fig. 3). Last, we applied the draws of age attenuation factors of each age group to the dose–response risk curve for the reference age group to produce the age group-specific dose–response risk curves for each CVD outcome (Supplementary Fig. 4).

## Discussion

Using our burden-of-proof meta-analytic methods, we re-estimated the dose–response risk of smoking on 36 health outcomes that had previously been demonstrated to be associated with smoking^30,186^. Using these methods, which account for both the reported uncertainty of the data and the between-study heterogeneity, we found that 29 of the 36 smoking–outcome pairs are supported by evidence that suggests a significant dose–response relationship between smoking and the given outcome (28 with a harmful association and 1 with a protective association). Conversely, after accounting for between-study heterogeneity, the available evidence of smoking risk on seven outcomes (that is, colon and rectum cancer, kidney cancer, leukemia, prostate cancer, fractures, liver cancer and asthma) was insufficient to reject the null or draw definitive conclusions on their relationship to smoking. Among the 29 outcomes that have evidence supporting a significant relationship to smoking, 8 had strong-to-very-strong evidence of a relationship, meaning that, given all the available data on smoking risk, we estimate that average exposure to smoking increases the risk of those outcomes by >50% (4- and 5-star outcomes). The currently available evidence for the remaining 21 outcomes with a significant association with current smoking was weak to moderate, indicating that smoking increases the risk of those outcomes by at least >0–50% (2- and 3-star associations).

Even under our conservative interpretation of the data, smoking is irrefutably harmful to human health, with the greatest increases in risk occurring for laryngeal cancer, aortic aneurysm, peripheral artery disease, lung cancer and other pharynx cancer (excluding nasopharynx cancer), which collectively represent large causes of death and ill-health. The magnitude of and evidence for the associations between smoking and its leading health outcomes are among the highest currently analyzed in the burden-of-proof framework^29^. The star ratings assigned to each smoking–outcome pair offer policy makers a way of categorizing and comparing the evidence for a relationship between smoking and its potential health outcomes (https://vizhub.healthdata.org/burden-of-proof). We found that, for seven outcomes in our analysis, there was insufficient or inconsistent evidence to demonstrate a significant association with smoking. This is a key finding because it demonstrates the need for more high-quality data for these particular outcomes; availability of more data should improve the strength of evidence for whether or not there is an association between smoking and these health outcomes.

Our systematic review approach and meta-analytic methods have numerous benefits over existing systematic reviews and meta-analyses on the same topic that use traditional random effects models. First, our approach relaxes the log(linear) assumption, using a spline ensemble to estimate the risk^29^. Second, our approach allows variable reference groups and exposure ranges, allowing for more accurate estimates regardless of whether or not the underlying relative risk is log(linear). Furthermore, it can detect outliers in the data automatically. Finally, it quantifies uncertainty due to between-study heterogeneity while accounting for small numbers of studies, minimizing the risk that conclusions will be drawn based on spurious findings.

We believe that the results for the association between smoking and each of the 36 health outcomes generated by the present study, including the mean risk function, BPRF, ROS, average excess risk and star rating, could be useful to a range of stakeholders. Policy makers can formulate their decisions on smoking control priorities and resource allocation based on the magnitude of the effect and the consistency of the evidence relating smoking to each of the 36 outcomes, as represented by the ROS and star rating for each smoking–outcome association^187^. Physicians and public health practitioners can use the estimates of average increased risk and the star rating to educate patients and the general public about the risk of smoking and to promote smoking cessation^188^. Researchers can use the estimated mean risk function or BPRF to obtain the risk of an outcome at a given smoking exposure level, as well as uncertainty surrounding that estimate of risk. The results can also be used in the estimation of risk-attributable burden, that is, the deaths and disability-adjusted life-years due to each outcome that are attributable to smoking^30,186^. For the general public, these results could help them to better understand the risk of smoking and manage their health^189^.

Although our meta-analysis was comprehensive and carefully conducted, there are limitations to acknowledge. First, the bias covariates used, although carefully extracted and evaluated, were based on observable study characteristics and thus may not fully capture unobserved characteristics such as study quality or context, which might be major sources of bias. Second, if multiple risk estimates with different adjustment levels were reported in a given study, we included only the fully adjusted risk estimate and modeled the adjustment level according to the number of covariates adjusted for (rather than which covariates were adjusted for) and whether a standard adjustment for age and sex had been applied. This approach limited our ability to make full use of all available risk estimates in the literature. Third, although we evaluated the potential for publication bias in the data, we did not test for other forms of bias such as when studies are more consistent with each other than expected by chance^29^. Fourth, our analysis assumes that the relationships between smoking and health outcomes are similar across geographical regions and over time. We do not have sufficient evidence to quantify how the relationships may have evolved over time because the composition of smoking products has also changed over time. Perhaps some of the heterogeneity of the effect sizes in published studies reflects this; however, this cannot be discerned with the currently available information.

In the future, we plan to include crude and partially adjusted risk estimates in our analyses to fully incorporate all available risk estimates, to model the adjusted covariates in a more comprehensive way by mapping the adjusted covariates across all studies comprehensively and systematically, and to develop methods to evaluate additional forms of potential bias. We plan to update our results on a regular basis to provide timely and up-to-date evidence to stakeholders.

To conclude, we have re-estimated the dose–response risk of smoking on 36 health outcomes while synthesizing all the available evidence up to 31 May 2022. We found that, even after factoring in the heterogeneity between studies and other sources of uncertainty, smoking has a strong-to-very-strong association with a range of health outcomes and confirmed that smoking is irrefutably highly harmful to human health. We found that, due to small numbers of studies, inconsistency in the data, small effect sizes or a combination of these reasons, seven outcomes for which some previous research had found an association with smoking did not—under our meta-analytic framework and conservative approach to interpreting the data—have evidence of an association. Our estimates of the evidence for risk of smoking on 36 selected health outcomes have the potential to inform the many stakeholders of smoking control, including policy makers, researchers, public health professionals, physicians, smokers and the general public.

## Methods

### Overview

For the present study, we used a meta-analytic tool, MR-BRT (metaregression—Bayesian, regularized, trimmed), to estimate the dose–response risk curves of the risk of a health outcome across the range of current smoking levels along with uncertainty estimates^28^. Compared with traditional meta-analysis using linear mixed effect models, MR-BRT relaxes the assumption of a log(linear) relationship between exposure and risk, incorporates between-study heterogeneity into the uncertainty of risk estimates, handles estimates reported across different exposure categories, automatically identifies and trims outliers, and systematically tests and adjusts for bias due to study designs and characteristics. The meta-analytic methods employed by the present study followed the six main steps proposed by Zheng et al.^28,29^, namely: (1) enacting a systematic review approach and data extraction following a pre-specified and standardized protocol; (2) estimating the shape of the relationship between exposure and RR; (3) evaluating and adjusting for systematic bias as a function of study characteristics and risk estimation; (4) quantifying between-study heterogeneity while adjusting for within-study correlation and the number of studies; (5) evaluating potential publication or reporting biases; and (6) estimating the mean risk function and the BPRF, calculating the ROS and categorizing smoking–outcome pairs using a star-rating scheme from 1 to 5.

The estimates for our primary indicators of this work—mean RRs across a range of exposures, BRPFs, ROSs and star ratings for each risk–outcome pair—are not specific to or disaggregated by specific populations. We did not estimate RRs separately for different locations, sexes (although the RR of prostate cancer was estimated only for males and of cervical and breast cancer only for females) or age groups (although this analysis was applied to disease endpoints in adults aged ≥30 years only and, as detailed below, age-specific estimates were produced for the five CVD outcomes).

The present study complies with the PRISMA guidelines^190^ (Supplementary Tables 9 and 10 and Supplementary Information 1.5) and Guidelines for Accurate and Transparent Health Estimates Reporting (GATHER) recommendations^191^ (Supplementary Table 11). The study was approved by the University of Washington Institutional Review Board (study no. 9060). The systematic review approach was not registered.

### Selecting health outcomes

In the present study, current smoking is defined as the current use of any smoked tobacco product on a daily or occasional basis. Health outcomes were initially selected using the World Cancer Research Fund criteria for convincing or probable evidence as described in Murray et al.^186^. The 36 health outcomes that were selected based on existing evidence of a relationship included 16 cancers (lung cancer, esophageal cancer, stomach cancer, leukemia, liver cancer, laryngeal cancer, breast cancer, cervical cancer, colorectal cancer, lip and oral cavity cancer, nasopharyngeal cancer, other pharynx cancer (excluding nasopharynx cancer), pancreatic cancer, bladder cancer, kidney cancer and prostate cancer), 5 CVDs (ischemic heart disease, stroke, atrial fibrillation and flutter, aortic aneurysm and peripheral artery disease) and 15 other diseases (COPD, lower respiratory tract infections, tuberculosis, asthma, type 2 diabetes, Alzheimer’s disease and related dementias, Parkinson’s disease, multiple sclerosis, cataracts, gallbladder diseases, low back pain, peptic ulcer disease, rheumatoid arthritis, macular degeneration and fracture). Definitions of the outcomes are described in Supplementary Table 1.

### Step 1: systematic review approach to literature search and data extraction

Informed by the systematic review approach we took for the GBD 2019 (ref. ^30^), for the present study we identified input studies in the literature using a systematic review approach for all 36 smoking–outcome pairs using updated search strings to identify all relevant studies indexed in PubMed up to 31 May 2022 and extracted data on smoking risk estimates. Briefly, the studies that were extracted represented several types of study design (for example, cohort and case–control studies), measured exposure in several different ways and varied in their choice of reference categories (where some compared current smokers with never smokers, whereas others compared current smokers with nonsmokers or former smokers). All these study characteristics were catalogued systematically and taken into consideration during the modeling part of the analysis.

In addition, for CVD outcomes, we also estimated the age pattern of risk associated with smoking. We applied a systematic review of literature approach for smoking risk for the five CVD outcomes. We developed a search string to search for studies reporting any association between binary smoking status (that is, current, former and ever smokers) and the five CVD outcomes from 1 January 1970 to 31 May 2022, and included only studies reporting age-specific risk (RR, odds ratio (OR), hazard ratio (HR)) of smoking status. The inclusion criteria and results of the systematic review approach are reported in accordance with PRISMA guidelines^31^. Details for each outcome on the search string used in the systematic review approach, refined inclusion and exclusion criteria, data extraction template and PRISMA diagram are given in Supplementary Information 1. Title and/or abstract screening, full text screening and data extraction were conducted by 14 members of the research team and extracted data underwent manual quality assurance by the research team to verify accuracy.

### Selecting exposure categories

Cumulative exposure in pack-years was the measure of exposure used for COPD and all cancer outcomes except for prostate cancer, to reflect the risk of both duration and intensity of current smoking on these outcomes. For prostate cancer, CVDs and all the other outcomes except for fractures, we used cigarette-equivalents smoked per day as the exposure for current smoking, because smoking intensity is generally thought to be more important than duration for these outcomes. For fractures, we used binary exposure, because there were few studies examining intensity or duration of smoking on fractures. The smoking–outcome pairs and the corresponding exposures are summarized in Supplementary Table 4 and are congruent with the GBD 2019 (refs. ^30,186^).

### Steps 2–5: modeling dose–response RR of smoking on the selected health outcomes

Of the six steps proposed by Zheng et al.^29^, steps 2–5 cover the process of modeling dose–response risk curves. In step 2, we estimated the shape (or the ‘signal’) of the dose–response risk curves, integrating over different exposure ranges. To relax the log(linear) assumption usually applied to continuous dose–response risk and make the estimates robust to the placement of spline knots, we used an ensemble spline approach to fit the functional form of the dose–response relationship. The final ensemble model was a weighted combination of 50 models with random knot placement, with the weight of each model proportional to measures of model fit and total variation. To avoid the influence of extreme data and reduce publication bias, we trimmed 10% of data for each outcome as outliers. We also applied a monotonicity constraint to ensure that the mean risk curves were nondecreasing (or nonincreasing in the case of Parkinson’s disease).

In step 3, following the GRADE approach^192,193^, we quantified risk of bias across six domains, namely, representativeness of the study population, exposure, outcome, reverse causation, control for confounding and selection bias. Details about the bias covariates are provided in Supplementary Table 4. We systematically tested for the effect of bias covariates using metaregression, selected significant bias covariates using the Lasso approach^194,195^ and adjusted for the selected bias covariates in the final risk curve.

In step 4, we quantified between-study heterogeneity accounting for within-study correlation, uncertainty of the heterogeneity, as well as small number of studies. Specifically, we used a random intercept in the mixed-effects model to account for the within-study correlation and used a study-specific random slope with respect to the ‘signal’ to capture between-study heterogeneity. As between-study heterogeneity can be underestimated or even zero when the number of studies is small^196,197^, we used Fisher’s information matrix to estimate the uncertainty of the heterogeneity^198^ and incorporated that uncertainty into the final results.

In step 5, in addition to generating funnel plots and visually inspecting for asymmetry (Figs. 1c, 2c, 3c and 4c and Extended Data Fig. 6c) to identify potential publication bias, we also statistically tested for potential publication or reporting bias using Egger’s regression^199^. We flagged potential publication bias in the data but did not correct for it, which is in line with the general literature^10,200,201^. Full details about the modeling process have been published elsewhere^29^ and model specifications for each outcome are in Supplementary Table 6.

### Step 6: estimating the mean risk function and the BPRF

In the final step, step 6, the metaregression model inclusive of the selected bias covariates from step 3 (for example, the highest adjustment level) was used to predict the mean risk function and its 95% UI, which incorporated the uncertainty of the mean effect, between-study heterogeneity and the uncertainty in the heterogeneity estimate accounting for small numbers of studies. Specifically, 1,000 draws were created for each 0.1 level of doses from 0 pack-years to 100 pack-years or cigarette-equivalents smoked per day using the Bayesian metaregression model. The mean of the 1,000 draws was used to estimate the mean risk at each exposure level, and the 25th and 95th draws were used to estimate the 95% UIs for the mean risk at each exposure level.

The BPRF^29^ is a conservative estimate of risk function consistent with the available evidence, correcting for both between-study heterogeneity and systemic biases related to study characteristics. The BPRF is defined as either the 5th (if harmful) or 95th (if protective) quantile curve closest to the line of log(RR) of 0, which defines the null (Figs. 1a, 2b, 3a and 4a). The BPRF represents the smallest harmful (or protective) effect of smoking on the corresponding outcome at each level of exposure that is consistent with the available evidence. A BPRF opposite null from the mean risk function indicates that insufficient evidence is available to reject null, that is, that there may not be an association between risk and outcome. Likewise, the further the BPRF is from null on the same side of null as the mean risk function, the higher the magnitude and evidence for the relationship. The BPRF can be interpreted as indicating that, even accounting for between-study heterogeneity and its uncertainty, the log(RR) across the studied smoking range is at least as high as the BPRF (or at least as low as the BPRF for a protective risk).

To quantify the strength of the evidence, we calculated the ROS for each smoking–outcome association as the signed value of the log(BPRF) averaged between the 15th and 85th percentiles of observed exposure levels for each outcome. The ROS is a single summary of the effect of smoking on the outcome, with higher positive ROSs corresponding to stronger and more consistent evidence and a higher average effect size of smoking and a negative ROS, suggesting that, based on the available evidence, there is no significant effect of smoking on the outcome after accounting for between-study heterogeneity.

For ease of communication, we further classified each smoking–outcome association into a star rating from 1 to 5. Briefly, 1-star associations have an ROS <0, indicating that there is insufficient evidence to find a significant association between smoking and the selected outcome. We divided the positive ROSs into ranges 0.0–0.14 (2-star), >0.14–0.41 (3-star), >0.41–0.62 (4-star) and >0.62 (5-star). These categories correspond to excess risk ranges for harmful risks of 0–15%, >15–50%, >50–85% and >85%. For protective risks, the ranges of exposure-averaged decreases in risk by star rating are 0–13% (2 stars), >13–34% (3 stars), >34–46% (4 stars) and >46% (5 stars).

Among the 36 smoking–outcome pairs analyzed, smoking fracture was the only binary risk–outcome pair, which was due to limited data on the dose–response risk of smoking on fracture^202^. The estimation of binary risk was simplified because the RR was merely a comparison between current smokers and nonsmokers or never smokers. The concept of ROS for continuous risk can naturally extend to binary risk because the BPRF is still defined as the 5th percentile of the effect size accounting for data uncertainty and between-study heterogeneity. However, binary ROSs must be divided by 2 to make them comparable with continuous ROSs, which were calculated by averaging the risk over the range between the 15th and the 85th percentiles of observed exposure levels. Full details about estimating mean risk functions, BPRFs and ROSs for both continuous and binary risk–outcome pairs can be found elsewhere^29^.

### Estimating the age-specific risk function for CVD outcomes

For non-CVD outcomes, we assumed that the risk function was the same for all ages and all sexes, except for breast, cervical and prostate cancer, which were assumed to apply only to females or males, respectively. As the risk of smoking on CVD outcomes is known to attenuate with increasing age^203–206^, we adopted a four-step approach for GBD 2020 to produce age-specific dose–response risk curves for CVD outcomes.

First, we estimated the reference dose–response risk of smoking for each CVD outcome using dose-specific RR data for each outcome regardless of the age group information. This step was identical to that implemented for the other non-CVD outcomes. Once we had generated the reference curve, we determined the age group associated with it by calculating the weighted mean age across all dose-specific RR data (weighted by the reciprocal of the s.e.m. of each datum). For example, if the weighted mean age of all dose-specific RR data was 56.5, we estimated the age group associated with the reference risk curve to be aged 55–59 years. For cohort studies, the age range associated with the RR estimate was calculated as a mean age at baseline plus the mean/median years of follow-up (if only the maximum years of follow-up were reported, we would halve this value and add it to the mean age at baseline). For case–control studies, the age range associated with the OR estimate was simply the reported mean age at baseline (if mean age was not reported, we used the midpoint of the age range instead).

In the third step, we extracted age group-specific RR data and relevant bias covariates from the studies identified in our systematic review approach of age-specific smoking risk on CVD outcomes, and used MR-BRT to model the age pattern of excess risk (that is, RR-1) of smoking on CVD outcomes with age group-specific excess RR data for all CVD outcomes. We modeled the age pattern of smoking risk on CVDs following the same steps we implemented for modeling dose–response risk curves. In the final model, we included a spline on age, random slope on age by study and the bias covariate encoding exposure definition (that is, current, former and ever smokers), which was picked by the variable selection algorithm^28,29^. When predicting the age pattern of the excess risk of smoking on CVD outcomes using the fitted model, we did not include between-study heterogeneity to reduce uncertainty in the prediction.

In the fourth step, we calculated the age attenuation factors of excess risk compared with the reference age group for each CVD outcome as the ratio of the estimated excess risk for each age group to the excess risk for the reference age group. We performed the calculation at the draw level to obtain 1,000 draws of the age attenuation factors for each age group. Once we had estimated the age attenuation factors, we carried out the last step, which consisted of adjusting the risk curve for the reference age group from step 1 using equation (1) to produce the age group-specific risk curves for each CVD outcome:

1 $$\begin{array}{rcl}{\mathrm{RR}}_{{\mathrm{age}}_i} & = & ({\mathrm{RR}}_{{\mathrm{ref}}} - 1) \times {\mathrm{AF}}_{{\mathrm{age}}_i} + 1 \\{\mathrm{RR}}_{{\mathrm{age}}_i} & = & {\mathrm{Relative}}\, {\mathrm{risk}}\, {\mathrm{at}}\, {\mathrm{age}}\, {\mathrm{group}}\, {\mathrm{i}}\\{\mathrm{RR}}_{\mathrm{ref}} & = & {\mathrm{Risk}}\, {\mathrm{curve}}\, {\mathrm{at}}\, {\mathrm{the}}\, {\mathrm{reference}}\, {\mathrm{age}}\, {\mathrm{group}}\\{\mathrm{AF}}_{{\mathrm{age}}_i} & = & {\mathrm{Age}}\, {\mathrm{attenuation}}\, {\mathrm{factor}}\, {\mathrm{for}}\, {\mathrm{age}}\, {\mathrm{group}}\, {\mathrm{i}}\end{array}$$

We implemented the age adjustment at the draw level so that the uncertainty of the age attenuation factors could be naturally incorporated into the final adjusted age-specific RR curves. A PRISMA diagram detailing the systematic review approach, a description of the studies included and the full details about the methods are in Supplementary Information 1.5 and 5.2.

### Estimating the theoretical minimum risk exposure level

The theoretical minimum risk exposure level for smoking was 0, that is, no individuals in the population are current or former smokers.

### Model validation

The validity of the meta-analytic tool has been extensively evaluated by Zheng and colleagues using simulation experiments^28,29^. For the present study, we conducted two additional sensitivity analyses to examine how the shape of the risk curves was impacted by applying a monotonicity constraint and trimming 10% of data. We present the results of these sensitivity analyses in Supplementary Information 6. In addition to the sensitivity analyses, the dose–response risk estimates were also validated by plotting the mean risk function along with its 95% UI against both the extracted dose-specific RR data from the studies included and our previous dose–response risk estimates from the GBD 2019 (ref. ^30^). The mean risk functions along with the 95% UIs were validated based on data fit and the level, shape and plausibility of the dose–response risk curves. All curves were validated by all authors and reviewed by an external expert panel, comprising professors with relevant experience from universities including Johns Hopkins University, Karolinska Institute and University of Barcelona; senior scientists working in relevant departments at the WHO and the Center for Disease Control and Prevention (CDC) and directors of nongovernmental organizations such as the Campaign for Tobacco-Free Kids.

### Statistical analysis

Analyses were carried out using R v.3.6.3, Python v.3.8 and Stata v.16.

### Statistics and reproducibility

The study was a secondary analysis of existing data involving systematic reviews and meta-analyses. No statistical method was used to predetermine sample size. As the study did not involve primary data collection, randomization and blinding, data exclusions were not relevant to the present study, and, as such, no data were excluded and we performed no randomization or blinding. We have made our data and code available to foster reproducibility.

### Reporting summary

Further information on research design is available in the Nature Research Reporting Summary linked to this article.

## Online content

Any methods, additional references, Nature Research reporting summaries, extended data, supplementary information, acknowledgements, peer review information; details of author contributions and competing interests; and statements of data and code availability are available at 10.1038/s41591-022-01978-x.

## Supplementary information

Supplementary Information Supplementary Information 1: Data source identification and assessment. Supplementary Information 2: Data inputs. Supplementary Information 3: Study quality and bias assessment. Supplementary Information 4: The dose–response RR curves and their 95% UIs for all smoking–outcome pairs. Supplementary Information 5: Supplementary methods. Supplementary Information 6: Sensitivity analysis. Supplementary Information 7: Binary smoking–outcome pair. Supplementary Information 8: Risk curve details. Supplementary Information 9: GATHER and PRISMA checklists.

Reporting summary

## Data Availability

The findings from the present study are supported by data available in the published literature. Data sources and citations for each risk–outcome pair can be downloaded using the ‘download’ button on each risk curve page currently available at https://vizhub.healthdata.org/burden-of-proof. Study characteristics and citations for all input data used in the analyses are also provided in Supplementary Table 3, and Supplementary Table 2 provides a template of the data collection form.

## Extended data

are available for this paper at 10.1038/s41591-022-01978-x.

## Code availability

All code used for these analyses is publicly available online (https://github.com/ihmeuw-msca/burden-of-proof).
