# Supplementary material for: Health effects associated with smoking: a Burden of Proof study
Source: Nat Med. 2022 Oct 10;28(10):2045–55. doi: 10.1038/s41591-022-01978-x (PMC9556318; doi:10.1038/s41591-022-01978-x)
Supplement: Supplementary file 1 — Supplementary Information 1: Data source identification and assessment. Supplementary Information 2: Data inputs. Supplementary Information 3: Study quality and bias assessment. Supplementary Information 4: The dose–response RR curves and their 95% UIs for all smoking–outcome pairs. Supplementary Information 5: Supplementary methods. Supplementary Information 6: Sensitivity analysis. Supplementary Information 7: Binary smoking–outcome pair. Supplementary Information 8: Risk curve details. Supplementary Information 9: GATHER and PRISMA checklists. [file 41591_2022_1978_MOESM1_ESM.pdf]

nature portfolio

https://doi.org/10.1038/s41591-022-01978-x

Supplementary information

Health effects associated with smoking: a Burden of Proof study

In the format provided by the authors and unedited

Supplementary Information: data sources and supplementary results for "Health effects associated with smoking: a Burden of Proof study"

This appendix provides detailed information on input data sources and supplementary results for "Health effects associated with smoking: a Burden of Proof Study."

Table of Contents

Section 1: Data source identification and assessment 2

Section 2: Data inputs 44

Section 3: Study quality & bias assessment 172

Section 4: The dose-response RR curves and their 95% uncertainty interval for all smoking-outcome pairs 193

Section 5: Supplementary methods 202

Section 6: Sensitivity analysis 211

Section 7: Binary smoking-outcome pair (i.e., smoking-fracture) 230

Section 8: Risk curve details 232

Section 9: GATHER and PRISMA checklists 295

Section 1: Data source identification and assessment

The data in this systematic review includes effect sizes from cohort studies and case-control studies. Age-stratified effect sizes from cross-sectional studies were used to inform age-attenuated curves for cardiovascular outcomes. More detailed information on the data is available at http://vizhub.healthdata.org/burden-of-proof/.

Section 1.1: Literature studies

We conducted literature searches to obtain input data on the relationship between current smoking behavior and our 36 target outcomes. We also searched the citation lists of systematic reviews identified during the literature searches to identify primary research articles that may not have been captured with the original search string.

Section 1.1.1: PubMed search

Literature search updates were performed on PubMed on 6/21/2019, 7/2/2019-7/18/2019 and captured studies published since the previous literature search up to 5/1/2019. Another update was conducted in June 2022, capturing studies from 5/1/2019 through 31/5/2022.

Section 1.1.2: Search strings

Subarachnoid Hemorrhage

("Smoking"[Mesh] OR "smoking"[tiab]) AND ("Subarachnoid Hemorrhage"[Mesh] OR "subarachnoid hemorrhage"[tiab] OR "sah"[tiab]) AND ("Risk"[Mesh] OR "relative risk"[tiab] OR "hazard ratio"[tiab] OR "odds ratio"[tiab] OR "rate"[tiab] OR "risk"[tiab]) AND ("Cohort Studies"[Mesh] OR "Prospective Studies"[Mesh] OR "Cohort"[tiab] OR "prospective"[tiab] OR "longitudinal"[tiab] OR "Case-Control Studies"[Mesh] OR "case-control"[tiab] OR "case control"[tiab] OR "case-crossover"[tiab]) AND ("2018/05/01"[PDAT] : "2022/05/31"[PDAT]) NOT (animals[MeSH] NOT humans[MeSH])

Stroke

("Smoking"[Mesh] OR "smoking"[tiab]) AND ("Stroke"[Mesh] OR "stroke"[tiab] OR "cva"[tiab] OR "cerebrovascular"[tiab] OR "hemorrhage"[tiab] OR "haemorrhage"[tiab] OR "aneurism"[tiab] OR "aneurysm"[tiab]) AND ("Risk"[Mesh] OR "relative risk"[tiab] OR "hazard ratio"[tiab] OR "odds ratio"[tiab] OR "rate"[tiab] OR "risk"[tiab]) AND ("Cohort Studies"[Mesh] OR "Prospective Studies"[Mesh] OR "Cohort"[tiab] OR "prospective"[tiab] OR "longitudinal"[tiab] OR "Case-Control Studies"[Mesh] OR "case-control"[tiab] OR "case control"[tiab] OR "case-crossover"[tiab]) AND ("2018/05/01"[PDAT] : "2022/05/31"[PDAT]) NOT (animals[MeSH] NOT humans[MeSH])

Atrial Fibrillation and Flutter

("Smoking"[Mesh] OR "smoking"[tiab]) AND ("Atrial Fibrillation"[Mesh] OR "Atrial Flutter"[Mesh] OR "atrial fibrillation"[tiab] OR "atrial flutter"[tiab]) AND ("Risk"[Mesh] OR "relative risk"[tiab] OR "hazard ratio"[tiab] OR "odds ratio"[tiab] OR "rate"[tiab] OR "risk"[tiab]) AND ("Cohort Studies"[Mesh] OR "Prospective Studies"[Mesh] OR "Cohort"[tiab] OR "prospective"[tiab] OR "longitudinal"[tiab] OR "Case-Control Studies"[Mesh] OR "case-control"[tiab] OR "case control"[tiab] OR "case-crossover"[tiab]) AND ("2018/05/01"[PDAT] : "2022/05/31"[PDAT]) NOT (animals[MeSH] NOT humans[MeSH])

Diabetes

("Smoking"[Mesh] OR "smoking"[tiab]) AND ("Diabetes Mellitus, Type 1"[Mesh] OR "Diabetes Mellitus, Type 2"[Mesh] OR "diabetes"[ti]) AND ("Risk"[Mesh] OR "relative risk"[tiab] OR "hazard ratio"[tiab] OR "odds ratio"[tiab] OR "rate"[tiab] OR "risk"[tiab]) AND ("Cohort Studies"[Mesh] OR "Prospective Studies"[Mesh] OR "Cohort"[tiab] OR "prospective"[tiab] OR "longitudinal"[tiab] OR "Case-Control Studies"[Mesh] OR "case-control"[tiab] OR "case control"[tiab] OR "case-crossover"[tiab]) AND ("2018/05/01"[PDAT] : "2022/05/31"[PDAT]) NOT (animals[MeSH] NOT humans[MeSH])

Lower respiratory infections

("Smoking"[Mesh] OR "smoking"[tiab]) AND ("respiratory tract infections"[MeSH] OR "lower respiratory"[tiab] OR "pneumonia"[tiab] OR "bronchiolitis"[tiab]) AND ("Risk"[Mesh] OR "relative risk"[tiab] OR "hazard ratio"[tiab] OR "odds ratio"[tiab] OR "rate"[tiab] OR "risk"[tiab]) AND ("Cohort Studies"[Mesh] OR "Prospective Studies"[Mesh] OR "Cohort"[tiab] OR "prospective"[tiab] OR "longitudinal"[tiab] OR "Case-Control Studies"[Mesh] OR "case-control"[tiab] OR "case control"[tiab] OR "case-crossover"[tiab]) AND ("2018/05/01"[PDAT] : "2022/05/31"[PDAT]) NOT (animals[MeSH] NOT humans[MeSH])

Low back pain

("Smoking"[Mesh] OR "smoking"[tiab]) AND ("Low Back Pain"[Mesh] OR "low back pain"[tiab]) AND ("Risk"[Mesh] OR "relative risk"[tiab] OR "hazard ratio"[tiab] OR "odds ratio"[tiab] OR "rate"[tiab] OR "risk"[tiab]) AND ("Cohort Studies"[Mesh] OR "Prospective Studies"[Mesh] OR "Cohort"[tiab] OR "prospective"[tiab] OR "longitudinal"[tiab] OR "Case-Control Studies"[Mesh] OR "case-control"[tiab] OR "case control"[tiab] OR "case-crossover"[tiab]) AND ("2018/05/01"[PDAT] : "2022/05/31"[PDAT]) NOT (animals[MeSH] NOT humans[MeSH])

Parkinson's disease

("Smoking"[Mesh] OR "smoking"[tiab]) AND ("Parkinson Disease"[Mesh] OR "parkinson"[tiab]) AND ("Risk"[Mesh] OR "relative risk"[tiab] OR "hazard ratio"[tiab] OR "odds ratio"[tiab] OR "rate"[tiab] OR "risk"[tiab]) AND ("Cohort Studies"[Mesh] OR "Prospective Studies"[Mesh] OR "Cohort"[tiab] OR "prospective"[tiab] OR "longitudinal"[tiab] OR "Case-Control Studies"[Mesh] OR "case-control"[tiab] OR "case control"[tiab] OR "case-crossover"[tiab]) AND ("2018/05/01"[PDAT] : "2022/05/31"[PDAT]) NOT (animals[MeSH] NOT humans[MeSH])

Macular degeneration

("Smoking"[Mesh] OR "smoking"[tiab]) AND ("Macular Degeneration"[Mesh] OR "macular degeneration"[tiab]) AND ("Risk"[Mesh] OR "relative risk"[tiab] OR "hazard ratio"[tiab] OR "odds ratio"[tiab] OR "rate"[tiab] OR "risk"[tiab]) AND ("Cohort Studies"[Mesh] OR "Prospective Studies"[Mesh] OR "Cohort"[tiab] OR "prospective"[tiab] OR "longitudinal"[tiab] OR "Case-Control Studies"[Mesh] OR "case-control"[tiab] OR "case control"[tiab] OR "case-crossover"[tiab]) AND ("2018/05/01"[PDAT] : "2022/05/31"[PDAT]) NOT (animals[MeSH] NOT humans[MeSH])

Multiple sclerosis

("Smoking"[Mesh] OR "smoking"[tiab]) AND ("Multiple Sclerosis"[Mesh] OR "multiple sclerosis"[tiab]) AND ("Risk"[Mesh] OR "relative risk"[tiab] OR "hazard ratio"[tiab] OR "odds ratio"[tiab] OR "rate"[tiab] OR "risk"[tiab]) AND ("Cohort Studies"[Mesh] OR "Prospective Studies"[Mesh] OR "Cohort"[tiab] OR "prospective"[tiab] OR "longitudinal"[tiab] OR "Case-Control Studies"[Mesh] OR "case-control"[tiab] OR "case control"[tiab] OR "case-crossover"[tiab]) AND ("2018/05/01"[PDAT] : "2022/05/31"[PDAT]) NOT (animals[MeSH] NOT humans[MeSH])

Rheumatoid arthritis

("Smoking"[Mesh] OR "smoking"[tiab]) AND ("Arthritis, Rheumatoid"[Mesh] OR "rheumatoid"[tiab] OR "caplan syndrome"[tiab] OR "felty syndrome"[tiab] OR "sjogren"[tiab] OR "still's disease"[tiab]) AND ("Risk"[Mesh] OR "relative risk"[tiab] OR "hazard ratio"[tiab] OR "odds ratio"[tiab] OR "rate"[tiab] OR "risk"[tiab]) AND ("Cohort Studies"[Mesh] OR "Prospective Studies"[Mesh] OR "Cohort"[tiab] OR "prospective"[tiab] OR "longitudinal"[tiab] OR "Case-Control Studies"[Mesh] OR "case-control"[tiab] OR "case control"[tiab] OR "case-crossover"[tiab]) AND ("2018/05/01"[PDAT] : "2022/05/31"[PDAT]) NOT (animals[MeSH] NOT humans[MeSH])

Peptic ulcer disease

("Smoking"[Mesh] OR "smoking"[tiab]) AND ("Peptic Ulcer"[Mesh] OR "peptic ulcer"[tiab] OR "stomach ulcer"[tiab]) AND ("Risk"[Mesh] OR "relative risk"[tiab] OR "hazard ratio"[tiab] OR "odds ratio"[tiab] OR

"rate"[tiab] OR "risk"[tiab]) AND ("Cohort Studies"[Mesh] OR "Prospective Studies"[Mesh] OR "Cohort"[tiab] OR "prospective"[tiab] OR "longitudinal"[tiab] OR "Case-Control Studies"[Mesh] OR "case-control"[tiab] OR "case control"[tiab] OR "case-crossover"[tiab]) AND ("2018/05/01"[PDAT] : "2022/05/31"[PDAT]) NOT (animals[MeSH] NOT humans[MeSH])

Ischemic heart disease

("Smoking"[Mesh] OR "smoking"[tiab]) AND ("Myocardial Ischemia"[Mesh] OR "heart disease"[tiab] OR "coronary"[tiab] OR "myocardial infarction"[tiab] OR "heart attack"[tiab] OR "heart disease"[tiab]) AND ("Risk"[Mesh] OR "relative risk"[tiab] OR "hazard ratio"[tiab] OR "odds ratio"[tiab] OR "rate"[tiab] OR "risk"[tiab]) AND ("Cohort Studies"[Mesh] OR "Prospective Studies"[Mesh] OR "Cohort"[tiab] OR "prospective"[tiab] OR "longitudinal"[tiab] OR "Case-Control Studies"[Mesh] OR "case-control"[tiab] OR "case control"[tiab] OR "case-crossover"[tiab]) AND ("2018/05/01"[PDAT] : "2022/05/31"[PDAT]) NOT (animals[MeSH] NOT humans[MeSH])

Tuberculosis

("Smoking"[Mesh] OR "smoking"[tiab]) AND ("Tuberculosis"[Mesh] OR "tuberculosis"[tiab] OR "TB"[tiab]) AND ("Risk"[Mesh] OR "relative risk"[tiab] OR "hazard ratio"[tiab] OR "odds ratio"[tiab] OR "rate"[tiab] OR "risk"[tiab]) AND ("Cohort Studies"[Mesh] OR "Prospective Studies"[Mesh] OR "Cohort"[tiab] OR "prospective"[tiab] OR "longitudinal"[tiab] OR "Case-Control Studies"[Mesh] OR "case-control"[tiab] OR "case control"[tiab] OR "case-crossover"[tiab]) AND ("2018/05/01"[PDAT] : "2022/05/31"[PDAT]) NOT (animals[MeSH] NOT humans[MeSH])

Gallbladder

("Smoking"[Mesh] OR "smoking"[tiab]) AND ("Biliary Tract Diseases"[Mesh] OR "gallbladder"[tiab] OR "biliary"[tiab]) AND ("Risk"[Mesh] OR "relative risk"[tiab] OR "hazard ratio"[tiab] OR "odds ratio"[tiab] OR "rate"[tiab] OR "risk"[tiab]) AND ("Cohort Studies"[Mesh] OR "Prospective Studies"[Mesh] OR "Cohort"[tiab] OR "prospective"[tiab] OR "longitudinal"[tiab] OR "Case-Control Studies"[Mesh] OR "case-control"[tiab] OR "case control"[tiab] OR "case-crossover"[tiab]) AND ("2018/05/01"[PDAT] : "2022/05/31"[PDAT]) NOT (animals[MeSH] NOT humans[MeSH])

Fractures

("Smoking"[Mesh] OR "smoking"[tiab]) AND ("Fractures, Bone"[Mesh] OR "fracture"[tiab]) AND ("Risk"[Mesh] OR "relative risk"[tiab] OR "hazard ratio"[tiab] OR "odds ratio"[tiab] OR "rate"[tiab] OR "risk"[tiab]) AND ("Cohort Studies"[Mesh] OR "Prospective Studies"[Mesh] OR "Cohort"[tiab] OR "prospective"[tiab] OR "longitudinal"[tiab] OR "Case-Control Studies"[Mesh] OR "case-control"[tiab] OR "case control"[tiab] OR "case-crossover"[tiab]) AND ("2018/05/01"[PDAT] : "2022/05/31"[PDAT]) NOT (animals[MeSH] NOT humans[MeSH])

Dementia

("Smoking"[Mesh] OR "smoking"[tiab]) AND ("Dementia"[Mesh] OR "dementia"[tiab] OR "alzheimer"[tiab] OR "huntington"[tiab] OR "kluver-bucy"[tiab] OR "lewy body"[tiab] OR "creutzfeldt-jakob"[tiab] OR "aphasia"[tiab]) AND ("Risk"[Mesh] OR "relative risk"[tiab] OR "hazard ratio"[tiab] OR "odds ratio"[tiab] OR "rate"[tiab] OR "risk"[tiab]) AND ("Cohort Studies"[Mesh] OR "Prospective Studies"[Mesh] OR "Cohort"[tiab] OR "prospective"[tiab] OR "longitudinal"[tiab] OR "Case-Control Studies"[Mesh] OR "case-control"[tiab] OR "case control"[tiab] OR "case-crossover"[tiab]) AND ("2018/05/01"[PDAT] : "2022/05/31"[PDAT]) NOT (animals[MeSH] NOT humans[MeSH])

Chronic obstructive pulmonary disease

("Smoking"[Mesh] OR "smoking"[tiab]) AND ("Pulmonary Disease, Chronic Obstructive"[Mesh] OR "COPD"[tiab] OR "emphysema"[tiab] OR "chronic obstructive pulmonary disease"[tiab]) AND ("Risk"[Mesh] OR "relative risk"[tiab] OR "hazard ratio"[tiab] OR "odds ratio"[tiab] OR "rate"[tiab] OR "risk"[tiab]) AND ("Cohort

Studies"[Mesh] OR "Prospective Studies"[Mesh] OR "Cohort"[tiab] OR "prospective"[tiab] OR "longitudinal"[tiab] OR "Case-Control Studies"[Mesh] OR "case-control"[tiab] OR "case control"[tiab] OR "case-crossover"[tiab]) AND ("2018/05/01"[PDAT] : "2022/05/31"[PDAT]) NOT (animals[MeSH] NOT humans[MeSH])

Cataracts

("Smoking"[Mesh] OR "smoking"[tiab]) AND ("Cataract"[Mesh] OR "cataract"[tiab] OR "opacity"[tiab] OR "pseudophakia"[tiab]) AND ("Risk"[Mesh] OR "relative risk"[tiab] OR "hazard ratio"[tiab] OR "odds ratio"[tiab] OR "rate"[tiab] OR "risk"[tiab]) AND ("Cohort Studies"[Mesh] OR "Prospective Studies"[Mesh] OR "Cohort"[tiab] OR "prospective"[tiab] OR "longitudinal"[tiab] OR "Case-Control Studies"[Mesh] OR "case-control"[tiab] OR "case control"[tiab] OR "case-crossover"[tiab]) AND ("2018/05/01"[PDAT] : "2022/05/31"[PDAT]) NOT (animals[MeSH] NOT humans[MeSH])

Cancers

("Smoking"[Mesh] OR "smoking"[tiab]) AND ("Neoplasms"[Mesh] OR "cancer"[tiab] OR "neoplasm"[tiab] OR "carcinoma"[tiab] OR "leukemia"[tiab]) AND ("Risk"[Mesh] OR "relative risk"[tiab] OR "hazard ratio"[tiab] OR "odds ratio"[tiab] OR "rate"[tiab] OR "risk"[tiab]) AND ("Cohort Studies"[Mesh] OR "Prospective Studies"[Mesh] OR "Cohort"[tiab] OR "prospective"[tiab] OR "longitudinal"[tiab] OR "Case-Control Studies"[Mesh] OR "case-control"[tiab] OR "case control"[tiab] OR "case-crossover"[tiab]) AND ("2018/05/01"[PDAT] : "2022/05/31"[PDAT]) NOT (animals[MeSH] NOT humans[MeSH])

Asthma

("Smoking"[Mesh] OR "smoking"[tiab]) AND ("Asthma"[Mesh] OR "asthma"[tiab]) AND ("Risk"[Mesh] OR "relative risk"[tiab] OR "hazard ratio"[tiab] OR "odds ratio"[tiab] OR "rate"[tiab] OR "risk"[tiab]) AND ("Cohort Studies"[Mesh] OR "Prospective Studies"[Mesh] OR "Cohort"[tiab] OR "prospective"[tiab] OR "longitudinal"[tiab] OR "Case-Control Studies"[Mesh] OR "case-control"[tiab] OR "case control"[tiab] OR "case-crossover"[tiab]) AND ("2018/05/01"[PDAT] : "2022/05/31"[PDAT]) NOT (animals[MeSH] NOT humans[MeSH])

Aortic aneurysm

("Smoking"[Mesh] OR "smoking"[tiab]) AND ("Aortic Aneurysm"[MESH] OR "aortic aneurysm"[tiab]) AND ("Risk"[Mesh] OR "relative risk"[tiab] OR "hazard ratio"[tiab] OR "odds ratio"[tiab] OR "rate"[tiab] OR "risk"[tiab]) AND ("Cohort Studies"[Mesh] OR "Prospective Studies"[Mesh] OR "Cohort"[tiab] OR "prospective"[tiab] OR "longitudinal"[tiab] OR "Case-Control Studies"[Mesh] OR "case-control"[tiab] OR "case control"[tiab] OR "case-crossover"[tiab]) AND ("2018/05/01"[PDAT] : "2022/05/31"[PDAT]) NOT (animals[MeSH] NOT humans[MeSH])

Section 1.2: Inclusion & Exclusion criteria

Of those articles captured by each search string, prospective cohort and case-control studies were included if they reported the effect sizes (relative risk, hazard ratio, or odds ratio) of an association between a continuous or categorical dose for smoked tobacco consumption and a GBD outcome with uncertainty. Studies were excluded if they used cross-sectional or retrospective cohort design or if the study was conducted among specific high-risk populations (e.g., people with diabetes or drug users, etc.). For systematic review studies, we extracted data from each underlying study unless data extraction from the underlying studies was not possible, in which case we extracted the pooled estimates from the systematic review.

Section 1.3: Outcome definitions

Table 1: Definitions of 36 included outcomes

Cause Name	Definition
Alzheimer's disease and related dementias	Dementia is a progressive, degenerative, and chronic neurological disorder typified by cognitive dysfunctions that interfere with daily functioning. We use the Diagnostic and Statistical Manual of Mental Disorders III, IV, or V, or ICD case definitions as the reference.

Aortic aneurysm	Full-thickness dilation of the aorta, usually due to atherosclerosis, elevated blood pressure, or inflammation of the blood vessel.
Asthma	The onset of asthma as measured by a reported doctor's diagnosis and wheezing in the past 12 months
Atrial fibrillation and flutter	Diagnosis based on ECG findings, including:<br>1) irregularly irregular RR intervals (in the absence of complete atrio-ventricular block);<br>2) no distinct P waves on the surface ECG, and;<br>3) an atrial cycle length (when visible) that is usually variable and less than 200 milliseconds.
Bladder cancer	Malignant neoplasm of the bladder
Breast cancer	Malignant neoplasm of the breast
Cataracts	Blindness (acuity in the better eye of <3/60 or <10% visual field around central fixation point) from cataract. Presenting vision is measured using any corrective lenses currently in use.
Cervical cancer	Malignant neoplasm of the cervix
Colorectal cancer	Malignant neoplasm of the colon and rectum
COPD	Cases diagnosed based on GOLD criteria, i.e. spirometry results of FEV1/FVC <0.7 post-bronchodilator.
Esophageal cancer	Malignant neoplasm of the esophagus
Fracture	Hip or non-hip bone fractures related to ICD N-codes
Gallbladder diseases	Inflammation of the gallbladder and/or bile duct identified through ultrasound diagnosis
Ischemic heart disease	Acute myocardial infarction (MI): definite and possible MI according to the third universal definition of myocardial infarction; includes recurrent cases and cases who died before reaching medical care
Kidney cancer	Malignant neoplasm of the kidney
Laryngeal cancer	Malignant neoplasm of the larynx
Leukemia	Malignant neoplasm of the Blood and Bone Marrow (Leukemia)
Lip and oral cavity cancer	Malignant neoplasm of the lips and oral cavity
Liver cancer	Malignant neoplasm of the liver
Low back pain	Current low back pain (with or without pain referred into one or both lower limbs) that lasts for at least one day. The “low back” is defined as the area on the posterior aspect of the body from the lower margin of the twelfth ribs to the lower gluteal folds
Lower respiratory tract infections	Clinician-diagnosed episode of pneumonia or bronchiolitis.
Lung cancer	Malignant neoplasm of the trachea, bronchus, and lungs
Macular degeneration	Blindness (presenting visual acuity in the better eye of <3/60 or <10% visual field around central fixation point) due to macular degeneration, which is a deterioration of the macula, the part of the retina responsible for central vision. Presenting vision is measured with any corrective lenses currently in use.
Multiple sclerosis	Multiple sclerosis is a chronic, degenerative, and progressive neurological condition typified by the damaging of the myelin sheaths of nerves.
Nasopharyngeal cancer	Malignant neoplasm of the nasopharynx
Other pharynx cancer	Malignant neoplasm of the pharynx
Pancreatic cancer	Malignant neoplasm of the pancreas
Parkinson's disease	New cases according to Gelb criteria (presence of two out of four cardinal symptoms: rest tremor, bradykinesia, rigidity, unilateral onset).
Peptic ulcer disease	Disease characterized by defects in the lining of the stomach or duodenum that extend through the muscularis mucosa into deeper layers and which have caused symptoms or complications (hemorrhage, perforation, obstruction) sufficient to prompt an individual with access to seek care in the last 12 months and leading to diagnosis by a healthcare provider. This can include individuals in an asymptomatic period following one or more symptomatic periods or complications

Peripheral artery disease	Peripheral arterial disease diagnosed by an Ankle-Brachial Index <0.9.
Prostate cancer	Malignant neoplasm of the prostate
Rheumatoid arthritis	Four of the 1987 criteria by the American College of Rheumatology (ACR 1987), which stipulate seven diagnostic criteria, need to be satisfied for a diagnosis.
Stomach cancer	Malignant neoplasm of the stomach
Stroke	Stroke was defined according to WHO criteria of rapidly developing clinical signs of usually focal disturbance of cerebral function lasting more than 24 h or leading to death.
Tuberculosis	All-forms of Tuberculosis including pulmonary TB that is bacteriologically confirmed (smear and/or culture confirmed) and extrapulmonary TB that is bacteriologically confirmed or clinically diagnosed.
Diabetes	Fasting plasma glucose greater or equal to 126 mg/dl (7 mmol/l) or current treatment (insulin or drugs).

Section 1.4: Data extraction

Table 2: Data extraction template

**Extracted variable (column name)**	**Extraction guidance provided**
extractor	id of person who extracted the data
seq	
underlying_nid	Underlying NID: Enter the underlying NID of the study (if applicable). Always talk to a data indexer if you don't know if an underlying NID is needed. They may be used for meta-analyses, certain database sources, and in some other specific cases.
nid	Found in GHDx, created through the epi form, or created by Data Indexer
field_citation_value	IHME Zotero format or if source has NID, citation info from GHDx
file_path	optional; full file path of article; Only needed if source doesn't have NID, to facilitate NID creation.
risk	Risk: Select the risk factor, if not listed here, contact the causal criteria team
risk_mapping	the relationship between study definition of risk and GBD definition of risk for a particular effect size
outcome	Outcome: Select the outcome.
outcome_mapping	the relationship between study definition of outcome and GBD definition of outcomefor a particular effect size
location_name	location name (from locations tab). Do a fast double-click in this field to get the drop-down menu, then start typing the location_name. For location_names with special characters, you may need to use the scroll bar.
location_id	autopopulated from location_name
rep_geography	Were the study partcipants representative of the geography? 1=yes, 0=no
rep_selection_criteria	If rep_geography is 0, please specify the selection criteria of the study that is used in the analysis
rep_prevalent_disease	Is the study aiming to evaluate the risk or mortality of people who have already developed the outcome? 1=yes 0=no (i.e. yes if for SBP-IHD paper, all partcipants have IHD at baseline and the paper is looking at mortality due to SBP, no if for SBP-IHD paper the participants have other prevalent diseases)
year_start_study	year the study was started. If not specified, leave blank
year_end_study	year the study was finished (including most recent follow up). If not specified, leave blank
age_start	ages from 1 and above must be entered as an integer. Ages <1 can be entered as decimal values, e.g., 3 days = 3/365.

age_end	ages from 1 and above must be entered as an integer. Ages <1 can be entered as decimal values, e.g., 3 days = 3/365.
age_mean	Mean age
age_sd	SD of age
age_issue	0 = no issue flagged; 1 = issue flagged for modeler; always include explanatory notes the note_SR column
percent_male	what percent of the population is male (0-1), if pop is all female then it would be 0
sex_issue	sex_issue
design	Study design: Specify the design of the study
study_name	Study Name: Enter the name of the study (e.g., Nurses' Health Study), if provided. Do not enter the title of the article.
exp_assess_level	Level of exposure assessment: The exposure was assessed…
exp_instrument	Exposure assessment instrument: Specify the name of the exposure assessment instrument. For self-reported exposures, please specify the name of the questionnaire e.g., International Physical Activity Questionnaire (IPAQ). If more than one instrument specify all
exp_assess_period	What was the frequency of exposure assessment?
exp_assess_num	if multiple, specify the number of times that exposure was assessed (excluding baseline)
exp_method_1	Please specify the method of exposure assessment. If there are more than 1, please add in the next columns labeled ""exp_method_2"".
exp_method_2	Please specify the method of exposure assessment. If there are more than 2, please add in the next columns labeled ""exp_method_3"".
exp_method_3	Please specify the method of exposure assessment.
exp_recall_period	This field describes the unit of exposure recall used in data collection ONLY for self-report. Select the correct option from the drop-down menu. If the unit is days, weeks, months, or years, please enter the number in exp_recall_period_value (next column). If the unit is 'lifetime', nothing needs to be entered in exp_recall_period_value. For example, if the study said the recall period was 4 weeks, enter 4 in exp_recall_period_value, and 'weeks' in the field exp_recall_period. If 'other' is selected, please describe in exp_recall_period_other
exp_recall_period_value	If you entered days, weeks, months, or years in the field 'exp_recall_period', please enter the corresponding integer in this field. For example, if the study said the recall period was 4 weeks, enter 4 in exp_recall_period_value, and 'weeks' in the field exp_recall_period.
exp_recall_period_other	If 'other' was selected in exp_recall_period, please describe the exposure recall period that the study specified (e.g., recall of exposure from 12 to 18 years).
exp_type	Which form of the exposure was included in relative risk estimation analysis?
outcome_def	Outcome definition: Provide a brief description of the outcome as reported in the study.
outcome_type	Outcome type: please specify if the outcome definition included incidence of or mortality from a disease endpoint
outcome_assess_1	Method of outcome assessment: Specify the method of assessment of the study outcome. If more than 1 are appropriate, enter additional methods in the next column labeled ""outcome_assess_2""
outcome_assess_2	Method of outcome assessment: Specify the method of assessment of the study outcome. If more than 2 are appropriate, enter additional methods in the next column labeled ""outcome_assess_3""
outcome_assess_3	Method of outcome assessment: Specify the method of assessment of the study outcome.

duration_fup_measure	Type of follow up measure (i.e. mean, median, max, min)
duration_fup_units	Units of follow up duration
value_of_duration_fup	Enter the length of participant follow-up.
confounders_age	if controlled for in the relative risk estimation analysis, mark 1 for yes. Mark 0 for no
confounders_sex	if controlled for in the relative risk estimation analysis, mark 1 for yes. Mark 0 for no
confounders_education	if controlled for in the relative risk estimation analysis, mark 1 for yes. Mark 0 for no
confounders_income	if controlled for in the relative risk estimation analysis, mark 1 for yes. Mark 0 for no
confounders_smoking	if controlled for in the relative risk estimation analysis, mark 1 for yes. Mark 0 for no
confounders_alcohol_use	if controlled for in the relative risk estimation analysis, mark 1 for yes. Mark 0 for no
confounders_physical_activity	if controlled for in the relative risk estimation analysis, mark 1 for yes. Mark 0 for no
confounders_dietary_components	if controlled for in the relative risk estimation analysis, mark 1 for yes. Mark 0 for no
confounders_bmi	if controlled for in the relative risk estimation analysis, mark 1 for yes. Mark 0 for no
confounders_hypertension	if controlled for in the relative risk estimation analysis, mark 1 for yes. Mark 0 for no
confounders_diabetes	if controlled for in the relative risk estimation analysis, mark 1 for yes. Mark 0 for no
confounders_hypercholesterolemia	if controlled for in the relative risk estimation analysis, mark 1 for yes. Mark 0 for no
confounders_other	For other confounders that not listed, list here
page_num_effect_size	Page number (where you found effect_size) from literature, or survey question where you found effect size; Use page number(s) of article, not page # of pdf
custom_exp_level_lower_sign	can use a <. >, = etc in association with custom_exp_level_lower
custom_exp_level_lower	if don't have a mean/midpoint exposure level can use this column in conjecture with the custom_exp_level_upper to enter in a range
custom_exp_level_upper_sign	can use a <. >, = etc in association with custom_exp_level_upper
custom_exp_level_upper	if don't have a mean/midpoint exposure level can use this column in conjecture with the custom_exp_level_lower to enter in a range
custom_unexp_level_lower_sign	can use a <. >, = etc in association with custom_unexp_level_lower
custom_unexp_level_lower	if don't have a mean/midpoint exposure level can use this column in conjecture with the custom_unexp_level_upper to enter in a range
custom_unexp_level_upper_sign	can use a <. >, = etc in association with custom_unexp_level_upper
custom_unexp_level_upper	if don't have a mean/midpoint exposure level can use this column in conjecture with the custom_unexp_level_lower to enter in a range
effect_size_measure	Effect size measure: Specify the measure of effect size
effect_size	Effect size estimate: Provide the effect size estimate
lower	Provide the lower limit of the confidence interval. Enter on a ""per 1"" basis. (If the CI is reported as a percent, you must convert to a decimal.) These 3 fields must all be filled in if any of them are filled in: lower, upper, uncertainty_type_value.
upper	Provide the upper limit of the confidence interval. Enter on a ""per 1"" basis. (If the CI is reported as a percent, you must convert to a decimal.) These 3 fields must all be filled in if any of them are filled in: lower, upper, uncertainty_type_value.

CI_uncertainty_type_value	This field is required if 'lower' & 'upper' are entered. This column represents the confidence level which is reported at (Eg. 95, 90, 99). These 3 fields must all be filled in if any of them are filled in: lower, upper, uncertainty_type_value.
nonCI_uncertainty_value	Numerical value of the nonCI_uncertainty_type entered in that column. For example, if SD=5.3, you'd put 5.3 in this column, and choose SD from the drop down menu in nonCI_uncertainty_type.
nonCI_uncertainty_type	Enter SE or SD if appropriate. For example, if SD=5.3, you'd put 5.3 in nonCI_uncertainty_value, and choose SD from the drop down menu in this column (nonCI_uncertainty_type).
uncertainty_issue	Mark with a 1 if no uncertainty is reported, if some sort of uncertainty is reported, mark 0
custom_webplot_digitizer	1 if webplot digitizer was used to pull the effect size
subgroup_analysis	1 if RR is from main analysis (all participants), 0 if sub-analysis (only males, or among a specific age group, etc.)
subgroup_analysis_free_text	if a sub-analysis, describe it (i.e. age, sex, etc.)
effect_size_multi_location	1 if the reported effect size is from a multi-country study and only one effect size has been reported for all locations, otherwise 0
effect_size_multi_location_specify	which geography level is the RR for
pooled_cohort	1 if the reported effect size is from a pooled analysis and only pooled effect size has been reported, otherwise 0
dose_response	Does the study support a dose-response relationship between the exposure and the outcome? (1= yes, 0=no)
dose_response_detail	If ""1"" was specified in the dose_response field, please specify in this field the type of evidence supporting the dose-response relationship. For example, ""statistically significant p value for linear trend"".
cohort_person_years_exp	Please specify the person years of follow up in the exposed group
cohort_person_years_unexp	Please specify the person years of follow up in the unexposed group
cohort_person_years_total	Enter the total person-years of follow-up if person-years of follow up in exposed and unexposed not reported
cohort_number_events_exp	Please specify the number of events in the exposed group
cohort_number_events_unexp	Please specify the number of events in the unexposed group
cohort_number_events_total	Enter the total number of events/cases if number of events in exposed and unexpoxed not reported
cohort_sample_size_exp	Please specify the number of people in the exposed group if person-years of follow up in exposed not reported
cohort_sample_size_unexp	Please specify the number of people in the unexposed group if person-years of follow up in unexposed not reported
cohort_sample_size_total	Please specify the number of people included in the analysis if total person-years of follow up in not reported
cohort_dropout_rate	Dropout rate?: Specify the dropout rate (%) at the end of the study. Enter on a ""per 1"" basis. For example: 23% is entered as .23.
cohort_dropout_assess	Specify how dropout rate was defined in the study.
cohort_exposed_def	exposed group definition: Provide a brief description of the exposed group (i.e., the comparison group) as used in estimation of the relative risk (e.g., never smokers)
cohort_exp_unit_rr	Exposure unit (for continuous risks): Specify the unit of exposure (e.g., grams/day).
cohort_exp_level_rr	Exposure level in the exposed group (for continuous risks): Specify the mean/median level of exposure in the exposed group.
cohort_unexp_def	unexposed group definition: Provide a brief description of the unexposed group (i.e., the comparison group) as used in estimation of the relative risk (e.g., never smokers)

cohort_unexp_unit_rr	Exposure unit (for continuous risks): Specify the unit of exposure (e.g., grams/day) for the unexposed group
cohort_unexp_level_rr	Exposure level in the unexposed group (for continuous risks): Specify the mean/median level of exposure in the unexposed group.
cohort_exp_level_dr	Exposure level in for dose-repose RRs (for continuous risks): If the study reports dose-repose RR, please specify the level of exposure for the reported RR
cc_community	Were the controls selected from the community? 1 = yes, 0=no
cc_cases	Number of cases
cc_control	Number of controls
cc_exposed_def	Exposed group definition: Provide a brief description of the exposed group for which the the relative risk is reported (e.g., current smokers)
cc_exp_unit_rr	Exposure unit (for continuous risks): Specify the unit of exposure (e.g., grams/day).
cc_exp_level_rr	Exposure level in the exposed group (for continuous risks): Specify the mean/median level of exposure in the exposed group.
cc_unexposed_def	Unexposed group definition: Provide a brief description of the unexposed group (i.e., the comparison group) as used in estimation of the relative risk (e.g., never smokers)
cc_unexp_unit_rr	
cc_unexp_level_rr	Exposure level in the unexposed group (for continuous risks): Specify the mean/median level of exposure in the unexposed group.
cc_exp_level_dr	Exposure level in for dose-repose RRs (for continuous risks): If the study reports dose-repose RR, please specify the level of exposure for the reported RR
int_intervention_description	Intervention definition: Provide a brief description of the intervention as reported in the study.
int_control_description	control definition: Provide a brief description of the control as reported in the study.
int_intervention_multi_rf	Does this intervention simultaneously target more than one risk? (1=yes, 0=no)
int_intervention_multi_rf_specify	Specify the risks that are targted by the interevention
int_intervention_level	Level of intervention: The intervention was implemented …
int_adhere_assess	Specify how adherence was defined in the study.
int_adhere_rate_intervention	adherence rate in the intervention group; Enter on a ""per 1"" basis. For example: 23% is entered as .23.
int_adhere_rate_control	adherence rate in the control group; Enter on a ""per 1"" basis. For example: 23% is entered as .23.
int_dropout_rate_intervention	Dropout rate? in the intervention group: Specify the dropout rate (%) at the end of the study. Enter on a ""per 1"" basis. For example: 23% is entered as .23.
int_dropout_rate_control	Dropout rate? in the control group: Specify the dropout rate (%) at the end of the study. Enter on a ""per 1"" basis. For example: 23% is entered as .23.
int_dropout_assess	Specify how dropout rate was defined in the study.
int_blinding	For interventional studies. Blinding: The trial was … (select 1)
int_exp_unit	For trials, specify the unit of exposure (e.g., mmol/l)
int_baseline_exp_int	For trials, specify the exposure level in the intervention group at baseline
int_baseline_exp_comp	For trials, specify the exposure level in the comparison group at baseline
int_fup_exp_int	For trials, specify the exposure level in the intervention group at the end of the follow-up time
int_fup_exp_comp	For trials, specify the exposure level in the comparison group at the end of follow up time

int_fup_exp_int_difference	For trials, please specify the difference of exposure level between baseline and follow up time for the intervention group
int_fup_exp_comp_difference	For trials, please specify the difference of exposure level between baseline and follow up time for the comparison group
int_person_years_int	Please specify the number of person years of follow up for the intervention group
int_person_years_comp	Please specify the number of person years of follow up in the comparison group
int_number_events_int	For trials, specify the number of cases in the intervention group at the end of follow up
int_number_events_comp	For trials, specify the number of cases in the control group at the end of follow up
int_sample_size_int_group_baseline	For trials, specify the sample size in the intervention group at baseline
int_sample_size_comparison_group_baseline	For trials, specify the sample size in the comparison group at baseline
int_sample_size_int_group_follow_up	For trials, specify the sample size in the intervention group at the end of the follow-up time
int_sample_size_comparison_group_follow_up	For trials, specify the sample size in the comparison group at the end of follow up time
note_modeler	for modelers only, audience is modeler, not for correspondence
note_sr	notes related to extraction, including assumptions, data adjustment, problems with source, any other notes that may be relevant, etc.

Section 1.5: PRISMA diagrams

See next page.

PRISMA 2020 flow diagram for a new systematic review of the Smoking and Cardiovascular disease risk-outcome pair for age-specific relative risks

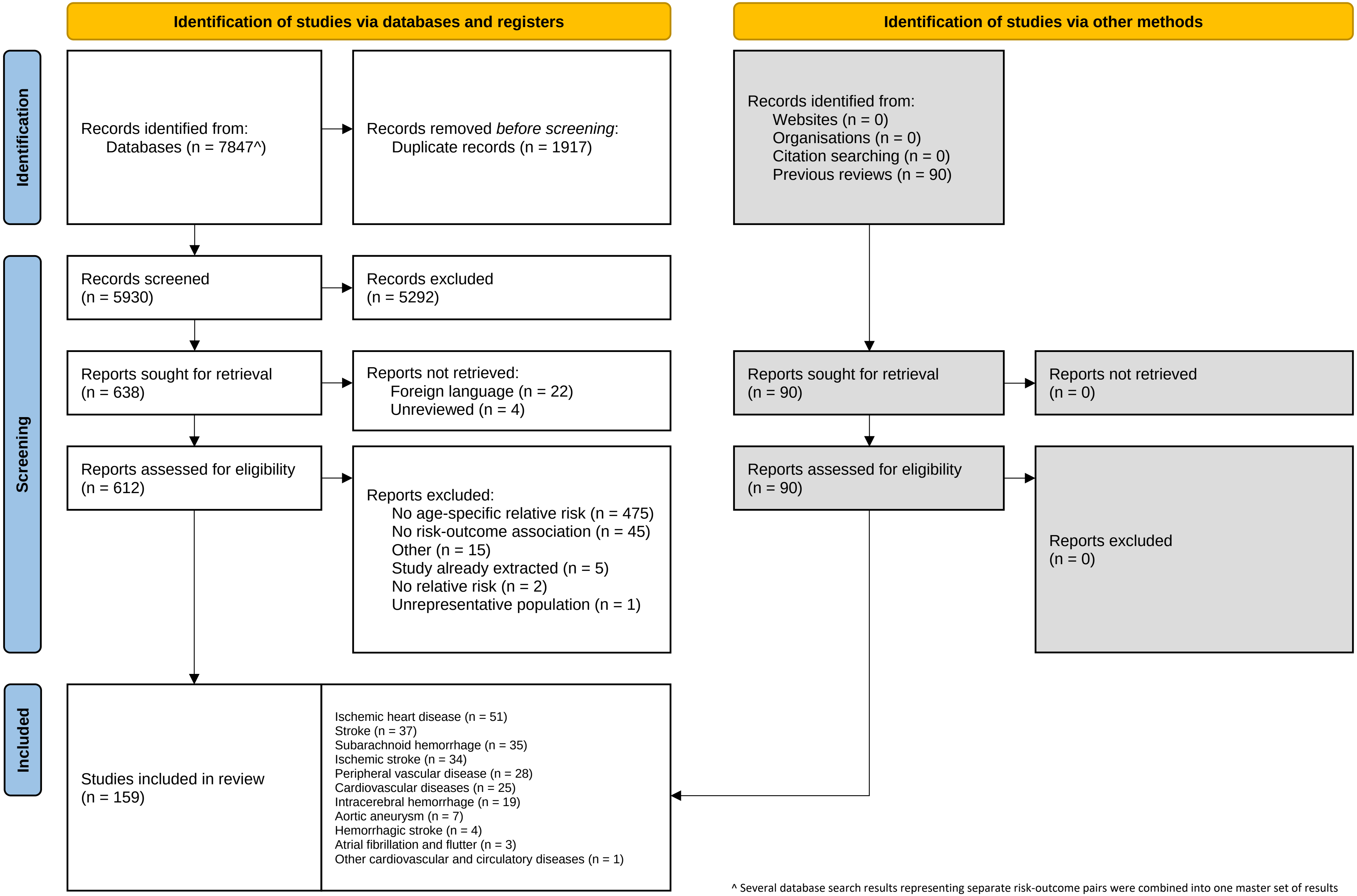

^ Several database search results representing separate risk-outcome pairs were combined into one master set of results

PRISMA 2020 flow diagram for an updated systematic review of the Smoking and Atrial fibrillation and flutter risk-outcome pair

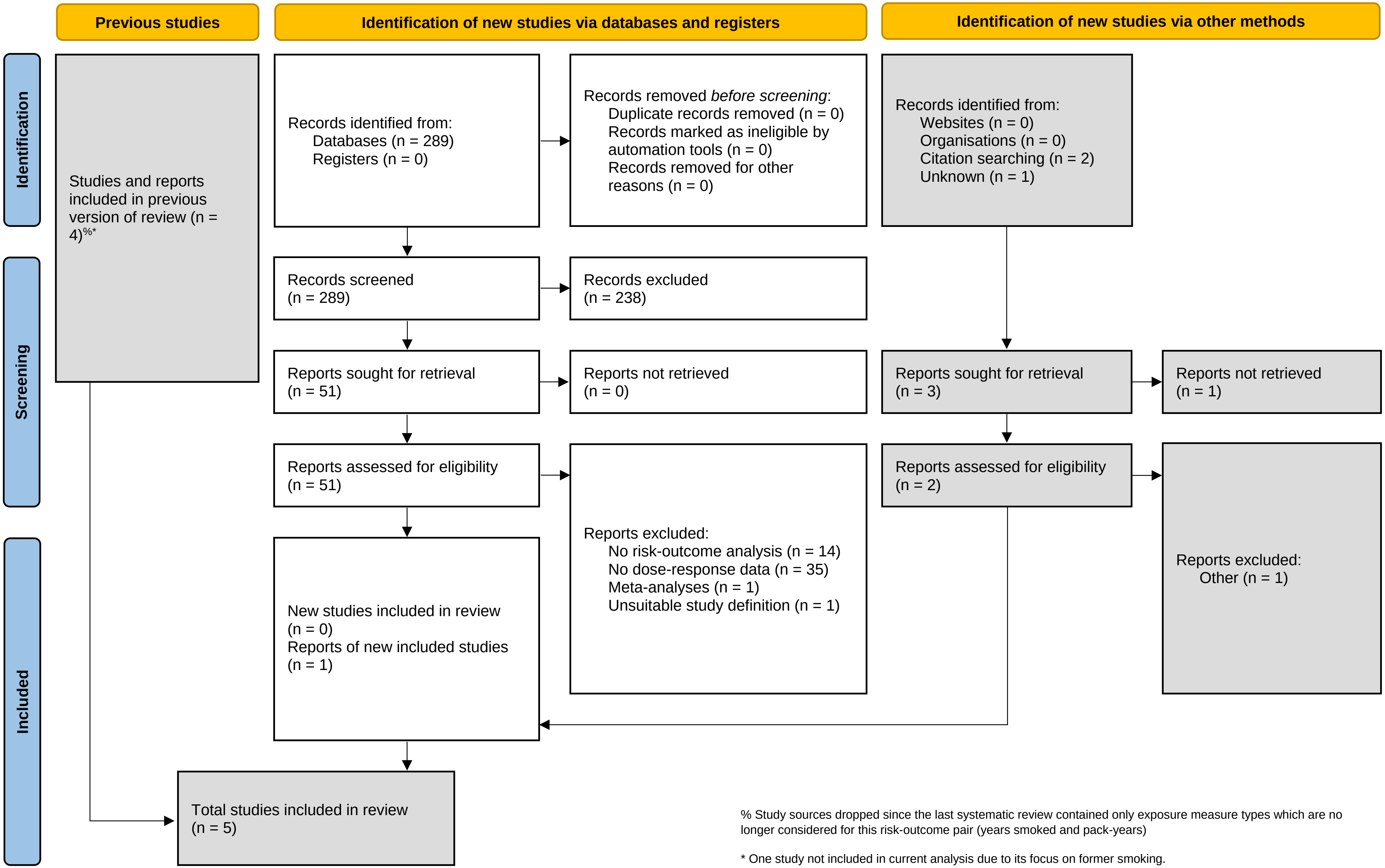

% Study sources dropped since the last systematic review contained only exposure measure types which are no longer considered for this risk-outcome pair (years smoked and pack-years)

* One study not included in current analysis due to its focus on former smoking.

PRISMA 2020 flow diagram for an updated systematic review of the Smoking and Aortic aneurysm risk-outcome pair

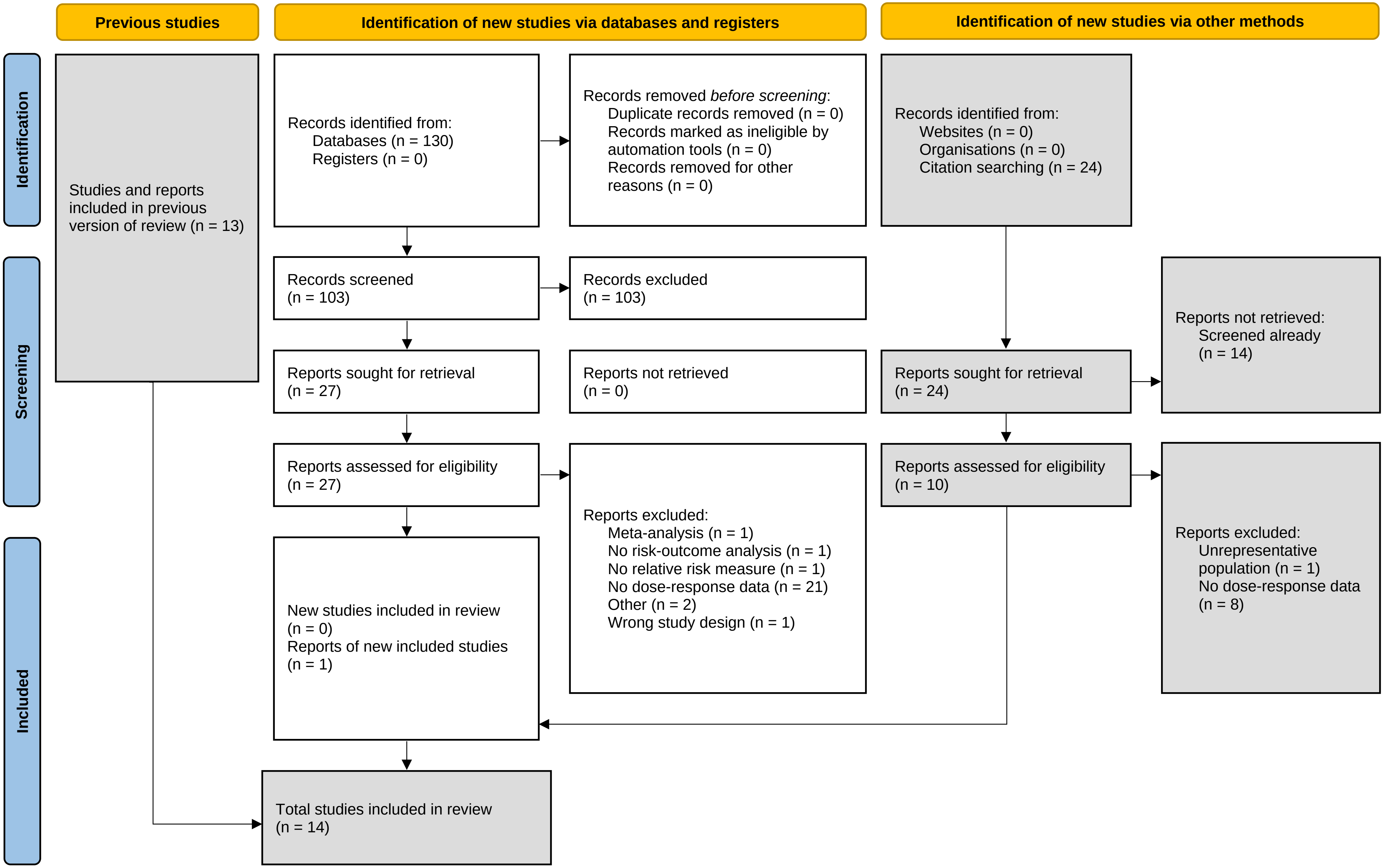

PRISMA 2020 flow diagram for an updated systematic review of the Smoking and Ischemic heart disease risk-outcome pair

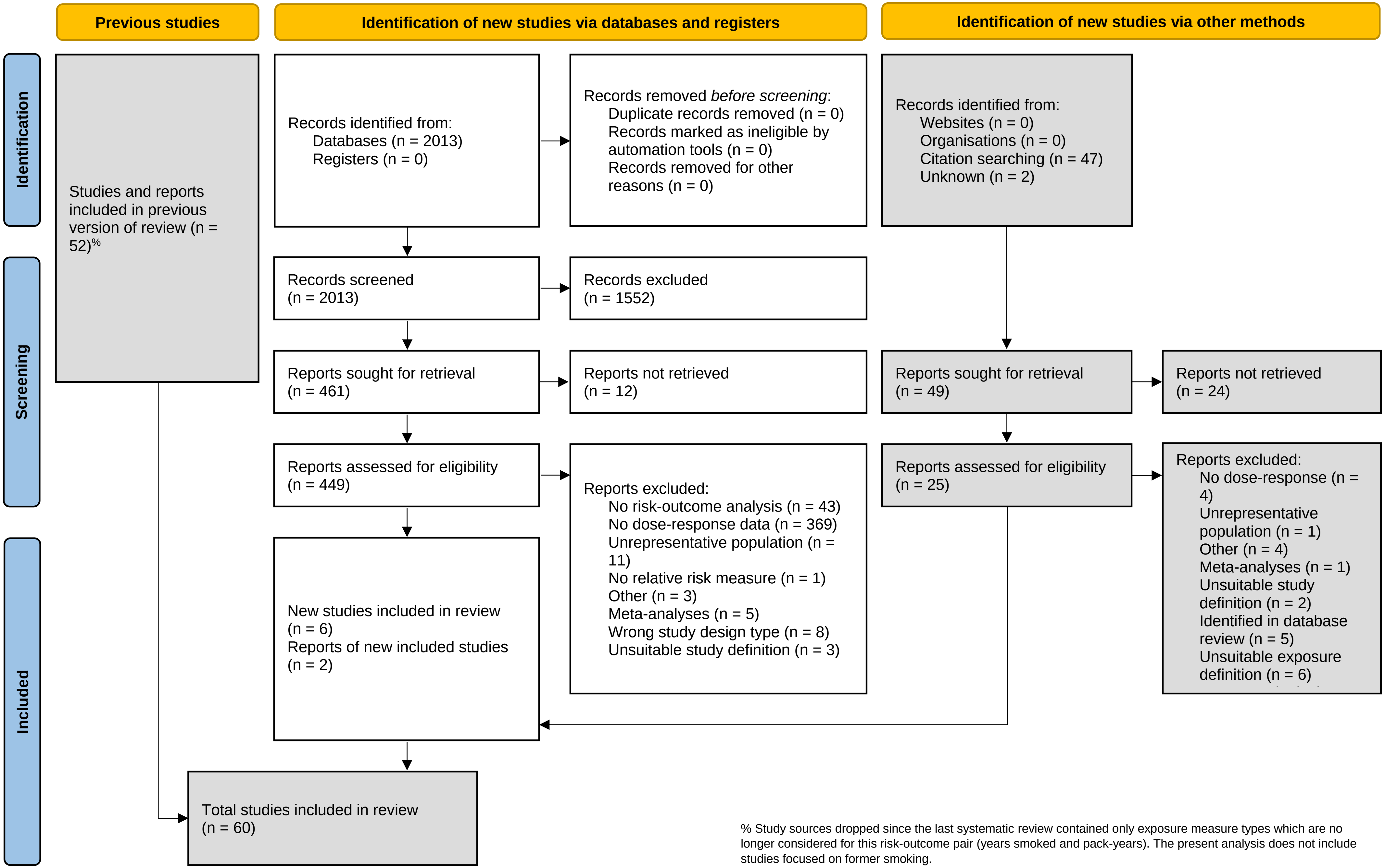

% Study sources dropped since the last systematic review contained only exposure measure types which are no longer considered for this risk-outcome pair (years smoked and pack-years). The present analysis does not include studies focused on former smoking.

PRISMA 2020 flow diagram for an updated systematic review of the Smoking and Lower extremity peripheral arterial disease risk-outcome pair

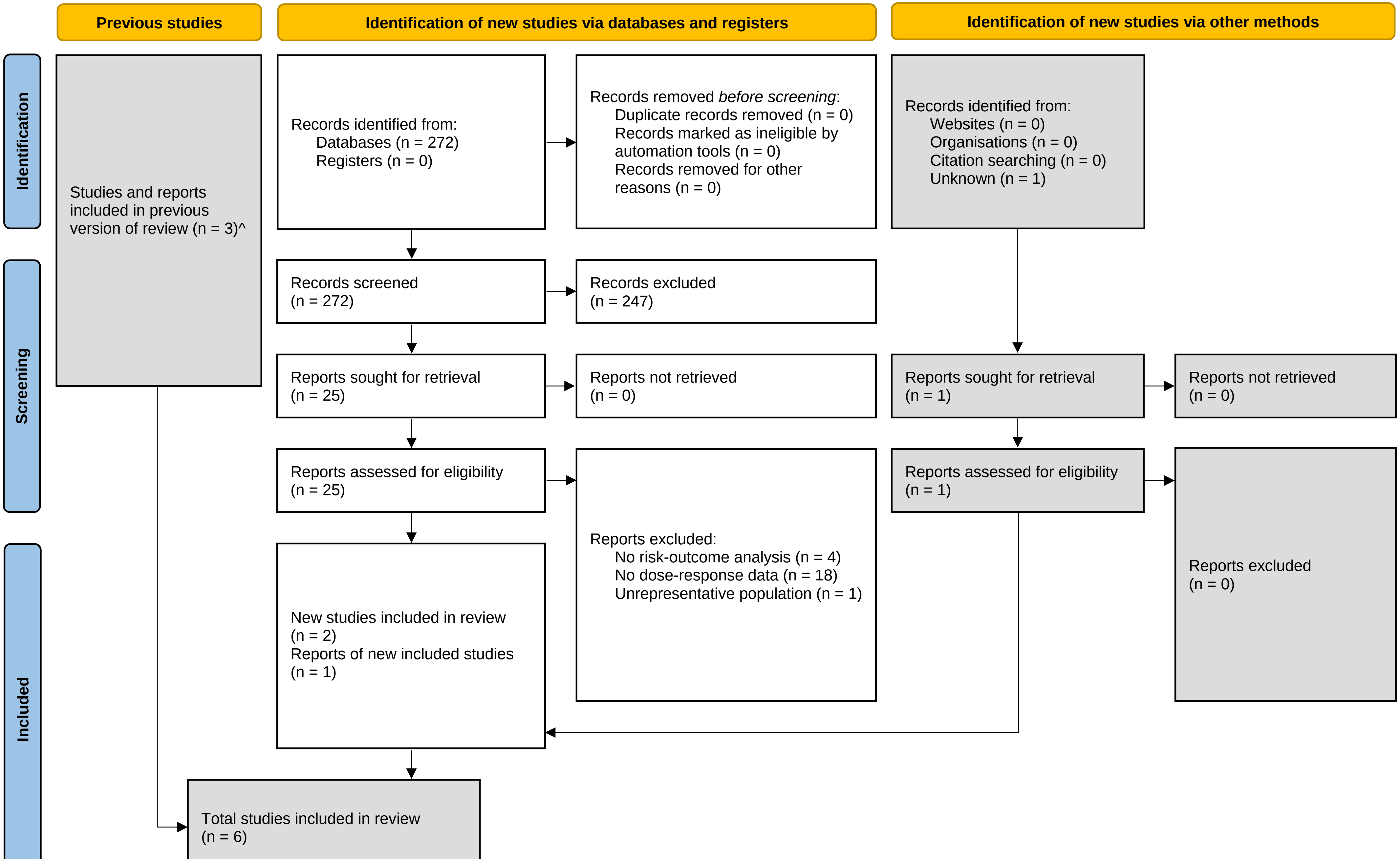

^ Studies focusing on former smoking were not included in the present analysis.

PRISMA 2020 flow diagram for an updated systematic review of the Smoking and Stroke risk-outcome pair

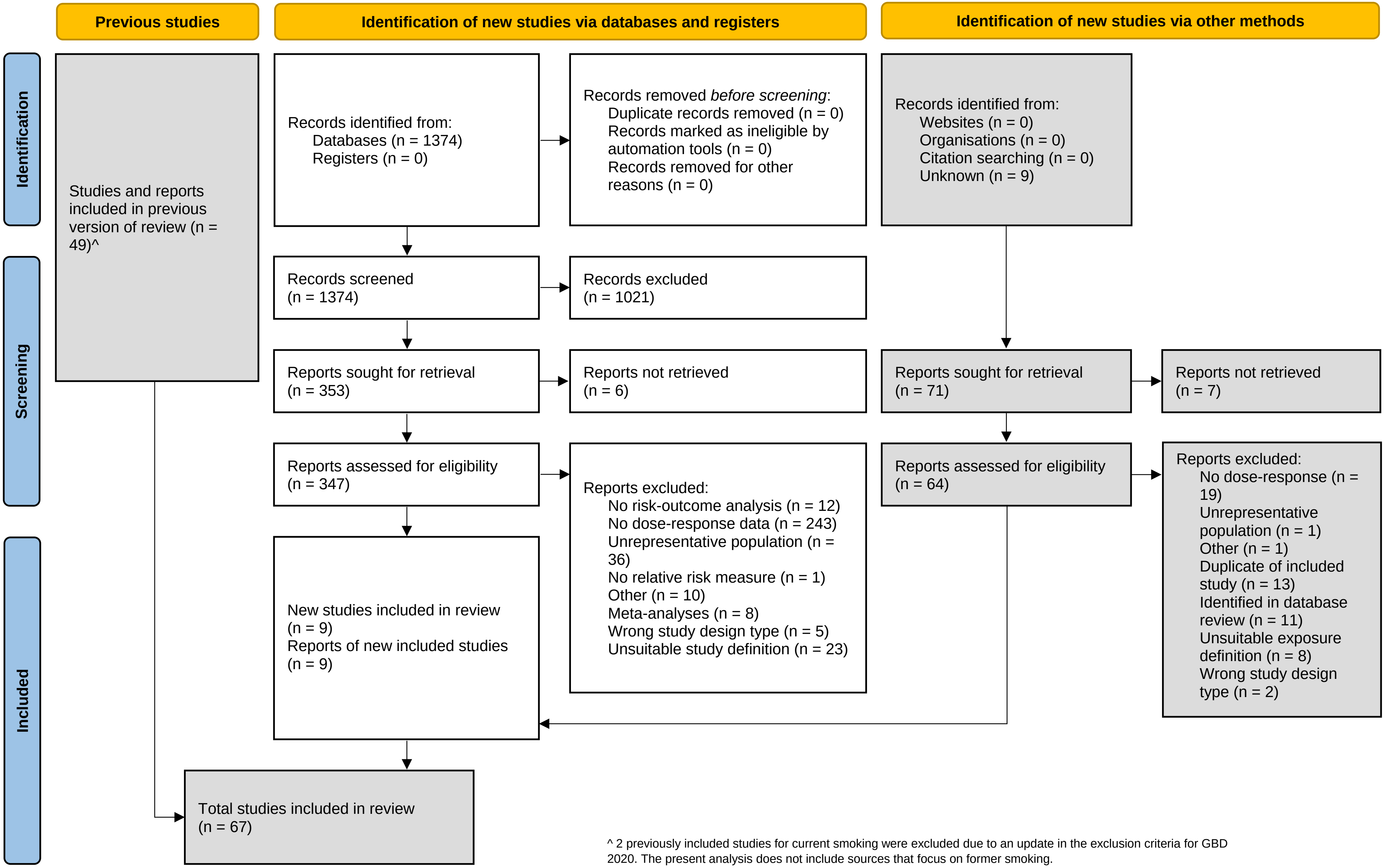

^ 2 previously included studies for current smoking were excluded due to an update in the exclusion criteria for GBD 2020. The present analysis does not include sources that focus on former smoking.

PRISMA 2020 flow diagram for an updated systematic review of the Smoking and Gallbladder and biliary diseases risk-outcome pair

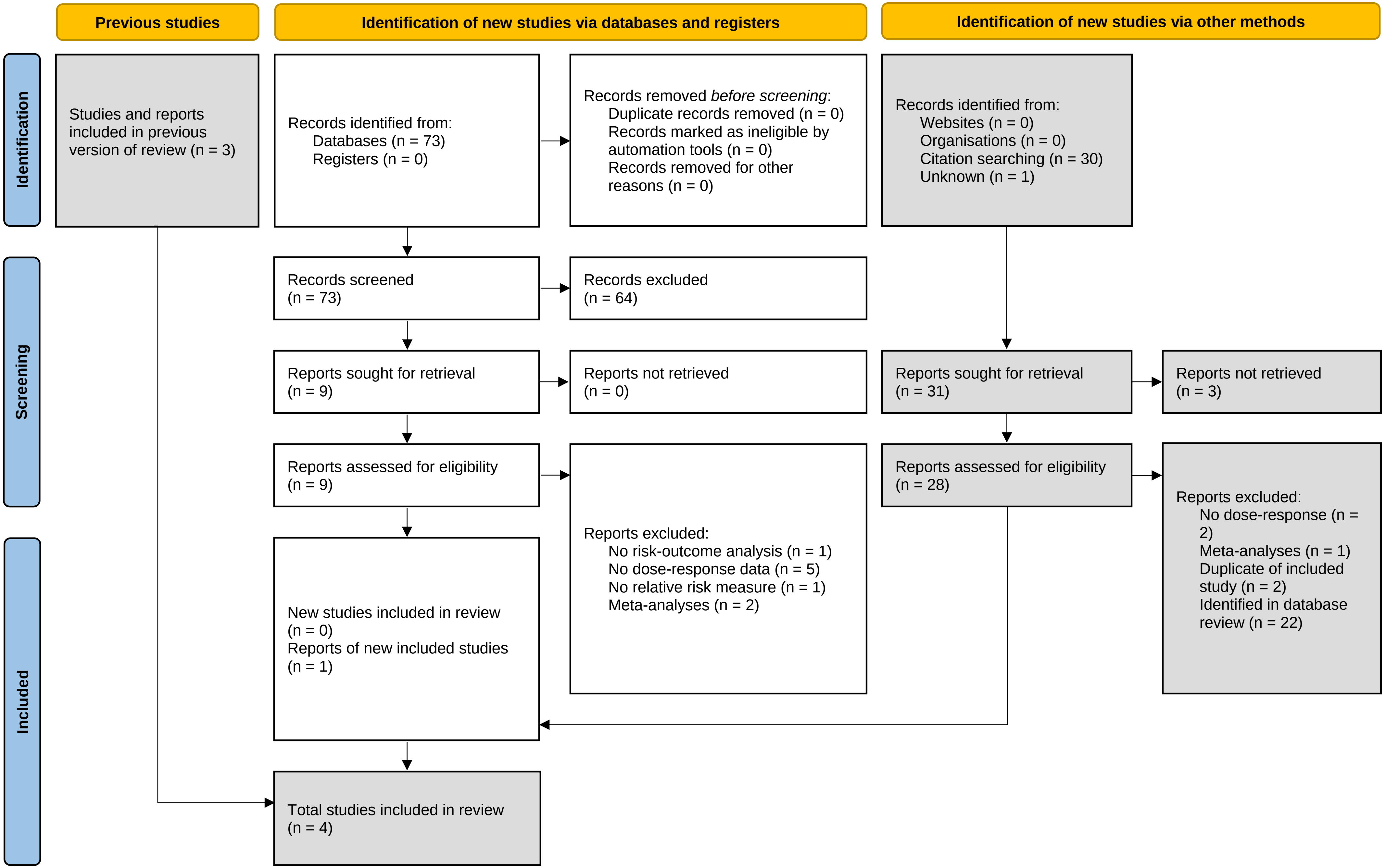

PRISMA 2020 flow diagram for an updated systematic review of the Smoking and Peptic ulcer disease risk-outcome pair

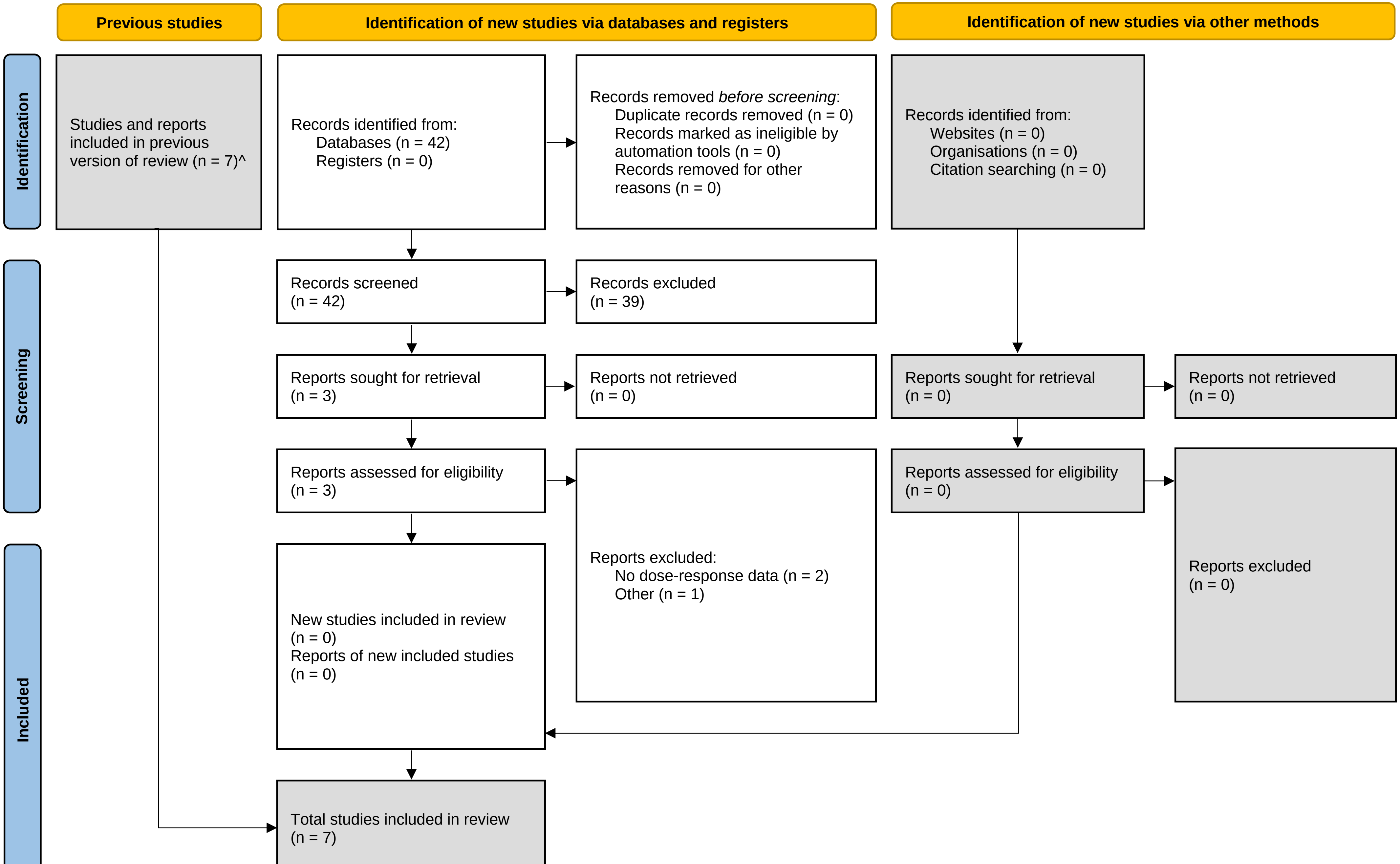

^ 2 previously included studies for current smoking (cig/day) were excluded due to an update in the exclusion criteria for GBD 2020

PRISMA 2020 flow diagram for an updated systematic review of the Smoking and Lower respiratory infections risk-outcome pair

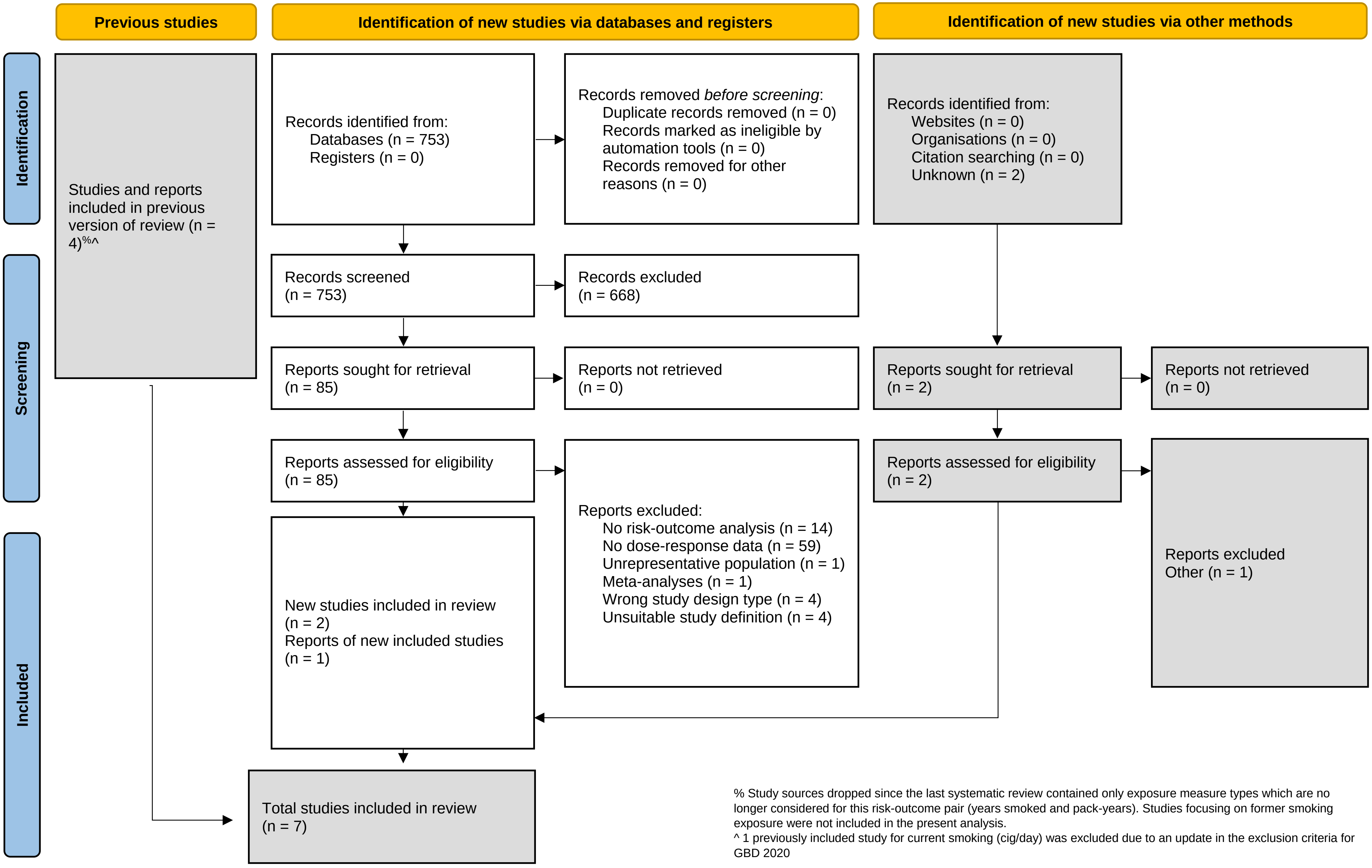

% Study sources dropped since the last systematic review contained only exposure measure types which are no longer considered for this risk-outcome pair (years smoked and pack-years). Studies focusing on former smoking exposure were not included in the present analysis.

^ 1 previously included study for current smoking (cig/day) was excluded due to an update in the exclusion criteria for GBD 2020

PRISMA 2020 flow diagram for an updated systematic review of the Smoking and Low back pain risk-outcome pair

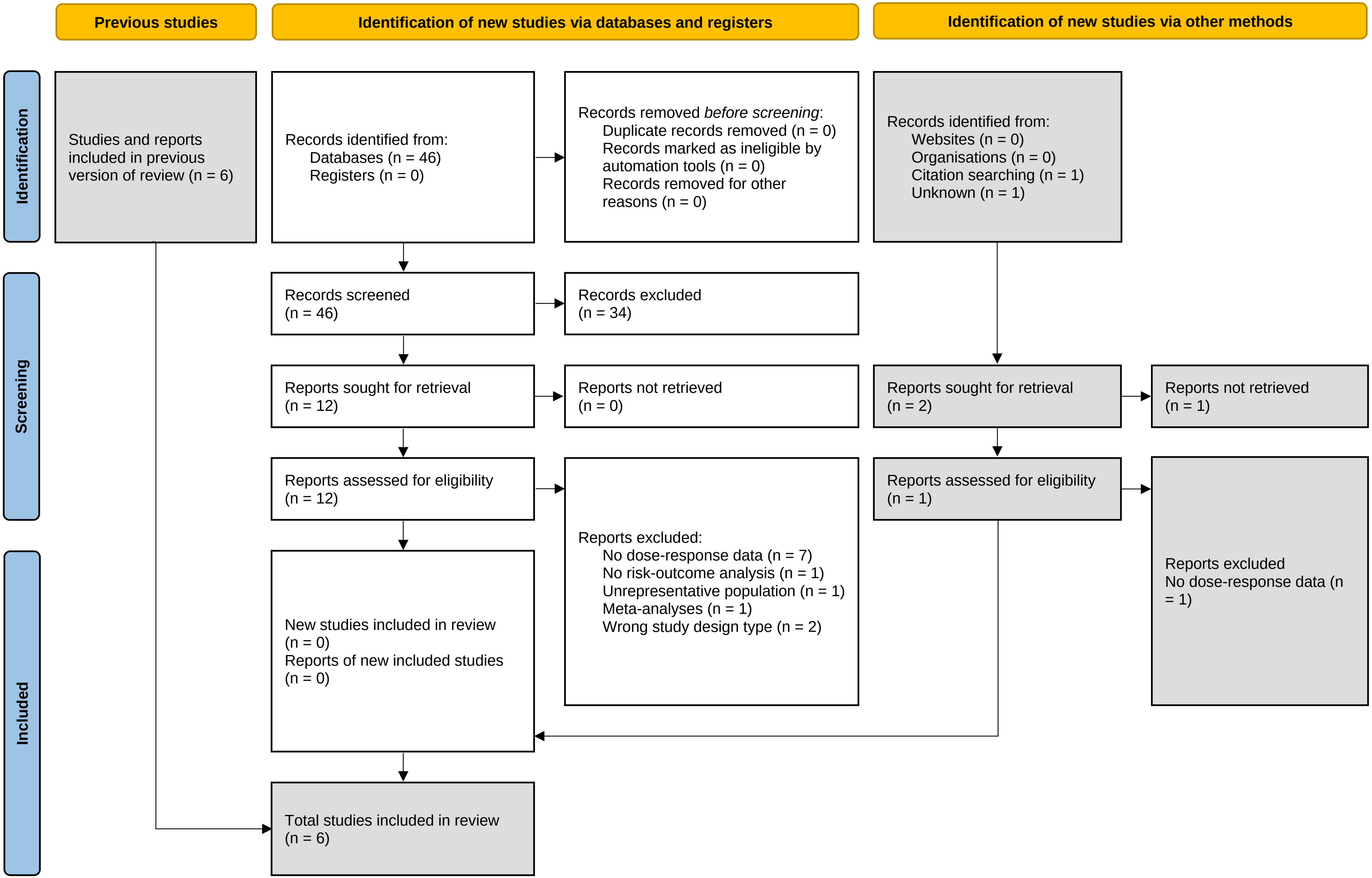

PRISMA 2020 flow diagram for an updated systematic review of the Smoking and Rheumatoid arthritis risk-outcome pair

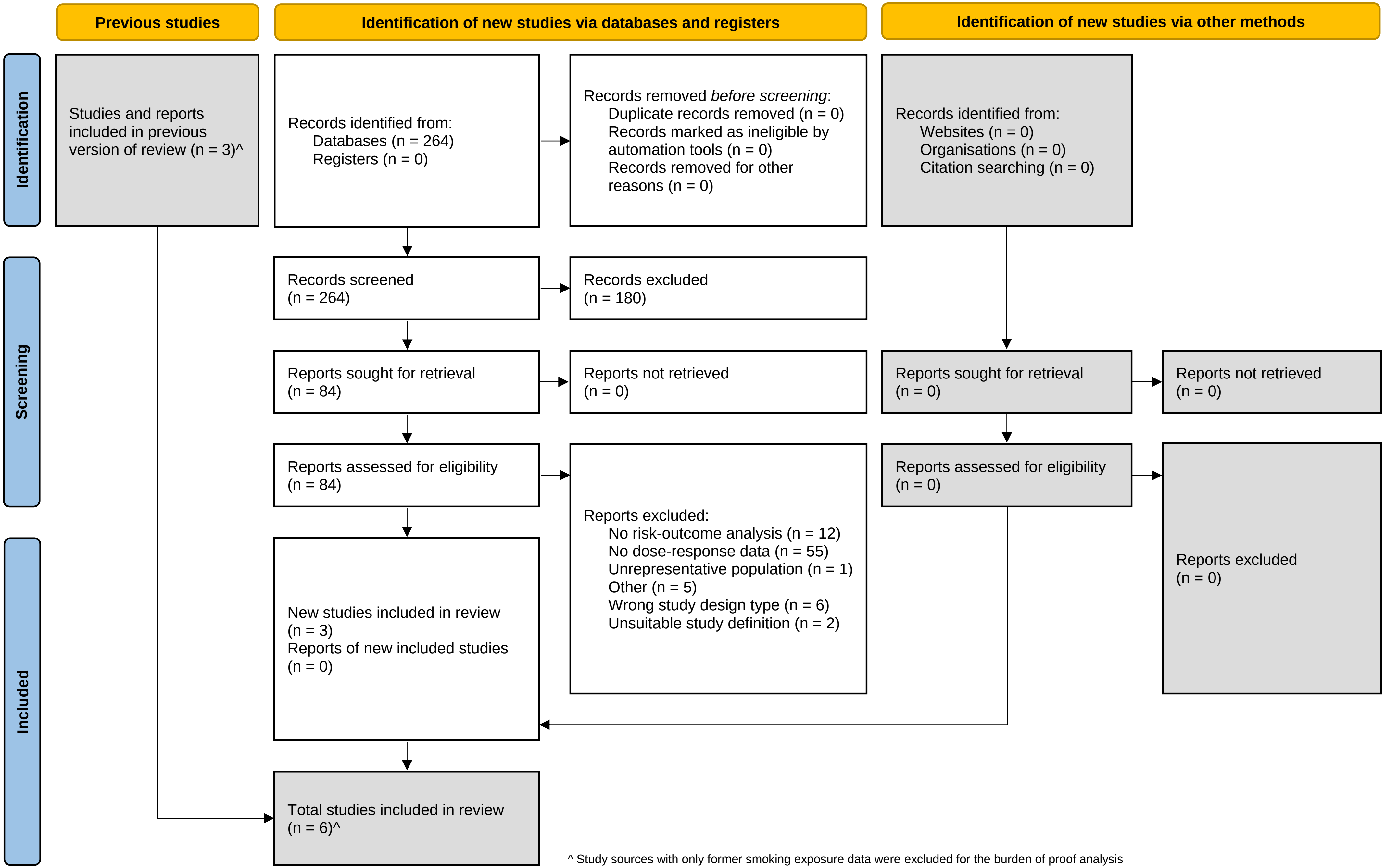

^ Study sources with only former smoking exposure data were excluded for the burden of proof analysis

PRISMA 2020 flow diagram for an updated systematic review of the Smoking and Bladder cancer risk-outcome pair

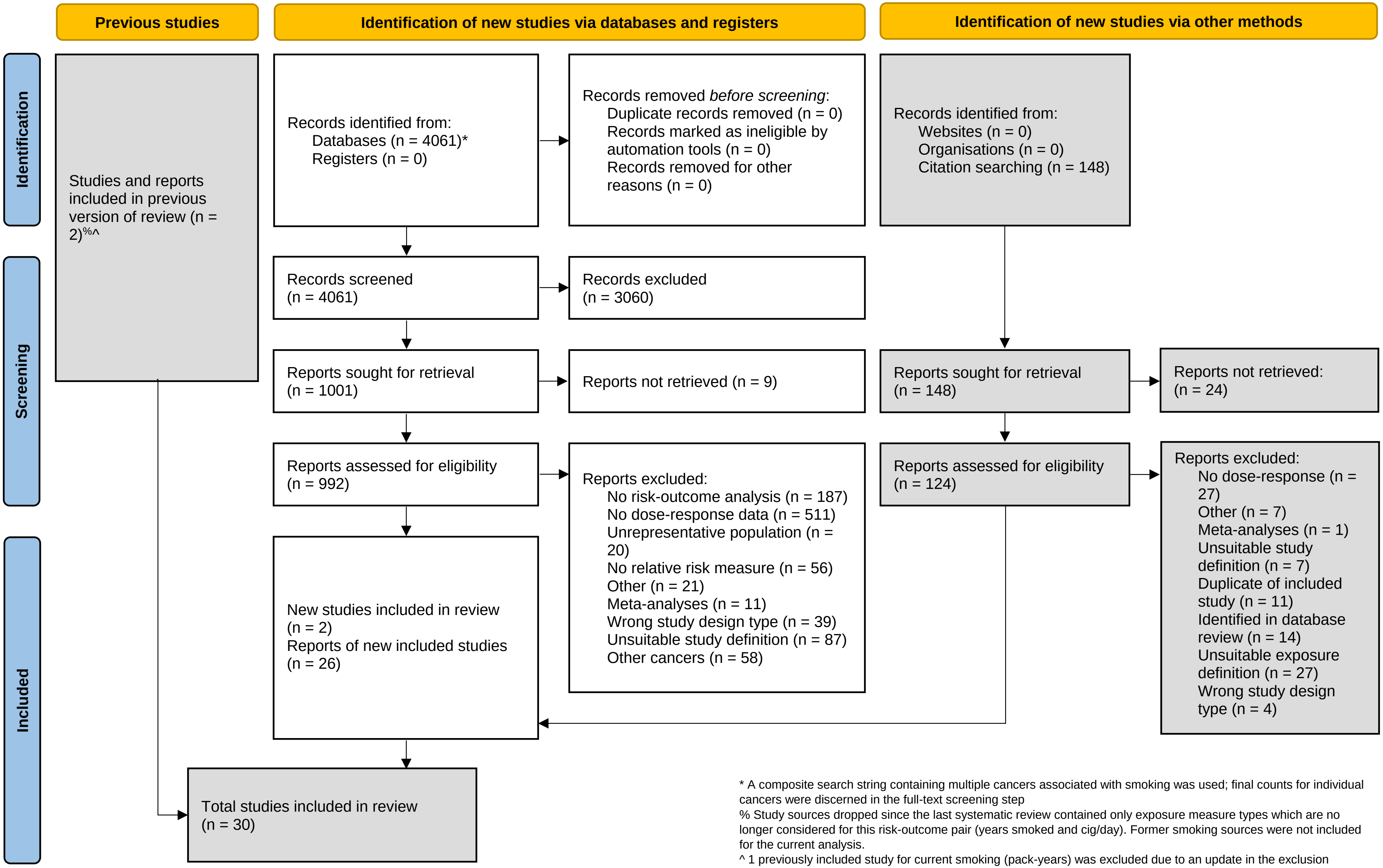

* A composite search string containing multiple cancers associated with smoking was used; final counts for individual cancers were discerned in the full-text screening step

% Study sources dropped since the last systematic review contained only exposure measure types which are no longer considered for this risk-outcome pair (years smoked and cig/day). Former smoking sources were not included for the current analysis.

^ 1 previously included study for current smoking (pack-years) was excluded due to an update in the exclusion criteria for GBD 2020

PRISMA 2020 flow diagram for an updated systematic review of the Smoking and Cervical cancer risk-outcome pair

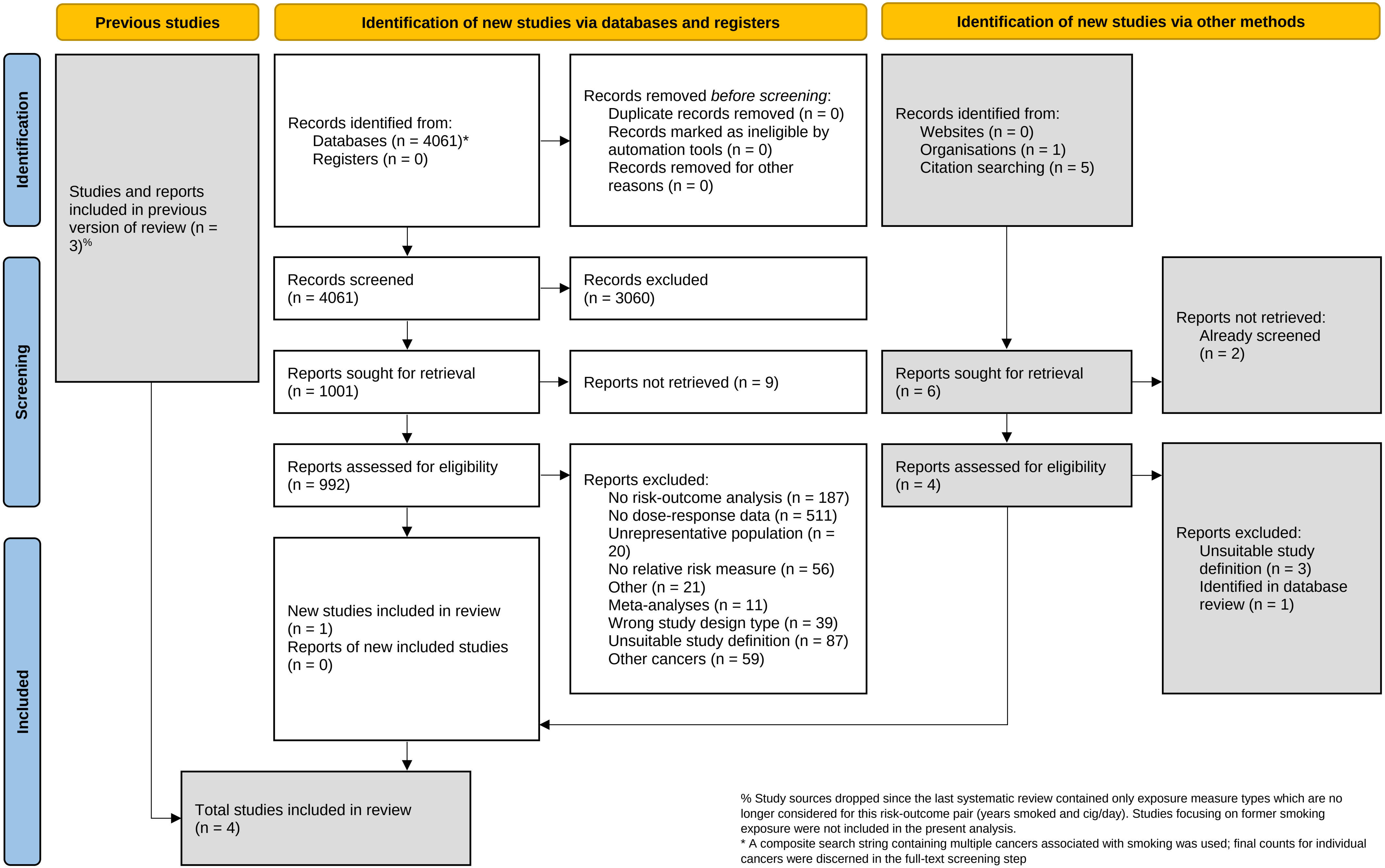

% Study sources dropped since the last systematic review contained only exposure measure types which are no longer considered for this risk-outcome pair (years smoked and cig/day). Studies focusing on former smoking exposure were not included in the present analysis.

* A composite search string containing multiple cancers associated with smoking was used; final counts for individual cancers were discerned in the full-text screening step

PRISMA 2020 flow diagram for an updated systematic review of the Smoking and Colon and rectum cancer risk-outcome pair

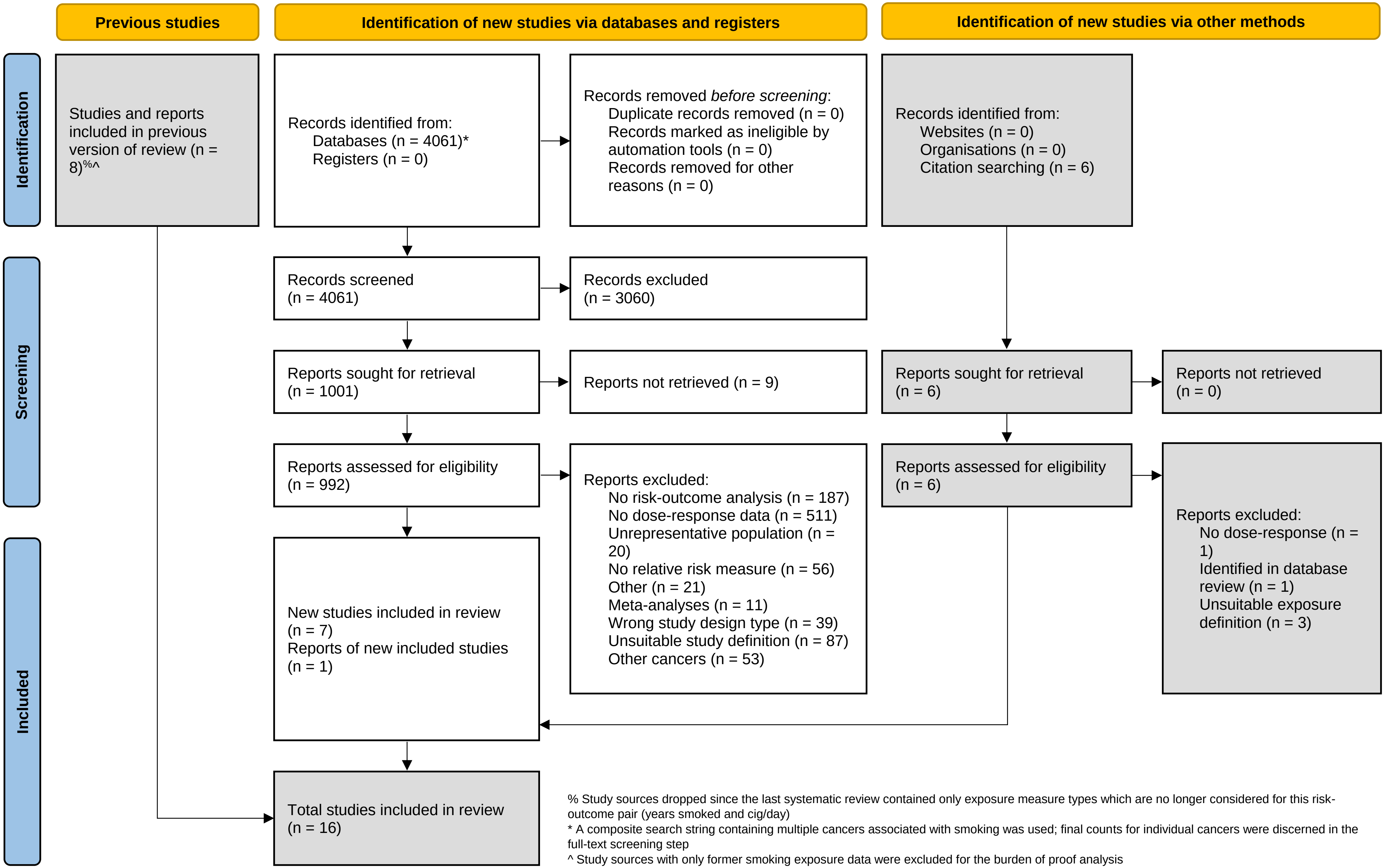

% Study sources dropped since the last systematic review contained only exposure measure types which are no longer considered for this risk-outcome pair (years smoked and cig/day)

* A composite search string containing multiple cancers associated with smoking was used; final counts for individual cancers were discerned in the full-text screening step

^ Study sources with only former smoking exposure data were excluded for the burden of proof analysis

PRISMA 2020 flow diagram for an updated systematic review of the Smoking and Esophageal cancer risk-outcome pair

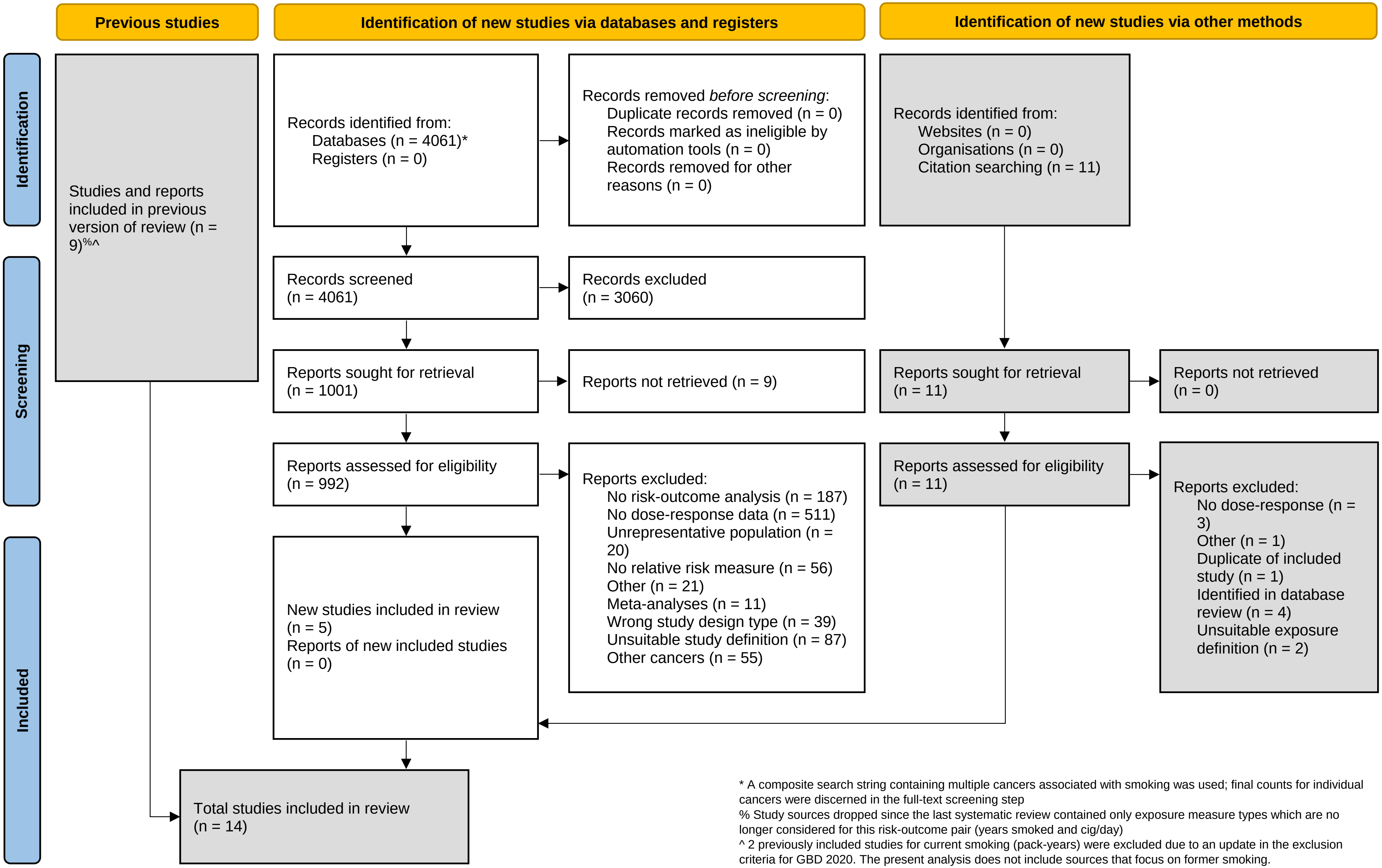

* A composite search string containing multiple cancers associated with smoking was used; final counts for individual cancers were discerned in the full-text screening step

% Study sources dropped since the last systematic review contained only exposure measure types which are no longer considered for this risk-outcome pair (years smoked and cig/day)

^ 2 previously included studies for current smoking (pack-years) were excluded due to an update in the exclusion criteria for GBD 2020. The present analysis does not include sources that focus on former smoking.

PRISMA 2020 flow diagram for an updated systematic review of the Smoking and Kidney cancer risk-outcome pair

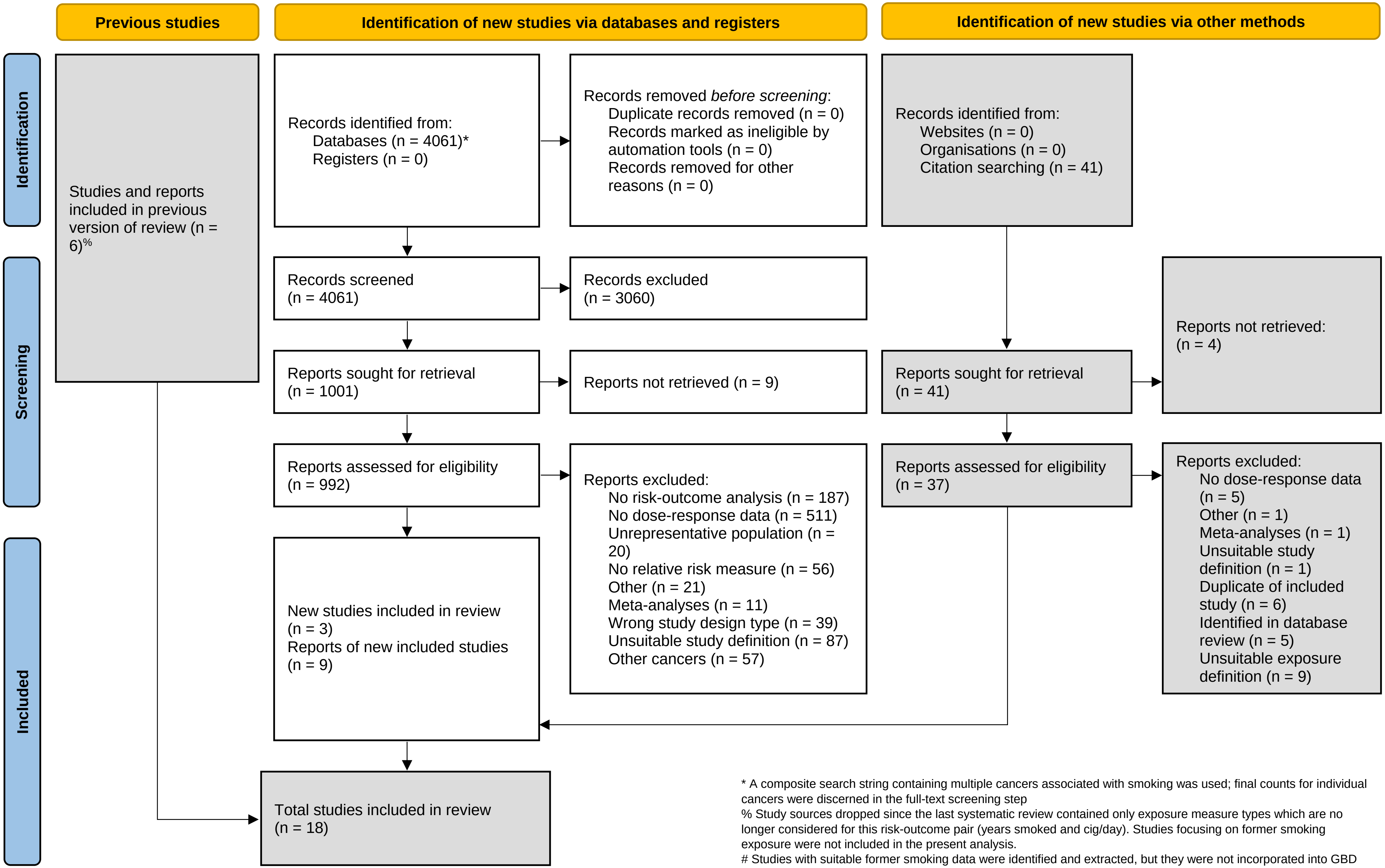

* A composite search string containing multiple cancers associated with smoking was used; final counts for individual cancers were discerned in the full-text screening step

% Study sources dropped since the last systematic review contained only exposure measure types which are no longer considered for this risk-outcome pair (years smoked and cig/day). Studies focusing on former smoking exposure were not included in the present analysis.

Studies with suitable former smoking data were identified and extracted, but they were not incorporated into GBD 2020 results

PRISMA 2020 flow diagram for an updated systematic review of the Smoking and Laryngeal cancer risk-outcome pair

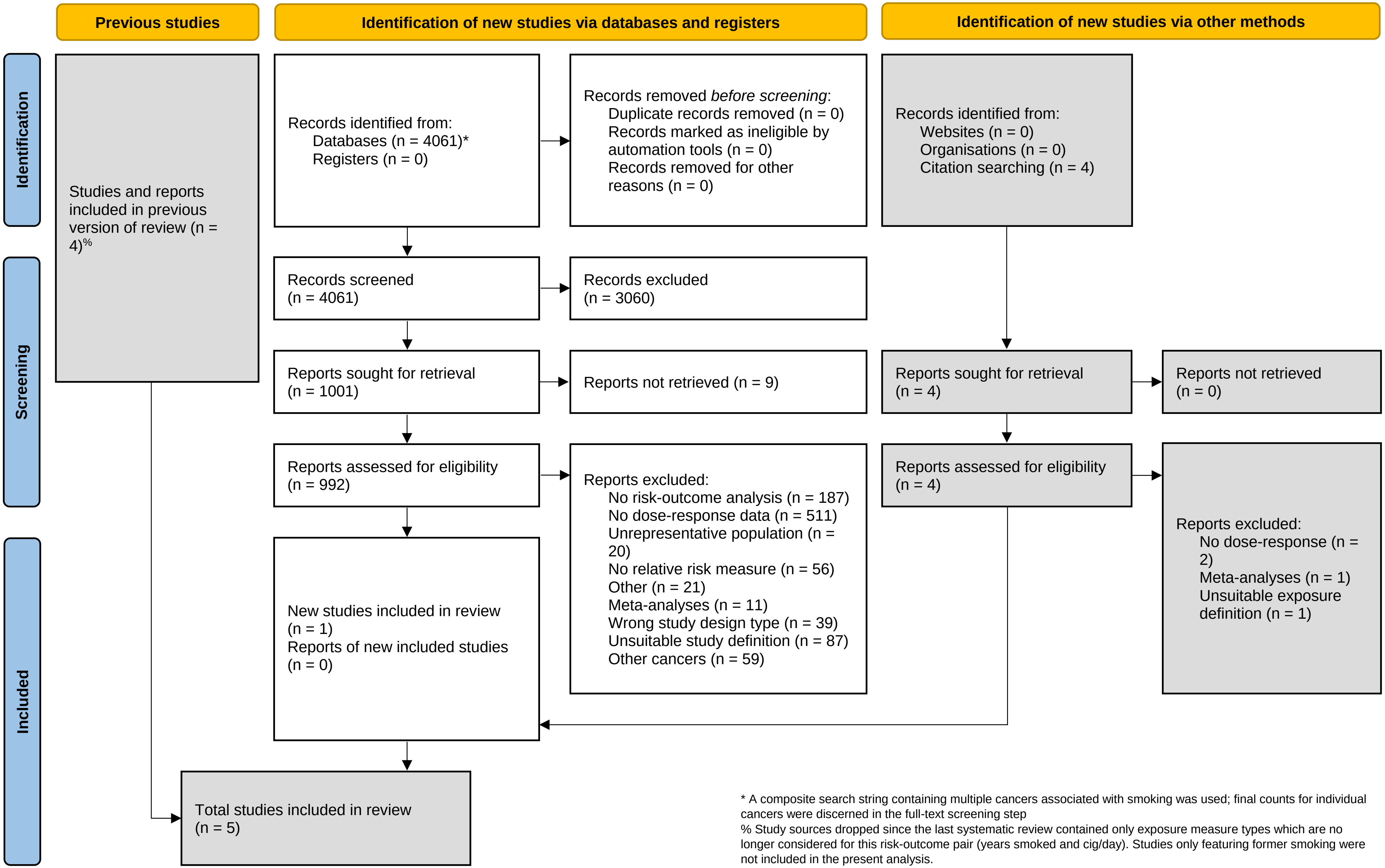

* A composite search string containing multiple cancers associated with smoking was used; final counts for individual cancers were discerned in the full-text screening step

% Study sources dropped since the last systematic review contained only exposure measure types which are no longer considered for this risk-outcome pair (years smoked and cig/day). Studies only featuring former smoking were not included in the present analysis.

PRISMA 2020 flow diagram for an updated systematic review of the Smoking and Leukemia risk-outcome pair

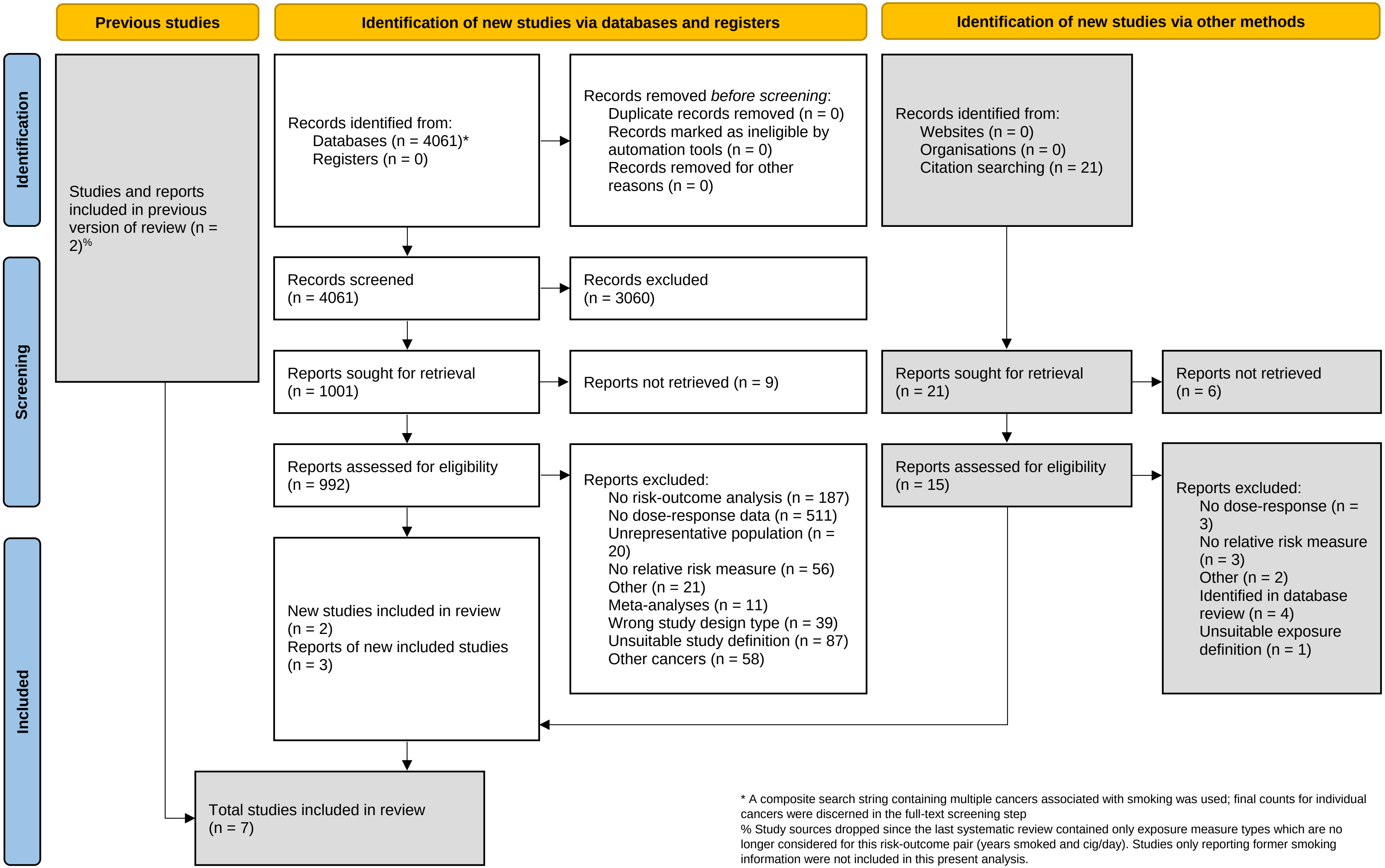

* A composite search string containing multiple cancers associated with smoking was used; final counts for individual cancers were discerned in the full-text screening step

% Study sources dropped since the last systematic review contained only exposure measure types which are no longer considered for this risk-outcome pair (years smoked and cig/day). Studies only reporting former smoking information were not included in this present analysis.

PRISMA 2020 flow diagram for an updated systematic review of the Smoking and Liver cancer risk-outcome pair

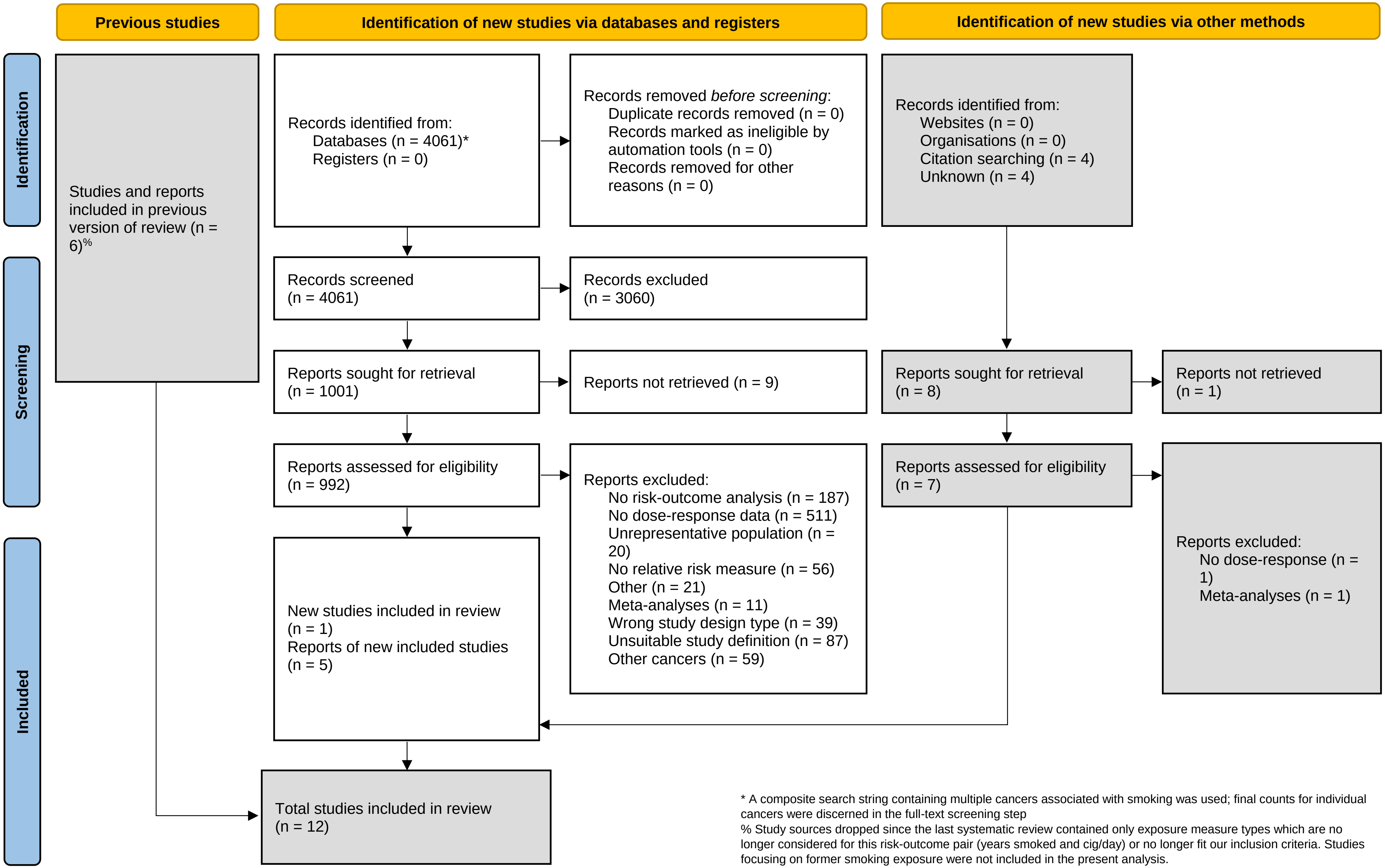

* A composite search string containing multiple cancers associated with smoking was used; final counts for individual cancers were discerned in the full-text screening step

% Study sources dropped since the last systematic review contained only exposure measure types which are no longer considered for this risk-outcome pair (years smoked and cig/day) or no longer fit our inclusion criteria. Studies focusing on former smoking exposure were not included in the present analysis.

PRISMA 2020 flow diagram for an updated systematic review of the Smoking and Lip and oral cavity cancer risk-outcome pair

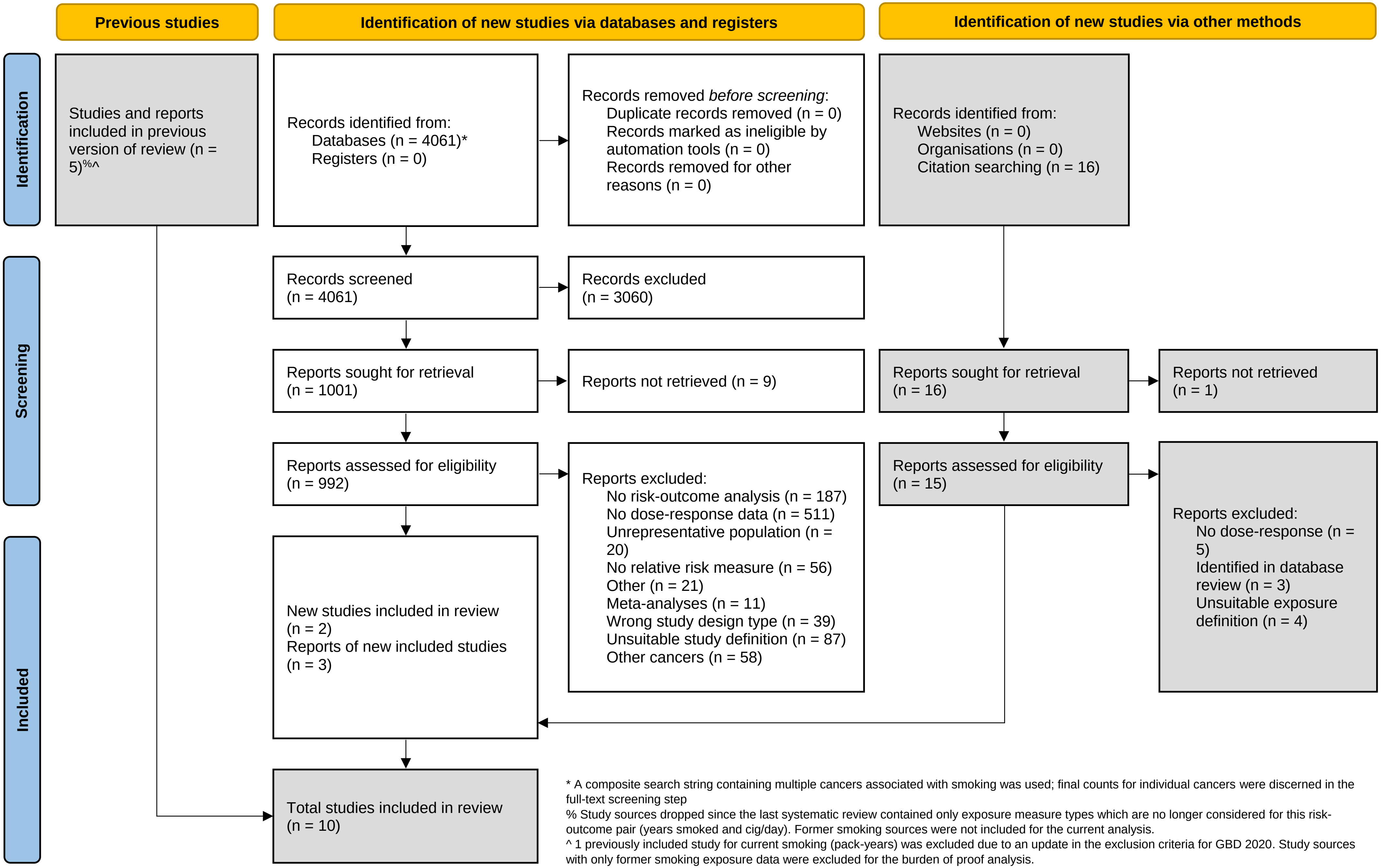

* A composite search string containing multiple cancers associated with smoking was used; final counts for individual cancers were discerned in the full-text screening step

% Study sources dropped since the last systematic review contained only exposure measure types which are no longer considered for this risk-outcome pair (years smoked and cig/day). Former smoking sources were not included for the current analysis.

^ 1 previously included study for current smoking (pack-years) was excluded due to an update in the exclusion criteria for GBD 2020. Study sources with only former smoking exposure data were excluded for the burden of proof analysis.

PRISMA 2020 flow diagram for an updated systematic review of the Smoking and Nasopharynx risk-outcome pair

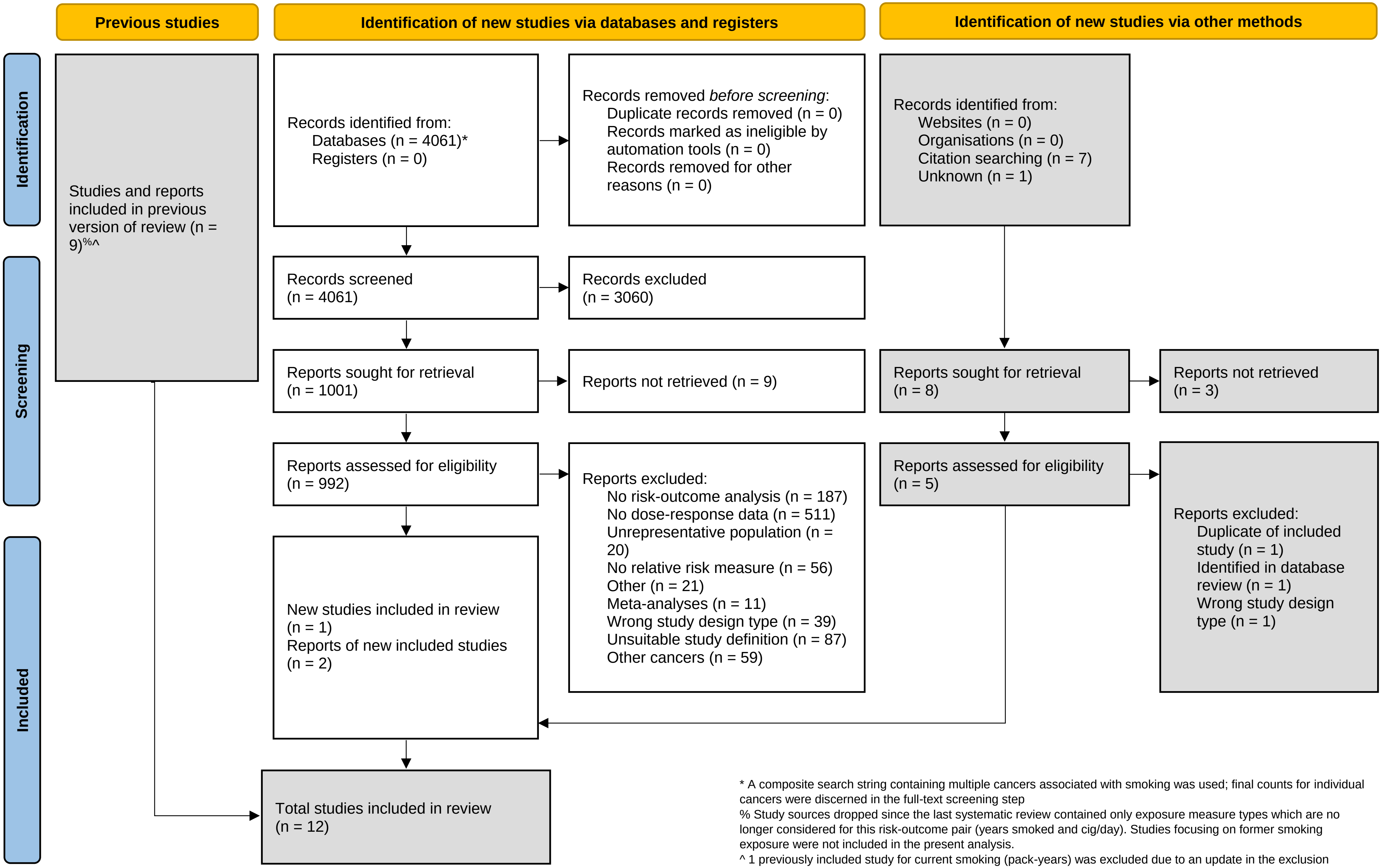

* A composite search string containing multiple cancers associated with smoking was used; final counts for individual cancers were discerned in the full-text screening step

% Study sources dropped since the last systematic review contained only exposure measure types which are no longer considered for this risk-outcome pair (years smoked and cig/day). Studies focusing on former smoking exposure were not included in the present analysis.

^ 1 previously included study for current smoking (pack-years) was excluded due to an update in the exclusion criteria for GBD 2020

PRISMA 2020 flow diagram for an updated systematic review of the Smoking and Other pharynx cancer risk-outcome pair

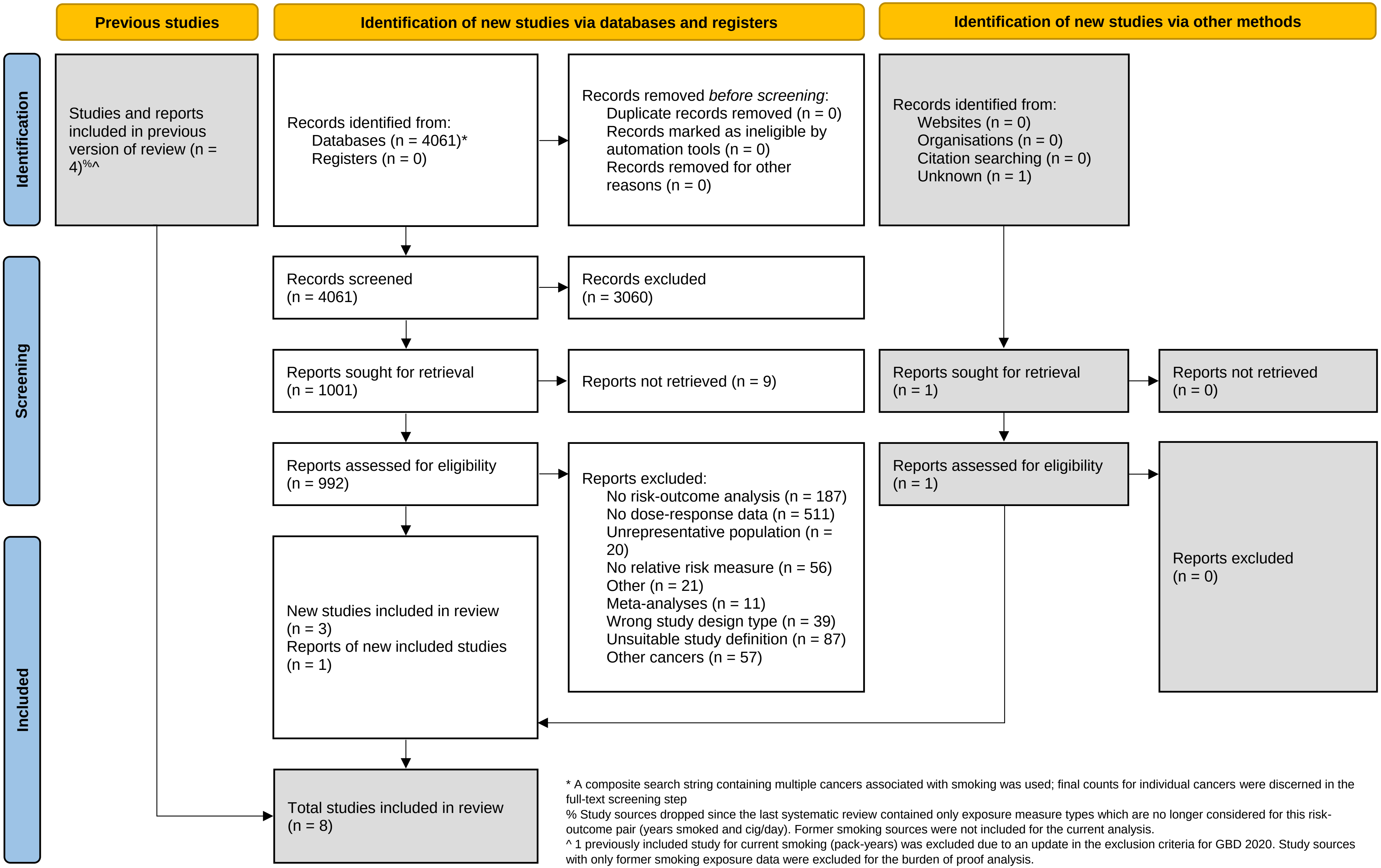

* A composite search string containing multiple cancers associated with smoking was used; final counts for individual cancers were discerned in the full-text screening step

% Study sources dropped since the last systematic review contained only exposure measure types which are no longer considered for this risk-outcome pair (years smoked and cig/day). Former smoking sources were not included for the current analysis.

^ 1 previously included study for current smoking (pack-years) was excluded due to an update in the exclusion criteria for GBD 2020. Study sources with only former smoking exposure data were excluded for the burden of proof analysis.

PRISMA 2020 flow diagram for an updated systematic review of the Smoking and Pancreatic cancer risk-outcome pair

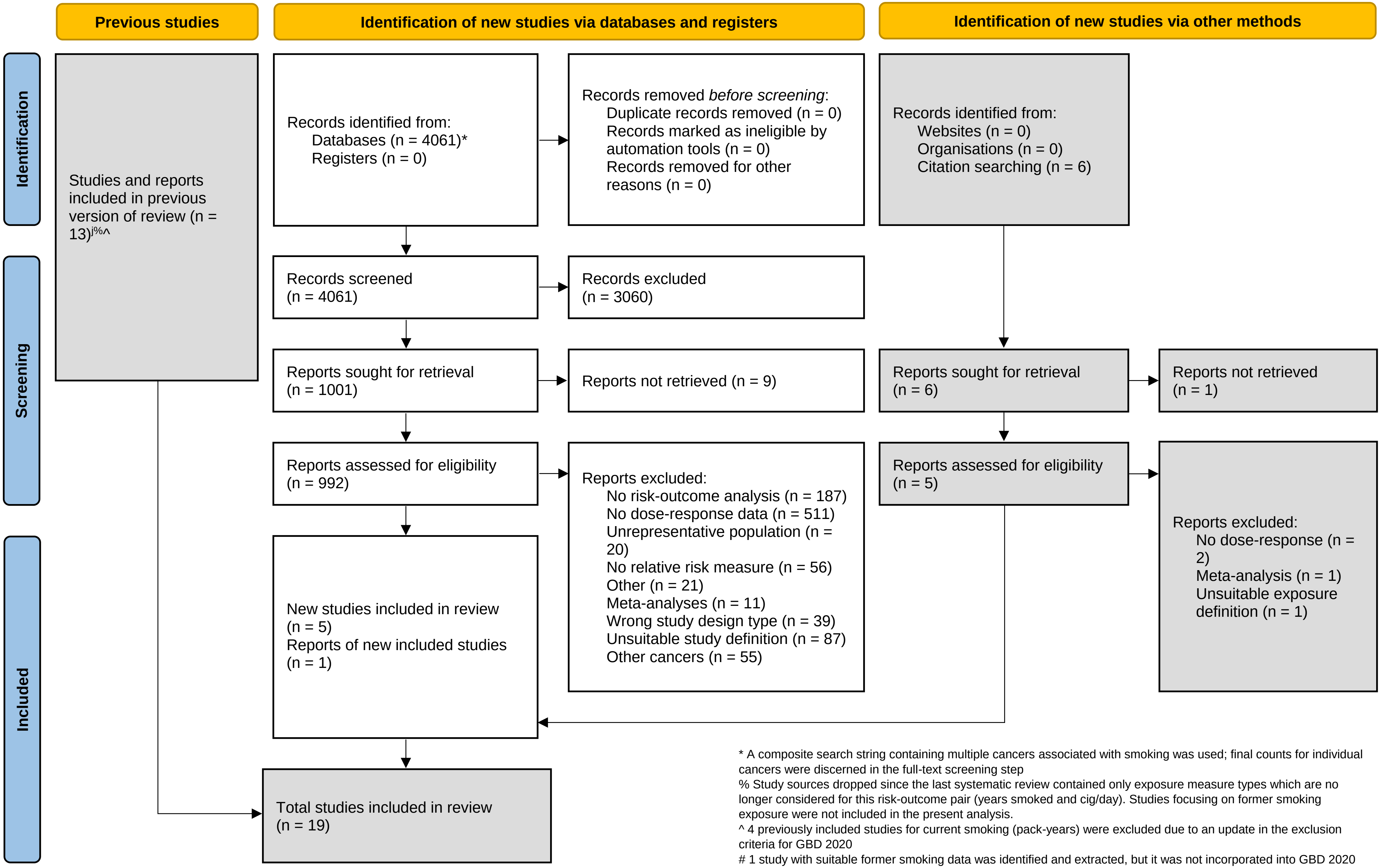

* A composite search string containing multiple cancers associated with smoking was used; final counts for individual cancers were discerned in the full-text screening step

% Study sources dropped since the last systematic review contained only exposure measure types which are no longer considered for this risk-outcome pair (years smoked and cig/day). Studies focusing on former smoking exposure were not included in the present analysis.

^ 4 previously included studies for current smoking (pack-years) were excluded due to an update in the exclusion criteria for GBD 2020

1 study with suitable former smoking data was identified and extracted, but it was not incorporated into GBD 2020 results

PRISMA 2020 flow diagram for an updated systematic review of the Smoking and Stomach cancer risk-outcome pair

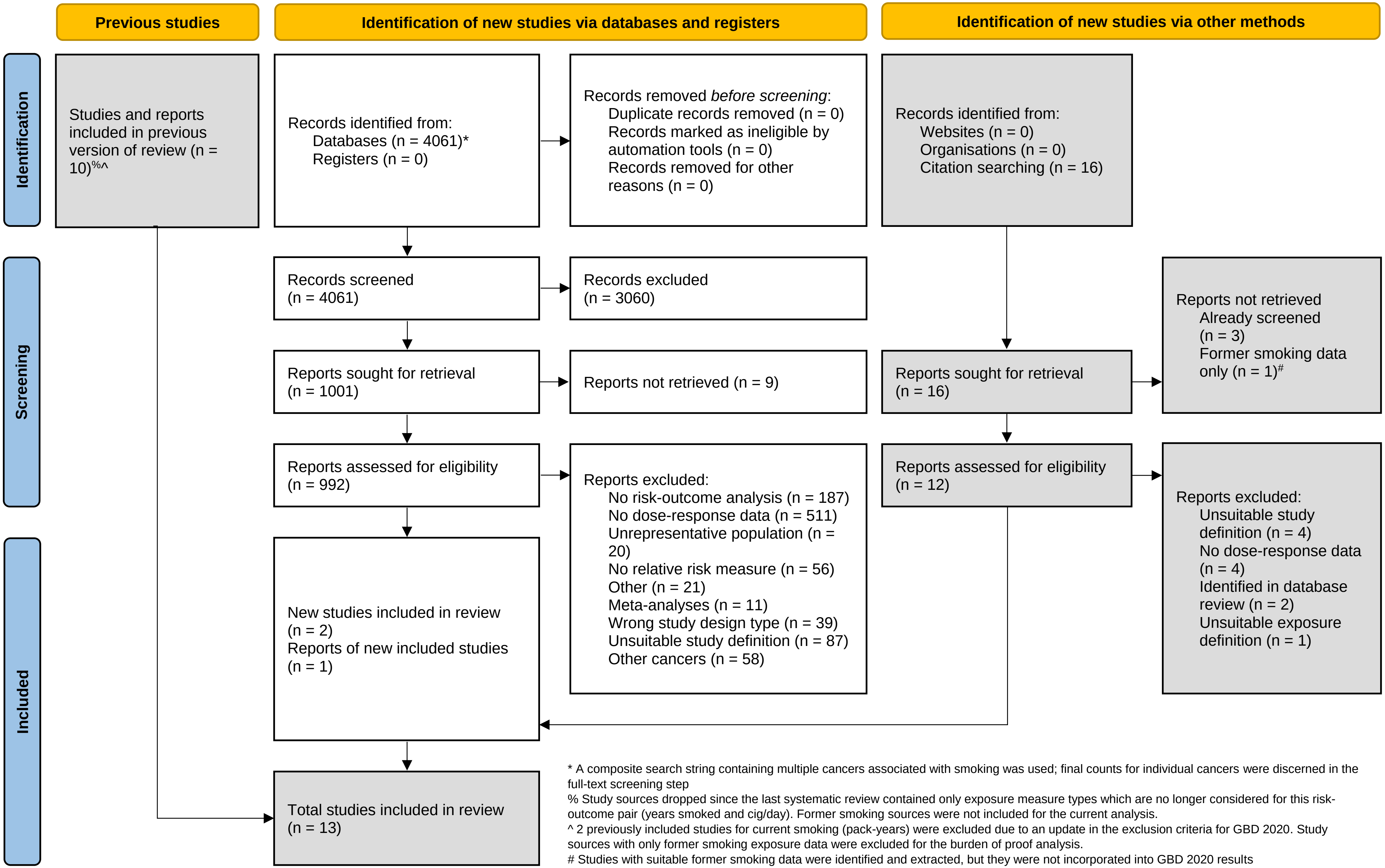

* A composite search string containing multiple cancers associated with smoking was used; final counts for individual cancers were discerned in the full-text screening step

% Study sources dropped since the last systematic review contained only exposure measure types which are no longer considered for this risk-outcome pair (years smoked and cig/day). Former smoking sources were not included for the current analysis.

^ 2 previously included studies for current smoking (pack-years) were excluded due to an update in the exclusion criteria for GBD 2020. Study sources with only former smoking exposure data were excluded for the burden of proof analysis.

Studies with suitable former smoking data were identified and extracted, but they were not incorporated into GBD 2020 results

PRISMA 2020 flow diagram for an updated systematic review of the Smoking and Alzheimer's disease and other dementias risk-outcome pair

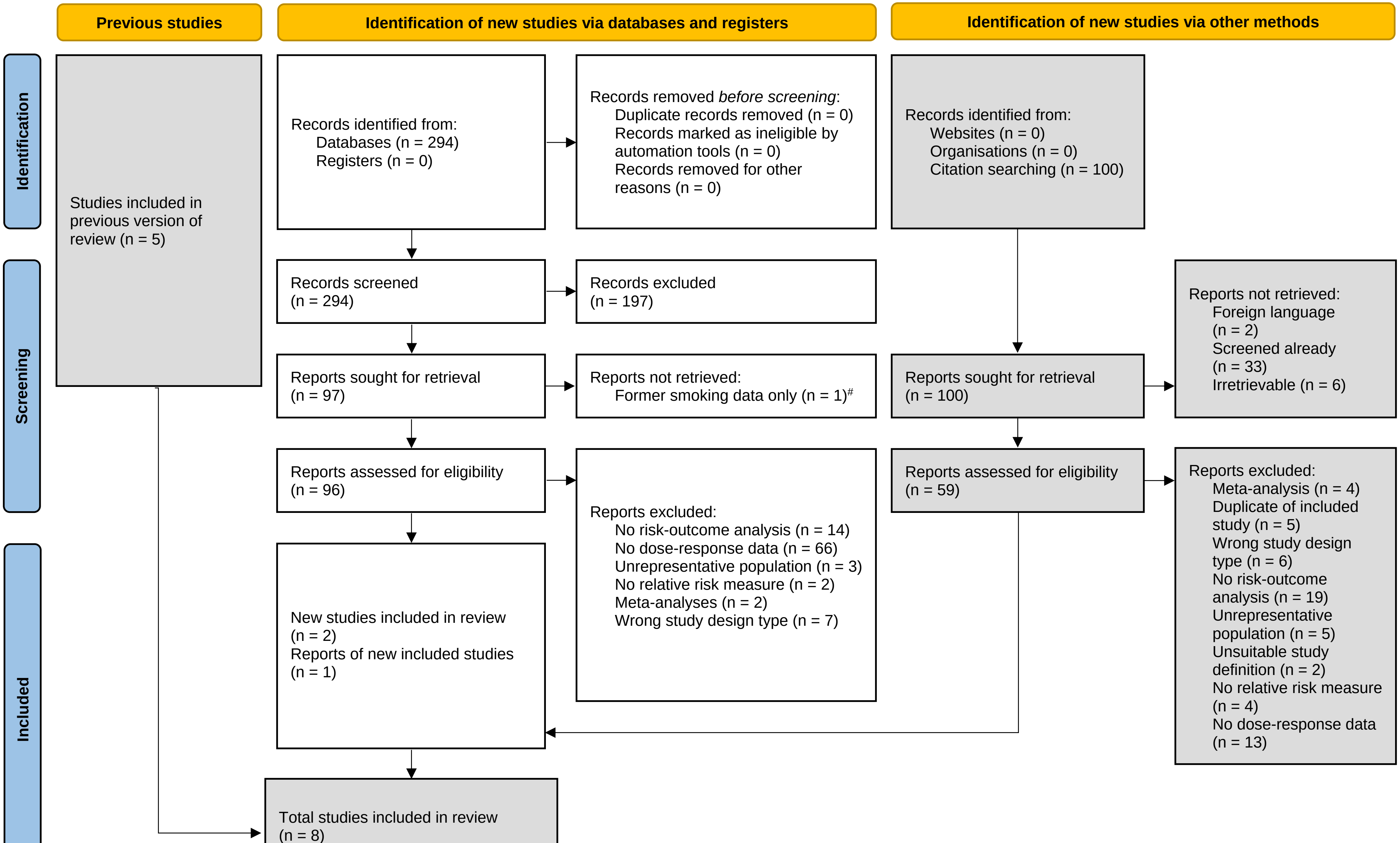

Studies with suitable former smoking data were identified and extracted, but they were not incorporated into GBD 2020 results

PRISMA 2020 flow diagram for an updated systematic review of the Smoking and Multiple sclerosis risk-outcome pair

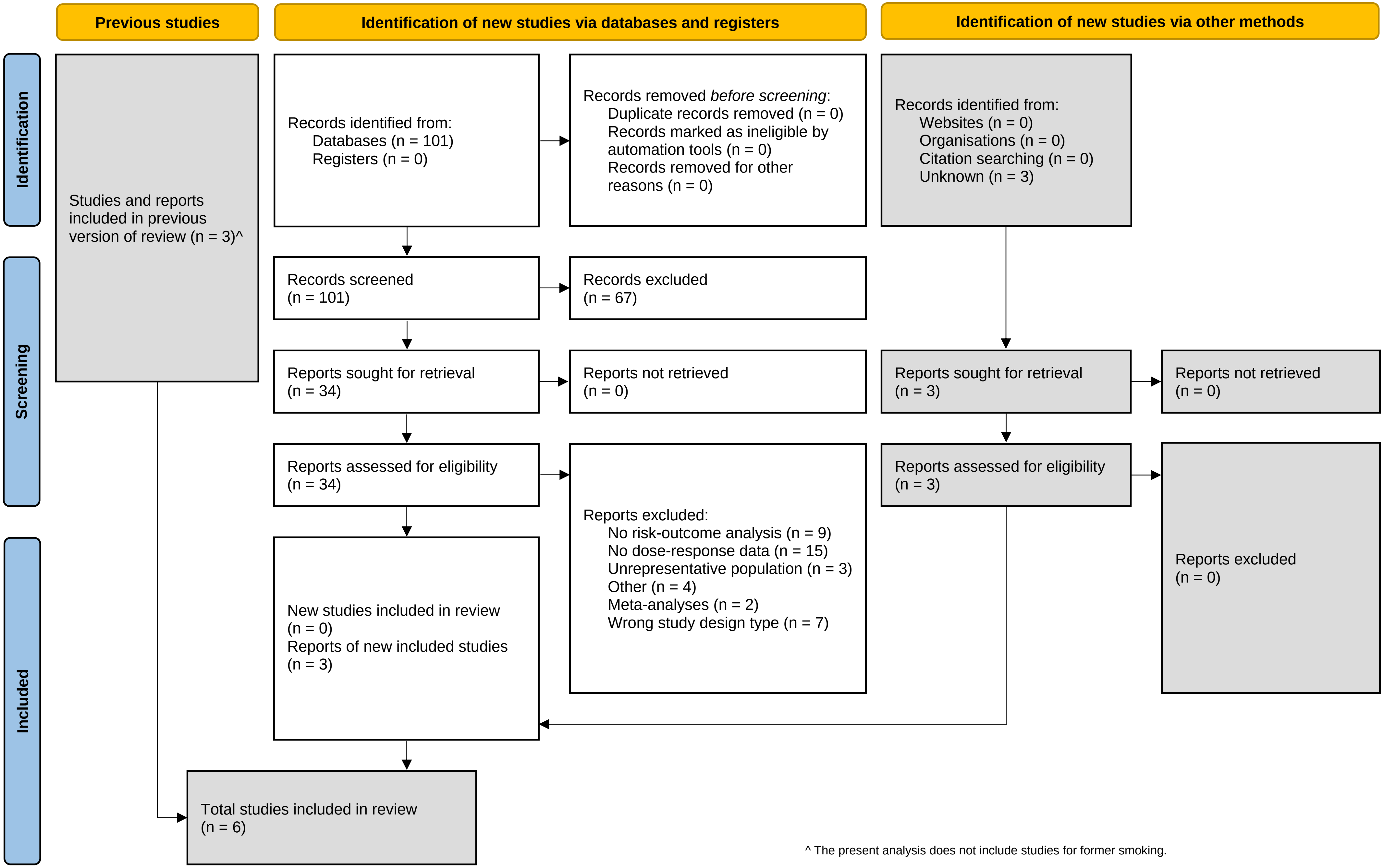

PRISMA 2020 flow diagram for an updated systematic review of the Smoking and Parkinson's disease risk-outcome pair

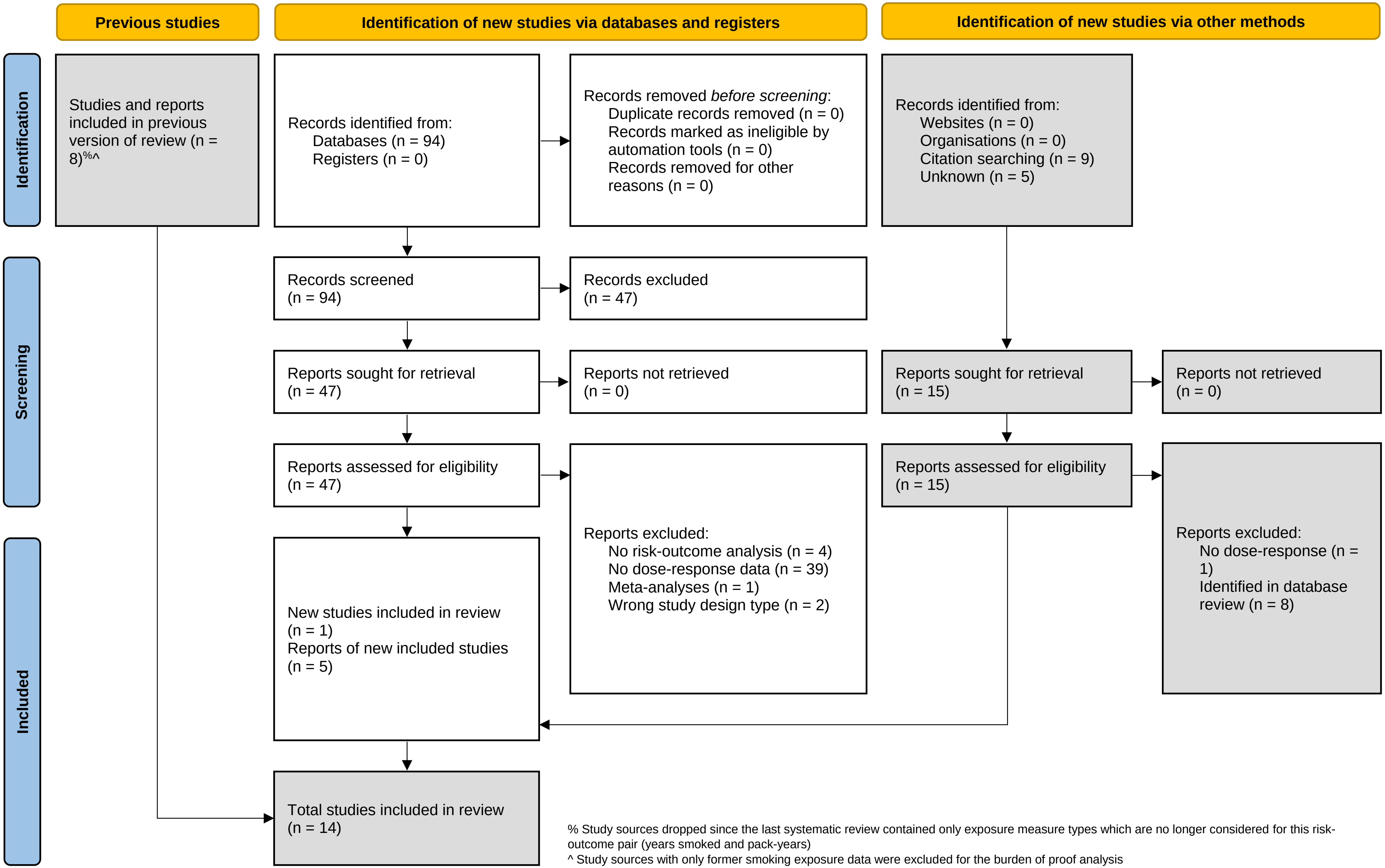

% Study sources dropped since the last systematic review contained only exposure measure types which are no longer considered for this risk-outcome pair (years smoked and pack-years)

^ Study sources with only former smoking exposure data were excluded for the burden of proof analysis

PRISMA 2020 flow diagram for an updated systematic review of the Smoking and Asthma risk-outcome pair

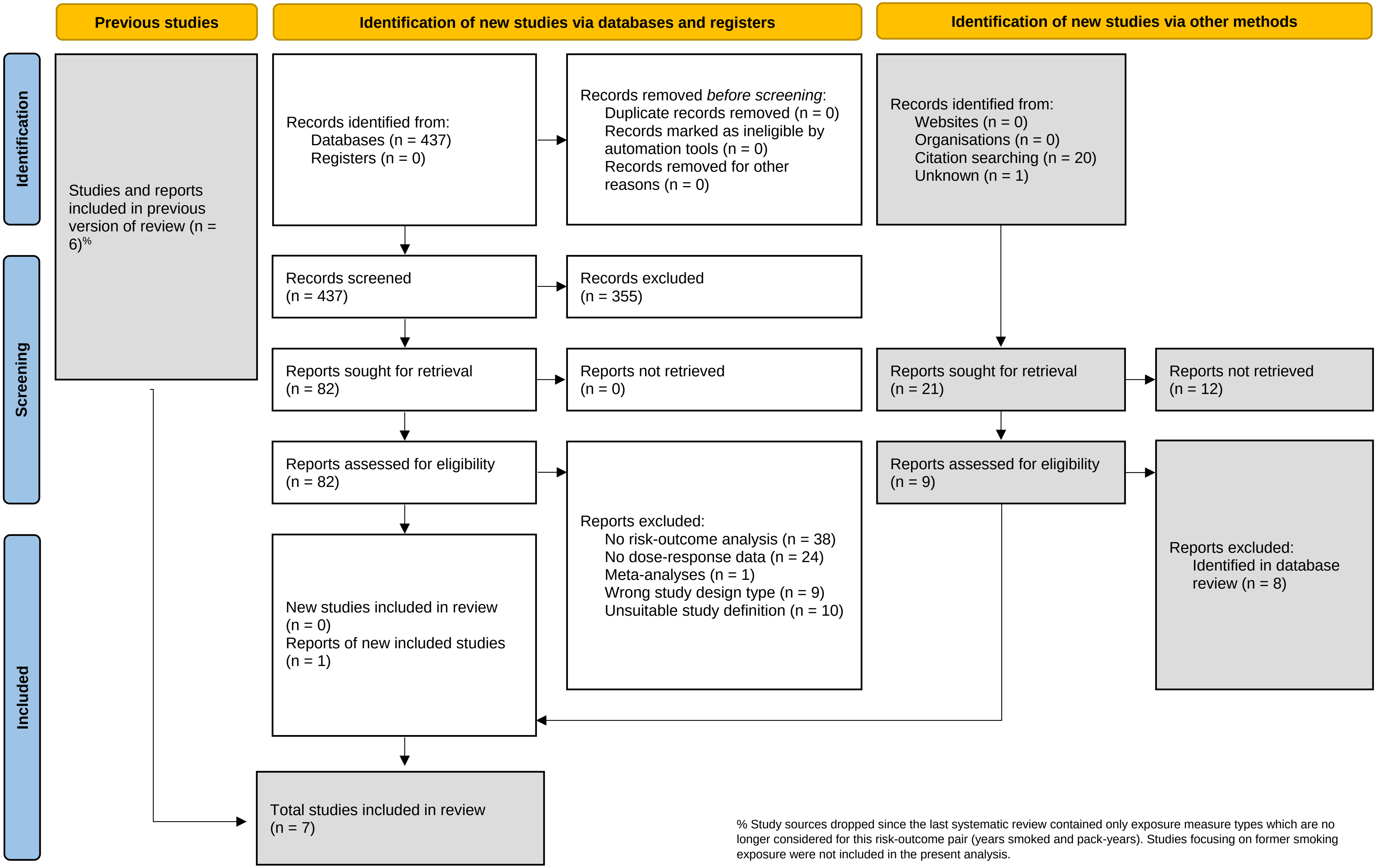

% Study sources dropped since the last systematic review contained only exposure measure types which are no longer considered for this risk-outcome pair (years smoked and pack-years). Studies focusing on former smoking exposure were not included in the present analysis.

PRISMA 2020 flow diagram for an updated systematic review of the Smoking and Cataract risk-outcome pair

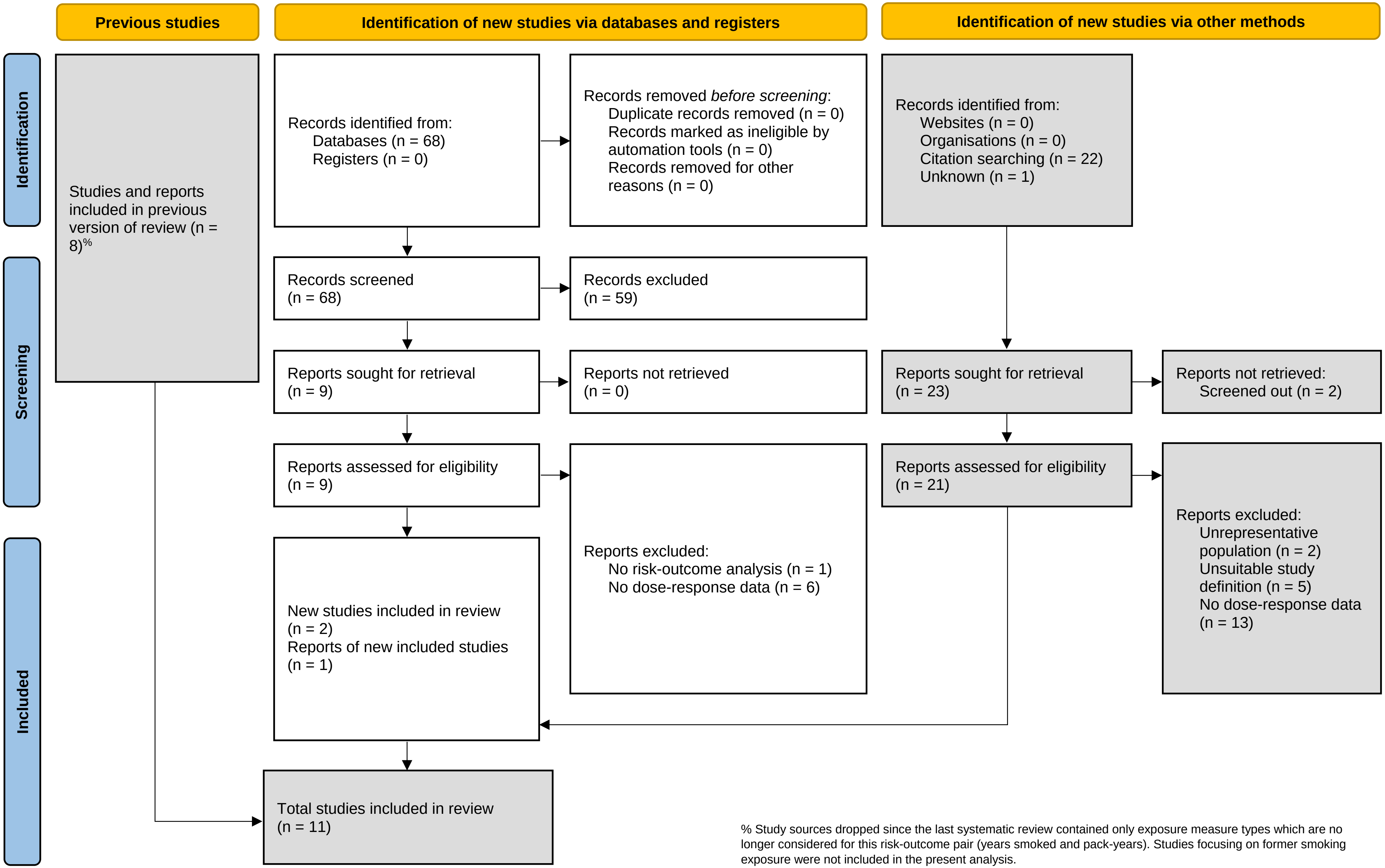

% Study sources dropped since the last systematic review contained only exposure measure types which are no longer considered for this risk-outcome pair (years smoked and pack-years). Studies focusing on former smoking exposure were not included in the present analysis.

PRISMA 2020 flow diagram for an updated systematic review of the Smoking and Age-related macular degeneration risk-outcome pair

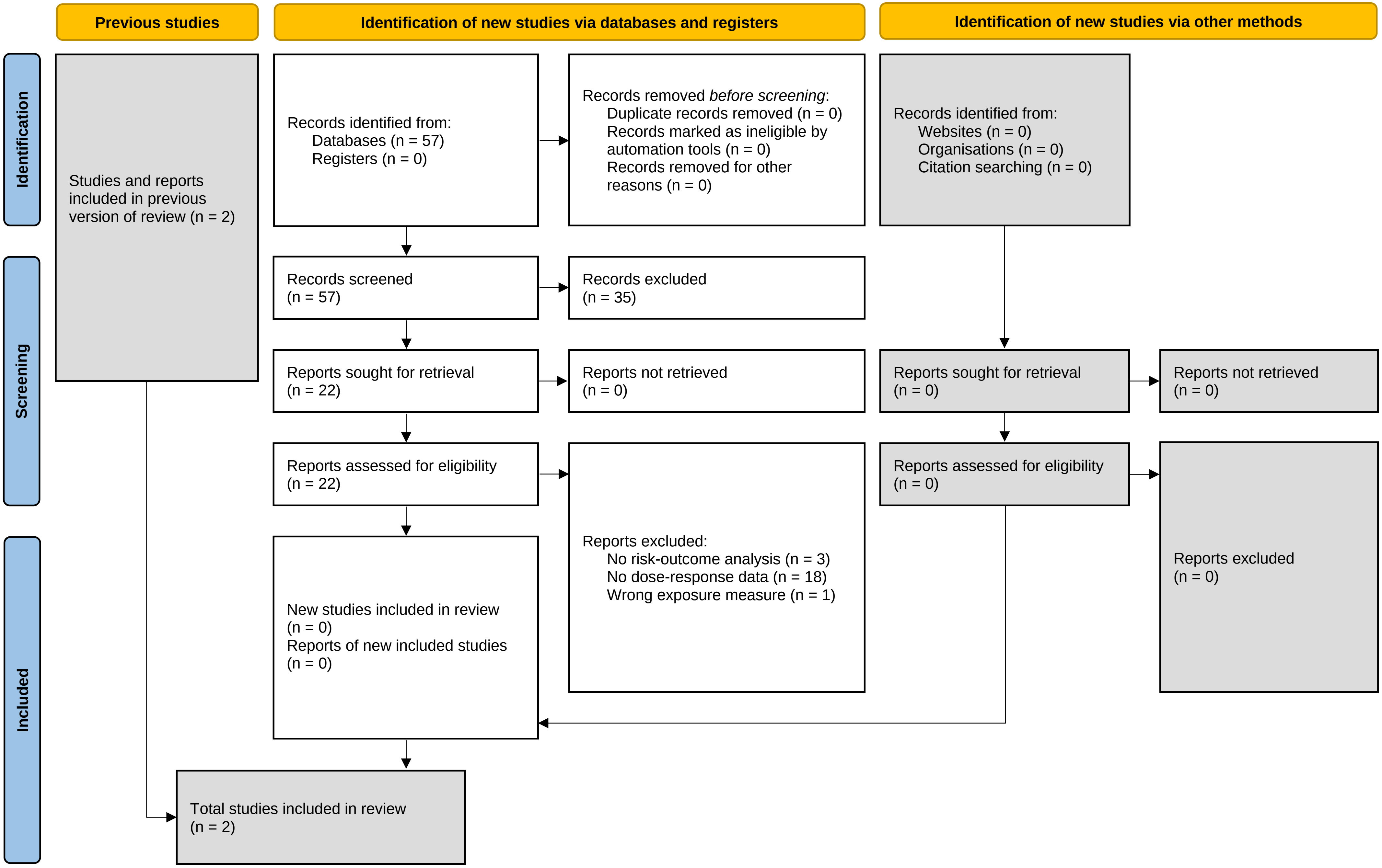

PRISMA 2020 flow diagram for an updated systematic review of the Smoking and Tuberculosis risk-outcome pair

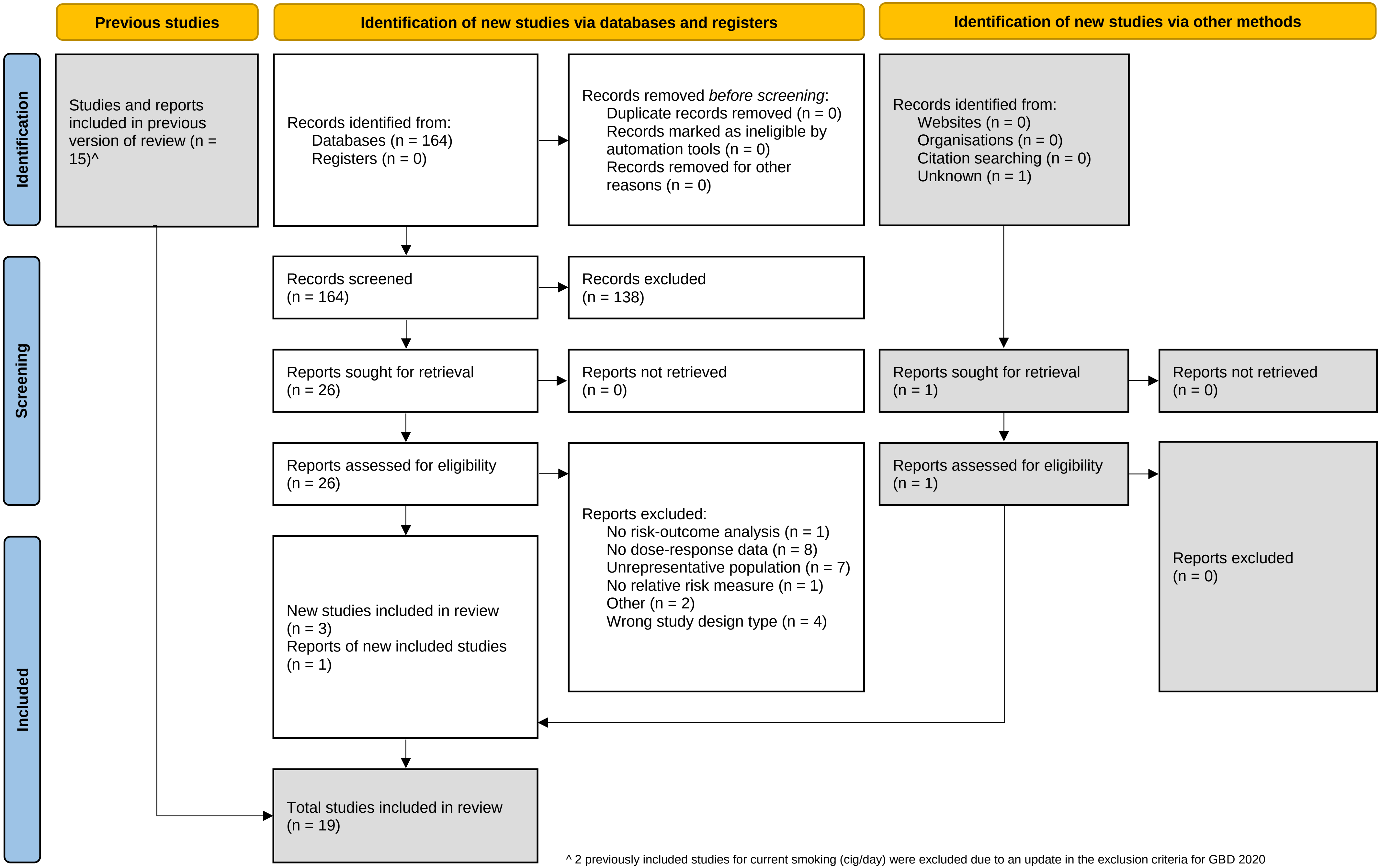

^ 2 previously included studies for current smoking (cig/day) were excluded due to an update in the exclusion criteria for GBD 2020

PRISMA 2020 flow diagram for a new systematic review of the Smoking and Fractures risk-outcome pair

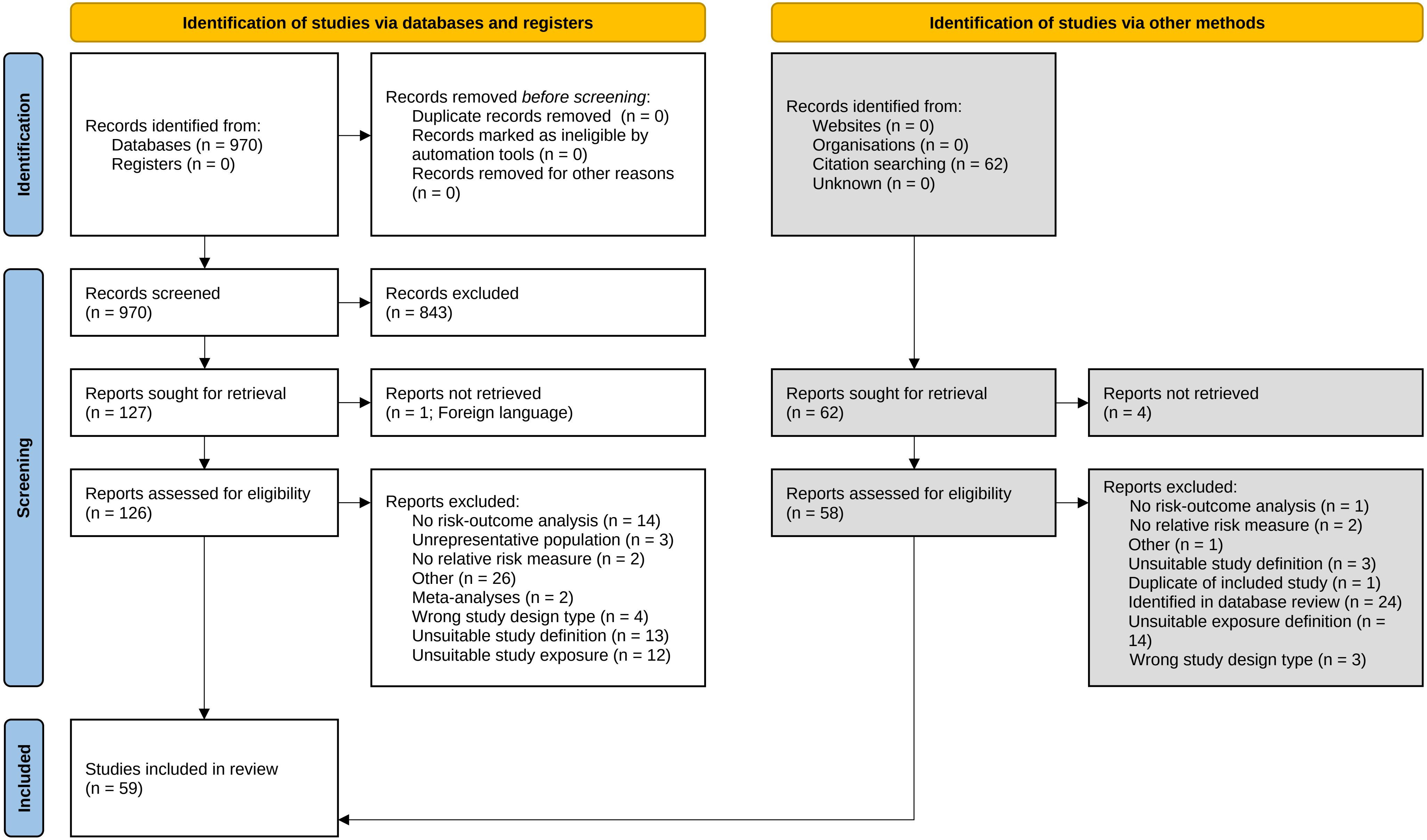

Section 2: Data inputs

Section 2.1: Study characteristics

Table 3: Summary of study characteristics

Cause	NID	Underlying NID	Author	Year	Study name	Population	Location	Study design	Sex	Follow-up	Age start	Age end	Exposure assessment	Endpoint	Disease ascertainment	Person-years	Events	Sample size	Outcomes	Cases	Controls	Control pool	Exposed
aortic_aneurism	345571		Kahn	1966	U.S. Veterans Study	U.S. veterans who held U.S. government life insurance policies in 1953	United States	Prospective cohort	Both	8.5	31	84	Self-administered questionnaire	Mortality	Vital records	2265674	491	293658	Nonsyphilitic aneurysm of aorta				
cvd	465049		Hammond	1966	Cancer Prevention Study I (CPS I)	Individuals in 25 states other than mobile communities and individuals in long-term medical institutions	United States	Prospective cohort	Both		35	84	Questionnaire	Mortality	Vital records, physician confirmation	3764571		1003229	Aortic aneurysm				
stroke	344362		Ostfeld	1974		Noninstitutionalized Black and white adults receiving Old Age Assistance in Cook County, IL	United States	Prospective cohort	Both	2.675	65	74	Administered questionnaire	Incidence	Medical records, Medical examination		312	3141	Stroke				
stroke	122237		Okada	1976		Residents of two rural communities	Japan	Prospective cohort	Both	7	40	79	Self-administered questionnaire	Incidence	Medical records, vital records	36696	69	3778	Cerebral hemorrhage				
stroke	343647		Doll	1976		British male doctors	United Kingdom	Prospective cohort	Male	20	20	99	Postal questionnaire	Mortality	Registrars-General of the United Kingdom, General Medical Council, other registries		616	34440	Cerebral thrombosis				

cvd	464286		Petitti	1978		White women in Northern California	United States	Case-control	Female	33	58		Self-administered questionnaire	Incidence	Discharge records, vital records, hospital admission records				Subarachnoid hemorrhage	11	3956	Population	
ihd	355955		Doll	1980	British Doctors' Study	Doctors residing in the United Kingdom	United Kingdom	Prospective cohort	Female	22	20	99	Mailed questionnaire	Mortality	Vital records		179	9164	Ischemic heart disease				
lri	355955		Doll	1980	British Doctors' Study	British female doctors	United Kingdom	Prospective cohort	Female	22	18	99	Self-administered questionnaire	Mortality	Vital records		44	6194	Pneumonia				
aortic_aneurism	350131		Rogot	1980	U.S. Veterans Study	U.S. veterans who held U.S. government life insurance policies in 1953	United States	Prospective cohort	Both	16	31	84	Self-administered questionnaire	Mortality	Vital records		900	248046	Aortic aneurysm (ICD-7, 451)				
aortic_aneurism	355955		Doll	1980	British Doctors' Study	Female British doctors listed on teh British Medical Register	United Kingdom	Prospective cohort	Female	22	18	99	Self-administered questionnaire	Mortality	Vital records		11	6194	Aortic aneurysm(ICD-7)				
parkinson	355955		Doll	1980	British Doctors' Study	Female doctors who resided in the United Kingdom	United Kingdom	Prospective cohort	Female	22	18	99	Mailed questionnaire	Mortality	Administrative records		5	6194	Parkinsonism				
parkinson	355955		Doll	1980	British Doctors' Study	Female doctors who resided in the United Kingdom	United Kingdom	Prospective cohort	Female	22	18	99	Mailed questionnaire	Mortality	Administrative records		5	6194	Parkinsonism				
ihd	359114		Willett	1981		Married registered nurses in 11 US states	United States	Case-control	Female		30	55	Mailed questionnaire	Hospitalization	Self-reported, medical records				Acute myocardial infarction	249	4977	Population	
gallbladder_diseases	351491		Layde	1982	Oxford/Family Planning Association Contraceptive Study	Females who attended family planning	United Kingdom	Prospective cohort	Female	9.1	25	39	Interview	Incidence	Surgically-confirmed gallbladder disease ascertained	154400	227	17032	Surgically-confirmed gallbladder disease (cholelithi				

						clinics and using oral contraceptives, an intrauterine device, or a diaphragm for birth control									from hospital admissions summaries and diagnoses				asis (ICD-8 574) or cholecystitis (ICD-8 575, with the surgical code for cholecystectomy)				
stroke	334967		Fuller	1983	Whitehall Study	Male civil servants	United Kingdom	Prospective cohort	Male	10	40	64	Screening survey	Mortality	Central registry		84	16484	Stroke				
ihd	357948		Rosenberg	1983		Females enrolled in a study on oral contraceptive use	United States	Case-control	Female		25	49	Standard questionnaire	Hospitalization	Clinical diagnosis				Acute myocardial infarction	255	796	Hospital	
ihd	359320		Kaufman	1983		Patients in 78 hospitals in the northeast	United States	Case-control	Male		30	54	Administered questionnaire	Hospitalization	Clinical diagnosis, medical review				Nonfatal first myocardial infarction	502	835	Hospital	
cvd	359320		Kaufman	1983		Patients in 78 hospitals in the northeast	United States	Case-control	Male	30	54		Administered questionnaire	Hospitalization	Clinical diagnosis, medical review				Nonfatal first myocardial infarction	502	835	Hospital	
fractures	498442		Seeman	1983		Male patients at the Metabolic Bone Disease Clinic with vertebral fractures due to spinal osteoporosis	United States	Case-control	Male		44	85	Administered questionnaire	Incidence	Medical reports				Vertebral fractures due to osteoporosis	105	105	Men with Pagel's disease	
cvd	441318		Khaw	1984		Adult residents of a white, upper-middle class community in Southern California	United States	Prospective cohort	Both	9	65	84	Standardized interview	Mortality	Vital records, family report, hospital and clinical records		73	2107	Stroke				
ihd	328460		Kono	1985		Male Japanese doctors in 9 prefectural	Japan	Prospective cohort	Male	12.7	18	99	Administered questionnaire	Mortality	Vital records		121	5446	Coronary heart disease (ICD-8 410-414)				

						medical associations																	
stroke	328460		Kono	1985		Male Japanese doctors in 9 prefectural medical associations	Japan	Prospective cohort	Male	12.7	18	99	Administered questionnaire	Mortality	Vital records		154	5446	Stroke (430-438)				
lung_cancer	358213		Vena	1985		White patients admitted to Roswell Park Memorial Institute	United States	Case-control	Male		35	79	Pre-admission questionnaire	Incidence	Clinical records				Lung cancer	1002	2121	Hospital	
ihd	359113		Rosenberg	1985		Patients in 78 hospitals in the northeast	United States	Case-control	Male		20	54	Administered questionnaire	Hospitalization	Admission records				First myocardial infarction	1873	2775	Hospital	
stroke	350743		Bonita	1986		Residents of the Auckland region	New Zealand	Case-control	Both		35	64	Administered questionnaire	Incidence	Medical diagnosis				Subarachnoid hemorrhage	115	1586	Population survey	
stroke	358506		Bonita	1986		Residents of Auckland	New Zealand	Case-control	Both		35	64	Administered questionnaire	Incidence	Medical diagnosis				Stroke	132	1586	Population	
liver_cancer	343515		Austin	1986		Patients and controls from 12 hospitals affiliated with one of five participating study centers in the US	United States	Case-control	Both		18	80	Personal interviews	Incidence	Medical records, autopsy records				Hepatocellular carcinoma	86	161	Hospital based	
cvd	344312		Abbott	1986	Honolulu Heart Program	Men of Japanese ancestry on Oahu	United States	Prospective cohort	Male	12	45	69	Self-reported	Incidence or Mortality	Hospital records, vital records, autopsy reports		288	7872	Ischemic stroke; Hemorrhagic stroke; Stroke				
cvd	350743		Bonita	1986		Residents of the Auckland region	New Zealand	Case-control	Both	35	64		Administered questionnaire	Incidence	Medical diagnosis				Subarachnoid hemorrhage	115	1586	Population survey	
aortic_aneurism	347380		Carstensen	1987		Random sample of	Sweden	Prospective cohort	Male	15	18	69	Self-administered questionnaire	Mortality	Vital records		15	25129	Aortic aneurysm				

						Swedish men													(ICD-8, 441)				
lung_cancer	193987		Brownson	1987		White adults in Denver metropolitan area	Colorado, USA	Case-control	Both		30	99	Personal interview	Incidence	Disease registry				Lung denocarcinoma (ICD 163)	102	233	Disease registry	
alzheimer_other_dementia	358838		Shalat	1987		Male patients and residents of eastern Massachusetts	Massachusetts, United States	Case-control	Male		18	99	Self-administered questionnaire	Incidence	Clinical records, autopsy records				Alzheimer's	98	162	General population	
parkinson	359161		Rajput	1987		Residents of Rochester	United States	Case-control	Both		40	99	Medical records	Incidence	Medical records				Idiopathic Parkinson's disease	118	236	Rochester residents who attended the same medical facility around the same time as the cases	
parkinson	359161		Rajput	1987		Residents of Rochester	United States	Case-control	Both		40	99	Medical records	Incidence	Medical records				Idiopathic Parkinson's disease	118	236	Rochester residents who attended the same medical facility around the same time as the cases	
stroke	344359		Colditz	1988	Nurses' Health Study	Female registered nurses	United States	Prospective cohort	Female	8	30	55	Self-administered questionnaire	Incidence or Mortality	Self-reported, medical records, vital records	908447	274	118539	Total stroke (subarachnoid hemorrhage, intracerebral heorrhage, thromboembolic stroke, other)				

stroke	344360		Wolf	1988	Framingham Heart Study	Adults in Framingham , MA	United States	Prospective cohort	Both	26	36	68	Systematic examination	Incidence or Mortality	Medical records, Medical examinatio n		459	4255	Stroke or Transient ischemic attack				
cvd	432105		Syrjanen	1988		Patients from Helsinki University Central Hospital	Finland	Case-control	Both	17	49		Interview	Incidence	Medical diagnosis				Ischemic stroke	54	54	Populatio n	
cvd	464146		Oleckno	1988		Patients from four community hospitals in northern Illinois	United States	Case-control	Femal e	15	40		Medical records	Incidence	Disease registry, medical records				Stroke	54	864	Populatio n	
cataracts	350491		Flaye	1989	City Eye Study	Residents living around London	United Kingdom	Prospective cohort	Both	3	54	65	Self-administered questionnaire	Incidence	Physician diagnosis		95	1012	Nuclear opacity				
fractures	414225		Wickham	1989		Residents of eight study areas	United Kingdom	Prospective cohort	Femal e	15	65	99	Interview and medical assessment	Incidence	Medical records	7870	44	1340	Hip fracture				
ihd	357353		Gramenzi	1989		Coronary care units in northern Italy	Italy	Case-control	Femal e		22	69	PENDING ILLIAD	Hospitaliz ation	Hospital records				Acute myocardial infarction	262	519	Hospital	
ihd	357456		Quek	1989		Females admitted to the coronary care unit of the General Hospital in Kuala Lumpur	Malaysia	Case-control	Femal e		30	99	Administered interview	Hospitaliz ation	Clinical diagnosis based on clinical symptoms and test results				Acute myocardial infarction	264	437	Hospital	
ihd	359115		Palmer	1989		Patients in 78 hospitals in the northeast	United States	Case-control	Femal e		25	64	Administered questionnaire	Hospitaliz ation	Admission records				Nonfatal first myocardial infarction	910	2375	Hospital	
stroke	357431		Gill	1989		Patients in two medical centers	United Kingdom	Case-control	Both		20	70	Administered questionnaire	Hospitaliz ation	Medical records and clinical diagnosis				Stroke	621	573	Survey of industrial workers	
periphera l_artery_ disease	369290		Skalkidis	1989		Laikon Hospita in Athens	Greece	Case-control	Both		18	99	Interview	Incidence	Medical evaluation				Peripheral arterial occlusive disease	100	100	Hospital	

lbp	409803		Ryden	1989		Employees of the Children's Hospital and Health Center in San Diego	United States	Case-control	Both		18	99	Employee health records	Incidence	Employee health records				Low-back injury	84	168	Selected from the same populatio n as the cases	
cvd	357431		Gill	1989		Patients in two medical centers	United Kingdom	Case-control	Both	20	70		Administered questionnaire	Hospitaliz ation	Medical records and clinical diagnosis				Stroke	621	573	Survey of industrial workers	
cvd	464154		Thompson	1989		Women from 83 practices througout the UK	United Kingdom	Nested case-control	Femal e	45	69		Interview	Incidence	Medical records				Stroke; Ischemic heart disease; Cardiovasc ular diseases	603	1206	Cohort populatio n	
bladder_ cancer	344435		Burch	1989		Residents in the province of Alberta, Toronto, and southern-central Ontario	Canada	Case-control	Both		35	79	Interview	Incidence	Biomarker				Bladder cancer	835	792	From province-wide, annually updated listings	
fractures	498444		Kleerekop er	1989		Postmenopa usal white females	Michigan, United States	Retrospectiv e Cohort	Femal e		45	75	Questionnaire	Incidence	Hospital records		529	663	Vertebral and Non-vertebral fracture				
copd	343461		Hirayama	1990	Six Prefecture Cohort Study	General population of six prefectures from across Japan	Japan	Prospective cohort	Both	14.5	40	99	Self-report	Mortality	Administra tive medical records or disease registries, death certificates	384963 7		265118	Cancer of the stomach (as coded by ICD-7)				
ihd	343461		Hirayama	1990	Six Prefectures Study	Residents of 29 health center districts	Japan	Prospective cohort	Both	17	40	99	Administered questionnaire	Mortality	Disease registry, vital records	384963 7	3548	265118	Ischemic heart disease				
peptic_ul cer	262563		Anda	1990	First National Health and Nutrition Examination Survey Epidemiolo	Noninstituti onalized female adult population of the United States	United States	Prospective cohort	Femal e	9	24	76	Interview	Incidence	Self-reported physician diagnosis, hosptial discharge records,		140	2851	Gastric ulcer, duodenal ulcer, or peptic ulcer site unspecifie				

					gic Follow-up Study (NHEFS)										death certificates				d (ICD-9 531.0-533.9)				
prostate_cancer	347383		Hsing	1990	Lutheran Brotherhood Cohort	White male insurance policy holders	United States	Prospective cohort	Male	10	35		Mailed quesionnaire	Mortality	Vital records	286731	149	26030	Prostate cancer				
parkinson	359307		Sasco	1990		Males who attended either Harvard of the University of Pennsylvania	United States	Nested case-control	Male		15	25	Administrative records	Incidence	Self-report, death certificates				Parkinson's disease	96	384	Live subjects from the same cohort as the cases who self-reported in the 1976-78 questionnaire as not having Parkinson's disease	
breast_cancer	310487		Chu	1990		General population	United States	Case-control	Female		20	54	Interview	Incidence	Population-based cancer registries				Histologically-confirmed breast cancer	4730	4688	Residents from the same geographic areas as the cases	
cvd	343461		Hirayama	1990	Six Prefectures Study	Residents of 29 health center districts	Japan	Prospective cohort	Both	17	40	99	Administered questionnaire	Mortality	Disease registry, vital records	3849637	3548	265118	Ischemic heart disease				
cvd	464527		Harmsen	1990	Multifactor Primary Prevention Study	Men in Goteborg	Sweden	Prospective cohort	Male	11.8	47	55	Postal questionnaire	Incidence or Mortality	Disease registry, vital records		230	7495	Subarachnoid hemorrhage; Intracerebral hemorrhage; Ischemic stroke; Stroke				
parkinson	359307		Sasco	1990		Males who attended either Harvard of	United States	Nested case-control	Male		15	25	Administrative records	Incidence	Self-report, death certificates				Parkinson's disease	96	384	Live subjects from the same	

						the University of Pennsylvania																cohort as the cases who self-reported in the 1976-78 questionnaire as not having Parkinson's disease	
bladder_cancer	502468		Harris	1990		Hospital patients	United States	Case-control	Both		24	NA	Structured interview	Incidence	Hospital records				Bladder cancer	1663	4930	Patients with with primary diagnoses judged unrelated to tobacco and/or alcohol use	
prostate_cancer	343354	347952	Hsing	1991	US Veterans' Cohort	Veterans who served in the Armed forces between 1917 and 1940	United States	Prospective cohort	Male	26	31	84	Questionnaire	Mortality	Insurance records		4607	293916	Prostate cancer				
stroke	334410		Kuller	1991	Multiple Risk Factor Intervention Trial (MRFIT)	Men screened at 22 centers	United States	Prospective cohort	Male	10.5	35	57	Screening survey	Mortality			262	361662	Stroke				
stroke	343297		Shaper	1991	British regional heart study	Patients at general practices in 24 towns	United Kingdom	Prospective cohort	Male	8	40	59	Administered questionnaire	Incidence or Mortality	Vital records, Disease registry		110	7735	Stroke (ICD 430-8)				
lung_cancer	193948		Liu	1991		Farmers in Xuanwei, China	China	Case-control	Male		18	99	Administered questionnaire	Incidence	Pathological diagnosis or clinical history				Lung cancer	56	224	Population	
lung_cancer	355963		Potter	1991		Women from the 1985 Iowa	Iowa, United States	Nested case-control	Female		55	69	Mailed questionnaire	Incidence	Disease registry				Lung cancer (ICD 0-162)	109	2009	Population	

						driver's license list																	
lung_cancer	357961		Becher	1991		Hospital cases and controls and population controls from residence registries in the northwest	Germany	Case-control	Both		33	90	Administered interviews	Incidence	Clinical records of histologically confirmed cases				Histologically confirmed lung cancer	194	582	Hospital, Population	
cvd	408694		Knekt	1991	Social Insurance Institution	Residents of 34 rural, semiurban, and industrial communities	Finland	Prospective cohort	Both	5	20	69	Self-administered questionnaire	Hospitalization or Mortality	Hospital discharge records, vital records	503485	187	42862	Subarachnoid hemorrhage				
cvd	465269		Mangion	1991		Elderly population attending a medical outpatient clinic	New Zealand	Cross-sectional	Both	68	92		Self-report	Prevalence	Biomarker		147	295	Peripheral artery disease				94
bladder_cancer	502452		Burns	1991		Residents of the metropolitan Detroit, Michigan (United States)	Michigan, United States	Case-control	Both		40	84	Telephone interview	Incidence	Cancer registry				Bladder cancer	2160	3979	Controls were persons diagnosed with cancer of the colon or rectum	
fractures	261377		Paganini-Hill	1991	Leisure World study	All residents of Leisure World, Laguna Hills	Los Angeles, California	Prospective cohort		4.8	≤65	≥85	Self-administered questionnaire	Incidence	Hospital records	66785	86	13987	Hip fracture				
fractures	498410		Vecchia	1991		Woemn admitted to network of teaching and general hospitals in the greater Milan area	Milan, Italy	Case-control	Female		29	74	Questionnaire	Incidence	Medical reports				Hip/proximal femur fracture	209	1449	Patients at same network of hospitals admitted for non-traumatic, acute conditions	

lung_cancer	355961		Chyou	1992	Honolulu Heart Program	American men of Japanese ancestry in Oahu	Hawaii, United States	Prospective cohort	Male	22	46	65	Administered interview	Incidence	Disease surveillance of hospitals with tissue confirmation		212	8009	Lung cancer (ICD-8 162.1)				
cataracts	350493		Hankinson	1992	Nurses' Health Study	Female registered nurses living in one of 11 US states	United States	Prospective cohort	Female	8	45	67	Self-administered questionnaire	Incidence	Medical records, physician diagnosis	470302	493	50828	Senile cataract, cataract extraction				
cataracts	359119		Christen	1992	Physicians' Health Study	Male physicians in the US	United States	Prospective cohort	Male	5	40	84	Self-administered questionnaire	Incidence	Medical records		557	17824	Cataract				
gallbladder_diseases	350721		Stampfer	1992	Nurses' Health Study	Female registered nurses living in eleven large states in the United States	United States	Prospective cohort	Female	6.7	34	59	Mailed questionnaire	Incidence	Self-reported in mailed questionnaire	607104	2610	90302	Cholecystectomy or symptomatic but unremoved gallstones				
lung_cancer	357351		Jockel	1992		Patients and hospital controls from seven hospitals in five German cities, and population controls from the city areas	Germany	Case-control	Both		38	87	Administered interview	Incidence	Clinical records of histologically confirmed lesions				Lung cancer	194	582	Population	
lung_cancer	358479		Chiazze	1992		Participants in the Newark (TIMA) plant cohort	Ohio, United States	Nested case-control	Both		18	99	Administered interviews	Mortality	Death records coded by a qualified nosologist				Lung cancer	144	404	Population	
ihd	309699		Kalandidi	1992		Patients at the Hippokrateion Hospital	Greece	Case-control	Both		30	99	Interviews	Hospitalization	Diagnosis with hospitalization				Coronary heart disease	329	570	Hospital	
stroke	298316		Longstreth	1992		Residents of King County, Washington	United States	Case-control	Both		18	99	Structured in-person interview	Incidence	Disease surveillance, emergency medical system reports,				Subarachnoid hemorrhage	149	298	Population	

															medical records								
parkinson	359162		Jimenez-Jimenez	1992		Population from within a single health area of Madrid	Spain	Case-control	Both		18	99	Interview	Incidence	Medical records				Parkinson's disease	128	256	Patients at the same hospital as the cases who attended the emergency room with nonneurological ailments	
stomach_cancer	340554		Hoshiyama	1992		Cases and controls admitted to the Saitama Cancer Center Hospital, which covers seven provincial cities and two town within its vicinity; additional population controls were selected from across Saitama prefecture	Japan	Case-control	Both		18	99	Interview	Incidence	Physician diagnosis				Newly diagnosed adenocarcinoma of the stomach	294	496	Hosptial controls: inpatients without neoplasms, without digestive diseases, lived in Saitama prefecture for at least 10 years, and not on a long-term special diet for medical reasons; Population controls: Study area resident randomly selected from the electoral roll	

laryngeal_cancer	298147		Zheng	1992		Residents of urban Shanghai	China	Case-control	Male		20	75	Structured interview	Incidence	Disease registry				Laryngeal cancer	177	269	General population	
nasopharyngeal_cancer	346030		Nam	1992		Randomly selected white Americans who died of nasopharyngeal carcinoma and those that died of causes unrelated to smoking and alcohol use	United States	Case-control	Both		18	99	Self-administered questionnaire	Mortality	Vital records				Nasopharyngeal carcinoma	204	408	General population	
kidney_cancer	358807		McCredie	1992		Residents of New South Wales	Australia	Case-control	Both		20	79	Self-administered questionnaire	Incidence	Disease registry				Kidney cancer, renal cell carcinoma	489	523	General population	
cvd	298316		Longstreth	1992		Residents of King County, Washington	United States	Case-control	Both	18	99		Structured in-person interview	Incidence	Disease surveillance, emergency medical system reports, medical records				Subarachnoid hemorrhage	149	298	Population	
cvd	294115		Huhtasaari	1992		Male residents of northern Sweden	Sweden	Case-control	Male	35	54		Self-administered questionnaire	Incidence	Physician diagnosis, discharge registers, vital records				Ischemic heart disease	585	589	Population	
cvd	464829		Woo	1992		Chinese patients admitted to a district general hospital in Hong Kong	Hong Kong	Case-control	Both	45	69		Self-administered-questionnaire	Hospitalization	Clinical diagnosis, autopsy				Ischemic stroke; Intracerebral hemorrhage	278	278	Population	
parkinson	359162		Jimenez-Jimenez	1992		Population from within a single health area of Madrid	Spain	Case-control	Both		18	99	Interview	Incidence	Medical records				Parkinson's disease	128	256	Patients at the same hospital as the cases who	

																						attended the emergency room with nonneurological ailments	
kidney_cancer	502480		McLaughlin	1992		All residents of urban Shanghai	Shanghai, China	Case-control	Both		35	74	Structured interview	Incidence	Cancer registry				Kidney cancer	223	157	From the general population of the Shanghai urban area	
fractures	261424		Kiel	1992	Framingham Study	Residents of Framingham, Massachusetts		Prospective cohort	Female	NA	28	62	Questionnaire	Incidence	Hospital records, self-report	NA	207	2673	Hip fracture				
lung_cancer	355965		Chyou	1993	Honolulu Heart Program	American men of Japanese ancestry in Oahu	Hawaii, United States	Prospective cohort	Male	22	46	65	Administered interview	Incidence	Disease surveillance		227	7733	Lung cancer				
stroke	334028		Kawachi	1993	Nurses' Health Study	Female registered nurses	United States	Prospective cohort	Female	12	30	55	Self-administered questionnaire	Incidence or Mortality	Medical records, Autopsy reports, vital records	1370000	448	117006	Total stroke				
multiple_sclerosis	350717		Villard-Mackintosh	1993	Oxford FPA study	Married women	United Kingdom	Prospective cohort	Female	23	25	50	Questionnaire	Onset	Clinical records and diagnosis	275867	63		Multiple sclerosis				
rheumatoid_arthritis	261510		Heliovaara	1993	Social Insurance Institution's Mobile Clinic Health Examination Survey	Urban, rural and factory worker populations of 34 study regions distributed over the whole of Finland	Finland	Prospective cohort	Both	18.6	15	99	Mailed questionnaire	Incidence	Population register	981184	512	52809	Rheumatoid arthritis				
lung_cancer	357757		Risch	1993		Adults in metropolitan Toronto area and St. Catharine's-	Canada	Case-control	Both		30	79	Administered questionnaire	Incidence	Clinical records				Lung cancer	550	1100	Population	

						Niagra Falls region of southern Ontario																	
lung_cancer	358161		Brockmoller	1993		Patients from one specialized hospital in Berlin	Germany	Case-control	Both		32	84	Administered interviews	Incidence	Clinical records				Lung cancer	117	272	Hospital	
stroke	358476		Juvela	1993		Patients at the Helsinki University Hospital	Finland	Case-control	Both		15	60	Structured administered questionnaire	Hospitalization	Medical records				Aneurysmal subarachnoid hemorrhage	278	314	Hospitalized patients	
cvd	334028		Kawachi	1993	Nurses' Health Study	Female registered nurses	United States	Prospective cohort	Female	12	30	55	Self-administered questionnaire	Incidence or Mortality	Medical records, Autopsy reports, vital records	1370000	448	117006	Total stroke				
cvd	309715		Prineas	1993		Women on Iowa's driver registration list	United States	Prospective cohort	Female	4	55	69	Mailed questionnaire	Mortality	Vital records	114962	93	41837	Ischemic heart disease				
cvd	359224		Freund	1993	Framingham Cohort	Residents of Framingham, Massacheussettes	United States	Prospective cohort	Both	34	45	84	Self-reported in medical examination	Incidence or Mortality	Physician review of medical records, hospital records, vital records	102578	1826	4503	Ischemic heart disease; Cardiovascular diseases				
cvd	358476		Juvela	1993		Patients at the Helsinki University Hospital	Finland	Case-control	Both	15	60		Structured administered questionnaire	Hospitalization	Medical records				Aneurysmal subarachnoid hemorrhage	278	314	Hospitalized patients	
cvd	120237		Vogt	1993		General female population of Pittsburgh, Pennsylvania	United States	Cross-sectional	Female	65	93		Study of Osteoporotic Fractures Questionnaire	Prevalence	Biomarker		82	1491	Peripheral artery disease				147
bladder_cancer	502462		Chyou	1993		American men of	Hawaii	Prospective cohort	Male	22	49	68	Interview	Incidence	Hospital surveillanc	175890	96	7995	Bladder cancer				

						Japanese ancestry									e and cancer registry								
prostate_ cancer	347385		Hiatt	1994		Members of a pre-paid health plan in northern California	United States	Prospective cohort	Male	4.6	30		Questionnaire	Incidence	Insurance disease registry, hospital records	199254	238	43432	Prostate cancer				
stroke	343400	344308	Robbins	1994	Physicians' Health Study	US male physicians	United States	Prospective cohort	Male	9.7	40	84	Postal questionnaire	Incidence or Mortality	Self-reported, medical records, familial reporting	207579	340	22071	Total stroke, ischemic stroke, hemorrhagic stroke				
lri	173863		Doll	1994	British Doctors' Study	British male doctors	United Kingdom	Prospective cohort	Male	40	18	99	Self-administered questionnaire	Mortality	Vital records		864	34439	Pneumonia				
tb	173863		Doll	1994	British Doctors' Study	Male doctors who resided in the United Kingdom	United Kingdom	Prospective cohort	Male	40	18	99	Mailed questionnaire	Mortality	Administrative records		66	10812	Pulmonary tuberculosis				
asthma	173863		Doll	1994	British Doctors' Study	British male doctors	United Kingdom	Prospective cohort	Male	40	18	99	Self-administered questionnaire	Mortality	Vital records		70	34439	Asthma				
aortic_an eurism	173863		Doll	1994	British Doctors' Study	British male doctors	United Kingdom	Prospective cohort	Male	40	18	99	Self-administered questionnaire	Mortality	Vital records		331	34439	Aortic aneurysm				
parkinson	173863		Doll	1994	British Doctors' Study	Male doctors who resided in the United Kingdom	United Kingdom	Prospective cohort	Male	40	18	99	Mailed questionnaire	Mortality	Administrative records		152	10812	Parkinsonism				
lung_can cer	357765		Sankaranarayanan	1994		Patients, visitors, and bystanders at the Regional Cancer Center	Kerala, India	Case-control	Both		30	99	Administered interviews	Incidence	Hospital disease registry				Lung cancer	281	1488	Hospital visitors	
lung_can cer	419667		Suzuki	1994		Hospital-based cases and controls from Rio de Janiero, Brazil	Brazil	Case-control	Both		30	89	Administered interview	Incidence	Histological confirmed cases				Lung carcinomas	123	246	Hospital	
stroke	357445		Jamrozik	1994		Residents of Perth	Australia	Case-control	Both		18	99	Semi-structured	Incidence	Disease registry				First stroke	501	931	Population	

													interview and questionnaire										
stroke	358540		Hannaford	1994		Participants of the RCGP Oral Contraception Study recruited by general practitioners	United Kingdom	Nested case-control	Female		21	70	Medical records	Incidence	Physician-reported				First stroke (ICD-8 4300-4389) or amaurosis fugax (ICD-8 3791)	253	759	Clinic patients	
stroke	358652		Carrieri	1994		Italian male patients in the Department of Neurology	Italy	Case-control	Male		40	75	Admission interview	Hospitalization	Medical diagnosis, including CT scan				First incidence of ischemic stroke	164	164	Hospitalized patients	
tb	298340		Buskin	1994		Population who would visit a tuberculosis clinic in King County, Washington	United States	Case-control	Both		18	99	Self-administered questionnaire	Incidence	Physician diagnosis				Tuberculosis	151	545	King County, Washington residents older than 17 years of age seeking care at the same tuberculosis clinic as the cases	
alzheimer_other_dementia	358836		Prince	1994		Adults recruited from the UK Medical Research Council elderly hypertension treatment trial	United Kingdom	Case-control	Both		72	87	Self-administered questionnaire	Incidence	Clinical records				Dementia	50	223	Clinic based	
parkinson	359164		Morano	1994		Mixed urban and rural population in Caceres province of Spain	Spain	Case-control	Male		18	99	Administered questionnaire	Incidence	Medical records				Parkinson's disease	33	66	Subjects presenting to the emergency room because of minor, non-	

																						neurological ailments or to neurology clinics with functional CNS pathology	
esophageal_cancer	309841		Gao	1994		Permanent residents of urban Shanghai	China	Case-control	Both		30	74	Structured, standardized questionnaire	Incidence	Disease registry				Esophageal cancer	556	799	Population	
stomach_cancer	340578		Guo	1994		General population without disabilities or cancer residing in four of Linxian county's four communes	China	Nested case-control	Male		40	69	Interview	Incidence	Physician diagnosis, medical records				Stomach cancer (cardia and non-cardia)	539	2695	Randomly-selected from the remaining study cohort who did not develop cancer	
pancreatic_cancer	347176		Silverman	1994		Residents from Atlanta, GA, Detroit, MI, and New Jersey	United States (Goergia, Michigan, New Jersey)	Case-control	Both		30	79	Personal interview	Incidence	Medical records				Carcinoma of the pancreas (ICD 157)	526	2153	General population	
fractures	414227		Mallmin	1994		Residents of Uppsala	Sweden	Case-control	Female		40	80	Mailed questionnaire	Incidence	Radiology reports and casualty reports				Distal forearm fracture	367	367	Population	
fractures	414229		Cumming	1994		Elderly residents of Sydney	Australia	Case-control	Both		65	100	Administered questionnaire	Incidence	Medical reports				Hip fracture	209	207	Population	
cvd	282925		Bolinder	1994	Swedish Construction Industry's Organization for Working Environment Safety	Construction industry employees	Sweden	Prospective cohort	Male	12	35	65	Administered questionnaire	Mortality	Vital records		1285	19426	Ischemic heart disease; Stroke				
cvd	335809		Kawachi	1994	Nurses' Health Study	Registered female nurses	United States	Prospective cohort	Female	12	30	55	Questionnaire	Incidence or Mortality	Self-reported, medical	1370000	970	121700	Ischemic heart disease				

															records, vital records								
parkinson	173863		Doll	1994	British Doctors' Study	Male doctors who resided in the United Kingdom	United Kingdom	Prospective cohort	Male	40	18	99	Mailed questionnaire	Mortality	Administrative records		152	10812	Parkinsonism				
parkinson	359164		Morano	1994		Mixed urban and rural population in Caceres province of Spain	Spain	Case-control	Male		18	99	Administered questionnaire	Incidence	Medical records				Parkinson's disease	33	66	Subjects presenting to the emergency room because of minor, non-neurological ailments or to neurology clinics with functional CNS pathology	
bladder_cancer	502458		Siemiatycki	1994		Resident in the Montreal metrop area	Montreal, Canada	Case-control	Male		35	70	Interview or questionnaire	Incidence	Biomarker				Bladder cancer	484	2238	Cancer patients, individuals from electoral lists in the Montreal area and random digit dialing	
kidney_cancer	502441		Mellemgaard	1994		All histologically verified cases of renal-cell cancer	Denmark	Case-control	Both		20	79	Structured questionnaire	Incidence	Cancer registry				Kidney cancer	368	396	From the Central Popuatlion Register	
fractures	261365		Forsen	1994		All residents of Nørd-Trondelag invited to a health screening	Nord-Trøndelag, Norway	Prospective cohort	Both	2.8	50	99	Questionnaire	Incidence	Hospital records	108907	524	38356	Hip fracture				

fractures	315458		Lobo	1994		Sample drawn from Zarazoga Dementia and Depression Project	Spain	Prospective cohort	Both	16	55	99	Interview	Incidence	Disease registry	76,848	275	4803	Hip fracture				
fractures	261387		Hemenwa y	1994	Health Professional s Follow-up Study	Male health professional s 40-75 years of age in 1986	United States	Prospective cohort	Male	5.4	40	75	Questionnaire	Incidence	Self-reported	271484	67	49895	Hip fracture				
lung_can cer	502458		Siemiatyck i	1994		Patients admitted to the hospital with lung cancer at the Graduate Institute of Medical Education and Research (PGIMER), Chandigarh. "	Quebec, Canada	case-control	Male				administered interview	Incidence	Hospital records				lung cancer	146	146	Varied	
ihd	356109		Watt	1995	Renfrew/Pai sley (MIDSPAN) Study	Residents of Paisley and Renfrew, Scotland	United Kingdom	Prospective cohort	Both	15	45	64	Unspecified	Mortality	Vital records		1567	15411	Coronary heart disease (ICD 410-414)				
asthma	346743		Troisi	1995		Female registered nurses	United States	Prospective cohort	Femal e	10	30	55	Self-administered questionnaire	Incidence	Physician diagnosis	692423	671	74072	Asthma				
diabetes	348079		Rimm	1995	Health Professional s Follow-up Cohort	Male health professional s in the US	United States	Prospective cohort	Male	6	40	75	Self-administered questionnaire	Incidence	Self-report, medical records	230769	509	41810	Non-insulin-dependent diabetes mellitus				
liver_can cer	343580		Siemiatyck i	1995		Males residing in the Montreal metropolitan area	Canada	Case-control	Male		35	70	Personal interviews	Incidence	Histologic ally confirmed cases				Liver cancer	48	2238	General populatio n and cancer controls	
liver_can cer	343632		Tanaka	1995		Cases and controls pooled from three studies in the Fukouka,	Japan	Pooled case-control	Femal e		35	79	Personal interviews	Incidence	Physician diagnosed				Hepatocell ular carcinoma	120	257	Hospital based	

						Saga, and Osaka prefectures																	
nasopharyngeal_cancer	346040		Zhu	1995		Men in areas covered by eight cancer registries in the US	United States	Case-control	Male		15	39	Personal interview	Incidence	Disease registry				Nasopharyngeal cancer	113	1910	General population	
lip_oral_cavity_cancer	348052		Macfarlane	1995		Residents of Beijing city, western New York state and Turin city	China, United States, Italy	Pooled case-control	Both		18	99	Subject interview	Incidence	Histologically-confirmed diagnosis				Oral cancer	835	1300	Hospital patients and general population	
pancreatic_cancer	346870		Ji	1995		Residents of the 10 urban districts of Shanghai	Shanghai, China	Case-control	Both		30	74	Personal interview	Incidence	Disease registry, histopathologically confirmed cases				Pancreatic cancer	451	1552	General population	
kidney_cancer	358809		Schlehofer	1995		Residents from the Rhein-Neckar-Odenwald area, cases recruited from 10 urology departments in the area	Germany	Case-control	Both		20	75	Personal interviews	Incidence	Histologically confirmed cases				Renal cell cancer (ICD 9: 189.0)	277	286	General population	
kidney_cancer	358812		Muscat	1995		Residents from centers across 4 US states	United States (Illinois, Michigan, New York, Pennsylvania)	Case-control	Both		18	99	Administered questionnaire	Incidence	Histologically confirmed cases				Renal cell carcinoma (ICD 9: 189.0)	788	779	Hospital patients	
cvd	463522		Wannamethee	1995	British Regional Heart Study	Registries of general practices in 24 towns in England, Wales, and Scotland	United Kingdom	Prospective cohort	Male	8	40	59	Administered questionnaire	Mortality	Doctor report, vital records	488		7735	Ischemic heart disease				
esophageal_cancer	343580		Siemiatycki	1995		Males in the Montreal metropolitan area	Montreal, Canada	Case-control	Male		35	70	Interview or questionnaire	Incidence	Biomarker				Esophageal cancer	99	2238	From electoral lists in the	

																						Montreal area	
stomach_cancer	343580		Siemiatycki	1995		Males in the Montreal metropolitan area	Montreal, Canada	Case-control	Male		35	70	Interview or questionnaire	Incidence	Biomarker				stomach cancer	251	2238	From electoral lists in the Montreal area	
liver_cancer	343580		Siemiatycki	1995		Males in the Montreal metropolitan area	Montreal, Canada	Case-control	Male		35	70	Interview or questionnaire	Incidence	Biomarker				Liver cancer	48	2238	From electoral lists in the Montreal area	
colon_and_rectum_cancer	343580		Siemiatycki	1995		Males in the Montreal metropolitan area	Montreal, Canada	Case-control	Male		35	70	Interview or questionnaire	Incidence	Biomarker				Colon cancer	505	1492	From electoral lists in the Montreal area	
bladder_cancer	343580		Siemiatycki	1995		Males in the Montreal metropolitan area	Montreal, Canada	Case-control	Male		35	70	Interview or questionnaire	Incidence	Biomarker				Bladder cancer	484	2238	From electoral lists in the Montreal area	
kidney_cancer	502478		McLaughlin	1995		Patients with renal-cell cancer	Australia, Denmark, Germany, Sweden, United States	Case-control	Both		20	79	Interview	Incidence	Cancer registry and hospitals/pathologists				Kidney cancer	707	992	From registers covering the entire population (Denmark), electoral rolls (Australia), residential lists (Germany), Health Care Financing Administration lists or random digit	

																						dialing (United States)	
kidney_cancer	343580		Siemiatycki	1995		Males in the Montreal metropolitan area	Montreal, Canada	Case-control	Male		35	70	Interview or questionnaire	Incidence	Biomarker				Kidney cancer	177	2238	From electoral lists in the Montreal area	
fractures	261418		Cummings	1995		Women who were able to walk and at least 65 years contacted through mailings lists	Portland, Oregon, Minneapolis, Baltimore, Monongahela Valley, Pennsylvania	Prospective cohort	Female	4.1	65	87	Questionnaire	Incidence	Self-report, Radiographs	39016	192	9516	Hip fracture				
fractures	498453		Johnell	1995		Men and women aged 50 years or over from 14 centers in six countries in Southern Europe	France, Greece, Italy, Portugal, Spain, Turkey	Case-control	Both		50	100	Standardized interviews	Incidence	Medical reports				Hip fracture	2086	3532	Population	
fractures	498446		Tavani	1995		Postmenopausal women	Italy	Case-control	Female		45	74	Standardized interviews	Incidence	Medical reports				Hip fracture	241	719	Hospital	
lung_cancer	343580		Siemiatycki	1995		"Department of Pulmonary Medicine of Post	Quebec, Canada	case-control	Male				administered interview	Incidence	Hospital records				lung cancer (ICD C33-C34)	370	370	Population	
pancreactic_cancer	343580		Seimiatycki	1995			Quebec, Canada	case-control	Male		35	70	self-report	Incidence of pancreatic cancer	Administrative medical records or disease registries				Pancreatic Cancer	176	352	Population	
ihd	328472		Yuan	1996		Male residents of Shanghai	China	Prospective cohort	Male	5.4	45	64	Structured interviews	Mortality	Vital records		68	18244	Ischemic heart disease (ICD-9 410-414)				
asthma	111344		Strachan	1996	British national child development study	All people in England, Scotland, or Wales born during one	United Kingdom	Prospective cohort	Both	33	17	33	Administered interview by proxy	Incidence	Self-reported		2051	18559	Asthma or wheezing				

						week, 3-9 March 1958																	
pancreatic_cancer	346826		Fuchs	1996	Health Professionals Follow-up Study (HPFS)	American female registered nurses and male health professionals	United States	Prospective cohort	Both	12.6	30	75	Self-administered questionnaire	Incidence	Self-report, vital records, medical records	2116229	186	167767	Pancreatic cancer				
macular_degeneration	261355		Christen	1996	Physicians' Health Study	US male physicians	United States	Prospective cohort	Male	12.2	40	84	Baseline questionnaire	Incidence	Medical record review	258115	268	21157	Age-related macular degeneration				
macular_degeneration	359130		Seddon	1996	Nurses' Health Study	Female registered nurses from 11 states	United States	Prospective cohort	Female	12	50	59	Baseline questionnaire	Incidence	Self-report and medical record review	556338	215	31843	Age-related macular degeneration with vision loss				
lung_cancer	357400		De Stefani	1996		Cases and controls admitted to the Instituto National de Oncologia of Montevideo	Uruguay	Case-control	Male		25	84	Administered interview	Incidence	Clinical records				Lung cancer	497	994	Hospital	
lung_cancer	357402		Lei	1996		Adults in Guangzhou	China	Case-control	Male		18	99	Administered interviews by proxy	Mortality	Vital records				Lung cancer	792	1376	Population	
lung_cancer	358338		Cascorbi	1996		German patients select hospitals	Germany	Case-control	Both		17	84	Self-reported	Incidence	Clinical records				Lung cancer	389	1046	Hospital	
lung_cancer	419669		De Stefani	1996		Hospital-based cases and controls from 7 major hospitals in Montevideo, Uruguay	Uruguay	Case-control	Both		30	89	Administered interviews and questionnaires	Incidence	Clinical, radiological, and endoscopic diagnoses				Lung cancer	320	640	Hospital	
copd	356344		Perez-Padilla	1996		Economically deprived population of Mexico with no social	Mexico	Case-control	Female		40	99	Interview	Incidence	Biomarker				Chronic airway obstruction (CAO), defined as	127	375	Controls were selected from among four	

						security health plans													FEV1 < 75%			groups at the hospital: patients with pulmonary tuberculosis, patients with interstitial lung disease, patiens with ear, nose, and throat ailments, and healthy visitors	
ihd	357441		Pais	1996		Adults of South Asian descent	India	Case-control	Both		30	60	Standardized interviews	Hospitalization	ECG results and Clinical diagnosis				Acute myocardial infarction	200	200	Hospital	
liver_cancer	293871		Shin	1996		Patients admitted to the Inje University Pusan Paik Hospital	Republic of Korea	Case-control	Both		18	99	Personal interviews	Incidence	Histologically confirmed cases, angiography, liver scanning, tomography, and magnetic resonance imaging				Hepatocellular carcinoma	203	406	Hospital based	
breast_cancer	309776		Morabia	1996		General population of Geneva	Switzerland	Case-control	Female		18	74	Interview	Incidence	Cancer Register of Canton Geneva				First diagnosis of invasive breast cancer	244	1032	Female resident of Geneva without previous breast cancer aged 30-74 years	
nasopharyngeal_cancer	346032		Vaughan	1996		Cases from disease registries in	United States	Case-control	Both		18	74	Personal interview	Incidence	Disease registry				Nasopharyngeal cancer	231	244	General population	

						Washington, Michigan, Connecticut, Iowa, and Utah and randomly selected controls													(ICD-O 147)				
pancreatic_cancer	359139		Lee	1996		Inpatients at the Veterans General Hospital-Taipei	Taiwan	Case-control	Both		18	99	Medical record review	Incidence	Clinical records, histological confirmed cases				Pancreatic cancer	282	282	Hospital based	
cataracts	359121		Phillips	1996		Patients at the Princess Alexandra Eye Pavillion, Edinburgh, UK	Scotland, United Kingdom	Case-control	Both		10	99	Administered questionnaire	Incidence	Physician diagnosis				Cataract	996	996	Hospital based	
peptic_ulcer	349377		Wang	1996		Factory workers in Shanghai	China	Case-control	Both		18	99	Interview	Incidence	Confirmed by endoscopy or gastrointestinal barium examination				New or recurrent peptic ulcer within the past two years	500	500	Randomly chosen among factory workers without peptic ulcer	
cvd	438147		Iribarren	1996	Kaiser Permanente Medical Care Program	Enrollees in a health plan in the San Francisco-Oakland area	United States	Prospective cohort	Both	16	40	89	Health examination	Hospitalization or Mortality	Hospital discharge records, vital records		386	61756	Intracerebral hemorrhage				
cvd	464569		Njølstad	1996	Finnmark Study	Residents of Finnmark county	Norway	Prospective cohort	Both	14	20	49	Questionnaire	Incidence or Mortality	Hospital discharge records, medical records, vital recrods	187336	241	13266	Stroke; Subarachnoid hemorrhage; Intracerebral hemorrhage; Ischemic stroke				
cvd	464576		Haheim	1996	Oslo Study	Men living in Oslo	Norway	Prospective cohort	Male	18	40	49	Screening questionnaire	Mortality	Vital records	273940	85	16173	Stroke				

cvd	464113		Whisnant	1996		Residents of Rochester, MN	United States	Nested case-control	Both	50	90		Medical records	Incidence	Disease registry, medical records				Ischemic stroke	1444	1444	Population	
cvd	465004		Ogren	1996		Elderly males born in 1914 residing in Malmo	Sweden	Cross-sectional	Male	68	68		Questionnaire	Prevalence	Biomarker		55	388	Peripheral artery disease				129
fractures	261433		Torgerson	1996		Women randomly selected from the community health index	Aberdeen, Scotland	Prospective cohort	Female	2	45	49	Self-administered questionnaire	Incidence	Self-report	3714	44	1857	Fracture				
lung_cancer	358717		Yong	1997	NHANES I Epidemiologic Followup Study	Sample of the civilian noninstitutionalized population of the United States	United States	Prospective cohort	Both	22	25	74	Administered interviews and questionnaires	Incidence	Hospital records and death certificates	191292	248	10068	Lung cancer (ICD-9 162)				
prostate_cancer	165618		Veierod	1997		Men screened in three counties	Norway	Prospective cohort	Male	12.4	16	56	Questionnaire	Incidence	Disease registry, vital records	319588	72	25708	Prostate cancer				
prostate_cancer	347435		Rodriguez	1997	Cancer Prevention Study-II	Residents with at least one household member over 45 years old	United States	Prospective cohort	Male	9	30		Self-administered questionnaire	Mortality	Vital records	3928139	1748	450279	Prostate cancer				
ihd	328274		Chen	1997		Factory workers in Shanghai	China	Prospective cohort	Male	16	35	64	Standardized interview	Mortality	Factory records, vital records, hospital records, interviews	101949	69	9351	Coronary heart disease (ICD-9 410-414)				
diabetes	309588		Kawakami	1997		Male employees of a large electrical company in Japan	Japan	Prospective cohort	Male	8	18	53	Self-administered questionnaire	Incidence	Physician diagnosis	18375	41	2312	Non-insulin-dependent diabetes mellitus				

pancreatic_cancer	346828		Harnack	1997	Iowa Women's Health Study (IWHS)	Postmenopausal women in Iowa	Iowa, United States	Prospective cohort	Female	9	55	69	Self-administered questionnaire	Incidence & Mortality	Vital records, disease registry	291598	66	33976	Pancreatic cancer				
cataracts	261527		Hiller	1997	Framingham Eye Study	Surviving members of the Framingham Eye Study I and II	United States	Prospective cohort	Both	12.5	52	80	Personal examination	Incidence	Physician diagnosis		251	660	Nuclear, Cortical and Posterior-subcapsular lens opacity				
peptic_ulcer	349381		Aldoori	1997	Health Professionals Follow-up Study (HPFS)	Male health professionals who resided in the fifty US states	United States	Prospective cohort	Male	5.5	40	75	Baseline survey questionnaire	Incidence	Self-administered follow-up questionnaire, medical records	261165	138	47806	Duodenal ulcer				
prostate_cancer	165518		Cerhan	1997	Iowa 65+ Rural Health Study	Noninstitutionalized residents age 65 years old or older of Iowa and Washington counties in Iowa	United States	Prospective cohort	Male	8.1	65	101	Interview	Incidence	State Health Agency of Iowa's cancer database	8474	71	1050	Prostate cancer				
lung_cancer	298142		Dosemeci	1997		Non-civil servant employees	Turkey	Case-control	Male		18	99	Administrative medical records	Incidence	Administrative medical records				Lung cancer	1210	2039	Clinic	
lung_cancer	357367		Jockel	1997		Patients and population controls from Bremen and Frankfurt/Main	Germany	Case-control	Both		33	80	Administered interview	Incidence	Clinical records of histologically or cytologically confirmed cancerous legions				Lung cancer	1004	2008	Population	
lung_cancer	357404		Pawlega	1997		Male lung cancer patients and adults in the electoral roll	Poland	Case-control	Male		30	99	Self-administered questionnaire	Incidence	Disease registry of histologically confirmed cases				Lung cancer	176	517	Population	

lung_cancer	357437		Barbone	1997		Adults in the province of Trieste	Italy	Case-control	Male		36	98	Structured interview	Mortality	Autopsy reports				Lung cancer	755	755	Population	
lung_cancer	419671		Hu	1997		hospital-based cases and controls from Heilongjiang Province in China	Heilongjiang, China	Case-control	Both		18	99	Administered interviews and questionnaires	Incidence	Histological confirmed cases				Lung cancer	227	454	Hospital	
laryngeal_cancer	298142		Dosemeci	1997		Employees in the Marmara region, except for civil servants	Turkey	Case-control	Male		18	99	Standardized admission form	Incidence	Patient records, Histologic confirmation				Laryngeal cancer	832	829	Cancer patients unrelated to smoking or alcohol	
fractures	414223		Mortiz	1997		White and Black women in New York City or Philadelphia	United States	Case-control	Female		45	103	Standardized interviews	Incidence	Radiology reports				Hip fracture	425	766	Hospital and Community	
cvd	429870		Ross	1997		Male residents from Shanghai	China	Nested case-control	Male	45	64		Interview	Mortality	Vital records				Stroke	245	1225	Cohort population	
cvd	464302		Kubota	1997		Patients from 48 hospitals around Japan	Japan	Case-control	Both	31	76		Self-administered questionnaire	Incidence	Clinical records				Subarachnoid hemorrhage; Intracerebral hemorrhage; Ischemic stroke	502	502	Hospital	
cvd	464726		You	1997		Patients from Austin, Royal Melbourne, Box Hill, Preston, and Northcote community hospitals in Melbourne	Australia	Case-control	Both	15	60		Interview	Incidence	Clinical diagnosis				Ischemic stroke	201	201	Population	
cvd	464839		Lee	1997		Subjects recruited from the	United Kingdom (Scotland)	Nested case-control	Both	55	74		Self-administered-questionnaire	Incidence	Vital records,				Aortic aneurysm	40	200	Population	

						Edinburgh Artery Study									medical records								
lip_oral_cavity_cancer	343432		Zheng T	1997		cases presenting between 1988 to 1989 to a hospital in the Beijing area	China	case-control	Both		20	80	self-report	histologically confirmed tongue cancer	Administrative medical records or disease registries				Lip and Oral Cavity Cancer	159	348	population	
bladder_cancer	502393		Yu	1997		Patients who received surgery for tumour removal and patients with non-urinary system and non-neoplastic diseases	Heilongjang, China	Case-control	Both		20	99	Interview	Incidence	Biomarker				Bladder cancer	127	254	Patients with non-urinary system and non-neoplastic diseases	
fractures	498367		Grisso	1997		Men aged 45 years and older with a radiologically confirmed diagnosis of a first hip fracture	United States	Case-control	Male		45	99	Administered questionnaire	Incidence	Medical reports				Hip fracture	356	402	Men randomly selected from lists of enrollees in the Kaiser Permanente Medical Care program of Northern California.	
lung_cancer	328266		Liaw	1998	12-Year Follow-Up Study	Adult residents of study townships and precincts	Taiwan	Prospective cohort	Both	12	41	99	Administered interview	Mortality	Vital records	140493	127	14397	Trachea, bronchus, and lung cancer				
stroke	344345		Prescott	1998	Copenhagen City Heart Study; Glostrup Population Studies;	Adults in the Copenhagen area	Denmark	Pooled prospective cohort	Both	30	20	99	Self-adminitered questionnaire	Mortality	Vital records	453000	548	30809	Cerebrovascular disease (ICD 430-438)				

					Copenhagen Male Study																		
multiple_sclerosis	350715		Thorogood	1998	Royal College of General Practitioners' Oral Contraception Study	Married women	United Kingdom	Prospective cohort	Female		18	99	Standardized questionnaire	Incident episode	Clinical diagnosis	563907	114		Multiple sclerosis				
esophageal_cancer	328266		Liaw	1998		Residents of 12 townships and precincts	Taiwan (Province of)	Prospective cohort	Both	9.8	41	99	Standardized interview	Mortality	Vital records, medical records	140493	27	14397	Esophageal cancer				
liver_cancer	328266		Liaw	1998		Residents in 12 townships and precincts in Taiwan	Taiwan	Prospectve cohort	Both	9.8	40	99	Personal interview	Mortality	Vital records	140493	128	14397	Liver cancer (ICD-9)				
nasopharyngeal_cancer	328266		Liaw	1998		Residents in 12 townships and precincts in Taiwan	Taiwan	Prospective cohort	Both	9.8	41	99	Personal interview	Mortality	Vital records	140493	18	14397	Nasopharynx cancer (ICD-9)				
gallbladder_diseases	350725		Sahi	1998	Harvard Alumni Health Study	Males who matriculated as undergraduates at Harvard University	United States	Prospective cohort	Male	61	32	74	Interview	Incidence	Self-reported physician diagnosis and validated by contacting the physician		685	16785	Gallbladder disease				
fractures	261399		Jacqmin-Gadda	1998	The Paquid study	Elderly folks living at home at baseline in 75 civil parishes	France	Prospective cohort	Both	5	65	99	Structured interviews	Incidence	Self-reported, physician confirmed	12886	306	3216	Fracture				
lung_cancer	357406		Wunsch-Filho	1998		Hospital based cases and controls in Sao Paolo	Brazil	Case-control	Both		33	90	Administered questionnaire	Incidence	Clinical records of histologically or cytologically confirmed				Lung cancer	398	1258	Hospital	

															cancerous legions								
lung_cancer	357447		Matos	1998		Patients and residents in the Province of Buenos Aires	Argentina	Case-control	Male		30	99	Administered interviews	Incidence	Clinical records				Lung cancer	200	597	Hospital	
lung_cancer	357451		De Stefani	1998		Hospital-based cases and controls in Montevideo that were residents of Uruguay for more than 10 years	Uruguay	Case-control	Male		30	89	Administered interviews	Incidence	Clinical records				Adenocarcinoma of the lung	426	845	Hospital	
lung_cancer	419675		Kreuzer	1998		Cases and population controls from several regions in East and West Germany	Germany	Case-control	Both		18	69	Administered questionnaire	Incidence	Histologically or cytologically confirmed cases				Lung cancer	2260	4579	Population	
tb	236195		Liu	1998		Population living in urban and rural areas across China	China	Case-control	Male		35	69	Proxy informants	Mortality	Administrative records, medical recoreds, proxy informants				Respiratory tuberculosis	13419	38875	Deceased who subjects who did not die due to a neoplastic, respiratory, or vascular disease	
stomach_cancer	340513		De Stefani	1998		Admitted patients of four hospitals in Montevideo	Uruguay	Case-control	Male		25	84	Interview	Incidence	Microscopically-confirmed diagnosis				Adenocarcinoma of the stomach	331	622	Admitted patients from the same hospitals as the cases who were dianosed with a disease not related to	

																						tobacco smoking and/or alcohol, diagnosed with a disease not related to the digestive tract, and be in the same age range as the cases	
breast_cancer	310463		Gammon	1998		General population of five counties in central New Jersey, three-county area surrounding Seattle, Washington, and the metropolitan area of Atlanta, Georgia	United States	Case-control	Female		18	54	Interview	Incidence	Physician diagnosis				In situ and invasive breast cancer	1497	1645	Selected by random digit dialing and matched with cases by age group and geographic area	
other_pharynx_cancer	343450		De Stefani	1998		Residents of Montevideo, Uruguay	Uruguay	Case-control	Male		25	84	Subject interview	Incidence	Microscopically-confirmed diagnosis				Paryngeal cancer (oropharynx, hypopharynx, pharynx unspecified)	219	427	Patients from the same hospitals who were diagnosed with diseases unrelated to tobacco smoking and/or alcohol intake, who have no non-neoplasti	

																						c lesions of the oral cavity and pharynx, who are in the same age range as the cases	
cvd	464556		Prescott	1998	Copenhagen City Heart Study; Glostrup Population Studies	Residents of central Copenhagen, Copenhagen suburbs, and 14 large workspaces in Copenhagen	Denmark	Pooled prospective cohort	Both	12.3	20	93	Self-administered questionnaire	Hospitalization or Mortality	Hospital discharge records, national health database		1763	24663	Ischemic heart disease				
cvd	464945		Adlerberth	1998	Multifactorial Primary Prevention Trial	Male residents of Goteborg born between 1915 and 1925, except those born in 1923	Sweden	Prospective cohort	Male	16	51	59	Postal questionnaire	Mortality	Vital records		1725	7100	Ischemic heart disease				
cvd	464095		Feigin	1998		Residents of the district of Novosibirsk Russian population	Russia	Case-control	Both	50	90		Interview	Incidence	Disease registry, vital records, clinical records				Ischemic stroke	237	237	Population	
cvd	465267		Hooi	1998		General population of the province of Limburg	The Netherlands	Cross-sectional	Both	40	78		Self-administered questionnaire	Prevalence	Biomarker		458	3650	Peripheral artery disease				
lip_oral_cavity_cancer	502352		Su, WZ	1998		patients diagnosed with squamous cell carcinoma between March, 1989 and September,	China	case-control	Male		27	82	self-report	incidence of oral cavity cancer	Physician diagnosis				Lip and Oral Cavity Cancer	101	101	population	

						1995 Affiliated Stomatological Hospital of China Medical University																	
bladder_cancer	502429		Taylor	1998		Hospital patients	United States	Case-control	Both		25	94	Structured interview	Incidence	Biomarker				Bladder cancer	230	203	Hospital patients	
fractures	261411		Mussolino	1998	National Health and Nutrition Examination Survey (NHANES) III	National probability sample of the civilian noninstitutionalized population	United States	Prospective cohort	Both	13.9	45	74	Questionnaire	Incidence or Mortality	Hospital records and death certificates	39914	61	2808	Hip fracture				
fractures	261426		Elmståhl	1998	Malmo¨Diet and Cancer study	6576 men, aged 46–68 years, living in the city of Malmo¨, in the southern part of Sweden	Malmo, Sweden	Prospective cohort	Male	2.4	46	68	Questionnaire	Incidence	Hospital records	15782	160	6576	Fracture				
fractures	498427		Clark	1998		Men and women from two major hospitals in Mexico City	Mexico	Case-control	Both		45	100	Administered questionnaire	Incidence	Radiology reports				Hip fracture	152	143	Healthy controls in hospital waiting room	
lung_cancer	334460		Nordlund	1999	Swedish prospective cohort	Adults in the Swedish population register	Sweden	Prospective cohort	Both	26	18	69	Administered interviews and questionnaires	Incidence	Disease registry and vital records		345	41710	Lung cancer				
ihd	165539		Jacobs	1999	The Seven Countries Study	Men in 16 cohorts in 7 countries	United States; Finland; Netherlands; Italy; Croatia; Serbia; Greece; Japan	Prospective cohort	Male	25	40	59	Standardized questionnaire	Mortality	Medical records, vital records, proxy interviews		1827	12763	Coronary heart disease				
stroke	334731		Hart	1999	Renfrew/Paisley Study	Residents of Renfrew and Paisley in Scotland	United Kingdom	Prospective cohort	Both	20	45	64	Self-administered questionnaire	Mortality	Vital records		689	15406	Stroke				

stroke	165539		Jacobs	1999	The Seven Countries Study	Men in 16 cohorts in 7 countries	United States; Finland; Netherlands; Italy; Croatia; Serbia; Greece; Japan	Prospective cohort	Male	25	40	59	Standardized questionnaire	Mortality	Medical records, vital records, proxy interviews		797	12763	Stroke				
diabetes	348081		Uchimoto	1999	Osaka Health Survey	Male employees of a company in Osaka, Japan	Osaka, Japan	Prospective cohort	Male	16	35	60	Self-administered questionnaire	Incidence	Physician diagnosis	60904	450	6250	Type 2 diabetes mellitus				
lbp	348204		Feldman	1999		High school students in grades 7 to 9 in Montreal, Quebec	Canada	Prospective cohort	Both	1	13	15	Self-administered questionnaire	Incidence	Self-administered questionnaire		65	377	Low back pain occurring at a frequency of at least once a week within the past 6 months				
prostate_cancer	347458		Giovannucci	1999	Health Professionals Follow-up Study	United States male dentists, optometrists, osteopaths, podiatrists, parmacists, and veterinarians	United States	Prospective cohort	Male	7.4	40	75	Self-administered questionnaire	Incidence & Mortality	Self-report, proxy report, medical reports	351261	1369	47781	Non-stage A1 cases of prostate cancer				
fractures	261429		Kato	1999	New York University Women's Health Study	Post-menopausal women that received a mammographic screening in New York City or Florida between 1985 and 1991	United States	Prospective cohort	Female	8.6	34	65	Self-administered questionnaire	Incidence	Self-reported		1025	6250	Fracture				

lung_cancer	357792		Band	1999		Male cancer patients	Canada	Nested case-control	Male		20	99	Self-administered questionnaire	Incidence	Disease registry				Lung cancer (ICD-9 162)	2998	7265	Disease registry	
lung_cancer	419696		Armadans	1999		Hospital-based cases and controls from a public teaching hospital in Barcelona, Spain	Spain	Case-control	Male		30	79	Administered interviews and questionnaires	Incidence	Histologically or cytologically confirmed cases				Lung cancer	325	650	Hospital	
prostate_cancer	347464		Parker	1999		Iowa residents	United States	Prospective cohort	Male	9	40	86	Mailed questionnaire or telephone interview	Incidence	Disease registry		81	1177	Prostate cancer				
ihd	357274		Dunn	1999		Participants of the MICA study	United Kingdom	Case-control	Female		16	44	Interviews	Hospitalization or Mortality	Hospital records, vital records				Incident myocardial infarction	448	1728	Community	
ihd	357952		Rosenberg	1999		Subscribers of Essence magazine	United States	Case-control	Female		21	69	Self-administered questionnaire	Incidence	Self-reported, medical records				Coronary heart disease	352	1760	Population	
ihd	358066		Bosetti	1999		Patients in Italian coronary care unti network	Italy	Pooled case-control	Both		18	74	Administered questionnaire	Hospitalization	Clinical diagnosis				First non-fatal acute myocardial infarction	1230	1839	Hospital	
stroke	357417		Bonita	1999		Residents of the Auckland region	New Zealand	Case-control	Both		35	74	Administered questionnaire	Incidence	Disease registry				First acute stroke	521	1851	Population survey	
stroke	358462		Thrift	1999		Residents and patients of the Melbourn metropolitan area	Australia	Case-control	Both		18	80	Structured administered questionnaire	Incidence	Vital records, Medical records				First intracerebral hemorrhage	331	331	Population	
lri	355112		Almirall	1999		Residents of Maresme county in Barcelona	Spain	Case-control	Both		15	75	Administered questionnaire	Incidence	Radiological diagnosis				Pneumonia	205	475	General population	
aortic_aneurism	350128		Wilmink	1999		Men over the age of 50 in Huntingdon, UK	England, United Kingdom	Nested case-control	Male		50	89	Self-administered questionnaire	Incidence	Physician diagnosis				Abdominal aortic aneurysm	210	237	General population	

laryngeal_cancer	343375		Schlecht	1999		Residents of three metropolitan centres of southern Brazil	Brazil	Case-control	Both		15	84	Blinded histological questionnaire	Incidence	Histological confirmation				Laryngeal cancer	194	361	Hospital-based	
cervical_cancer	347864		Ylitalo	1999		Female residents of Uppsala county	Sweden	Nested case-control	Female		25	49	Structured phone questionnaire	Incidence	Disease registry				Cervial carcinoma in situ	422	422	Cytology register	
nasopharyngeal_cancer	346047		Cheng	1999		Residents recruited from hospitals and the community in Taipei	Taiwan	Case-control	Both		15	74	Self-administered questionnaire	Incidence	Histologically confirmed cases				Nasopharyngeal carcinoma	375	327	General population	
cvd	165539		Jacobs	1999	The Seven Countries Study	Men in 16 cohorts in 7 countries	United States; Finland; Netherlands; Italy; Croatia; Serbia; Greece; Japan	Prospective cohort	Male	25	40	59	Standardized questionnaire	Mortality	Medical records, vital records, proxy interviews		2624	12763	Coronary heart disease, Stroke				
cvd	358462		Thrift	1999		Residents and patients of the Melbourn metropolitan area	Australia	Case-control	Both	18	80		Structured administered questionnaire	Incidence	Vital records, Medical records				First intracerebral hemorrhage	331	331	Population	
cvd	294121		Huhtasaari	1999		Male residents in the two northern-most counties in Sweden	Sweden	Case-control	Male	25	64		Interview	Incidence	Discharge records, physician diagnosis, vital records				Ischemic heart disease	687	687	Population	
cvd	389100		You	1999		Patients from 3 hospitals in Victoria, Australia	Australia	Case-control	Both	30	88		Interview	Incidence	Medical diagnosis				Ischemic stroke	452	452	Population	
cvd	463869		Connelly	1999		Residents of New York	United States	Case-control	Both	25	99		Interview	Incidence	Clinical diagnosis				Subarachnoid hemorrhage	342	1188	Population	

bladder_cancer	502413		Fortuny	1999		Hospital patients		Case-control	Both		NA	NA	Questionnaire	Incidence	Physician diagnosis				Bladder cancer	146	3366	Individuals from the same study center (general population and hospitals)	
fractures	261498		Cornuz	1999	Nurse's Health Study	Female registered nurses 30-55 years of age in 1976	United States	Prospective cohort	Female	12	30	55	Questionnaire	Incidence	Self-report	1,320,000	377	121,701	Hip fracture				
fractures	261381		Burger	1999	Rotterdam Study	All 10,275 inhabitants of Ommord, a district in Rotterdam, the Netherlands, were invited to participate	Ommoord, Rotterdam, Netherlands	Prospective cohort	Both	3.8	55	99	Interview	Incidence	Medical records	19790	50	5208	Hip fracture				
fractures	498392		Michaëlsson	1999		a large population-based case-control study in Swedish women	Stockholm, Uppsala, Va ̈stmanland, O ̈rebro, Go ̈teborg and Malmo ̈hus, Sweden	case-control	Female		51	80	Questionnaire	Incidence	Medical reports				Hip fracture, Cervical fracture, trochanteric fracture	1610	4059	Controls were native-born women randomly selected from the national, continuously updated population register in the counties included	
fractures	498396		Melhus	1999		Women who were residents in Sweden	Sweden	Case-control	Female		40	76	Questionnaire	Incidence	Self-report				Hip fracture	247	783	Population	
lri	298305		Baik	2000		Male health professionals and female registered nurses	United States	Prospective cohort	Both	8	27	79	Administered questionnaire	Incidence	Medical records, vital records		595	104491	Pneumonia				

diabetes	348083		Manson	2000	Physicians' Health Study	US male physicians	United States	Prospective cohort	Male	12	40	84	Self-administered questionnaire	Incidence	Self-report	255830	770	21068	Type 2 diabetes mellitus				
diabetes	348085		Nakanishi	2000		Japanese male office workers at T corporation in Osaka, Japan	Osaka, Japan	Prospective cohort	Male	5	35	59	Self-administered questionnaire	Incidence	Physician diagnosis	5937	54	1266	Type 2 diabetes				
prostate_cancer	165571		Putnam	2000		Iowa residents	United States	Prospective cohort	Male	9	40	86	Mailed questionnaire	Incidence	Disease registry	9509	101	1572	Prostate cancer				
pancreatic_cancer	346832		Nilson	2000		Residents in the Nord-Trondelag county in Norway	Norway	Prospective cohort	Both	9.8	30	99	Self-administered questionnaire	Incidence	Disease registry	622721	166	63374	Pancreatic cancer				
prostate_cancer	347448		Lotufo	2000	Physicians' Health Study	US male physicians with no history of myocardial infarcation, stroke, transient ischemic attack, or cancer (except non-melanoma skin)	United States	Prospective cohort	Male	12.5	40	84	Self-administered questionnaire	Incidence & Mortality	Self-report, proxy report, National Death Index	260926	996	21985	Prostate cancer				
prostate_cancer	359212		Lund	2000		All residents aged 20 years or more in the county of Nord-Trondelag	Norway	Prospective cohort	Male	9.3	49.2	96.2	Mailed questionnaire	Incidence	Cancer Registry of Norway	212720	644	22895	Prostate cancer				
fractures	132098		Huopio	2000		Female residents of Kuopio Province	Finland	Prospective cohort	Female	3.6	47	56	Questionnaire	Incidence	Self-reported, radiological reports		257	3068	Fracture (except for those due to motor vehicle accidents)				
lung_cancer	286844		Dikshit	2000		Males in Bhopal	India	Case-control	Male		18	99	Administered questionnaire	Incidence	Disease registry				Lung cancer	163	423	Population	
ihd	357413		Miyake	2000		Residents/patiens of Fukuoka City and	Japan	Case-control	Both		40	79	Administered questionnaire	Non-fatal hospitalization	Clinical diangosis by collaborati				Non-fatal acute myocardial infarction	384	656	Population	

						adjacent municipalities									ng cardiologists								
lri	355108		Nuorti	2000		Patients and residents from Atlanta, Baltimore, and Toronto	Canada, United States	Case-control	Both		18	64	Administered questionnaire	Incidence	Clinical records				Invasive pneumococcal disease	228	301	General population	
parkinson	359151		Benedetti	2000		General population of Omsted County, Minnesota	United States	Case-control	Both		41	97	Medical records	Incidence	Medical records				Parkinson's disease	196	196	General population of Olmsted County, Minnesota and free of Parkinson's disease, other parkinsonism, or tremor of any type in the index year	
esophageal_cancer	339730		Cheng	2000		Residents of study areas	United Kingdom	Case-control	Female		18	79	Interview	Incidence	Disease registry and histological confirmation				Esophageal adenocarcinoma	74	74	Population	
stomach_cancer	340528		Zaridze	2000		Cases were selected from the two main cancer-treatment hospitals in Moscoe and controls were selected from the two major general hospitals in Moscow	Russia	Case-control	Male		18	99	Self-administered questionnaire	Incidence	Physician diagnosis				Newly diagnosed, histologically-confirmed stomach cancer	248	292	Patients from the two major general hospitals in Moscow with the same catchment area as the two major cancer-treatment	

						with the same catchment area as the hospitals from which the cases were selected; cases and controls were restricted to residents of Moscow city																hospitals without cancer or gastrointestinal diseases	
nasopharyngeal_cancer	346049		Yuan	2000		Patients identified from the Shanghai Cancer Registry and controls randomly selected from the urban Shanghai population	Shanghai, China	Case-control	Both		15	74	Personal interview	Incidence	Histologically confirmed cases, disease registry				Nasopharyngeal carcinoma	935	1032	General population	
prostate_cancer	359218		Nomura	2000		Japanese-American men on the Hawaiian island of Oahu	United States	Nested case-control	Male		45	68	Interview	Incidence	Hospital discharge records, Hawaii Tumor Registry				Prostate cancer	249	249	Cohort members who were not cases	
cvd	463446		Thun	2000	Cancer Prevention Study II (CPS-II)	Nationwide residents	United States	Prospective cohort	Both	6	30		Postal questionnaire	Mortality	Direct inequiry, vital records		23738	974150	Ischemic heart disease; Other cardiovascular and circulatory diseases; Stroke				
cvd	463455		Takagi	2000		Residents of two rural communities of Hokkaido	Japan	Prospective cohort	Both	18	40	64	Administered interview	Mortality	Familial reporting, public health reporting, vital records,			1996	Cardiovascular diseases				

															hospital records								
cvd	464667		Kunze	2000		Patients from the Departments of Neurology and Neurosurgery of the University of Heidelberg	Germany	Case-control	Both	20	84		Standardized questionnaire	Incidence	Clinical diagnosis				Subarachnoid hemorrhage; Intracerebral hemorrhage	100	100	Hospital	
cvd	465270		Meijer	2000		General population residing in the suburb of Ommoord in Rotterdam	The Netherlands	Cross-sectional	Both	55	88		Self-report	Prevalence	Biomarker		1226	6450	Peripheral artery disease				1294
parkinson	359151		Benedetti	2000		General population of Omsted County, Minnesota	United States	Case-control	Both		41	97	Medical records	Incidence	Medical records				Parkinsonism	196	196	General population of Olmsted County, Minnesota and free of Parkinson's disease, other parkinsonism, or tremor of any type in the index year	
bladder_cancer	502401		Wakai	2000		Patients in seven hospitals	Aichi Prefecture, Central Japan	Case-control	Both		20	99	Structured interview	Incidence	Physician diagnosis, biomarker				Bladder cancer	300	300	Hospital patients with other diagnoses excluding cancer	
fractures	261375		Høidrup	2000	Copenhagen County Centre of Preventive	Individuals from central Copenhagen, suburbs of	Copenhagen, Denmark	Prospective cohort		19.5	20	93	Self-administered questionnaire	Incidence	Hospital records	598847	1169	30772	Hip fracture				

					Medicine, Copenhagen City Heart Study, Copenhagen Male Study	Copenhagen, and 14 major work sites in the Copenhagen area																	
ihd	328259		Nilsson	2001		Swedish adults	Sweden	Prospective cohort	Both	33	18	69	Self-administered questionnaire, telephone interview	Mortality	Vital records		7388	41544	Ischemic heart disease (ICD-8 410-414)				
stroke	344343		Wang	2001	Systolic Hypertension in China (Syst-China) Trial	Clinical trial patients	China	Prospective cohort	Both	3	60	99	Administered questionnaire	Incidence or Mortality	Screenings	6414	56	2284	Stroke				
stroke	328259		Nilsson	2001		Swedish adults	Sweden	Prospective cohort	Both	33	18	69	Self-administered questionnaire, telephone interview	Mortality	Vital records	877635	352	41544	All cerebrovascular disease				
diabetes	309592		Hu	2001	Nurses' Health Study	Female nurses in the US	United States	Prospective cohort	Female	16	34	59	Self-administered questionnaire	Incidence	Self-report	1301055	3300	84941	Type 2 diabetes				
diabetes	348088		Wannamethee	2001	British Regional Heart Study	Men recruited from one general practice in each of 24 towns in England, Wales, and Scotland	England, United Kingdom	Prospective cohort	Male	16.8	40	59	Administered questionnaire	Incidence	Physician diagnosis, medical records, vital records		290	7124	Diabetes				
diabetes	356075		Will	2001	CPS I	Residents from 25 states throughout the US	United States	Prospective cohort	Both	13	30	99	Self-administered questionnaire	Incidence	Self-report, vital records	6723960	25397	709827	Diabetes mellitus				
aortic_aneurism	328259		Nilsson	2001		Swedish residents	Sweden	Prospective cohort	Both	28	18	69	Self-administered questionnaire, personal interview	Mortality	Vital records	877635	196	41544	Aortic aneurysm (ICD-8, 441)				
peptic_ulcer	328259		Nilsson	2001		1960 Swedish census population	Sweden	Prospective cohort	Both	13.9	18	69	Survey questionnaire, telephone interview	Mortality	Cause of Death Registry	877635	170	41544	Peptic ulcer (ICD-8 531-534)				

lung_cancer	357415		Mao	2001		Adults in the provinces of British Columnia, Alberta, Saskatchewan, Manitoba, Ontario, Prince Edward Island, Nova Scotia, and Newfoundland	Canada	Case-control	Both		20	99	Mailed questionnaire and phone interviews	Incidence	Disease surveillance system of hystologically confirmed cases				Lung cancer	3280	8353	Population	
lung_cancer	357644		Simonato	2001		Hospital-based cases and controls and population-based controls in 6 countries	Sweden, Germany, United Kingdom, France, Spain, Italy	Pooled case-control	Both		15	99	Administered interviews	Incidence	Clinical records				Lung cancer	6035	14002	Varied	
lung_cancer	419728		Rachtan	2001		Hospital-based cases and controls at a hospital in Cracow, Poland	Poland	Case-control	Female		18	99	Administered interviews and questionnaires	Incidence	Histologically confirmed cases				Lung cancer (ICD-9 162)	242	594	Hospital contacts	
ihd	358054		Suh	2001		Patients in a university teaching hospital in Seoul	South Korea	Case-control	Male		30	75	Administered interview	Hospitalization	Clinically confirmed diagnosis				First acute myocardial infarction	108	142	Hospital	
stroke	343582		Lam	2001		Chinese residents of Hong Kong	Hong Kong	Case-control	Both		35	69	Administered interview or self-adminitered questionnaire	Mortality	Vital records				Stroke	721	6410	Population	
stroke	350747		Yamaura	2001		Patients from multiple health centers	Japan	Case-control	Both		18	99	Structured questionnaire	Incidence	Medical diagnosis, confirmed by cerebral angiography				Aneurysmal subarachnoid hemorrhage	127	127	Clinical outpatients	
tb	343582		Lam	2001		Ethnic Chinese in Hong Kong	China	Case-control	Both		35	99	Proxy informants	Mortality	Death registry				Respiratory tuberculosis	27507	13054	Living relatives of the	

																						deceased cases	
parkinson	359160		Paganini-Hill	2001		Residents who owned homes in the Leisure World Laguna Hills retirement community in California	United States	Nested case-control	Both		55	99	Mailed questionnaire	Incidence	Self-report, medical records, death certificates				Parkinson's disease	201	1210	Portion of cohort who were not cases	
multiple_sclerosis	369019		Ghadirian	2001		Residents of greater Montreal area	Canada	Case-control	Both		1	99	Administered questionnaire	Incidence	Diagnosis				Multiple sclerosis	200	202	Population	
esophageal_cancer	345324		Matsuo	2001		Patients at the Aichi Cancer Center	Japan	Case-control	Both		40	76	Self-administered	Incidence	Clinical diagnosis				Esphageal cancer	102	241	Outpatients	
other_pharynx_cancer	343434		Zavras	2001		Residents of metropolitan Athens and Attica	Greece	Case-control	Both		22	91	Subject interview	Incidence	Primary histopathologically-confirmed diagnosis				Oral and pharyngeal cancer (ICD-9 141, 143-145, 148-149)	110	115	Patients from the same hospitals who were hospitalized for conditions unrelated to cancer or who were trauma patients	
bladder_cancer	343155		Chiu	2001		Residents of Iowa	Iowa, USA	Case-control	Both		40	85	Mailed questionnaire and/or telephone interview	Incidence	Disease registry				Kidney cancer adenocarcinomas, excluding cancers of the renal pelvis	1406	2336	Population	
kidney_cancer	343155		Chiu	2001		Residents of Iowa	Iowa, United States	Case-control	Both		40	85	Self-administered questionnaire	Incidence	Disease registry				Kidney cancer	406	2434	General population	
kidney_cancer	358814		Semenza	2001		Residents from Orange	California, United States	Case-control	Both		20	74	Personal interviews	Incidence	Disease registry, histologica				Renal cell carcinoma	115	259	General population	

						County, California									lly confirmed cases				(ICD C64.9)				
fractures	414220		Baron	2001		Post-menopausal women in study areas	Sweden	Case-control	Female		50	81	Mailed questionnaire	Incidence	Hospital discharge records				Hip fracture	1327	3262	Population	
cvd	298324		Suh	2001	Korea Medical Insurance Corporation (KMIC) Study	Civil services workers, private school workers, and dependents	South Korea	Prospective cohort	Male	6	35	59	Self-administered questionnaire	Incidence or Mortality	Hospital discharge records, vital records		358	114793	Intracerebral hemorrhage; Subarachnoid hemorrhage				
cvd	350747		Yamaura	2001		Patients from multiple health centers	Japan	Case-control	Both	18	99		Structured questionnaire	Incidence	Medical diagnosis, confirmed by cerebral angiography				Aneurysmal subarachnoid hemorrhage	127	127	Clinical outpatients	
cvd	463965		Qureshi	2001		Patients recruited from the Neurosciences Critical Care Unit at John Hopkins Hospital	United States	Case-control	Both	25	80		Interview	Incidence	Medical records, disease registry				Subarachnoid hemorrhage	323	969	Population	
parkinson	359152		Hernan	2001	Nurses' Health Study and Health Professionals Follow-up Study	Male health professionals from all states and female registered nurses in eleven states	United States	Pooled prospective cohort	Both	20 (10)	30	75	Mailed questionnaire	Incidence	Self-report, medical records, physician questionnaire, vital records	2681638	299	173229	Definite and probable idiopathic Parkinson's disease				
parkinson	359160		Paganini-Hill	2001		Residents who owned homes in the Leisure World Laguna Hills retirement community in California	United States	Nested case-control	Both		55	99	Mailed questionnaire	Incidence	Self-report, medical records, death certificates				Parkinson's disease	201	1210	Portion of cohort who were not cases	

bladder_cancer	502403		Geoffroy-Perez	2001		Patients from seven hospitals	France	Case-control	Male		45	75	Structured interview	Incidence	Physician diagnosis				Bladder cancer	602	615	Hospital patients with other diagnoses excluding cancer	
fractures	498389		Lau	2001		Men and women who were 50 years and older and who were admitted with a diagnosis of hip fracture were recruited	Malaysia, Philippines, Singapore, Thailand	Case-control	Both		50	99	Administered questionnaire	Incidence	Self-report				Hip fracture	1176	1162	In Malaysia, subjects presenting in general practice clinics for minor ailments were used. In the Philippines, subjects seen by doctors in outreach health programs were used. In Thailand, neighborhood controls were used. In Singapore, community controls were recruited by household surveys.	
ihd	336682		Jonsdottir	2002	Reykjavik Study	Residents of Reykjavik	Iceland	Prospective cohort	Both	28	30	84	Administered questionnaire	Incidence or Mortality	Medical records, hospitaliza		2406	98569	Non-fatal myocardial infarction				

															tion records, vital records, autopsy reports				or coronary death (ICD-9 410-414.9)				
ihd	343310		Prescott	2002	Copenhagen City Heart Study	Residents of study area in Copenhagen	Denmark	Prospective cohort	Both	22	30	99	Self-administered questionnaire	Incidence or Mortality	Vital records, discharge records		1348	12149	Myocardial infarction (ICD-10 I21-I22)				
stroke	324092		Klatsky	2002		Members of a pre-paid healthcare system	United States	Prospective cohort	Both	18	18	99	Questionnaire	Hospitalization or Mortality	Medical records, vital records		431	128934	Hemorrhagic stroke				
stomach_cancer	340582		Chao	2002	Cancer Prevention Study (CPS) II	Individuals at least 30 years of age and living in a household with one person at least 45 years of age in all 50 states of the United States, District of Columbia, and Puerto Rico	United States, Puerto Rico	Prospective cohort	Both	13.2	30	99	Self-administered questionnaire	Mortality	Death certificates, National Death Index	13893594	1505	1055841	Stomach cancer (ICD-9 151.0-151.9)				
pancreatic_cancer	346876		Lin	2002	Japan Collaborative Cohort Study for Evaluation of Cancer Risk (JACC)	Residents from 45 areas throughout Japan	Japan	Prospective cohort	Male	8.1	40	79	Self-administered questionnaire	Mortality	Vital records	807407	225	110792	Pancreatic cancer (ICD-10, C25)				
cataracts	261496		Weintraub	2002		Female registered nurses and male health professionals from all 50 states	United States	Prospective cohort	Both	8.8	30	75	Self-administered questionnaire	Incidence	Medical records, physician diagnosis	1038493	4281	117731	Cataract extraction				
lbp	348202		Tubach	2002	GAZEL cohort	Workers from the French national	France	Prospective cohort	Both	7	35	50	Self-administered questionnaire	Incidence	Self-administered		416	2205	Low back pain in the past year				

						electricity and gas company in which there positions were highly exposed to physical stress									questionnaire								
lung_cancer	419717		Kubik	2002		Hospital-based cases and controls from Prague University	Czechia	Case-control	Female		25	89	Administered interviews and questionnaires	Incidence	Microscopically verified cases				Lung cancer	269	1348	Case contacts	
tb	348046		Kolappan	2002		30 villages from two areas of Tiruvallur district of Tamil Nadu	India	Nested case-control	Male		20	50	Interview	Incidence	Biomarker				Tuberculosis	85	459	Subjects who were screened and declared free of tuberculosis	
cvd	336682		Jonsdottir	2002	Reykjavik Study	Residents of Reykjavik	Iceland	Prospective cohort	Both	28	30	84	Administered questionnaire	Incidence or Mortality	Medical records, hospitalization records, vital records, autopsy reports		2406	98569	Non-fatal myocardial infarction or coronary death (ICD-9 410-414.9)				
cvd	328257		Lam	2002		Retired military cadres in Xi'an	China	Prospective cohort	Male	12	66	105	Standardized administered questionnaire	Mortality	Hospital death certificates		66	807	Cardiovascular diseases; Ischemic heart disease; Stroke				
cvd	463932		Isaksen	2002		Residens of Tromso, Norway	Norway	Case-control	Both	26	80		Structured questionnaire	Incidence	Discharge records, disease registry, medical records				Subarachnoid hemorrhage	26	104	Population	
cvd	463953		Moons	2002		Particpants recruited from three European	Finland; The Netherlands; United Kingdom	Nested case-control	Male	42	99		Structured questionnaire	Incidence	Clinical diagnosis, disease registry,				Stroke	219	479	Cohort population	

						cohort studies									discharge records								
cvd	137303		Fowler	2002		General male elderly population of metropolitan Perth, Western Australia	Australia	Cross-sectional	Male	65	83		Self-administered questionnaire	Prevalence	Biomarker		744	4470	Peripheral artery disease				463
lung_cancer	358502		Ando	2003	Japan Collaborative Cohort (JACC)	Adults in 45 study areas	Japan	Prospective cohort	Both	9	40	79	Questionnaire	Mortality	Vital records	816614	597	100736	Lung cancer				
ihd	332104		Shaper	2003	British Regional Heart Study	Male patients in a general practice in 24 towns	United Kingdom	Prospective cohort	Male	21.8	40	59	Administered questionnaire	Incidence or Mortality	Medical records, self-reported, vital records		1133	7735	Myocardial infarction, Sudden cardiac death				
stroke	193978		Yamada	2003	Japan Collaborative Cohort Study		Japan	Prospective cohort	Both	9.9	40	79	Self-administered questionnaire	Mortality	Vital records	1099662	244	109293	Subarachnoid hemorrhage				
stroke	344318		Kurth	2003	Women's Health Study	Female health professionals	United States	Prospective cohort	Female	9	45	99	Postal questionnaire	Incidence or Mortality	Medical records, vital records	354899	70	39783	Hemorrhagic stroke				
stroke	350749		Kurth	2003	Physicians' Health Study	US male physicians	United States	Prospective cohort	Male	17.8	40	84	Postal questionnaire	Incidence or Mortality	Self-reported, medical records, familial reporting	385419	1069	22022	Total stroke (ischemic, hemorrhagic, unknown)				
stroke	332104		Shaper	2003	British Regional Heart Study	Male patients in a general practice in 24 towns	United Kingdom	Prospective cohort	Male	21.8	40	59	Administered questionnaire	Incidence or Mortality	Medical records, self-reported, vital records		440	7121	Major stroke events				
diabetes	230548		Sawada	2003		Male employees who had participated in an annual health examination at the Tokyo	Japan	Prospective cohort	Male	14	20	40	Self-administered questionnaire	Incidence	Physician diagnosis, self-report	64434	280	4745	Type 2 diabetes				

						Gas Company																	
colon_and_rectum_cancer	164581		Shimizu	2003	Takayama Study	Residents of Takayama	Japan	Prospective cohort	Both	8	35	99	Self-administered questionnaire	Incidence	Hospital diagnosis		295	29051	Histologically-diagnosed colon or rectal cancer				
colon_and_rectum_cancer	309619		Otani	2003	Japan Public Health Center-based prospective study on cancer and cardiovascular disease (JPHC study, cohorts I and II)	Residents residing covered be five public health centers in five prefectures (Iwate, Akita, Nagano, Okinawa, Tokyo) in Cohort I and six public health centers in six prefectures (Ibaraki, Niigata, Kochi, Nagasaki, Okinawa, Osaka) in Cohort II	Japan	Prospective cohort	Male	7.6	40	69	Self-administered questionnaire	Incidence	Hosptial records, population-based cancer registries, death certificates	324524	457	42540	Colorectral, colon, or rectum cancer (ICD-O-2 C180-189, C199, C209)				
colon_and_rectum_cancer	347927		Limburg	2003	Iowa Women's Health Study (IWHS)	Iowa females who held a valid driver's license	United States	Prospective cohort	Female	12.6	55	69	Mailed questionnaire	Incidence & Mortality	Iowa Cancer Registry, death certificate	434700	869	34467	Colorectal cancer (ICD-O 18.0, 18.2-18.9, 19.9, 20.9 for incidence and ICD-O 153.0-154.1 for mortality)				
peptic_ulcer	338384		Rosenstock	2003		Randomly-sampled Danish adults aged 30, 40, 50, and 60 years	Denmark	Prospective cohort	Both	11	30	60	Self-administered questionnaire	Incidence	Self-reported first-time diagnosis, medical records		71	2416	Gastric ulcier (ICD-8 531), duodenal ulcer				

																			(ICD-8 532), gastro-duodenal ulcer (ICD-8 533)				
ihd	357453		Piegas	2003		Adults in 51 cities	Bazil	Case-control	Both		40	99	Administered questionnaire	Hospitalization	Clinical diagnosis, test results				ST-segment elevation acute myocardial infarction	1279	1279	Hospital	
stroke	344340		Broderick	2003		Patients in 44 hospitals	United States	Case-control	Both		18	49	Administered questionnaire	Incidence	Medical diagnosis				Symptomatic aneurysmal subarachnoid hemorrhagic stroke	312	618	Population	
liver_cancer	343586		Matsuo	2003		Residents in either the Fukouka or Saga Prefecture. Community controls were residents of Kurume City	Fukouka, Japan	Case-control	Both		40	80	Personal interviews	Incidence	Medical records				Hepatocellular carcinoma	222	548	General population and hospital controls	
liver_cancer	343588		Munaka	2003		Patients and controls from the University of Occupational and Environmental Health Hospital in Japan	Fukouka, Japan	Case-control	Both		34	92	Administered questionnaire	Incidence	Medical records				Hepatocellular carcinoma	78	138	Hospital based	
pancreatic_cancer	346137		Inoue	2003		Cancer and cancer-free outpatients of the Aichi Cancer Center Hospital	Japan	Nested case-control	Both		30	89	Self-administered questionnaire	Incidence	Disease registry				Pancreatic cancer	200	2000	Hospital based	

cvd	332104		Shaper	2003	British Regional Heart Study	Male patients in a general practice in 24 towns	United Kingdom	Prospective cohort	Male	21.8	40	59	Administered questionnaire	Incidence or Mortality	Medical records, self-reported, vital records		1573	7735	Myocardial infarction, Sudden cardiac death, Major stroke events				
cvd	344318		Kurth	2003	Women's Health Study	Female health professionals	United States	Prospective cohort	Female	9	45	99	Postal questionnaire	Incidence or Mortality	Medical records, vital records	354899	70	39783	Hemorrhagic stroke				
cvd	350749		Kurth	2003	Physicians' Health Study	US male physicians	United States	Prospective cohort	Male	17.8	40	84	Postal questionnaire	Incidence or Mortality	Self-reported, medical records, familial reporting	385419	1069	22022	Total stroke (ischemic, hemorrhagic, unknown)				
cvd	344340		Broderick	2003		Patients in 44 hospitals	United States	Case-control	Both	18	49		Administered questionnaire	Incidence	Medical diagnosis				Symptomatic aneurysmal subarachnoid hemorrhagic stroke	312	618	Population	
cvd	463924		Ohkuma	2003		Patients recruited from Aomori prefecture	Japan	Case-control	Both	32	85		Structured questionnaire	Incidence	Clinical diagnosis				Subarachnoid hemorrhage	390	390	Hospital	
cvd	464293		Okamoto	2003		Patients from 2 hospitals in Nagoya, Aichi Prefecture	Japan	Case-control	Both	30	79		Interview	Incidence	Clinical records				Subarachnoid hemorrhage	195	390	Hospital and population	
fractures	498391		Roy	2003	European Prospective Osteoporosis Study	In brief, subjects aged 50–79 years were recruited from population registers in 36 European centers.	Muliple countries in Europe	Prospective cohort	Both	3.8	50	79	Questionnaire	Incidence	Medical reports, Physician Diagnosis	24985	193	6575	Vertebral fracture				

lung_cancer	502460		Chan-Yeung	2003		lung cancer patients from the Aichi Cancer Center, National Nagoya Hospital, and Nagoya First Red Cross Hospital	Hong Kong, China	case-control	Both		25	90	self-administered questionnaire	Incidence	Histological confirmed cases				lung cancer	282	389	Varied	
lung_cancer	502421		Kreuzer	2003		residents of north eastern poland	Germany	case-control	Female		18	75	administered interview	Incidence	Histological confirmed cases				lung cancer	187	691	Varied	
lung_cancer	450624		Zatloukal	2003		Women's Health Initiative Studies	Prague, Czech Republic	case-control	Female		25	89	administered interview	Incidence	Histological confirmed cases				lung cancer	334	578	Hospital	
lung_cancer	358563		Nishino	2004	Miyagi Prefectural Cancer Registry	Residents in 14 municipalities of Miyagi Prefecture	Japan	Prospective cohort	Male	7	40	64	Self-administered questionnaire	Incidence	Clinical records, radiology and pathology records, autopsy records, mass screening records, and death certificates	166804	141	22836	Lung cancer				
ihd	174238		Doll	2004	British Doctors' Study	Male doctors residing in the UK	United Kingdom	Prospective cohort	Male	50	18	99	Self-administered questionnaire	Mortality	Professional records, vital records		7628	34439	Ischemic heart disease				
stroke	331710		Ueshima	2004	NIPPON DATA80	Japanese households	Japan	Prospective cohort	Both	14	30	99	Self-administered questionnaire	Mortality	Vital records	118044	203	9638	Stroke				
stroke	332100		Mannami	2004	JPHC Study Cohort I	Individuals registered in 14 administrative districts	Japan	Prospective cohort	Male	11	40	59	Self-administered questionnaire	Incidence or Mortality	Medical records, vital records	217151	702	19782	Stroke				
stroke	412060		Ueshima	2004	NIPPON DATA80	Household members aged 30 years or	Japan	Prospective cohort	Both	13.2	30	99	Self-administered questionnaire	Mortality	Vital records	118044	203	10546	Stroke (ICD-9 430-438)				

						more in 300 census tracts																	
stroke	174238		Doll	2004	British Doctors' Study	Male doctors residing in the UK	United Kingdom	Prospective cohort	Male	50	18	99	Self-administered questionnaire	Mortality	Professional records, vital records		3307	34439	Cerebrovascular disease				
tb	348053		Leung	2004		Residents at 18 elderly health centers in Hong Kong	China	Prospective cohort	Both	2.4	65	99	Health database	Incidence	Tuberculosis notification registry		286	42655	Tuberculosis				
diabetes	348091		Carlsson	2004	Nord-Trondelag Health Survey	Residents of the Norwegian county of Nord-Trondelag	Trondelag, Norway	Prospective cohort	Both	11	20		Self-administered questionnaire	Incidence	Self-report		1130	38805	Type 2 diabetes				
diabetes	348100		Sairenchi	2004		Japanese men and women who underwent health checkups conducted by the Ibaraki Health Service Association in Ibaraki-ken	Ibaraki, Japan	Prospective cohort	Both	8	40	79	Personal interview	Incidence	Physician diagnosis	614311	7990	128141	Type 2 diabetes mellitus				
breast_cancer	310438		Reynolds	2004	California Teachers Study	Active and retired female enrollees in the California State Teachers Retirement System who teach from the kindergarten to community college level	United States	Prospective cohort	Female	5	18	99	Baseline survey	Incidence	California Cancer Registry		2005	116544	Invasive breast cancer				
fractures	414210		Porthouse	2004		Women 70 years or older in	United Kingdom	Prospective cohort	Female	1	70		Questionnaire	Incidence	Self-reported		330	4292	Non-vertebral fracture				

						North Yorkshire and North Cumbria																	
ihd	309729		Tavani	2004		Hospitals in Northern Italy	Italy	Pooled case-control	Female		18	79	Structured interviews	Incidence	Medical records				Acute myocardial infarction	558	1044	Hospital	
ihd	357373		Ismail	2004		Young South Asian adults from Karachi	Pakistan	Case-control	Both		15	45	Standardized questionnaire	Non-fatal hospitalization	Hospital records				Non-fatal myocardial infarction	193	193	Population	
stroke	343300		Anderson	2004		Residents of Adelaide, Hobart, Perth, and Auckland	Australia; New Zealand	Case-control	Both		15	99	Structured in-person interview	Incidence	Medical records				Subarachnoid hemorrhage	432	473	Population	
stroke	357435		Hajat	2004		Residents of South London	United Kingdom	Case-control	Both		45	74	Medical records, self-reported	Incidence	Disease registry				First ischemic stroke	664	716	Population	
tb	298335		Crampin	2004		Population of Karongo District	Malawi	Case-control	Male		15	99	Interview	Incidence	Medical records, self-report, biomarker				Tuberculosis	157	373	Randomly selected in the population and matched with cases	
tb	348044		Ariyothai	2004		Population with access to health services at Taksin Hospital	Thailand	Case-control	Both		15	99	Interview	Incidence	Physician diagnosis, biomarker				New pulmonary tuberculosis	100	100	Non-tuberculosis case attending the outpatient department for medical check-up or inpatient department with no evidence of lung disease	
asthma	346730		Piipari	2004		Adults living in the Pirkanmaa	Finland	Case-control	Both		21	63	Self-administered questionnaire	Incidence	Physician diagnosis				Asthma	521	932	General population	

						Hospital District in southern Finland																	
cvd	329007		Wen	2004		Residents of northern, central, and southern Taiwan, and civil service employees and teachers	Taiwan	Pooled prospective cohort	Both	9	35	108	Interview and self-administered questionnaire	Mortality	Vital records		413	86580	Ischemic heart disease; Stroke				
cvd	343300		Anderson	2004		Residents of Adelaide, Hobart, Perth, and Auckland	Australia; New Zealand	Case-control	Both	15	99		Structured in-person interview	Incidence	Medical records				Subarachnoid hemorrhage	432	473	Population	
cvd	357435		Hajat	2004		Residents of South London	United Kingdom	Case-control	Both	45	74		Medical records, self-reported	Incidence	Disease registry				First ischemic stroke	664	716	Population	
cvd	463885		Tan	2004		Patients admitted to the First Department of Neurology of the Chang Hung Memorial Hospital, Kaohsiung	Taiwan	Case-control	Both	39	85		Interview	Incidence	Clinical diagnosis, medical records				Ischemic stroke	228	228	Hospital	
cvd	464712		Yusuf	2004		Patients recruited from 262 centers from 52 countries	Multi-location	Case-control	Both	18	99		Structured questionnaire	Incidence	Hospital admission records				Ischemic heart disease	15152	14820	Hospital and population	
bladder_ cancer	502417		Sun	2004	Shanghai Cohort Study	Chinese adults in Shanghai	Shanghai, China	Prospective cohort	Male	12.8	45	64	Structured interview	Incidence or Mortality	Re-contacts of participants and death registry	233,482	61	18244	Bladder cancer				
bladder_ cancer	502423		Hung	2004		Male residents of Brescia	Brescia, Italy	Case-control	Male		40	80	Structured interview	Incidence	Physician diagnosis, biomarker				Bladder cancer	201	214	Patients admitted to the hospitals with urological non-neoplastic	

																						diseases, including hydronephrosis, urolithiasis, malformative urological diseases, prostatic adenoma and hypertrophia, urological traumas, orchiepididymitis, hydrocele and unspecified urinary symptoms	
bladder_cancer	502444		Quirk	2004		Adult patients admitted to the Roswell Park Cancer Institute	New York, United States	Case-control	Both		19	94	Self-administered questionnaire	Incidence	Physician diagnosis, biomarker				Bladder cancer	499	1922	Patients at Roswell Park Cancer Institute who were discharged without a diagnosis of malignancy	
bladder_cancer	502425		Gaertner	2004		Cases from provincial cancer registries	Canada	Case-control	Both		20	74	Mailed questionnaires	Incidence	Cancer registry				Bladder cancer	887	2847	General population in Newfoundland and Alberta	

fractures	165283		Johansson	2004		Randomly selected residents	Sheffield, United Kindom	Prospective cohort	Female	3	75	99	Interview	Incidence	Self-report	6723	282	2113	Fracture				
fractures	414212		van der Klift	2004	Rotterdam Study	All 10,275 inhabitants of Ommord, a district in Rotterdam, the Netherlands, were invited to participate	Ommoord, Rotterdam, Netherlands	Prospective cohort	Both	6.3	55	99	Interview; clinical examination	Incidence	Clinic visit	26,561	157	4216	Vertebral fracture				
lung_cancer	250095		Marugame	2005	Three-Prefecture Cohort Study	Adults in Miyagi, Aichi, and Osaka	Japan	Prospective cohort	Both	10	40	80	Self-administered questionnaire	Mortality	Disease registry	748935	598	88153	Lung cancer				
copd	315813		Johannessen	2005	Hordaland County Study	General adult population of the county of Hordaland	Norway	Prospective cohort	Both	9	18	74	Mailed questionnaire	Incidence	Biomarker		40	908	COPD				
ihd	236194		Bjartveit	2005		Residents of Oslo and three Norwegian counties	Norway	Prospective cohort	Both	30	35	49	Self-administered questionnaire	Mortality	Vital records	1087107	2253	42722	Ischemic heart disease (ICD-9 410-414; ICD-10 I20-I25)				
ihd	336801		Woodward	2005	Asia Pacific Cohort Studies Collaboration	Varied Asian and Australasian populations	Multi-country	Pooled prospective cohort	Both	6.8	20	99	Self-reported	Incidence or Mortality	Unspecified	3000000	3976	562338	Coronary heart disease (ICD 410-414)				
stroke	331371		Iso	2005	Japan Collaborative Cohort Study for Evaluation of Cancer Risk	Residents of 45 study areas	Japan	Prospective cohort	Both	9.9	40	79	Self-administered questionnaire	Mortality	Vital records	212219	698	94683	Total stroke				
stroke	336801		Woodward	2005	Asia Pacific Cohort Studies Collaboration	Varied Asian and Australasian populations	Multi-country	Pooled prospective cohort	Both		20	99	Self-reported	Incidence or Mortality	Unspecified	3879000	5930	562338	Stroke (430-438), hemorrhagic stroke (431.0-432.9), ischemic stroke				

																			(433.0-434.9)				
diabetes	255463		Waki	2005	JPHC Cohort I	Residents in one of 14 administrative districts supervised by four public health centres (Iwate, Akita, Nagano, and Okinawa Prefectures)	Japan	Prospective cohort	Both	10	40	59	Self-administered questionnaire	Incidence	Self report, physician diagnosis		1183	28893	Type 2 diabetes				
diabetes	348105		Patja	2005		Random sample of Finnish residents	Finland	Prospective cohort	Both	21	25	64	Self-administered questionnaire	Incidence	Disease registry	850728	2770	41372	Type 2 diabetes				
esophageal_cancer	345310		Sakata	2005	Japan Collaborative Cohort Study for Evaluation of Cancer (JACC)	Residents of 45 study areas	Japan	Prospective cohort	Male	12.7	40	79	Self-administered questionnaire	Mortality	Vital records	414646	100	42,578	Esophageal cancer				
stomach_cancer	347810		Fujino	2005	Japan Collaborative Cohort (JACC) Study	Health individuals from 45 areas across Japan	Japan	Prospective cohort	Both	9.9	40	79	Self-administered questionnaire	Mortality	Death certificates	970251	757	98062	Stomach cancer (ICD-10 C16)				
breast_cancer	310414		Gram	2005	The Norwegian-Swedish Women's Lifestyle and Health Cohort Study	General female population in Norway and general population residing in the Uppsala Health Care Region in Sweden	Norway, Sweden	Prospective cohort	Female	9.1	30	50	Self-adiminstered questionnaire	Incidence	Naitonal registries of cancer and statistics	924768	1240	102098	Primary invasive breast cancer				
pancreatic_cancer	164536		Larsson	2005	Cohort of Swedish Men and Swedish Mammography Cohort	Men and women residing in central Sweden (Vastmanland, Uppsala,	Sweden	Prospective cohort	Both	6.6	18	99	Self-administered questionnaire	Incidence	Disease registry	560666	136	83053	Pancreatic cancer (ICD-9, 157)				

						and Orebro counties)																	
kidney_cancer	164492		Flaherty	2005		Female nurses and male health professionals in the US	United States	Prospective cohort	Both		30	75	Self-administered questionnaire	Incidence	Medical records, vital records		265	167144	Renal cell carcinoma				
cataracts	263023		Lindblad	2005	Swedish Mammography Cohort	Women living in Uppsala and Vastmanland Couinty, Sweden	Sweden	Prospective cohort	Female	5	49	83	Self-administered questionnaire	Incidence	Disease registry, vital records		2128	34595	Age-related cataract extraction (ICD-10, H25)				
lbp	259680		Mustard	2005	Ontario Child Health Study (OCHS)	Representative sample of young adults aged 21-34 years from the province of Ontario	Canada	Prospective cohort	Both	13	21	35	Follow-up survey questionnaire	Incidence	Self-reported in follow-up survey questionnaire		143	1928	First episode of back pain in the past year				
fractures	394006		Kanis	2005	Canadian Multicenter Osteoporosis Study; Dubbo Osteoporosis Epidemiology Study; European Vertebral Osteoporosis Study; Gothenburg I; Gothenburg II; Adult Health Study in Hiroshima; Rochester cohort; Rotterdamn; Kuopio Osteoporosis Risk Factor and Prevention study; Sheffield cohort	Residents of study areas	Multi-location	Pooled prospective cohort	Both		18	99	Varied	Incidence	Varied	249897	5444	59232	Fracture				

ihd	357927		Rastogi	2005		Urban hospitals in Bangalore and New Delhi	India	Case-control	Male		21	74	Administered interview	Hospitalization	Clinical diagnosis				Definite myocardial infarction	309	618	Hospital	
stroke	358470		Song	2005		Male Korean public servants that received health examinations by the national insurance scheme	South Korea	Case-control	Male		18	99	Self-administered questionnaire	Incidence	Hospital discharge records, physician confirmation				Acute first ischemic stroke	290	1160	Population	
liver_cancer	343590		Gelatti	2005		Cases and controls were recruited from 3 major hospitals in Brescia, at the Aviano Cancer Center and at the Pordenone General Hospital	Friuli-Venezia Giulia, Italy	Case-control	Both		40	79	Personal interviews	Incidence	Histology, cytology, sonography/tomography				Hepatocellular carcinoma	200	400	Hospital based	
liver_cancer	125938		Marrero	2005		Cases and controls from the Liver or General Medicine Clinics at University of Michigan	United States	Case-control	Both		18	99	Personal interviews	Incidence	Histopathologically confirmed cases				Hepatocellular carcinoma	70	70	Hospital based	
breast_cancer	310428		Li	2005		Three county Seattle-Puget Sound metropolitan area with no history of in situ or invasive breast cancer	United States	Case-control	Female		65	79	Interview	Incidence	Cancer Surveillance System tumor registry				Invasive breast cancer	975	1007	Selected from Health Care Financing Administration records	

fractures	414208		Kelsey	2005		Patients in five Kaiser Permanente medical centers	United States	Case-control	Both		45	99	Standardized administered questionnaire	Incidence	Radiology reports and medical records				Pelvis fracture, except for coccyx fracture	192	2402	Clinic members	
cvd	465261		Kennedy	2005	Cardiovascular Health Study	Residents of 4 communities in North Carolina, California, Maryland, and Pennsylvania	United States	Prospective cohort	Both	6	65		Medical history	Incidence	Physical examination		218	2289	Peripheral vascular disease				
cvd	464776		Hervas	2005		Residents of a basic health zone	Spain	Case-control	Both	45	95		Medical records	Incidence	Medical records, clinical diagnosis				Stroke	91	182	Population	
cvd	464989		Wanhainen	2005		Residents living in the Norsjo municipality	Sweden	Case-control	Both	65	75		Self-administered questionnaire	Incidence	Clinical diagnosis				Aortic aneurysm	35	140	Population	
cvd	465266		Zheng	2005		Black and white Americans	United States	Cross-sectional	Both	45	64		Self-report	Prevalence	Biomarker		464	15173	Peripheral artery disease				3944
cvd	465268		Jensen	2005		General population of Nord-Trondelag County	Norway	Cross-sectional	Both	40	69		Mailed questionnaire	Prevalence	Self-report		230	19748	Peripheral artery disease				6070
leukemia	502419		Kasim	2005		Participants in the Canadian National Enhanced Cancer Surveillance System		Case-control	Both		20	74	Mailed questionnaires	Incidence	Cancer registry				Leukemia	1068	5034	Individuals from provincial health insurance plans per province, and random-digit dialing	
bladder_cancer	343198		Cao	2005		Hospital patients	New York, United States	Case-control	Both		18	60	Structured questionnaire	Incidence	Biomarker				Bladder cancer	233	204	From the hopsitals blood bank	
bladder_cancer	502431		Band	2005		Patients from British Columbia	Canada	Case-control	Male		20	99	Self-administered questionnaire	Incidence	Cancer registry				Bladder cancer	1125	8492	Controls consisting of all	

						Cancer Registry																other cancer sites, excluding lung cancer and cancers of unknown primary site	
kidney_cancer	413175		Hu	2005		Patients with kidney cancer in provincial cancer registries	Canada	Case-control	Both		20	99	Self-administered questionnaire	Incidence	Cancer registry				Kidney cancer	1279	5380	From the general population within a province	
fractures	498418		Olofsson	2005	Uppsala Longitudinal Study of Adult Men	Men born between 1920 and 1924 living in the municipality of Uppsala, Sweden	Sweden	Prospective cohort	Male	30	49	51	Questionnaire and interview and examination	Incidence	Hospital records	57755	272	2322	Fracture				
lung_cancer	502433		Sreeja	2005		female patients with newly diagnosed lung cancer from Prague University Hospital	Kerela, India	case-control	Both		25	90	self-administed questionnaire	Incidence	Hospital records				lung cancer	366	1624	Hospital Visitor	
ihd	298268		Gun	2006	Health Watch	Employees of Australian Institute of Petroleum companies	Australia	Prospective cohort	Male	20	30	99	Administered questionnaire	Mortality	Vital records	242368	295		Ischemic heart disease				
asthma	346726		Genuneit	2006	International Study of Asthma and Allergies in Childhood II	9-11 year old participants in phase II of the ISAAC II study in Munich and Dresden	Germany	Prospective cohort	Both	8	9	11	Self-administered questionnaire	Incidence	Physician diagnosis		64	2936	Asthma				

asthma	346728		Gilliland	2006	Children's Health Study	School-aged children in 12 southern California commuities	California, United States	Prospective cohort	Both	8	8	15	Administered interview	Incidence	Physician diagnosis		255	2609	Asthma				
diabetes	348108		Meisinger	2006	MONICA	Residents from Augsburg, Germany	Germany	Prospective cohort	Both	18	25	74	Personal interview	Incidence	Self-report, medical records		672	10892	Type 2 diabetes mellitus				
lbp	348208		Hestbaek	2006	Danish Twin Registry	Twins of any zygosity, but the researchers noted that the twins who participated in the study are representati ve of the general population	Denmark	Prospective cohort	Both	8	12	22	Self-administered questionnaire	Incidence	Nordic Back Pain questionna ire		636	6554	Low back pain during the past year				
rheumato id_arthrit is	261500		Costenbad er	2006	Nurses' Health Study	Female nurses	United States	Prospective cohort	Femal e	22.1	30	55	Mailed questionnaire	Incidence	Self-reported physician diagnosis, medical record review	229075 9	680	103818	Rheumatoi d arthritis or other connective tissue diseases				
cvd	120239		He	2006		General elderly metropolitan population of Beijing	China	Cross-sectional	Both	60	95		Interview	Prevalence	Biomarker		462	2334	Peripheral artery disease				353
cvd	140176		Woo	2006		General elderly population of Hong Kong	Hong Kong	Cross-sectional	Both	65	83		Interview	Prevalence	Physician diagnosis, Biomarker		23	3998	Peripheral artery disease				273
cvd	465078		Allison	2006		General population free of clinically apparent cardiovascul ar disease	United States	Cross-sectional	Both	45	85		Questionnaire	Prevalence	Biomarker		275	6653	Peripheral artery disease				870

cvd	465260		Collins	2006		Primary care patients within Harris County, Texas	United States	Cross-sectional	Both	63	65		Interviewer-administered Lifestyle and Clinical Survey (LCS)	Prevalence	Biomarker		67	403	Peripheral artery disease				76
cvd	465262		Cui	2006		Elderly male population residing in farming communitie s	Japan	Cross-sectional	Male	60	79		Interview	Prevalence	Biomarker		60	1215	Peripheral artery disease				492
bladder_ cancer	502486		Puente	2006		Hospital patients and individuals from the general population	Germany, Spain, Italy, United States, France, Greece, Denmark, Canada	Case-control	Both		30	79	Interview	Incidence	Biomarker				Bladder cancer	8316	17406	From general populatio n and hospitals	
bladder_ cancer	502464		Samanic	2006		Hospital patients in Barcelona, Valles, Asturias, Alicante, and Tenerife	Spain	Case-control	Both		21	80	Computer-assisted interview	Incidence	Physician diagnosis				Bladder cancer	1219	1271	Hospital patients with other diagnose s excludin g cancer	
fractures	498421		Holmberg	2006	The Malmö Preventive Project	Middle-aged population	Sweden	Prospective cohort	Both	12.5	27	61	Questionnaire and examination	Incidence	Hospital records	416825	2535	33346	Fracture				
fractures	498406		White	2006	The Leisure World Cohort Study		Southern California, United States	Prospective cohort	Both	2	73	99	Questionnaire	Incidence	Self-report	49586	278	4769	Hip fracture				
ihd	330932		Lam	2007		Elderly Chinese adults enrolled in 18 Elderly Health Centers	Hong Kong	Prospective cohort	Both	4.1	65	99	Structured interviews	Mortality	Vital records	73291	190	56167	Ischemic heart disease				
stroke	330932		Lam	2007		Elderly Chinese adults enrolled in 18 Elderly Health Centers	Hong Kong	Prospective cohort	Both	4.1	65	99	Structured interviews	Mortality	Vital records	86992	191	54216	Stroke				

tb	359230		Pednekar	2007	Mumbai Cohort Study	Male population living in the main (island) city of Mumbai	India	Prospective cohort	Male	6	35	99	Interview	Mortality	Death certificates	490402	633	81443	Tuberculosis				
aortic_aneurism	358843		Wong	2007	Health Professionals Follow-up Study	Male health professionals	United States	Prospective cohort	Male	16	40	75	Self-administered questionnaire	Incidence	Medical records, self-report, vital records	576374	376	39352	Abdominal aortic aneurysm				
aortic_aneurism	358845		Iribarren	2007	Kaiser Permanente Multiphasic Cohort Study	Subscribers of Kaiser Permanente (KP) who attended voluntary Multiphasic Health Checkups at KP locations in Oakland and San Francisco	California, United States	Prospective cohort	Both	32	18	99	Self-administered questionnaire	Incidence	Physician diagnosis		605	104813	Abdominal aortic aneurysm				
prostate_cancer	347451		Rohrmann	2007	1963 Washington County Census Cohort	Washington County households	United States	Prospective cohort	Male	19	18		Census responses	Incidence & Mortality	Disease registry	412559	258	38857	Prostate cancer				
parkinson	359159		Thacker	2007	Cancer Prevention Study (CPS) II Nutrition Cohort	General population	United States	Prospective cohort	Both	8.4	55	84	Mailed questionnaire	Incidence	Self-report, physician diagnosis, medical records	1208264	405	143325	Definite or probable Parkinson disease				
esophageal_cancer	328339		Ozasa	2007	Japan Collaborative Cohort Study for Evaluation of Cancer (JACC)	Residents of 45 study areas	Japan	Prospective cohort	Both	12.7	40	79	Self-administered questionnaire	Mortality	Vital records	1257974	183	109778	Esophageal cancer				
leukemia	328339		Ozasa	2007	Japan Collaborative Cohort Study for Evaluation of Cancer (JACC)	Residents of 45 study areas	Japan	Prospective cohort	Both	12.7	40	79	Self-administered questionnaire	Mortality	Vital records	1257974	43	109778	Myeloid leukemia				

liver_cancer	328339		Ozasa	2007	Japan Collaborative Cohort Study for Evaluation of Cancer	Residents from 45 areas in Japan	Japan	Prospectve cohort	Both	20	40	79	Self-administered questionnaire	Mortality	Disease registry, vital records	1257974	620	109778	Liver cancer (ICD-10, C22)				
breast_cancer	328339		Ozasa	2007	Japan Collaborative Cohort Study for Evaluation of Cancer (JACC)	General population	Japan	Prospective cohort	Female	12.9	40	79	Self-adiminstered questionnaire	Mortality	Administrative records	817669	103	63600	Breast cancer				
cervical_cancer	328339		Ozasa	2007	Japan Collaborative Cohort Study for Evaluation of Cancer (JACC)	Residents of 45 study areas	Japan	Prospective cohort	Female	12.7	40	79	Self-administered questionnaire	Mortality	Vital records	709227	33	63600	Cervix uteri cancer (ICD-10 C53)				
colon_and_rectum_cancer	328339		Ozasa	2007	Japan Collaborative Cohort Study for Evaluation of Cancer (JACC)	Residents living in 45 areas all across Japan without previous history of cancer	Japan	Prospective cohort	Both	12.7	40	79	Self-administered questionnaire	Mortality	Administrative records	1393146	484	109778	Cancer of the colon (ICD-10 C18), cancer of the rectum (ICD-10 C19-20)				
pancreatic_cancer	164547		Luo	2007	Japan Public Health Center-based (JPHC) Prospective Study	All registered Japanese inhabitants in 11 public health centers throughout Japan	Japan	Prospective cohort	Male	11	40	69	Self-administered questionnaire	Incidence	Disease registry, vital records, histologically confirmed cases	1063235	224	99670	Pancreatic cancer				
kidney_cancer	328339		Ozasa	2007	Japan Collaborative Cohort Study for Evaluation of Cancer	Residents from 45 areas in Japan	Japan	Prospective cohort	Both	20	40	79	Self-administered questionnaire	Mortality	Disease registry, vital records	1257974	56	109778	Kidney cancer (ICD-10, C64)				
prostate_cancer	328339		Ozasa	2007	Japan Collaborative Cohort Study for Evaluation of Cancer (JACC)	Population recruitment in 45 across the whole of Japan	Japan	Prospective cohort	Male	12.5	40	79	Self-administered questionnaire	Mortality	Administrative records	404992	105	46178	Prostate cancer (ICD-10 C61)				

ihd	357410		Kabagambe	2007		Hispanic Americans in the central valley	Costa Rica	Case-control	Both		18	99	Administered questionnaire	Non-fatal hospitalization	Diagnosis by two cardiologists				Non-fatal acute myocardial infarction	889	1167	Population	
ihd	357427		Oliveira	2007		Residents of the catchment area of select hospitals in Porto	Portugal	Case-control	Both		18	45	Administered questionnaire	Hospitalization	Clinical diagnosis				First incidence of acute myocardial infarction	329	778	Population	
breast_cancer	310366		Magnusson	2007		Swedish-born females	Sweden	Case-control	Female		50	74	Mailed questionnaire	Incidence	Regional Cancer Registries				Invasive breast cancer	3345	3454	Randomly selected from the study population	
cvd	455688		Tapp	2007	Data from an Epidemiological Study of the Insulin Resistance Syndrome (DESIR)	Patients from 10 health examination centers in western central France that are insured by the French Social Security system	France	Prospective cohort	Both	6	30	65	Questionnaire	Incidence	Medical examination, including ankle brachial pressure index and symptom questions		173	3632	Peripheral vascular disease				
cvd	463449		Hozawa	2007	NIPPON DATA80	Household members older than 30 years old in 300 census tracts	Japan	Prospective cohort	Both	19	30		Administered questionnaire	Mortality	Vital records	154767	604	8912	Cardiovascular diseases				
cvd	441652		Lipska	2007		Patients at the Sree Chitra Tirunal Institute for Medical Sciences and Technology, Trivandrum, Kerala	India	Case-control	Both	15	45		Interview	Incidence	Medical diagnosis				Ischemic stroke	214	195	Hospital and population	

cvd	465264		Sritara	2007		Employees of the Electricity Generating Authority (EGAT) of Thailand	Thailand	Cross-sectional	Both	52	73		Self-administered questionnaire	Prevalence	Biomarker		121	2305	Peripheral artery disease				357
parkinson	359159		Thacker	2007	Cancer Prevention Study (CPS) II Nutrition Cohort	General population	United States	Prospective cohort	Both	8.4	55	84	Mailed questionnaire	Incidence	Self-report, physician diagnosis, medical records	1208264	405	143325	Definite or probable Parkinson disease				
kidney_cancer	164580		Setiawan	2007	Multiethnic Cohort	African Americans, Japanese, Latinos, Native Hawaiians, and Whites living in Hawaii and California	Hawaii and California, United States	Prospective cohort	Both	8.3	45	75	Self-administered questionnaire	Incidence	Cancer registry	1348881	347	161126	Kidney cancer				
fractures	414206		Robbins	2007		Postmenopausal women aged 50-79 years from 40 clinical centers	United States	Prospective cohort	Female	7.6	50	79	Interview	Incidence	Self-report	711,938	791	93676	Hip fracture				
lung_cancer	358577		Shimazu	2008	Japan Public Health Center-based Prospective study	All registered Japanese inhabitants in the ten public health center areas	Japan	Prospective cohort	Male	14	40	69	Self-administered questionnaire	Incidence	Voluntary reports from major hospitals, disease registries, and vital records	536325	561	50364	Lung cancer				
ihd	331705		Lawlor	2008	Korean National Health System Prospective Cohort Study	Male Korean public servants	South Korea	Prospective cohort	Male	10	30	64	Questionnaire	Hospitalization or Mortality	Medical claim data, vital records	648346	3329	648346	Myocardial infarction (ICD-10 I21-I24)				
stroke	155207		Chiuve	2008	Health Professionals Follow-up Study; Nurses'	Male health professionals and female nurses	United States	Prospective cohort	Both	20	35	99	Questionnaire	Incidence or Mortality	Medical records, self-reported hospitaliza		2553	114928	Total stroke				

					Health Study										tion, vital records, verbal autopsy								
stroke	330677		Kelly	2008	China National Hypertensio n Survey	Adults on mainland China	China	Prospective cohort	Both	8.3	40	99	Administered questionnaire	Incidence or Mortality	Medical records, vital records, verbal autopsy		678	155131	Stroke				
stroke	343306		Kenfield	2008	Nurses' Health Study	Female registered nurses	United States	Prospective cohort	Femal e	24	30	55	Self-administered questionnaire	Mortality	Familial reporting, vital records	232370 4	734	104519	Cerebrova scular disease				
stroke	331705		Lawlor	2008	Korean National Health System Prospective Cohort Study	Male Korean public servants	South Korea	Prospective cohort	Male	10	30	64	Questionnaire	Hospitaliz ation or Mortality	Medical claim data, vital records	571841 3	9475	648346	All strokes (I60 –I69), ischemic (I63, I67.8), intracerebr al hemorrhag e (I61), subarachn oid stroke (I60)				
diabetes	348112		NON-ENGLISH SOURCE	2008			Republic of Korea	Retrospectiv e cohort	Male					Incidence			50	1717	Type 2 diabetes				
afib_and _flutter	350084		Heeringa	2008	The Rotterdam Study	Residents of Ommoord	The Netherlands	Prospective cohort	Both	7.2	55	99	Computerized questionnaire	Incidence	ECG results		371	5668	Atrial fibrillation or atrial flutter				
aortic_an eurism	331705		Lawlor	2008	Korean National Health System Prospective Cohort Study	Korean male public servants who underwent a health examination through the Korean Medical Insurance Corporation	Republic of Korea	Prospective cohort	Male	10	30	64	Self-administered questionnaire	Incidence & Mortality	Vital records, hospital admission data	447212 9	269	648346	Aortic aneurysm (ICD-10, I71)				
colon_an d_rectum _cancer	347935		Hooker	2008		Residents of Washington County, Maryland 25 years old or	United States	Prospective cohort	Both	11.8	25	99	Mailed questionnaire	Incidence	Washingto n County Cancer Registry	539590	148	45749	Rectal carcinoma (ICD-8 154.0-154.8)				

						older and having no prior cancer diagnosis																	
lbp	348206		Mikkonen	2008	Oulu Back Study (OBS)	Subcohort of Northern Finland Birth Cohort 1986 comprised of study participants who lived within 100 km of the city of Oulu	Finland	Prospective cohort	Both	2	16	16	Mailed questionnaire	Incidence	Mailed questionnaire		539	1987	Incident cases were defined as those reporting LBP at 18 but not at 16 years of age and persistent cases as those reporting LBP at both time points				
stroke	344315		Bhat	2008		Residents of the greater Baltimore-Washington area	United States	Case-control	Female		18	49	Standardized interviews	Hospitalization	Discharge surveillance, medical records				First cerebral infarction	466	604	Population	
tb	359225		Dhamgaye	2008		Population in a catchment areas of a public population-based tuberculosis hospital in Yavatnal district	India	Case-control	Male		20	99	Interview	Incidence	Physician diagnosis, biomarker				Active pulmonary tuberculosis	153	160	Healthy individuals who were accompanying other patients to the same hospital as the cases	
afib_and_flutter	350091		Maattioli	2008		Family doctor patients	Italy	Case-control	Both				Interview	Incidence	Clinical examination and ECG confirmation				Acute lone atrial fibrillation	400	400	Clinic-based	
alzheimer_other_dementia	358834		Ikeda	2008		Elderly adults from the town of Kyowa	Ibaraki, Japan	Nested case-control	Both		65	99	Personal interview	Incidence	Physician diagnosis				Dementia	208	416	General population	
cvd	330677		Kelly	2008	China National	Adults on mainland China	China	Prospective cohort	Both	8.3	40	99	Administered questionnaire	Incidence or Mortality	Medical records, vital		678	155131	Stroke				

					Hypertension Survey										records, verbal autopsy								
cvd	331705		Lawlor	2008	Korean National Health System Prospective Cohort Study	Male Korean public servants	South Korea	Prospective cohort	Male	10	30	64	Self-administered questionnaire	Hospitalization or Mortality	Medical claim data, vital records	648346	3329	648346	Myocardial infarction (ICD-10 I21-I24)				
cvd	331705		Lawlor	2008	Korean National Health System Prospective Cohort Study	Male Korean public servants	South Korea	Prospective cohort	Male	10	30	64	Self-administered questionnaire	Hospitalization or Mortality	Medical claim data, vital records	5718413	9475	648346	All strokes (I60 –I69), ischemic (I63, I67.8), intracerebral hemorrhage (I61), subarachnoid stroke (I60)				
cvd	331705		Lawlor	2008	Korean National Health System Prospective Cohort Study	Male Korean public servants	South Korea	Prospective cohort	Male	10	30	64	Self-administered questionnaire	Incidence orMortality	Vital records, hospital admission data	4472129	269	648346	Aortic aneurysm (ICD-10, I71)				
cvd	343306		Kenfield	2008	Nurses' Health Study	Female registered nurses	United States	Prospective cohort	Female	24	30	55	Self-administered questionnaire	Mortality	Familial reporting, vital records	2323704	734	104519	Cerebrovascular disease				
cvd	350084		Heeringa	2008	The Rotterdam Study	Residents of Ommoord	The Netherlands	Prospective cohort	Both	7.2	55	99	Computerized questionnaire	Incidence	ECG results		371	5668	Atrial fibrillation or atrial flutter				
cvd	463463		Kubo	2008	Hisayama Study	Residents of Hisayama	Japan	Prospective cohort	Both	13	40	84	Standardized questionnaire	Incidence or Mortality	Follow-up, autopsy reports, clinical records	60353	430	5293	Ischemic stroke				
cvd	344315		Bhat	2008		Residents of the greater Baltimore-Washington area	United States	Case-control	Female	18	49		Standardized interviews	Hospitalization	Discharge surveillance, medical records				First cerebral infarction	466	604	Population	
cvd	464531		Koshy	2008		Patients from the	India	Case-control	Both	22	75			Incidence	Clinical records				Subarachnoid	122	224	Hospital	

						Department of Neurosurgery at Sree Chitra Tirunal Institute for Medical Science and Technology, Kerala													hemorrhage				
cvd	465070		Schgoer	2008		Patients from the St. John of God Hospital in Linz, Austria	Austria	Nested case-control	Both	46	88			Hospitalization	Clinical diagnosis				Peripheral vascular disease	433	433	Cohort population	
stroke	429295		Lu	2008	Swedish Women's Lifestyle and Health Cohort Study	random sample of 96,000 women born between 1943 and 1962 and residing in the Uppsala Health Care Region	Sweden	Prospective cohort	Female	11.4	30	50	Self-administered questionnaire	Incidence	Disease registry	518118	170	45449	Stroke (ischemic stroke (occlusion of cerebral arteries, IS; ICD9: 434; ICD10: I63.3–I63.9), intracerebral hemorrhage (ICD9: 431; ICD10: I61), and undefined stroke (ICD9: 436; ICD10: I64))				
lip_oral_cavity_cancer	286841		Muwonge R	2008		apparently healthy individuals aged 35 years and above living in 13 clusters called ‘panchayaths’	India	case-control	Both		35	75	self-report	incidence of oral cavity cancer	Death certificates				Lip and Oral Cavity Cancer	282	1410	population	

						(municipal administrative units in rural areas of India, with total populations of 20 000–50 000) in Trivandrum district																	
bladder_cancer	502415		Liang	2008		Patients from the Texas M.D. Anderson Cancer Center and Baylor College of Medicine	Texas, United States	Case-control	Both		31	95	Structured questionnaire	Incidence	Physician diagnosis, biomarker				Bladder cancer	386	389	Healthy individuals coming to the clinics for annual health check-ups	
bladder_cancer	502435		Covolo	2008		Male residents in Brescia province (Northern Italy)	Brescia, Italy	Case-control	Male		30	80	Structured interview	Incidence	Biomarker				Bladder cancer	197	211	Residents in Brescia province	
kidney_cancer	502427		Theis	2008		Hospital patients	Florida, United States	Case-control	Both		20	99	Structured questionnaire	Incidence	Hospital records				Kidney cancer	304	337	From random-digit dialing	
kidney_cancer	502448		Demirel	2008		Hospital patients	Russia, Romania, Poland, and Czech Republic	Case-control	Both		20	79	Standardized interviews	Incidence	Hospital records				Kidney cancer	1097	1476	Patients from the same hospitals	
copd	115406		Van Durme	2009	Rotterdam Study	Ommoord, a well-defined suburb of the city of Rotterdam	Netherlands	Prospective cohort	Both	8.8	55	99	Interview	Incidence	Hospital disharge records, medical records, biomarker	70209	648	7983	Definite or probable COPD				
stroke	335914		Honjo	2009	Japan Public Health Center-Based Prospective (JPHC) Study;	Japanese adults	Japan	Pooled prospective cohort	Both	9.6	40	79	Screening survey	Mortality	Vital records	2855395	3131	296836	Stroke				

					Three-Prefecture Cohort Study; JACC Study																		
tb	327949		Lin	2009		General population	Taiwan	Prospective cohort	Both	3.3	12	99	Administrative records	Incidence	Nathional Health Insurance database		54	16820	Active Tuberculosis				
tb	327956		Jee	2009	Korea Cancer Prevention Study	Enrollees in the National Health Insurance Corporation	South Korea	Prospective cohort	Both	12.8	30	95	Administered questionnaire	Incidence & Mortality	Physician diagnosis, medication data, outpatient and hospitalization records, death data	16540000	827	1294504	Tuberculosis				
asthma	346737		Nakamura	2009	Takayama Study	Men and women 35 years and older who resided in Takayama, Gifu	Gifu, Japan	Prospective cohort	Both	10	35	69	Self-administered questionnaire	Incidence	Physician diagnosis	120609	197	12394	Asthma				
prostate_ cancer	359211		Bulter	2009	Singapore Chinese Health Study	Premanent residents or citizens living in government-built housing	Singapore	Prospective cohort	Male	10.4	45	74	In-person interview	Incidence	Clinical diagnosis, histological confirmation	224661	181	27293	Invasive prostate cancer				
prostate_ cancer	347454		Watters	2009	NIH-AARP Diet and Health Study	AARP members of eight states	United States	Prospective cohort	Male	7	50	71	Mailed questionnaire	Incidence	Disease registry, vital records		16640	283312	Prostate cancer				
afib_and _flutter	357504		Rosengren	2009	Primary Prevention Study	Men born in Goteborg	Sweden	Prospective cohort	Male	34.3	47	56	Medical screening	Hospitalization or mortality	Hospital discharge records		1253	6903	Atrial fibrillation				
aortic_aneurism	358841		Forsdahl	2009	Tromso Study	Residents of Tromso, Norway	Norway	Prospective cohort	Both	7	25	82	Self-administered questionnaire	Incidence	Physician diagnosis, ulstrasound examination		119	4345	Abdominal aortic aneurysm				
alzheimer_other_ dementia	413859		Alonso	2009	Seven Countries Study	Participants from 13	Finland, Greece, Italy, the	Prospective cohort	Male	40	40	59	Self-administered questionnaire	Mortality	Vital records, clinical	258104	160	10211	Dementia				

						cohorts in 7 countries	Netherlands, Japan, Serbia, United States								records, physician diagnosis								
parkinson	359158		Driver	2009	Physicians' Health Study (PHS)	US male physicians without history of cardiovascular disease, cancer (except nonmelanoma skin cancer), or other serious illnesses	United States	Prospective cohort	Male	21.3	40	84	Mailed questionnaire	Incidence	Self-report, medical records	467316.5	563	21970	New diagnosis of Parkinson disease				
esophageal_cancer	298512		Ishiguro	2009	Japan Public Health Center-based Prospective Study on Cancer and Cardiovascular Disease (JPHC study)	Japanese residents of nine Public Health Centers	Japan	Prospective cohort	Male	14	40	59	Self-administered quesionnaire	Incidence	Vital records, Disease registry	526612	215	44970	Esophageal squamous cell carcinoma				
colon_and_rectum_cancer	347932		Gram	2009	The Norwegian Women and Cancer (NOWAC) Study	Country-wide, and population-based cohort of the female population in Norway	Norway	Prospective cohort	Female	7.8	30	69	Mailed questionnaire	Incidence	Cancer Registry of Norway	533786	425	68160	Incident cases of histologically-confirmed primary invasive colorectal cancers				
gallbladder_diseases	350723		Liu	2009	Million Women Study	Middle-aged females in England in Scotland who participated in the United Kingdom National Health Service Breast	United Kingdom	Prospective cohort	Female	6.1	40	65	Recruitment and repeat self-completed survey questionnaires	Incidence	NHS central registries	7871519	23989	1290413	Gallbladder disease (ICD-10 K80-K81)				

						Screening Service																	
ihd	357429		Oliveira	2009		White Portuguese adults in Porto	Portugal	Case-control	Both		18	99	Administered questionnaire	Non-fatal hospitalization	Clinical diagnosis				First incidence of acute myocardial infarction with more than four days of survival	918	2316	Population	
multiple_sclerosis	369022		Jafari	2009		Families attending outpatient ErasMS clinic or other neurological clinic	the Netherlands	Case-control	Both		30	81	Self-administered questionnaire	Incidence	Disease database				Multiple sclerosis	136	204	Families	
cvd	335914		Honjo	2009	Japan Public Health Center-Based Prospective (JPHC) Study; Three-Prefecture Cohort Study; JACC Study	Japanese adults	Japan	Pooled prospective cohort	Both	9.6	40	79	Screening survey	Mortality	Vital records	2855395	3131	296836	Stroke				
cvd	408671		Sandavei	2009	HUNT	Residents of Nord-Trondelag county	Norway	Prospective cohort	Both	22	28	90	Self-administered questionnaire	Hospitalization or Mortality	Hospital database, vital records		132	74845	Subarachnoid hemorrhage				
cvd	437824		Simons	2009	Dubbo study	Non-institutionalized residents of Dubbo	Australia	Prospective cohort	Both	16	59		Administered risk assessment	Hospitalization or Mortality	Hospital records, vital records, mailed survey		1273	2805	Ischemic heart disease; Ischemic stroke; Cardiovascular diseases				
cvd	463811		Chuang	2009	CVDFACTS	Residents in 5 villages in Chu-Dung and Pu-Tzu, each	Taiwan	Prospective cohort	Both	10.4	20	99	Questionnaire-based interview	Incidence or Mortality	Self-reported, vital records, insurance claims		128	6312	Ischemic stroke				

cvd	437516		Oliveira	2009		Male patients from four hospitals in Porto	Portugal	Case-control	Male	18	99		Interview	Incidence	Medical diagnosis				Ischemic heart disease	638	851	Population	
cvd	463834		Urbanus	2009		Patients from 16 centers and the University Medical Centre Utrecht	The Netherlands	Case-control	Female	23	55		Structured questionnaire	Incidence	Medical records, physician diagnosis				Ischemic heart disease; Ischemic stroke	378	628	Population	
cvd	463856		Woo	2009		Patients within 50 miles of the University of Cincinnati	United States	Case-control	Both	26	77		Interview	Incidence	Clinical records, hospital discharge records				Subarachnoid hemorrhage	339	1016	Population	
cvd	465073		Agarwal	2009		General civilian and noninstitutionalized population of the United States	United States	Cross-sectional	Both	40	91		Interview	Prevalence	Biomarker		647	7751	Peripheral artery disease				1856
cvd	465271		Cacoub	2009		General population at high risk for PAD	France	Cross-sectional	Both	55	86		Self-report	Prevalence	Biomarker		1579	5679	Peripheral artery disease				1292
parkinson	359158		Driver	2009	Physicians' Health Study (PHS)	US male physicians without history of cardiovascular disease, cancer (except nonmelanoma skin cancer), or other serious illnesses	United States	Prospective cohort	Male	21.3	40	84	Mailed questionnaire	Incidence	Self-report, medical records	467316.5	563	21970	New diagnosis of Parkinson disease				
leukemia	502395		Björk	2009		Individuals with acute myeloid leukemia and myelodyspla	Sweden	Case-control	Both		20	80	Structured interview	Incidence	Local diagnostic laboratories, physician diagnosis,				Leukemia	179	278	From the general population of Southern Sweden	

						stic syndromes in the Southern Health Care Region of Sweden									and cancer registry							and melanom a patients from hospitals	
leukemia	502397		Wong	2009		Patients in 29 hospitals in Shanghai	Shanghai, China	Case-control	Both		18	99	Structured interview	Incidence	Hospital records				Leukemia	722	1444	Patients from the same hospitals	
bladder_ cancer	502456		Kurahashi	2009	Japan Public Health Center-based Prospective Study (Cohort I and II)	Registered individuals in five Public Health Center areas	Japan	Prospective cohort	Both	12.6	40	69	Self-administered questionnaire	Incidence or Mortality	Hospital notificatio n and cancer registry	131458 6	206	104440	Bladder cancer				
bladder_ cancer	502450		Baris	2009		Residents of three states (Maine, Vermont, New Hampshire)	United States	Case-control	Both		30	79	Structured interview	Incidence	Physician diagnosis, biomarker				Bladder cancer	1170	1413	Controls were randomly selected from Departm ent of Motor Vehicle records in each state and the beneficia ry records of the Centers for Medicare and Medicaid Services	
bladder_ cancer	502446		Stern	2009		Residents of Los Angeles County and Shanghai	Shanghai, China, Los Angeles, United States	Case-control	Both		25	68	Self-administered questionnaire	Incidence	Physician diagnosis				Bladder cancer	1042	1123	Resident s in neighbou rhoods of cases in Los Angeles County	

																						and Shanghai	
bladder_cancer	502476		Wallace	2009		Residents in the New Hampshire State Department of Health and Human Services' Cancer Registry	New Hampshire, United States	Case-control	Both		25	74	Personal interview	Incidence	Physician diagnosis				Bladder cancer	857	1191	Residents from population lists obtained from the New Hampshire Department of Transportation and provided by the Centers for Medicare & Medicaid Services of New Hampshire	
lung_cancer	502234		Hosseini	2009		patients at various hospitals diagnosed with lung cancer at participating hospitals	Tehran, Iran	case-control	Both		20	99	administered interview	Incidence	clinical records, confirmed with histology and cytology				Lung cancer				
tb	327906		Wen	2010		General population	Taiwan	Prospective cohort	Both	7.9	20	99	Administered questionnaire	Incidence & Mortality	Self-report	3853845	5036	486341	Tuberculosis				
diabetes	309610		Jee	2010	Korea Cancer Prevention Study	Korean men and women who participated in a National Health Insurance Corporation	Republic of Korea	Prospective cohort	Both	14	30	95	Self-administered questionnaire	Incidence & Mortality	Medical records, vital records		102005	1236443	Diabetes (ICD-10, E11-E11x and E14-E14x				
peripheral_artery_disease	359221		St-Pierre	2010	Quebec Cardiovascular Study	Residents of seven Quebec suburbs	Canada	Prospective cohort	Male	24	35	64	Self-reported	Incidence	Medical evaluation		300	4076	Intermittent claudication				

peripheral_artery_disease	359223		Lakshmanan	2010	Health in Men Study	Men on the Western Australia electoral roll	Australia	Prospective cohort	Male	5.7	65	83	Administered questionnaire	Incidence	Self-reported and medical record review		638	11332	Peripheral arterial disease				
pancreatic_cancer	359138		Vrieling	2010	European Prospective Investigation into Cancer and Nutrition (EPIC)	Residents recruited from 23 centers in 10 European countries	Denmark, France, Germany, Greece, Italy, Netherlands, Norway, Spain, Sweden, United Kingdom	Prospective cohort	Both	8.7	35	70	Self-administered questionnaire	Incidence	Disease registry, health insurance records	4051920	524	465910	Pancreatic cancer (ICD-O-2, C25)				
lung_cancer	359451		Boffetta	2010		hospital and community-based participants from 6 locations in the United States	United States (California, Hawaii, Massachusetts, Michigan, New York)	Pooled case-control	Both		18	99	Self-reported	Incidence	Histological confirmed cases				Lung cancer, bronchioloalveolar carcinoma	799	16658	Varied	
alzheimer_other_dementia	349159		Garcia	2010		Cases were from five towns of the region of Valencia and controls from general Spain population	Spain	Case-control	Both		18	99	Personal interview	Incidence	Disease registry, personal interviews with proxies				Alzheimer's	176	246	General population	
parkinson	359157		Chen	2010		AARP members from six US states and 2 metropolitan areas	United States	Case-control	Both		50	71	Self-administered questionnaire	Incidence	Self-report, medical records				Parkinson disease	1662	303806	Portion of cohort who were not cases	
laryngeal_cancer	364113		Lubin	2010		Pooled results from the International Health and Neck Cancer Epidemiology (INHANCE	Multiple	Pooled case-control	Both		18	99	Self-reported	Incidence	Varied				Laryngeal cancer	1356	6910	Varied	

						) Consortium																	
breast_cancer	359082		Conlon	2010		Population within the catchment area of the Northestern Ontario REgional Cancer Centre	Canada	Case-control	Female		25	75	Mailed questionnaire	Incidence	Ontario Cancer Registry				Histologically-confirmed primary invasive breast cancer (ICD-9 174)	347	775	Female resident of the NEORCC catchment area who has never been diagnosed with breast cancer and aged 25-75 years	
other_pharynx_cancer	364113		Lubin	2010		Cases and controls from 15 case-control studies	Italy, Switzerland, Slovakia, Romania, Hungary, Poland, Russia, United States, Puerto Rico, Argentina, Cuba, Brazil, Spain, Ireland, Canada, Australia, India, Sudan, South Sudan	Pooled case-control	Both		18	99	Subject interview	Incidence	Administrative records, diseaase registry				Pharynx cancer (invasive cancer of the oropharynx, hypopharynx, unspecified phayrnx)	2277	9502	Hospital patients and general population	
lip_oral_cavity_cancer	364113		Lubin	2010		Cases and controls from 15 case-control studies	Italy, Switzerland, Slovakia, Romania, Hungary, Poland, Russia, United States, Puerto Rico, Argentina, Cuba, Brazil,	Pooled case-control	Both		18	99	Subject interview	Incidence	Administrative records, diseaase registry				Oral cavity cancer (invasive tumor of the oral cavity or unspecified oral cavity)	1872	9502	Hospital patients and general population	

							Spain, Ireland, Canada, Australia, India, Sudan, South Sudan																
cvd	359221		St-Pierre	2010	Quebec Cardiovascular Study	Residents of seven Quebec suburbs	Canada	Prospective cohort	Male	24	35	64	Self-reported	Incidence	Medical evaluation		300	4076	Intermittent claudication				
cvd	359223		Lakshmanan	2010	Health in Men Study	Men on the Western Australia electoral roll	Australia	Prospective cohort	Male	5.7	65	83	Administered questionnaire	Incidence	Self-reported and medical record review		638	11332	Peripheral arterial disease				
cvd	463822		Koshy	2010		Patients from Sree Chitra Tirunal Institute of Medical Science and Technology and Ananthapuri Hospitals and Research Institute at Thiruvananthapuram and Calicut Medical College at Kozhikode	India	Case-control	Both	27	76		Structured questionnaire	Incidence	Clinical diagnosis				Subarachnoid hemorrhage	163	150	Hospital	
cvd	463859		O'Donnell	2010		Patients recruited from 84 centers in 22 countries	Multi-location	Case-control	Both	28	92		Structured questionnaire	Incidence	Clinical diagnosis				Ischemic stroke; Intracerebral hemorrhage; Stroke	3000	3000	Hospital and population	
cvd	115580		Alzamora	2010		General population of the city of Barcelona and the county of Barcelones	Spain	Cross-sectional	Both	47	82		Self-administered questionnaire	Prevalence	Biomarker		286	3786	Peripheral artery disease				624

						Nord-Maresme																	
parkinson	359157		Chen	2010		AARP members from six US states and 2 metropolitan areas	United States	Case-control	Both		50	71	Self-administered questionnaire	Incidence	Self-report, medical records				Parkinson disease	1662	303806	Portion of cohort who were not cases	
bladder_cancer	502466		Meliker	2010		Residents in Genesee, Huron, Ingham, Jackson, Lapeer, Livingston, Oakland, Sanilac, Shiawassee, Tuscola, and Washtenaw	Michigan, United States	Case-control	Both		21	80	Telephone interview	Incidence	Cancer registry				Bladder cancer	411	566	From telephone directories, automobile and motorcycle registries, real estate listings, and driver’s license data	
lung_cancer	502241		Naghibzadeh-Tahami	2010		"incident lung cancer cases from Cheng Ching General Hospital and Tungs’ Taichung MetroHarbor Hospital in central	Iran	case-control	Both		40	80	self-administed questionnaire	Incidence	Disease Registry - Cancer				Lung cancer	242	242	Hospital Visitor	
copd	356165		Forey	2011		General adult population	Multiple locations	Meta-analysis	Both		18	99	Unknown	Incidence & Mortality	Depends on underlying study								
copd	356342		Omori	2011		Population which would attend a health screening examination at the Japanese Red Cross	Japan	Prospective cohort	Male	12	30	76	Interview	Incidence	Biomarker		91	913	COPD				

						Kumamoto Health Care Center																	
ihd	335762		Zhang	2011	MONICA/KORA Augsburg Cohort Study	Residents of Augsburg and two adjacent counties	Germany	Prospective cohort	Both	13.3	25	74	Face-to-face interview	Incidence or Mortality	Disease registry, vital records, medical records		387	8296	Myocardial infarction				
stroke	334814		Kondo	2011		Male Japanese workers	Japan	Prospective cohort	Male	7.5	20	61	Administered interviews	Mortality	Medical records, vital records	116053	73	25464	Stroke				
peripheral_artery_disease	350659		Conen	2011	Women's Health Study	Female healthcare professionals	United States	Prospective cohort	Female	12.7	45	99	Baseline questionnaire	Incidence	Self-reported and medical record review	507329	178	39825	Symptomatic peripheral artery disease				
alzheimer_other_dementia	328517		Rusanen	2011	Kaiser Permanente	Members of the Kaiser Permanente Medical Care Program of Northern California	California, United States	Prospective cohort	Both	23	50	60	Personal interview	Incidence	Clinical records, physician diagnosis		5367	21123	Dementia				
breast_cancer	310352		Luo	2011	Women's Health Initiative	General female postmenopausal population	United States	Prospective cohort	Female	10.3	50	79	Self-adminstered questionnaire	Incidence	Self-report, medical records		3250	79990	Invasive breast cancer				
lung_cancer	358565		Papadopoulos	2011		Female residents in 10 of the 11 French departements	France	Case-control	Female		18	76	Administered questionnaire	Incidence	Pathology reports and clinical records				Lung cancer	648	1423	Population	
nasopharyngeal_cancer	346056		Ji	2011		Han Chineses patients recruited from the Zhongman Hospital of Wuhan University in Wuhan, China	Hubei, China	Case-control	Both		15	99	Self-administered questionnaire	Incidence	Histologically confirmed cases				Nasopharyngeal carcinoma	1044	1095	General population	

cvd	334814		Kondo	2011		Male Japanese workers	Japan	Prospective cohort	Male	7.5	20	61	Administered interviews	Mortality	Medical records, vital records	116053	73	25464	Stroke				
cvd	350087		Chamberlain	2011	The Atherosclerosis Risk in Communities (ARIC) Study	Black and white adults from four US communities	United States	Prospective cohort	Both	13.1	45	64	Home and telephone interviews	Incidence	ECG results, hospital records, vital records	201355	876	15329	Atrial fibrillation and flutter				
cvd	437404		Merry	2011	CAREMA	Residents of the Maastricht region covered by the PPHVZ and MORGEN monitoring projects	The Netherlands	Prospective cohort	Both	11.1	20	59	Self-administered questionnaire	Incidence or Mortality	Vital records, hospital records	209573	420	19096	Ischemic heart disease				
cvd	464362		Ji	2011	Beijing Iron and Steel Complex	Male steelworkers	China	Prospective cohort	Male	20.84	18	74	Clinical interview	Incidence or Mortality	Self-reported, hospital records, vital records, clinical reports	86288	391	4238	Cardiovascular diseases				
cvd	463814		Martin	2011		Residents in the UK primary care database	United Kingdom	Nested case-control	Both	40	84		Interview	Incidence	Medical records				Ischemic stroke	2953	10000	Cohort population	
cvd	463816		Debette	2011		Patients from neurology departments in 18 centers across 8 countries	Multi-location	Case-control	Both	24	68		Structured questionnaire	Incidence	Disease registry				Ischemic stroke	556	1170	Population	
cvd	465263		Lee	2011		Male population residing in the Dong-gu district of Gwangju Metropolitan City	South Korea	Cross-sectional	Male	51	82		Interview	Prevalence	Biomarker		103	2517	Peripheral artery disease				603
cvd	465265		Taylor-Piliae	2011		Older members of Kaiser	United States	Cross-sectional	Both	60	69		Mailed questionnaire	Prevalence	Biomarker		22	1017	Peripheral artery disease				76

						Permanente of Northern California																	
bladder_cancer	502437		Zheng	2011		Hospital patients from three referral cancer centers	Egypt	Case-control	Both		19	80	Structured interview	Incidence	Physician diagnosis				Bladder cancer	1988	2716	General population	
breast_cancer	310353		Xue	2011	Nurses' Health Study	Married female nurses living in eleven states	United States	Prospective cohort	Female	30	30	55	Questionnaire	Incidence	Self-report, medical records, vital records,	3005863	8772	111140	Invasive breast cancer				
diabetes	255434		Doi	2012		Residents in the town of Hisayama, Japan	Japan	Prospective cohort	Both	11.8	40	79	Self-administered questionnaire	Incidence	Physician diagnosis		286	1935	Type 2 diabetes				
diabetes	309584		Teratini	2012		Male workers at a Japanese steel company	Chiba, Japan	Prospective cohort	Male	4.4	18	99	Self-administered questionnaire	Incidence	Physician diagnosis, medical records	37311	644	8423	Diabetes mellitus				
lung_cancer	355970		Pesch	2012		Eight European and one Canadian case-control studies in SYNERGY database	Europe and Canada	Pooled case-control	Both		18	99	Administered interview	Incidence	Clinical records				Lung cancer	13168	16008	Varied	
lung_cancer	358552		De Matteis	2012		Cases and population controls in 216 municipalities in Lombardy	Italy	Case-control	Both		35	79	Computer-assisted interview	Incidence	Tissue pathology, cytology, or review of clinical records				Lung cancer	1943	4059	Population	
tb	369826		Alavi	2012		Population who would attend the Boo-Ali Hospital in Zahedan	Iran	Case-control	Both		18	99	Administered questionnaire	Incidence	Physician diagnosis, biomarker				Pulmonary tuberculosis	253	312	Subjects who attended Boo-Ali Hosptial and underwent a physical examination	

nasopharyngeal_cancer	346071		Fachiroh	2012		Cases from seven regional cancer centers in Thailand and controls were those who visited the patients	Thailand	Case-control	Both		18	99	Personal interview	Incidence	Clinically and patholgically confirmed cases				Nasopharyngeal carcinoma (ICD-O, C11)	681	1078	Residents who visited patients admitted in the health centers	
peptic_ulcer	349542		Al-Zubeer	2012		Mosul city	Iraq	Case-control	Both		18	99	Questionnaire	Incidence	Physician diagnosis				Superficial or deep, single or multiple, duodenal, gastric, or esophageal ulcers	180	240	Healthy people from the community; medical students and their friends and family; relatives of patients (without peptic ulcer or gastritis)	
cvd	463665		Bertoia	2012	Women's Health Initiative	Participants in 40 study sites	United States	Prospective cohort	Female	10.8			Standardized questionnaire	Mortality	Medical records, vital records, autopsy reports, lab reports		418	160291	Ischemic heart disease				
cvd	465158		Xu	2012	Strong Heart Study	American Indians in three geographic regions	United States	Prospective cohort	Both	6.3-17.4	54	74	Administered questionnaire	Incidence or Mortality	Surveillance, physical examiation, medical records	44184	761	3563	Ischemic heart disease				
cvd	350745		Kim	2012		Patients from 33 hospitals throughout the Republic of Korea and their siblings/friends	South Korea	Case-control	Both	30	84		Administered questionnaire	Incidence	Medical diagnosis				Subarachnoid hemorrhage	426	426	Population	

cvd	463669		Ohira	2012		Japanese residents from the Ikawa, Noichi, Kyowa, and Yao communities	Multi-location	Nested case-control	Both	30	84		Interview	Incidence	Medical records, vital records				Ischemic heart disease	239	717	Cohort population	
cvd	463789		Shiue	2012		Residents from Adelaide, Hobart, and Perth, Australia and Auckland, New Zealand	Australia	Case-control	Both	29	83		Interview	Incidence	Medical records, discharge records, vital records				Subarachnoid hemorrhage	432	473	Population	
cvd	464955		Duval	2012		Outpatients from clinics in 44 countries	Multi-locations	Cross-sectional	Both	50	90		Self-report	Prevalence	Self-report, medical records		2113	23169	Peripheral artery disease				452
lung_cancer	426275		Thun	2013		Participants from 7 US-based cohort studies	United States	Prospective cohort	Both	30	55	99	Administered questionnaire	Mortality	Disease registry	5724508	5905	1463295	Lung cancer				
lung_cancer	328215		Bae	2013	Seoul Male Cancer Cohort	Male beneficiaries of Korean Medical Insurance Corporation	Republic of Korea	Prospective cohort	Male	16	40	75	Self-administered questionnaire	Incidence	Disease registries	203870	123	14272	Lung cancer				
lung_cancer	358558		He	2013		Workers at a machinery factor in Xi'an	China	Prospective cohort	Both	35	33.5	69.8	Administered interviews	Mortality	Hystopathological, clinical, or radiological diagnoses	22076	45	1494	Lung cancer				
prostate_cancer	359185		Rohrmann	2013	European Prospective Investigation into Cancer and Nutrition (EPIC)	Dependent on location	Denmark, France, Germany, United Kingdom, Greece, Italy, The Netherlands, Spain, Nroway, Sweden	Prospective cohort	Male	11.9			Questionnaire	Incidence & Mortality	Disease registry, health insurance records, vital records		4623	145112	Prostate cancer				

copd	426275		Thun	2013	Cancer Prevention Study (CPS) II	All 50 states of the United States, District of Columbia, Puerto Rico, Guam	United States, Puerto Rico, Guam	Prospective cohort	Both	5.2	55	99	Mailed questionnaire	Mortality	Death certificates	3952920	2100	758097	COPD				
copd	426275		Thun	2013	Women's Health Initiative (WHI)	General female population across the United States	United States	Prospective cohort	Female	10	55	79	Survey	Mortality	Death certificates		704	150864	COPD				
ihd	236197		Pirie	2013	Million Women Study	Participants of the National Health Service Breast Screening Programme	United Kingdom	Prospective cohort	Female	12	50	69	Questionnaire	Mortality	Vital records		4458	1180652	Coronary heart disease (I21-25)				
ihd	249384		Iversen	2013	Tromso Study	Residents in the municipality of Tromso	Norway	Prospective cohort	Both	11	25	99	Self-administered questionnaire	Hospitalization or Mortality	Discharge records, Medical records, Vital records, Autopsy reports		1222	24968	Myocardial infarction (ICD-10 I20-I25, R96, R98-99)				
ihd	335266		Molshatzki	2013		Civil servants and municipal employees	Israel	Prospective cohort	Male	28.1	40	99	Administered questionnaire	Mortality	Vital records, hospital records	257442	1769	10059	Coronary heart disease (ICD 410, 411, 414, 798)				
ihd	336216		Thun	2013	HPFS; Women's Health Initiative	Varied	United States	Pooled prospective cohort	Both	10	55	99	Varied	Mortality	Vital records		3767	190695	Ischemic heart disease				
stroke	350757		Xu	2013		Elderly residents of Hong Kong	Hong Kong	Prospective cohort	Both	10.9	65	99	Standardized structured interview	Mortality	Vital records, telephone interviews		648	65510	Hemorrhagic stroke				
stroke	356591		Hippisley-Cox	2013		Patients from general practices in England and Wales	United Kingdom	Prospective cohort	Both	7	25	84	Medical records	Incidence	Medical records, vital records	24800000	77578	3549478	First incidence of stroke or transient ischemic				

																			attack (excluding hemorrhagic stroke)				
stroke	335266		Molshatzki	2013		Civil servants and municipal employees	Israel	Prospective cohort	Male	28.1	40	99	Administered questionnaire	Mortality	Vital records, hospital records	257442	665	10029	Stroke (ICD 431-438)				
stroke	336216		Thun	2013	CPS I; CPS II; NIH-AARP; ACS CPS II Nutrition Cohort; Women's Health Initiative; Nurses' Health Study; Health Professionals Follow-up Study	Varied	United States	Pooled prospective cohort	Both	10-Jun	18	99	Varied	Mortality	Vital records		17463	2222223	Any type of stroke				
lri	236197		Pirie	2013	Million Women Study	Women recruited through National Health Service Breast Screening Programme	United Kingdom	Prospective cohort	Female	12	50	69	Administered questionnaire	Mortality	Vital records	14167824	902	1180652	Pneumonia (ICD-10, J12-18)				
diabetes	236197		Pirie	2013	Million Women Study	Women recruited throught the National Health Service Breast Screening Programme	United Kingdom	Prospective cohort	Female	12	50	69	Personal interview	Mortality	Vital records		314	1180652	Diabetes (ICD-10, E10-14)				
aortic_aneurism	236197		Pirie	2013	Million Women Study	Women recruited through the National Health Service Breaset Screening Programme	United Kingdom	Prospective cohort	Female	12	50	69	Self-administered questionnaire	Mortality	Vital records		494	1180652	Aortic aneurysm (ICD-10, I71)				

aortic_an eurism	358857		Sode	2013	Copenhagen City Heart Study and Copenhagen General Population Study	Randomly selected white individuals of Danish descent	Denmark	Prospective cohort	Both	34	20	99	Self-administered questionnaire	Incidence & Mortality	Disease registry, vital records		504	71283	Aortic aneurysm (ICD-8, 441; ICD-10, 171)				
breast_ca ncer	358689		Lemogne	2013	Gaz et Electricité (GAZEL) Cohort Study	Employees of the French national gas and electricity company	France	Prospective cohort	Femal e	15.2	35	50	Mailed questionnaire	Incidence	Registry at the medical department of the French national gas and electric company		138	3697	Breast cancer				
cervical_ cancer	358583		Roura	2013	European Prospective Investigatio n into Cancer and Nutrition (EPIC)	Residents of study areas (France: enrollees in a health insurance scheme of academic employees, the Netherlands and Italy (partial): participants of a breast cancer screening program, Italy (partial) and Spain: blood donors, UK: vegetarians)	Denmark, France, Germany, Greece, Italy, the Netherlands, Norway, Spain, Sweden, the United Kingdom	Prospective cohort	Femal e	9	35	70	Medical and lifestyle questionnaires	Primary incidence	Health insurance records, disease registry, active follow-up	277523 5	1065	308036	Cervix uteri cancer (ICD-10 C53)				
colon_an d_rectum _cancer	358689		Lemogne	2013	Gaz et Electricité (GAZEL) Cohort Study	Empolyees of the French national gas and electricity company	France	Prospective cohort	Both	15.2	35	50	Mailed questionnaire	Incidence	Registry at the medical department of the French national gas and electric company	215886	125	14203	Colorectal cancer				

pancreatic_cancer	359135		Lin	2013	Japan Collaborative Cohort Study (JACC)	Residents from 45 areas throughout Japan	Japan	Prospective cohort	Both	19	40	79	Self-administered questionnaire	Mortality	Vital records	1594210	611	98354	Pancreatic cancer (ICD-10, C25)				
pancreatic_cancer	359140		Kuzmickiene	2013	Multifactorial Ischemic Heart Disease Prevention Study (MIHDPS)	Male residents of the city of Kaunas	Lithuania	Prospective cohort	Male	19.3	40	59	Personal interview	Incidence	Disease registry, vital records		77	7132	Pancreatic cancer (ICD-9, 157 or ICD-10, C25)				
rheumatoid_arthritis	350653		Di Giuseppe	2013	Swedish Mammography Cohort	All females born between 1914 and 1948 and residing in two Swedish counties (Uppsala and Vastmanland)	Sweden	Prospective cohort	Female	7.5	54	89	Mailed questionnaire	Incidence	Medical registers	254996	219	34101	Rheumatoid arthritis				
prostate_cancer	358689		Lemogne	2013	Gaz et Electricité (GAZEL) Cohort Study	Employees of the French national gas and electric company	France	Prospective cohort	Male	15.2	40	50	Mailed questionnaire	Incidence	Registry kept in the medical department of the French national gas and electric company, self-report		412	10506	Primary prostate cancer				
multiple_sclerosis	348023		Hedstrom	2013		Residents of defined study areas	Sweden	Pooled case-control	Both		16	70	Standardized questionnaire	Incidence	Disease registry, hospital records				Multiple sclerosis	7883	9264	Population	
lip_oral_cavity_cancer	359126		Fu	2013		Hospitalized residents admitted to eight different hospitals location in Beijing, Shanghai, Shandong, Jiangsu, Hunan,	China	Case-control	Male		31	99	Subject interview	Incidence	Primary patologically-confirmed diagnosis				Oral squamous cell carcinoma	319	428	Hospital patients from oral and maxillofacial surgergy department, head and neck surgery departme	

						Hubei, Guangdong, Guangxi																nt, orthognathic surgery department, traum ward	
cvd	236197		Pirie	2013	Million Women Study	Participants of the National Health Service Breast Screening Programme	United Kingdom	Prospective cohort	Female	12	50	69	Self-administered questionnaire	Mortality	Vital records		4952	1180652	Coronary heart disease (I21-25), Aortic aneurysm (ICD-10, I71)				
cvd	336216		Thun	2013	HPFS; Women's Health Initiative	Varied	United States	Pooled prospective cohort	Both	10	55	99	Varied	Mortality	Vital records		3767	190695	Ischemic heart disease				
cvd	336216		Thun	2013	CPS I; CPS II; NIH-AARP; ACS CPS II Nutrition Cohort; Women's Health Initiative; Nurses' Health Study; Health Professionals Follow-up Study	Varied	United States	Pooled prospective cohort	Both	10-Jun	18	99	Varied	Mortality	Vital records		17463	2222223	Any type of stroke				
cvd	350757		Xu	2013		Elderly residents of Hong Kong	Hong Kong	Prospective cohort	Both	10.9	65	99	Standardized structured interview	Mortality	Vital records, telephone interviews		648	65510	Hemorrhagic stroke				
cvd	334964		Gellert	2013	ESTHER	Residents of Saarland	Germany	Prospective cohort	Both	9.1	50	74	Self-administered questionnaire	Incidence or Mortality	Self-reported, clinical review, vital records		647	5864	Ischemic heart disease; Stroke; Cardiovascular diseases				
cvd	463346		Gram	2013	NOWAC	Norwegian middle-aged women	Norway	Prospective cohort	Female	14	26	71	Baseline questionnaire	Mortality	Vital records	1200000	426	85320	Cardiovascular diseases; Ischemic				

																			heart disease; Stroke				
cvd	463679		Karppi	2013	Kuopio Ischemic Heart Disease Risk Factor (KIHD) cohort	Middle-aged men from Eastern Finland living in Kuopio and rural neighboring communitie s	Finland	Prospective cohort	Male	15.9	42	60	Self-administered questionnaire	Mortality	Hospital records, vital records, clinical data	19393	59	1031	Ischemic heart disease				
cvd	330725		Zhang	2013		Residents in Baotou, Inner Mongolia of China	China	Case-control	Both	28	87		Interview	Incidence	Medical records				Subarachn oid hemorrhag e	226	434	Populatio n	
cvd	463620		Vlak	2013		Patients admitted at the Utrecht Stroke Centre of the University of Medical Center Utrecht	The Netherlands	Case-control	Both	24	86		Structured questionnaire	Incidence	Clinical diagnosis				Subarachn oid hemorrhag e	250	574	Hospital	
breast_ca ncer	310343		Bjerkaas	2013		Norwegian women born between 1899 and 1975, recruited into three large Norwegian prospective cohort studies	Norway	Prospective cohort	Femal e	14	28	75	Self-administered questionnaire	Incidence	Disease registry	416431 4	7490	302865	Breast cancer (ICD-7: 170)				
lung_can cer	328909		Everatt	2014	Kunas-Rotterdam Intervention Study (KRIS) and Multifactora l Ischemic Heart Disease Prevention	Adult males in Kuanas	Lithuania	Prospective cohort	Male	30	40	59	Administered interview	Incidence	Disease registry and vital records	133642 .4	1780	6976	Lung cancer (ICD C33-34, 162)				

					Study (MIHDPS)																		
ihd	343312		Ehteshami-Afshar	2014	Tehran Lipid and Glucose Study	Residents of District 13 in Tehran	Iran	Prospective cohort	Male	9.3	30	99	Administered questionnaire	Incidence or Mortality	Interview, medical records		257	3059	Definite and probable myocardial infarction				
breast_cancer	310321		Dossus	2014	European Prospective Investigation into Cancer and Nutrition (EPIC)	General population	Denmark, France, Germany, Greece, Italy, Netherlands, Norway, Spain, Sweden, United Kingdom	Prospective cohort	Female	11	18	99	Questionnaire	Incidence	Population cancer registries, self-report, proxy informants, health insurance records, pathology registries		9822	322988	Breast cancer				
cataracts	359118		Lindblad	2014	Cohort of Swedish Men	Men living in central Sweden (Orebro and Vastmanland)	Sweden	Prospective cohort	Male	12	45	79	Self-administered questionnaire	Incidence	Disease registry, vital records		5713	44371	Age-related cataract extraction (ICD-10, H25)				
prostate_cancer	359215		Sawada	2014	Japan Public Health Center-based study (JPHC)	General population binned into one of two cohorts covered by either a set of five public health centers or six public health centers across Japan	Japan	Prospective cohort	Male	16	40	69	Self-administered questionnaire	Incidence	Hospital records, population-based cancer registries, death certificates	769715	913	48218	Prostate cancer				
diabetes	356105		Consortium	2014		Residents from 8 out of 10 EPIC countries (26 centers)	Denmark, France, Germany, Italy, Netherlands, Spain, Sweden, United Kingdom	Case-cohort	Both		18	99	Self-administered questionnaire	Incidence	Self-report, medical records, hospital admission data, vital records				Type 2 diabetes	10327	13863	General population	
parkinson	359154		Van der Mark	2014		Population who	Netherlands	Case-control	Both		34	91	Telephone interview	Incidence	Hospital records				Parkinson disease	444	876	Selected from	

						attended departments of neurology at five hospitals in fours areas of the Netherlands																among those who attended the same hospital department of neurology as the cases	
breast_cancer	359086		Kawai	2014		Three-county Seattle-Puget Sound metropolitan areas (King, Pierce, Snohomish counties)	United States	Case-control	Female		20	44	Interview	Incidence	Cancer Surveillance System tumor registry				Invasive breast cancer	960	939	General female population who were residents of King, Pierce, and Snohomish counties	
colon_and_rectum_cancer	358658		Cross	2014		Adults aged 55-74 enrolled at ten different centers in the United States without prior cancer (except skin cancer), except those who developed a rare cancer during follow-up or self-reported Crohn's disease, ulcerative colitis, familial polyposis, Gardner's syndrome or colorectal polyps	United States	Nested case-control	Both		55	74	Self-administered questionnaire	Incidence	Medical and pathologic reports				Primary incident colorectal cancers (ICD-O-3 C180-189, C199, C209, C260)	255	254	Randomly sampled from the cohort who were free from any cancer at the time matched to a case	

cvd	343312		Ehteshami -Afshar	2014	Tehran Lipid and Glucose Study	Residents of District 13 in Tehran	Iran	Prospective cohort	Male	9.3	30	99	Administered questionnaire	Incidence or Mortality	Interview, medical records		257	3059	Definite and probable myocardial infarction				
cvd	464867		Svensjo	2014		Males born in 1941 or 1942 in Uppsala county	Sweden	Prospective cohort	Male	65	70		Screening	Incidence	Disease registry		87	2701	Aortic aneurysm				
parkinson	359154		Van der Mark	2014		Population who attended departments of neurology at five hospitals in fours areas of the Netherlands	Netherlands	Case-control	Both		34	91	Telephone interview	Incidence	Hospital records				Parkinson disease	444	876	Selected from among those who attended the same hospital department of neurology as the cases	
fractures	498378		Bawab	2014		Cases of hip fracture as well as controls were recruited from 3 hospitals in Beirut (Al Zahraa, Al Makassed and Al Sahel) and 3 hospitals in south Lebanon (Jabal Amel, Lebanese Italian and Hiram).	Lebanon	Case-control	Both		40	99	Administered questionnaire	Incidence	Medical reports				Hip fracture	65	130	Controls were matched to cases by gender and region of residence	
fractures	498383		Sommar	2014		Fracture cases were identified from a 12-year prospective injury-fracture	Västerbotten County, Sweden	Nested case-control	Female		50	69	Administered questionnaire	Incidence	injury-fracture database at Umea University Hospital				Hip fracture	158	314	Controls were selected from the same NSHDS cohort and	

						database at the Umea University Hospital.																matched for sex, age at recruitment, and date of blood sampling.	
lung_cancer	321342		Freedman	2015	NIH-AARP	AARP members	United States	Prospective cohort	Both	11	50	71	Self-administered questionnaire	Incidence	Disease registry		1973	452131	Lung cancer				
lung_cancer	413177		Yun	2015		Government employees and teachers in Korea	Republic of Korea	Prospective cohort	Both	12	20	99	Self-administered questionnaire	Incidence	Disease registry	16270692	6491	1355891	Lung cancer				
ihd	356114		Schnohr	2015	Copenhagen City Heart Study	White residents of Copenhagen	Denmark	Prospective cohort	Both	21.5	20	93	Self-administered questionnaire	Hospitalization or Mortality	Vital records, patient registry		1731	8882	Coronary heart disease (ICD 410-414, I20-I25)				
ihd	357254		Hippisley-Cox	2015	QResearch	Primary care patients	United Kingdom	Prospective cohort	Both	10	25	84	Electronic medical record	Incidence	Medical records, vital records		25480	437806	Heart failure (ICD-10 I110, I130, I42, I50)				
diabetes	356095		Akter	2015	Japan Epidemiology Collaboration on Occupational Health Study	Workers of 9 companies from various industries in Japan	Japan	Prospective cohort	Both	5	15	84	Self-administered questionnaire	Incidence	Physician diagnosis		2441	53930	Type 2 diabetes				
diabetes	356097		Hilawe	2015	Aichi Workers' Cohort Study II	Civil servants in Aichi Prefecture	Japan	Prospective cohort	Both	9	35	66	Self-administered questionnaire	Incidence	Self-report, medical records	25140	310	3338	Type 2 diabetes mellitus				
alzheimer_other_dementia	328492		Ohara	2015	Hisayama Study	Adult residents in the town of Hisayama	Japan	Prospective cohort	Both	17	65	84	Self-administered questionnaire	Incidence	Clinical records, autopsy records		395	754	All-cause Dementia, Alzheimers				
prostate_cancer	328210		Meyer	2015	National Research Program 1A, Socio-Medical Indicators	Swiss residents	Switzerland	Pooled prospective cohort	Male	18.9	14	99	Unspecified	Mortality	Vital records	676309	170	35703	Prostate cancer				

					for the Population of Switzerland (SOMIPOPS), Swiss Monitoring of trends and determinants in cardiovascular disease (MONICA), Swiss Health Survey (SHS)																		
prostate_cancer	359183		Parker	2015	Multiethnic Cohort Study	Residents of Hawaii and California	United States	Prospective cohort	Male	13.9	45	75	Self-administered questionnaire	Incidence	Disease registry		7115	75216	Prostate cancer				
nasopharyngeal_cancer	328921		Lin	2015	Guangzhou Occupational Cohort	Chinese factory workers and drivers in Guangzhou, China	Guangdong, China	Prospective cohort	Both	7.3	30	87	Employee records	Mortality	Medical records, vital records	746159	34	101823	Nasopharyngeal carcinoma (ICD-9)				
tb	359228		Marahatta	2015		Population with tuberculosis in the central development region	Nepal	Case-control	Both		10	99	Administered questionnaire	Incidence	Physician diagnosis, biomarker				Multidrug resistant tuberculosis	186	372	New smear positive pulmonary tuberculosis cases as per Nepal national tuberculosis protocol	
tb	359273		Smith	2015		Population who have health insurance through a health maintenance organization, like the Kaiser Permanente	United States	Nested case-control	Both		21	98	Medical records	Incidence	Medical records				Pulmonary tuberculosis disease	591	1098	Kaiser Permanente Northern California members who were free of	

						Northern California membership																tuberculosis	
rheumatoid_arthritis	353080		Fisher	2015		General population of four study sites: Murcia, Spain; Naples, Turin, Ragusa in Italy	Spain, Italy	Nested case-control	Both		18	99	Questionnaire	Incidence	Primary health care records, prescription records, hospital discharge records, telephone follow-up of participants, then validated by medical record review				Rheumatoid arthritis	103	309	Randomly chosen among cohort members, who were chosen from the general population	
cvd	356114		Schnohr	2015	Copenhagen City Heart Study	White residents of Copenhagen	Denmark	Prospective cohort	Both	21.5	20	93	Self-administered questionnaire	Hospitalization or Mortality	Vital records, patient registry		1731	8882	Coronary heart disease (ICD 410-414, I20-I25)				
cvd	350755		Pujades-Rodriguez	2015	CALIBER	Patients from general practices in England	Great Britain	Prospective cohort	Both	5.5	40	74	Self-reported in medical review	Incidence or Mortality	Disease registry, vital records, medical records	11600000	114859	1937360	Ischemic heart disease; Ischemic stroke; Subarachnoid hemorrhage; Intracerebral hemorrhage; Peripheral vascular disease; Aortic aneurysm				
cvd	465190		Farzan	2015	New Hampshire Skin Cancer Study	Participants of the case-control study on keratinocyte cancers	United States	Prospective cohort	Both	14	25	74	Personal interview	Mortality	Vital records	55034	312	3939	Cardiovascular diseases				

lung_cancer	502407		Schwartz	2015		newly diagnosed lung cancer patients at Hong Kong's biggest oncology center	Sao Paulo, Brazil	case-control	Both		33	90	administered interview	Incidence	Histological confirmed cases				lung cancer (ICD C33-C34)	1208	1069	Population	
ihd	356141		Lubin	2016	Atherosclerosis Risk in Communities (ARIC)	Resisdents of four study areas	United States	Prospective cohort	Both	21	45	64	Administered questionnaire, telephone interview	Incidence	Hospital records, vital records, autopsy records, telephone interviews	232002	2705	14127	Validated, definite, or probable myocardial infarction or coronary heart disease death				
stroke	350753		Lindbohm	2016	FINRISK study cohort	Finnish adults	Finland	Prospective cohort	Both	14.8	18	99	Self-administered questionnaire	Incidence or Mortality	Vital records, hospital discharge records, autopsy	1380000	492	65521	Subarachnoid hemorrhage				
cataracts	359116		Floud	2016	Million Women Study	Postmenopausal women in England and Scotland	United Kingdom (England, Scotland)	Prospective cohort	Female	10.7	50	64	Self-administered questionnaire	Incidence	Disease registry		89343	1312051	Cataract surgery				
prostate_cancer	356281		Taghizadeh	2016	Vlagtwedde-Vlaardingen study	White individuals of Dutch descent	Netherlands	Prospective cohort	Male	43	20	65	Questionnaire	Mortality	Vital records		83	8465	Prostate cancer				
peptic_ulcer	359166		Deding	2016		Danish citizens who resided in eleven municipalities covering the entire of northern Jutland	Denmark	Prospective cohort	Both	2.75	17	99	Self-administered questionnaire	Incidence	National Patient Registry		121	17525	Duodenal or gastric peptic ulcer (ICD-10 K25-K27.9)				
fractures	414201		Thorin	2016	Osteoporosis Risk Assessment study	Female residents of Malmo	Sweden	Prospective cohort	Female	10	75	75	Self-assessment questionnaire	Incidence	Medical records		420	1033	Fracture				
diabetes	356099		Hou	2016		Chinese residents	China	Case-control	Male		20	98	Self-administered questionnaire	Incidence	Physician diagnosis				Diabetes mellitus	1246	15040	General population	

diabetes	356101		Rasouli	2016		Residents of Scania county in Sweden	Sweden	Case-control	Both		18	99	Self-administered questionnaire	Incidence	Physician diagnosis				Type 2 diabetes	1188	1472	General population	
cvd	322259		Alzamora	2016	ARTPER	Residents of 24 Primary Health Centers of the metropolitan Barcelona area and Barcelones Nord-Maresme	Spain	Prospective cohort	Both	2.9	59	89	Telephone and clinical history reviews	Incidence	ABI measurements		95	2256	Peripheral vascular disease				
cvd	457129		Velescu	2016	REGICOR	Residents in Girona and three neighboring rural villages	Spain	Prospective cohort	Both	5.7	35	79	Administered MONICA questionnaire	Incidence or Mortality	Biomarker (ABI), clinical evaluation, medical records, vital records		118	5434	Peripheral vascular disease				
cvd	464806		Piotrowski	2016	WOBASZ; WOBASZ Senior	Polish residents	Poland	Pooled prospective cohort	Both	5, 8.2	20			Mortality	Vital records		568	15865	Cardiovascular diseases				
cvd	464817		Lachman	2016	EPIC-Norfolk	Registries of general practices in Norfolk	United Kingdom	Prospective cohort	Both	15	39	79	Questionnaire	Hospitalization or Mortality	Hospital database, vital records	103961	1225	10043	Cardiovascular diseases				
cvd	465192		Cooney	2016	SCORE	General population	Italy, Belgium, Denmark, Norway	Pooled prospective cohort	Both	6.0 to 10.1	65	101	Varied	Mortality	Varied	2100000	4144	205000	Ischemic heart disease; Cardiovascular diseases				
cvd	463531		Martinez	2016		Patients from Santa Ana Hospital, Granada, Spain	Spain	Case-control	Both	47	94		Interview	Incidence	Medical diagnosis				Ischemic heart disease; Stroke	134	67	Hospital	
breast_cancer	448179		Gram	2016	Norwegian Women and Cancer Study	random sample of Norwegian women	Norway	Prospective cohort	Female	13.4	34	70	Self-administered questionnaire	Incidence	Disease registry	1747584	2813	130053	Breast cancer(ICD-7: 170)				
fractures	414192		Cauley	2016	The Osteoporotic Fractures	Men aged over 65 between	United States	Prospective cohort	Male	8.6	65	99	Self-report, interview, or examination	Incidence	Self-report	50,534	178	5876	Hip fracture				

					in Men Study (MrOS)	2000-2002 in AL, MN, PA, CA, OR.																	
fractures	414194		Wiklund	2016		Participants from the Umea 85+ Gerontological Regional Database population-based cohort were interviewed and assessed	Västerbotten County, Sweden	Prospective cohort	Both	2.7	85	99	Interview	Incidence	Medical records and hospital discharge registries	2573	96	953	Hip fracture				
lung_cancer	358794		Hansen	2017		Norwegians recruited from Norwegian Counties Study, 40 Years Study, and Cohort of Norway (CONOR) Study	Norway	Prospective cohort	Both	39	20	103	Administered questionnaire	Incidence	Disease registry	11553611	6534	585583	Carcinomas of the traches, broncus, and lung (ICD-7 162)				
ihd	356107		Lv	2017	China Kadoorie Biobank Study	Able-bodied permanent residents of 10 geographic study areas	China	Prospective cohort	Both	7.2	30	79	Baseline questionnaire	Incidence or Mortality	Disease registry, vital records, insurance records, active follow-up	3300000	21857	461211	Ischemic heart disease (ICD-10 I20-I25)				
ihd	356143		Lubin	2017	US Agricultural Health Study Cohort; Finnish ATBC Study	Licensed pesticide applicators and families in North Carolina and Iowa, Male smokers in southwestern Finnish study areas	Finland, United States	Pooled prospective cohort	Both	25	50	69	Administered questionnaire, enrollment questionnaire	Incidence or Mortality	Questionnaire, vital records, disease registry	1554562	10083	118789	Coronary heart disease (ICD-10 I20-I25)				
diabetes	356103		Lv	2017	China Kadoorie Biobank cohort	Chinese residents enrolled from 10	China	Prospective cohort	Both	9	30	79	Self-administered questionnaire	Incidence	Disease registry, vital records		8784	461211	Type 2 diabetes mellitus (ICD-10,				

						study areas across China													E11 and E14)				
afib_and_flutter	324155		Zuo	2017	Hordaland Health Study	Residents of Bergen or neighbouring suburban areas	Norway	Prospective cohort	Both	11	46	74	Administered questionnaire	Hospitalization or mortality	Disease registries		538	6682	Atrial fibrillation				
afib_and_flutter	324168		Kokubo	2017	The Suita Study	Residents of Suita City	Japan	Prospective cohort	Both	14	30	79	Administered questionnaire	Incidence	Biannual health examinations, self-reported, medical record review	95180	311	6898	Atrial fibrillation or atrial flutter				
leukemia	343390		Ugai	2017	The Japan Public Health Center-based Prospective Study	Residents of 10 prefectural public health center areas	Japan	Prospective cohort	Both	18.3	40	69	Self-administered questionnaire	Incidence	Disease registry, vital records, medical records	178276 2	47	96992	Leukemia				
breast_cancer	359074		Jones	2017	Generations Study	General female population of Great Britain	United Kingdom	Prospective cohort	Female	7.7	16	102	Questionnaire	Incidence	Self-report, cancer registries	788361	1815	120927	Invasive breast cancer				
breast_cancer	359076		Van den Brandt	2017	Netherlands Cohort Study (NLCS)	General postmenopausal population	Netherlands	Case-cohort	Female	20.3	55	69	Self-adiminstered questionnaire	Incidence	Netherlands Cancer Registry and Dutch Pathology Registry		2526	4342	Postmenopausal breast cancer				
breast_cancer	359078		White	2017	National Institute of Environmental Health Sciences (NIEHS) Sister Study	Females without history of breast cancer who have a sister with diagnosed breast cancer	United States, Puerto Rico	Prospective cohort	Female	6.4	35	74	Telephone questionnaire	Incidence	Self-report	326242	1843	50733	Breast cancer				
copd	356275		Deng	2017		COPD cases and healthy volunteers who underwent routine health	China	Case-control	Both		18	99	Interview	Incidence	Physician diagnosis, biomarker				COPD, fulfilling GOLD diagnostic criteria	120	481	Healthy volunteers who underwent a routine checkup	

						checkups at the same hospital																at the same hospital where cases were indentified	
breast_cancer	359094		Arthur	2017		Females who had a biopsy for benign breast disease within the Kaiser Permanente Northwest Region health care system covering southwest Washington state and Northwest Oregon	United States	Nested case-control	Female		21	85	Medical records	Incidence	KPNW Tumor Registry				Invasive breast cancer	526	526	Females with a biopsy who were alive but had not developed breast cancer	
nasopharyngeal_cancer	373803		Chang	2017		Residents from the Zhaoqing area of Guangdong Province and the Wuzhou and Guiping/Pingnan areas of Guangxi Autonomous Region	China (Guangdong, Guangxi)	Case-control	Male		20	74	Personal interview, administered questionnaire	Incidence	Histologically confirmed cases				Nasopharyngeal carcinoma	2530	2595	General population	
cvd	463489		Aigner	2017		Patients from 26 German centers	Germany	Case-control	Both	18	55		Interview	Incidence	Clinical diagnosis				Stroke; Ischemic stroke	2125	8500	Population	
cvd	463527		Kumar	2017		Patients recruited from the All India Institute of Medical	India	Case-control	Both	27	77			Incidence	Clinical diagnosis				Ischemic stroke	250	250	Hospital	

						Sciences, New Delhi																	
cvd	463529		Lioutas	2017		Residents recruited from Framingham, Massachusetts	United States	Nested case-control	Both	50	100			Incidence	Clinical diagnosis				Intracerebral hemorrhage	106	318	Cohort population	
breast_cancer	502439		Ellingjord-Dale	2017		Norwegian women aged 50-69 who attended a mammographic	Norway	Nested case-control	Female		50	69		Breast cancer	Disease registry				Invasive breast cancer (ICD10: C50)	4402	24760	Community	
lung_cancer	502482		Lawania	2017		newly diagnosed lung cancer patients from 15 hospitals around Germany	Chandigarh, India	case-control	Both		26	90	self-administed questionnaire	Incidence	Hospital records				lung cancer (ICD C33-C34)	811	912	Population	
lung_cancer	358597		Tindle	2018	Framingham Heart Study	Adult residents of Framingham, Massacheusettes	United States	Prospective cohort	Both	28.7	18	99	Incidence	Incidence	Disease surveillance through medical record review, pathology reports, and laboratory reports	384506	543	8907	Lung cancer				
copd	356273		Leem	2018	Ansung-Ansan Cohort Study	Adult Koreans covering the urban and rural populations (population-based sample from Ansan and Ansung)	South Korea	Prospective cohort	Both	3.5	40	69	Survey	Incidence	Biomarker	22724	329	6517	COPD				
stroke	336319		Jamrozik	2018	Australian Longitudinal Study on Women's Health;	Adults in specific birth cohorts	Australia	Pooled prospective cohort	Both		65	84	Postal or face-to-face interview	Mortality	Vital records		502	23861	Stroke (ICD I60-I69, G45-G46)				

					Health in Men Study																		
other_pharynx_cancer	359128		Lu	2018	Japan Public Health Center (JPHC) Study	General population of 11 prefectures across Japan	Japan	Prospective cohort	Both	16.2	20	99	Baseline survey questionnaire	Incidence	Cancer registry	1544819	59	95525	Oropharyngeal cancer (ICD-O-3 C09-C10), hypopharyngeal cancer (ICD-O-3 C12-C13)				
lip_oral_cavity_cancer	359128		Lu	2018	Japan Public Health Center (JPHC) Study	General population of 11 prefectures across Japan	Japan	Prospective cohort	Both	16.2	20	99	Baseline survey questionnaire	Incidence	Cancer registry	1544819	74	95525	Oral cavity cancer (ICD-O-3 C01-C06				
stroke	358466		Markidan	2018		Adult males	United States	Case-control	Male		15	49	Standardized interviews	Incidence					First ischemic stroke	615	530	Population	
diabetes	413800		Radzevicie ne	2018		Cases and controls from an outpatient clinic in Kaunas, Lithuania	Lithuania	Case-control	Female		18	99	Administered questionnaire	Incidence	Physician diagnosis				Type 2 diabetes mellitus	168	336	Hospital based	
cvd	336319		Jamrozik	2018	Australian Longitudinal Study on Women's Health; Health in Men Study	Adults in specific birth cohorts	Australia	Pooled prospective cohort	Both		65	84	Postal or face-to-face interview	Mortality	Vital records		502	23861	Stroke (ICD I60-I69, G45-G46)				
cvd	364043		Imtiaz Ahmad	2018	REasons for Geographic And Racial Differences in Stroke (REGARDS)	Residents of continental US, particularly Black residents and those in the so-called 'stroke belt'	United States	Prospective cohort	Both	10.6			Computer-assisted telephone interview	Incidence	Study electrocardiogram, self-reported		954	11047	Atrial fibrillation and flutter				
cvd	412189		Millett	2018	UK Biobank	Residents assessed in 22 health centers	United Kingdom	Prospective cohort	Both	7	40	69	Face-to-face interview	Incidence or Mortality	Hospital admission data		5081	471998	Ischemic heart disease				
cvd	413802		Tan	2018		Men in 49 randomly	China	Prospective cohort	Male	15	40		Administered questionnaire	Mortality	Vital records,		18833	213221	Cardiovascular				

						selected districts/counties									residential follow-up				diseases; Ischmic heart disease; Stroke				
cvd	463016		Honda	2018	The Hisayama Study	Residents of Hisayama	Japan	Prospective cohort	Both	24	40	84	Standardized questionnaire	Incidence or Mortality	Regular monitoring, autopsy reports, clinical history, clinical data		1029	2462	Cardiovascular diseases; Ischemic heart disease; Stroke				
cvd	463196		Calling	2018	Women's Health in the Lund Area Study	Women in Southern Sweden	Sweden	Prospective cohort	Female	17	50	64	Questionnaire	Hospitalization	Hospital discharge records		205	6711	Ischemic heart disease				
cvd	463202		Kim	2018	National Health Insurance Service National Health Screening Cohort	Citizens that underwent mandatory national health checkups under the national insurance scheme	South Korea	Prospective cohort	Male	7	40		Self-reported questionnaire	Hospitalization	Hospital admissions records	836962	5333	108242	Ischemic heart disease; Stroke; Ischemic stroke; Intracerebral hemorrhage				
cvd	463336		Virtanen	2018	Whitehall II	Civil servants in 20 London-based departments	United Kingdom	Prospective cohort	Both	11	35	55	Baseline survey	Incidence	Disease registry, vital records, clinical data, hospital records	107907	799	8335	Ischemic heart disease				
cvd	463461		Veronesi	2018	MONICA-Brianza; PAMELA; SEMM	Civil servants, residents of the Biranza area	Italy	Pooled prospective cohort	Male	15	25	64	Self-reported questionnaires	Incidence or Mortality	Hospital discharge, clinical data		162	2532	Cardiovascular diseases				
cvd	464601		Crowson	2018		Patients with rheumatoid arthritis	Multi-location	Prospective cohort	Both	5.8			Baseline questionnaire	Incidence or Mortality	Medical record review, study visits		389	5638	Cardiovascular diseases				
cvd	358466		Markidan	2018		Adult males	United States	Case-control	Male	15	49		Standardized interviews	Incidence					First ischemic stroke	615	530	Population	

cvd	462833		Kivioja	2018		Patients from Helsinki University Hospital	Finland	Case-control	Both	25	49		Administered questionnaire	Incidence	Discharge records				Ischemic stroke	961	1403	Population	
cvd	463194		Ivert	2018		Residents from Stockholm, Sweden	Sweden	Nested case-control	Both	18	49		Cohort registers	Incidence	Disease registry, vital records				Cardiovascular diseases	2925	14660	Cohort population	
cvd	463298		Lu	2018		Patients residing in Shandon Province and admitted to Binzhou Medical University	China	Case-control	Both	36	88			Incidence	Clinical diagnosis				Ischemic stroke	152	168	Population	
cvd	463473		Nordendahl	2018		Patients recruited from 17 Swedish hospitals	Sweden	Case-control	Both	18	75		Structured questionnaires	Incidence	Medical records				Ischemic heart disease	785	792	Population	
cvd	463479		Zyriax	2018		Pre- and post-menopausal women admitted to the University Hospital Hamburg-Eppendorf	Germany	Case-control	Female	30	80		Self-administered questionnaire	Incidence	Clinical diagnosis				Ischemic heart disease	200	225	Population	
diabetes	502177		White	2018	Jackson Heart Study	Black folks aged 21 to 84 years who were recruited from the tri-county area surrounding Jackson, MS	Jackson, Missisipi	Prospective cohort	Both	8	21	84	Self-administered questionnaire	Incidence	Physician diagnosis	23928	479	2991	Diabetes mellitus				
cvd	462792		Banks	2019	45 and Up	General population of New South Wales	Australia	Prospective cohort	Both	7.4	45	99	Postal questionnaire	Incidence or Mortality	Hospital admissions data, vital records	1350000	11778	188167	Ischemic heart disease				
cvd	462795		Barengo	2019	The National FINRISK Study	Adult population in five study areas	Finland	Prospective cohort	Both	11.8	45	74	Self-administered questionnaire	Incidence or Mortality	Vital records, hospital			13030	Cardiovascular diseases				

															discharge records								
cvd	462821		Wu	2019		Residents of rural Tianjin, who are primarily low-income farmers	China	Prospective cohort	Both	23.16			Face-to-face interview	Incidence	Clinical examination, medical records	85346.5	638	3906	Ischemic stroke; Stroke; Hemorrhagic stroke				
cvd	463012		Pencina	2019		Varied from four publically available datasets	Multi-location	Pooled prospective cohort	Both	10	45	84	Varied	Incidence			2114	22626	Ischemic heart disease				
cvd	463471		Kouvari	2019	ATTICA, GREECS	Healthy volunteers in the greater metropolitan area of Athens; Hospitalized patients in 6 major general hospitals	Greece	Pooled prospective cohort	Both	10			Enrollment interview	Incidence or Mortality	Self-report, vital records, familial reporting		1128	5124	Cardiovascular diseases				
copd	502472		Li	2019	All participants were from the Uyghur population in Kashgar; patients	China	Case-control	Both			18	99	Questionnaire	Incidence	Physician diagnosis				COPD (a forced expiratory volume/forced vital capacity (FEV1/FVC) < 0.70 in the first second after inhalation of a	440	384	Community	
ihd	462792		Banks	2019	Sax Institute's 45 and Up Study	men and women aged 45 and over at baseline, randomly sampled from the general population of New South Wales	New South Wales, Australia	Prospective cohort	Both	7.4	45	99	Self-administered questionnaire	Incidence	Hospital admissions, death registry	1350000	11778	188167	IHD (ICD-10-AM I20-I25) as well as acute myocardial infarction (AMI, I21)				

						(NSW), Australia																	
ihd	502077		Jee	2019	Korean Life Course Health Study	Koreans who were screened by KMIC in 1992 and 1994	Republic of Korea	Prospective cohort	Male	23	20	29	Interview	Incidence & Mortality	Hospital admissions, death certificates	5191823	2786	118531	IHD ((ICD (International Classification of Diseases) 10 codes, I20– I25), acute myocardial infarction (AMI) alone (I21) and angina pectoris alone (ICD 10 codes, I20))				
ihd	432350		Zhu	2019		participants from 10 geographically diverse areas of China, including five urban and five rural areas	China	Prospective cohort	Both	10.2	30	79	Self-administered questionnaire	Mortality	Death registries	4800000	13003	452657	IHD (ICD-10 (I20-I25))				
stroke	432350		Zhu	2019		participants from 10 geographically diverse areas of China, including five urban and five rural areas	China	Prospective cohort	Both	10.2	30	79	Self-administered questionnaire	Mortality	Death registries	4800000	3987	452657	Ischemic stroke				
stroke	502077		Jee	2019	Korean Life Course Health Study	Koreans who were screened by KMIC in 1992 and 1994	Republic of Korea	Prospective cohort	Male	23	20	29	Interview	Incidence & Mortality	Hospital admissions, death certificates	5191823	2368	118531	Stroke (ICD-10 I60-I69)				
stroke	501896		Ding	2019	ARIC (Atherosclerosis Risk In Communities) Study	participants age 45 to 64 years from 4 U.S.	United States	Prospective cohort	Both	26	45	64	Interview	Incidence	Hospital admissions	347230	1106	13355	Stroke (definite or probable ischemic and				

						communities													hemorrhagic stroke cases)				
lri	413804		Hamer	2019	Health Survey for England and Scottish Health Survey	Households representative for the populations of the countries	United Kingdom, Scotland	Prospective cohort	Both	9.4	18	99	Interview	Mortality	Death registry	919,949	579	97,844	Lower respiratory infections				
peripheral_artery_disease	462792		Banks	2019	The Sax Institute's 45 and Up Study	General population of New South WAles, Australia	Australia	Cohort	Both	10	45	99	incident peripheral artery disease	Incidence	Self-report	1350000	2311	267153	Peripheral arterial disease				
peripheral_artery_disease	501896		Ding	2019	Atherosclerosis Risk in Communities (ARIC)	US communities	United States	Cohort	Both	26	45	64	hospitalization with corresponding ICD codes	Incidence	Administrative medical records or disease registries	42319	199	7117	Peripheral arterial disease				
parkinson	502009		Gallo	2019	NeuroEPIC4PD study	General population residing in defined geographical areas	Sweden, United Kingdom, Germany, Spain, Italy, Greece	Prospective cohort	Both	12.8	37	70	Interview	Incidence or Mortality	Administrative records, physician diagnosis	2666206	715	213818	Parkinson's disease				
esophageal_cancer	439528		Jin	2019		Newly registered primary cases of esophageal cancer in local cancer registries	Jiangsu, China	Case-control	Both		18		Structured interview	Incidence	Cancer registry				Esophageal cancer	2969	8019	From a demographic database for four counties in Jiangsu	
leukemia	502184		Viner	2019	Alberta's Tomorrow Project	Residents contacted through random digit dialing in Alberta	Alberta, Canada	Prospective cohort	Both	12.3	35	69	Health and Lifestyle Questionnaire	Incidence	Cancer registry	314966	98	25607	Leukemia				
breast_cancer	502184		Viner	2019	Alberta's Tomorrow Project	women aged 35–69 years from Alberta	Alberta, Canada	Prospective cohort	Female	12.3	35	69	Self-administered questionnaire	Incidence	Disease registry	203946	465	16581	Breast cancer (ICD-3)				
colon_and_rectum_cancer	502184		Viner	2019	Alberta's Tomorrow Project	Residents contacted through random digit	Alberta, Canada	Prospective cohort	Both	12.3	35	69	Health and Lifestyle Questionnaire	Incidence	Cancer registry	314966	88	25607	Colon cancer				

						dialing in Alberta																	
colon_and_rectum_cancer	502236		Lee	2019		Patients in the National Cancer Center	Korea	Case-control	Both		<50	60+	Structured interview	Incidence	Physician diagnosis, biomarker				Colon cancer	925	2775	Patients that visited the hospital for a health-screening program provided by the National Health Insurance Service	
other_pharynx_cancer	439813		Yamashita	2019		Treated Japanese patients at a university hospital	Japan	Case-control	Male		18	99	Self-administered questionnaire, interview	Incidence	Hospital records				Other pharynx cancer	61	71	Patients who were treated who were treated for inflammatory disease and benign tumors, such as chronic sinusitis, chronic tonsillitis, and benign salivary tumor	
other_pharynx_cancer	439809		Zeng	2019			China	Case-control	Both		18	99	Questionnaire	Incidence	Medical records				Other pharynx cancer	278	693	Patients with diseases unrelated to alcohol, tobacco, or dietary practices in two	

																						hospital centers	
pancreatic_cancer	502474		Yuriko N Koyanagi	2019		Japanese adults participating in 1 of 10 population studies	Japan	Prospective cohort	Male	12.7	35	104	self-report	incident diagnosis of pancreatic cancer	Administrative medical records or disease registries	727175	256	53540	Pancreatic Cancer				
pancreatic_cancer	502220		Brian Z Huang	2019	Mulitethnic Cohort (MEC)	The mulitethnic cohort participants	United States	Prospective cohort	Both		45	75	self-report	incident diagnosis of pancreatic cancer	Physician diagnosis		1,532	184,559	Pancreatic Cancer				
kidney_cancer	502217		Everatt	2019	Kaunas Rotterdam Intervention Study and the Multifactorial Ischaemic Heart Disease Prevention Study	Men living in the city of Kaunas, Lithuania	Lithuana	Prospective cohort	Male	19.2	40	59	Interview	Incidence or mortality	Administrative medical registry and death register	131322	79	6849	Kidney cancer				
rheumatoid_arthritis	502084		Xinyi Liu	2019	Nurses Health Study	participants of the NHS and NHSII studies, female nurses aged 30-50	United States	Prospective Cohort	Female	38	33	75	Self-report	incident RA	self-report		1,528	230732	Rheumatoid arthritis				
lung_cancer	502184		Viner	2019	Alberta's Tomorrow Project	adults aged 35-69 in Alberta, Canada who answered random digit dialing and had not been previously diagnosed with cancer, live in alberta for 1 year, and speak English	Alberta, Canada	Prospective Cohort	Both	12.3	35	69	Self-Report Questionnaire	Incidence	Linkage with Alberta Cancer Registry		2370	26607	prostate cancer, breast cancer, endometrial cancer, colon cancer, lung cancer, leukemia, non-hodgkin lymphoma, hematological cancers				

lung_cancer	502252		Lai	2019		lung cancer patients registered with the Kerman Cancer Registry	Taiwan, China	case-control	Both		30	80	self-administed questionnaire	Incidence	Histological confirmed cases				lung cancer	140	280	Population	
lung_cancer	502208		Jin	2019		histologically confirmed lung cancer patients from Queen Mary Hosptial	China	case-control	Female		18	95	self-administed questionnaire	Incidence	CDC-managed local cancer registries				lung cancer	331	331	Hospital	
ihd	501896		Ding	2020	ARIC Study	participants aged 45–64 years from four U.S. communities	United States	Prospective cohort	Both	26	45	64	Interview	Incidence & Mortality	Hospital admissions	276908	1798	13355	CHD (definite or probable myocardial infarction or fatal CHD)				
ihd	502134		Wilsgaard	2020	The Tromso Study	participants from the municipality of Tromso, Norway	Norway	Prospective cohort	Both	19	25	69	Self-administered questionnaire	Incidence	Disease and death registries		2139	14965	Myocardial infarction				
stroke	502250		Oshunbade	2020	Jackson Heart Study	participants, aged 21 to 84 years, who were recruited from the tricounty area surrounding Jackson, MS (Hinds, Rankin, and Madison).	Jackson, MI	Prospective cohort	Both	11.8	21	84	Self-administered questionnaire	Incidence	Self-report, hospital admission records, death certificates	52038	220	4410	Stroke				
stroke	502248		Peters	2020	UK Biobank Study	women and men 40 to 69 years of age at baseline who attended 1 of the 22 centers across the United Kingdom	United Kingdom	Prospective cohort	Both	9	40	69	Self-administered questionnaire	Incidence	Hospital admissions, death registries	4247739	4662	471971	Stroke (fatal or nonfatal ischemic stroke (I63 or I64), hemorrhagic stroke (I61), and all stroke as defined by codes				

																			I60, I61, I63, or I64 in ICD-10)				
stroke	501860		Rautalin	2020		same-sex twin pairs of Finnish origin from the older Finnish Twin Cohort	Finland	Prospective cohort	Both	43	18	99	Self-administered questionnaire	Mortality	Death registry	869469	120	16282	Subarachnoid hemorrhage (ICD codes 430 (8th and 9th versions) and I60.0 to I60.9 (10th version)				
tb	501936		Tewatia	2020		Hospital admitted patients	Uttarakhand, India	Case-control	Both		18	95	Self-report	Incidence	Hospital records				Tuberculosis	92	184	Hospital	
nasopharyngeal_cancer	502223		Hsu	2020		Patients in six hospitals	Taiwan	Case-control	Male		18	69	Structured interview	Incidence	Physician diagnosis, Biomarker				Nasopharynx cancer	1235	1262	From Health Examination Centers or ENT wards/outpatient clinics	
pancreatic_cancer	502188		Esther Molina-Montes	2020		PanGenEU study	Western Europe	case-control	Both		18	99	self-report	incidence of pancreatic cancer	Physician diagnosis				Pancreatic Cancer	2009	1532	Population	
rheumatoid_arthritis	502082		Jinma Ren	2020		CLUE I study	United States	case-control			18	76	self-report	incident RA	physician diagnosis				Rheumatoid arthritis	54	216	CLUE I cohort participants without RA	
lung_cancer	502246		Shimatani	2020		Taiwan"	Aichi, Japan	case-control	Both		35	81	administered questionnaire	Incidence	Histological confirmed cases				Lung cancer	132	132	Hospital	
lung_cancer	502143		Hawrysz	2020		JFC study	Poland	case-control	Male		45	80	self-administered questionnaire	Incidence	X-ray and CT confirmed				lung cancer	68	1808	Population	
copd	502095		Zhang	2021		community-based participants aged 40–69 years from	United Kingdom	Prospective cohort	Both	8	40	69	Self-administered questionnaire	Incidence	Medical records or death registry	6449172	7956	411569	COPD (ICD 9th Revision codes 492, 492·0,				

						across the UK													492·8, 496.X or 10th				
copd	502107		Thomson	2021		men and women aged 35 years or older, from two districts of Mexico City	Mexico	Prospective cohort	Both	20	35	89	Interview	Mortality	Death registry		375	129151	COPD (ICD-10)				
copd	502093		Ganbold	2021	Patients who had been referred to the First, Second, and Third	Ulaanbaatar, Mongolia	Case-control	Both			18	99	Self-admnistered questionnaire	Incidence	Physician diagnosis				COPD (defined as an FEV1 <70% of	181	292	Community	
stroke	501942		Sato	2021	Jichi Medical School Cohort Study		Japan	Prospective cohort	Both	10.7	18	69	Interview	Incidence	Medical records	121166	417	11324	Stroke (including intracerebral hemorrhages, cerebral infarctions. and subarachnoid hemorrhages)				
stroke	501943		Zhang	2021	UK Biobank Study	participants (39 to 74 years old) who attended 1 of the 22 assessment centers across the UK between 2006 and 2010	United Kingdom	Prospective cohort	Both	11.4	50	74	Self-administered questionnaire	Incidence	Medical records	4168774	10053	365682	Stroke (ICD-10: stroke (I60,I61,I62.9,I63,I64,I67.8,I69.0,and I69.3), ischemic stroke (I63), hemorrhagic stroke (I60 and I62.9))				
tb	501926		Li	2021	Singapore Chinese Health Study		Chinese adults from 2 major dialect groups (Hokkien	Prospective cohort study	Both	18.2	45	74	Cigarettes/day	Incidence or Mortality	Disease registry	18.2	1358	60245	Tuberculosis				

							and Cantonese)																
alzheimer_other_dementia	502162		Gong	2021	UK Biobank	Participants who attended 1 of the 22 assessment centers	United Kingdom	Prospective cohort	Both	11.8	40	69	Self-report	Incidence or Mortality	Hospital records and death register	5926266	4068	502,226	Dementia				
alzheimer_other_dementia	501943		Zhang	2021	UK Biobank	Participants who attended 1 of the 22 assessment centers	United Kingdom	Prospective cohort	Both	11.4	39	74	Self-report	Incidence or Mortality	Hospital records	4168774	5079	365,682	Dementia				
esophageal_cancer	500795		Jayalekshmi	2021		Residents of the Karunagappally taluk	Kerala, India	Prospective cohort	Both	16.7	30	84	Standardized questionnaire	Incidence or mortality	Cancer registry and death registry	1087155	158	65528	Esophageal cancer				
esophageal_cancer	502130		Mezzoiuso	2021	FRiCaM Multisite Cohort Study	Residents in the municipality of Milan	Milan, Italy	Prospective cohort	Female	15	41	76	Self-report through questionnaire	Incidence	Cancer registry	1120900	33	75324	Esophageal cancer				
esophageal_cancer	502488		Okello	2021		Patients in the endoscopy unit of Mbarara Regional Referral Hospital (MRRH), southwestern Uganda	Uganda	Case-control	Both		40	86	Interview	Incidence	Physician diagnosis				Esophageal cancer	31	54	Healthy community individuals	
stomach_cancer	502130		Mezzoiuso	2021	FRiCaM Multisite Cohort Study	Residents in the municipality of Milan	Milan, Italy	Prospective cohort	Female	15	41	76	Self-report through questionnaire	Incidence	Cancer registry	1120900	225	75324	Stomach cancer				
stomach_cancer	502141		Zhang	2021		Residents of Taixing for at least 5 years		Case-control	Both		40	85	Structured interview	Incidence	Cancer registry				Stomach cancer	944	1972	Taixing Population Registry	
leukemia	502130		Mezzoiuso	2021	FRiCaM Multisite Cohort Study	Residents in the municipality of Milan	Milan, Italy	Prospective cohort	Female	15	41	76	Self-administered questionnaire	Incidence	Cancer registry	1120900	128	75324	Leukemia				
liver_cancer	502130		Mezzoiuso	2021	FRiCaM Multisite Cohort Study	Residents in the municipality of Milan	Milan, Italy	Prospective cohort	Female	15	41	76	Self-administered questionnaire	Incidence	Cancer registry	1120900	143	75324	Liver cancer				

laryngeal_cancer	502130		Mezzoiuso	2021	FRiCaM Multisite Cohort Study	Residents in the municipality of Milan	Milan, Italy	Prospective cohort	Female	15	41	76	Self-report through questionnaire	Incidence	Cancer registry	1120900	29	75324	Laryngeal cancer				
breast_cancer	502130		Mezziouso	2021	FRiCaM Multisite Cohort Study	women aged 41-76 years who resided in the municipality of Milan	Italy	Prospective cohort	Female	15	41	76	Self-administered questionnaire	Incidence	Disease registry	1129860	2952	75324	Breast cancer (ICD10: C50)				
cervical_cancer	502130		Mezzoiuso	2021	FRiCaM Multisite Cohort Study	Residents in the municipality of Milan	Milan, Italy	Prospective cohort	Female	15	41	76	Self-administered questionnaire	Incidence	Cancer registry	1120900	62	75324	Cervical cancer				
colon_and_rectum_cancer	502484		Roh	2021		Patients who participated in the voluntary health screening program of the National Cancer Center, Korea	Korea	Prospective cohort	Both	NA	19	78	Self-administered questionnaire	Incidence	Cancer registry	NA	NA	8121	Colon cancer				
colon_and_rectum_cancer	502130		Mezzoiuso	2021	FRiCaM Multisite Cohort Study	Residents in the municipality of Milan	Milan, Italy	Prospective cohort	Female	15	41	76	Self-report through questionnaire	Incidence	Cancer registry	1120900	197	75324	Colon cancer				
colon_and_rectum_cancer	502135		Chen	2021		Participants in the MJ Health Management Institution for selfpaying medical screening in Taiwan	Taiwan	Prospective cohort	Both	7.4	40	99	Self-report through questionnaire	Incidence	Cancer registry and death registry	1731925	1972	234044	Colon cancer				
colon_and_rectum_cancer	502196		Chottanapund	2021		Patients in 11 Thai provincial hospitals	Thailand	Case-control	Both		35	99	Standardized questionnaire	Incidence	Hospital records				Colon cancer	504	997	Patients that visited the hospitals for health check ups	

other_pharynx_cancer	502194		Shewale	2021		Patients in the outpatient otolaryngology at The Ohio State University	Ohio, United States	Case-control	Both		18	99	Computer-assisted self-interview	Incidence	Hospital records				Other pharynx cancer	249	478	Patients without history of cancer seen for benign conditions	
lip_oral_cavity_cancer	502130		Angelo Giosuè Mezzoiuso	2021	FRiCAM (Risk Factors for Breast Cancer)	participants in the FRiCAM study living in Milano, Italy	Italy	Prospective cohort	Female	15	14	76	self-report	Incident diagnosis of oral cancer	Physician diagnosis		112	75,324	Lip and Oral Cavity Cancer				
pancreatic_cancer	502130		Angelo Giosuè Mezzoiuso	2021	FRiCAM (Risk Factors for Breast Cancer)	participants in the FRiCAM study living in Milano, Italy	Italy	Prospective cohort	Female	15	14	76	self-report	Incident diagnosis of pancreatic cancer	Physician diagnosis		309	75,324	Pancreatic Cancer				
pancreatic_cancer	502128		Ahmad Naghibzadeh-Tahami	2021		adults living in Kerman Province	Kerman, Iran	case-control	Both		18	99	self-report	incidence of pancreatic cancer	Administrative medical records or disease registries				Pancreatic Cancer	176	352	Population	
bladder_cancer	502130		Mezzoiuso	2021	FRiCaM Multisite Cohort Study	Residents in the municipality of Milan	Milan, Italy	Prospective cohort	Female	15	41	76	Self-administered questionnaire	Incidence	Cancer registry	1120900	170	75324	Bladder cancer				
bladder_cancer	502089		Abdolahinia	2021		Patients in a teaching hospital	Kerman, Iran	Case-control	Both		40	93	Structured questionnaire	Incidence	Hospital records				Bladder cancer	100	200	Patients from other hospitals who had undergone cystoscopy due to diseases unrelated to the bladder	
kidney_cancer	502132		Minami	2021	Japan Public Health Center-based Prospective Study	Japanese residents from 11 public health centers	Japan	Prospective cohort	Both	19.1	40	69	Self-administered questionnaire	Incidence	Hospital notification, cancer registry, and death registry	2020364	340	106003	Kidney cancer				

					(Cohort I and II)																		
kidney_c ancer	502130		Mezzoiuso	2021	FRiCaM Multisite Cohort Study	Residents in the municipality of Milan	Milan, Italy	Prospective cohort	Femal e	15	41	76	Self-administered questionnaire	Incidence	Cancer registry	112090 0	167	75324	Kidney cancer				
cataracts	501958		Jiaqing Zhang	2021	Sax Institute's 45 and Up Study		Australia	Prospective Cohort	Both	10	45	55	self-report	Incidence	incidence of cataract surgery during the follow up period before the age of 65	434675	1057	49284	Cataracts				
lung_can cer	499241		Hansen	2021	NOWAC	The Central Population Register selected a random sample of women according to the year of birth from University of Tromsø–The Arctic University of Norway.	Norway	Prospective Cohort	Femal e	25	30	70	Self-Report Questionnaire	Incidence	Clinical records	764512	1507	142508	lung cancer				
lung_can cer	502124		Weber	2021	45 and up study	adults enrolled in the 45 and up study living in New South Wales, Australia	New South Wales, Australia	Prospective Cohort	Both	10	45	99	Self-Report Questionnaire	Incidence	Linkage with Cancer Registry		18475	229028	Lung, Myelodysp lasia, Small intestine, Mesothelio ma, Haematop oietic, Multiple Myeloma, Thyroid, Brain, Endometri um, NHL, Ovary, Melanoma , Breast (female), Prostate, Renal and Ureter,				

																			Myeloid , Leukaemia , Kidney, Colorectum, Stomach, Gallbladder and extrahepatic bile ducts, Head and Neck, Pancreas , Bladder, Unknown primary site , Oesophagus, Liver, Larynx				
lung_cancer	502130		Mezzoiuso	2021	FRiCAM multicenter cohort study	all women 41-76 residing in Milan from 2003 to 2006 who had a mammogram	Italy	Prospective Cohort	Female	15	41	76	Self-Report Questionnaire	Incidence	Self-report diagnosis		9487	75324	Lung cancer				
lung_cancer	502190		Park	2021	Korean National Cancer Center Community Cohort	adults more than 20 years old diagnosed with any type of cancer	South Korea	Prospective Cohort	Both	23	20	90	Self-Report Questionnaire	Mortality	Clinical Records	939852.65	173	8542	Lung cancer				
lung_cancer	502192		Jia	2021	UK Biobank	participants in the UK biobank study, adults from England, Scotland, and Wales between 40-69	United Kingdom	Prospective Cohort	Both	5.8	40	69	Self-Report Questionnaire	Incidence	linkage with hospital records		1779	308490	Lung cancer				
lung_cancer	502147		Huang	2021		2015) of China"	Taiwan, China	case-control	Both		20	80	administered interview	Incidence	Hospital records, diagnosed with				Lung cancer				

															ICD10 C33-C44								
lung_cancer	502202		Rusmaully	2021		patients with lung cancer from Taichung Cheng Ching Hospital, Chung Shan Medical University, and Taichung Tungs' Taichung MetroHarbor Hospital in central Taiwan	France	case-control	Female		18	75	self-administed questionnaire	Incidence	diagnosis of primary cancer of the lung				lung cancer	237	474	Varied	
diabetes	502053		Huh	2022		young adults from the South Korean National Health Insurance Service (NHIS) database	Republic of Korea	Prospective cohort	Both	8.2	20	39	Self-administered questionnaire	Incidence	Disease registry	24910805	71952	3026551	Type 2 diabetes				
colon_and_rectum_cancer	502139		Ugai	2022	Nurses' Health Study, Health Professionals Follow-up Study	Females and male health professionals	United States	Prospective cohort	Both	27.8	30	75	Self-report	Incidence or mortality	Self-report, death registry, and US post office authorities	3648370	3092	131144	Colon cancer				
lip_oral_cavity_cancer	502243		Isabela Firigato	2022		OSCC patients with histologically confirmation of the diagnosis were recruited from the Heliopolis ´ Hospital, Sao ˜ Paulo, Brazil	Brazil	case-control	Both		18	99	self-report	incidence of oral cavity cancer	Physician diagnosis				Lip and Oral Cavity Cancer	234	422	Hospital	

cataracts	501887		Soheil Mehmandoost	2022		newly diagnosed patients with cataracts eligible for surgery within the next 3 months from Shafa hospital in Kerman City	Iran	case-control	Both		50	64	self-report	Incidence	Physician diagnosis				cataracts	160	320	Hospital	
lung_cancer	502075		Zhang	2022	UK Biobank	participants in the UK biobank study, adults from England, Scotland, and Wales between 40-69	United Kingdom	Prospective Cohort	Both	7.2	40	69	Self-Report Questionnaire	Incidence	Self-report diagnosis	2,454,915	1687	344107	Lung cancer				
lung_cancer	502126		Guo	2022	Cancer Screening Program in Urban China	"40–74 years old residents (40–69 years old between 2012 and	China	Prospective Cohort	Both	8	40	79	Self-Report Questionnaire	Incidence	Clinical Records	6,491,000	589	282,254	Lung cancer				
lung_cancer	502215		Huang	2022		adults with lung cancer in Tehran, Iran without suspected pulmonay metastases from a different primary tumor	Taiwan, China	case-control	Both		29	93	administered interview	Incidence	Pathologic confirmation				Lung cancer	190	380	Hospital	
lung_cancer	502213		Tse	2022		WELCA study	Hong Kong, China	case-control	Male		35	79	self-administered questionnaire	Incidence	Histological confirmed cases				lung cancer (ICD C33-C34)	716	757	Population	
aortic_aneurism	343424		Burns		Cancer Prevention Study I	Friends, neighbors and acquaintances of ACS volunteers	United States	Prospective cohort	Both	6	50	99	Self-administered questionnaire	Mortality	Vital records			786387	Aortic aneurysm (ICD-7, 451)				

						from 25 states in the US																	

Section 3: Study quality & bias assessment

For each study that met the inclusion criteria, one reviewer assessed the indicators of bias included in the extraction template (Table 2). These indicators of bias were quantified as binary covariates included in risk curve estimations upon determining significant indicators by cause (Table 7).

Table 4: Study bias characteristics

NID	Cause	Author	Year	Study design	Location	cv_adj_L0	cv_adj_L1	cv_adj_L2	cv_exposure_study	cv_older	cv_subpopulation
328492	Alzheimer's and other dementia	Ohara	2015	Prospective cohort	Japan	0	0	0	1	1	0
328517	Alzheimer's and other dementia	Rusanen	2011	Prospective cohort	United States	1	1	1	1	0	1
349159	Alzheimer's and other dementia	Garcia	2010	Case-control	Spain	0	0	0	1	0	1
358834	Alzheimer's and other dementia	Ikeda	2008	Nested case-control	Japan	0	0	0	1	1	1
358836	Alzheimer's and other dementia	Prince	1994	Case-control	United Kingdom	1	1	1	1	1	1
358838	Alzheimer's and other dementia	Shalat	1987	Case-control	United States	1	1	1	1	0	1
501943	Alzheimer's and other dementia	Zhang	2021	Prospective cohort	United Kingdom	0	0	0	1	0	0
502162	Alzheimer's and other dementia	Gong	2021	Prospective cohort	United Kingdom	1	0	0	1	0	1
173863	Aortic aneurism	Doll	1994	Prospective cohort	United Kingdom	1	0	0	0	0	1
236197	Aortic aneurism	Pirie	2013	Prospective cohort	United Kingdom	0	0	0	1	0	0
328259	Aortic aneurism	Nilsson	2001	Prospective cohort	Sweden	1	0	0	1	0	0
331705	Aortic aneurism	Lawlor	2008	Prospective cohort	Republic of Korea	0	0	0	1	0	1
343424	Aortic aneurism	Burns	1997	Prospective cohort	United States	1	1	1	1	0	1
347380	Aortic aneurism	Carstensen	1987	Prospective cohort	Sweden	1	0	0	1	0	0
350128	Aortic aneurism	Wilmink	1999	Nested case-control	England, United Kingdom	0	0	0	1	0	1
350131	Aortic aneurism	Rogot	1980	Prospective cohort	United States	1	1	1	1	0	1
358841	Aortic aneurism	Forsdahl	2009	Prospective cohort	Norway	0	0	0	1	0	0
358843	Aortic aneurism	Wong	2007	Prospective cohort	United States	0	0	0	1	0	1
358845	Aortic aneurism	Iribarren	2007	Prospective cohort	United States	0	0	0	1	0	1
358857	Aortic aneurism	Sode	2013	Prospective cohort	Denmark	0	0	0	1	0	1
111344	Asthma	Strachan	1996	Prospective cohort	United Kingdom	1	1	1	1	0	0
173863	Asthma	Doll	1994	Prospective cohort	United Kingdom	1	0	0	0	0	1
346726	Asthma	Genuneit	2006	Prospective cohort	Germany	1	0	0	1	0	1
346728	Asthma	Gilliland	2006	Prospective cohort	United States	1	0	0	1	0	1
346730	Asthma	Piipari	2004	Case-control	Finland	1	0	0	1	0	1
346737	Asthma	Nakamura	2009	Prospective cohort	Japan	0	0	0	1	0	0

346743	Asthma	Troisi	1995	Prospective cohort	United States	1	1	0	1	0	1
324155	Atrial fibrillation and flutter	Zuo	2017	Prospective cohort	Norway	0	0	0	1	0	0
324155	Atrial fibrillation and flutter	Zuo	2017	Prospective cohort	Norway	1	1	0	1	0	0
324168	Atrial fibrillation and flutter	Kokubo	2017	Prospective cohort	Japan	1	1	0	1	0	0
350084	Atrial fibrillation and flutter	Heeringa	2008	Prospective cohort	Netherlands	0	0	0	1	1	0
350084	Atrial fibrillation and flutter	Heeringa	2008	Prospective cohort	Netherlands	1	1	0	1	1	0
350091	Atrial fibrillation and flutter	Maattioli	2008	Case-control	Italy	1	0	0	1	0	0
357504	Atrial fibrillation and flutter	Rosengren	2009	Prospective cohort	Sweden	1	1	0	1	0	0
343155	Bladder cancer	Chiu	2001	Case-control	United States	0	0	0	1	0	1
343198	Bladder cancer	Cao	2005	Case-control	China	1	0	0	1	0	1
343580	Bladder cancer	Siemiatycki	1995	Case-control	Canada	1	1	1	1	0	0
344435	Bladder cancer	Burch	1989	Case-control	Canada	1	0	0	1	0	0
502089	Bladder cancer	Abdolahinia	2021	Case-control	Iran	1	0	0	1	0	1
502130	Bladder cancer	Mezzoiuso	2021	Prospective cohort	Italy	1	0	0	1	0	0
502393	Bladder cancer	Yu	1997	Case-control	China	0	0	0	1	0	1
502401	Bladder cancer	Wakai	2000	Case-control	Japan	1	0	0	1	0	1
502403	Bladder cancer	Geoffroy-Perez	2001	Case-control	France	1	0	0	1	0	1
502413	Bladder cancer	Fortuny	1999	Case-control	Denmark, France, Germany, Greece, Italy, Spain	1	0	0	1	1	0
502415	Bladder cancer	Liang	2008	Case-control	United States	1	1	0	1	0	1
502417	Bladder cancer	Sun	2004	Prospective cohort	China	1	1	1	1	0	1
502423	Bladder cancer	Hung	2004	Case-control	Italy	1	1	0	1	0	1
502425	Bladder cancer	Gaertner	2004	Case-control	Canada	0	0	0	1	0	1
502429	Bladder cancer	Taylor	1998	Case-control	United States	1	1	0	1	0	1
502431	Bladder cancer	Band	2005	Case-control	Canada	1	1	1	1	0	1
502435	Bladder cancer	Covolo	2008	Case-control	Italy	1	1	1	1	0	1
502437	Bladder cancer	Zheng	2011	Case-control	Egypt	1	1	1	1	0	1
502444	Bladder cancer	Quirk	2004	Case-control	China	1	1	1	1	0	1

502446	Bladder cancer	Stern	2009	Case-control	China, United States	1	1	0	1	0	1
502446	Bladder cancer	Stern	2009	Case-control	China, United States	1	1	1	1	0	0
502450	Bladder cancer	Baris	2009	Case-control	United States	1	0	0	1	0	1
502450	Bladder cancer	Baris	2009	Case-control	United States	1	1	0	1	0	1
502452	Bladder cancer	Burns	1991	Case-control	United States	1	1	0	1	0	1
502456	Bladder cancer	Kurahashi	2009	Prospective cohort	Japan	1	1	1	1	0	0
502458	Bladder cancer	Siemiatycki	1994	Case-control	Canada	1	1	1	1	0	0
502462	Bladder cancer	Chyou	1993	Prospective cohort	United States	1	1	1	1	0	1
502464	Bladder cancer	Samanic	2006	Case-control	Spain	1	1	1	1	0	0
502466	Bladder cancer	Meliker	2010	Case-control	United States	1	1	1	1	1	0
502468	Bladder cancer	Harris	1990	Case-control	United States	1	0	0	1	1	1
502476	Bladder cancer	Wallace	2009	Case-control	United States	1	1	0	1	0	1
502486	Bladder cancer	Puente	2006	Case-control	Germany, Spain, Italy, United States, France, Greece, Denmark, Canada	1	0	0	1	0	1
309776	Breast cancer	Morabia	1996	Case-control	Switzerland	0	0	0	1	0	1
310321	Breast cancer	Dossus	2014	Prospective cohort	Denmark, France, Germany, Greece, Italy, Netherlands, Norway, Spain, Sweden, United Kingdom	1	1	1	1	0	0
310343	Breast cancer	Bjerkaas	2013	Prospective cohort	Norway	0	0	0	1	1	0
310352	Breast cancer	Luo	2011	Prospective cohort	United States	0	0	0	1	0	0
310353	Breast cancer	Xue	2011	Prospective cohort	United States	0	0	0	0	0	1
310366	Breast cancer	Magnusson	2007	Case-control	Sweden	1	0	0	1	0	1
310414	Breast cancer	Gram	2005	Prospective cohort	Norway, Sweden	0	0	0	1	0	0
310428	Breast cancer	Li	2005	Case-control	United States	0	0	0	1	1	1
310438	Breast cancer	Reynolds	2004	Prospective cohort	United States	0	0	0	1	0	1
310463	Breast cancer	Gammon	1998	Case-control	United States	0	0	0	1	0	1
310487	Breast cancer	Chu	1990	Case-control	United States	0	0	0	1	0	1
310487	Breast cancer	Chu	1990	Case-control	United States	1	1	1	1	0	1
328339	Breast cancer	Ozasa	2007	Prospective cohort	Japan	1	0	0	1	0	0

358689	Breast cancer	Lemogne	2013	Prospective cohort	France	0	0	0	1	0	1
359074	Breast cancer	Jones	2017	Prospective cohort	United Kingdom	0	0	0	0	0	1
359076	Breast cancer	Van den Brandt	2017	Case-cohort	Netherlands	0	0	0	1	1	0
359078	Breast cancer	White	2017	Prospective cohort	United States, Puerto Rico	0	0	0	1	0	1
359082	Breast cancer	Conlon	2010	Case-control	Canada	1	1	0	1	0	1
359086	Breast cancer	Kawai	2014	Case-control	United States	1	0	0	1	0	1
359094	Breast cancer	Arthur	2017	Nested case-control	United States	1	1	1	1	0	1
448179	Breast cancer	Gram	2016	Prospective cohort	Norway	1	1	1	1	1	0
502130	Breast cancer	Mezziouso	2021	Prospective cohort	Italy	1	0	0	1	0	0
502184	Breast cancer	Viner	2019	Prospective cohort	Canada	0	0	0	1	0	1
502439	Breast cancer	Ellingjord-Dale	2017	Nested case-control	Norway	1	1	1	1	0	0
115406	COPD	Van Durme	2009	Prospective cohort	Netherlands	1	1	0	1	1	0
315813	COPD	Johannessen	2005	Prospective cohort	Norway	1	0	0	1	0	0
343461	COPD	Hirayama	1990	Prospective cohort	Japan	1	1	0	1	0	0
356165	COPD	Forey	2011	Meta-analysis	United Kingdom, Italy, Chile, Canada, United States, Poland, Sweden, Norway, China, Belgium, Spain, Greece, South Africa, Turkey, Japan, Netherlands, Denmark, Nigeria, Finland, France, India, South Korea, Czechia, Slovakia, Taiwan, Iceland, Thailand, Australia, Brazil, Mexico, Uruguay, Venezuela, Estonia, Germany, Nepal	1	1	0	1	0	1
356273	COPD	Leem	2018	Prospective cohort	Republic of Korea	1	0	0	0	0	0
356275	COPD	Deng	2017	Case-control	China	1	0	0	1	0	1
356342	COPD	Omori	2011	Prospective cohort	Japan	1	0	0	0	0	1
356344	COPD	Perez-Padilla	1996	Case-control	Mexico	1	1	1	1	0	1

426275	COPD	Thun	2013	Prospective cohort	United States	1	0	0	1	0	1
426275	COPD	Thun	2013	Prospective cohort	United States, Puerto Rico, Guam	1	0	0	1	0	1
426275	COPD	Thun	2013	Prospective cohort	United States	1	0	0	1	0	0
426275	COPD	Thun	2013	Prospective cohort	United States, Puerto Rico, Guam	1	0	0	1	0	0
502093	COPD	Ganbold	2021	Both	Mongolia	0	0	0	1	0	0
502095	COPD	Zhang	2021	Prospective cohort	United Kingdom	0	0	0	1	0	0
502107	COPD	Thomson	2021	Prospective cohort	Mexico	0	0	0	0	0	0
502472	COPD	Li	2019	Both	China	1	1	1	1	1	1
261496	Cataracts	Weintraub	2002	Prospective cohort	United States	0	0	0	0	0	1
261527	Cataracts	Hiller	1997	Prospective cohort	United States	1	0	0	1	1	1
263023	Cataracts	Lindblad	2005	Prospective cohort	Sweden	0	0	0	1	0	1
350491	Cataracts	Flaye	1989	Prospective cohort	United Kingdom	1	1	1	0	1	0
350493	Cataracts	Hankinson	1992	Prospective cohort	United States	0	0	0	1	0	0
359116	Cataracts	Floud	2016	Prospective cohort	United Kingdom	0	0	0	1	0	1
359118	Cataracts	Lindblad	2014	Prospective cohort	Sweden	0	0	0	1	0	0
359119	Cataracts	Christen	1992	Prospective cohort	United States	0	0	0	1	0	1
359119	Cataracts	Christen	1992	Prospective cohort	United States	1	1	0	1	0	1
359121	Cataracts	Phillips	1996	Case-control	Scotland, United Kingdom	1	1	0	1	0	1
501958	Cataracts	Jiaqing Zhang	2021	Prospective Cohort	Australia	1	1	1	1	0	0
328339	Cervical cancer	Ozasa	2007	Prospective cohort	Japan	1	0	0	1	0	0
347864	Cervical cancer	Ylitalo	1999	Nested case-control	Sweden	1	1	1	1	0	0
358583	Cervical cancer	Roura	2013	Prospective cohort	Denmark, France, Germany, Greece, Italy, the Netherlands, Norway, Spain, Sweden, the United Kingdom	1	1	1	1	0	0
502130	Cervical cancer	Mezzoiuso	2021	Prospective cohort	Italy	1	0	0	1	0	0
164581	Colon and rectum cancer	Shimizu	2003	Prospective cohort	Japan	0	0	0	1	1	1
309619	Colon and rectum cancer	Otani	2003	Prospective cohort	Japan	0	0	0	1	0	1
328339	Colon and rectum cancer	Ozasa	2007	Prospective cohort	Japan	1	0	0	1	1	1

343580	Colon and rectum cancer	Siemiatycki	1995	Case-control	Canada	1	1	1	1	0	0
347927	Colon and rectum cancer	Limburg	2003	Prospective cohort	United States	0	0	0	1	1	1
347932	Colon and rectum cancer	Gram	2009	Prospective cohort	Norway	0	0	0	1	0	0
347935	Colon and rectum cancer	Hooker	2008	Prospective cohort	United States	1	0	0	1	0	1
358658	Colon and rectum cancer	Cross	2014	Nested case-control	United States	0	0	0	1	1	1
358689	Colon and rectum cancer	Lemogne	2013	Prospective cohort	France	0	0	0	1	0	1
502130	Colon and rectum cancer	Mezzoiuso	2021	Prospective cohort	Italy	1	0	0	1	0	0
502135	Colon and rectum cancer	Chen	2021	Prospective cohort	Taiwan	0	0	0	1	0	1
502139	Colon and rectum cancer	Ugai	2022	Prospective cohort	United States	0	0	0	0	0	1
502184	Colon and rectum cancer	Viner	2019	Prospective cohort	Canada	0	0	0	1	0	1
502196	Colon and rectum cancer	Chottanapund	2021	Case-control	Thailand	1	0	0	1	0	0
502236	Colon and rectum cancer	Lee	2019	Case-control	Republic of Korea	0	0	0	1	0	0
502484	Colon and rectum cancer	Roh	2021	Prospective cohort	Republic of Korea	1	0	0	1	0	1
298512	Esophageal cancer	Ishiguro	2009	Prospective cohort	Japan	0	0	0	1	0	1
309841	Esophageal cancer	Gao	1994	Case-control	China	0	0	0	1	0	1
328266	Esophageal cancer	Liaw	1998	Prospective cohort	Taiwan	1	0	0	1	0	0
328339	Esophageal cancer	Ozasa	2007	Prospective cohort	Japan	1	0	0	1	0	0
339730	Esophageal cancer	Cheng	2000	Case-control	United Kingdom	1	1	0	1	0	1
343383	Esophageal cancer	Vaughan	1995	Case-control	United States	0	0	0	1	0	0
343580	Esophageal cancer	Siemiatycki	1995	Case-control	Canada	1	1	1	1	0	0
345310	Esophageal cancer	Sakata	2005	Prospective cohort	Japan	1	0	0	1	0	0
345324	Esophageal cancer	Matsuo	2001	Case-control	Japan	1	1	0	1	0	1
345328	Esophageal cancer	Yokoyama	2002	Case-control	Japan	1	1	0	1	0	0
439528	Esophageal cancer	Jin	2019	Case-control	China	0	0	0	1	0	1
500795	Esophageal cancer	Jayalekshmi	2021	Prospective cohort	India	1	1	1	1	0	0
502130	Esophageal cancer	Mezzoiuso	2021	Prospective cohort	Italy	1	0	0	1	0	0
502488	Esophageal cancer	Okello	2021	Case-control	Uganda	0	0	0	1	0	1
350721	Gallbladder disease	Stampfer	1992	Prospective cohort	United States	0	0	0	1	0	1
350723	Gallbladder disease	Liu	2009	Prospective cohort	United Kingdom	0	0	0	1	0	1
350725	Gallbladder disease	Sahi	1998	Prospective cohort	United States	0	0	0	1	0	1

351491	Gallbladder disease	Layde	1982	Prospective cohort	United Kingdom	1	1	1	1	0	1
351491	Gallbladder disease	Layde	1982	Prospective cohort	United Kingdom	0	0	0	1	0	1
165539	Ischemic heart disease	Jacobs	1999	Prospective cohort	United States, Finland, Netherlands, Italy, Croatia, Serbia, Greece, Japan	0	0	0	1	0	1
174238	Ischemic heart disease	Doll	2004	Prospective cohort	United Kingdom	1	0	0	0	0	1
236194	Ischemic heart disease	Bjartveit	1005	Prospective cohort	Norway	1	1	0	1	0	0
236197	Ischemic heart disease	Pirie	2013	Prospective cohort	United Kingdom	0	0	0	1	0	0
249384	Ischemic heart disease	Iversen	2013	Prospective cohort	Norway	1	1	1	1	0	1
298268	Ischemic heart disease	Gun	2006	Prospective cohort	Australia	1	0	0	1	0	0
309699	Ischemic heart disease	Kalandidi	1992	Case-control	Greece	1	0	0	1	0	1
309729	Ischemic heart disease	Tavani	2004	Pooled case-control	Italy	0	0	0	1	1	1
309729	Ischemic heart disease	Tavani	2004	Pooled case-control	Italy	0	0	0	1	0	1
328259	Ischemic heart disease	Nilsson	2001	Prospective cohort	Sweden	1	0	0	1	0	0
328274	Ischemic heart disease	Chen	1997	Prospective cohort	China	1	0	0	1	0	1
328460	Ischemic heart disease	Kono	1985	Prospective cohort	Japan	1	1	0	1	0	1
328472	Ischemic heart disease	Yuan	1996	Prospective cohort	China	1	0	0	1	0	1
330932	Ischemic heart disease	Lam	2007	Prospective cohort	China	0	0	0	1	1	0
331705	Ischemic heart disease	Lawlor	2008	Prospective cohort	Republic of Korea	0	0	0	1	0	0
332104	Ischemic heart disease	Shaper	2003	Prospective cohort	United Kingdom	0	0	0	1	0	1
334410	Ischemic heart disease	Kuller	1991	Prospective cohort	United States	1	0	0	1	0	0
335266	Ischemic heart disease	Molshatzki	2013	Prospective cohort	Israel	0	0	0	1	0	0
335762	Ischemic heart disease	Zhang	2011	Prospective cohort	Germany	0	0	0	1	0	0
336216	Ischemic heart disease	Thun	2013	Pooled prospective cohort	United States	1	0	0	1	1	0
336319	Ischemic heart disease	Jamrozik	2011	Pooled prospective cohort	Australia	0	0	0	1	1	0
336682	Ischemic heart disease	Jonsdottir	2002	Prospective cohort	Iceland	0	0	0	1	0	0
336801	Ischemic heart disease	Woodward	2005	Pooled prospective cohort	Multi-country	1	1	1	1	0	1
343310	Ischemic heart disease	Prescott	2002	Prospective cohort	Denmark	0	0	0	1	0	0
343312	Ischemic heart disease	Ehteshami-Afshar	2014	Prospective cohort	Iran	0	0	0	1	0	0
343400	Ischemic heart disease	Kawachi	1994	Prospective cohort	United States	1	1	0	0	0	1

343461	Ischemic heart disease	Hirayama	1990	Prospective cohort	Japan	1	1	0	1	0	0
355955	Ischemic heart disease	Doll	1980	Prospective cohort	United Kingdom	1	1	0	0	0	1
355955	Ischemic heart disease	Doll	1980	Prospective cohort	United Kingdom	1	1	0	1	1	0
355955	Ischemic heart disease	Doll	1980	Prospective cohort	United Kingdom	1	1	0	1	0	0
356107	Ischemic heart disease	Lv	2017	Prospective cohort	China	1	0	0	1	0	0
356109	Ischemic heart disease	Watt	1995	Prospective cohort	United Kingdom	1	1	0	1	0	1
356114	Ischemic heart disease	Schnohr	2015	Prospective cohort	Denmark	1	1	1	1	0	0
356141	Ischemic heart disease	Lubin	2016	Prospective cohort	United States	0	0	0	1	0	0
356143	Ischemic heart disease	Lubin	2017	Pooled prospective cohort	Finland, United States	0	0	0	1	0	0
356143	Ischemic heart disease	Lubin	2017	Pooled prospective cohort	Finland, United States	1	1	1	1	0	0
357254	Ischemic heart disease	Hippisley-Cox	2015	Prospective cohort	United Kingdom	1	1	1	1	0	0
357264	Ischemic heart disease	Ciruzzi	2002	Case-control	Argentina	1	1	0	1	1	0
357264	Ischemic heart disease	Ciruzzi	2002	Case-control	Argentina	0	0	0	1	1	0
357274	Ischemic heart disease	Dunn	1999	Case-control	United Kingdom	1	1	0	1	0	1
357353	Ischemic heart disease	Gramenzi	1989	Case-control	Italy	0	0	0	1	0	1
357373	Ischemic heart disease	Ismail	2004	Case-control	Pakistan	1	1	0	1	0	1
357410	Ischemic heart disease	Kabagambe	2007	Case-control	Costa Rica	0	0	0	1	0	1
357413	Ischemic heart disease	Miyake	2000	Case-control	Japan	0	0	0	1	0	1
357413	Ischemic heart disease	Miyake	2000	Case-control	Japan	0	0	0	1	1	1
357427	Ischemic heart disease	Oliveira	2007	Case-control	Portugal	0	0	0	1	0	1
357429	Ischemic heart disease	Oliveira	2009	Case-control	Portugal	0	0	0	1	0	1
357441	Ischemic heart disease	Pais	1996	Case-control	India	1	1	0	1	0	1
357453	Ischemic heart disease	Piegas	2003	Case-control	Bazil	1	1	0	1	0	1
357456	Ischemic heart disease	Quek	1989	Case-control	Malaysia	1	1	1	1	0	1
357927	Ischemic heart disease	Rastogi	2005	Case-control	India	0	0	0	1	0	1
357948	Ischemic heart disease	Rosenberg	1983	Case-control	United States	1	1	0	1	0	1
357952	Ischemic heart disease	Rosenberg	1999	Case-control	United States	0	0	0	1	0	1
357956	Ischemic heart disease	Rosenberg	2001	Case-control	United States	1	0	0	1	0	1
357959	Ischemic heart disease	Rossi	2011	Case-control	Costa Rica	0	0	0	1	0	1
358054	Ischemic heart disease	Suh	2001	Case-control	Republic of Korea	1	0	0	1	0	1

358066	Ischemic heart disease	Bosetti	1999	Pooled case-control	Italy	0	0	0	1	0	1
359113	Ischemic heart disease	Rosenberg	1985	Case-control	United States	1	1	0	1	0	1
359114	Ischemic heart disease	Willett	1981	Case-control	United States	1	1	0	1	0	1
359115	Ischemic heart disease	Palmer	1989	Case-control	United States	0	0	0	1	0	1
359320	Ischemic heart disease	Kaufman	1983	Case-control	United States	0	0	0	1	0	1
432350	Ischemic heart disease	Zhu	2019	Prospective cohort	China	0	0	0	1	0	0
462792	Ischemic heart disease	Banks	2019	Prospective cohort	Australia	0	0	0	0	0	1
501896	Ischemic heart disease	Ding	2020	Prospective cohort	United States	0	0	0	1	0	0
502077	Ischemic heart disease	Jee	2019	Prospective cohort	Republic of Korea	1	1	1	1	0	1
502134	Ischemic heart disease	Wilsgaard	2020	Prospective cohort	Norway	0	0	0	1	0	0
164492	Kidney cancer	Flaherty	2005	Prospective cohort	United States	1	0	0	1	0	1
164580	Kidney cancer	Setiawan	2007	Prospective cohort	United States	0	0	0	1	0	0
328339	Kidney cancer	Ozasa	2007	Prospective cohort	Japan	1	0	0	1	0	0
343155	Kidney cancer	Chiu	2001	Case-control	United States	0	0	0	1	0	1
343580	Kidney cancer	Siemiatycki	1995	Case-control	Canada	1	1	1	1	0	0
358807	Kidney cancer	McCredie	1992	Case-control	Australia	1	0	0	1	0	1
358809	Kidney cancer	Schlehofer	1995	Case-control	Germany	1	1	0	1	0	1
358812	Kidney cancer	Muscat	1995	Case-control	United States	1	0	0	1	0	1
358814	Kidney cancer	Semenza	2001	Case-control	United States	1	0	0	1	0	1
413175	Kidney cancer	Hu	2005	Case-control	Canada	0	0	0	1	0	0
502130	Kidney cancer	Mezzoiuso	2021	Prospective cohort	Italy	1	0	0	1	0	0
502132	Kidney cancer	Minami	2021	Prospective cohort	Japan	1	1	1	1	0	0
502217	Kidney cancer	Everatt	2019	Prospective cohort	Lithuana	1	0	0	1	0	0
502427	Kidney cancer	Theis	2008	Case-control	United States	1	0	0	1	0	1
502427	Kidney cancer	Theis	2008	Case-control	United States	1	0	0	1	0	0
502441	Kidney cancer	Mellemgaard	1994	Case-control	Denmark	1	1	1	1	0	0
502448	Kidney cancer	Demirel	2008	Case-control	Russia, Romania, Poland, Czech Republic	1	0	0	1	0	1
502478	Kidney cancer	McLaughlin	1995	Case-control	Australia, Denmark, Germany, Sweden, United States	1	0	0	1	0	1
502480	Kidney cancer	McLaughlin	1992	Case-control	China	1	0	0	1	0	1

298142	Laryngeal cancer	Dosemeci	1997	Case-control	Turkey	1	0	0	1	0	1
298147	Laryngeal cancer	Zheng	1992	Case-control	China	1	0	0	1	0	1
343375	Laryngeal cancer	Schlecht	1999	Case-control	Brazil	0	0	0	1	0	1
364113	Laryngeal cancer	Lubin	2010	Pooled case-control	Multi-country	0	0	0	1	0	1
502130	Laryngeal cancer	Mezzoiuso	2021	Prospective cohort	Italy	1	0	0	1	0	0
328339	Leukemia	Ozasa	2007	Prospective cohort	Japan	1	0	0	1	1	0
343390	Leukemia	Ugai	2017	Prospective cohort	Japan	1	0	0	1	0	0
343390	Leukemia	Ugai	2017	Prospective cohort	Japan	1	1	0	1	0	0
502130	Leukemia	Mezzoiuso	2021	Prospective cohort	Italy	1	0	0	1	0	0
502184	Leukemia	Viner	2019	Prospective cohort	Canada	0	0	0	1	0	1
502395	Leukemia	Björk	2009	Case-control	Sweden	1	0	0	1	0	1
502397	Leukemia	Wong	2009	Case-control	China	1	1	0	1	0	1
502419	Leukemia	Kasim	2005	Case-control	Canada	1	0	0	1	0	0
286841	Lip and oral cavity cancer	Muwonge R	2008	case-control	India	1	1	1	1	0	0
343375	Lip and oral cavity cancer	Schlecht	1999	Case-control	Brazil	0	0	0	0	0	0
343432	Lip and oral cavity cancer	Zheng T	1997	case-control	China	1	0	0	1	0	1
348052	Lip and oral cavity cancer	Macfarlane	1995	Pooled case-control	China, United States, Italy	0	0	0	1	0	1
359126	Lip and oral cavity cancer	Fu	2013	Case-control	China	1	0	0	1	0	1
359128	Lip and oral cavity cancer	Lu	2018	Prospective cohort	Japan	0	0	0	1	0	0
364113	Lip and oral cavity cancer	Lubin	2010	Pooled case-control	Italy, Switzerland, Slovakia, Romania, Hungary, Poland, Russia, United States, Puerto Rico, Argentina, Cuba, Brazil, Spain, Ireland, Canada, Australia, India, Sudan, South Sudan	0	0	0	1	0	1
502130	Lip and oral cavity cancer	Angelo Giosuè Mezzoiuso	2021	Prospective cohort	Italy	1	0	0	1	0	0
502243	Lip and oral cavity cancer	Isabela Firigato	2022	case-control	Brazil	1	0	0	1	0	1
502352	Lip and oral cavity cancer	Su, WZ	1998	case-control	China	1	0	0	1	0	0
125938	Liver cancer	Marrero	2005	Case-control	United States	1	0	0	1	0	1
293871	Liver cancer	Shin	1996	Case-control	Republic of Korea	1	0	0	1	0	1

328266	Liver cancer	Liaw	1998	Prospectve cohort	Taiwan	1	1	1	1	0	0
328339	Liver cancer	Ozasa	2007	Prospectve cohort	Japan	1	0	0	1	0	0
343515	Liver cancer	Austin	1986	Case-control	United States	1	0	0	1	0	1
343580	Liver cancer	Siemiatycki	1995	Case-control	Canada	0	0	0	1	0	1
343580	Liver cancer	Siemiatycki	1995	Case-control	Canada	1	1	1	1	0	0
343586	Liver cancer	Matsuo	2003	Case-control	Japan	1	0	0	1	0	1
343588	Liver cancer	Munaka	2003	Case-control	Japan	1	1	0	1	0	1
343590	Liver cancer	Gelatti	2005	Case-control	Italy	0	0	0	1	0	1
343632	Liver cancer	Tanaka	1995	Pooled case-control	Japan	1	1	0	1	0	1
502130	Liver cancer	Mezzoiuso	2021	Prospective cohort	Italy	1	0	0	1	0	0
259680	Lower back pain	Mustard	2005	Prospective cohort	Canada	1	1	1	1	0	1
348202	Lower back pain	Tubach	2002	Prospective cohort	France	1	1	1	1	0	1
348204	Lower back pain	Feldman	1999	Prospective cohort	Canada	0	0	0	1	0	1
348206	Lower back pain	Mikkonen	2008	Prospective cohort	Finland	1	1	1	1	0	1
348208	Lower back pain	Hestbaek	2006	Prospective cohort	Denmark	1	0	0	1	0	1
409803	Lower back pain	Ryden	1989	Case-control	United States	1	1	0	1	0	1
173863	Lower respiratory infection	Doll	1994	Prospective cohort	United Kingdom	1	0	0	0	0	1
236197	Lower respiratory infection	Pirie	2013	Prospective cohort	United Kingdom	1	1	1	1	0	1
298305	Lower respiratory infection	Baik	2000	Prospective cohort	United States	1	0	0	1	0	0
355108	Lower respiratory infection	Nuorti	2000	Case-control	Canada, United States	0	0	0	1	0	1
355112	Lower respiratory infection	Almirall	1999	Case-control	Spain	1	1	1	1	0	1
355955	Lower respiratory infection	Doll	1980	Prospective cohort	United Kingdom	1	1	0	0	0	1
413804	Lower respiratory infection	Hamer	2019	Prospective cohort	United Kingdom	0	0	0	1	0	1
413804	Lower respiratory infection	Hamer	2019	Prospective cohort	United Kingdom, Scotland	0	0	0	1	0	1
413804	Lower respiratory infection	Hamer	2019	Prospective cohort	United Kingdom	0	0	0	1	0	0
413804	Lower respiratory infection	Hamer	2019	Prospective cohort	United Kingdom, Scotland	0	0	0	1	0	0
193948	Lung cancer	Liu	1991	Case-control	China	1	0	0	1	0	1

193987	Lung cancer	Brownson	1987	Case-control	United States	1	1	0	1	0	1
250095	Lung cancer	Marugame	2005	Prospective cohort	Japan	1	0	0	1	0	1
286844	Lung cancer	Dikshit	2000	Case-control	India	1	1	0	1	0	1
298142	Lung cancer	Dosemeci	1997	Case-control	Turkey	1	0	0	1	0	1
321342	Lung cancer	Freedman	2015	Prospective cohort	United States	1	0	0	1	0	1
328215	Lung cancer	Bae	2013	Prospective cohort	Republic of Korea	1	0	0	1	0	1
328266	Lung cancer	Liaw	1998	Prospective cohort	Taiwan	1	1	0	0	0	0
328909	Lung cancer	Everatt	2014	Prospective cohort	Lithuania	1	0	0	1	0	0
334460	Lung cancer	Nordlund	1999	Prospective cohort	Sweden	1	1	0	1	0	0
343580	Lung cancer	Siemiatycki	1995	case-control	Quebec, Canada	0	0	0	1	0	1
343580	Lung cancer	Siemiatycki	1995	case-control	Quebec, Canada	1	1	1	1	0	0
355961	Lung cancer	Chyou	1992	Prospective cohort	United States	1	1	0	1	0	1
355963	Lung cancer	Potter	1991	Nested case-control	United States	1	1	1	1	1	1
355965	Lung cancer	Chyou	1993	Prospective cohort	United States	1	1	0	1	0	1
355970	Lung cancer	Pesch	2012	Pooled case-control	Europe, Canada	1	0	0	1	0	1
357351	Lung cancer	Jockel	1992	Case-control	Germany	1	1	0	1	0	1
357367	Lung cancer	Jockel	1997	Case-control	Germany	1	0	0	1	0	1
357400	Lung cancer	De Stefani	1996	Case-control	Uruguay	1	0	0	1	0	1
357402	Lung cancer	Lei	1996	Case-control	China	1	1	0	1	0	1
357404	Lung cancer	Pawlega	1997	Case-control	Poland	0	0	0	1	0	1
357406	Lung cancer	Wunsch-Filho	1998	Case-control	Brazil	1	1	0	1	0	1
357415	Lung cancer	Mao	2001	Case-control	Canada	1	0	0	1	0	1
357437	Lung cancer	Barbone	1997	Case-control	Italy	1	1	0	1	0	1
357447	Lung cancer	Matos	1998	Case-control	Argentina	1	1	0	1	0	1
357451	Lung cancer	De Stefani	1998	Case-control	Uruguay	1	0	0	1	0	1
357644	Lung cancer	Simonato	2001	Pooled case-control	Sweden, Germany, United Kingdom, France, Spain, Italy	1	0	0	1	0	0
357757	Lung cancer	Risch	1993	Case-control	Canada	1	1	0	1	0	1
357765	Lung cancer	Sankaranarayanan	1994	Case-control	India	1	1	1	1	0	1
357792	Lung cancer	Band	1999	Nested case-control	Canada	1	1	0	1	0	1
357961	Lung cancer	Becher	1991	Case-control	Germany	1	1	0	1	0	1

358161	Lung cancer	Brockmoller	1993	Case-control	Germany	1	1	1	1	0	1
358213	Lung cancer	Vena	1985	Case-control	United States	1	1	0	1	0	1
358338	Lung cancer	Cascorbi	1996	Case-control	Germany	1	1	0	1	0	1
358479	Lung cancer	Chiazze	1992	Nested case-control	United States	1	1	1	1	0	1
358502	Lung cancer	Ando	2003	Prospective cohort	Japan	1	1	0	1	0	0
358552	Lung cancer	De Matteis	2012	Case-control	Italy	1	0	0	1	0	1
358558	Lung cancer	He	2013	Prospective cohort	China	0	0	0	0	1	1
358563	Lung cancer	Nishino	2004	Prospective cohort	Japan	0	0	0	1	0	1
358565	Lung cancer	Papadopoulos	2011	Case-control	France	0	0	0	1	0	1
358577	Lung cancer	Shimazu	2008	Prospective cohort	Japan	0	0	0	1	0	1
358597	Lung cancer	Tindle	2018	Prospective cohort	United States	1	1	1	1	0	1
358717	Lung cancer	Yong	1997	Prospective cohort	United States	1	1	1	1	0	0
358794	Lung cancer	Hansen	2017	Prospective cohort	Norway	1	0	0	1	0	0
359451	Lung cancer	Boffetta	2010	Pooled case-control	United States	1	0	0	1	0	1
413177	Lung cancer	Yun	2015	Prospective cohort	Republic of Korea	0	0	0	1	0	1
419667	Lung cancer	Suzuki	1994	Case-control	Brazil	1	1	1	1	0	1
419669	Lung cancer	De Stefani	1996	Case-control	Uruguay	0	0	0	1	0	1
419671	Lung cancer	Hu	1997	Case-control	China	1	0	0	1	0	1
419675	Lung cancer	Kreuzer	1998	Case-control	Germany	1	0	0	1	1	1
419675	Lung cancer	Kreuzer	1998	Case-control	Germany	1	0	0	1	0	1
419696	Lung cancer	Armadans	1999	Case-control	Spain	1	1	0	1	0	1
419717	Lung cancer	Kubik	2002	Case-control	Czechia	1	0	0	1	0	1
419728	Lung cancer	Rachtan	2001	Case-control	Poland	1	1	0	1	0	1
426275	Lung cancer	Thun	2013	Prospective cohort	United States	1	0	0	1	0	1
426275	Lung cancer	Thun	2013	Prospective cohort	United States	1	0	0	1	0	0
450624	Lung cancer	Zatloukal	2003	case-control	Czech Republic	1	1	0	1	0	1
499241	Lung cancer	Hansen	2021	Prospective Cohort	Norway	1	0	0	0	0	0
502075	Lung cancer	Zhang	2022	Prospective Cohort	United Kingdom	0	0	0	1	0	0
502124	Lung cancer	Weber	2021	Prospective Cohort	Australia	0	0	0	1	0	0
502126	Lung cancer	Guo	2022	Prospective Cohort	China	1	1	1	1	0	0
502130	Lung cancer	Mezzoiuso	2021	Prospective Cohort	Italy	1	0	0	1	0	0
502143	Lung cancer	Hawrysz	2020	case-control	Poland	1	1	0	1	0	0

502147	Lung cancer	Huang	2021	case-control	Taiwan, China	1	1	0	1	0	0
502184	Lung cancer	Viner	2019	Prospective Cohort	Canada	0	0	0	1	0	1
502190	Lung cancer	Park	2021	Prospective Cohort	Republic of Korea	0	0	0	1	0	0
502192	Lung cancer	Jia	2021	Prospective Cohort	United Kingdom	1	1	1	1	0	0
502202	Lung cancer	Rusmaully	2021	case-control	France	1	0	0	1	0	0
502208	Lung cancer	Jin	2019	case-control	China	1	1	1	1	0	1
502213	Lung cancer	Tse	2022	case-control	China	1	1	1	1	0	1
502215	Lung cancer	Huang	2022	case-control	Taiwan, China	1	0	0	1	0	1
502234	Lung cancer	Hosseini	2009	case-control	Iran	0	0	0	1	0	1
502241	Lung cancer	Naghibzadeh -Tahami	2010	case-control	Iran	1	0	0	1	0	1
502246	Lung cancer	Shimatani	2020	case-control	Japan	1	1	1	1	0	1
502252	Lung cancer	Lai	2019	case-control	Taiwan, China	1	0	0	1	0	1
502407	Lung cancer	Schwartz	2015	case-control	Brazil	1	1	1	1	0	1
502407	Lung cancer	Schwartz	2015	case-control	Brazil	1	1	1	1	0	0
502421	Lung cancer	Kreuzer	2003	case-control	Germany	1	1	1	1	0	1
502433	Lung cancer	Sreeja	2005	case-control	India	1	1	0	1	0	0
502458	Lung cancer	Siemiatycki	1994	case-control	Quebec, Canada	1	1	1	1	0	0
502460	Lung cancer	Chan-Yeung	2003	case-control	China	1	1	1	1	0	1
502482	Lung cancer	Lawania	2017	case-control	India	1	1	1	1	1	1
261355	Macular degeneration	Christen	1996	Prospective cohort	United States	0	0	0	1	0	1
359130	Macular degeneration	Seddon	1996	Prospective cohort	United States	0	0	0	1	0	1
221241	Multiple sclerosis	Asadollahi	2013	Case-control	Iran	0	0	0	1	0	0
348023	Multiple sclerosis	Hedstrom	2013	Pooled case-control	Sweden	0	0	0	1	0	1
350715	Multiple sclerosis	Thorogood	1998	Prospective cohort	United Kingdom	1	1	1	1	1	1
350717	Multiple sclerosis	Villard-Mackintosh	1993	Prospective cohort	United Kingdom	1	0	0	1	0	1
369019	Multiple sclerosis	Ghadirian	2001	Case-control	Canada	1	0	0	1	0	1
369022	Multiple sclerosis	Jafari	2009	Case-control	Netherlands	1	1	0	1	0	1
328266	Nasopharyngeal cancer	Liaw	1998	Prospective cohort	Taiwan	1	0	0	0	0	1
328921	Nasopharyngeal cancer	Lin	2015	Prospective cohort	China	0	0	0	1	0	1
328952	Nasopharyngeal cancer	Hsu	2009	Prospective cohort	Taiwan	1	1	1	1	0	0
346030	Nasopharyngeal cancer	Nam	1992	Case-control	United States	1	1	1	1	0	1

346032	Nasopharyngeal cancer	Vaughan	1996	Case-control	United States	1	0	0	1	0	1
346040	Nasopharyngeal cancer	Zhu	1995	Case-control	United States	0	0	0	1	0	1
346047	Nasopharyngeal cancer	Cheng	1999	Case-control	Taiwan	0	0	0	1	0	1
346049	Nasopharyngeal cancer	Yuan	2000	Case-control	China	0	0	0	1	0	1
346056	Nasopharyngeal cancer	Ji	2011	Case-control	China	1	0	0	1	0	1
346071	Nasopharyngeal cancer	Fachiroh	2012	Case-control	Thailand	1	0	0	1	0	1
373803	Nasopharyngeal cancer	Chang	2017	Case-control	China	0	0	0	1	0	1
502223	Nasopharyngeal cancer	Hsu	2020	Case-control	Taiwan	1	0	0	1	0	1
343375	Other pharynx cancer	Schlecht	1999	Case-control	Brazil	0	0	0	0	0	0
343434	Other pharynx cancer	Zavras	2001	Case-control	Greece	1	0	0	1	0	1
343450	Other pharynx cancer	De Stefani	1998	Case-control	Uruguay	0	0	0	1	0	1
359128	Other pharynx cancer	Lu	2018	Prospective cohort	Japan	1	0	0	1	0	0
364113	Other pharynx cancer	Lubin	2010	Pooled case-control	Italy, Switzerland, Slovakia, Romania, Hungary, Poland, Russia, United States, Puerto Rico, Argentina, Cuba, Brazil, Spain, Ireland, Canada, Australia, India, Sudan, South Sudan	0	0	0	1	0	1
439809	Other pharynx cancer	Zeng	2019	Case-control	China	1	1	1	1	0	1
439813	Other pharynx cancer	Yamashita	2019	Case-control	Japan	1	1	1	1	0	1
502194	Other pharynx cancer	Shewale	2021	Case-control	United States	1	1	1	1	0	0
164536	Pancreatic cancer	Larsson	2005	Prospective cohort	Sweden	0	0	0	1	0	0
164547	Pancreatic cancer	Luo	2007	Prospective cohort	Japan	0	0	0	1	0	0
346137	Pancreatic cancer	Inoue	2003	Nested case-control	Japan	0	0	0	1	0	1
346826	Pancreatic cancer	Fuchs	1996	Prospective cohort	United States	1	0	0	0	0	1
346828	Pancreatic cancer	Harnack	1997	Prospective cohort	United States	1	1	0	1	1	1
346832	Pancreatic cancer	Nilson	2000	Prospective cohort	Norway	1	1	0	1	0	0
346870	Pancreatic cancer	Ji	1995	Case-control	China	1	0	0	1	0	1
346876	Pancreatic cancer	Lin	2002	Prospective cohort	Japan	1	0	0	1	0	0

347176	Pancreatic cancer	Silverman	1994	Case-control	United States	0	0	0	1	0	1
359135	Pancreatic cancer	Lin	2013	Prospective cohort	Japan	1	0	0	1	0	0
359138	Pancreatic cancer	Vrieling	2010	Prospective cohort	Denmark, France, Germany, Greece, Italy, Netherlands, Norway, Spain, Sweden, United Kingdom	0	0	0	1	0	0
359139	Pancreatic cancer	Lee	1996	Case-control	Taiwan	1	1	0	1	0	1
359140	Pancreatic cancer	Kuzmickiene	2013	Prospective cohort	Lithuania	1	0	0	1	0	0
502128	Pancreatic cancer	Ahmad Naghibzadeh -Tahami	2021	case-control	Iran	0	0	0	1	0	1
502130	Pancreatic cancer	Angelo Giosuè Mezzoiuso	2021	Prospective cohort	Italy	1	0	0	1	0	0
502188	Pancreatic cancer	Esther Molina-Montes	2020	case-control	Europe	1	0	0	1	0	1
502220	Pancreatic cancer	Brian Z Huang	2019	Prospective cohort	United States	0	0	0	1	0	0
502474	Pancreatic cancer	Yuriko N Koyanagi	2019	Prospective cohort	Japan	1	0	0	1	0	1
502474	Pancreatic cancer	Yuriko N Koyanagi	2019	Prospective cohort	Japan	0	0	0	1	0	1
173863	Parkinson's disease	Doll	1994	Prospective cohort	United Kingdom	1	0	0	0	0	1
355955	Parkinson's disease	Doll	1980	Prospective cohort	United Kingdom	1	1	0	0	0	1
359151	Parkinson's disease	Benedetti	2000	Case-control	United States	1	1	1	1	0	0
359152	Parkinson's disease	Hernan	2001	Pooled prospective cohort	United States	1	1	1	0	0	1
359154	Parkinson's disease	Van der Mark	2014	Case-control	Netherlands	1	1	1	1	0	0
359157	Parkinson's disease	Chen	2010	Case-control	United States	1	0	0	1	0	1
359158	Parkinson's disease	Driver	2009	Prospective cohort	United States	1	1	1	1	0	0
359159	Parkinson's disease	Thacker	2007	Prospective cohort	United States	1	1	0	1	1	0
359160	Parkinson's disease	Paganini-Hill	2001	Nested case-control	United States	1	1	1	1	1	0
359161	Parkinson's disease	Rajput	1987	Case-control	United States	1	1	1	1	0	0
359162	Parkinson's disease	Jimenez-Jimenez	1992	Case-control	Spain	1	1	1	1	1	0
359164	Parkinson's disease	Morano	1994	Case-control	Spain	1	1	1	1	1	0
359307	Parkinson's disease	Sasco	1990	Nested case-control	United States	1	1	1	1	1	0
502009	Parkinson's disease	Gallo	2019	Prospective cohort	Sweden, United Kingdom, Germany,	1	0	0	1	1	0

					Spain, Italy, Greece						
262563	Peptic ulcer	Anda	1990	Prospective cohort	United States	0	0	0	1	0	0
328259	Peptic ulcer	Nilsson	2001	Prospective cohort	Sweden	1	1	0	1	0	0
338384	Peptic ulcer	Rosenstock	2003	Prospective cohort	Denmark	0	0	0	1	0	0
349377	Peptic ulcer	Wang	1996	Case-control	China	1	0	0	1	0	1
349381	Peptic ulcer	Aldoori	1997	Prospective cohort	United States	0	0	0	1	0	1
349542	Peptic ulcer	Al-Zubeer	2012	Case-control	Iraq	1	1	1	1	0	1
359166	Peptic ulcer	Deding	2016	Prospective cohort	Denmark	0	0	0	1	0	0
350659	Peripheral artery disease	Conen	2011	Prospective cohort	United States	0	0	0	1	0	1
359221	Peripheral artery disease	St-Pierre	2010	Prospective cohort	Canada	0	0	0	1	0	0
359223	Peripheral artery disease	Lakshmanan	2010	Prospective cohort	Australia	1	0	0	1	1	0
369290	Peripheral artery disease	Skalkidis	1989	Case-control	Greece	0	0	0	1	0	1
462792	Peripheral artery disease	Banks	2019	Cohort	Australia	0	0	0	1	0	0
501896	Peripheral artery disease	Ding	2019	Cohort	United States	0	0	0	1	0	0
261500	Rheumatoid Arthritis	Costenbader	2006	Prospective cohort	United States	0	0	0	1	0	1
261510	Rheumatoid Arthritis	Heliovaara	1993	Prospective cohort	Finland	1	1	0	1	0	1
350653	Rheumatoid Arthritis	Di Giuseppe	2013	Prospective cohort	Sweden	1	1	1	1	1	1
353080	Rheumatoid Arthritis	Fisher	2015	Nested case-control	Spain, Italy	1	1	1	1	0	1
502082	Rheumatoid Arthritis	Jinma Ren	2020	case-control	United States	1	1	1	1	0	0
502084	Rheumatoid Arthritis	Xinyi Liu	2019	Prospective Cohort	United States	0	0	0	0	1	0
309835	Stomach cancer	Gammon	1997	Case-control	United States	0	0	0	0	0	0
328266	Stomach cancer	Liaw	1998	Prospective cohort	Taiwan	1	0	0	0	0	0
339695	Stomach cancer	Kneller	1991	Prospective cohort	United States	1	1	0	1	0	1
339697	Stomach cancer	Ji	1996	Case-control	China	1	0	0	1	0	0
340513	Stomach cancer	De Stefani	1998	Case-control	Uruguay	0	0	0	1	0	1
340528	Stomach cancer	Zaridze	2000	Case-control	Russia	1	0	0	1	0	1
340554	Stomach cancer	Hoshiyama	1992	Case-control	Japan	1	0	0	1	0	1
340578	Stomach cancer	Guo	1994	Nested case-control	China	1	0	0	1	0	1
340582	Stomach cancer	Chao	2002	Prospective cohort	United States	1	1	0	1	0	0
340582	Stomach cancer	Chao	2002	Prospective cohort	United States	0	0	0	1	0	0
343580	Stomach cancer	Siemiatycki	1995	Case-control	Canada	1	1	1	1	0	0
347810	Stomach cancer	Fujino	2005	Prospective cohort	Japan	1	1	0	1	0	0

347810	Stomach cancer	Fujino	2005	Prospective cohort	Japan	0	0	0	1	0	0
502130	Stomach cancer	Mezzoiuso	2021	Prospective cohort	Italy	1	0	0	1	0	0
502141	Stomach cancer	Zhang	2021	Case-control	China	1	1	0	1	0	0
122237	Stroke	Okada	1976	Prospective cohort	Japan	1	1	1	1	0	1
155207	Stroke	Chiuve	2008	Prospective cohort	United States	1	0	0	0	0	1
165539	Stroke	Jacobs	1999	Prospective cohort	United States, Finland, Netherlands, Italy, Croatia, Serbia, Greece, Japan	1	0	0	1	0	1
174238	Stroke	Doll	2004	Prospective cohort	United Kingdom	1	0	0	0	0	1
193978	Stroke	Yamada	2003	Prospective cohort	Japan	1	1	0	0	0	0
298316	Stroke	Longstreth	1992	Case-control	United States	1	0	0	1	0	1
324092	Stroke	Klatsky	2002	Prospective cohort	United States	0	0	0	1	0	0
328259	Stroke	Nilsson	2001	Prospective cohort	Sweden	1	0	0	1	0	0
328460	Stroke	Kono	1985	Prospective cohort	Japan	1	1	0	1	0	1
330677	Stroke	Kelly	2008	Prospective cohort	China	0	0	0	1	0	0
330932	Stroke	Lam	2007	Prospective cohort	China	0	0	0	1	1	0
331371	Stroke	Iso	2005	Prospective cohort	Japan	0	0	0	1	0	0
331705	Stroke	Lawlor	2008	Prospective cohort	Republic of Korea	0	0	0	1	0	1
331710	Stroke	Ueshima	2004	Prospective cohort	Japan	0	0	0	1	0	0
332100	Stroke	Mannami	2004	Prospective cohort	Japan	0	0	0	1	0	1
332100	Stroke	Mannami	2004	Prospective cohort	Japan	0	0	0	1	0	0
332104	Stroke	Shaper	2003	Prospective cohort	United Kingdom	0	0	0	1	0	0
334028	Stroke	Kawachi	1993	Prospective cohort	United States	0	0	0	0	0	1
334410	Stroke	Kuller	1991	Prospective cohort	United States	1	1	0	1	0	0
334731	Stroke	Hart	1999	Prospective cohort	United Kingdom	1	1	0	1	0	0
334814	Stroke	Kondo	2011	Prospective cohort	Japan	1	0	0	1	0	0
334967	Stroke	Fuller	1983	Prospective cohort	United Kingdom	1	1	0	1	0	0
335266	Stroke	Molshatzki	2013	Prospective cohort	Israel	1	1	0	1	0	0
335914	Stroke	Honjo	2009	Pooled prospective cohort	Japan	1	0	0	1	0	0
336216	Stroke	Thun	2013	Pooled prospective cohort	United States	1	1	1	1	0	1
336216	Stroke	Thun	2013	Pooled prospective cohort	United States	1	0	0	1	0	0
336216	Stroke	Thun	2013	Pooled prospective cohort	United States	1	0	0	0	0	1

336216	Stroke	Thun	2013	Pooled prospective cohort	United States	1	0	0	0	0	0
336319	Stroke	Jamrozik	2018	Pooled prospective cohort	Australia	0	0	0	1	1	0
336319	Stroke	Jamrozik	2018	Pooled prospective cohort	Australia	0	0	0	1	1	1
336801	Stroke	Woodward	2005	Pooled prospective cohort	Multi-country	1	1	1	1	0	1
343297	Stroke	Shaper	1991	Prospective cohort	United Kingdom	1	1	0	1	0	0
343300	Stroke	Anderson	2004	Case-control	Australia, New Zealand	0	0	0	1	0	1
343306	Stroke	Kenfield	2008	Prospective cohort	United States	1	1	0	1	0	1
343582	Stroke	Lam	2001	Case-control	China	1	0	0	1	1	0
343582	Stroke	Lam	2001	Case-control	China	1	0	0	1	0	0
343647	Stroke	Doll	1976	Prospective cohort	United Kingdom	1	1	0	1	1	0
343647	Stroke	Doll	1976	Prospective cohort	United Kingdom	1	1	0	1	0	0
344315	Stroke	Bhat	2008	Case-control	United States	0	0	0	1	0	0
344318	Stroke	Kurth	2003	Prospective cohort	United States	1	0	0	1	0	1
344318	Stroke	Kurth	2003	Prospective cohort	United States	0	0	0	1	0	1
344340	Stroke	Broderick	2003	Case-control	United States	1	0	0	1	0	1
344340	Stroke	Broderick	2003	Case-control	United States	1	1	1	1	0	0
344343	Stroke	Wang	2001	Prospective cohort	China	1	1	1	1	1	1
344345	Stroke	Prescott	1998	Pooled prospective cohort	Denmark	1	0	0	1	0	0
344359	Stroke	Colditz	1988	Prospective cohort	United States	0	0	0	1	0	0
344360	Stroke	Wolf	1988	Prospective cohort	United States	1	0	0	1	0	0
344362	Stroke	Ostfeld	1974	Prospective cohort	United States	1	1	1	1	1	1
350743	Stroke	Bonita	1986	Case-control	New Zealand	1	1	0	1	0	1
350743	Stroke	Bonita	1986	Case-control	New Zealand	1	1	0	1	0	0
350747	Stroke	Yamaura	2001	Case-control	Japan	1	1	1	1	0	0
350747	Stroke	Yamaura	2001	Case-control	Japan	1	1	1	1	0	1
350749	Stroke	Kurth	2003	Prospective cohort	United States	1	0	0	0	0	1
350753	Stroke	Lindbohm	2016	Prospective cohort	Finland	0	0	0	1	0	1
350757	Stroke	Xu	2013	Prospective cohort	China	0	0	0	1	1	0
356591	Stroke	Hippisley-Cox	2013	Prospective cohort	United Kingdom	0	0	0	1	0	1
357417	Stroke	Bonita	1999	Case-control	New Zealand	1	1	0	1	0	0
357431	Stroke	Gill	1989	Case-control	United Kingdom	0	0	0	1	0	1
357435	Stroke	Hajat	2004	Case-control	United Kingdom	1	0	0	1	0	1

357445	Stroke	Jamrozik	1994	Case-control	Australia	1	0	0	1	0	1
358462	Stroke	Thrift	1999	Case-control	Australia	0	0	0	1	0	1
358466	Stroke	Markidan	2018	Case-control	United States	0	0	0	1	0	0
358470	Stroke	Song	2005	Case-control	Republic of Korea	1	1	1	1	0	0
358476	Stroke	Juvela	1993	Case-control	Finland	1	0	0	1	0	1
358506	Stroke	Bonita	1986	Case-control	New Zealand	1	1	1	1	0	0
358540	Stroke	Hannaford	1994	Nested case-control	United Kingdom	1	0	0	1	0	1
358652	Stroke	Carrieri	1994	Case-control	Italy	0	0	0	1	0	1
412060	Stroke	Ueshima	2004	Prospective cohort	Japan	0	0	0	1	0	0
429295	Stroke	Lu	2008	Prospective cohort	Sweden	0	0	0	1	0	0
432350	Stroke	Zhu	2019	Prospective cohort	China	0	0	0	1	0	0
501860	Stroke	Rautalin	2020	Prospective cohort	Finland	1	1	1	1	1	0
501896	Stroke	Ding	2019	Prospective cohort	United States	0	0	0	1	0	0
501942	Stroke	Sato	2021	Prospective cohort	Japan	0	0	0	1	1	0
501943	Stroke	Zhang	2021	Prospective cohort	United Kingdom	0	0	0	1	0	0
502077	Stroke	Jee	2019	Prospective cohort	Republic of Korea	1	1	1	1	0	1
502248	Stroke	Peters	2020	Prospective cohort	United Kingdom	0	0	0	1	0	1
502250	Stroke	Oshunbade	2020	Prospective cohort	United States	1	0	0	1	0	0
173863	Tuberculosis	Doll	1994	Prospective cohort	United Kingdom	1	0	0	0	0	1
236195	Tuberculosis	Liu	1998	Case-control	China	1	1	1	1	0	0
298335	Tuberculosis	Crampin	2004	Case-control	Malawi	1	0	0	1	0	1
298340	Tuberculosis	Buskin	1994	Case-control	United States	1	1	1	1	0	1
327906	Tuberculosis	Wen	2010	Prospective cohort	Taiwan	0	0	0	1	0	1
327949	Tuberculosis	Lin	2009	Prospective cohort	Taiwan	0	0	0	1	0	0
327956	Tuberculosis	Jee	2009	Prospective cohort	Republic of Korea	1	0	0	1	0	0
343383	Tuberculosis	Anderson	1997	Case-control	United States	1	0	0	1	0	1
343582	Tuberculosis	Lam	2001	Case-control	China	1	0	0	1	0	1
343582	Tuberculosis	Lam	2001	Case-control	China	1	0	0	1	1	1
348044	Tuberculosis	Ariyothai	2004	Case-control	Thailand	1	1	1	1	0	1
348046	Tuberculosis	Kolappan	2002	Nested case-control	India	1	1	1	1	0	1
348053	Tuberculosis	Leung	2004	Prospective cohort	China	0	0	0	1	1	1
359225	Tuberculosis	Dhamgaye	2008	Case-control	India	1	1	0	1	0	1

359228	Tuberculosis	Marahatta	2015	Case-control	Nepal	1	1	1	1	0	1
359230	Tuberculosis	Pednekar	2007	Prospective cohort	India	1	0	0	1	0	0
359273	Tuberculosis	Smith	2015	Nested case-control	United States	1	0	0	0	0	1
369826	Tuberculosis	Alavi	2012	Case-control	Iran	0	0	0	1	1	1
501926	Tuberculosis	Li	2021	Prospective cohort study	China	0	0	0	0	0	1
501936	Tuberculosis	Tewatia	2020	Case-control	India	1	1	0	1	0	1
230548	Type 2 diabetes	Sawada	2003	Prospective cohort	Japan	0	0	0	1	0	1
236197	Type 2 diabetes	Pirie	2013	Prospective cohort	United Kingdom	1	1	1	1	0	1
255434	Type 2 diabetes	Doi	2012	Prospective cohort	Japan	0	0	0	1	0	0
255463	Type 2 diabetes	Waki	2005	Prospective cohort	Japan	0	0	0	1	0	0
309584	Type 2 diabetes	Teratini	2012	Prospective cohort	Japan	0	0	0	1	0	1
309588	Type 2 diabetes	Kawakami	1997	Prospective cohort	Japan	0	0	0	1	0	1
309592	Type 2 diabetes	Hu	2001	Prospective cohort	United States	0	0	0	0	0	1
309610	Type 2 diabetes	Jee	2010	Prospective cohort	Republic of Korea	0	0	0	1	0	1
348079	Type 2 diabetes	Rimm	1995	Prospective cohort	United States	0	0	0	1	0	1
348081	Type 2 diabetes	Uchimoto	1999	Prospective cohort	Japan	0	0	0	1	0	1
348083	Type 2 diabetes	Manson	2000	Prospective cohort	United States	0	0	0	1	0	1
348085	Type 2 diabetes	Nakanishi	2000	Prospective cohort	Japan	0	0	0	1	0	1
348088	Type 2 diabetes	Wannamethe e	2001	Prospective cohort	England, United Kingdom	1	0	0	1	0	1
348091	Type 2 diabetes	Carlsson	2004	Prospective cohort	Norway	1	0	0	1	0	0
348100	Type 2 diabetes	Sairenchi	2004	Prospective cohort	Japan	0	0	0	1	0	0
348105	Type 2 diabetes	Patja	2005	Prospective cohort	Finland	0	0	0	1	0	0
348108	Type 2 diabetes	Meisinger	2006	Prospective cohort	Germany	0	0	0	1	0	0
348112	Type 2 diabetes	Park	2008	Retrospective cohort	Republic of Korea	1	1	0	1	0	1
356075	Type 2 diabetes	Will	2001	Prospective cohort	United States	0	0	0	1	0	0
356095	Type 2 diabetes	Akter	2015	Prospective cohort	Japan	0	0	0	1	0	1
356097	Type 2 diabetes	Hilawe	2015	Prospective cohort	Japan	0	0	0	1	0	1
356099	Type 2 diabetes	Hou	2016	Case-control	China	0	0	0	1	0	0
356101	Type 2 diabetes	Rasouli	2016	Case-control	Sweden	1	0	0	1	0	1
356103	Type 2 diabetes	Lv	2017	Prospective cohort	China	0	0	0	1	0	0
356105	Type 2 diabetes	Consortium	2014	Case-cohort	Denmark, France, Germany, Italy,	0	0	0	1	0	0

					Netherlands, Spain, Sweden, United Kingdom						
413800	Type 2 diabetes	Radzevicien e	2018	Case-control	Lithuania	1	0	0	1	0	1
502053	Type 2 diabetes	Huh	2022	Prospective cohort	Republic of Korea	0	0	0	1	0	0
502177	Type 2 diabetes	White	2018	Prospective cohort	United States	0	0	0	1	0	0

Section 4: The dose-response RR curves and their 95% uncertainty interval for all smoking-outcome pairs

Section 4.1: Five-star smoking-outcome pairs

Laryngeal cancer

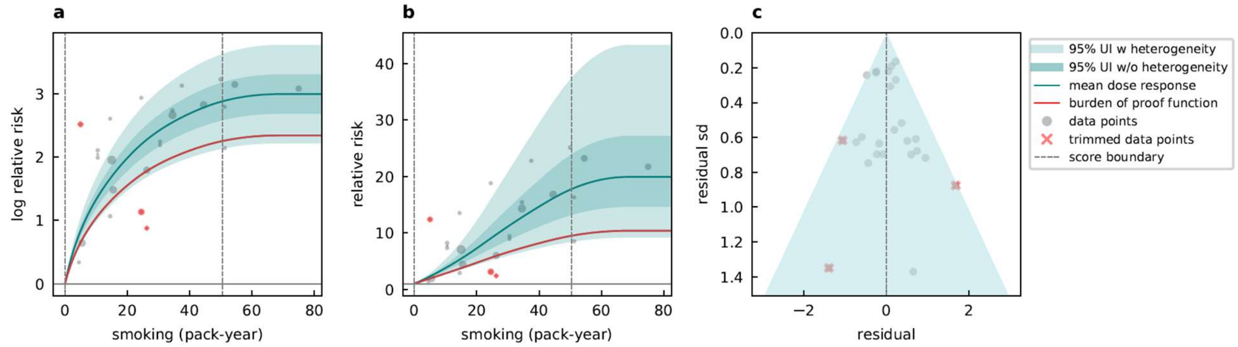

Aortic aneurysm (reference age group: 55-59)

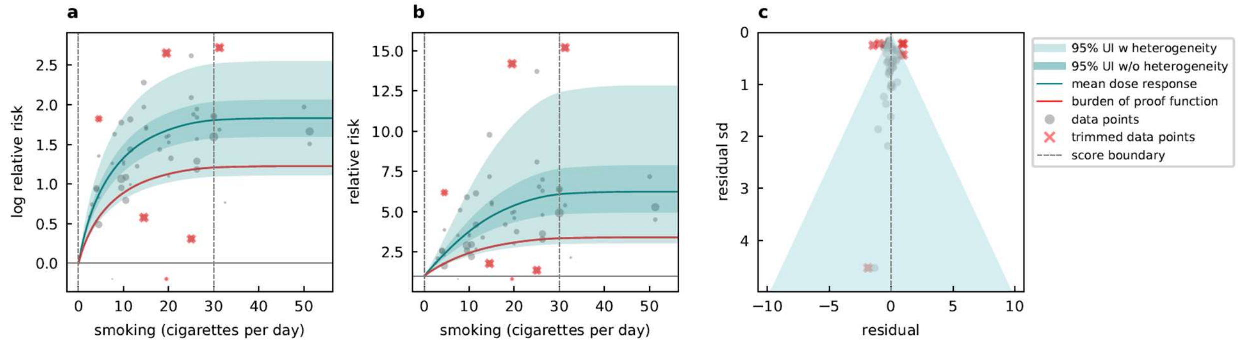

Peripheral artery disease (reference age group: 60-64)

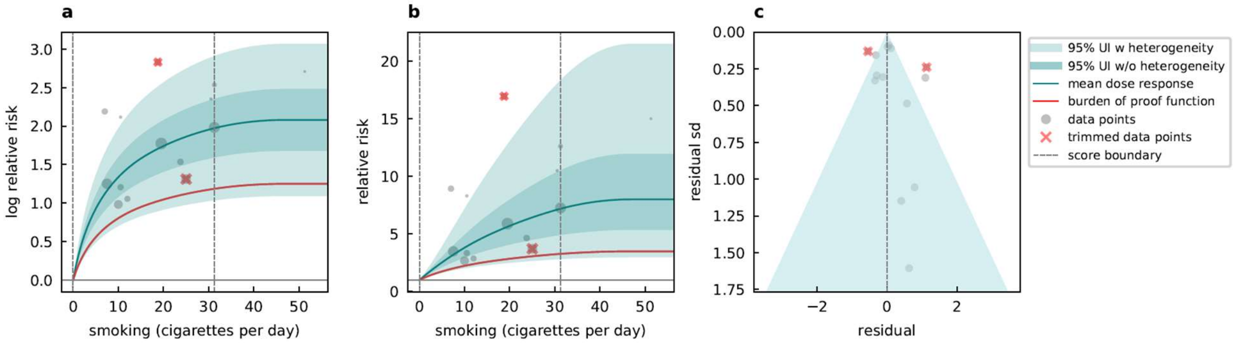

Lung cancer

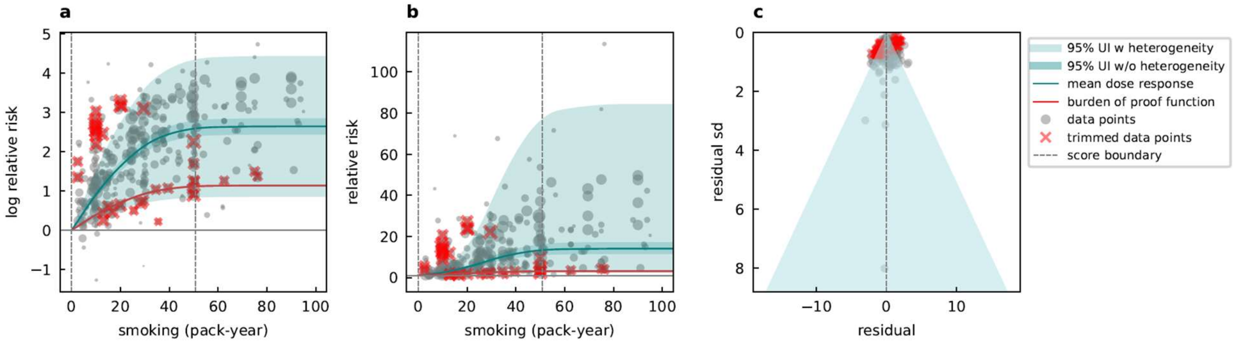

Other pharynx cancer

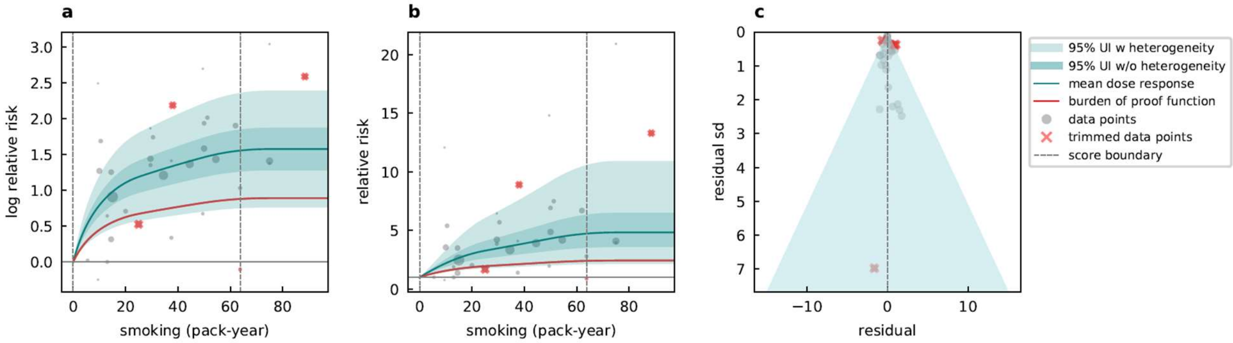

Section 4.2: Four-star smoking-outcome pairs

Pancreatic cancer

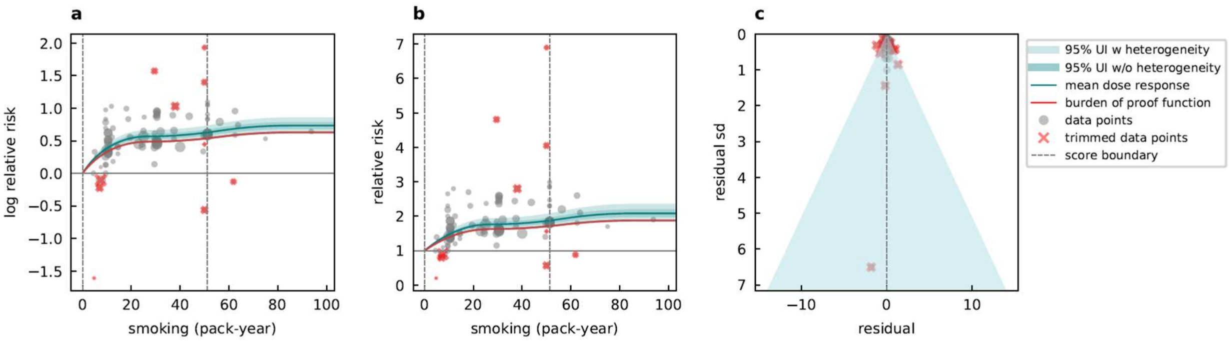

COPD

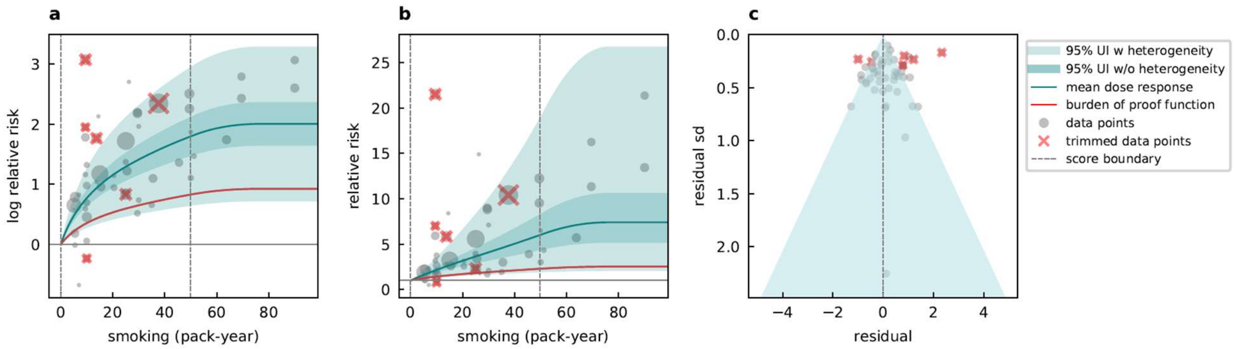

Lower respiratory infection

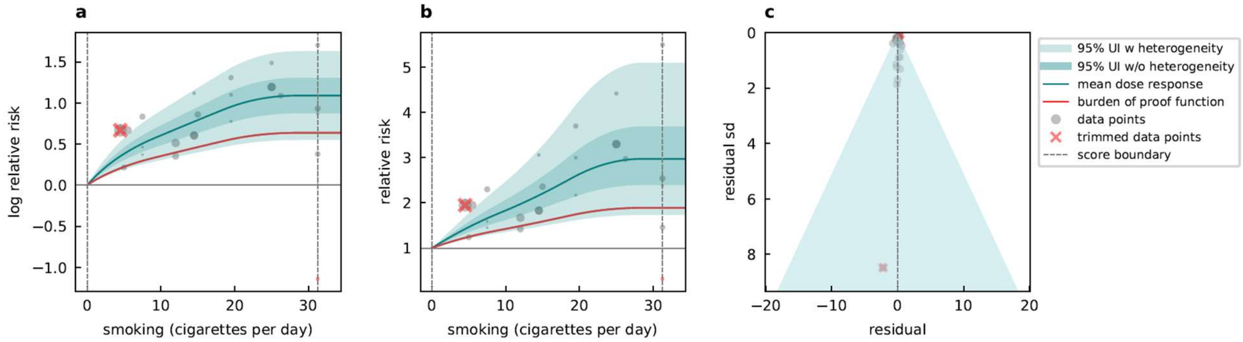

Section 4.3: Three-star smoking-outcome pairs

Bladder cancer

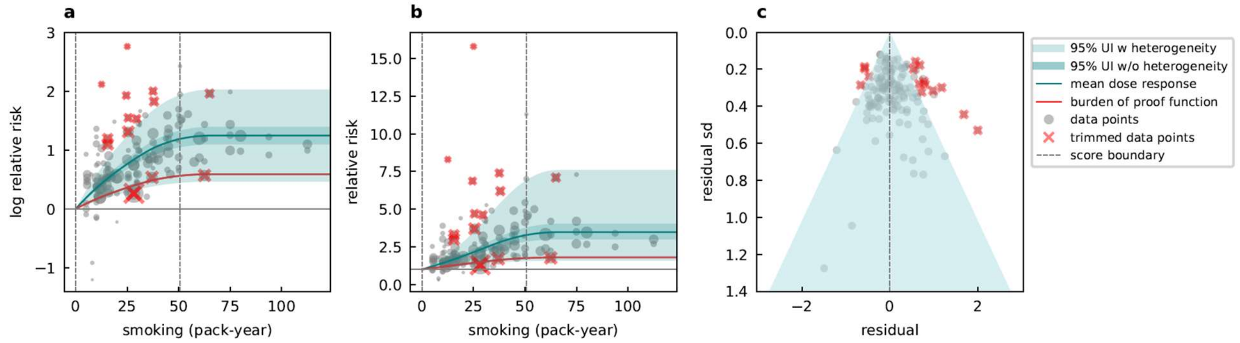

Stomach cancer

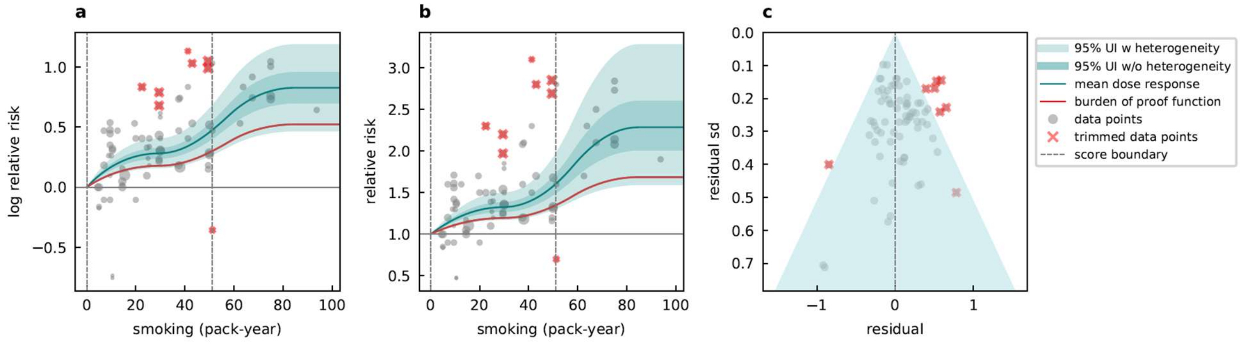

Diabetes

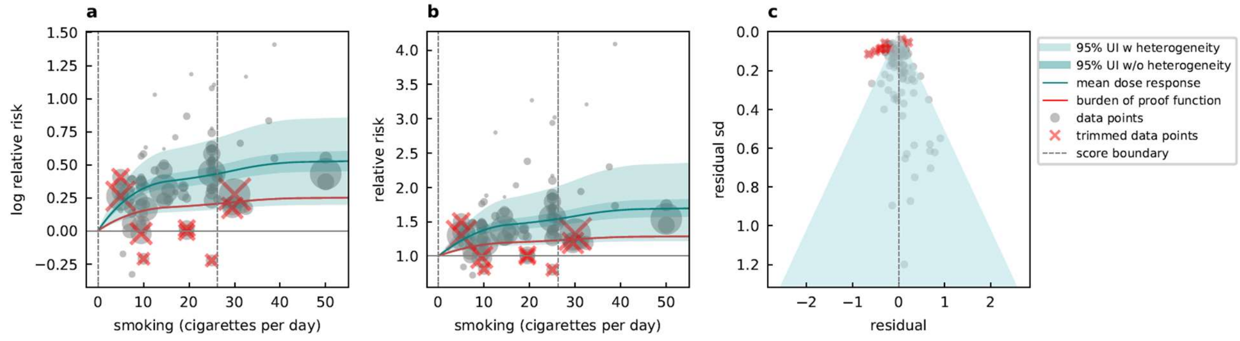

Tuberculosis

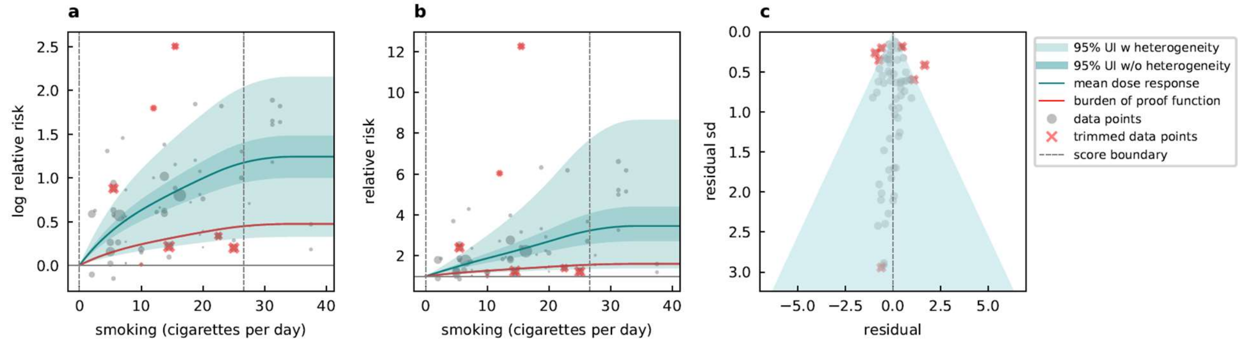

Multiple sclerosis

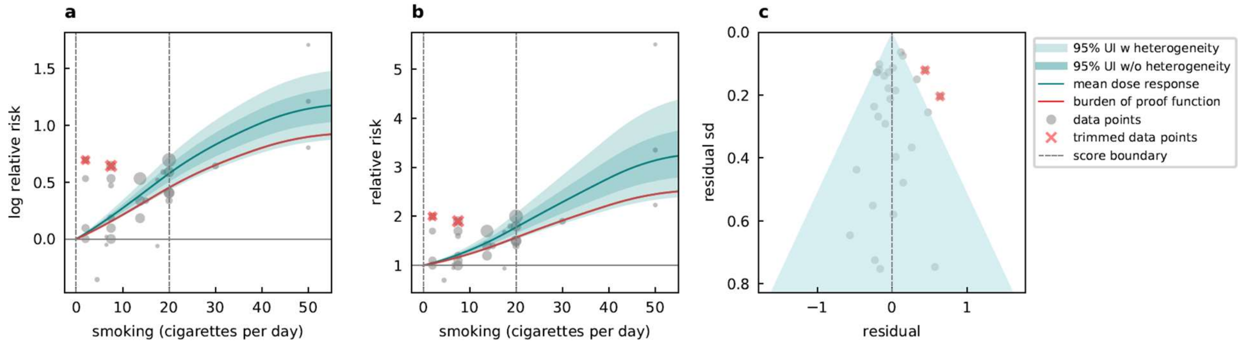

Low back pain

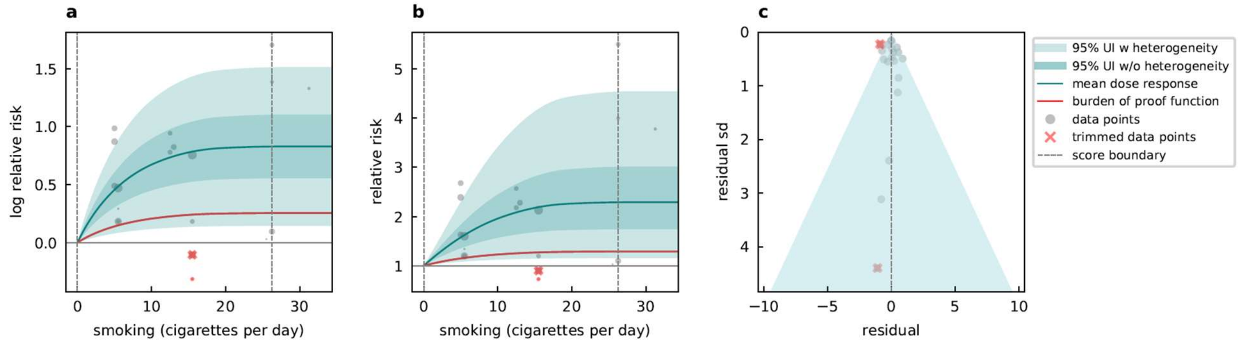

Esophageal cancer

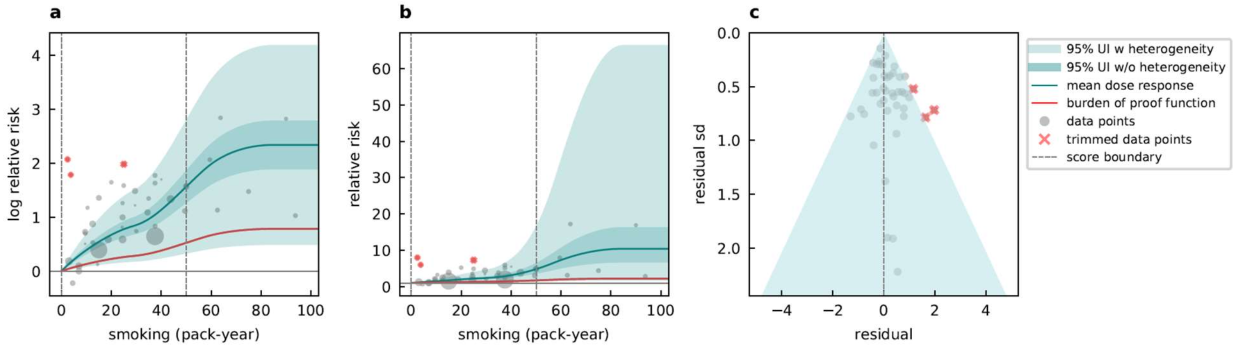

Ischemic heart disease (reference age group: 55-59)

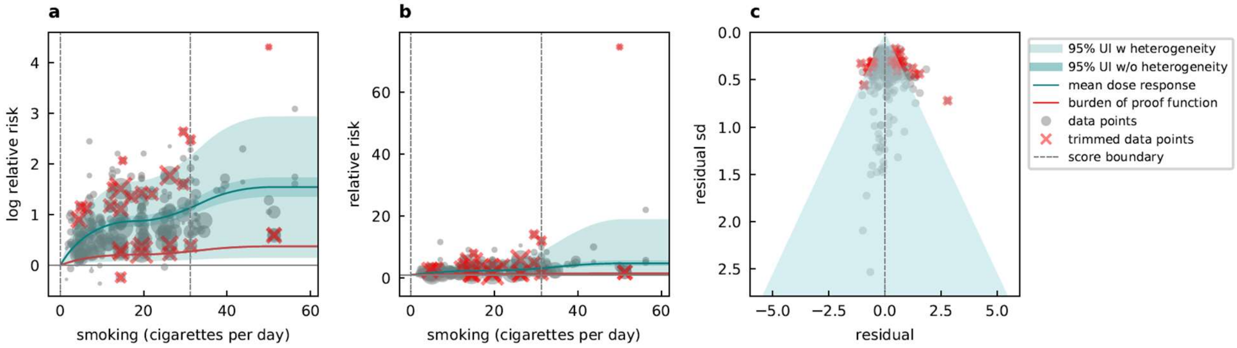

Peptic ulcer disease

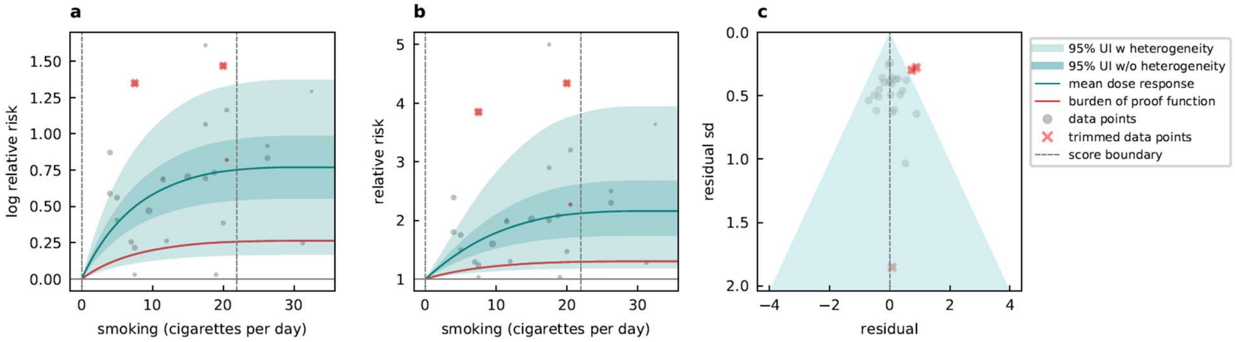

Stroke (reference age group: 55-59)

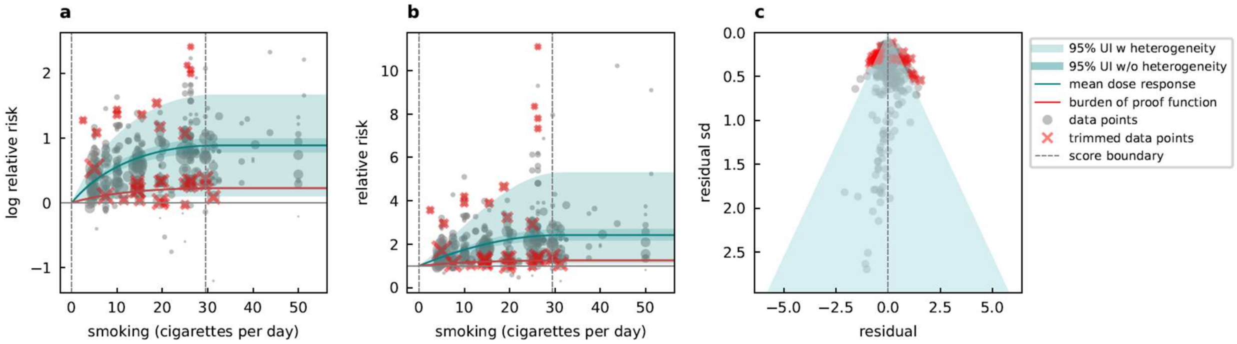

Cervical cancer

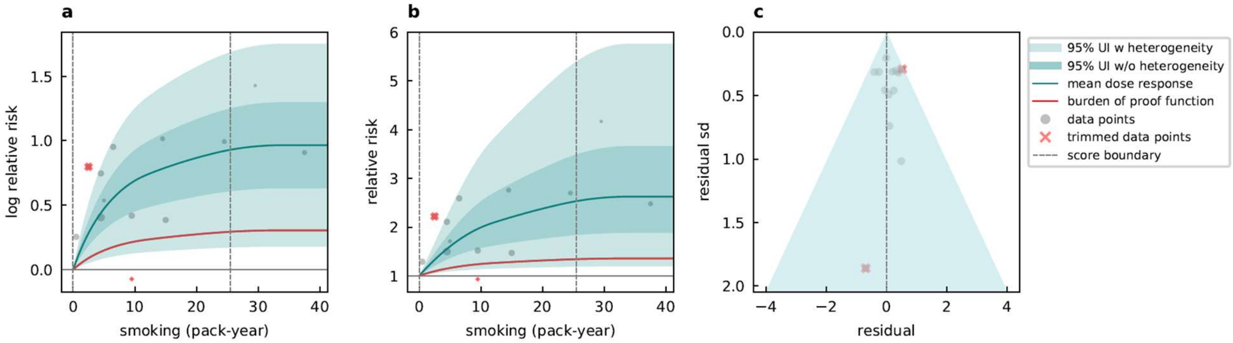

Macular degeneration

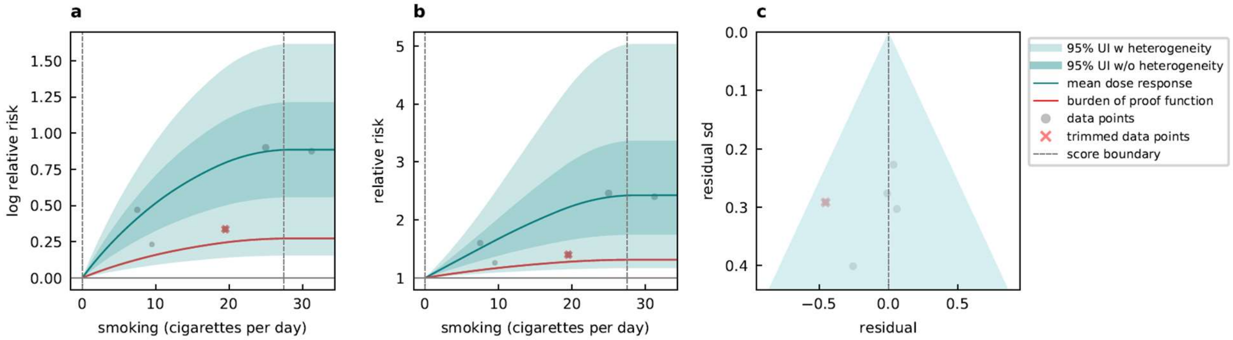

Cataracts

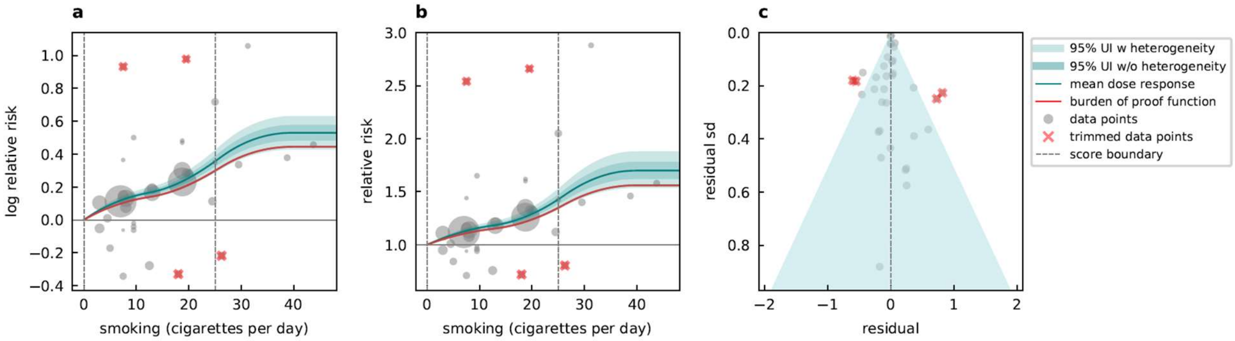

Rheumatoid arthritis

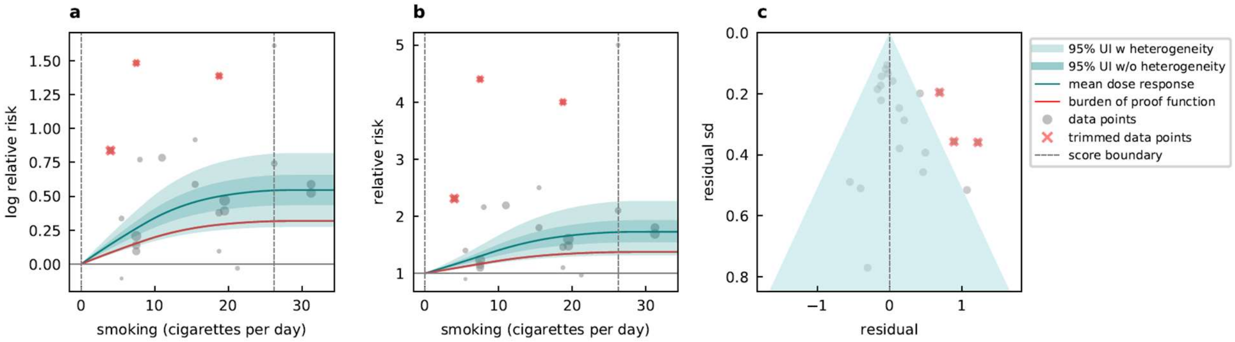

Parkinson's disease (protective effects)

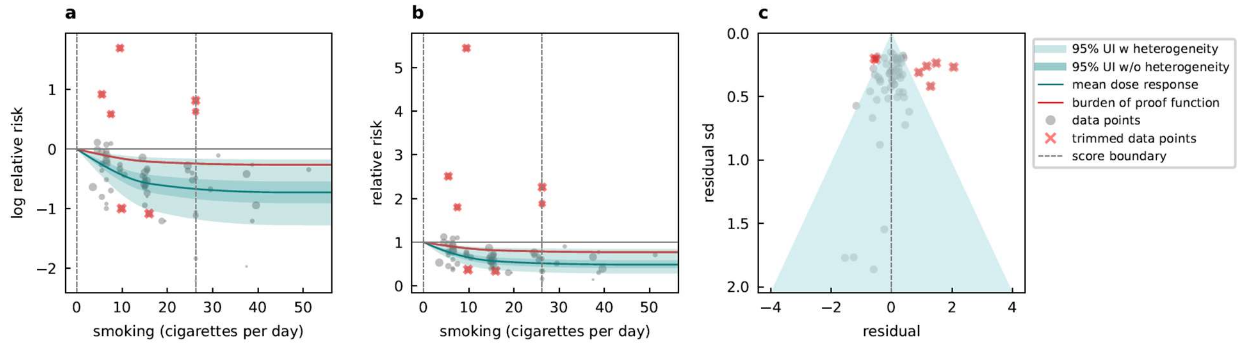

Section 4.4: Two-star smoking-outcome pairs

Nasopharyngeal cancer

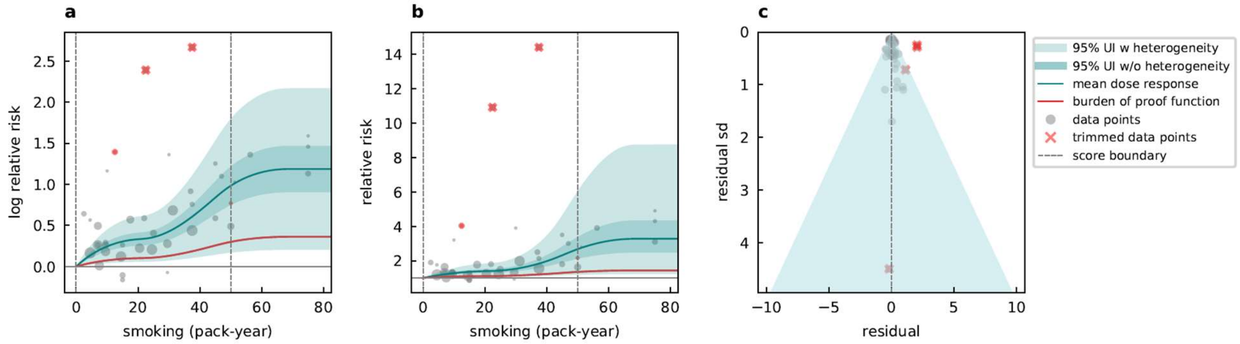

Alzheimer's and other dementia

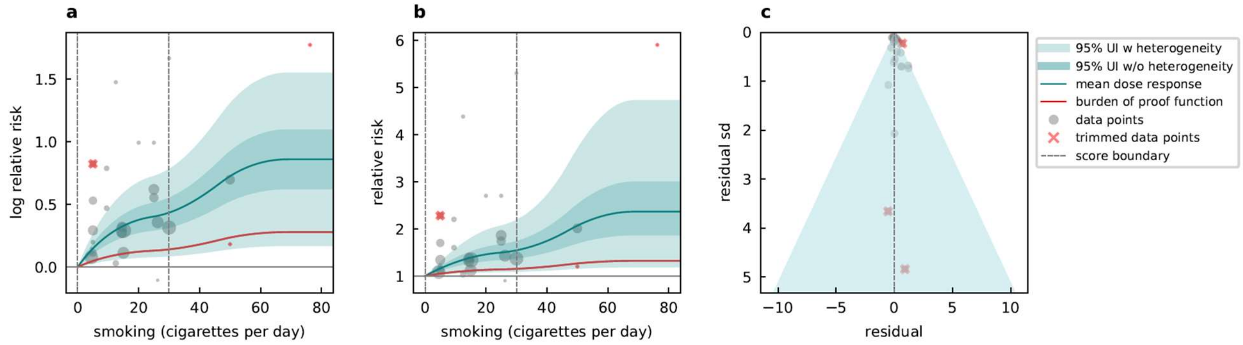

Gallbladder disease

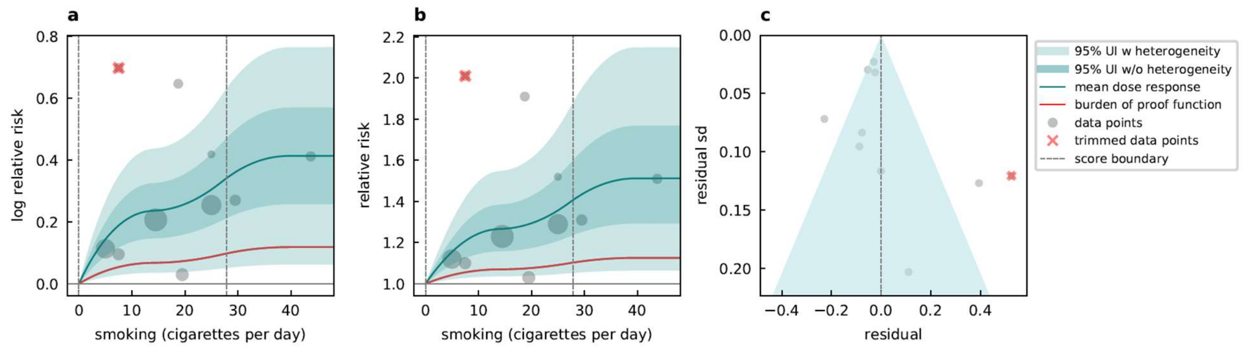

Lip and oral cavity cancer

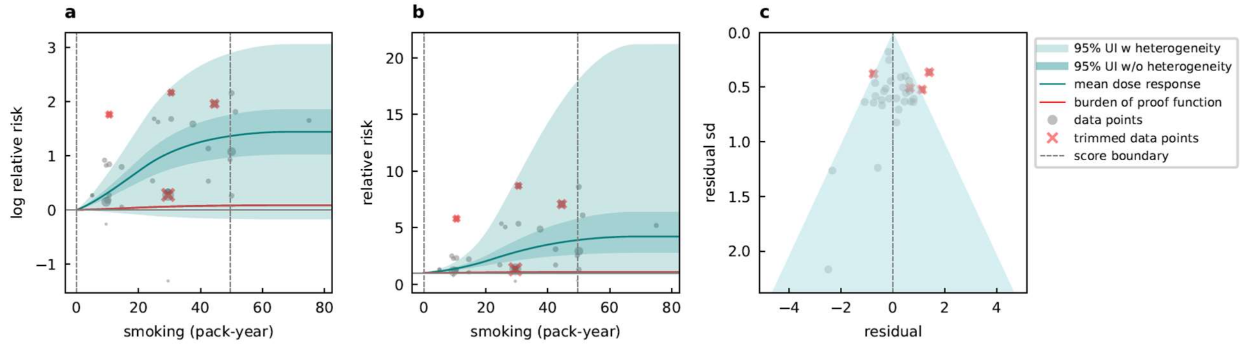

Atrial fibrillation and flutter (reference age group: 55-59)

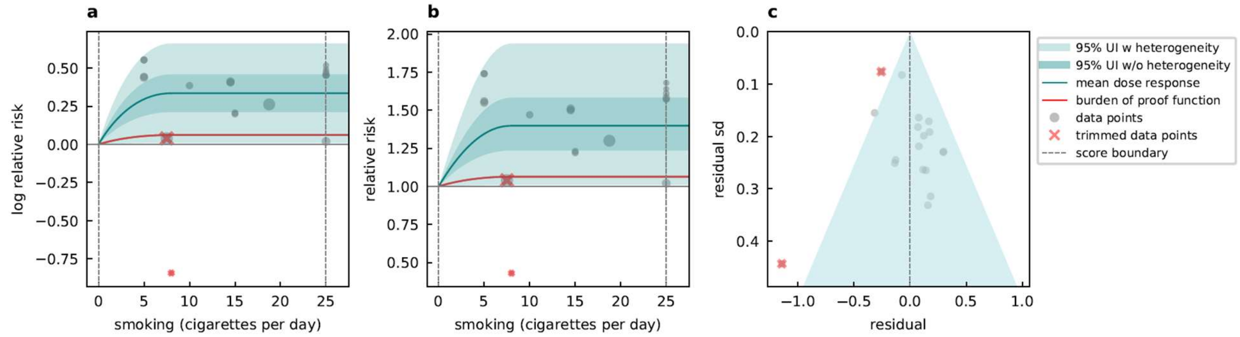

Breast cancer

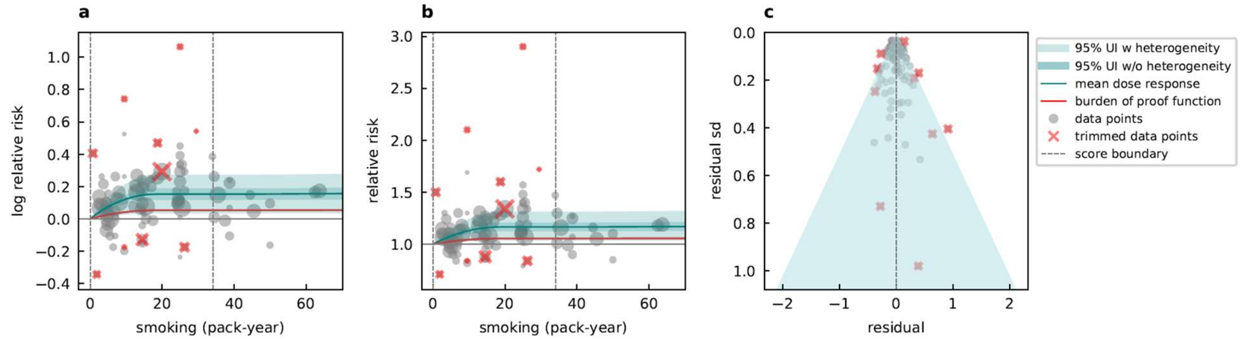

Section 4.5: One-star smoking-outcome pairs

Leukemia

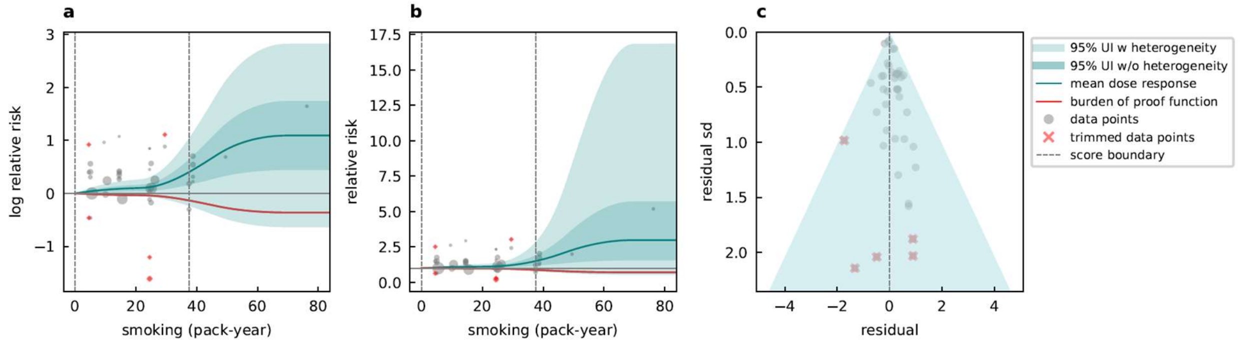

Liver cancer

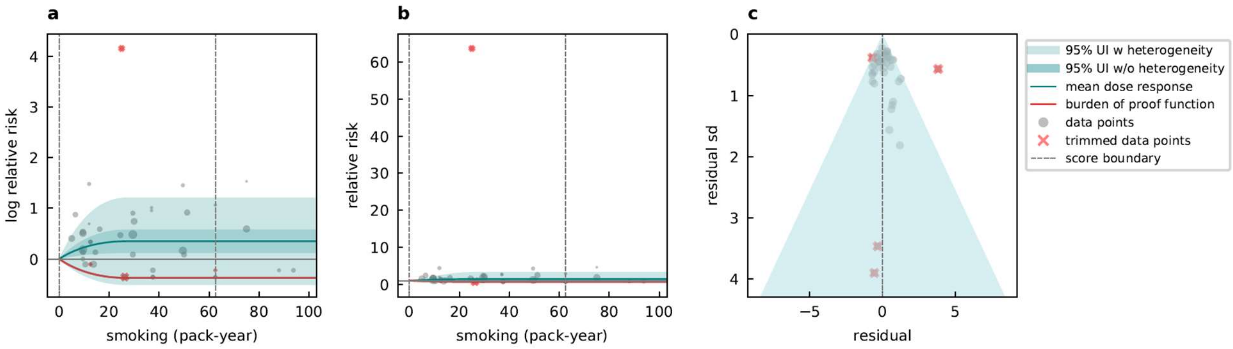

Kidney cancer

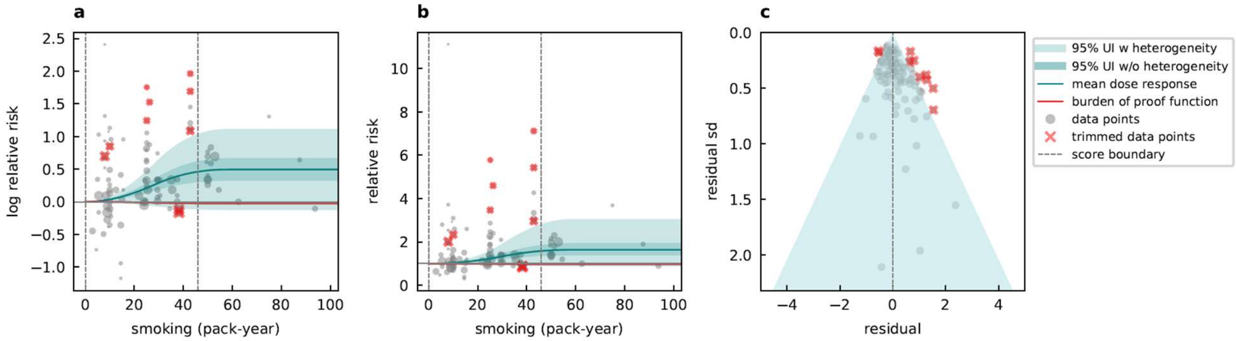

Colon and rectum cancer

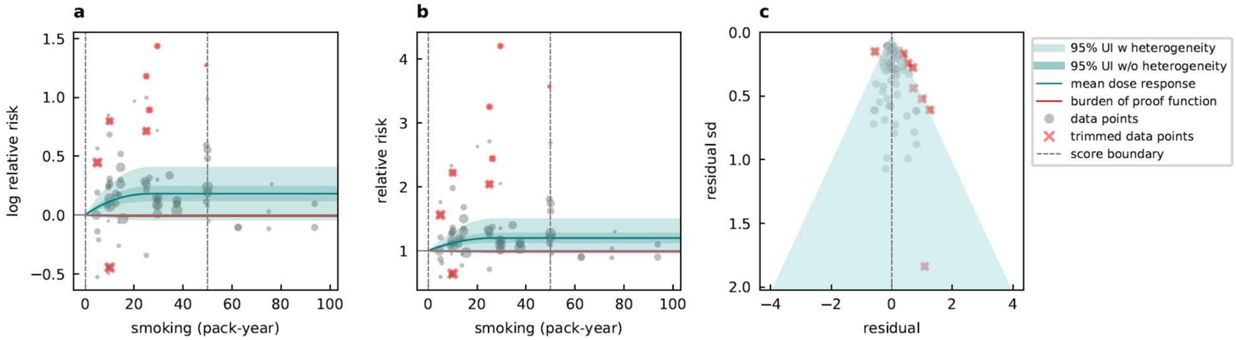

Prostate cancer

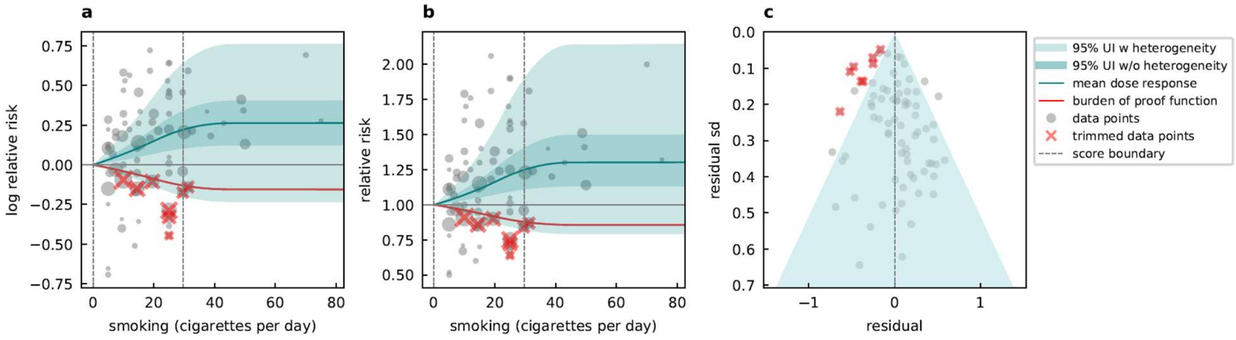

Asthma

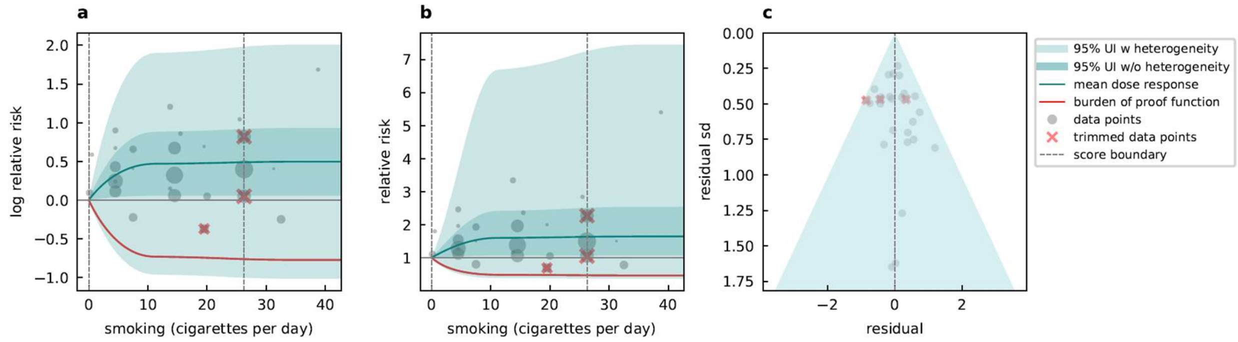

Figure 1: Dose-response relative risk curves of smoking on different health outcomes.
A. *Log-relative risk function.*

B. *Relative risk function.*

C. *A modified funnel plot showing the residuals (relative to 0) on the x-axis and the estimated standard deviation (SD) that includes reported SD and between-study heterogeneity on the y-axis.*

Section 5: Supplementary methods

Section 5.1: The scope of the systematic literature review

Since GBD 2016 we have performed systematic review and meta-analysis of all case-control and prospective cohort studies reporting a relative risk (RR), hazard ratio (HR), or odds ratio (OR) for any risk-outcome pair studied in GBD 2016. In GBD 2019, we had included 36 risk-outcome pairs for smoking. Studies were included if they reported a categorical or continuous dose for smoked tobacco consumption (pack-years or cigarettes per day) as well as uncertainty measures of the estimated risk, and the population under study was the general population. Studies were excluded if they used cross-sectional or retrospective cohort design or if the study was conducted among specific high-risk populations (e.g., people with type 2 diabetes or drug users, etc.).

In GBD 2020, we undertook an effort to improve our RR curves by refining our search strings to capture a larger number of studies than was not identified by previous search strings. Studies published between 01/01/1970 and 31/05/2022 were reviewed. Of those articles captured, prospective cohort and case-control studies were included if they reported the effect sizes (RR, HR, or OR) of an association between a continuous or categorical dose for smoked tobacco consumption and a GBD outcome with uncertainty. Information on study design, confounders controlled for, sample representativeness, and measurement of exposure and outcomes was also extracted.

In GBD 2020, we also employed a new approach to produce age-specific RR curves for CVD outcomes, which involves estimating an age pattern of excess risk (i.e., RR-1) of smoking for CVD outcomes. To estimate the age pattern, we performed a systematic review of literature on risk of smoking for five CVD outcomes, namely, ischemic heart disease, stroke, atrial fibrillation and flutter, aortic aneurysm, and peripheral arterial disease. We developed a search string to search for articles reporting any association of binary smoking status (i.e., current, former, and ever smokers) on the five CVD outcomes from 01/01/1970 to 05/01/2019 and only included studies reporting age-specific risk (RR, OR, HR) of smoking status, which is different from the estimation of dose-response risk of smoking for which we only included studies reporting dose-specific risk. Information on study design, confounders controlled for, sample representativeness, type of exposure (i.e., current, former, and ever smoker), measurement of exposure and outcomes was also extracted for bias adjustment. Table 2 summarizes the number of studies included for estimating the dose-response risk curve and the age pattern of risk for the CVD outcomes.

Table 5: Data inputs for relative risks for smoked tobacco use

	Total unique sources	Total sources*	Total observations
Dose-response risk	559	634	2575
Age pattern of risk for CVD outcomes	159	244	1318

* The total number of source-outcome combinations included in the relative risk models, accounting for sources that reported relative risks for multiple outcomes.

Section 5.2: Detailed methods for estimating the dose-response risk curves of the 36 health outcomes

The same risk-outcome pairs from GBD 2019 were used for GBD 2020: tuberculosis, lower respiratory tract infections, esophageal cancer, stomach cancer, bladder cancer, liver cancer, laryngeal cancer, lung cancer, breast cancer, cervical cancer, colorectal cancer, lip and oral cancer, nasopharyngeal cancer, other pharyngeal cancer, pancreatic cancer, kidney cancer, leukemia, ischemic heart disease, ischemic stroke, hemorrhagic stroke, subarachnoid hemorrhage, atrial fibrillation and flutter, aortic aneurysm, peripheral arterial disease, chronic obstructive pulmonary disease, other chronic respiratory diseases, asthma, peptic ulcer disease, gallbladder and biliary tract diseases, Alzheimer disease and other dementias, Parkinson disease (protective), multiple sclerosis, type 2 diabetes, rheumatoid arthritis, low back pain, cataracts, macular degeneration, and fracture.

For GBD 2020, the risk of all risk outcome pairs is evaluated by continuous smoking exposure level (i.e., pack-year, cigarettes per smoker per day and years since cessation), expect for fracture, whose risk is evaluated by binary smoking exposure (i.e., smoker vs non-smoker/never smoker).

Section 5.2.1: Dose-response risk curves

Since GBD 2016, we had used the studies identified through the systematic review to estimate dose-response risk of smoking on related health outcomes, using DisMod ODE. We chose DisMod ODE rather than a conventional mixed effects meta-regression because of its ability to estimate nonparametric splines over doses (i.e., there is usually a non-linear relationship between smoking exposure level and outcome risk) and incorporate heterogeneous doses through dose-integration (i.e., most studies report smoking exposure level categorically in wide ranges and DisMod ODE can estimate risk of specific exposure level when categories overlap across studies, through an integration step).

For GBD 2020, we used the studies identified through the updated systematic review to estimate new dose-response curves using MR-BRT for all outcomes. Importantly, this new method takes into account the risk of biases in the RR estimation by selecting and including important covariates of the risk estimates in the model (e.g., measurement of exposure and outcomes, representativeness, and adjustment level of the risk estimates) and incorporates unexplained between-study heterogeneity into the uncertainty of the RR estimates. The results of the meta-regression were used to estimate a non-parametric curve for all doses between zero and 100 pack-years or cigarettes per smoker per day and their corresponding relative risks. For all outcomes, we assumed the relative risk was the same for both sexes, except for breast cancer, cervical cancer, and prostate cancer, which were assumed to apply only to females or to male.

For data-sparse risk-outcome pairs, we implemented the Fisher Scoring correction to the heterogeneity parameter. When data are sparse, the between-study heterogeneity parameter estimate may be 0, simply due to lack of data. The Fisher Scoring correction uses a quantile of gamma, which is sensitive to the number of studies, study design, and reported uncertainty.

We have also added methodology that can detect and flag publication bias. The approach is based on the classic Egger's Regression strategy, which is applied to the residuals in our model. In the current implementation, we do not correct for publication bias, but flag the risk-outcome pairs where the risk for publication bias is significant.

In the table below, we list each risk outcome pair that is updated in GBD 2020 along with several of the key modelling parameters and results. The formulation for MR-BRT is described in detail in Zheng et al. (2021)[28].

Table 6: MR-BRT model specifications by risk-outcome pair

**Risk-outcome**	**Type of risk**	**Spline degree, # interior knots**	**Priors & constraints**
Atrial fibrillation and flutter	Continuous, Harmful	Quadratic, 3 I knots	Monotonic increasing, right linear tail, Gaussian max derivative prior on the right tail (0, 0.001)
Alzheimer and other dementia	Continuous, Harmful	Quadratic, 3 I knots	Monotonic increasing, right linear tail, Gaussian max derivative prior on the right tail (0, 0.001)
Aortic aneurism	Continuous, Harmful	Quadratic, 3 I knots	Monotonic increasing, right linear tail, Gaussian max derivative prior on the right tail (0, 0.001)
Asthma	Continuous, Harmful	Quadratic, 3 I knots	Monotonic increasing, right linear tail, Gaussian max derivative prior on the right tail (0, 0.001)
Bladder cancer	Continuous, Harmful	Quadratic, 3 I knots	Monotonic increasing, right linear tail, Gaussian max derivative prior on the right tail (0, 0.001)
Breast cancer	Continuous, Harmful	Quadratic, 3 I knots	Monotonic increasing, right linear tail, Gaussian max derivative prior on the right tail (0, 0.001)
Cataracts	Continuous, Harmful	Quadratic, 3 I knots	Monotonic increasing, right linear tail, Gaussian max derivative prior on the right tail (0, 0.001)
Cervical cancer	Continuous, Harmful	Quadratic, 3 I knots	Monotonic increasing, right linear tail, Gaussian max derivative prior on the right tail (0, 0.001)
Colon and rectum cancer	Continuous, Harmful	Quadratic, 3 I knots	Monotonic increasing, right linear tail, Gaussian max derivative prior on the right tail (0, 0.001)
COPD	Continuous, Harmful	Quadratic, 3 I knots	Monotonic increasing, right linear tail, Gaussian max derivative prior on the right tail (0, 0.001)
Type 2 diabetes	Continuous, Harmful	Quadratic, 3 I knots	Monotonic increasing, right linear tail, Gaussian max derivative prior on the right tail (0, 0.001)
Esophageal cancer	Continuous, Harmful	Quadratic, 3 I knots	Monotonic increasing, right linear tail, Gaussian max derivative prior on the right tail (0, 0.001)
Gallbladder diseases	Continuous, Harmful	Quadratic, 3 I knots	Monotonic increasing, right linear tail, Gaussian max derivative prior on the right tail (0, 0.001)
Fracture (hip and non-hip)	Dichotomous, Harmful	N/A	N/A

Ischemic health disease	Continuous, Harmful	Quadratic, 3 I knots	Monotonic increasing, right linear tail, Gaussian max derivative prior on the right tail (0, 0.001)
Kidney cancer	Continuous, Harmful	Quadratic, 3 I knots	Monotonic increasing, right linear tail, Gaussian max derivative prior on the right tail (0, 0.001)
Laryngeal cancer	Continuous, Harmful	Quadratic, 3 I knots	Monotonic increasing, right linear tail, Gaussian max derivative prior on the right tail (0, 0.001)
Lower back pain	Continuous, Harmful	Quadratic, 3 I knots	Monotonic increasing, right linear tail, Gaussian max derivative prior on the right tail (0, 0.001)
Leukemia	Continuous, Harmful	Quadratic, 3 I knots	Monotonic increasing, right linear tail, Gaussian max derivative prior on the right tail (0, 0.001)
Lip oral cavity cancer	Continuous, Harmful	Quadratic, 3 I knots	Monotonic increasing, right linear tail, Gaussian max derivative prior on the right tail (0, 0.001)
Liver cancer	Continuous, Harmful	Quadratic, 3 I knots	Monotonic increasing, right linear tail, Gaussian max derivative prior on the right tail (0, 0.001)
lower respiratory infections	Continuous, Harmful	Quadratic, 3 I knots	Monotonic increasing, right linear tail, Gaussian max derivative prior on the right tail (0, 0.001)
Lung cancer	Continuous, Harmful	Quadratic, 3 I knots	Monotonic increasing, right linear tail, Gaussian max derivative prior on the right tail (0, 0.001)
Macular degeneration	Continuous, Harmful	Quadratic, 3 I knots	Monotonic increasing, right linear tail, Gaussian max derivative prior on the right tail (0, 0.001)
Multiple sclerosis	Continuous, Harmful	Quadratic, 3 I knots	Monotonic increasing, right linear tail, Gaussian max derivative prior on the right tail (0, 0.001)
Nasopharyngeal cancer	Continuous, Harmful	Quadratic, 3 I knots	Monotonic increasing, right linear tail, Gaussian max derivative prior on the right tail (0, 0.001)
Other pharynx cancer	Continuous, Harmful	Quadratic, 3 I knots	Monotonic increasing, right linear tail, Gaussian max derivative prior on the right tail (0, 0.001)
Pancreatic cancer	Continuous, Harmful	Quadratic, 3 I knots	Monotonic increasing, right linear tail, Gaussian max derivative prior on the right tail (0, 0.001)
Parkinson	Continuous, Protective	Quadratic, 3 I knots	Monotonic decreasing, right linear tail, Gaussian max derivative prior on the right tail (0, 0.001)
Peptic ulcer	Continuous, Harmful	Quadratic, 3 I knots	Monotonic increasing, right linear tail, Gaussian max derivative prior on the right tail (0, 0.001)
Peripheral artery disease	Continuous, Harmful	Quadratic, 3 I knots	Monotonic increasing, right linear tail, Gaussian max derivative prior on the right tail (0, 0.001)

Prostate cancer	Continuous, Harmful	Quadratic, 3 I knots	Monotonic increasing, right linear tail, Gaussian max derivative prior on the right tail (0, 0.001)
Rheumatoid arthritis	Continuous, Harmful	Quadratic, 3 I knots	Monotonic increasing, right linear tail, Gaussian max derivative prior on the right tail (0, 0.001)
Stomach cancer	Continuous, Harmful	Quadratic, 3 I knots	Monotonic increasing, right linear tail, Gaussian max derivative prior on the right tail (0, 0.001)
Stroke (ischemic stroke, hemorrhagic stroke, and subarachnoid hemorrhage)	Continuous, Harmful	Quadratic, 3 I knots	Monotonic increasing, right linear tail, Gaussian max derivative prior on the right tail (0, 0.001)
Tuberculosis	Continuous, Harmful	Quadratic, 3 I knots	Monotonic increasing, right linear tail, Gaussian max derivative prior on the right tail (0, 0.001)

Table 7: MR BRT estimated parameters and bias covariates by risk-outcome pair

**Risk-outcome**	**Unit of risk**	**Selected bias covariates**	**Mean gamma solution**
Atrial fibrillation and flutter	cigarettes per day	None	0.000
Alzheimer and other dementia	cigarettes per day	None	0.054
Aortic aneurism	cigarettes per day	None	0.000
Asthma	cigarettes per day	None	1.651
Bladder cancer	pack-year	None	0.052
Breast cancer	pack-year	cv_subpopulation	0.000
Cataracts	cigarettes per day	None	0.000
Cervical cancer	pack-year	None	0.000
Colon and rectum cancer	pack-year	None	0.090
COPD	pack-year	cv_older, cv_adj_L1	0.022
Type 2 diabetes	cigarettes per day	cv_subpopulation	0.055
Esophageal cancer	pack-year	None	0.106
Gallbladder diseases	cigarettes per day	cv_adj_L0	0.000
Fracture (hip and non-hip)	Binary smoking status	cv_subpopulation, cv_risk_measure, cv_adj_L2	0.032

Ischemic health disease	cigarettes per day	cv_adj_L2, cv_subpopulation, cv_older	0.190
Kidney cancer	pack-year	None	0.078
Laryngeal cancer	pack-year	None	0.000
Lower back pain	cigarettes per day	None	0.000
Leukemia	pack-year	None	0.000
Lip oral cavity cancer	pack-year	cv_adj_L1	0.158
Liver cancer	pack-year	None	0.429
Lower respiratory infection	cigarettes per day	None	0.000
Lung cancer	pack-year	cv_adj_L1, cv_adj_L0, cv_adj_L2	0.063
Macular degeneration	cigarettes per day	None	0.000
Multiple sclerosis	cigarettes per day	None	0.000
Nasopharyngeal cancer	pack-year	cv_adj_L0	0.065
Other pharynx cancer	pack-year	None	0.000
Pancreatic cancer	pack-year	None	0.000
Parkinson	cigarettes per day	cv_adj_L2, cv_outcome_selfreport	0.000
Peptic ulcer disease	cigarettes per day	cv_adj_L1, cv_subpopulation	0.000
Peripheral artery disease	cigarettes per day	cv_subpopulation	0.000
Prostate cancer	cigarettes per day	None	0.170
Rheumatoid arthritis	cigarettes per day	None	0.000
Stomach cancer	pack-year	None	0.000
Stroke (ischemic stroke, hemorrhagic stroke, and subarachnoid hemorrhage)	cigarettes per day	None	0.146
Tuberculosis	cigarettes per day	None	0.038

† Definitions of bias covariates:

cv_subpopulation: 0 for risk estimates are likely generalizable to the general population because the sample was based on the general population with reasonable exclusions for pre-existing disease states; 1 for risk estimates of sub-groups such as high-risk groups

cv_adj_L0, cv_adj_L1, cv_adj_L2: cascading dummy variables for adjustment level of the risk estimates (i.e., how many confounders are adjusted for in the regression model for the risk estimate). There are four adjustment levels, namely, 1.no adjustment, 2.only adjusting for age and sex, 3.adjusting for age and sex and <= 3 other covariates, and 4.adjusting for age and sex and > 3 other covariates. If the adjustment level is 1, cv_adj_L0=1, cv_adj_L1=1, cv_adj_L2=1; if the adjustment level is 2, cv_adj_L0=1, cv_adj_L1=1, cv_adj_L2=0; if the adjustment level is 3, then cv_adj_L0=1, cv_adj_L1=0, cv_adj_L2=0; if the adjustment level is 4, then cv_adj_L0=0, cv_adj_L1=0, cv_adj_L2=0.

cv_outcome_selfreport: 0 for measurement of outcome based on assays, tests, or physician observation and 1 for self-report outcome.

cv_older: 0 if the population contains both young and old people; 1 if the population only contains old people.

cv_non_smoker: 0 if the unexposed group is never smoker; 1 if the unexposed group is non-smoker (never smoker and possible former smoker).

cv_risk_measure: 0 if the risk is reported as Relative Risk; 1 if the risk is reported as Odds Ratios or Hazard Ratios.

Section 5.2.2: Age-specific dose-response risk curves for CVD outcomes

For all non-CVD outcomes, we assumed the risk curve to be the same for all ages. However, the risk of smoking on CVD outcomes (i.e., stroke, ischemic heart disease, atrial fibrillation and flutter, aortic aneurysm, and peripheral arterial disease) is well known to attenuate with increasing age and thus we produced age-specific risk curves for all CVD outcomes. Previously, we used a linear relationship between age and log risk to adjust all RR data to a specific age group (e.g., 45-49). Then, we modelled the risk curve for each age group using the adjusted age-group specific data. This approach often produced curves with different shapes for different age groups and tended to underestimate the risk for older age groups since we set the log RR to be 0 for the terminal age group (e.g., 95+) in the linear function.

In GBD 2020, we adopted a new approach to produce the age-specific risk curves by producing an age pattern of smoking risk on CVD outcomes and adjusting the risk curve of the reference age group using the age pattern of risk to produce age-group specific risk curves. Briefly, we first estimated the reference dose-response risk of smoking for each CVD outcome using dose-specific RR data of each outcome regardless of the age group information. This step was the same with other non-CVD outcomes. Once we had the reference curve, we determined the age group of the reference curve by calculating the weighted mean age across all dose-specific RR data (weighted by 1/SE of each datum). For example, if the weighted mean age of all dose-specific RR data was 56.5, we determined the age group of the reference risk curve to be 55-59. For cohort studies, the mean age was calculated as mean age at baseline plus the mean/median years of follow-up (if only maximum years of follow-up is reported, we added half of the maximum years to the mean age at baseline). For the case-control studies, the mean age was just the reported mean age at baseline (in case the mean age is not reported, we used the midpoint of age range as the mean age instead). In the third step, we extracted age-group specific RR data and relevant bias covariates from literature identified in the systematic review mentioned above, and we used MR-BRT to model the age pattern of excess risk (i.e., RR-1) of smoking on CVD outcomes with age-group specific excess RR data of all CVD outcomes. In the final model, we included age as spline, random effects of study, and the bias covariates of exposure types (i.e., current, former, and ever smokers), which were selected by an algorithm described elsewhere.[2] When predicting the age pattern of the excess risk of smoking on CVD outcomes using the fitted model, we did not include between study heterogeneity to reduce uncertainty in the prediction. Figure S1 below shows the estimated age pattern of excess risk of smoking on CVD along with its 95% uncertainty intervals. In the fourth step, we calculated the age attenuation factors (AF) of excess risk compared with the reference age group for each CVD outcome as ratio of the estimated excess risk of each age group to that of the reference age group. We did the calculation at the draw level to obtain 1000 draws of

the AF for each age group. Figure S2 below shows the AF for stroke along with its 95% uncertainty intervals. Once we had the AF, in the last step, we adjusted the risk curve of the reference age group from step 1 using equation (1) to produce the age-group specific risk curves for each CVD outcome.

We did the age adjustment on draw level so that the uncertainty of the AF can be naturally incorporated in the final adjusted age-specific RR curves. Figure S3 shows the age AF adjusted age-group specific RR curves for stroke outcome.

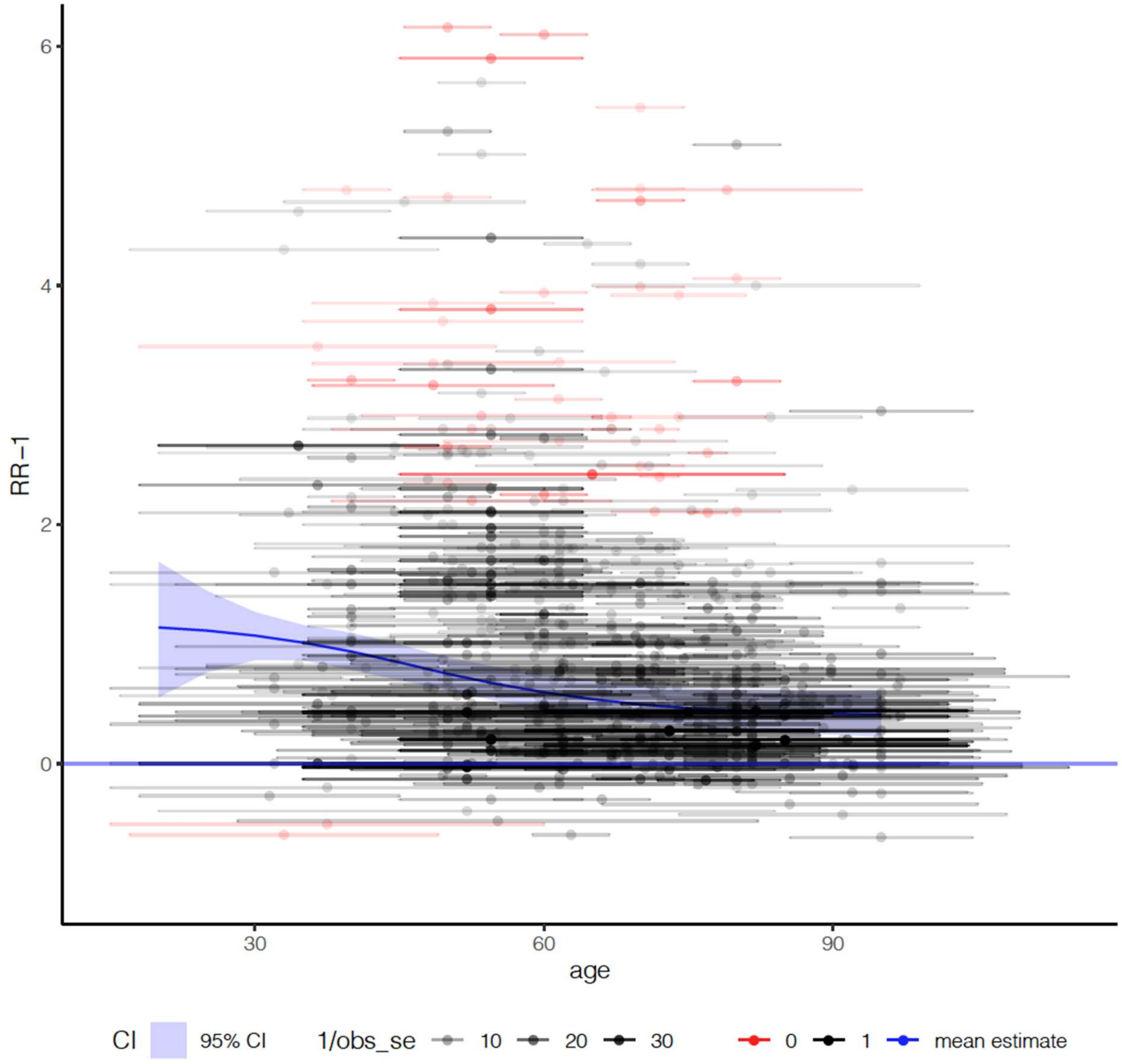

Figure 2: estimated age pattern of excess risk of smoking on CVD outcomes

Note: The red points are trimmed by MR-BRT and the black points are included in the final model. The blue curve is the age trends of risk of smoking on CVD outcomes. The shaded area is the 95% of the age trends not accounting for between study heterogeneity. The whiskers around the data points are the age range of each reported estimate. The opacity of the data point indicates the inverse of standard error of the estimate, with more opaque data having smaller standard error.

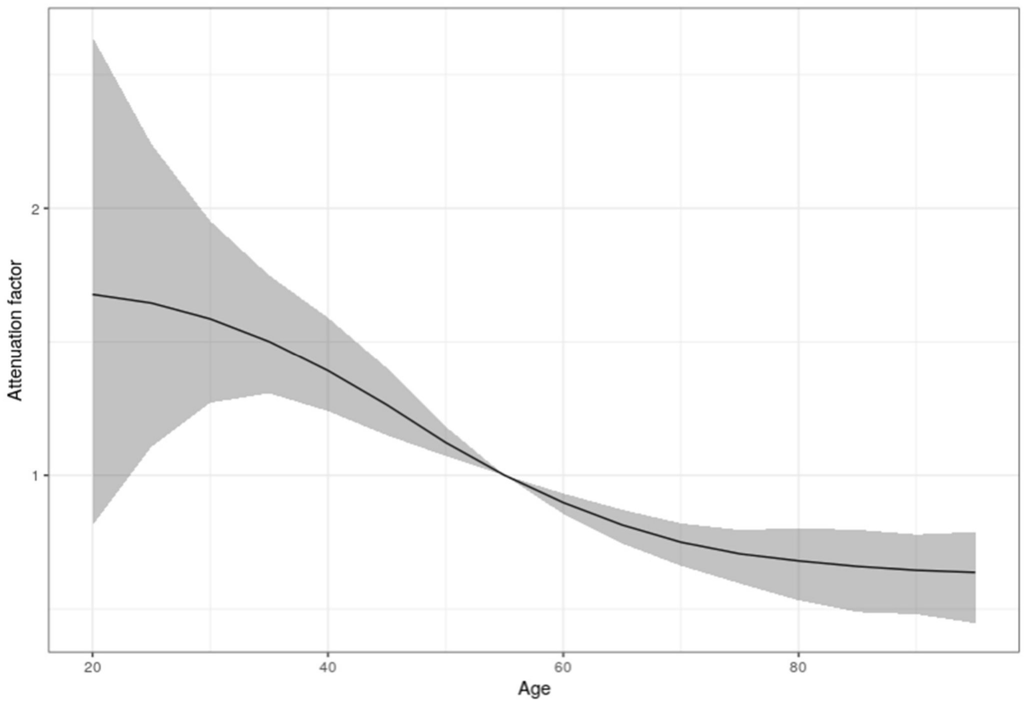

Figure 3: age attenuation factors of excess risk of smoking on stroke compared with the reference age group

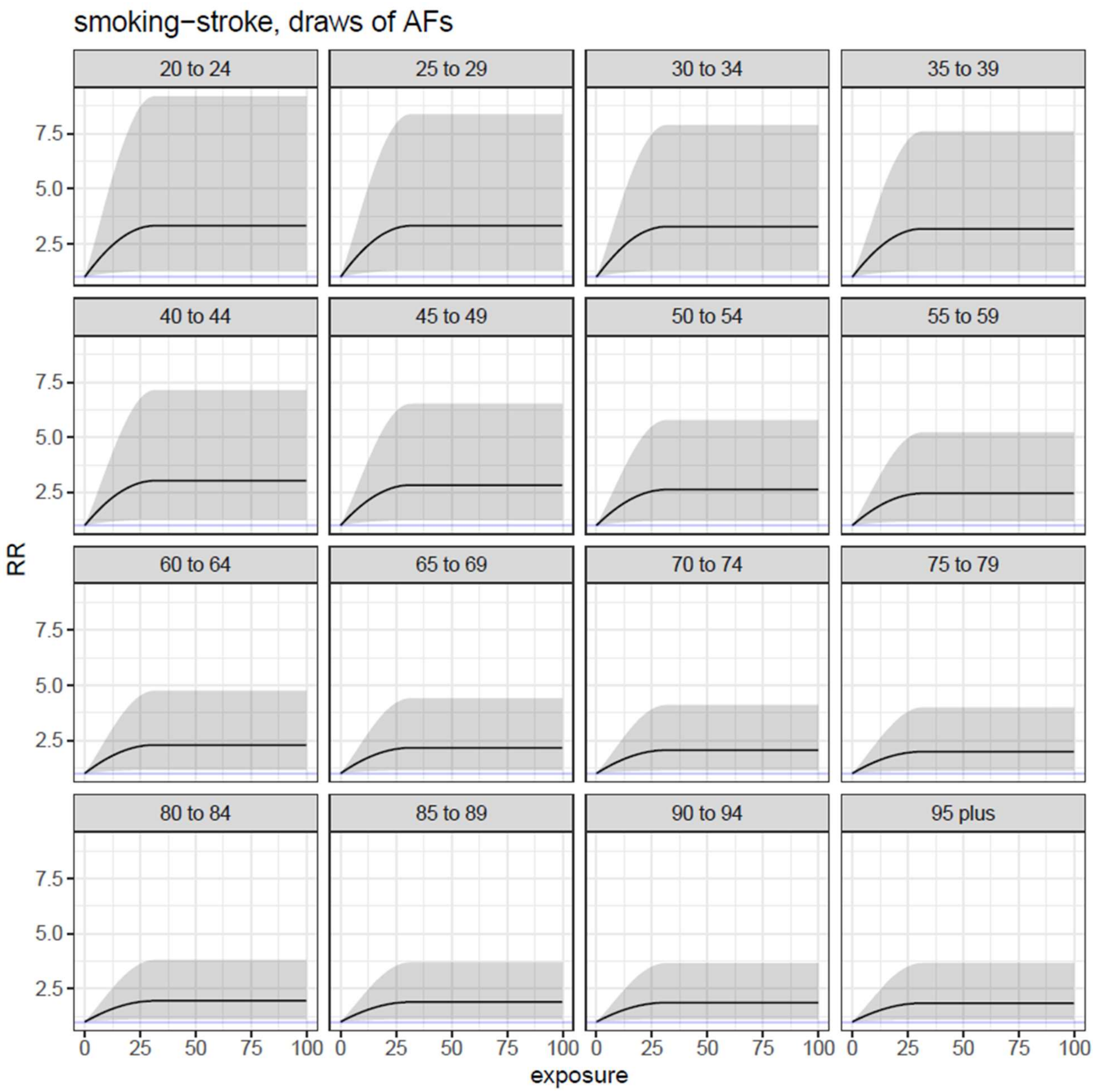

Figure 4: AF adjusted age-group specific RR curves for stroke, reference age group 55-59. The exposure is in units of cigarette-equivalents per smoker per day.

Section 6: Sensitivity analysis

Section 6.1: Testing sensitivity of trimming approach

Similar to our approach to evaluate the impact of the monotonicity constraint, we conducted another sensitivity analysis to evaluate the impact of our 10% trimming on the shape of the risk curves for each risk-outcome pair. Our final models involved trimming 10% of the data for each outcome to remove the likelihood of extreme data and reduce publication bias favoring extreme values. We ran preliminary models with the same model specifications as our final models, except for the 10% trimming, and compared the results of both sets of models. These results can be found below.

Figure 5: Results of model without 10% trimming

The figures below reflect the results of the model without trimming using the final model specificities for every risk-outcome pair. The first of each set of outcome plots presents the dose-response relative risk in log space, while the middle figure is the dose-response relative risk in linear space. The right-most figure reflects the residual cone for all of the data inputs in the risk-outcome pair. The reference exposure for all of these risk curves is the theoretical minimum risk exposure level, 0 cigarettes per day or 0 pack-years (depending on the outcome in question).

COPD

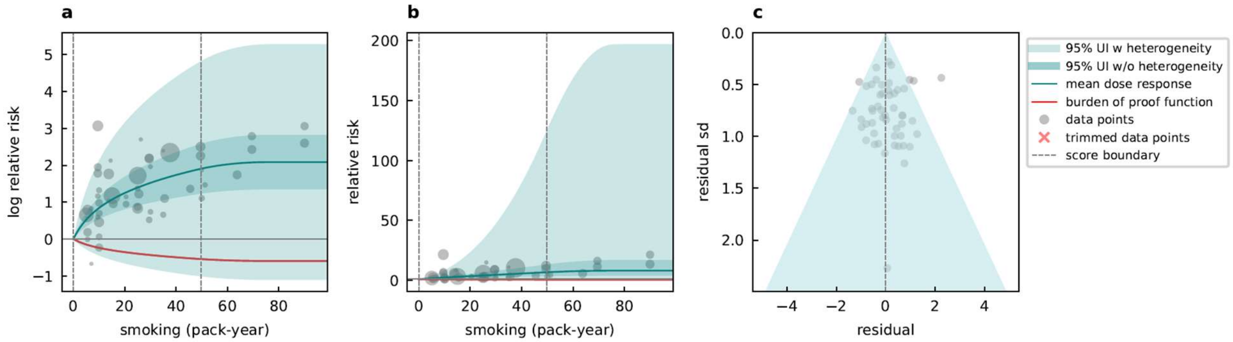

Colon and Rectum Cancer

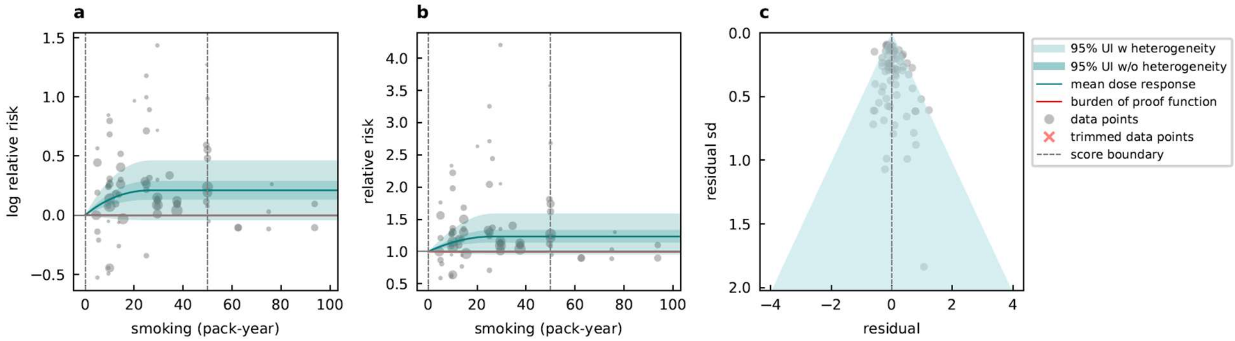

Cervical Cancer

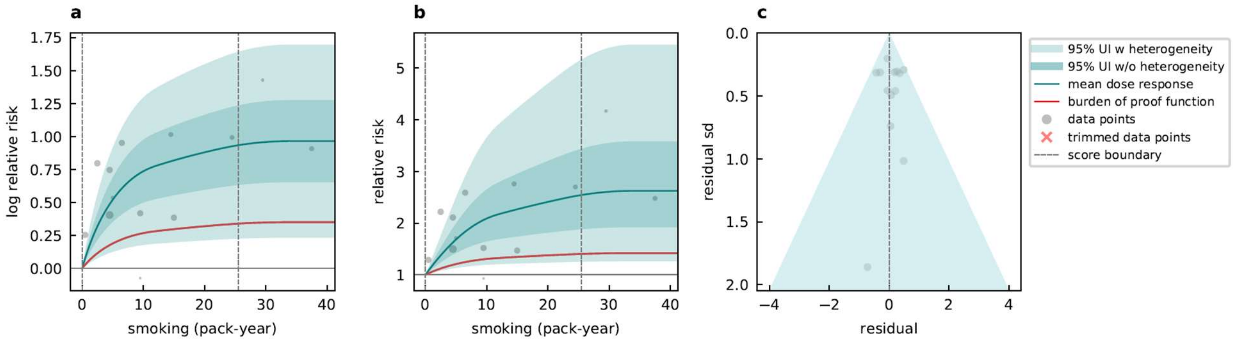

Cataracts

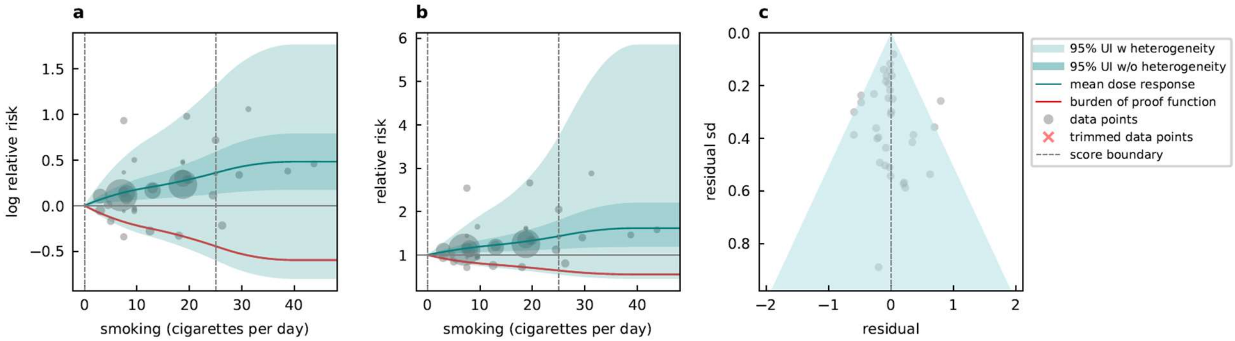

Breast Cancer

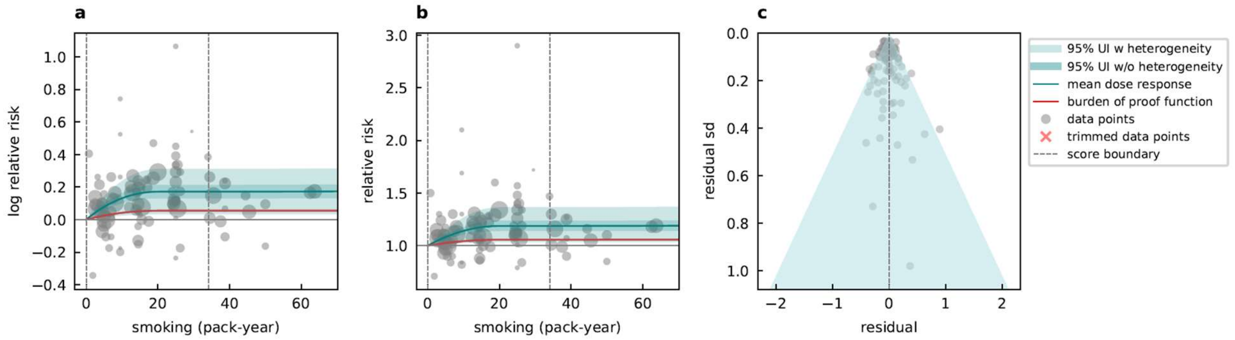

Bladder Cancer

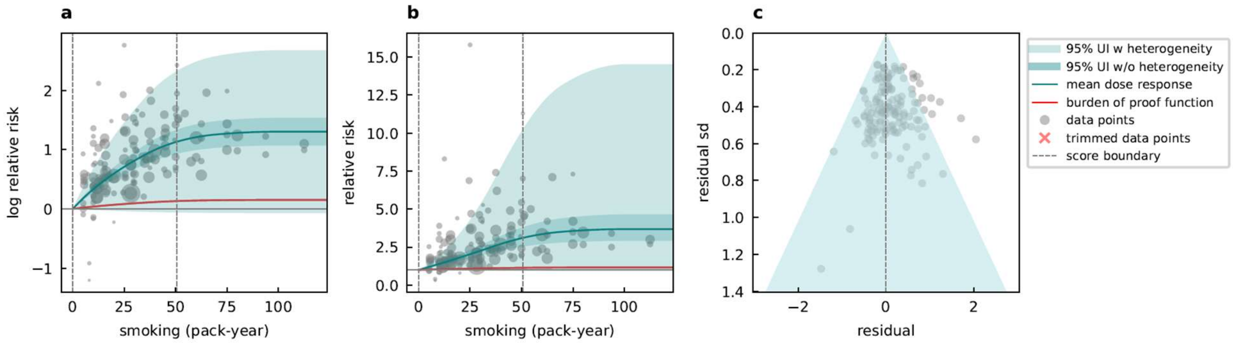

Asthma

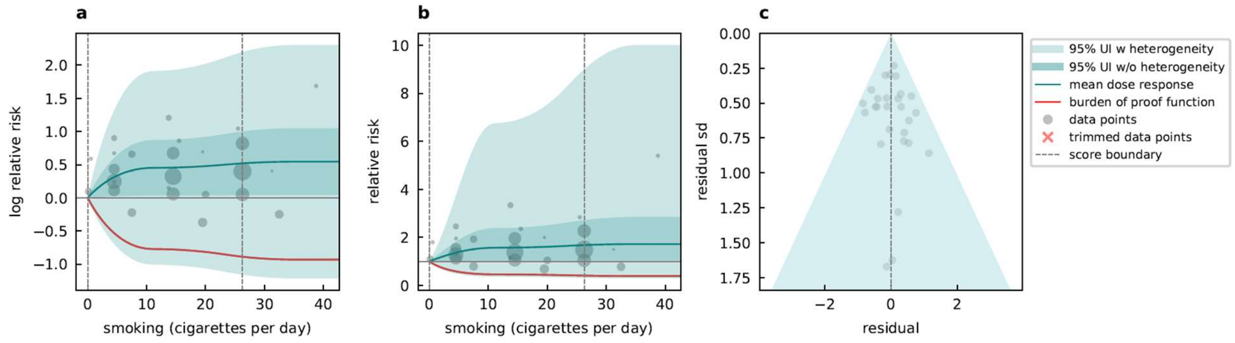

Aortic Aneurysm

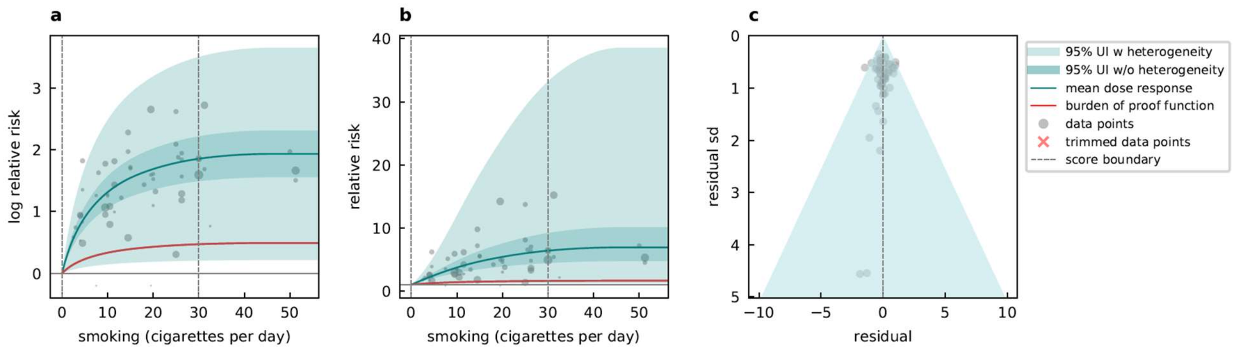

Atrial Fibrillation and Flutter

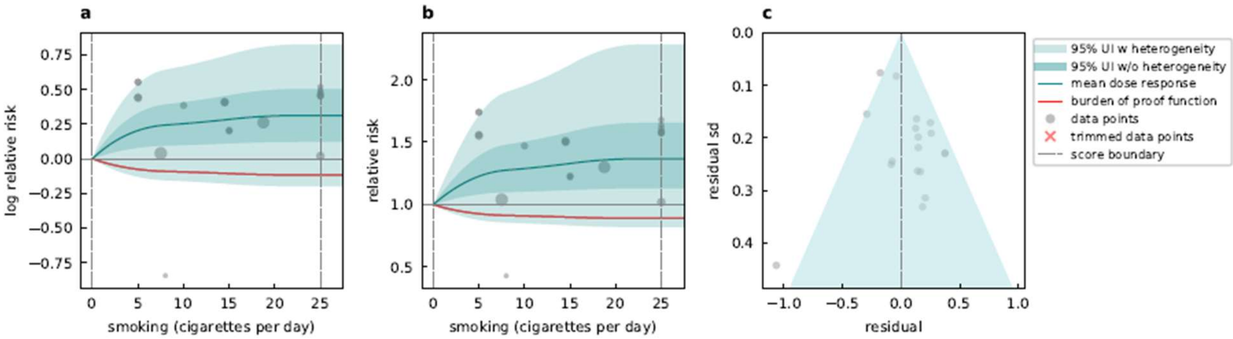

Alzheimer's and Other Dementia

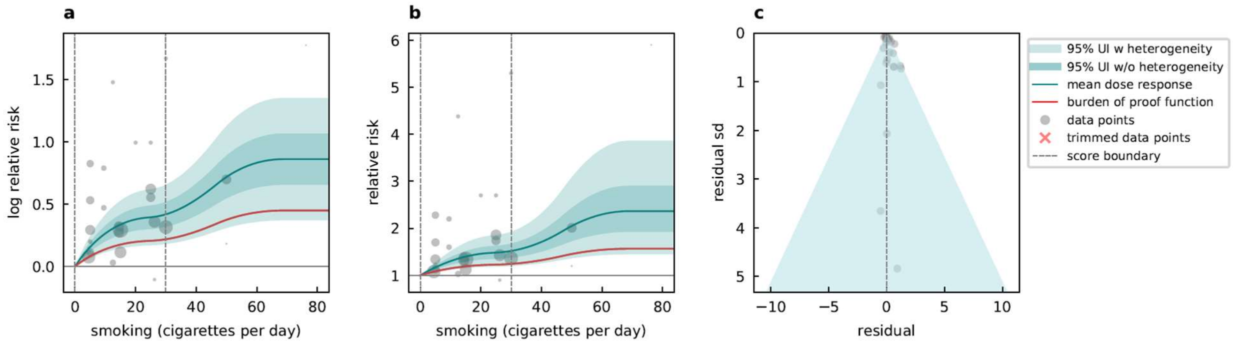

Lung Cancer

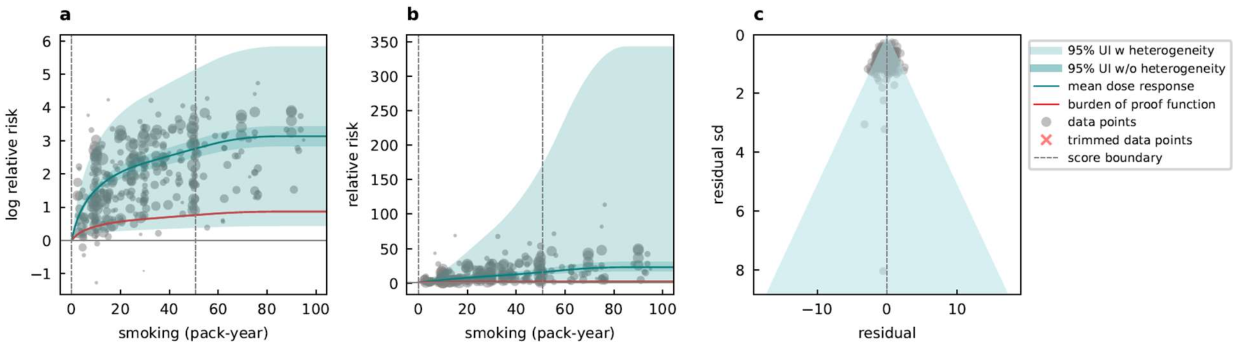

Lower Respiratory Infections

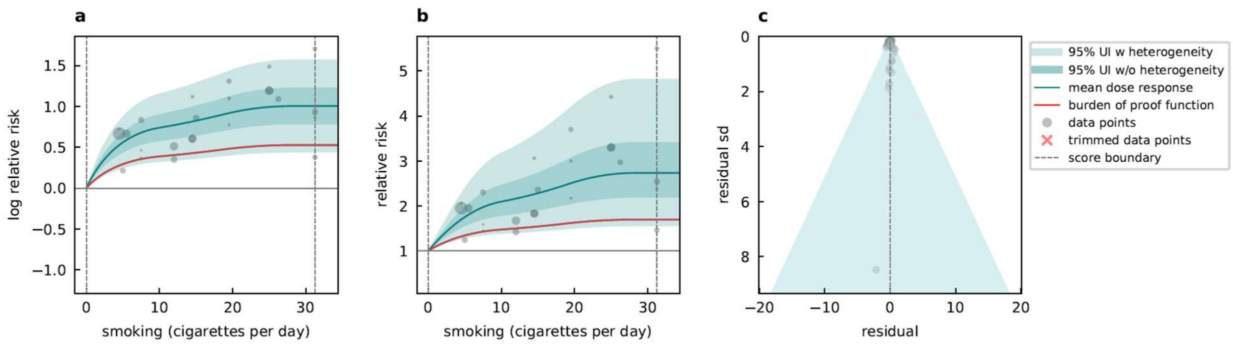

Liver Cancer

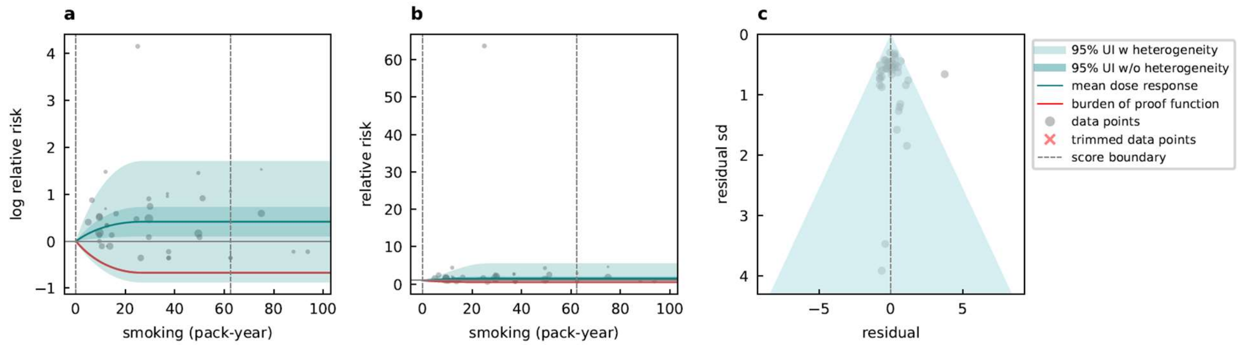

Lip and Oral Cavity Cancer

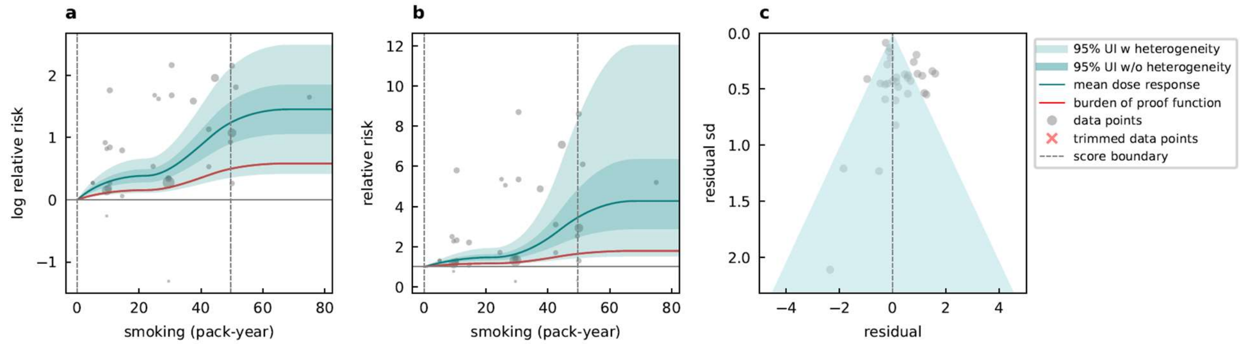

Leukemia

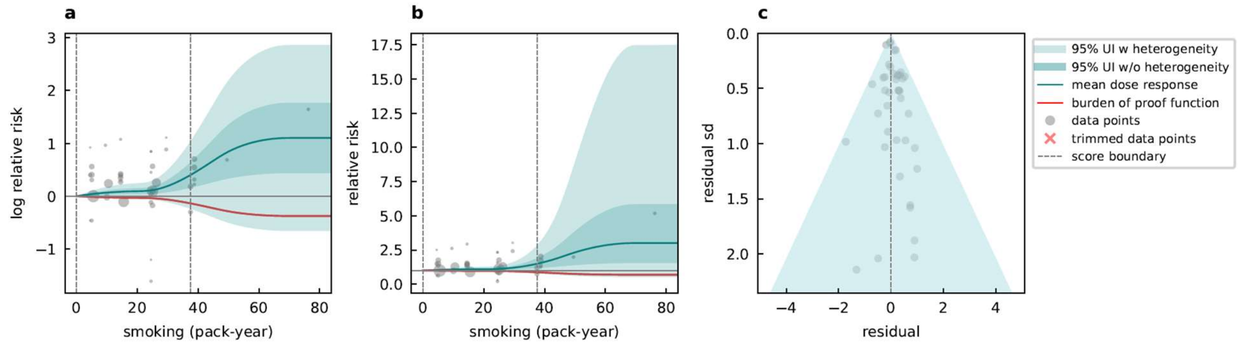

Low Back Pain

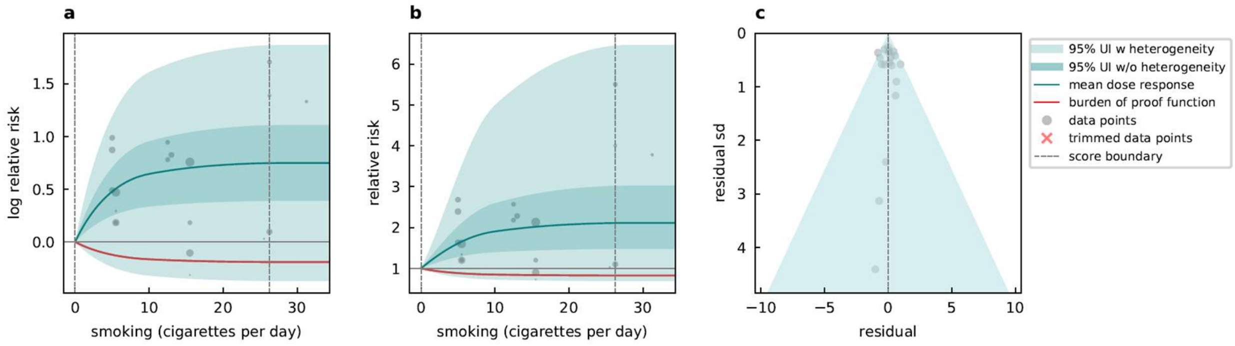

Laryngeal Cancer

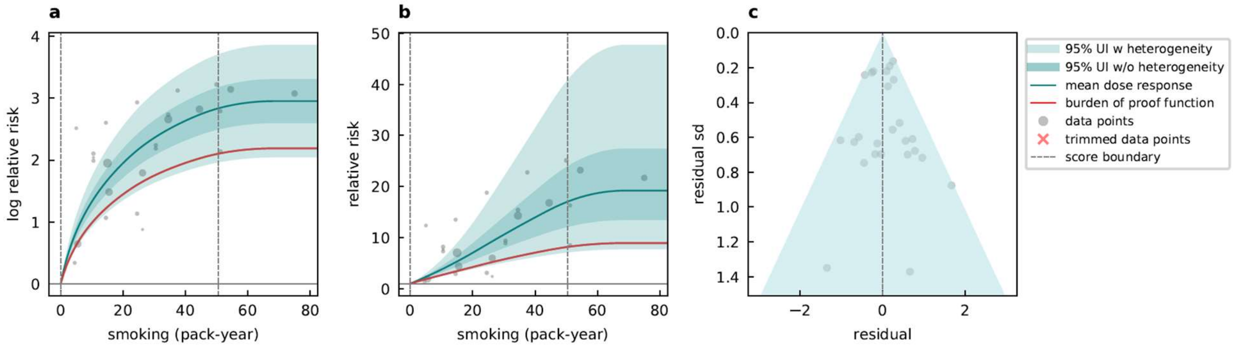

Kidney Cancer

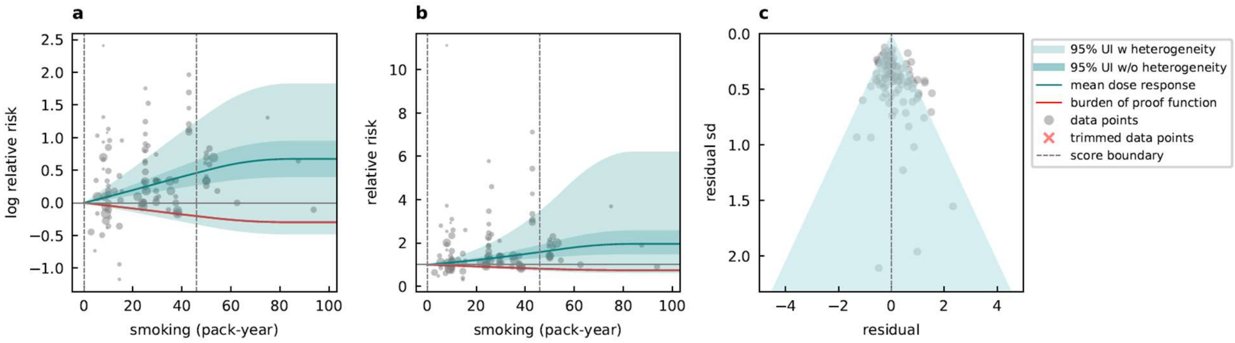

Ischemic Heart Disease

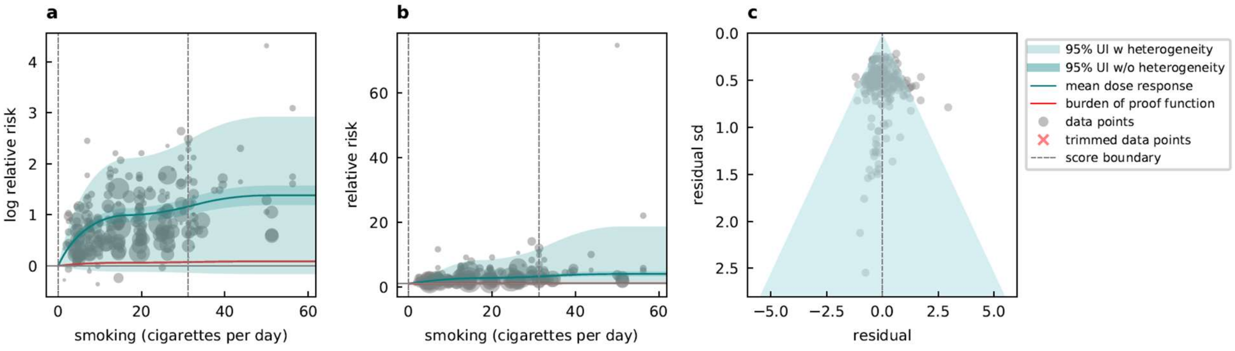

Gallbladder Diseases

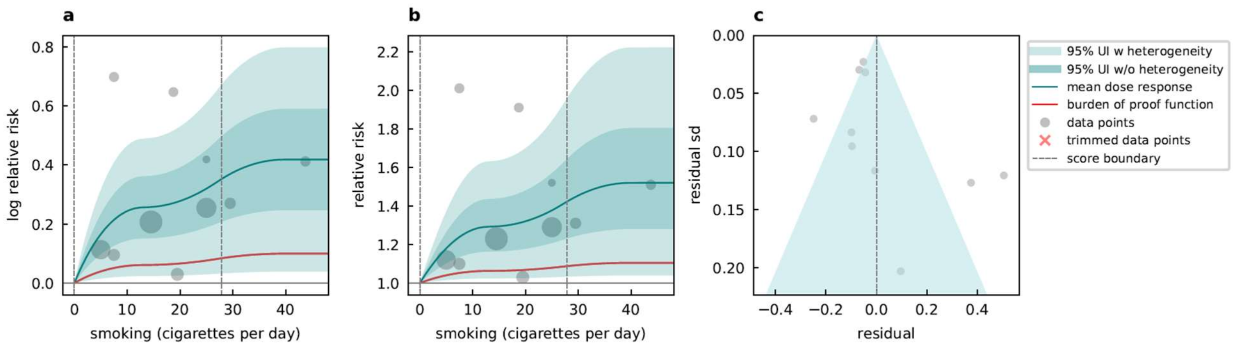

Esophageal Cancer

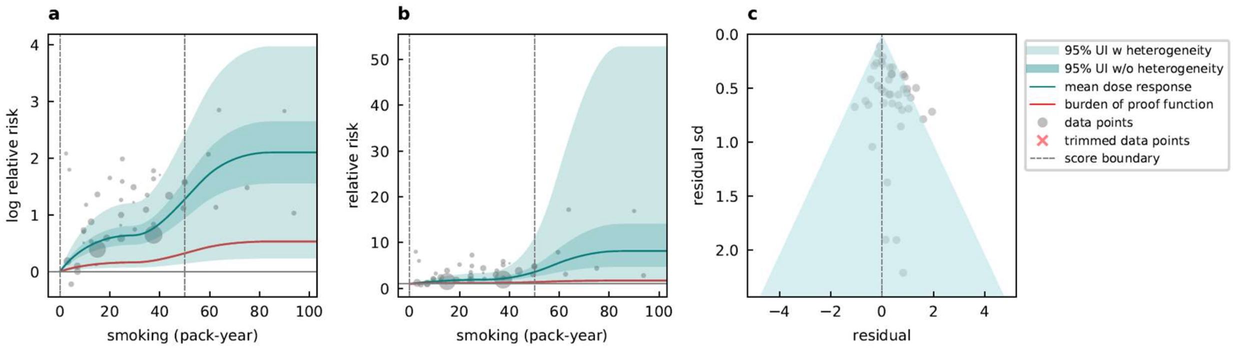

Diabetes

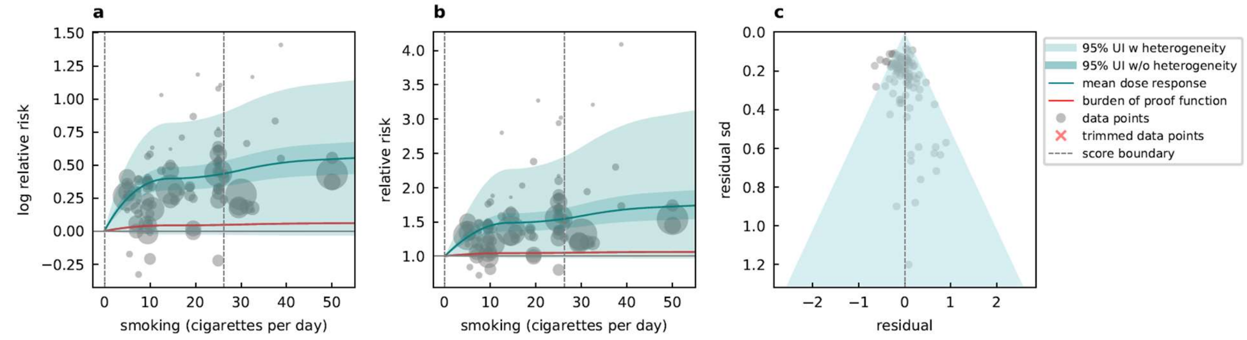

Tuberculosis

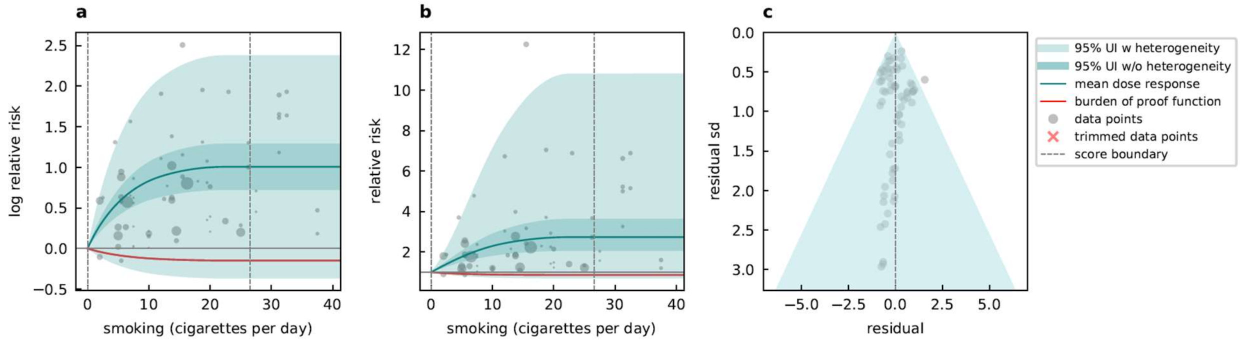

Stroke

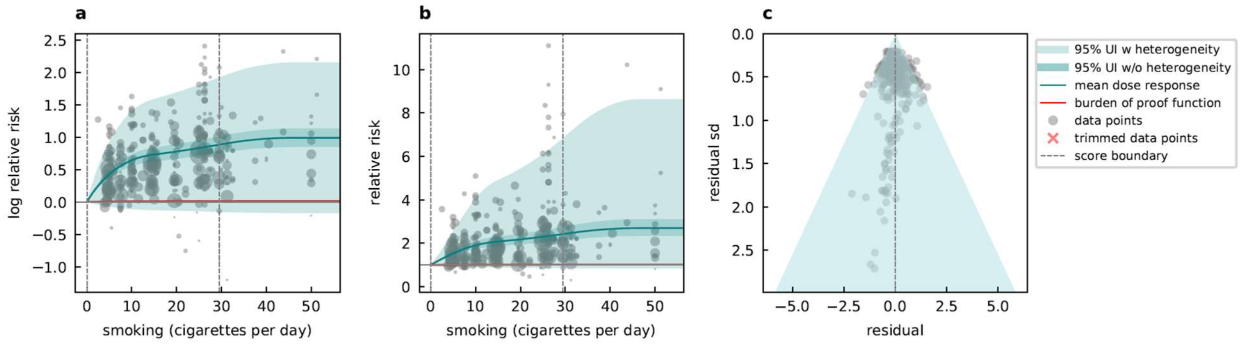

Stomach Cancer

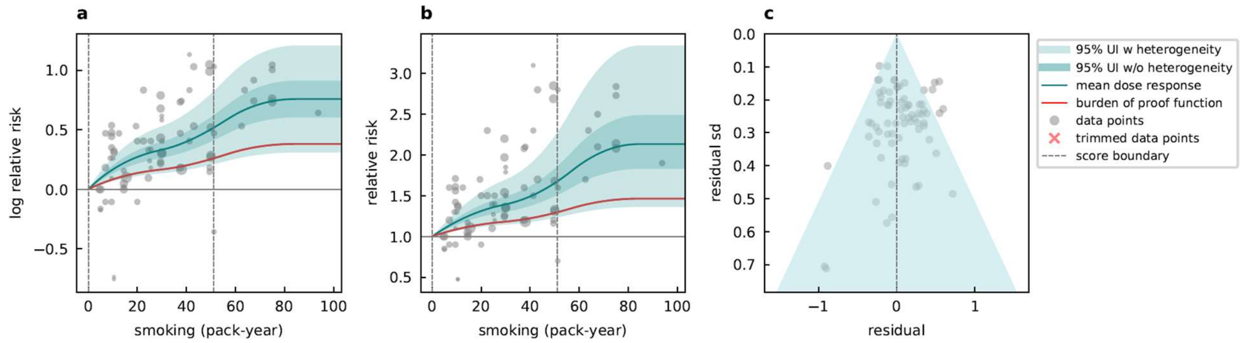

Rheumatoid Arthritis

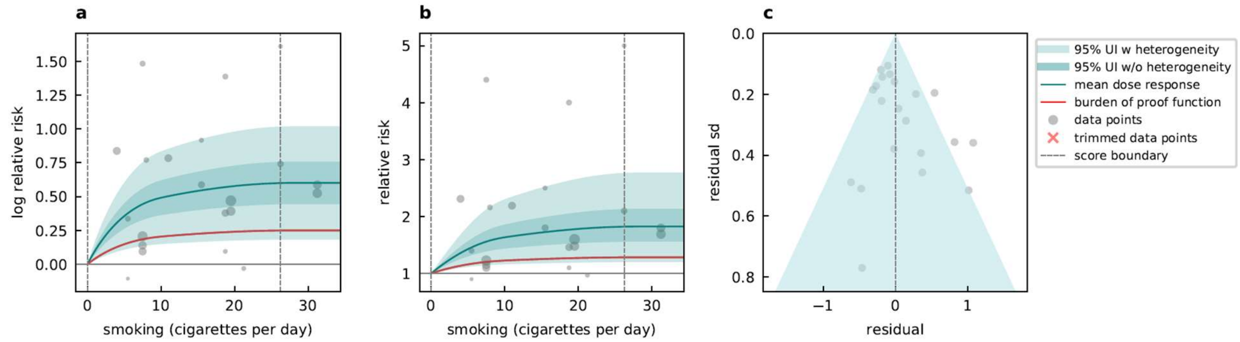

Prostate Cancer

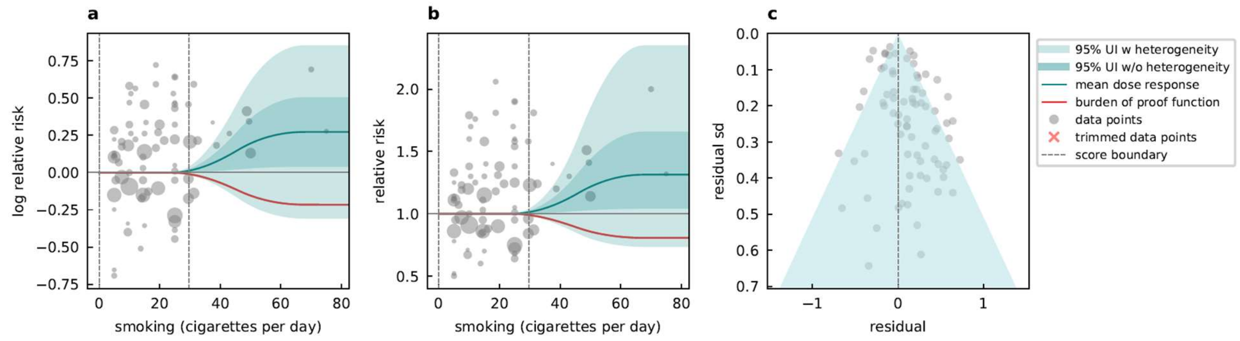

Peripheral Artery Disease

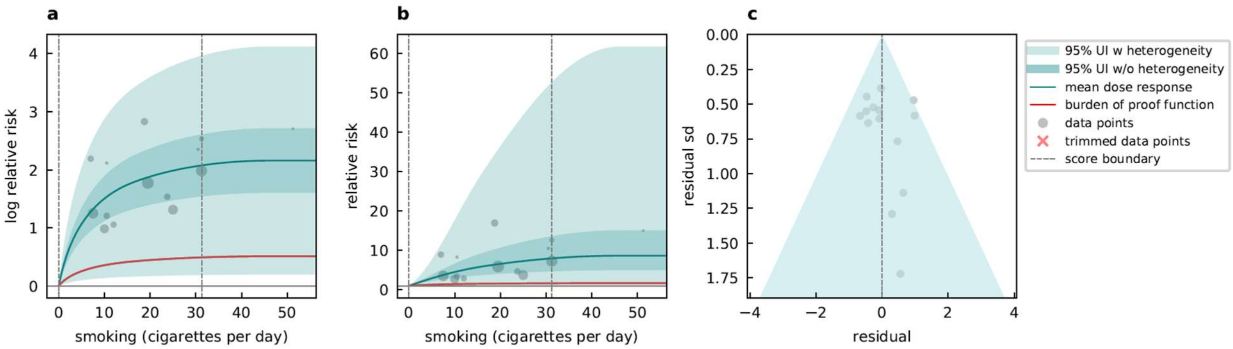

Peptic Ulcer

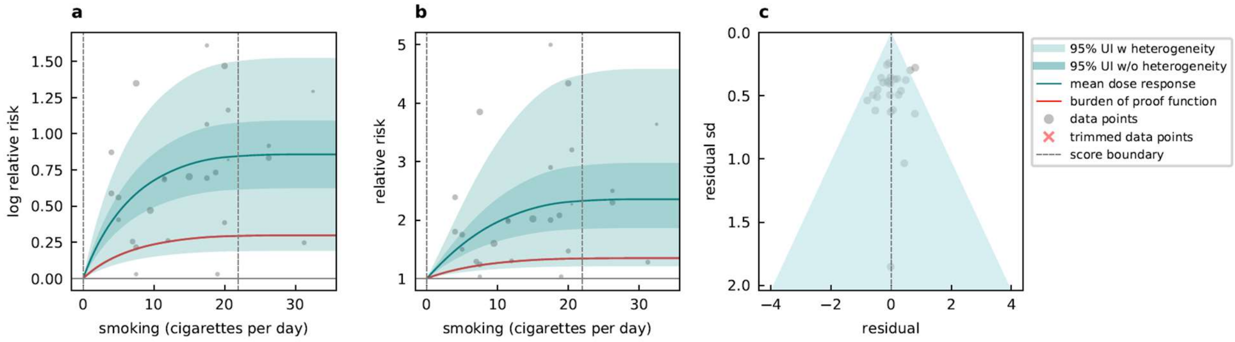

Parkinson's Disease

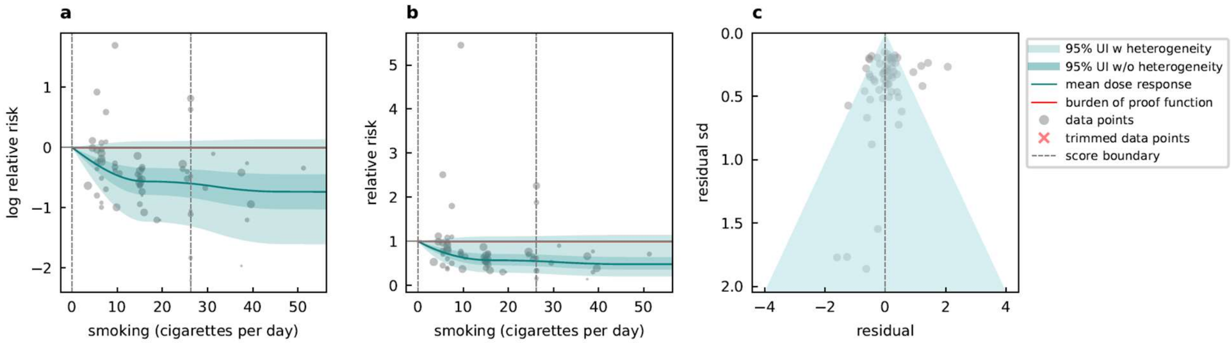

Pancreatic Cancer

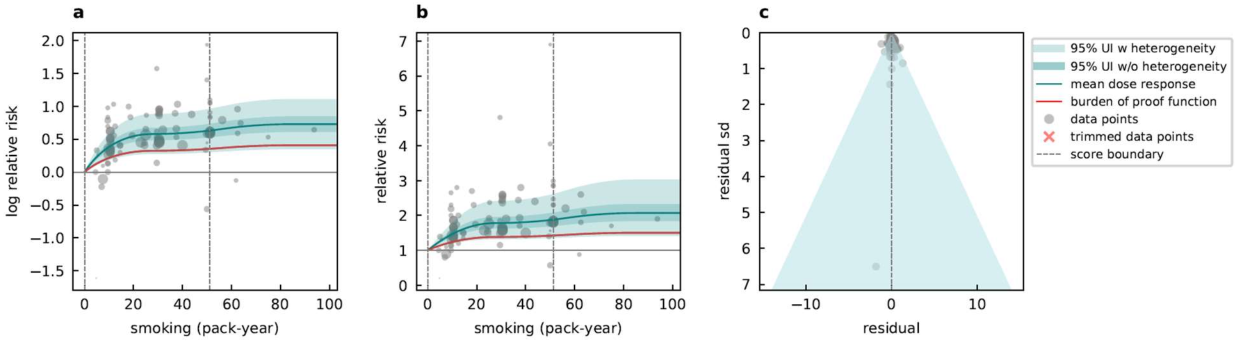

Other Pharynx Cancer

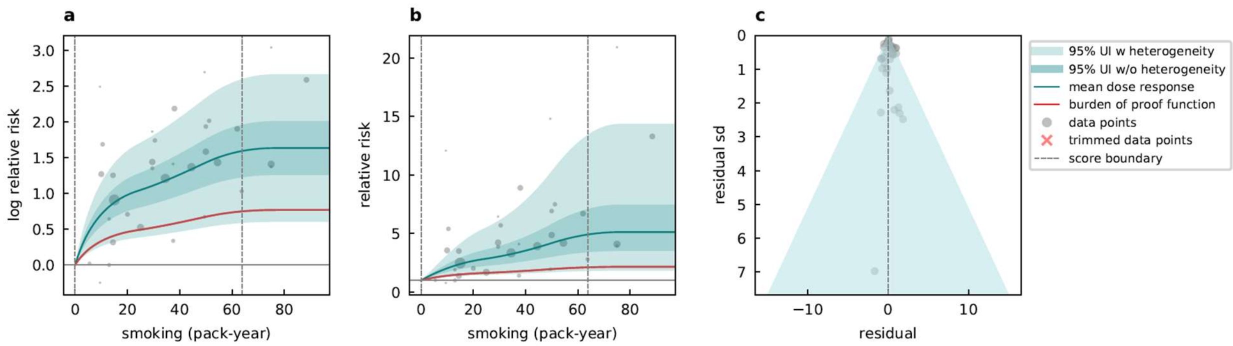

Nasopharyngeal Cancer

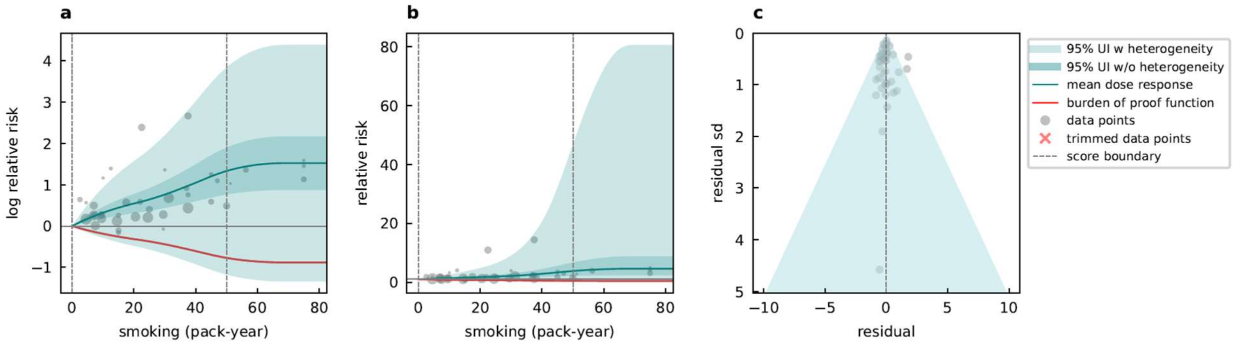

Multiple Sclerosis

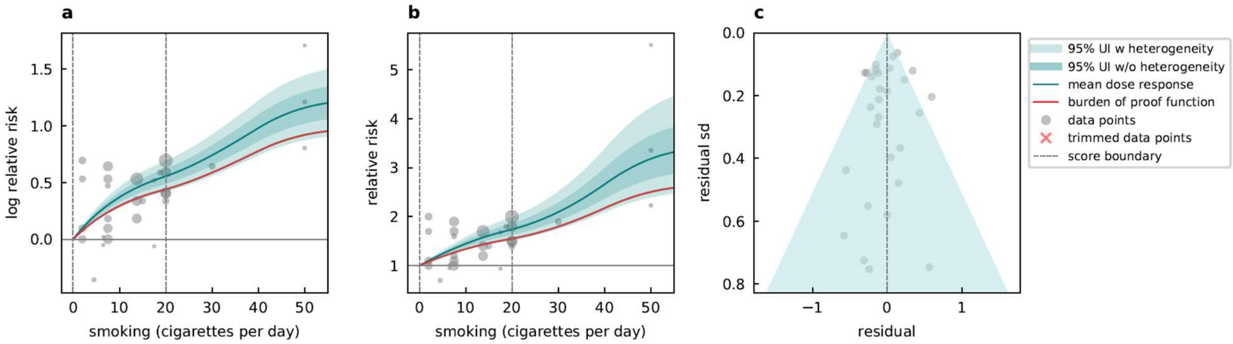

Macular Degeneration

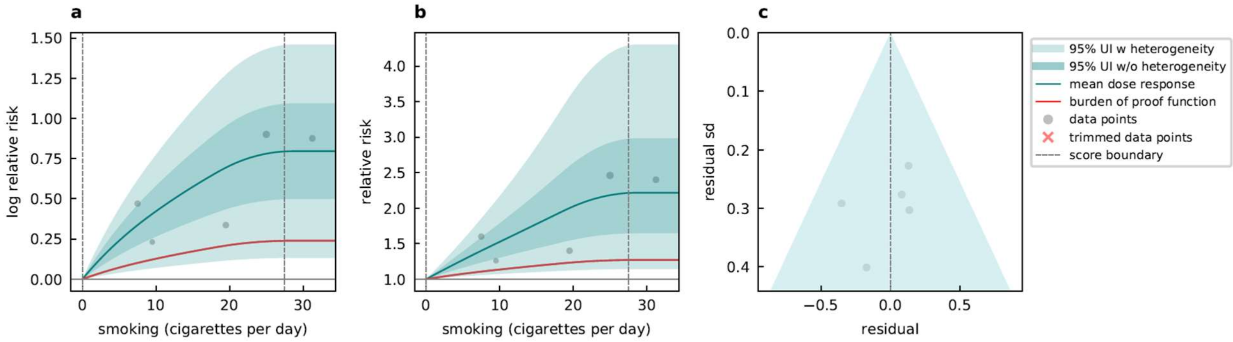

Section 6.2: Testing sensitivity of monotonicity constraints

We examined the impact of the monotonicity constraint on the shape of the risk curves for each risk-outcome pair to evaluate the sensitivity of our final model to the use of a monotonicity constraint. This constraint ensured that the mean risk curve was non-decreasing for risk-outcome pairs where the relationship is expected to be harmful and

non-increasing for Parkinson's disease-smoking pair. A preliminary model was run with the same inputs and model specifications as the final models but without the monotonicity constraint, and the results were compared to those of the final models. These results can be found below.

Figure 6: Results of model without monotonicity constraints

The figures below reflect the results of the model without monotonicity constrains using the final model specifications for every risk-outcome pair. The first of each set of outcome plots presents the dose-response relative risk in log space, while the middle figure is the dose-response relative risk in linear space. The right-most figure reflects the residual cone for all of the data inputs in the risk-outcome pair. The reference exposure for all of these risk curves is the theoretical minimum risk exposure level, 0 cigarettes per day or 0 pack-years (depending on the outcome in question).

COPD

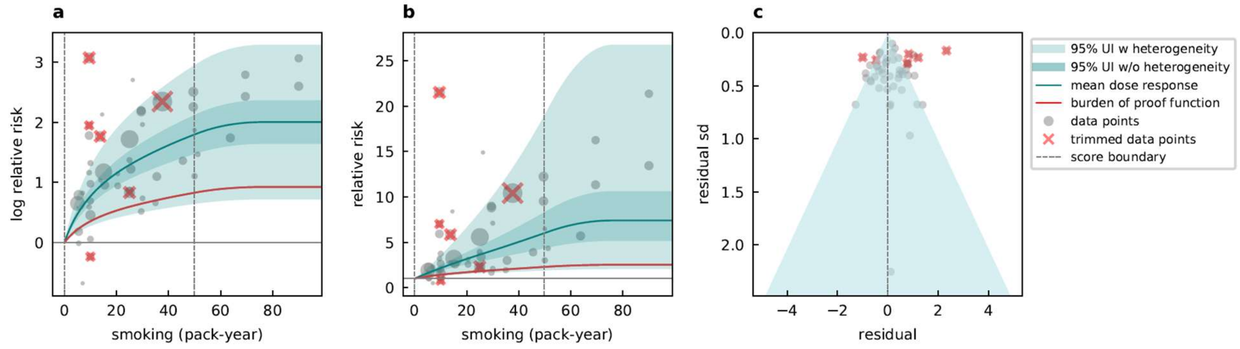

Colon and Rectum Cancer

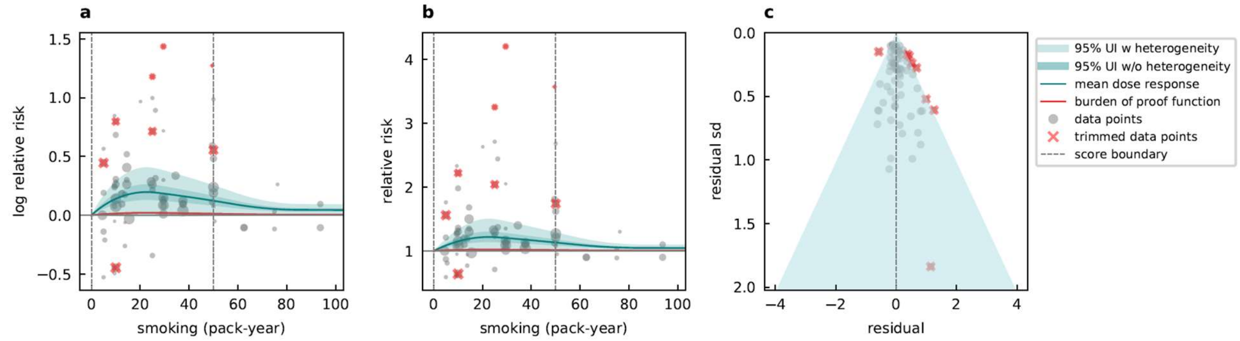

Cervical Cancer

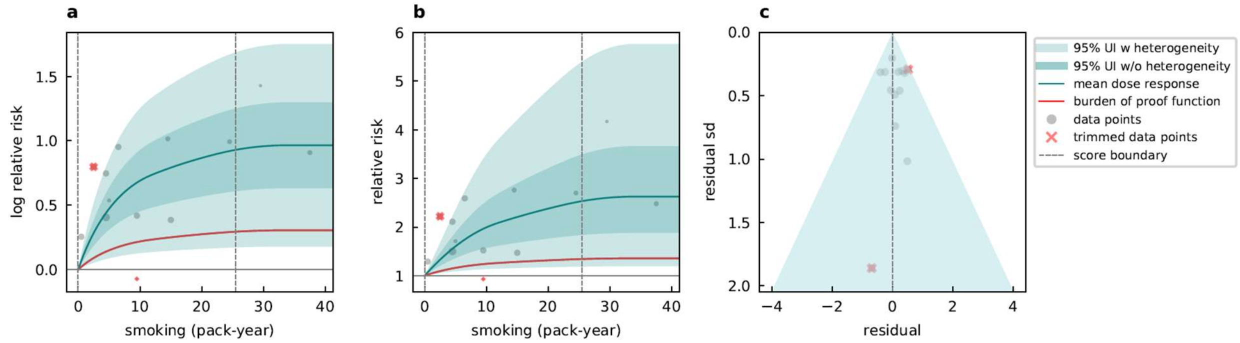

Cataracts

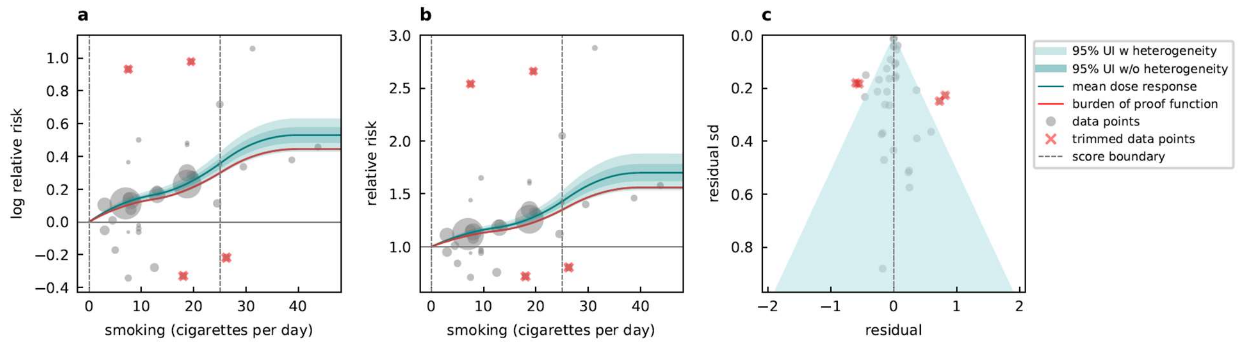

Breast Cancer

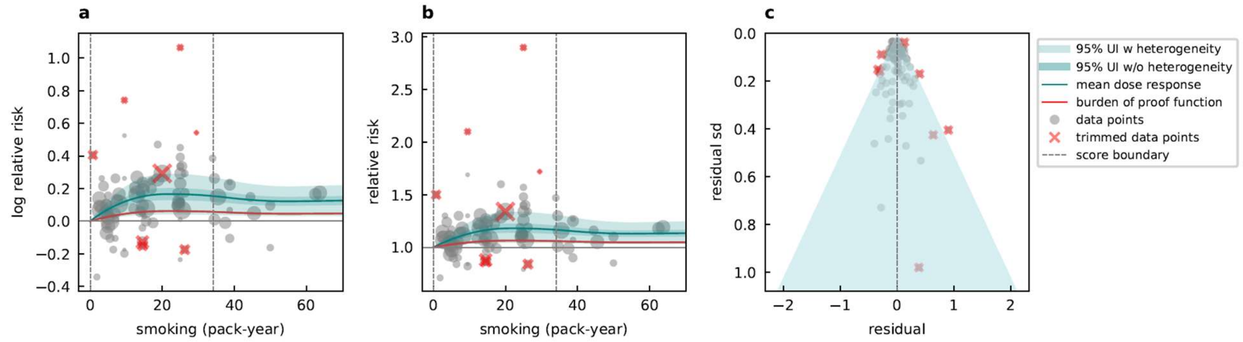

Bladder Cancer

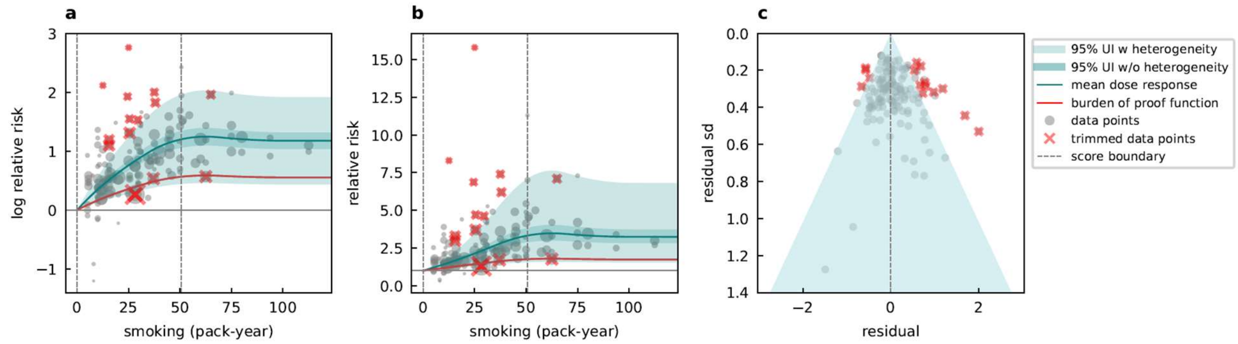

Asthma

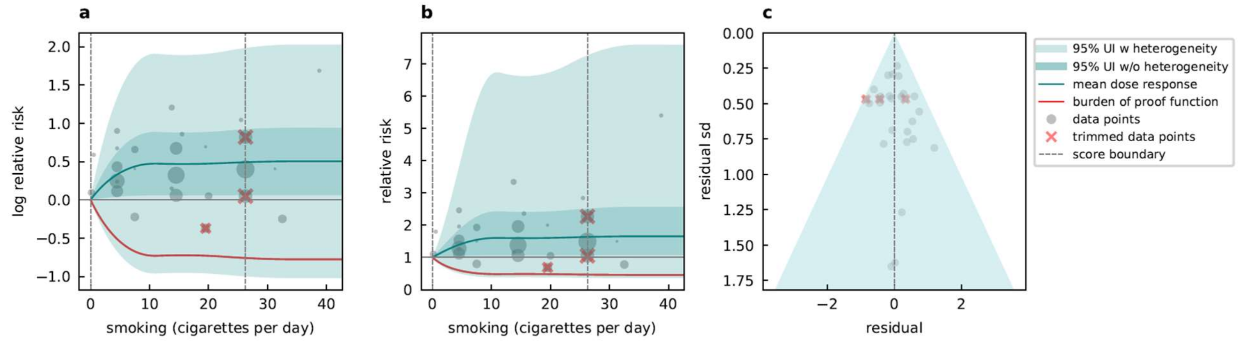

Aortic Aneurysm

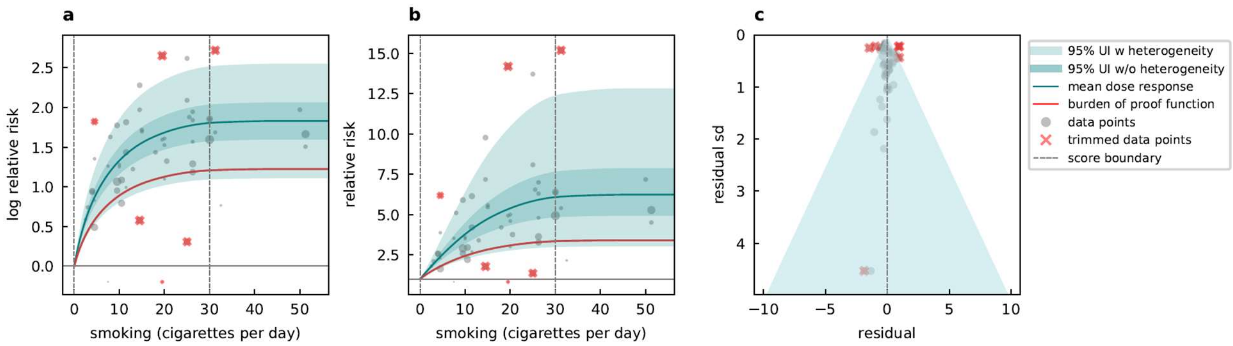

Atrial Fibrillation and Flutter

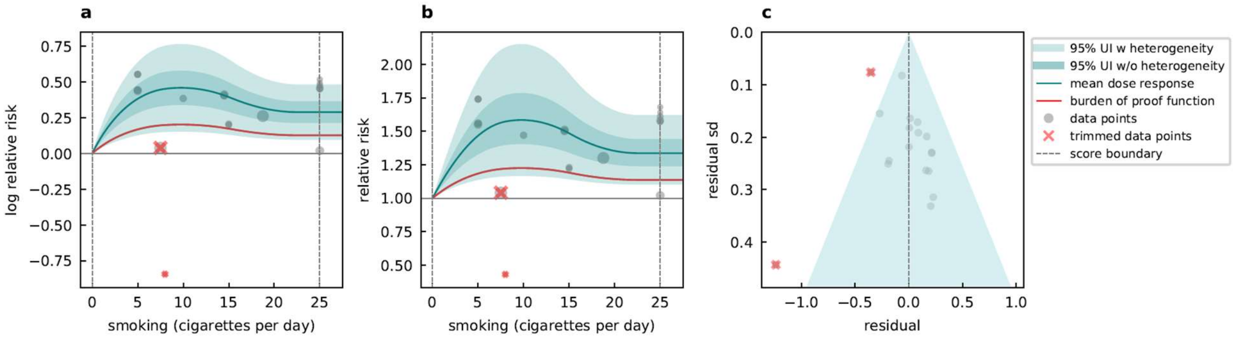

Alzheimer's and Other Dementia

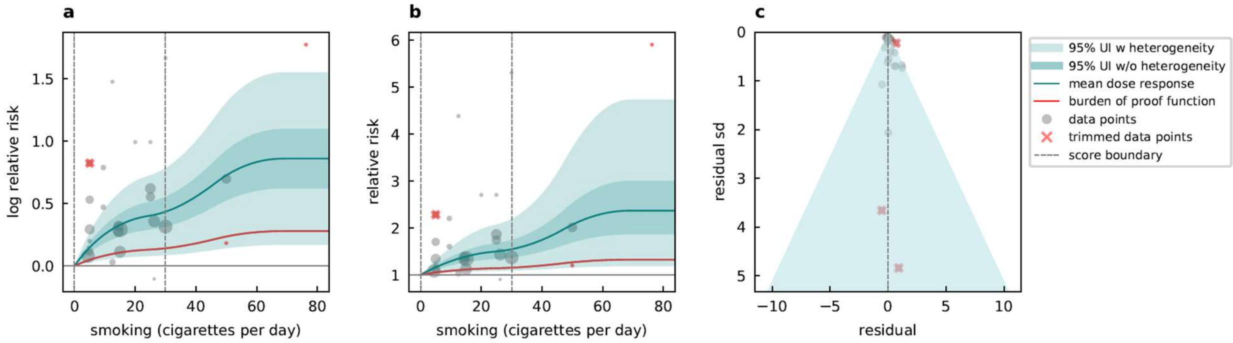

Lung Cancer

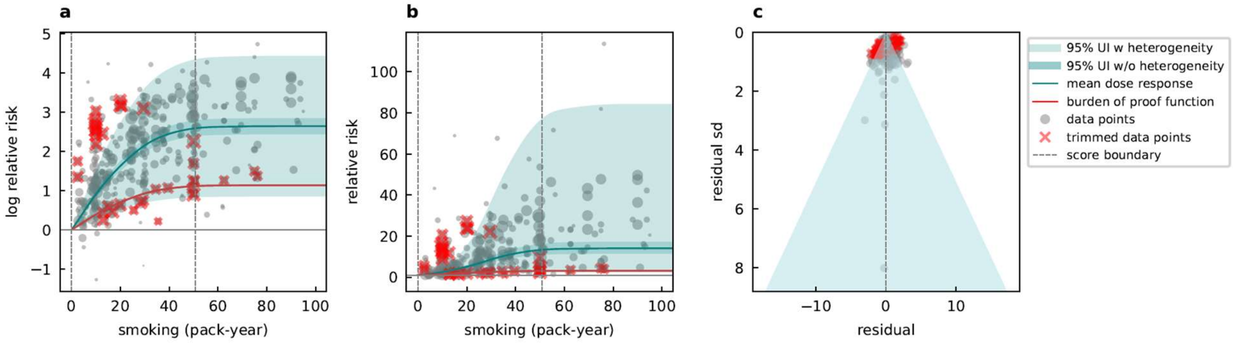

Lower Respiratory Infections

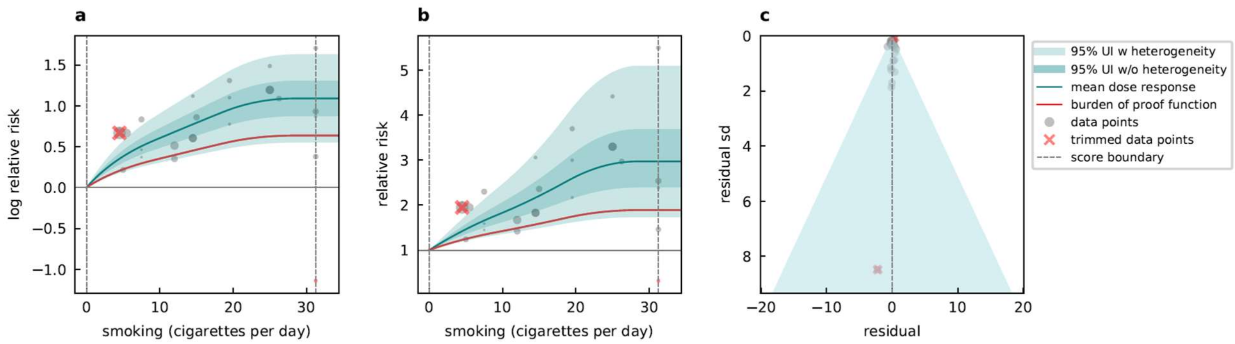

Liver Cancer

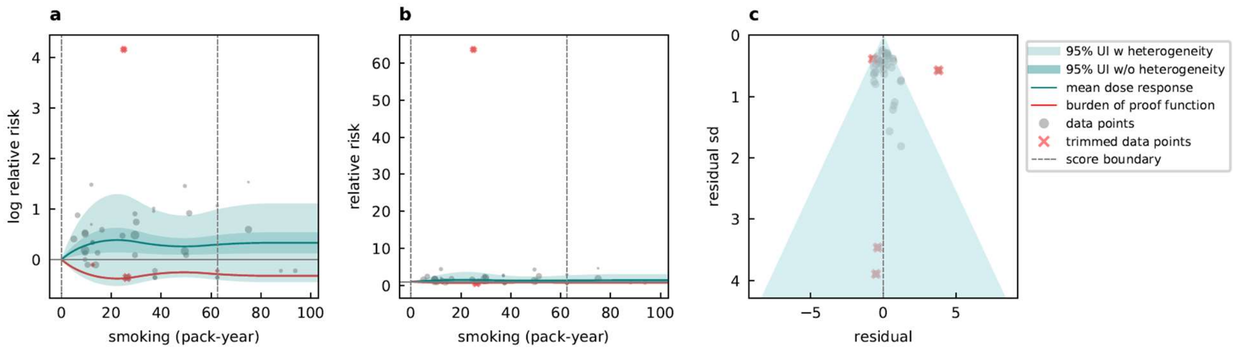

Lip and Oral Cavity Cancer

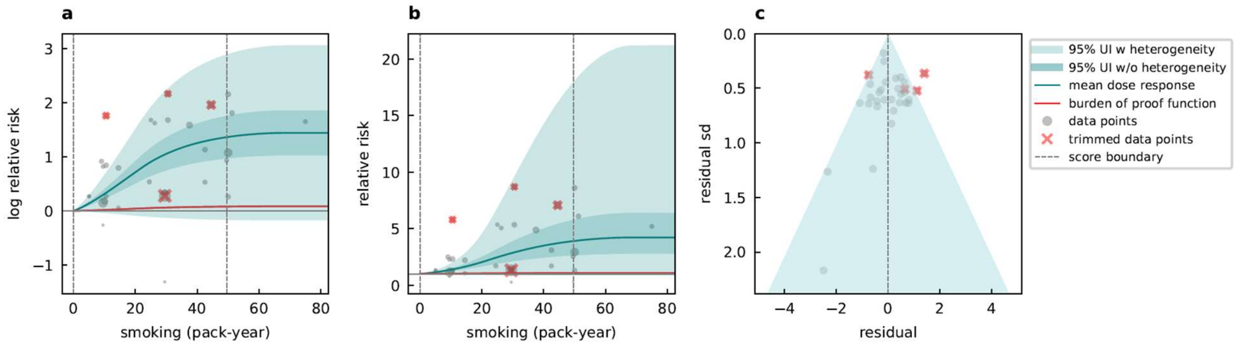

Leukemia

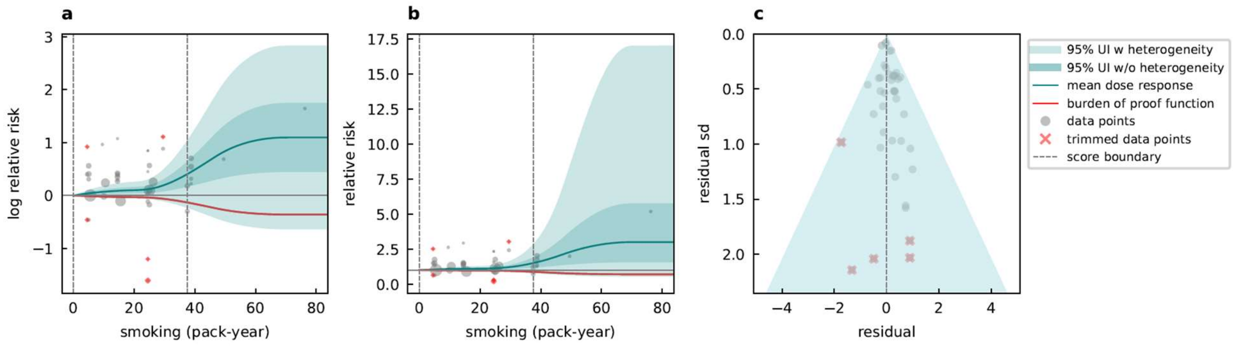

Low Back Pain

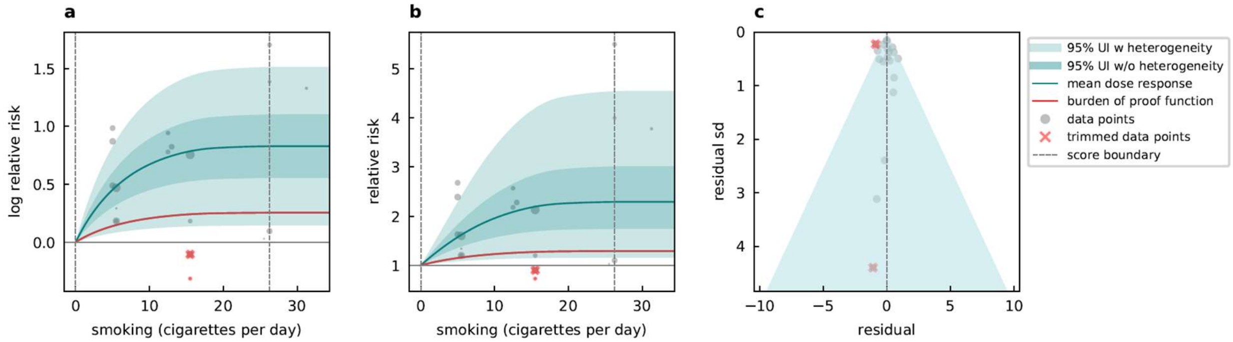

Laryngeal Cancer

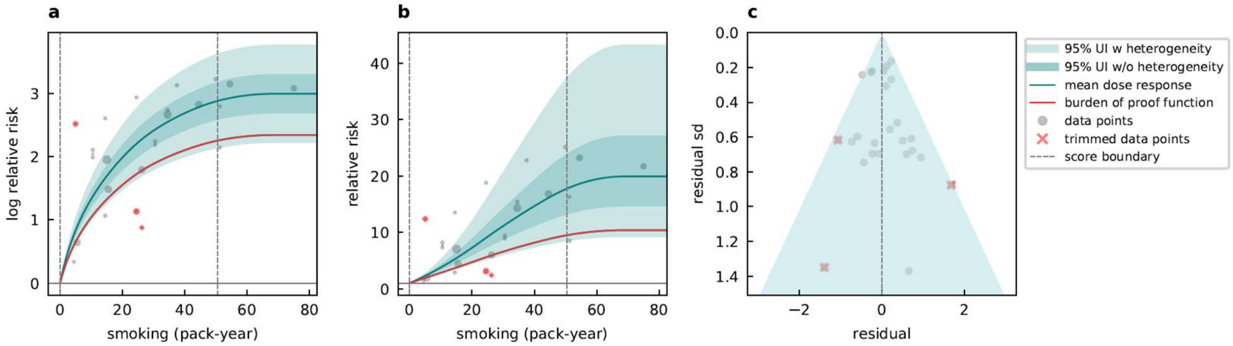

Kidney Cancer

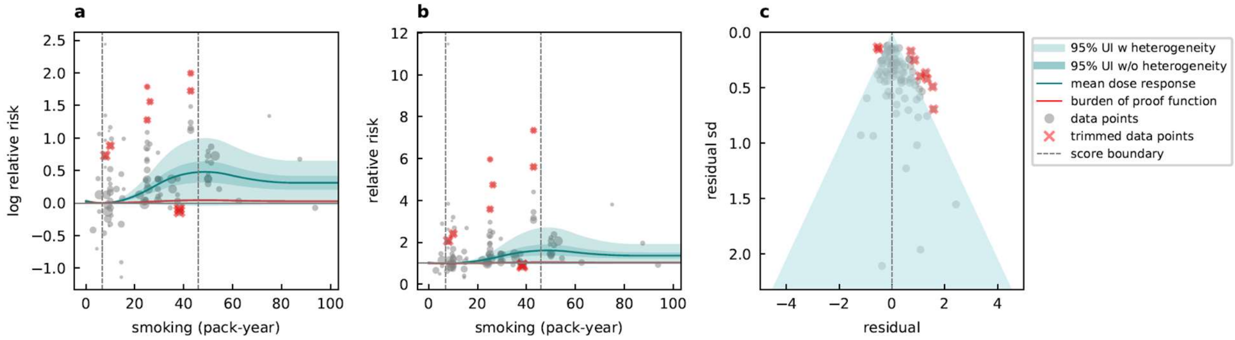

Ischemic Heart Disease

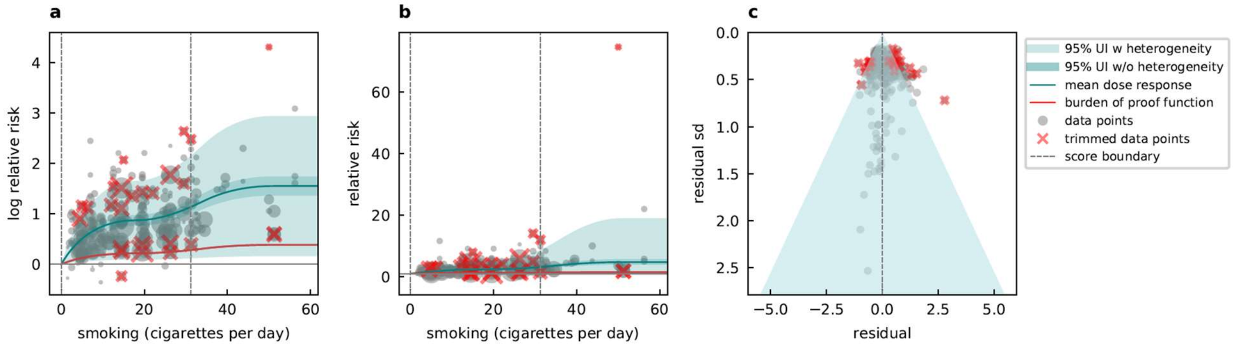

Gallbladder Diseases

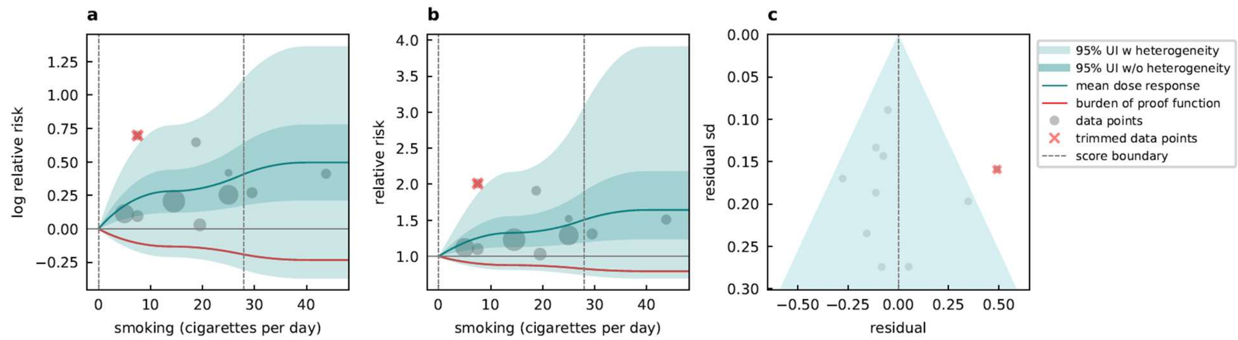

Esophageal Cancer

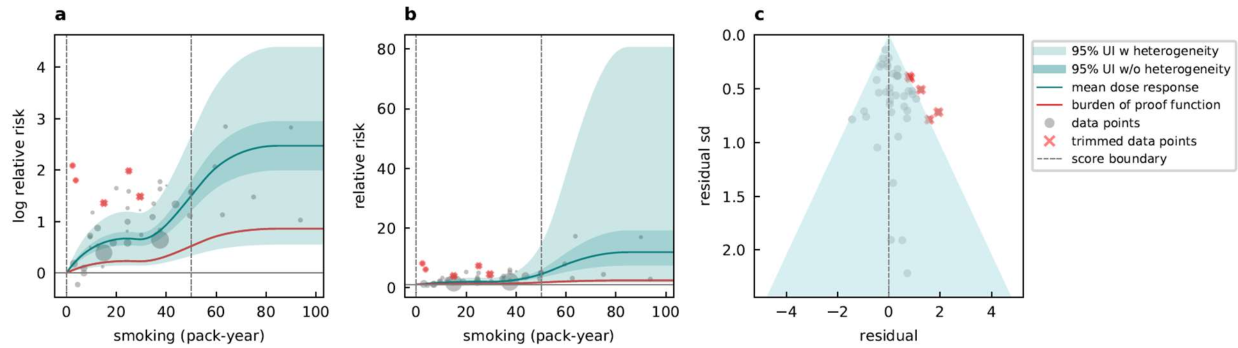

Diabetes

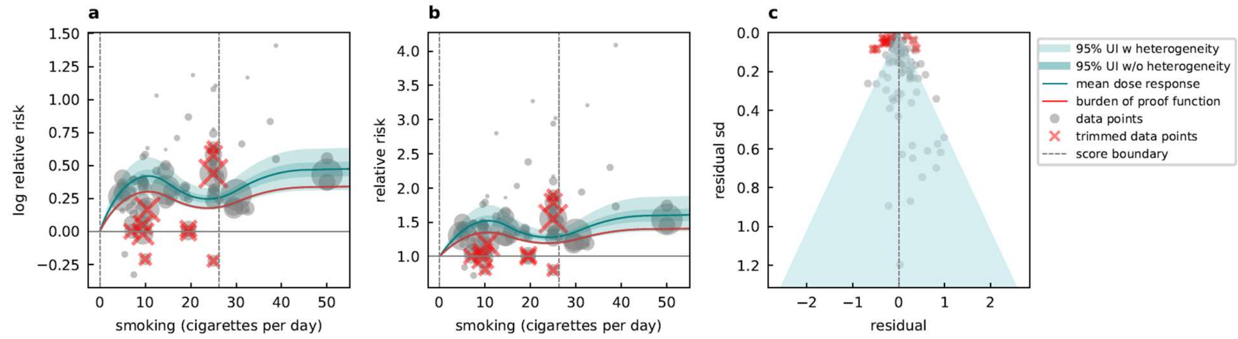

Tuberculosis

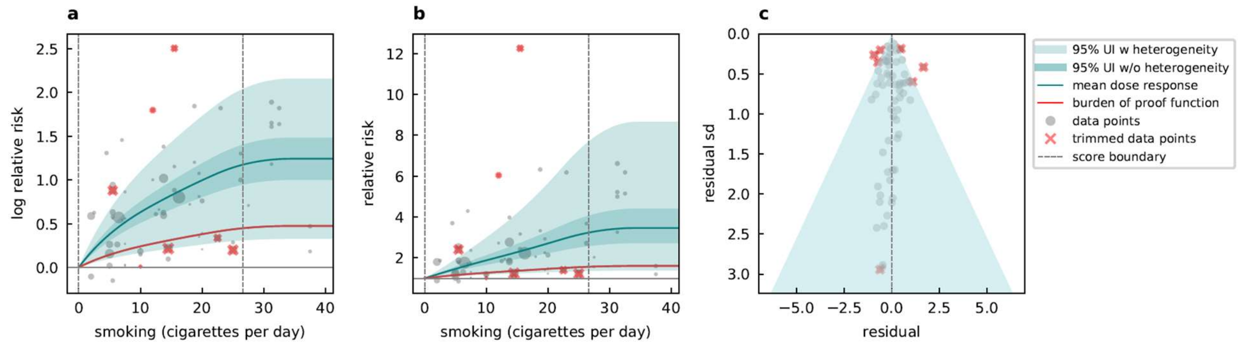

Stroke

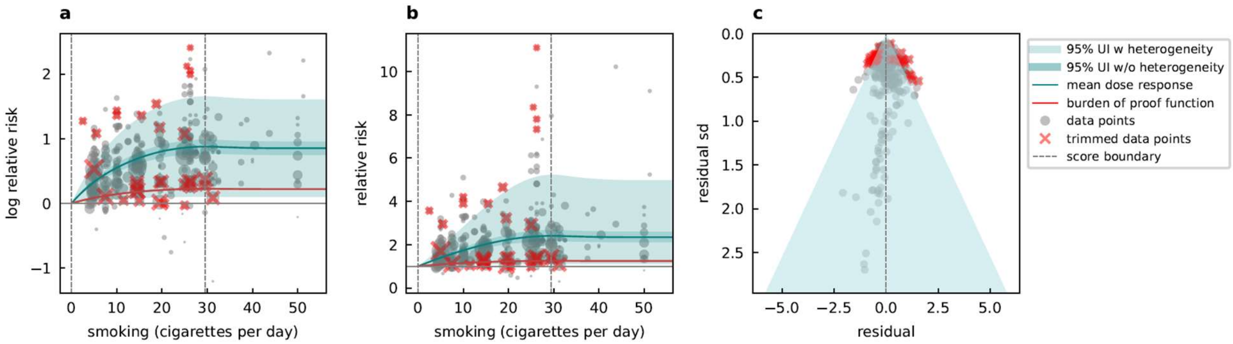

Stomach Cancer

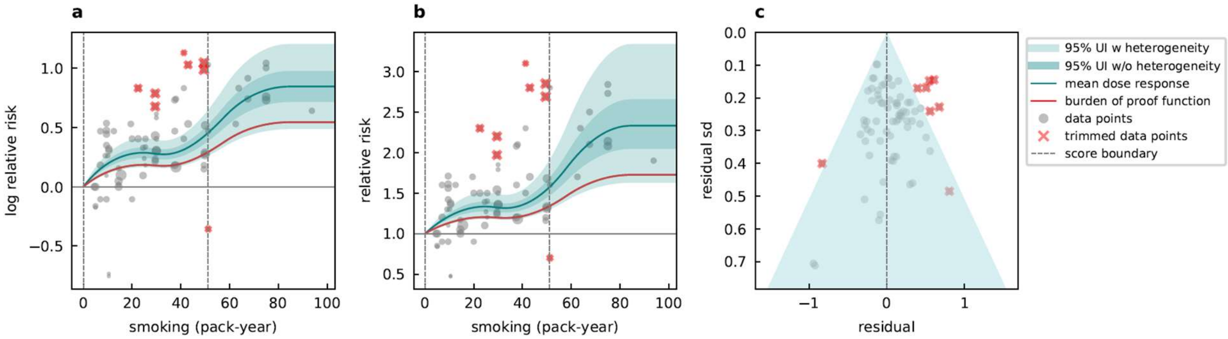

Rheumatoid Arthritis

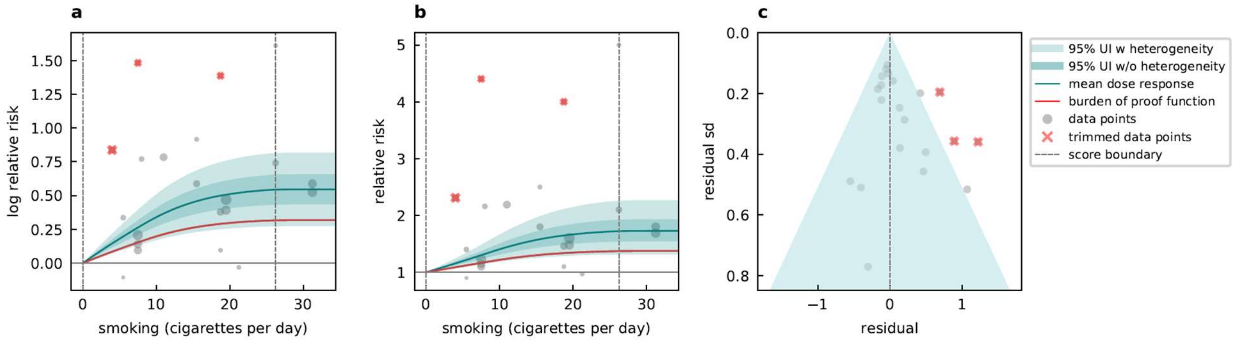

Prostate Cancer

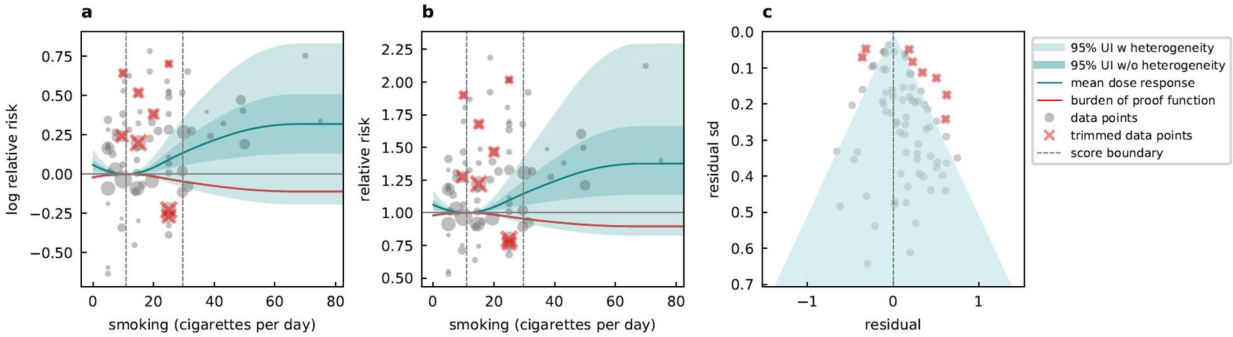

Peripheral Artery Disease

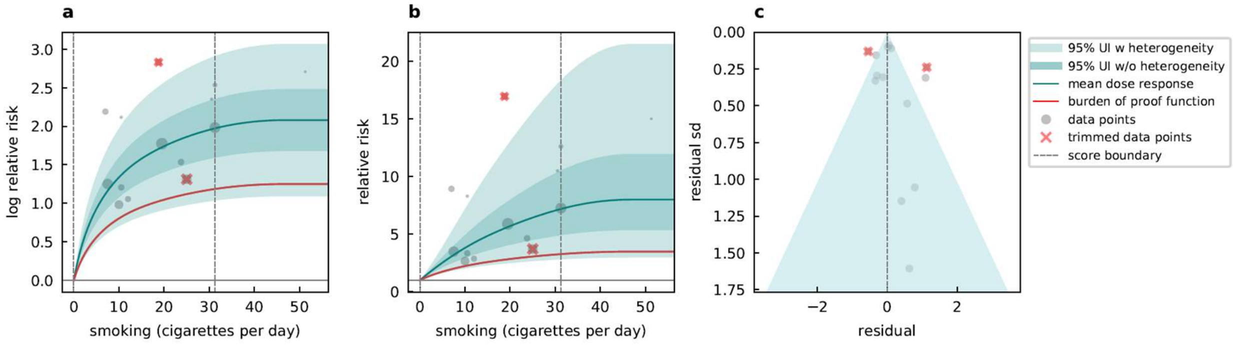

Peptic Ulcer

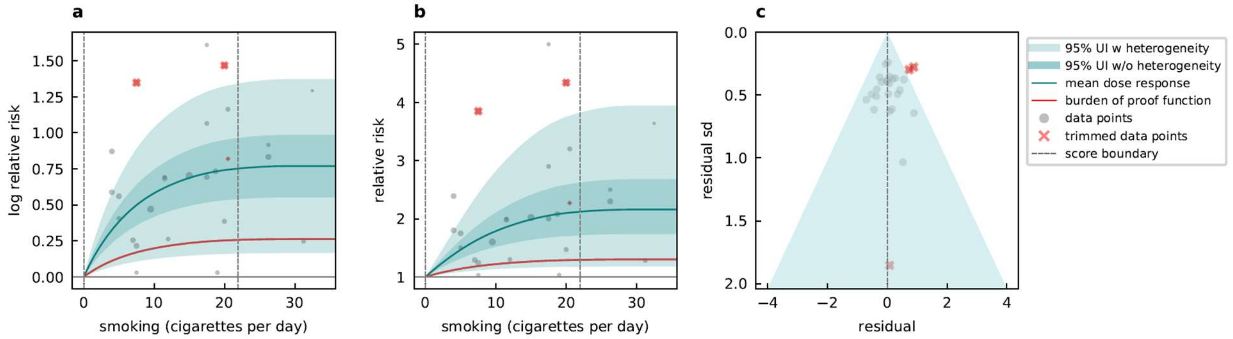

Parkinson’s Disease

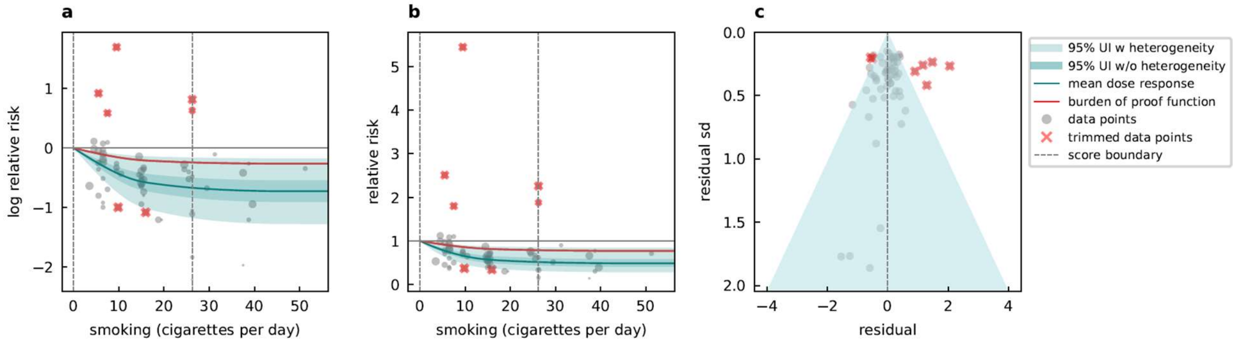

Pancreatic Cancer

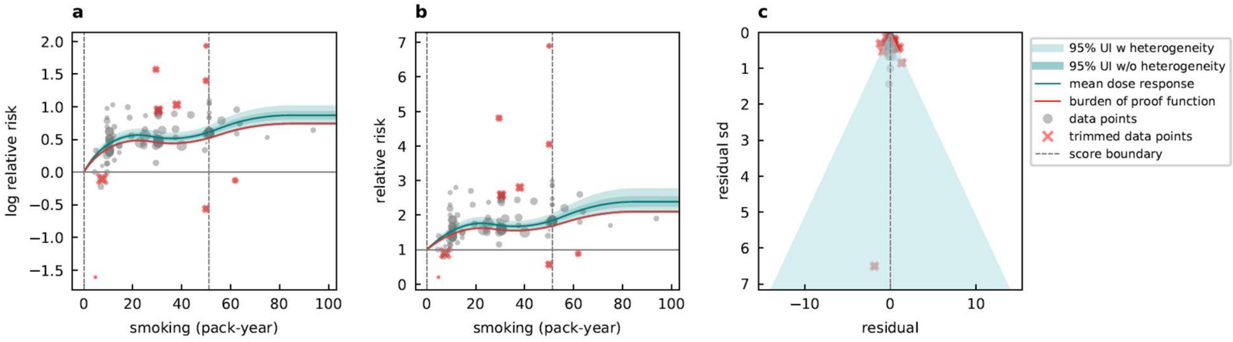

Other Pharynx Cancer

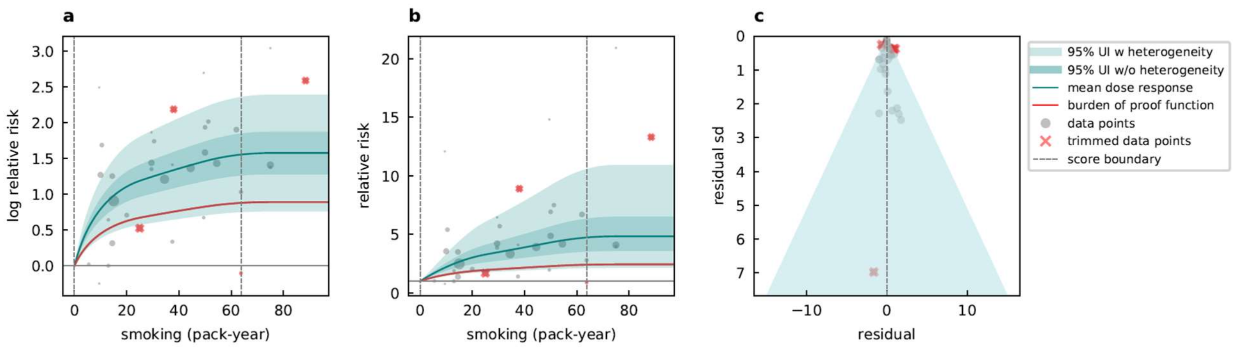

Nasopharyngeal Cancer

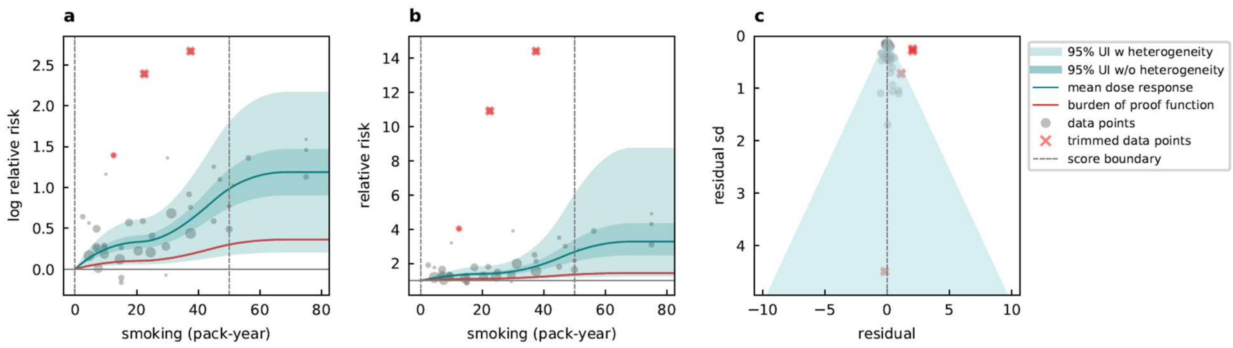

Multiple Sclerosis

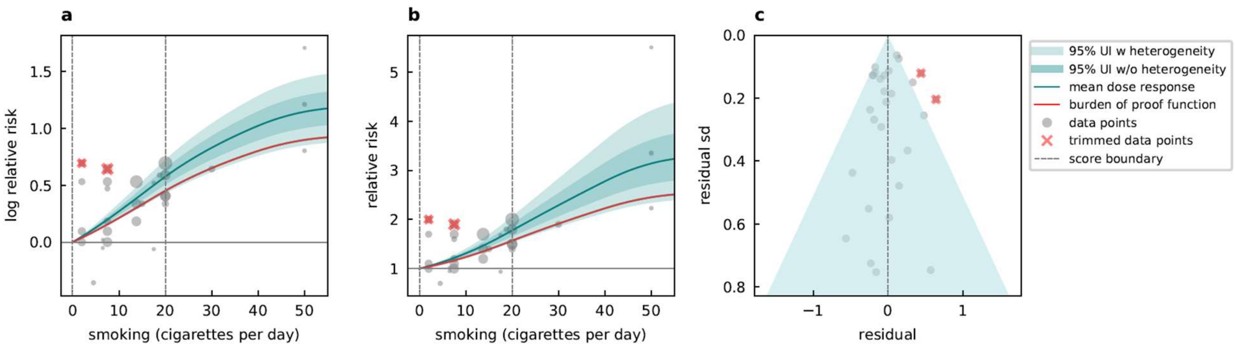

Macular Degeneration

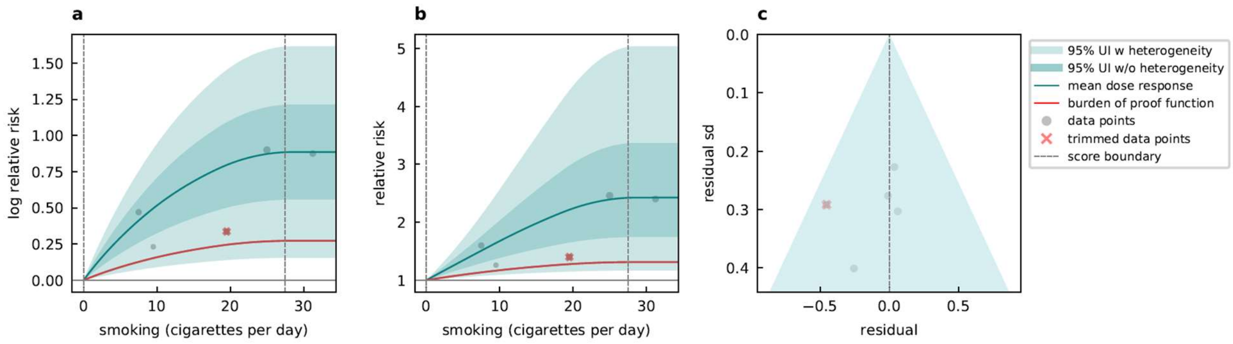

Section 7: Binary smoking-outcome pair (i.e., smoking-fracture)

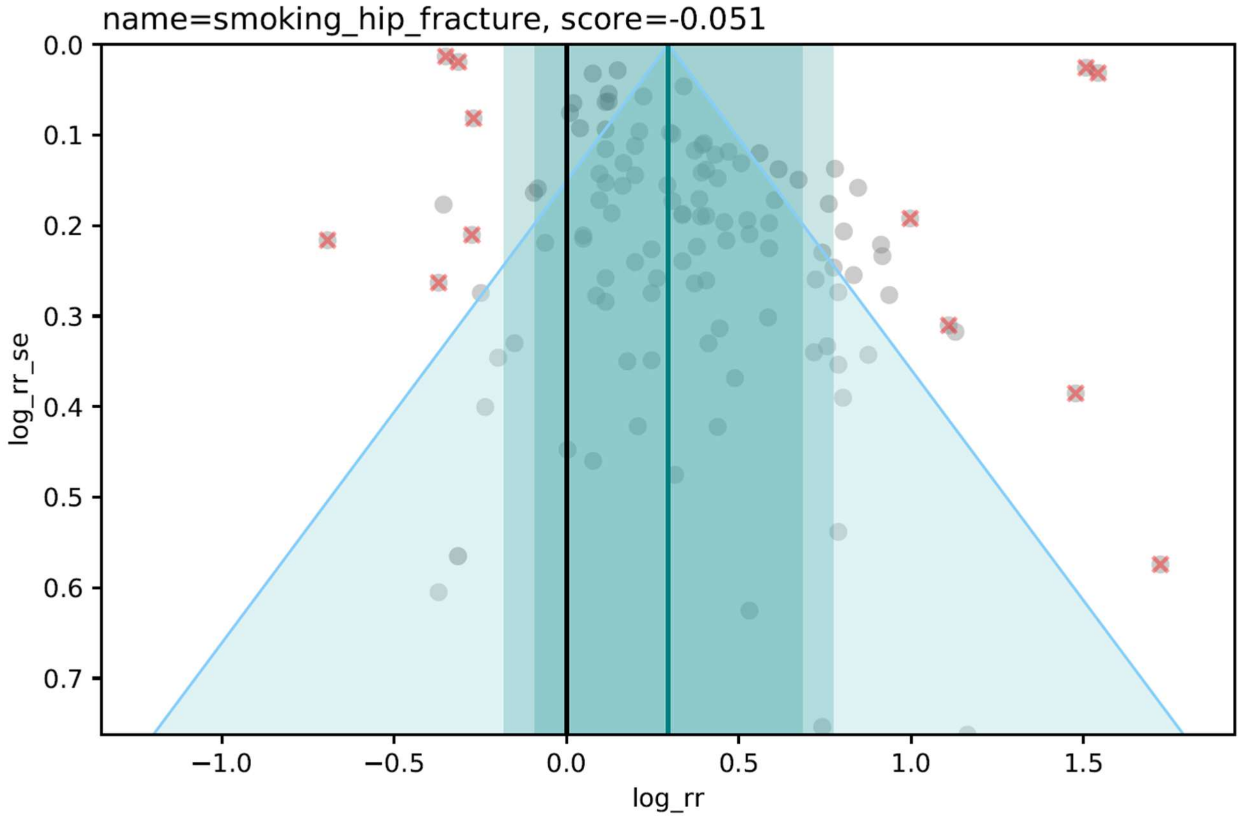

Figure 7: Natural log of relative risk of smoking (current smoker vs. never-smoker) on fracture.

Note: This is a funnel plot that shows the natural log of relative risk on the x-axis and the estimated standard error (SE) of natural log of relative risk that includes both reported SE and between-study heterogeneity on the y-axis. Outliers are denoted using red x marks in the plot. The light green and dark green shade areas represent the 95% confidence intervals of the mean natural log of risk accounting for and not accounting for between study heterogeneity, respectively.

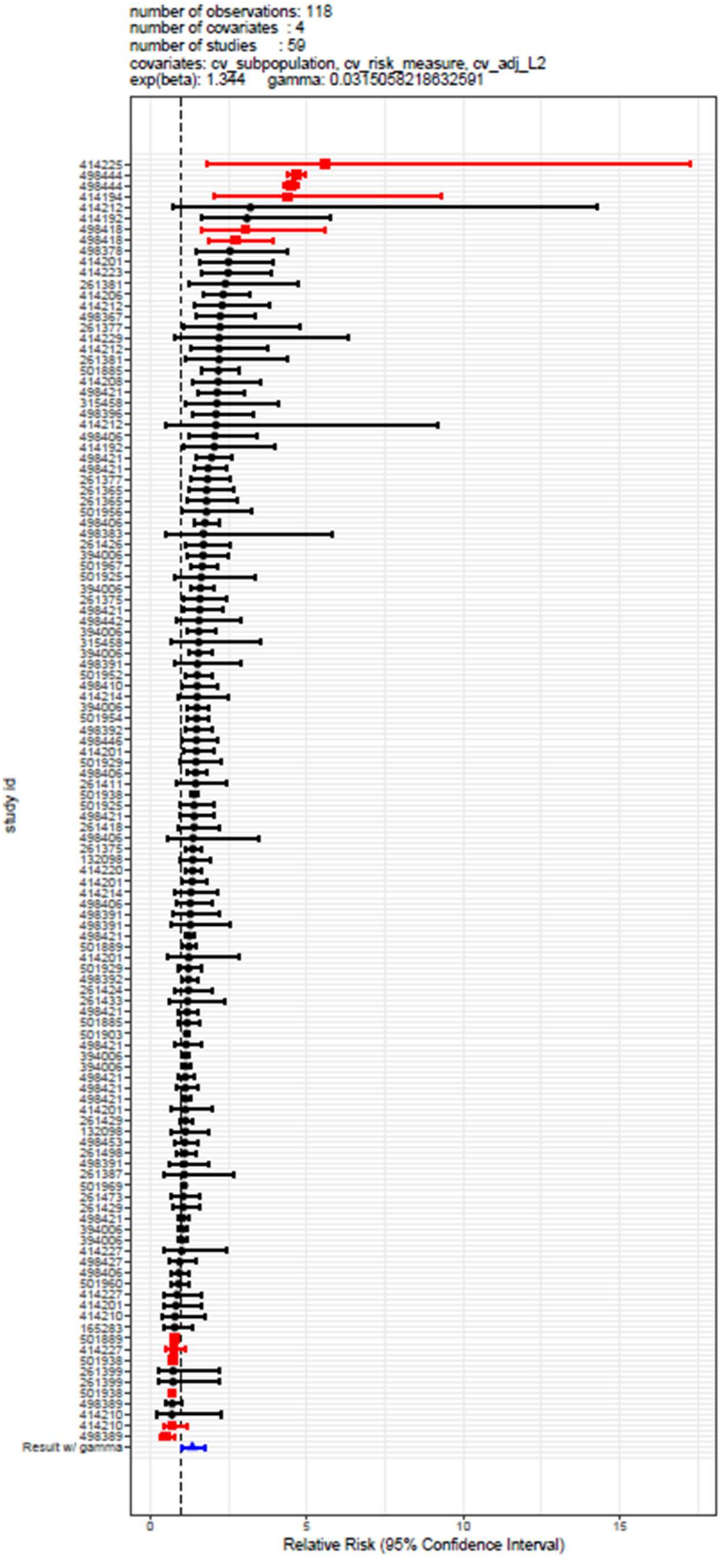
number of observations: 118
number of covariates : 4
number of studies : 59
covariates: cv_subpopulation, cv_risk_measure, cv_adj_L2
exp(beta): 1.344 gamma: 0.0315058218632591
study id
Result w/ gamma
0
5
10
15
Relative Risk (95% Confidence Interval)

Figure 8: The forest plot of relative risk of smoking on fracture.

Note: The red estimates are trimmed by MR-BRT, the black estimates are included in the final model, and the blue estimate is the pooled relative risk estimate of smoking on fracture. The whiskers around the data points are the 95% confidence intervals of the risk estimates.

Section 8: Risk curve details

The associated cause, reference exposure group, alternative exposure group, and the effect size and standard error in log space for each study included in the dose-response curve estimates, excluding those used to develop the age-attenuation factor for cardiovascular curves without dose-response data, are listed below.

Table 8: Summary of data inputs

Cause	NID	Outlier flag	Reference exposure	Alternative exposure	Log relative risk	Log standard error
afib_and_flutter	324155	0	0 cigarettes per day	10 to 19 cigarettes per day	0.405465	0.181973
afib_and_flutter	324155	0	0 cigarettes per day	10 to 19 cigarettes per day	0.41211	0.163873
afib_and_flutter	324155	0	0 cigarettes per day	1 to 9 cigarettes per day	0.438255	0.171165
afib_and_flutter	324155	0	0 cigarettes per day	1 to 9 cigarettes per day	0.444686	0.191327
afib_and_flutter	324155	0	0 cigarettes per day	20 to 30 cigarettes per day	0.494696	0.331322
afib_and_flutter	324155	0	0 cigarettes per day	20 to 30 cigarettes per day	0.518794	0.314322
afib_and_flutter	324168	0	0 cigarettes per day	1 to 19 cigarettes per day	0.385262	0.218659
afib_and_flutter	324168	0	0 cigarettes per day	20 to 30 cigarettes per day	0.457425	0.198592
afib_and_flutter	350084	0	0 cigarettes per day	0 to 10 cigarettes per day	0.553885	0.230178
afib_and_flutter	350084	0	0 cigarettes per day	0 to 10 cigarettes per day	0.553885	0.228712
afib_and_flutter	350084	0	0 cigarettes per day	20 to 30 cigarettes per day	0.476234	0.264609
afib_and_flutter	350084	0	0 cigarettes per day	10 to 20 cigarettes per day	0.198851	0.25092
afib_and_flutter	350084	0	0 cigarettes per day	10 to 20 cigarettes per day	0.207014	0.244732
afib_and_flutter	350084	0	0 cigarettes per day	20 to 30 cigarettes per day	0.451076	0.263226
afib_and_flutter	350091	1	0 cigarettes per day	1 to 15 cigarettes per day	-0.84397	0.443166
afib_and_flutter	350091	0	0 cigarettes per day	20 to 30 cigarettes per day	0.019803	0.154812
afib_and_flutter	357504	1	0 cigarettes per day	1 to 14 cigarettes per day	0.039221	0.07604
afib_and_flutter	357504	0	0 cigarettes per day	15 to 22.5 cigarettes per day	0.262364	0.082418
alzheimer_other_dementia	328492	0	0 cigarettes per day	10 to 15 cigarettes per day	0.029559	0.312067
alzheimer_other_dementia	328492	1	0 cigarettes per day	1 to 9 cigarettes per day	0.824175	0.226011
alzheimer_other_dementia	328517	0	0 cigarettes per day	0 to 9 cigarettes per day	0.076961	0.067555
alzheimer_other_dementia	328517	0	0 cigarettes per day	10 to 20 cigarettes per day	0.29267	0.051401
alzheimer_other_dementia	328517	0	0 cigarettes per day	20 to 40 cigarettes per day	0.314811	0.06331
alzheimer_other_dementia	328517	0	0 cigarettes per day	40 to 60 cigarettes per day	0.698135	0.128186
alzheimer_other_dementia	349159	0	0 cigarettes per day	21 to 31.5 cigarettes per day	-0.10536	1.074263
alzheimer_other_dementia	349159	0	0 cigarettes per day	0 to 10 cigarettes per day	0.198851	0.549933
alzheimer_other_dementia	349159	0	0 cigarettes per day	11 to 20 cigarettes per day	0.29267	0.613006
alzheimer_other_dementia	358834	0	0 cigarettes per day	0 to 19 cigarettes per day	0.788457	0.41744
alzheimer_other_dementia	358834	0	0 cigarettes per day	20 to 30 cigarettes per day	0.993252	0.68972
alzheimer_other_dementia	358836	0	0 cigarettes per day	0 to 9 cigarettes per day	0.14842	2.067206
alzheimer_other_dementia	358836	0	0 cigarettes per day	10 to 15 cigarettes per day	1.477049	0.67736
alzheimer_other_dementia	358838	1	0 cigarettes per day	61 to 91.5 cigarettes per day	1.774952	4.838291

alzheimer_other_dementia	358838	0	0 cigarettes per day	20 to 40 cigarettes per day	1.667707	0.731613
alzheimer_other_dementia	358838	1	0 cigarettes per day	40 to 60 cigarettes per day	0.182322	3.656463
alzheimer_other_dementia	358838	0	0 cigarettes per day	0 to 19 cigarettes per day	0.470004	0.398597
alzheimer_other_dementia	358838	0	0 cigarettes per day	20 to 20 cigarettes per day	0.993252	0.699169
alzheimer_other_dementia	501943	0	0 cigarettes per day	1 to 9 cigarettes per day	0.29267	0.1205
alzheimer_other_dementia	501943	0	0 cigarettes per day	10 to 20 cigarettes per day	0.113329	0.086609
alzheimer_other_dementia	501943	0	0 cigarettes per day	21 to 31.5 cigarettes per day	0.357674	0.085232
alzheimer_other_dementia	502162	0	0 cigarettes per day	10 to 19 cigarettes per day	0.270027	0.125771
alzheimer_other_dementia	502162	0	0 cigarettes per day	1 to 9 cigarettes per day	0.113329	0.196207
alzheimer_other_dementia	502162	0	0 cigarettes per day	10 to 19 cigarettes per day	0.322083	0.12458
alzheimer_other_dementia	502162	0	0 cigarettes per day	20 to 30 cigarettes per day	0.553885	0.141652
alzheimer_other_dementia	502162	0	0 cigarettes per day	1 to 9 cigarettes per day	0.530628	0.17894
alzheimer_other_dementia	502162	0	0 cigarettes per day	20 to 30 cigarettes per day	0.620576	0.105119
aortic_aneurism	173863	0	0 cigarettes per day	1 to 14 cigarettes per day	1.261298	1.052278
aortic_aneurism	173863	0	0 cigarettes per day	15 to 24 cigarettes per day	1.595339	0.753457
aortic_aneurism	173863	0	0 cigarettes per day	25 to 37.5 cigarettes per day	1.686399	0.687878
aortic_aneurism	236197	0	0 cigarettes per day	0 to 9 cigarettes per day	1.353255	0.95983
aortic_aneurism	236197	0	0 cigarettes per day	10 to 19 cigarettes per day	1.971299	0.517346
aortic_aneurism	236197	0	0 cigarettes per day	20 to 30 cigarettes per day	2.090629	0.459152
aortic_aneurism	328259	0	0 cigarettes per day	26 to 39 cigarettes per day	0.765468	1.864025
aortic_aneurism	328259	0	0 cigarettes per day	1 to 7 cigarettes per day	0.932164	0.347501
aortic_aneurism	328259	0	0 cigarettes per day	1 to 7 cigarettes per day	0.947789	0.326293
aortic_aneurism	328259	0	0 cigarettes per day	16 to 25 cigarettes per day	1.324419	0.411149
aortic_aneurism	328259	0	0 cigarettes per day	8 to 15 cigarettes per day	1.435085	0.309767
aortic_aneurism	328259	0	0 cigarettes per day	8 to 15 cigarettes per day	1.814825	0.330303
aortic_aneurism	331705	1	0 cigarettes per day	10 to 19 cigarettes per day	0.576613	0.21784
aortic_aneurism	331705	0	0 cigarettes per day	0 to 9 cigarettes per day	0.48858	0.234756
aortic_aneurism	331705	1	0 cigarettes per day	20 to 30 cigarettes per day	0.307485	0.249475
aortic_aneurism	343383	0	0 cigarettes per day	10 to 20 cigarettes per day	1.719189	0.665688
aortic_aneurism	343383	0	0 cigarettes per day	21 to 30 cigarettes per day	1.879465	0.567105
aortic_aneurism	343424	0	0 cigarettes per day	1 to 19 cigarettes per day	1.252763	1.061297
aortic_aneurism	343424	0	0 cigarettes per day	1 to 19 cigarettes per day	1.280934	1.031817
aortic_aneurism	343424	0	0 cigarettes per day	20 to 20 cigarettes per day	1.526056	0.807509
aortic_aneurism	343424	0	0 cigarettes per day	21 to 31.5 cigarettes per day	1.568616	0.773863
aortic_aneurism	343424	0	0 cigarettes per day	20 to 20 cigarettes per day	1.609438	0.742908
aortic_aneurism	343424	0	0 cigarettes per day	21 to 31.5 cigarettes per day	1.84055	0.58961
aortic_aneurism	347380	0	0 cigarettes per day	1 to 7 cigarettes per day	0.530628	2.185024
aortic_aneurism	347380	0	0 cigarettes per day	8 to 15 cigarettes per day	0.993252	1.375756
aortic_aneurism	347380	0	0 cigarettes per day	16 to 24 cigarettes per day	1.098612	1.23818
aortic_aneurism	350128	0	0 cigarettes per day	21 to 31.5 cigarettes per day	1.94591	0.55758
aortic_aneurism	350128	0	0 cigarettes per day	6 to 10 cigarettes per day	1.629241	0.55022
aortic_aneurism	350128	0	0 cigarettes per day	1 to 5 cigarettes per day	0.741937	0.655977
aortic_aneurism	350128	0	0 cigarettes per day	11 to 15 cigarettes per day	1.223775	0.562725

aortic_aneurism	350128	0	0 cigarettes per day	16 to 20 cigarettes per day	1.435085	0.5345
aortic_aneurism	350131	0	0 cigarettes per day	0 to 9 cigarettes per day	0.828552	1.62207
aortic_aneurism	350131	0	0 cigarettes per day	10 to 20 cigarettes per day	1.697449	0.680319
aortic_aneurism	350131	0	0 cigarettes per day	21 to 39 cigarettes per day	1.850028	0.584047
aortic_aneurism	350131	0	0 cigarettes per day	40 to 60 cigarettes per day	1.971299	0.517346
aortic_aneurism	355955	0	0 cigarettes per day	1 to 14 cigarettes per day	-0.19845	4.529928
aortic_aneurism	355955	1	0 cigarettes per day	15 to 24 cigarettes per day	-0.19845	4.529928
aortic_aneurism	358841	1	0 cigarettes per day	0 to 9 cigarettes per day	1.822935	0.43355
aortic_aneurism	358841	0	0 cigarettes per day	10 to 19 cigarettes per day	2.280339	0.383175
aortic_aneurism	358841	0	0 cigarettes per day	20 to 30 cigarettes per day	2.618855	0.458514
aortic_aneurism	358843	1	0 cigarettes per day	25 to 37.5 cigarettes per day	2.721295	0.224893
aortic_aneurism	358843	1	0 cigarettes per day	15 to 24 cigarettes per day	2.653242	0.217376
aortic_aneurism	358843	0	0 cigarettes per day	5 to 14 cigarettes per day	1.774952	0.363196
aortic_aneurism	358843	0	0 cigarettes per day	1 to 4 cigarettes per day	0.587787	0.992063
aortic_aneurism	358845	0	0 cigarettes per day	0 to 19 cigarettes per day	0.951658	0.303365
aortic_aneurism	358845	0	0 cigarettes per day	0 to 19 cigarettes per day	1.064711	0.177692
aortic_aneurism	358845	0	0 cigarettes per day	41 to 61.5 cigarettes per day	1.506297	0.576949
aortic_aneurism	358845	0	0 cigarettes per day	20 to 40 cigarettes per day	1.595339	0.154717
aortic_aneurism	358845	0	0 cigarettes per day	41 to 61.5 cigarettes per day	1.663926	0.188428
aortic_aneurism	358845	0	0 cigarettes per day	20 to 40 cigarettes per day	1.856298	0.267459
aortic_aneurism	358857	0	0 cigarettes per day	21 to 31.5 cigarettes per day	1.18479	0.293328
aortic_aneurism	358857	0	0 cigarettes per day	1 to 20 cigarettes per day	0.792993	0.247022
aortic_aneurism	358857	0	0 cigarettes per day	1 to 20 cigarettes per day	1.085189	0.257688
aortic_aneurism	358857	0	0 cigarettes per day	21 to 31.5 cigarettes per day	1.289233	0.251588
asthma	111344	1	0 cigarettes per day	21 to 31.5 cigarettes per day	0.04879	0.063411
asthma	111344	0	0 cigarettes per day	10 to 19 cigarettes per day	0.058269	0.068589
asthma	111344	0	0 cigarettes per day	0 to 9 cigarettes per day	0.113329	0.081997
asthma	111344	0	0 cigarettes per day	0 to 9 cigarettes per day	0.24686	0.051818
asthma	111344	0	0 cigarettes per day	10 to 19 cigarettes per day	0.322083	0.040668
asthma	111344	0	0 cigarettes per day	21 to 31.5 cigarettes per day	0.398776	0.035954
asthma	111344	0	0 cigarettes per day	0 to 9 cigarettes per day	0.431782	0.09939
asthma	111344	0	0 cigarettes per day	10 to 19 cigarettes per day	0.672944	0.072886
asthma	111344	1	0 cigarettes per day	21 to 31.5 cigarettes per day	0.81978	0.06518
asthma	173863	0	0 cigarettes per day	1 to 14 cigarettes per day	0.405465	1.581633
asthma	173863	0	0 cigarettes per day	25 to 37.5 cigarettes per day	0.405465	1.581633
asthma	173863	0	0 cigarettes per day	15 to 24 cigarettes per day	0.693147	1.186224
asthma	346726	0	0 cigarettes per day	11 to 16.5 cigarettes per day	1.205971	0.335299
asthma	346726	0	0 cigarettes per day	0 to 9 cigarettes per day	0.900161	0.344284
asthma	346728	0	0 cigarettes per day	0.0027 to 0.2712 cigarettes per day	0.09531	0.231911
asthma	346728	0	0 cigarettes per day	0.274 to 0.8192 cigarettes per day	0.587787	0.623583
asthma	346730	0	0 cigarettes per day	16 to 24 cigarettes per day	0.04879	0.206511
asthma	346730	0	0 cigarettes per day	1 to 14 cigarettes per day	0.65752	0.22338

asthma	346737	0	0 cigarettes per day	11 to 16.5 cigarettes per day	0.14842	0.646552
asthma	346737	0	0 cigarettes per day	0 to 9 cigarettes per day	0.239017	0.624699
asthma	346737	0	0 cigarettes per day	0 to 9 cigarettes per day	0.672944	0.715848
asthma	346737	0	0 cigarettes per day	11 to 20 cigarettes per day	0.858662	0.539389
asthma	346737	0	0 cigarettes per day	21 to 30 cigarettes per day	1.043804	0.591046
asthma	346737	0	0 cigarettes per day	31 to 46.5 cigarettes per day	1.686399	0.659486
asthma	346743	0	0 cigarettes per day	26 to 39 cigarettes per day	-0.24846	0.160256
asthma	346743	1	0 cigarettes per day	15 to 24 cigarettes per day	-0.37106	0.140491
asthma	346743	0	0 cigarettes per day	1 to 14 cigarettes per day	-0.22314	0.159439
bladder_cancer	343155	0	0 pack-years	0 to 20 pack-years	0.741937	0.42517
bladder_cancer	343155	0	0 pack-years	21 to 40 pack-years	0.993252	0.245654
bladder_cancer	343155	0	0 pack-years	0 to 20 pack-years	1.360977	0.327054
bladder_cancer	343155	0	0 pack-years	21 to 40 pack-years	1.458615	0.266967
bladder_cancer	343155	0	0 pack-years	41 to 61.5 pack-years	1.504077	0.243764
bladder_cancer	343155	0	0 pack-years	41 to 61.5 pack-years	1.526056	0.160825
bladder_cancer	343198	0	0 pack-years	1 to 19 pack-years	0.405465	0.379611
bladder_cancer	343198	0	0 pack-years	20 to 39 pack-years	0.470004	0.424747
bladder_cancer	343198	0	0 pack-years	40 to 59 pack-years	1.163151	0.49335
bladder_cancer	343198	0	0 pack-years	60 to 90 pack-years	1.987874	0.509558
bladder_cancer	343580	0	0 pack-years	0.05 to 25 pack-years	0.470004	0.243753
bladder_cancer	343580	0	0 pack-years	25.05 to 50 pack-years	0.916291	0.227289
bladder_cancer	343580	0	0 pack-years	50.05 to 75 pack-years	1.193922	0.231306
bladder_cancer	343580	0	0 pack-years	75.05 to 112.5 pack-years	0.916291	0.262658
bladder_cancer	344435	0	0 pack-years	20 to 39 pack-years	0.593327	0.302242
bladder_cancer	344435	0	0 pack-years	1 to 19 pack-years	0.329304	0.274954
bladder_cancer	344435	0	0 pack-years	40 to 60 pack-years	1.585145	0.427412
bladder_cancer	344435	0	0 pack-years	20 to 39 pack-years	0.858662	0.201283
bladder_cancer	344435	0	0 pack-years	1 to 19 pack-years	0.385262	0.202539
bladder_cancer	344435	0	0 pack-years	40 to 60 pack-years	0.875469	0.193384
bladder_cancer	502089	0	0 pack-years	1 to 19 pack-years	1.223775	0.490736
bladder_cancer	502089	1	0 pack-years	20 to 30 pack-years	2.76001	0.503111
bladder_cancer	502130	0	0 pack-years	0 to 10 pack-years	0.928219	0.469006
bladder_cancer	502130	0	0 pack-years	10 to 19 pack-years	1.118415	0.330134
bladder_cancer	502130	1	0 pack-years	20 to 29 pack-years	1.927164	0.249833
bladder_cancer	502130	1	0 pack-years	30 to 45 pack-years	2.00148	0.222311
bladder_cancer	502393	0	0 pack-years	16 to 24 pack-years	-0.22314	1.035827
bladder_cancer	502393	0	0 pack-years	1 to 15 pack-years	-1.20397	1.273936
bladder_cancer	502393	0	0 pack-years	16 to 24 pack-years	0.262364	0.36406
bladder_cancer	502393	0	0 pack-years	1 to 15 pack-years	-0.91629	0.633905
bladder_cancer	502401	0	0 pack-years	0.05 to 19.95 pack-years	-0.13926	0.330439
bladder_cancer	502401	0	0 pack-years	20 to 39.95 pack-years	0.444686	0.326596
bladder_cancer	502401	0	0 pack-years	40 to 60 pack-years	0.698135	0.307368
bladder_cancer	502403	0	0 pack-years	1 to 24 pack-years	0.450693	0.142378

bladder_cancer	502403	0	0 pack-years	25 to 45 pack-years	0.775096	0.124845
bladder_cancer	502403	0	0 pack-years	46 to 69 pack-years	0.796967	0.126581
bladder_cancer	502413	0	0 pack-years	21.52 to 40.51 pack-years	1.420696	0.662358
bladder_cancer	502413	0	0 pack-years	40.52 to 60.78 pack-years	2.424803	0.618586
bladder_cancer	502413	0	0 pack-years	0.06 to 21.51 pack-years	0.924259	0.759246
bladder_cancer	502413	0	0 pack-years	0.06 to 21.51 pack-years	0.770108	0.410044
bladder_cancer	502413	0	0 pack-years	21.52 to 40.51 pack-years	0.985817	0.372693
bladder_cancer	502413	0	0 pack-years	40.52 to 60.78 pack-years	1.947338	0.340188
bladder_cancer	502415	0	0 pack-years	1 to 19 pack-years	0.377957	0.1952
bladder_cancer	502415	0	0 pack-years	20 to 30 pack-years	1.311571	0.174185
bladder_cancer	502417	0	0 pack-years	1 to 19 pack-years	0.482426	0.337183
bladder_cancer	502417	0	0 pack-years	20 to 39 pack-years	0.431782	0.34702
bladder_cancer	502417	0	0 pack-years	40 to 60 pack-years	0.647103	0.39979
bladder_cancer	502423	0	0 pack-years	1 to 26 pack-years	0.774958	0.329439
bladder_cancer	502423	0	0 pack-years	27 to 40.5 pack-years	1.590174	0.312474
bladder_cancer	502425	0	0 pack-years	1 to 19 pack-years	0.223144	0.290816
bladder_cancer	502425	0	0 pack-years	20 to 39 pack-years	1.071584	0.214433
bladder_cancer	502425	0	0 pack-years	40 to 60 pack-years	1.420696	0.1925
bladder_cancer	502425	0	0 pack-years	1 to 19 pack-years	1.105257	0.238826
bladder_cancer	502425	1	0 pack-years	20 to 39 pack-years	1.530395	0.205306
bladder_cancer	502425	0	0 pack-years	40 to 60 pack-years	1.693779	0.242939
bladder_cancer	502429	0	0 pack-years	35 to 52.5 pack-years	1.193922	0.334164
bladder_cancer	502429	0	0 pack-years	1 to 34 pack-years	0.587787	0.339065
bladder_cancer	502431	0	0 pack-years	1 to 24 pack-years	0.369078	0.093233
bladder_cancer	502431	1	0 pack-years	25 to 49 pack-years	0.53462	0.091281
bladder_cancer	502431	1	0 pack-years	50 to 75 pack-years	0.569113	0.094811
bladder_cancer	502435	0	0 pack-years	1 to 26 pack-years	0.495793	0.234175
bladder_cancer	502435	0	0 pack-years	27 to 40.5 pack-years	0.886738	0.217395
bladder_cancer	502437	0	0 pack-years	23.25 to 36.75 pack-years	0.587787	0.156404
bladder_cancer	502437	0	0 pack-years	1 to 10 pack-years	0.587787	0.166818
bladder_cancer	502437	0	0 pack-years	11 to 23.24 pack-years	0.530628	0.145547
bladder_cancer	502437	0	0 pack-years	36.76 to 55.14 pack-years	0.587787	0.156404
bladder_cancer	502437	0	0 pack-years	1 to 10 pack-years	0.470004	0.188163
bladder_cancer	502444	0	0 pack-years	0.1 to 10 pack-years	-0.09431	0.398124
bladder_cancer	502444	0	0 pack-years	11 to 20 pack-years	0.693147	0.381878
bladder_cancer	502444	0	0 pack-years	40 to 50 pack-years	1.360977	0.167546
bladder_cancer	502444	0	0 pack-years	31 to 40 pack-years	0.916291	0.374066
bladder_cancer	502444	0	0 pack-years	40 to 50 pack-years	1.193922	0.275743
bladder_cancer	502444	0	0 pack-years	21 to 30 pack-years	0.530628	0.437449
bladder_cancer	502444	0	0 pack-years	31 to 40 pack-years	0.832909	0.223334
bladder_cancer	502444	0	0 pack-years	11 to 20 pack-years	0.530628	0.238344
bladder_cancer	502444	0	0 pack-years	21 to 30 pack-years	0.587787	0.247296
bladder_cancer	502444	0	0 pack-years	0.1 to 10 pack-years	0.09531	0.285686

bladder_cancer	502444	0	0 pack-years	40 to 50 pack-years	1.280934	0.132128
bladder_cancer	502444	0	0 pack-years	31 to 40 pack-years	0.832909	0.201137
bladder_cancer	502444	0	0 pack-years	21 to 30 pack-years	0.530628	0.197242
bladder_cancer	502444	0	0 pack-years	11 to 20 pack-years	0.530628	0.197242
bladder_cancer	502444	0	0 pack-years	0.1 to 10 pack-years	0	0.233748
bladder_cancer	502446	1	0 pack-years	22.5 to 33.75 pack-years	0.265053	0.072072
bladder_cancer	502446	0	0 pack-years	1 to 22.5 pack-years	0.190868	0.079266
bladder_cancer	502446	0	0 pack-years	22.5 to 33.75 pack-years	0.742842	0.094918
bladder_cancer	502446	0	0 pack-years	1 to 22.5 pack-years	0.332608	0.112269
bladder_cancer	502446	0	0 pack-years	22.6 to 33.9 pack-years	0.742842	0.094918
bladder_cancer	502446	0	0 pack-years	1 to 22.5 pack-years	0.190868	0.079266
bladder_cancer	502446	0	0 pack-years	1 to 22.5 pack-years	0.332608	0.112284
bladder_cancer	502446	1	0 pack-years	22.6 to 33.9 pack-years	0.265053	0.033218
bladder_cancer	502450	0	0 pack-years	60 to 90 pack-years	1.435085	0.142759
bladder_cancer	502450	0	0 pack-years	50 to 59 pack-years	1.609438	0.191404
bladder_cancer	502450	0	0 pack-years	1 to 19 pack-years	0.530628	0.12234
bladder_cancer	502450	0	0 pack-years	20 to 39 pack-years	1.163151	0.126198
bladder_cancer	502450	0	0 pack-years	40 to 49 pack-years	1.589235	0.165649
bladder_cancer	502452	0	0 pack-years	1 to 29 pack-years	0.405465	0.117228
bladder_cancer	502452	0	0 pack-years	30 to 59.9 pack-years	0.955511	0.107452
bladder_cancer	502452	0	0 pack-years	60 to 89.9 pack-years	0.993252	0.130313
bladder_cancer	502452	0	0 pack-years	90 to 135 pack-years	1.098612	0.134711
bladder_cancer	502452	0	0 pack-years	1 to 29 pack-years	0.530628	0.154626
bladder_cancer	502452	0	0 pack-years	30 to 59.9 pack-years	1.064711	0.132621
bladder_cancer	502452	0	0 pack-years	60 to 89.9 pack-years	1.252763	0.217727
bladder_cancer	502452	0	0 pack-years	90 to 135 pack-years	0.993252	0.301982
bladder_cancer	502456	0	0 pack-years	50 to 75 pack-years	0.806476	0.30131
bladder_cancer	502456	0	0 pack-years	25 to 37.5 pack-years	1.66203	0.743037
bladder_cancer	502456	0	0 pack-years	40 to 49 pack-years	0.662688	0.309054
bladder_cancer	502456	1	0 pack-years	1 to 24 pack-years	2.116256	0.43364
bladder_cancer	502456	0	0 pack-years	20 to 29 pack-years	0.277632	0.323714
bladder_cancer	502456	0	0 pack-years	1 to 19 pack-years	-0.16252	0.427895
bladder_cancer	502456	0	0 pack-years	30 to 39 pack-years	0.207014	0.310164
bladder_cancer	502458	0	0 pack-years	75.05 to 112.5 pack-years	1.223775	0.259163
bladder_cancer	502458	0	0 pack-years	50.05 to 75 pack-years	1.435085	0.241479
bladder_cancer	502458	0	0 pack-years	0.05 to 25 pack-years	0.530628	0.262658
bladder_cancer	502458	0	0 pack-years	25.05 to 50 pack-years	1.131402	0.223334
bladder_cancer	502462	0	0 pack-years	1 to 30 pack-years	0.751416	0.295513
bladder_cancer	502462	0	0 pack-years	31 to 46.5 pack-years	0.832909	0.290515
bladder_cancer	502464	0	0 pack-years	0 to 25 pack-years	1.029619	0.18345
bladder_cancer	502464	1	0 pack-years	25 to 51 pack-years	1.824549	0.170959
bladder_cancer	502464	1	0 pack-years	52 to 78 pack-years	1.960095	0.176823
bladder_cancer	502464	0	0 pack-years	1 to 14 pack-years	0.587787	0.673229

bladder_cancer	502464	0	0 pack-years	15 to 22.5 pack-years	1.481605	0.630613
bladder_cancer	502466	0	0 pack-years	1 to 19 pack-years	0.108316	0.347729
bladder_cancer	502466	0	0 pack-years	20 to 30 pack-years	0.834647	0.106224
bladder_cancer	502468	0	0 pack-years	1 to 10 pack-years	0.470004	0.417757
bladder_cancer	502468	0	0 pack-years	30 to 45 pack-years	0.832909	0.286717
bladder_cancer	502468	1	0 pack-years	21 to 30 pack-years	1.547563	0.204112
bladder_cancer	502468	1	0 pack-years	11 to 20 pack-years	1.193922	0.136612
bladder_cancer	502468	0	0 pack-years	1 to 10 pack-years	0.530628	0.187237
bladder_cancer	502468	0	0 pack-years	30 to 45 pack-years	0.693147	0.543782
bladder_cancer	502468	1	0 pack-years	21 to 30 pack-years	1.308333	0.103435
bladder_cancer	502468	0	0 pack-years	11 to 20 pack-years	0.641854	0.374066
bladder_cancer	502468	0	0 pack-years	30 to 45 pack-years	1.280934	0.097702
bladder_cancer	502468	1	0 pack-years	11 to 20 pack-years	1.098612	0.110425
bladder_cancer	502468	0	0 pack-years	1 to 10 pack-years	0.405465	0.176823
bladder_cancer	502468	0	0 pack-years	21 to 30 pack-years	0.993252	0.457081
bladder_cancer	502476	0	0 pack-years	1 to 31 pack-years	0.270103	0.157661
bladder_cancer	502476	0	0 pack-years	1 to 31 pack-years	0.319181	0.101467
bladder_cancer	502476	0	0 pack-years	1 to 31 pack-years	0.627061	0.093405
bladder_cancer	502476	0	0 pack-years	1 to 31 pack-years	0.902881	0.143099
bladder_cancer	502486	0	0 pack-years	20 to 20 pack-years	0.515813	0.12026
bladder_cancer	502486	0	0 pack-years	40 to 40 pack-years	0.831168	0.134615
bladder_cancer	502486	0	0 pack-years	80 to 80 pack-years	1.24069	0.085398
bladder_cancer	502486	0	0 pack-years	60 to 60 pack-years	0.86878	0.154798
bladder_cancer	502486	0	0 pack-years	40 to 40 pack-years	0.978702	0.064352
bladder_cancer	502486	0	0 pack-years	20 to 20 pack-years	0.555608	0.057154
bladder_cancer	502486	0	0 pack-years	60 to 60 pack-years	1.200266	0.069304
bladder_cancer	502486	0	0 pack-years	80 to 80 pack-years	0.980204	0.227258
breast_cancer	309776	1	0 pack-years	0 to 19 pack-years	0.741937	0.42517
breast_cancer	309776	1	0 pack-years	20 to 30 pack-years	1.064711	0.404645
breast_cancer	310321	0	0 pack-years	1 to 4 pack-years	0.076961	0.167706
breast_cancer	310321	0	0 pack-years	10 to 14 pack-years	0.076961	0.108655
breast_cancer	310321	0	0 pack-years	15 to 19 pack-years	0.198851	0.100368
breast_cancer	310321	0	0 pack-years	5 to 9 pack-years	0.262364	0.123626
breast_cancer	310321	0	0 pack-years	20 to 30 pack-years	0.29267	0.083765
breast_cancer	310343	0	0 pack-years	1 to 5 pack-years	0.067659	0.033426
breast_cancer	310343	0	0 pack-years	6 to 10 pack-years	0.10436	0.034371
breast_cancer	310343	0	0 pack-years	11 to 15 pack-years	0.165514	0.03899
breast_cancer	310343	1	0 pack-years	16 to 24 pack-years	0.29267	0.037862
breast_cancer	310352	0	0 pack-years	10 to 19 pack-years	0.19062	0.059032
breast_cancer	310352	0	0 pack-years	50 to 75 pack-years	0.165514	0.075666
breast_cancer	310352	0	0 pack-years	40 to 49 pack-years	0.14842	0.123153
breast_cancer	310352	0	0 pack-years	0 to 9 pack-years	0.039221	0.046605
breast_cancer	310352	0	0 pack-years	30 to 39 pack-years	0.00995	0.08335

breast_cancer	310352	0	0 pack-years	20 to 29 pack-years	0.122218	0.072241
breast_cancer	310353	0	0 pack-years	1 to 10 pack-years	0	0.033044
breast_cancer	310353	0	0 pack-years	11 to 20 pack-years	0.076961	0.035324
breast_cancer	310353	0	0 pack-years	21 to 30 pack-years	0.067659	0.035654
breast_cancer	310353	0	0 pack-years	31 to 40 pack-years	0.14842	0.041697
breast_cancer	310353	0	0 pack-years	41 to 50 pack-years	0.04879	0.058008
breast_cancer	310353	0	0 pack-years	51 to 76.5 pack-years	0.173953	0.05549
breast_cancer	310366	0	0 pack-years	21 to 30 pack-years	0.336472	0.109329
breast_cancer	310366	0	0 pack-years	11 to 20 pack-years	0	0.102041
breast_cancer	310366	0	0 pack-years	1 to 10 pack-years	-0.10536	0.141723
breast_cancer	310366	0	0 pack-years	31 to 46.5 pack-years	-0.10536	0.141723
breast_cancer	310414	0	0 pack-years	15 to 19 pack-years	0.24686	0.151467
breast_cancer	310414	0	0 pack-years	20 to 30 pack-years	0.392042	0.14134
breast_cancer	310428	0	0 pack-years	0 to 19 pack-years	0.262364	0.470958
breast_cancer	310428	0	0 pack-years	20 to 30 pack-years	0.336472	0.182216
breast_cancer	310438	0	0 pack-years	31 to 46.5 pack-years	0.223144	0.083673
breast_cancer	310438	0	0 pack-years	0 to 10 pack-years	0.019803	0.062525
breast_cancer	310438	0	0 pack-years	21 to 29 pack-years	0.113329	0.109329
breast_cancer	310438	0	0 pack-years	11 to 19 pack-years	0.215111	0.084348
breast_cancer	310463	0	0 pack-years	10.1 to 19 pack-years	-0.15082	0.16018
breast_cancer	310463	1	0 pack-years	21 to 31.5 pack-years	-0.17435	0.151846
breast_cancer	310463	0	0 pack-years	3.8 to 9 pack-years	-0.17435	0.1974
breast_cancer	310463	1	0 pack-years	0 to 3.7 pack-years	-0.34249	0.247916
breast_cancer	310487	0	0 pack-years	20 to 29 pack-years	0.09531	0.069573
breast_cancer	310487	0	0 pack-years	40 to 60 pack-years	0.09531	0.115955
breast_cancer	310487	0	0 pack-years	0 to 9 pack-years	0.09531	0.069573
breast_cancer	310487	0	0 pack-years	10 to 19 pack-years	0.09531	0.069573
breast_cancer	310487	0	0 pack-years	30 to 39 pack-years	0.262364	0.098116
breast_cancer	328339	1	0 pack-years	20 to 39 pack-years	0.542324	0.97888
breast_cancer	328339	0	0 pack-years	0 to 19 pack-years	0.524729	0.532846
breast_cancer	358689	0	0 pack-years	20 to 30 pack-years	0.00995	0.442008
breast_cancer	358689	1	0 pack-years	0 to 19 pack-years	-0.17435	0.728863
breast_cancer	359074	0	0 pack-years	5 to 9 pack-years	0.04879	0.10447
breast_cancer	359074	0	0 pack-years	1 to 4 pack-years	0.09531	0.081169
breast_cancer	359074	0	0 pack-years	10 to 19 pack-years	0.239017	0.092399
breast_cancer	359076	0	0 pack-years	40 to 60 pack-years	-0.16252	0.222089
breast_cancer	359076	0	0 pack-years	1 to 8 pack-years	0.10436	0.098823
breast_cancer	359076	0	0 pack-years	10 to 18 pack-years	0.165514	0.142684
breast_cancer	359076	0	0 pack-years	20 to 28 pack-years	0.173953	0.158635
breast_cancer	359076	0	0 pack-years	30 to 38 pack-years	0.385262	0.206511
breast_cancer	359078	0	0 pack-years	21 to 31.5 pack-years	0.173953	0.128623
breast_cancer	359078	0	0 pack-years	20 to 28 pack-years	0.09531	0.10436
breast_cancer	359078	0	0 pack-years	31 to 46.5 pack-years	0.067659	0.288957

breast_cancer	359078	0	0 pack-years	0 to 9 pack-years	-0.07257	0.065833
breast_cancer	359078	0	0 pack-years	0 to 19 pack-years	-0.19845	0.171105
breast_cancer	359078	0	0 pack-years	10 to 18 pack-years	0.058269	0.089045
breast_cancer	359082	0	0 pack-years	0 to 14 pack-years	0.00995	0.202061
breast_cancer	359082	0	0 pack-years	14 to 20 pack-years	0.24686	0.292969
breast_cancer	359082	0	0 pack-years	21 to 31.5 pack-years	0.29267	0.19799
breast_cancer	359086	0	0 pack-years	5 to 8.9 pack-years	-0.10536	0.255102
breast_cancer	359086	0	0 pack-years	10 to 13.9 pack-years	0.182322	0.297619
breast_cancer	359086	0	0 pack-years	2.5 to 3.9 pack-years	0.262364	0.294349
breast_cancer	359086	1	0 pack-years	0 to 1.5 pack-years	0.405465	0.170068
breast_cancer	359086	1	0 pack-years	15 to 22.5 pack-years	0.470004	0.191327
breast_cancer	359094	0	0 pack-years	31 to 46.5 pack-years	0.239017	0.345493
breast_cancer	359094	0	0 pack-years	10 to 20 pack-years	0.029559	0.356648
breast_cancer	359094	0	0 pack-years	20 to 30 pack-years	-0.23572	0.461767
breast_cancer	359094	0	0 pack-years	0 to 10 pack-years	-0.03046	0.32085
breast_cancer	448179	0	0 pack-years	0 to 5 pack-years	0.139762	0.059908
breast_cancer	448179	0	0 pack-years	6 to 10 pack-years	0.165514	0.064655
breast_cancer	448179	0	0 pack-years	11 to 15 pack-years	0.270027	0.068303
breast_cancer	448179	0	0 pack-years	15 to 22.5 pack-years	0.254642	0.064856
breast_cancer	502130	0	0 pack-years	0 to 10 pack-years	-0.03046	0.058229
breast_cancer	502130	1	0 pack-years	10 to 19 pack-years	-0.12783	0.089259
breast_cancer	502130	0	0 pack-years	20 to 29 pack-years	0.113329	0.085835
breast_cancer	502130	0	0 pack-years	30 to 45 pack-years	-0.01005	0.079321
breast_cancer	502184	0	0 pack-years	1 to 9 pack-years	0.157004	0.181448
breast_cancer	502184	0	0 pack-years	10 to 19 pack-years	0.04879	0.245258
breast_cancer	502184	0	0 pack-years	20 to 30 pack-years	0.451076	0.221451
breast_cancer	502184	0	0 pack-years	1 to 9 pack-years	0.058269	0.161461
breast_cancer	502184	0	0 pack-years	10 to 19 pack-years	-0.04082	0.208635
breast_cancer	502184	0	0 pack-years	20 to 30 pack-years	0.122218	0.15602
breast_cancer	502439	0	0 pack-years	10 to 14.9 pack-years	0.207014	0.083016
breast_cancer	502439	0	0 pack-years	15 to 19.9 pack-years	-0.01005	0.099253
breast_cancer	502439	0	0 pack-years	2.5 to 4.9 pack-years	0.215111	0.104276
breast_cancer	502439	0	0 pack-years	5 to 9.9 pack-years	0.131028	0.084327
breast_cancer	502439	0	0 pack-years	20 to 30 pack-years	0.231112	0.076907
cataracts	261496	0	0 cigarettes per day	1 to 14 cigarettes per day	-0.06188	0.881323
cataracts	261496	0	0 cigarettes per day	1 to 14 cigarettes per day	0.364643	0.575308
cataracts	261496	0	0 cigarettes per day	15 to 22.5 cigarettes per day	0.470004	0.517777
cataracts	261496	0	0 cigarettes per day	15 to 22.5 cigarettes per day	0.482426	0.511385
cataracts	261527	0	0 cigarettes per day	0 to 19 cigarettes per day	-0.0202	0.471158
cataracts	261527	0	0 cigarettes per day	20 to 30 cigarettes per day	0.350657	0.434751
cataracts	261527	0	0 cigarettes per day	0 to 19 cigarettes per day	0.500775	0.38961
cataracts	263023	0	0 cigarettes per day	1 to 5 cigarettes per day	-0.05129	0.126208
cataracts	263023	0	0 cigarettes per day	6 to 10 cigarettes per day	0.076961	0.09212

cataracts	263023	0	0 cigarettes per day	11 to 15 cigarettes per day	0.19062	0.109631
cataracts	263023	0	0 cigarettes per day	16 to 24 cigarettes per day	0.285179	0.161117
cataracts	350491	1	0 cigarettes per day	1 to 14 cigarettes per day	0.932164	0.227088
cataracts	350491	1	0 cigarettes per day	15 to 24 cigarettes per day	0.978326	0.249232
cataracts	350491	0	0 cigarettes per day	25 to 37.5 cigarettes per day	1.05779	0.364788
cataracts	350493	0	0 cigarettes per day	35 to 52.5 cigarettes per day	0.457425	0.264789
cataracts	350493	0	0 cigarettes per day	15 to 24 cigarettes per day	0.285179	0.153445
cataracts	350493	0	0 cigarettes per day	25 to 34 cigarettes per day	0.336472	0.213192
cataracts	350493	0	0 cigarettes per day	1 to 14 cigarettes per day	-0.34249	0.233544
cataracts	350493	0	0 cigarettes per day	1 to 14 cigarettes per day	0.14842	0.105559
cataracts	359116	0	0 cigarettes per day	0 to 14 cigarettes per day	0.113329	0.011388
cataracts	359116	0	0 cigarettes per day	15 to 22.5 cigarettes per day	0.231112	0.014172
cataracts	359118	0	0 cigarettes per day	1 to 5 cigarettes per day	0.10436	0.054875
cataracts	359118	0	0 cigarettes per day	6 to 10 cigarettes per day	0.131028	0.040363
cataracts	359118	0	0 cigarettes per day	11 to 15 cigarettes per day	0.165514	0.043342
cataracts	359118	0	0 cigarettes per day	15 to 22.5 cigarettes per day	0.300105	0.039616
cataracts	359119	0	0 cigarettes per day	0 to 19 cigarettes per day	-0.06188	0.374512
cataracts	359119	0	0 cigarettes per day	0 to 19 cigarettes per day	-0.04082	0.369366
cataracts	359119	0	0 cigarettes per day	20 to 30 cigarettes per day	0.71784	0.207815
cataracts	359121	0	0 cigarettes per day	0 to 9 cigarettes per day	0.00995	0.164175
cataracts	359121	0	0 cigarettes per day	20 to 29 cigarettes per day	0.113329	0.16855
cataracts	359121	0	0 cigarettes per day	31 to 46.5 cigarettes per day	0.378436	0.262091
cataracts	501958	0	0 cigarettes per day	10 to 15 cigarettes per day	-0.27813	0.150845
cataracts	501958	1	0 cigarettes per day	16 to 20 cigarettes per day	-0.32962	0.183545
cataracts	501958	0	0 cigarettes per day	1 to 9 cigarettes per day	-0.17162	0.21322
cataracts	501958	1	0 cigarettes per day	21 to 31.5 cigarettes per day	-0.21791	0.180479
cervical_cancer	328339	1	0 pack-years	0 to 19 pack-years	-0.07257	1.859776
cervical_cancer	328339	0	0 pack-years	20 to 39 pack-years	1.427916	1.016738
cervical_cancer	347864	0	0 pack-years	0 to 1 pack-years	0.254642	0.312451
cervical_cancer	347864	0	0 pack-years	12 to 18 pack-years	0.385262	0.315841
cervical_cancer	347864	0	0 pack-years	8 to 11 pack-years	0.41871	0.313843
cervical_cancer	347864	0	0 pack-years	4 to 5 pack-years	0.746688	0.305881
cervical_cancer	347864	1	0 pack-years	2 to 3 pack-years	0.797507	0.294172
cervical_cancer	347864	0	0 pack-years	6 to 7 pack-years	0.951658	0.321094
cervical_cancer	358583	0	0 pack-years	0 to 9 pack-years	0.405465	0.204082
cervical_cancer	502130	0	0 pack-years	0 to 10 pack-years	0.536493	0.740159
cervical_cancer	502130	0	0 pack-years	10 to 19 pack-years	1.015231	0.460475
cervical_cancer	502130	0	0 pack-years	20 to 29 pack-years	0.993252	0.492843
cervical_cancer	502130	0	0 pack-years	30 to 45 pack-years	0.908259	0.457922
colon_and_rectum_cancer	164581	0	0 pack-years	0 to 10 pack-years	-0.52763	0.609651
colon_and_rectum_cancer	164581	0	0 pack-years	11 to 16.5 pack-years	-0.26136	0.54996
colon_and_rectum_cancer	164581	0	0 pack-years	11 to 16.5 pack-years	-0.06188	1.071971
colon_and_rectum_cancer	164581	0	0 pack-years	0 to 20 pack-years	0.285179	0.489105

colon_and_rectum_cancer	164581	0	0 pack-years	0 to 20 pack-years	0.307485	0.288866
colon_and_rectum_cancer	164581	0	0 pack-years	21 to 31.5 pack-years	0.314811	0.281171
colon_and_rectum_cancer	164581	0	0 pack-years	0 to 10 pack-years	0.565314	0.658047
colon_and_rectum_cancer	164581	1	0 pack-years	21 to 31.5 pack-years	0.891998	0.437019
colon_and_rectum_cancer	309619	0	0 pack-years	30 to 39 pack-years	0.336472	0.173105
colon_and_rectum_cancer	328339	1	0 pack-years	40 to 59 pack-years	1.272566	1.837878
colon_and_rectum_cancer	328339	0	0 pack-years	20 to 39 pack-years	0.71784	0.990543
colon_and_rectum_cancer	328339	0	0 pack-years	40 to 59 pack-years	0.593327	0.267787
colon_and_rectum_cancer	328339	0	0 pack-years	0 to 19 pack-years	0.24686	0.406569
colon_and_rectum_cancer	328339	0	0 pack-years	20 to 39 pack-years	0.131028	0.2372
colon_and_rectum_cancer	328339	0	0 pack-years	40 to 59 pack-years	0.113329	0.264213
colon_and_rectum_cancer	328339	0	0 pack-years	20 to 39 pack-years	0.300105	0.725624
colon_and_rectum_cancer	328339	0	0 pack-years	0 to 19 pack-years	0.076961	0.375567
colon_and_rectum_cancer	328339	0	0 pack-years	60 to 90 pack-years	0.029559	0.517634
colon_and_rectum_cancer	328339	0	0 pack-years	0 to 19 pack-years	-0.05129	0.988185
colon_and_rectum_cancer	328339	0	0 pack-years	60 to 90 pack-years	-0.11653	0.464343
colon_and_rectum_cancer	328339	0	0 pack-years	0 to 19 pack-years	-0.4943	0.719304
colon_and_rectum_cancer	328339	0	0 pack-years	20 to 39 pack-years	0.113329	0.273324
colon_and_rectum_cancer	343580	0	0 pack-years	75.05 to 112.5 pack-years	0.09531	0.294051
colon_and_rectum_cancer	343580	0	0 pack-years	50.05 to 75 pack-years	-0.10536	0.262658
colon_and_rectum_cancer	343580	0	0 pack-years	25.05 to 50 pack-years	0.09531	0.240934
colon_and_rectum_cancer	343580	0	0 pack-years	0.05 to 25 pack-years	0.09531	0.240934
colon_and_rectum_cancer	343580	0	0 pack-years	50.05 to 75 pack-years	-0.10536	0.197242
colon_and_rectum_cancer	343580	0	0 pack-years	25.05 to 50 pack-years	0.09531	0.176823
colon_and_rectum_cancer	343580	0	0 pack-years	0.05 to 25 pack-years	0.182322	0.20687
colon_and_rectum_cancer	343580	0	0 pack-years	75.05 to 112.5 pack-years	-0.10536	0.233748
colon_and_rectum_cancer	347927	0	0 pack-years	40 to 60 pack-years	0.482426	0.226757
colon_and_rectum_cancer	347927	0	0 pack-years	20 to 39 pack-years	0.076961	0.238568
colon_and_rectum_cancer	347927	0	0 pack-years	1 to 19 pack-years	0.139762	0.106477
colon_and_rectum_cancer	347927	0	0 pack-years	20 to 39 pack-years	0.14842	0.116555
colon_and_rectum_cancer	347927	0	0 pack-years	40 to 60 pack-years	0.19062	0.130713
colon_and_rectum_cancer	347927	0	0 pack-years	1 to 19 pack-years	0.262364	0.202119
colon_and_rectum_cancer	347932	0	0 pack-years	10 to 19 pack-years	0.405465	0.136054
colon_and_rectum_cancer	347932	0	0 pack-years	0 to 9 pack-years	0	0.127551
colon_and_rectum_cancer	347932	0	0 pack-years	20 to 30 pack-years	0.262364	0.156986
colon_and_rectum_cancer	347935	0	0 pack-years	0 to 19 pack-years	-0.44629	0.613839
colon_and_rectum_cancer	347935	0	0 pack-years	40 to 60 pack-years	0.076961	0.576342
colon_and_rectum_cancer	347935	0	0 pack-years	20 to 39 pack-years	0.14842	0.580577
colon_and_rectum_cancer	347935	0	0 pack-years	0 to 19 pack-years	0.845868	0.786108
colon_and_rectum_cancer	347935	0	0 pack-years	40 to 60 pack-years	0.985817	0.615862
colon_and_rectum_cancer	347935	1	0 pack-years	20 to 39 pack-years	1.435085	0.607993
colon_and_rectum_cancer	358658	0	0 pack-years	0.25 to 40 pack-years	0.966984	0.879763
colon_and_rectum_cancer	358658	0	0 pack-years	61 to 91.5 pack-years	0.262364	0.792779

colon_and_rectum_cancer	358658	0	0 pack-years	41 to 60 pack-years	-0.05129	0.687433
colon_and_rectum_cancer	358689	0	0 pack-years	20 to 30 pack-years	-0.34249	0.402414
colon_and_rectum_cancer	358689	0	0 pack-years	0 to 19 pack-years	0.239017	0.283224
colon_and_rectum_cancer	502130	1	0 pack-years	0 to 10 pack-years	0.444686	0.169897
colon_and_rectum_cancer	502130	0	0 pack-years	10 to 19 pack-years	0.262364	0.139424
colon_and_rectum_cancer	502130	0	0 pack-years	20 to 29 pack-years	0.285179	0.144213
colon_and_rectum_cancer	502130	0	0 pack-years	30 to 45 pack-years	0.122218	0.136756
colon_and_rectum_cancer	502135	0	0 pack-years	30 to 45 pack-years	0.039221	0.09245
colon_and_rectum_cancer	502135	0	0 pack-years	1 to 30 pack-years	-0.03046	0.1013
colon_and_rectum_cancer	502139	0	0 pack-years	1 to 19 pack-years	0.076961	0.089019
colon_and_rectum_cancer	502139	0	0 pack-years	20 to 39 pack-years	0.086178	0.099627
colon_and_rectum_cancer	502139	0	0 pack-years	40 to 60 pack-years	0.239017	0.101794
colon_and_rectum_cancer	502184	0	0 pack-years	1 to 9 pack-years	-0.13926	0.303927
colon_and_rectum_cancer	502184	0	0 pack-years	10 to 19 pack-years	0.518794	0.296892
colon_and_rectum_cancer	502184	1	0 pack-years	20 to 30 pack-years	0.71295	0.238487
colon_and_rectum_cancer	502184	0	0 pack-years	1 to 9 pack-years	0.19062	0.326326
colon_and_rectum_cancer	502184	0	0 pack-years	10 to 19 pack-years	0.300105	0.342307
colon_and_rectum_cancer	502184	0	0 pack-years	20 to 30 pack-years	0.215111	0.286177
colon_and_rectum_cancer	502196	1	0 pack-years	0 to 20 pack-years	0.797507	0.275602
colon_and_rectum_cancer	502196	1	0 pack-years	20 to 30 pack-years	1.178655	0.521313
colon_and_rectum_cancer	502236	1	0 pack-years	1 to 19 pack-years	-0.44629	0.148762
colon_and_rectum_cancer	502236	0	0 pack-years	20 to 39 pack-years	0.00995	0.14467
colon_and_rectum_cancer	502236	0	0 pack-years	40 to 60 pack-years	0.553885	0.18302
colon_and_rectum_cancer	502236	0	0 pack-years	1 to 19 pack-years	0.683097	0.328534
colon_and_rectum_cancer	502236	0	0 pack-years	20 to 30 pack-years	0.996949	0.61681
colon_and_rectum_cancer	502484	0	0 pack-years	1 to 10 pack-years	-0.21072	0.423364
colon_and_rectum_cancer	502484	0	0 pack-years	11 to 16.5 pack-years	0.173953	0.292644
copd	115406	0	0 pack-years	1 to 10 pack-years	0.182322	0.170068
copd	115406	0	0 pack-years	11 to 20 pack-years	0.955511	0.155024
copd	115406	0	0 pack-years	31 to 40 pack-years	1.098612	0.157313
copd	115406	0	0 pack-years	21 to 30 pack-years	1.223775	0.144058
copd	115406	0	0 pack-years	41 to 50 pack-years	1.360977	0.178571
copd	115406	0	0 pack-years	51 to 76.5 pack-years	1.740466	0.149481
copd	315813	0	0 pack-years	1 to 9 pack-years	0.587787	2.253401
copd	315813	0	0 pack-years	10 to 19 pack-years	2.128232	0.65294
copd	315813	0	0 pack-years	21 to 31.5 pack-years	2.701361	0.636899
copd	343461	0	0 pack-years	28 to 42 pack-years	2.391511	0.929811
copd	343461	0	0 pack-years	14 to 27 pack-years	1.144223	0.489112
copd	343461	0	0 pack-years	14 to 27 pack-years	0.943906	0.271659
copd	343461	0	0 pack-years	28 to 42 pack-years	0.65752	0.325393
copd	343461	0	0 pack-years	1 to 13 pack-years	-0.67334	0.669142
copd	343461	0	0 pack-years	1 to 13 pack-years	0.824175	0.493841
copd	356165	0	0 pack-years	0 to 19 pack-years	1.163151	0.406569

copd	356165	0	0 pack-years	40 to 60 pack-years	1.868721	0.609804
copd	356165	0	0 pack-years	20 to 40 pack-years	1.961502	0.475401
copd	356273	0	0 pack-years	1 to 19 pack-years	0.457425	0.119478
copd	356273	1	0 pack-years	20 to 30 pack-years	0.832909	0.10315
copd	356273	0	0 pack-years	1 to 19 pack-years	0.978326	0.212905
copd	356273	0	0 pack-years	20 to 30 pack-years	1.371181	0.336035
copd	356275	0	0 pack-years	41 to 61.5 pack-years	1.466952	0.405186
copd	356275	0	0 pack-years	0.05 to 20 pack-years	0.692647	0.249104
copd	356275	0	0 pack-years	20 to 40 pack-years	0.736207	0.384364
copd	356342	0	0 pack-years	0 to 20 pack-years	1.321756	0.338095
copd	356342	0	0 pack-years	0 to 19 pack-years	0.593327	0.42423
copd	356344	0	0 pack-years	1 to 10 pack-years	-0.01005	0.405046
copd	356344	0	0 pack-years	11 to 16.5 pack-years	1.036737	0.654056
copd	426275	1	0 pack-years	0 to 19 pack-years	3.068053	0.102041
copd	426275	0	0 pack-years	60 to 79 pack-years	2.785011	0.179516
copd	426275	0	0 pack-years	80 to 100 pack-years	2.595255	0.148492
copd	426275	0	0 pack-years	40 to 59 pack-years	2.501436	0.125878
copd	426275	0	0 pack-years	60 to 79 pack-years	2.424803	0.154416
copd	426275	0	0 pack-years	80 to 100 pack-years	3.058707	0.178452
copd	426275	0	0 pack-years	20 to 39 pack-years	2.19277	0.154883
copd	426275	0	0 pack-years	20 to 39 pack-years	2.174752	0.133349
copd	426275	1	0 pack-years	0 to 19 pack-years	1.944481	0.191235
copd	426275	0	0 pack-years	0 to 19 pack-years	1.776646	0.164888
copd	426275	0	0 pack-years	40 to 59 pack-years	2.251292	0.137487
copd	502093	0	0 pack-years	0 to 20 pack-years	0.058269	0.273307
copd	502093	0	0 pack-years	20 to 39 pack-years	0.518794	0.285518
copd	502093	0	0 pack-years	40 to 60 pack-years	1.105257	0.311913
copd	502095	0	0 pack-years	0.1 to 10 pack-years	0.652325	0.055735
copd	502095	0	0 pack-years	10.1 to 20 pack-years	1.175573	0.042353
copd	502095	0	0 pack-years	20.1 to 30 pack-years	1.719189	0.038406
copd	502095	1	0 pack-years	30.1 to 45.15 pack-years	2.343727	0.031807
copd	502107	0	0 pack-years	1 to 10 pack-years	0.788457	0.112919
copd	502107	1	0 pack-years	11 to 16.5 pack-years	1.764731	0.106998
copd	502472	0	0 pack-years	20 to 30 pack-years	0.866512	0.208305
copd	502472	1	0 pack-years	1 to 19 pack-years	-0.23181	0.18533
diabetes	230548	0	0 cigarettes per day	1 to 20 cigarettes per day	0.198851	0.150552
diabetes	230548	0	0 cigarettes per day	21 to 31.5 cigarettes per day	0.239017	0.154668
diabetes	236197	0	0 cigarettes per day	0 to 9 cigarettes per day	0.300105	1.198413
diabetes	236197	0	0 cigarettes per day	10 to 19 cigarettes per day	0.620576	0.869816
diabetes	236197	0	0 cigarettes per day	20 to 30 cigarettes per day	0.774727	0.745556
diabetes	255434	0	0 cigarettes per day	1 to 9 cigarettes per day	0.157004	0.337956
diabetes	255434	0	0 cigarettes per day	10 to 15 cigarettes per day	0.457425	0.161457
diabetes	255463	0	0 cigarettes per day	1 to 19 cigarettes per day	0.067659	0.295632

diabetes	255463	0	0 cigarettes per day	1 to 19 cigarettes per day	0.131028	0.140977
diabetes	255463	0	0 cigarettes per day	20 to 30 cigarettes per day	0.314811	0.107999
diabetes	255463	0	0 cigarettes per day	20 to 30 cigarettes per day	1.07841	0.341004
diabetes	309584	0	0 cigarettes per day	1 to 10 cigarettes per day	-0.17435	0.264213
diabetes	309584	0	0 cigarettes per day	11 to 20 cigarettes per day	0.231112	0.119453
diabetes	309584	0	0 cigarettes per day	21 to 31.5 cigarettes per day	0.431782	0.127551
diabetes	309588	0	0 cigarettes per day	16 to 25 cigarettes per day	1.18479	0.617082
diabetes	309588	0	0 cigarettes per day	1 to 15 cigarettes per day	0.122218	0.893986
diabetes	309588	0	0 cigarettes per day	26 to 39 cigarettes per day	1.166271	0.697756
diabetes	309592	0	0 cigarettes per day	1 to 14 cigarettes per day	-0.3285	0.262188
diabetes	309592	0	0 cigarettes per day	1 to 14 cigarettes per day	0.131028	0.154404
diabetes	309592	0	0 cigarettes per day	15 to 22.5 cigarettes per day	0.270027	0.089578
diabetes	309592	0	0 cigarettes per day	15 to 22.5 cigarettes per day	0.329304	0.157833
diabetes	309592	0	0 cigarettes per day	15 to 22.5 cigarettes per day	0.336472	0.103863
diabetes	309592	0	0 cigarettes per day	1 to 14 cigarettes per day	0.385262	0.118006
diabetes	309610	0	0 cigarettes per day	20 to 30 cigarettes per day	0.438255	0.014812
diabetes	309610	0	0 cigarettes per day	20 to 30 cigarettes per day	0.582216	0.038479
diabetes	309610	0	0 cigarettes per day	10 to 19 cigarettes per day	0.500775	0.103587
diabetes	309610	0	0 cigarettes per day	10 to 19 cigarettes per day	0.457425	0.03875
diabetes	309610	0	0 cigarettes per day	20 to 30 cigarettes per day	0.451076	0.165735
diabetes	309610	1	0 cigarettes per day	1 to 9 cigarettes per day	0.405465	0.040816
diabetes	309610	0	0 cigarettes per day	20 to 30 cigarettes per day	0.285179	0.072886
diabetes	309610	0	0 cigarettes per day	10 to 19 cigarettes per day	0.314811	0.013034
diabetes	309610	0	0 cigarettes per day	1 to 9 cigarettes per day	0.29267	0.036171
diabetes	309610	0	0 cigarettes per day	1 to 9 cigarettes per day	0.357674	0.083845
diabetes	309610	1	0 cigarettes per day	1 to 9 cigarettes per day	0.262364	0.013736
diabetes	309610	0	0 cigarettes per day	10 to 19 cigarettes per day	0.231112	0.048591
diabetes	348079	0	0 cigarettes per day	1 to 14 cigarettes per day	0.314811	0.309102
diabetes	348079	0	0 cigarettes per day	25 to 37.5 cigarettes per day	0.662688	0.234063
diabetes	348079	0	0 cigarettes per day	15 to 24 cigarettes per day	0.8671	0.216515
diabetes	348081	0	0 cigarettes per day	1 to 20 cigarettes per day	0.336472	0.147595
diabetes	348081	0	0 cigarettes per day	31 to 46.5 cigarettes per day	0.548121	0.188746
diabetes	348081	0	0 cigarettes per day	21 to 30 cigarettes per day	0.336472	0.165816
diabetes	348083	0	0 cigarettes per day	0 to 19 cigarettes per day	0.405465	0.204082
diabetes	348083	0	0 cigarettes per day	20 to 30 cigarettes per day	0.530628	0.15006
diabetes	348085	0	0 cigarettes per day	1 to 20 cigarettes per day	0.631272	0.582121
diabetes	348085	0	0 cigarettes per day	21 to 30 cigarettes per day	1.105257	0.573557
diabetes	348085	0	0 cigarettes per day	31 to 46.5 cigarettes per day	1.408545	0.540766
diabetes	348088	0	0 cigarettes per day	20 to 30 cigarettes per day	0.536493	0.18797
diabetes	348088	0	0 cigarettes per day	1 to 19 cigarettes per day	0.582216	0.210922
diabetes	348091	0	0 cigarettes per day	0 to 19 cigarettes per day	0.04879	0.114189
diabetes	348091	0	0 cigarettes per day	20 to 30 cigarettes per day	0.494696	0.197549
diabetes	348100	0	0 cigarettes per day	0 to 19 cigarettes per day	0.378436	0.103089

diabetes	348100	0	0 cigarettes per day	0 to 19 cigarettes per day	0.329304	0.108281
diabetes	348100	0	0 cigarettes per day	20 to 30 cigarettes per day	0.231112	0.044542
diabetes	348100	0	0 cigarettes per day	20 to 30 cigarettes per day	0.322083	0.101671
diabetes	348105	0	0 cigarettes per day	0 to 19 cigarettes per day	0.198851	0.081549
diabetes	348105	0	0 cigarettes per day	0 to 19 cigarettes per day	0.378436	0.0961
diabetes	348105	0	0 cigarettes per day	20 to 30 cigarettes per day	0.451076	0.081243
diabetes	348105	0	0 cigarettes per day	20 to 30 cigarettes per day	0.625938	0.167794
diabetes	348108	0	0 cigarettes per day	1 to 14 cigarettes per day	0.223144	0.35102
diabetes	348108	0	0 cigarettes per day	15 to 19 cigarettes per day	0.29267	0.338867
diabetes	348108	0	0 cigarettes per day	20 to 30 cigarettes per day	0.314811	0.314688
diabetes	348108	0	0 cigarettes per day	1 to 14 cigarettes per day	0.392042	0.208563
diabetes	348108	0	0 cigarettes per day	15 to 19 cigarettes per day	0.708036	0.306625
diabetes	348108	0	0 cigarettes per day	20 to 30 cigarettes per day	0.741937	0.16035
diabetes	348112	0	0 cigarettes per day	1 to 9 cigarettes per day	0.371564	0.628079
diabetes	356075	1	0 cigarettes per day	0 to 19 cigarettes per day	-0.0202	0.026031
diabetes	356075	0	0 cigarettes per day	0 to 19 cigarettes per day	0.04879	0.034014
diabetes	356075	1	0 cigarettes per day	20 to 39 cigarettes per day	0.173953	0.027868
diabetes	356075	0	0 cigarettes per day	20 to 39 cigarettes per day	0.19062	0.031624
diabetes	356075	0	0 cigarettes per day	40 to 60 cigarettes per day	0.371564	0.040464
diabetes	356075	0	0 cigarettes per day	40 to 60 cigarettes per day	0.553885	0.07917
diabetes	356095	0	0 cigarettes per day	1 to 10 cigarettes per day	0.270027	0.093473
diabetes	356095	0	0 cigarettes per day	11 to 20 cigarettes per day	0.307485	0.056273
diabetes	356095	0	0 cigarettes per day	21 to 31.5 cigarettes per day	0.405465	0.093537
diabetes	356097	0	0 cigarettes per day	20 to 29 cigarettes per day	0.518794	0.224733
diabetes	356097	0	0 cigarettes per day	30 to 45 cigarettes per day	0.832909	0.236247
diabetes	356099	1	0 cigarettes per day	1 to 19 cigarettes per day	-0.21072	0.085034
diabetes	356099	1	0 cigarettes per day	20 to 30 cigarettes per day	-0.22314	0.082908
diabetes	356101	0	0 cigarettes per day	0 to 19 cigarettes per day	0.086178	0.231698
diabetes	356101	0	0 cigarettes per day	20 to 30 cigarettes per day	0.536493	0.361022
diabetes	356101	0	0 cigarettes per day	20 to 30 cigarettes per day	0.565314	0.647901
diabetes	356101	0	0 cigarettes per day	0 to 19 cigarettes per day	0.565314	0.239158
diabetes	356103	0	0 cigarettes per day	15 to 24 cigarettes per day	0.24686	0.145488
diabetes	356103	0	0 cigarettes per day	25 to 37.5 cigarettes per day	0.182322	0.055272
diabetes	356103	0	0 cigarettes per day	0 to 14 cigarettes per day	0.173953	0.083605
diabetes	356103	0	0 cigarettes per day	26 to 39 cigarettes per day	0.157004	0.390284
diabetes	356103	0	0 cigarettes per day	26 to 39 cigarettes per day	0.173953	0.05788
diabetes	356103	1	0 cigarettes per day	15 to 24 cigarettes per day	0.019803	0.045018
diabetes	356103	0	0 cigarettes per day	0 to 14 cigarettes per day	0	0.053571
diabetes	356103	1	0 cigarettes per day	15 to 24 cigarettes per day	-0.01005	0.048959
diabetes	356103	0	0 cigarettes per day	0 to 14 cigarettes per day	0.058269	0.045726
diabetes	356105	0	0 cigarettes per day	0 to 19 cigarettes per day	0.239017	0.064278
diabetes	356105	0	0 cigarettes per day	0 to 19 cigarettes per day	0.357674	0.074925
diabetes	356105	0	0 cigarettes per day	20 to 30 cigarettes per day	0.609766	0.091504

diabetes	356105	0	0 cigarettes per day	20 to 30 cigarettes per day	0.636577	0.079635
diabetes	413800	0	0 cigarettes per day	0 to 9 cigarettes per day	0.262364	0.431711
diabetes	413800	0	0 cigarettes per day	10 to 15 cigarettes per day	1.029619	0.610423
diabetes	502053	1	0 cigarettes per day	20 to 40 cigarettes per day	0.277632	0.011598
diabetes	502053	0	0 cigarettes per day	1 to 20 cigarettes per day	0.165514	0.019549
diabetes	502053	0	0 cigarettes per day	40 to 60 cigarettes per day	0.431782	0.01156
diabetes	502177	0	0 cigarettes per day	1 to 19 cigarettes per day	-0.01005	0.213329
diabetes	502177	0	0 cigarettes per day	20 to 30 cigarettes per day	0.512824	0.22942
esophageal_cancer	298512	0	0 pack-years	0 to 19 pack-years	0.727549	0.361086
esophageal_cancer	298512	0	0 pack-years	20 to 29 pack-years	0.996949	0.34547
esophageal_cancer	298512	0	0 pack-years	30 to 39 pack-years	1.088562	0.332406
esophageal_cancer	298512	0	0 pack-years	40 to 60 pack-years	1.570697	0.308138
esophageal_cancer	309841	0	0 pack-years	0 to 9 pack-years	-0.22314	0.414541
esophageal_cancer	309841	0	0 pack-years	0 to 14 pack-years	0	0.280612
esophageal_cancer	309841	0	0 pack-years	15 to 34 pack-years	0.587787	0.212585
esophageal_cancer	309841	0	0 pack-years	10 to 15 pack-years	0.875469	0.28699
esophageal_cancer	309841	0	0 pack-years	35 to 52.5 pack-years	1.335001	0.20811
esophageal_cancer	328266	0	0 pack-years	20 to 40 pack-years	0.741937	0.619534
esophageal_cancer	328339	0	0 pack-years	20 to 39 pack-years	1.48614	0.343984
esophageal_cancer	328339	0	0 pack-years	60 to 90 pack-years	1.477049	0.484577
esophageal_cancer	328339	0	0 pack-years	20 to 39 pack-years	1.22083	1.900849
esophageal_cancer	328339	0	0 pack-years	0 to 19 pack-years	0.698135	0.552087
esophageal_cancer	328339	0	0 pack-years	0 to 19 pack-years	0.506818	1.902508
esophageal_cancer	328339	0	0 pack-years	40 to 59 pack-years	1.115142	0.384744
esophageal_cancer	339730	0	0 pack-years	16.64 to 32.02 pack-years	0.815365	1.365812
esophageal_cancer	339730	0	0 pack-years	32.03 to 48.045 pack-years	1.701105	2.201884
esophageal_cancer	343383	0	0 pack-years	1 to 39 pack-years	1.648659	0.575098
esophageal_cancer	343383	0	0 pack-years	40 to 79 pack-years	2.066863	0.52703
esophageal_cancer	343383	0	0 pack-years	80 to 100 pack-years	2.827314	0.720553
esophageal_cancer	343580	0	0 pack-years	75.05 to 112.5 pack-years	1.029619	0.493071
esophageal_cancer	343580	0	0 pack-years	50.05 to 75 pack-years	1.131402	0.48075
esophageal_cancer	343580	0	0 pack-years	0.05 to 25 pack-years	0.530628	0.525099
esophageal_cancer	343580	0	0 pack-years	25.05 to 50 pack-years	0.832909	0.475311
esophageal_cancer	345310	0	0 pack-years	1 to 19.9 pack-years	1.175573	0.695232
esophageal_cancer	345310	0	0 pack-years	30 to 39.9 pack-years	1.348073	0.536708
esophageal_cancer	345310	0	0 pack-years	40 to 60 pack-years	1.581038	0.47766
esophageal_cancer	345310	0	0 pack-years	20 to 29.9 pack-years	1.587192	0.526376
esophageal_cancer	345324	1	0 pack-years	0 to 50 pack-years	1.983756	0.474763
esophageal_cancer	345324	0	0 pack-years	51 to 76.5 pack-years	2.844909	0.564932
esophageal_cancer	345328	0	0 pack-years	30 to 45 pack-years	1.633154	0.474962
esophageal_cancer	345328	0	0 pack-years	1 to 29 pack-years	1.358409	0.379211
esophageal_cancer	439528	0	0 pack-years	30 to 45 pack-years	0.647103	0.038706
esophageal_cancer	439528	0	0 pack-years	1 to 29 pack-years	0.392042	0.043048

esophageal_cancer	500795	0	0 pack-years	1 to 5 pack-years	0.182322	0.20687
esophageal_cancer	500795	0	0 pack-years	5 to 9 pack-years	0.09531	0.254727
esophageal_cancer	500795	0	0 pack-years	15 to 22.5 pack-years	0.587787	0.247296
esophageal_cancer	502130	0	0 pack-years	10 to 19 pack-years	0.122218	1.036997
esophageal_cancer	502130	0	0 pack-years	20 to 29 pack-years	1.261298	0.644523
esophageal_cancer	502130	0	0 pack-years	30 to 45 pack-years	1.774952	0.474365
esophageal_cancer	502488	1	0.1 pack-years	2 to 2.9 pack-years	2.047693	0.716361
esophageal_cancer	502488	1	0.1 pack-years	3 to 4.5 pack-years	1.7613	0.784888
gallbladder_diseases	350721	0	0 cigarettes per day	15 to 24 cigarettes per day	0.029559	0.071825
gallbladder_diseases	350721	0	0 cigarettes per day	1 to 14 cigarettes per day	0.09531	0.083488
gallbladder_diseases	350721	0	0 cigarettes per day	25 to 34 cigarettes per day	0.270027	0.09542
gallbladder_diseases	350721	0	0 cigarettes per day	35 to 52.5 cigarettes per day	0.41211	0.11657
gallbladder_diseases	350723	0	0 cigarettes per day	1 to 9 cigarettes per day	0.113329	0.031888
gallbladder_diseases	350723	0	0 cigarettes per day	10 to 19 cigarettes per day	0.207014	0.022814
gallbladder_diseases	350723	0	0 cigarettes per day	20 to 30 cigarettes per day	0.254642	0.029663
gallbladder_diseases	350725	0	0 cigarettes per day	20 to 30 cigarettes per day	0.41871	0.203075
gallbladder_diseases	351491	0	0 cigarettes per day	15 to 22.5 cigarettes per day	0.647103	0.126883
gallbladder_diseases	351491	1	0 cigarettes per day	1 to 14 cigarettes per day	0.698135	0.120571
hip_fracture	132098	0	NA	NA	0.307485	0.172848
hip_fracture	132098	0	NA	NA	0.113329	0.258061
hip_fracture	165283	0	NA	NA	-0.24846	0.274394
hip_fracture	261365	0	NA	NA	0.587787	0.225099
hip_fracture	261365	0	NA	NA	0.587787	0.197242
hip_fracture	261375	0	NA	NA	0.307485	0.098838
hip_fracture	261375	0	NA	NA	0.463734	0.216498
hip_fracture	261377	0	NA	NA	0.604316	0.171908
hip_fracture	261377	0	NA	NA	0.802002	0.390152
hip_fracture	261381	0	NA	NA	0.788457	0.353647
hip_fracture	261381	0	NA	NA	0.875469	0.342789
hip_fracture	261387	0	NA	NA	0.076961	0.459964
hip_fracture	261399	0	NA	NA	-0.31471	0.565197
hip_fracture	261399	0	NA	NA	-0.31471	0.565197
hip_fracture	261411	0	NA	NA	0.371564	0.263926
hip_fracture	261418	0	NA	NA	0.336472	0.239354
hip_fracture	261424	0	NA	NA	0.198851	0.240374
hip_fracture	261426	0	NA	NA	0.530628	0.209434
hip_fracture	261429	0	NA	NA	0.113329	0.093807
hip_fracture	261429	0	NA	NA	0.04879	0.210887
hip_fracture	261433	0	NA	NA	0.176471	0.349905
hip_fracture	261473	0	NA	NA	0.04879	0.214558
hip_fracture	261498	0	NA	NA	0.09531	0.142759
hip_fracture	315458	0	NA	NA	0.756122	0.333323
hip_fracture	315458	0	NA	NA	0.438255	0.422302

hip_fracture	394006	0	NA	NA	0.524729	0.193836
hip_fracture	394006	0	NA	NA	0.122218	0.062975
hip_fracture	394006	0	NA	NA	0.00995	0.075578
hip_fracture	394006	0	NA	NA	0.398776	0.109042
hip_fracture	394006	0	NA	NA	0.431782	0.121737
hip_fracture	394006	0	NA	NA	0.122218	0.054386
hip_fracture	394006	0	NA	NA	0.438255	0.147737
hip_fracture	394006	0	NA	NA	0.019803	0.06474
hip_fracture	394006	0	NA	NA	0.470004	0.118388
hip_fracture	414192	0	NA	NA	1.128171	0.317378
hip_fracture	414192	0	NA	NA	0.71784	0.339921
hip_fracture	414194	1	NA	NA	1.477049	0.385339
hip_fracture	414201	0	NA	NA	0.29267	0.155328
hip_fracture	414201	0	NA	NA	0.385262	0.170676
hip_fracture	414201	0	NA	NA	0.916291	0.233748
hip_fracture	414201	0	NA	NA	-0.19845	0.345939
hip_fracture	414201	0	NA	NA	0.113329	0.284213
hip_fracture	414201	0	NA	NA	0.207014	0.421664
hip_fracture	414206	0	NA	NA	0.845868	0.158262
hip_fracture	414208	0	NA	NA	0.774727	0.246375
hip_fracture	414210	1	NA	NA	-0.37106	0.263102
hip_fracture	414210	0	NA	NA	-0.37106	0.604994
hip_fracture	414210	0	NA	NA	-0.23572	0.400452
hip_fracture	414212	0	NA	NA	0.788457	0.273632
hip_fracture	414212	0	NA	NA	0.741937	0.753804
hip_fracture	414212	0	NA	NA	0.832909	0.254727
hip_fracture	414212	0	NA	NA	1.163151	0.762389
hip_fracture	414214	0	NA	NA	0.262364	0.258061
hip_fracture	414214	0	NA	NA	0.405465	0.260625
hip_fracture	414220	0	NA	NA	0.300105	0.097288
hip_fracture	414223	0	NA	NA	0.912283	0.221079
hip_fracture	414225	1	NA	NA	1.722767	0.574309
hip_fracture	414227	0	NA	NA	0.002996	0.447524
hip_fracture	414227	0	NA	NA	-0.15082	0.329899
hip_fracture	414227	1	NA	NA	-0.27444	0.210249
hip_fracture	414229	0	NA	NA	0.788457	0.53832
hip_fracture	498367	0	NA	NA	0.804241	0.206508
hip_fracture	498378	0	NA	NA	0.936093	0.276764
hip_fracture	498383	0	NA	NA	0.530628	0.625256
hip_fracture	498389	1	NA	NA	-0.69315	0.216147
hip_fracture	498389	0	NA	NA	-0.35667	0.176823
hip_fracture	498391	0	NA	NA	0.24686	0.348693
hip_fracture	498391	0	NA	NA	0.24686	0.274525

hip_fracture	498391	0	NA	NA	0.41211	0.329978
hip_fracture	498391	0	NA	NA	0.086178	0.277544
hip_fracture	498392	0	NA	NA	0.392042	0.141454
hip_fracture	498392	0	NA	NA	0.198851	0.111968
hip_fracture	498396	0	NA	NA	0.741937	0.229792
hip_fracture	498406	0	NA	NA	0.24686	0.22617
hip_fracture	498406	0	NA	NA	0.722706	0.259553
hip_fracture	498406	0	NA	NA	-0.08338	0.159087
hip_fracture	498406	0	NA	NA	0.314811	0.475311
hip_fracture	498406	0	NA	NA	0.371564	0.117111
hip_fracture	498406	0	NA	NA	0.559616	0.12013
hip_fracture	498410	0	NA	NA	0.405465	0.18927
hip_fracture	498418	1	NA	NA	1.108563	0.31012
hip_fracture	498418	1	NA	NA	0.996949	0.192127
hip_fracture	498421	0	NA	NA	0.672944	0.149366
hip_fracture	498421	0	NA	NA	0.039221	0.09245
hip_fracture	498421	0	NA	NA	0.113329	0.063538
hip_fracture	498421	0	NA	NA	0.113329	0.152663
hip_fracture	498421	0	NA	NA	0.336472	0.187132
hip_fracture	498421	0	NA	NA	0.223144	0.057384
hip_fracture	498421	0	NA	NA	0.615186	0.1378
hip_fracture	498421	0	NA	NA	0.131028	0.186331
hip_fracture	498421	0	NA	NA	0.457425	0.19615
hip_fracture	498421	0	NA	NA	0.165514	0.130873
hip_fracture	498421	0	NA	NA	0.760806	0.175977
hip_fracture	498421	0	NA	NA	0.113329	0.115563
hip_fracture	498427	0	NA	NA	-0.06188	0.219117
hip_fracture	498442	0	NA	NA	0.444045	0.313528
hip_fracture	498444	1	NA	NA	1.506962	0.025597
hip_fracture	498444	1	NA	NA	1.542015	0.031475
hip_fracture	498446	0	NA	NA	0.390013	0.18977
hip_fracture	498453	0	NA	NA	0.09531	0.17187
hip_fracture	501885	0	NA	NA	0.778774	0.137214
hip_fracture	501885	0	NA	NA	0.162799	0.156121
hip_fracture	501889	0	NA	NA	0.211314	0.096016
hip_fracture	501889	1	NA	NA	-0.26945	0.081555
hip_fracture	501903	0	NA	NA	0.14842	0.028496
hip_fracture	501925	0	NA	NA	0.336472	0.188392
hip_fracture	501925	0	NA	NA	0.48858	0.368541
hip_fracture	501929	0	NA	NA	0.198851	0.144339
hip_fracture	501929	0	NA	NA	0.378436	0.223334
hip_fracture	501938	1	NA	NA	-0.35027	0.01318
hip_fracture	501938	1	NA	NA	-0.31334	0.019259

hip_fracture	501938	0	NA	NA	0.339468	0.046156
hip_fracture	501952	0	NA	NA	0.405465	0.138244
hip_fracture	501954	0	NA	NA	0.39346	0.111003
hip_fracture	501956	0	NA	NA	0.584615	0.301446
hip_fracture	501960	0	NA	NA	-0.09475	0.163795
hip_fracture	501967	0	NA	NA	0.506818	0.131109
hip_fracture	501969	0	NA	NA	0.076127	0.032036
hip_fracture	132098	0	NA	NA	0.307485	0.172848
hip_fracture	132098	0	NA	NA	0.113329	0.258061
hip_fracture	165283	0	NA	NA	-0.24846	0.274394
hip_fracture	261365	0	NA	NA	0.587787	0.225099
hip_fracture	261365	0	NA	NA	0.587787	0.197242
hip_fracture	261375	0	NA	NA	0.307485	0.098838
hip_fracture	261375	0	NA	NA	0.463734	0.216498
hip_fracture	261377	0	NA	NA	0.604316	0.171908
hip_fracture	261377	0	NA	NA	0.802002	0.390152
hip_fracture	261381	0	NA	NA	0.788457	0.353647
hip_fracture	261381	0	NA	NA	0.875469	0.342789
hip_fracture	261387	0	NA	NA	0.076961	0.459964
hip_fracture	261399	0	NA	NA	-0.31471	0.565197
hip_fracture	261399	0	NA	NA	-0.31471	0.565197
hip_fracture	261411	0	NA	NA	0.371564	0.263926
hip_fracture	261418	0	NA	NA	0.336472	0.239354
hip_fracture	261424	0	NA	NA	0.198851	0.240374
hip_fracture	261426	0	NA	NA	0.530628	0.209434
hip_fracture	261429	0	NA	NA	0.113329	0.093807
hip_fracture	261429	0	NA	NA	0.04879	0.210887
hip_fracture	261433	0	NA	NA	0.176471	0.349905
hip_fracture	261473	0	NA	NA	0.04879	0.214558
hip_fracture	261498	0	NA	NA	0.09531	0.142759
hip_fracture	315458	0	NA	NA	0.756122	0.333323
hip_fracture	315458	0	NA	NA	0.438255	0.422302
hip_fracture	394006	0	NA	NA	0.524729	0.193836
hip_fracture	394006	0	NA	NA	0.122218	0.062975
hip_fracture	394006	0	NA	NA	0.00995	0.075578
hip_fracture	394006	0	NA	NA	0.398776	0.109042
hip_fracture	394006	0	NA	NA	0.431782	0.121737
hip_fracture	394006	0	NA	NA	0.122218	0.054386
hip_fracture	394006	0	NA	NA	0.438255	0.147737
hip_fracture	394006	0	NA	NA	0.019803	0.06474
hip_fracture	394006	0	NA	NA	0.470004	0.118388
hip_fracture	414192	0	NA	NA	1.128171	0.317378
hip_fracture	414192	0	NA	NA	0.71784	0.339921

hip_fracture	414194	1	NA	NA	1.477049	0.385339
hip_fracture	414201	0	NA	NA	0.29267	0.155328
hip_fracture	414201	0	NA	NA	0.385262	0.170676
hip_fracture	414201	0	NA	NA	0.916291	0.233748
hip_fracture	414201	0	NA	NA	-0.19845	0.345939
hip_fracture	414201	0	NA	NA	0.113329	0.284213
hip_fracture	414201	0	NA	NA	0.207014	0.421664
hip_fracture	414206	0	NA	NA	0.845868	0.158262
hip_fracture	414208	0	NA	NA	0.774727	0.246375
hip_fracture	414210	1	NA	NA	-0.37106	0.263102
hip_fracture	414210	0	NA	NA	-0.37106	0.604994
hip_fracture	414210	0	NA	NA	-0.23572	0.400452
hip_fracture	414212	0	NA	NA	0.788457	0.273632
hip_fracture	414212	0	NA	NA	0.741937	0.753804
hip_fracture	414212	0	NA	NA	0.832909	0.254727
hip_fracture	414212	0	NA	NA	1.163151	0.762389
hip_fracture	414214	0	NA	NA	0.262364	0.258061
hip_fracture	414214	0	NA	NA	0.405465	0.260625
hip_fracture	414220	0	NA	NA	0.300105	0.097288
hip_fracture	414223	0	NA	NA	0.912283	0.221079
hip_fracture	414225	1	NA	NA	1.722767	0.574309
hip_fracture	414227	0	NA	NA	0.002996	0.447524
hip_fracture	414227	0	NA	NA	-0.15082	0.329899
hip_fracture	414227	1	NA	NA	-0.27444	0.210249
hip_fracture	414229	0	NA	NA	0.788457	0.53832
hip_fracture	498367	0	NA	NA	0.804241	0.206508
hip_fracture	498378	0	NA	NA	0.936093	0.276764
hip_fracture	498383	0	NA	NA	0.530628	0.625256
hip_fracture	498389	1	NA	NA	-0.69315	0.216147
hip_fracture	498389	0	NA	NA	-0.35667	0.176823
hip_fracture	498391	0	NA	NA	0.24686	0.348693
hip_fracture	498391	0	NA	NA	0.24686	0.274525
hip_fracture	498391	0	NA	NA	0.41211	0.329978
hip_fracture	498391	0	NA	NA	0.086178	0.277544
hip_fracture	498392	0	NA	NA	0.392042	0.141454
hip_fracture	498392	0	NA	NA	0.198851	0.111968
hip_fracture	498396	0	NA	NA	0.741937	0.229792
hip_fracture	498406	0	NA	NA	0.24686	0.22617
hip_fracture	498406	0	NA	NA	0.722706	0.259553
hip_fracture	498406	0	NA	NA	-0.08338	0.159087
hip_fracture	498406	0	NA	NA	0.314811	0.475311
hip_fracture	498406	0	NA	NA	0.371564	0.117111
hip_fracture	498406	0	NA	NA	0.559616	0.12013

hip_fracture	498410	0	NA	NA	0.405465	0.18927
hip_fracture	498418	1	NA	NA	1.108563	0.31012
hip_fracture	498418	1	NA	NA	0.996949	0.192127
hip_fracture	498421	0	NA	NA	0.672944	0.149366
hip_fracture	498421	0	NA	NA	0.039221	0.09245
hip_fracture	498421	0	NA	NA	0.113329	0.063538
hip_fracture	498421	0	NA	NA	0.113329	0.152663
hip_fracture	498421	0	NA	NA	0.336472	0.187132
hip_fracture	498421	0	NA	NA	0.223144	0.057384
hip_fracture	498421	0	NA	NA	0.615186	0.1378
hip_fracture	498421	0	NA	NA	0.131028	0.186331
hip_fracture	498421	0	NA	NA	0.457425	0.19615
hip_fracture	498421	0	NA	NA	0.165514	0.130873
hip_fracture	498421	0	NA	NA	0.760806	0.175977
hip_fracture	498421	0	NA	NA	0.113329	0.115563
hip_fracture	498427	0	NA	NA	-0.06188	0.219117
hip_fracture	498442	0	NA	NA	0.444045	0.313528
hip_fracture	498444	1	NA	NA	1.506962	0.025597
hip_fracture	498444	1	NA	NA	1.542015	0.031475
hip_fracture	498446	0	NA	NA	0.390013	0.18977
hip_fracture	498453	0	NA	NA	0.09531	0.17187
hip_fracture	501885	0	NA	NA	0.778774	0.137214
hip_fracture	501885	0	NA	NA	0.162799	0.156121
hip_fracture	501889	0	NA	NA	0.211314	0.096016
hip_fracture	501889	1	NA	NA	-0.26945	0.081555
hip_fracture	501903	0	NA	NA	0.14842	0.028496
hip_fracture	501925	0	NA	NA	0.336472	0.188392
hip_fracture	501925	0	NA	NA	0.48858	0.368541
hip_fracture	501929	0	NA	NA	0.198851	0.144339
hip_fracture	501929	0	NA	NA	0.378436	0.223334
hip_fracture	501938	1	NA	NA	-0.35027	0.01318
hip_fracture	501938	1	NA	NA	-0.31334	0.019259
hip_fracture	501938	0	NA	NA	0.339468	0.046156
hip_fracture	501952	0	NA	NA	0.405465	0.138244
hip_fracture	501954	0	NA	NA	0.39346	0.111003
hip_fracture	501956	0	NA	NA	0.584615	0.301446
hip_fracture	501960	0	NA	NA	-0.09475	0.163795
hip_fracture	501967	0	NA	NA	0.506818	0.131109
hip_fracture	501969	0	NA	NA	0.076127	0.032036
ihd	165539	0	0 cigarettes per day	1 to 9 cigarettes per day	0.182322	0.095663
ihd	165539	0	0 cigarettes per day	10 to 15 cigarettes per day	0.587787	0.070862
ihd	174238	0	0 cigarettes per day	25 to 37.5 cigarettes per day	0.587787	1.346372
ihd	236194	0	0 cigarettes per day	1 to 4 cigarettes per day	0.97456	0.140547

ihd	236194	0	0 cigarettes per day	5 to 9 cigarettes per day	0.982078	0.088856
ihd	236194	0	0 cigarettes per day	1 to 4 cigarettes per day	1.033184	0.27689
ihd	236194	1	0 cigarettes per day	10 to 14 cigarettes per day	1.175573	0.073224
ihd	236194	0	0 cigarettes per day	5 to 9 cigarettes per day	1.305626	0.163155
ihd	236194	0	0 cigarettes per day	26 to 39 cigarettes per day	1.358409	0.973847
ihd	236194	1	0 cigarettes per day	15 to 19 cigarettes per day	1.358409	0.077383
ihd	236194	0	0 cigarettes per day	10 to 14 cigarettes per day	1.393766	0.158885
ihd	236194	0	0 cigarettes per day	26 to 39 cigarettes per day	1.403643	0.10718
ihd	236194	1	0 cigarettes per day	20 to 24 cigarettes per day	1.410987	0.082753
ihd	236194	0	0 cigarettes per day	20 to 24 cigarettes per day	1.506297	0.315625
ihd	236194	0	0 cigarettes per day	15 to 19 cigarettes per day	1.757858	0.204521
ihd	236197	1	0 cigarettes per day	21 to 31.5 cigarettes per day	1.778336	0.034904
ihd	236197	1	0 cigarettes per day	1 to 9 cigarettes per day	1.160021	0.10396
ihd	236197	1	0 cigarettes per day	10 to 19 cigarettes per day	1.515127	0.026912
ihd	249384	1	0 cigarettes per day	10 to 19 cigarettes per day	-0.23572	0.122707
ihd	249384	0	0 cigarettes per day	20 to 30 cigarettes per day	-0.03046	0.228803
ihd	249384	0	0 cigarettes per day	0 to 9 cigarettes per day	0.09531	0.150742
ihd	249384	0	0 cigarettes per day	10 to 19 cigarettes per day	0.81093	0.124717
ihd	249384	0	0 cigarettes per day	0 to 9 cigarettes per day	0.875469	0.191327
ihd	249384	0	0 cigarettes per day	20 to 30 cigarettes per day	0.916291	0.163265
ihd	298268	0	0 cigarettes per day	1 to 19 cigarettes per day	0.832909	0.249556
ihd	298268	0	0 cigarettes per day	20 to 29 cigarettes per day	1.05779	0.204613
ihd	298268	0	0 cigarettes per day	31 to 46.5 cigarettes per day	1.48614	0.211815
ihd	309699	0	0 cigarettes per day	0 to 20 cigarettes per day	0.19062	0.267752
ihd	309699	1	0 cigarettes per day	21 to 31.5 cigarettes per day	0.231112	0.070862
ihd	309729	0	0 cigarettes per day	10 to 15 cigarettes per day	1.808289	0.2091
ihd	309729	0	0 cigarettes per day	5 to 9 cigarettes per day	2.451005	0.318878
ihd	309729	0	0 cigarettes per day	5 to 9 cigarettes per day	1.774952	0.410758
ihd	309729	0	0 cigarettes per day	5 to 9 cigarettes per day	1.360977	0.3663
ihd	309729	0	0 cigarettes per day	10 to 15 cigarettes per day	0.741937	0.340136
ihd	309729	0	0 cigarettes per day	10 to 15 cigarettes per day	1.740466	0.308808
ihd	328259	0	0 cigarettes per day	1 to 7 cigarettes per day	0.215111	0.059661
ihd	328259	0	0 cigarettes per day	1 to 7 cigarettes per day	0.385262	0.062474
ihd	328259	0	0 cigarettes per day	8 to 15 cigarettes per day	0.41871	0.060419
ihd	328259	0	0 cigarettes per day	8 to 15 cigarettes per day	0.425268	0.063359
ihd	328259	0	0 cigarettes per day	8 to 15 cigarettes per day	0.524729	0.08604
ihd	328259	0	0 cigarettes per day	16 to 25 cigarettes per day	0.530628	0.076531
ihd	328259	0	0 cigarettes per day	27 to 40.5 cigarettes per day	0.698135	0.159915
ihd	328259	0	0 cigarettes per day	27 to 40.5 cigarettes per day	0.722706	0.70958
ihd	328259	0	0 cigarettes per day	16 to 25 cigarettes per day	0.806476	0.228909
ihd	328274	0	0 cigarettes per day	20 to 30 cigarettes per day	0.693147	1.211735
ihd	328274	0	0 cigarettes per day	1 to 19 cigarettes per day	0.530628	1.42557
ihd	328460	0	0 cigarettes per day	20 to 30 cigarettes per day	1.10194	0.342396

ihd	328460	0	0 cigarettes per day	10 to 19 cigarettes per day	0.751416	0.34174
ihd	328460	0	0 cigarettes per day	0 to 9 cigarettes per day	0.41211	0.452764
ihd	328472	0	0 cigarettes per day	0 to 19 cigarettes per day	0.587787	1.346372
ihd	328472	0	0 cigarettes per day	20 to 30 cigarettes per day	0.741937	1.154033
ihd	330932	0	0 cigarettes per day	22 to 33 cigarettes per day	1.893112	0.981605
ihd	330932	0	0 cigarettes per day	1 to 9 cigarettes per day	0.039221	0.470958
ihd	330932	0	0 cigarettes per day	1 to 9 cigarettes per day	0.09531	0.310761
ihd	330932	0	0 cigarettes per day	10 to 20 cigarettes per day	0.322083	0.288376
ihd	330932	0	0 cigarettes per day	22 to 33 cigarettes per day	0.385262	0.494586
ihd	330932	0	0 cigarettes per day	10 to 20 cigarettes per day	0.891998	0.600117
ihd	331705	0	0 cigarettes per day	21 to 31.5 cigarettes per day	1.061257	0.062672
ihd	331705	0	0 cigarettes per day	21 to 31.5 cigarettes per day	1.111858	0.085593
ihd	331705	0	0 cigarettes per day	1 to 9 cigarettes per day	0.683097	0.065708
ihd	331705	0	0 cigarettes per day	10 to 19 cigarettes per day	0.841567	0.084667
ihd	332104	0	0 cigarettes per day	20 to 20 cigarettes per day	0.751416	0.117925
ihd	332104	0	0 cigarettes per day	22 to 33 cigarettes per day	0.832909	0.108696
ihd	332104	0	0 cigarettes per day	1 to 19 cigarettes per day	0.615186	0.111693
ihd	334410	0	0 cigarettes per day	1 to 25 cigarettes per day	0.779325	0.140554
ihd	334410	0	0 cigarettes per day	26 to 39 cigarettes per day	0.947789	0.134149
ihd	335266	0	0 cigarettes per day	1 to 9 cigarettes per day	0.122218	0.072241
ihd	335266	0	0 cigarettes per day	22 to 33 cigarettes per day	0.678034	0.063452
ihd	335762	0	0 cigarettes per day	1 to 19 cigarettes per day	0.518794	0.422133
ihd	335762	0	0 cigarettes per day	1 to 19 cigarettes per day	0.850151	0.199503
ihd	335762	0	0 cigarettes per day	21 to 31.5 cigarettes per day	0.996949	0.189209
ihd	335762	0	0 cigarettes per day	21 to 31.5 cigarettes per day	1.350667	0.524083
ihd	336216	0	0 cigarettes per day	40 to 60 cigarettes per day	1.286474	0.122618
ihd	336216	1	0 cigarettes per day	10 to 19 cigarettes per day	1.111858	0.060419
ihd	336216	0	0 cigarettes per day	40 to 60 cigarettes per day	1.05779	0.337479
ihd	336216	0	0 cigarettes per day	20 to 39 cigarettes per day	1.229641	0.079067
ihd	336216	1	0 cigarettes per day	0 to 9 cigarettes per day	0.904218	0.054738
ihd	336216	0	0 cigarettes per day	10 to 19 cigarettes per day	0.896088	0.049979
ihd	336216	0	0 cigarettes per day	20 to 39 cigarettes per day	1.004302	0.052329
ihd	336216	0	0 cigarettes per day	0 to 9 cigarettes per day	0.688135	0.062814
ihd	336319	0	0 cigarettes per day	15 to 24 cigarettes per day	0.524729	0.256694
ihd	336319	0	0 cigarettes per day	1 to 14 cigarettes per day	0.24686	0.240047
ihd	336319	0	0 cigarettes per day	25 to 37.5 cigarettes per day	0.641854	0.307381
ihd	336319	0	0 cigarettes per day	15 to 24 cigarettes per day	0.500775	0.24299
ihd	336319	0	0 cigarettes per day	1 to 14 cigarettes per day	0.524729	0.153324
ihd	336319	0	0 cigarettes per day	25 to 37.5 cigarettes per day	0.604316	0.276253
ihd	336682	0	0 cigarettes per day	1 to 14 cigarettes per day	0.65752	0.089881
ihd	336682	0	0 cigarettes per day	15 to 24 cigarettes per day	0.693147	0.089286
ihd	336682	0	0 cigarettes per day	26 to 39 cigarettes per day	0.806476	0.115024
ihd	336682	0	0 cigarettes per day	1 to 14 cigarettes per day	0.81978	0.103389

ihd	336682	0	0 cigarettes per day	15 to 24 cigarettes per day	1.291984	0.107928
ihd	336682	0	0 cigarettes per day	26 to 39 cigarettes per day	1.644805	0.201422
ihd	336801	0	0 cigarettes per day	20 to 20 cigarettes per day	0.524729	0.081512
ihd	336801	0	0 cigarettes per day	21 to 31.5 cigarettes per day	0.65752	0.058158
ihd	336801	0	0 cigarettes per day	0 to 19 cigarettes per day	0.385262	0.069416
ihd	336801	0	0 cigarettes per day	0 to 19 cigarettes per day	0.29267	0.097091
ihd	336801	0	0 cigarettes per day	20 to 20 cigarettes per day	0.223144	0.132449
ihd	336801	0	0 cigarettes per day	21 to 31.5 cigarettes per day	0.336472	0.210459
ihd	343310	0	0 cigarettes per day	15 to 24 cigarettes per day	1.147402	0.155491
ihd	343310	0	0 cigarettes per day	1 to 2 cigarettes per day	-0.27444	0.986842
ihd	343310	0	0 cigarettes per day	3 to 5 cigarettes per day	0.029559	0.366554
ihd	343310	0	0 cigarettes per day	1 to 2 cigarettes per day	0.336472	0.976676
ihd	343310	0	0 cigarettes per day	15 to 24 cigarettes per day	0.476234	0.148942
ihd	343310	0	0 cigarettes per day	10 to 14 cigarettes per day	0.518794	0.162476
ihd	343310	0	0 cigarettes per day	26 to 39 cigarettes per day	0.672944	0.1692
ihd	343310	0	0 cigarettes per day	6 to 9 cigarettes per day	0.741937	0.21137
ihd	343310	0	0 cigarettes per day	3 to 5 cigarettes per day	0.760806	0.360004
ihd	343310	0	0 cigarettes per day	6 to 9 cigarettes per day	0.891998	0.251966
ihd	343310	0	0 cigarettes per day	10 to 14 cigarettes per day	1.12493	0.159025
ihd	343310	0	0 cigarettes per day	26 to 39 cigarettes per day	1.238374	0.33496
ihd	343312	0	0 cigarettes per day	21 to 31.5 cigarettes per day	1.415853	0.492867
ihd	343312	0	0 cigarettes per day	1 to 9 cigarettes per day	0.636577	0.36983
ihd	343312	0	0 cigarettes per day	10 to 19 cigarettes per day	1.22083	0.377009
ihd	343400	0	0 cigarettes per day	1 to 4 cigarettes per day	0.662688	0.234162
ihd	343400	0	0 cigarettes per day	5 to 14 cigarettes per day	0.928219	0.129792
ihd	343400	1	0 cigarettes per day	15 to 24 cigarettes per day	1.439835	0.086652
ihd	343400	1	0 cigarettes per day	25 to 34 cigarettes per day	1.60342	0.103016
ihd	343400	0	0 cigarettes per day	35 to 44 cigarettes per day	1.717395	0.124753
ihd	343400	0	0 cigarettes per day	45 to 67.5 cigarettes per day	1.747459	0.273332
ihd	343461	0	0 cigarettes per day	20 to 30 cigarettes per day	0.667829	0.060178
ihd	343461	0	0 cigarettes per day	1 to 9 cigarettes per day	0.524729	0.08755
ihd	343461	0	0 cigarettes per day	10 to 19 cigarettes per day	0.81093	0.0839
ihd	343461	0	0 cigarettes per day	10 to 19 cigarettes per day	0.48858	0.061037
ihd	343461	0	0 cigarettes per day	1 to 9 cigarettes per day	0.518794	0.080479
ihd	343461	0	0 cigarettes per day	20 to 30 cigarettes per day	1.327075	0.133303
ihd	355955	0	0 cigarettes per day	26 to 39 cigarettes per day	1.007958	0.884478
ihd	355955	0	0 cigarettes per day	26 to 39 cigarettes per day	1.015231	0.878069
ihd	355955	0	0 cigarettes per day	26 to 39 cigarettes per day	0.904218	0.981162
ihd	355955	0	0 cigarettes per day	26 to 39 cigarettes per day	0.371564	1.671358
ihd	355955	0	0 cigarettes per day	25 to 37.5 cigarettes per day	0.751416	1.143146
ihd	355955	0	0 cigarettes per day	15 to 24 cigarettes per day	0.788457	1.101577
ihd	355955	0	0 cigarettes per day	1 to 14 cigarettes per day	-0.04082	2.524447
ihd	356107	1	0 cigarettes per day	15 to 24 cigarettes per day	0.262364	0.027473

ihd	356109	0	0 cigarettes per day	25 to 37.5 cigarettes per day	0.165514	2.053788
ihd	356109	0	0 cigarettes per day	1 to 14 cigarettes per day	0.506818	1.459921
ihd	356109	0	0 cigarettes per day	15 to 24 cigarettes per day	0.518794	1.442541
ihd	356109	0	0 cigarettes per day	25 to 37.5 cigarettes per day	0.518794	1.442541
ihd	356109	0	0 cigarettes per day	1 to 14 cigarettes per day	0.548121	1.400849
ihd	356109	0	0 cigarettes per day	15 to 24 cigarettes per day	0.722706	1.176441
ihd	356114	0	0 cigarettes per day	16 to 24 cigarettes per day	0.647103	0.129554
ihd	356114	0	0 cigarettes per day	16 to 24 cigarettes per day	0.609766	0.09289
ihd	356114	0	0 cigarettes per day	5 to 14 cigarettes per day	0.500775	0.145331
ihd	356114	0	0 cigarettes per day	5 to 14 cigarettes per day	0.451076	0.092617
ihd	356114	0	0 cigarettes per day	1 to 4 cigarettes per day	0.41211	0.31761
ihd	356114	0	0 cigarettes per day	1 to 4 cigarettes per day	-0.06188	0.236105
ihd	356141	0	0 cigarettes per day	1 to 9 cigarettes per day	0.24686	0.079719
ihd	356141	0	0 cigarettes per day	10 to 19 cigarettes per day	0.438255	0.065833
ihd	356141	1	0 cigarettes per day	41 to 61.5 cigarettes per day	0.609766	0.069321
ihd	356141	0	0 cigarettes per day	20 to 29 cigarettes per day	0.615186	0.055157
ihd	356141	0	0 cigarettes per day	30 to 39 cigarettes per day	0.667829	0.078493
ihd	356143	0	0 cigarettes per day	30 to 39 cigarettes per day	0.887891	0.045142
ihd	356143	0	0 cigarettes per day	20 to 29 cigarettes per day	0.824175	0.021259
ihd	356143	0	0 cigarettes per day	10 to 19 cigarettes per day	0.760806	0.019073
ihd	356143	1	0 cigarettes per day	41 to 61.5 cigarettes per day	0.587787	0.063776
ihd	356143	0	0 cigarettes per day	41 to 61.5 cigarettes per day	1.054312	0.074664
ihd	356143	0	0 cigarettes per day	20 to 29 cigarettes per day	0.438255	0.064187
ihd	356143	0	0 cigarettes per day	10 to 19 cigarettes per day	0.425268	0.095038
ihd	356143	0	0 cigarettes per day	1 to 9 cigarettes per day	0.215111	0.065833
ihd	356143	0	0 cigarettes per day	1 to 9 cigarettes per day	0.512824	0.041244
ihd	357254	0	0 cigarettes per day	1 to 9 cigarettes per day	0.285179	0.038361
ihd	357254	0	0 cigarettes per day	1 to 9 cigarettes per day	0.29267	0.026652
ihd	357254	1	0 cigarettes per day	10 to 19 cigarettes per day	0.300105	0.047241
ihd	357254	1	0 cigarettes per day	21 to 31.5 cigarettes per day	0.41211	0.047304
ihd	357254	0	0 cigarettes per day	21 to 31.5 cigarettes per day	0.553885	0.058644
ihd	357264	0	0 cigarettes per day	1 to 25 cigarettes per day	0.506818	0.239988
ihd	357264	0	0 cigarettes per day	25 to 37.5 cigarettes per day	0.81978	0.407114
ihd	357264	0	0 cigarettes per day	1 to 25 cigarettes per day	0.385262	0.237559
ihd	357264	0	0 cigarettes per day	25 to 37.5 cigarettes per day	0.854415	0.392133
ihd	357274	1	0 cigarettes per day	40 to 60 cigarettes per day	4.312677	0.464577
ihd	357274	1	0 cigarettes per day	11 to 19 cigarettes per day	2.071913	0.216226
ihd	357274	1	0 cigarettes per day	20 to 39 cigarettes per day	2.641198	0.170007
ihd	357274	0	0 cigarettes per day	1 to 5 cigarettes per day	0.912283	0.449758
ihd	357274	0	0 cigarettes per day	6 to 10 cigarettes per day	1.403643	0.249461
ihd	357353	0	0 cigarettes per day	1 to 14 cigarettes per day	0.824175	0.253983
ihd	357353	0	0 cigarettes per day	15 to 24 cigarettes per day	1.780024	0.262845
ihd	357353	0	0 cigarettes per day	25 to 37.5 cigarettes per day	2.398804	0.429341

ihd	357373	0	0 cigarettes per day	1 to 10 cigarettes per day	0.636577	0.434618
ihd	357373	0	0 cigarettes per day	11 to 16.5 cigarettes per day	1.862529	0.356113
ihd	357410	0	0 cigarettes per day	20 to 30 cigarettes per day	1.756132	0.166984
ihd	357410	0	0 cigarettes per day	0 to 19 cigarettes per day	0.609766	0.15528
ihd	357413	0	0 cigarettes per day	0 to 19 cigarettes per day	-0.35667	0.619534
ihd	357413	0	0 cigarettes per day	0 to 19 cigarettes per day	0	0.382653
ihd	357413	0	0 cigarettes per day	20 to 30 cigarettes per day	1.029619	0.419096
ihd	357413	0	0 cigarettes per day	20 to 30 cigarettes per day	1.458615	0.355956
ihd	357427	0	0 cigarettes per day	26 to 39 cigarettes per day	2.122262	0.355615
ihd	357427	0	0 cigarettes per day	1 to 15 cigarettes per day	0.71295	0.34889
ihd	357427	0	0 cigarettes per day	16 to 25 cigarettes per day	1.23256	0.316089
ihd	357429	0	0 cigarettes per day	16 to 24 cigarettes per day	1.366092	0.184168
ihd	357429	0	0 cigarettes per day	16 to 24 cigarettes per day	2.209373	0.34639
ihd	357429	0	0 cigarettes per day	1 to 15 cigarettes per day	0.350657	0.251509
ihd	357429	0	0 cigarettes per day	1 to 15 cigarettes per day	0.845868	0.420426
ihd	357441	0	0 cigarettes per day	0 to 9 cigarettes per day	0.470004	0.355548
ihd	357441	0	0 cigarettes per day	10 to 15 cigarettes per day	1.902108	0.353716
ihd	357453	0	0 cigarettes per day	0 to 4 cigarettes per day	0.41871	0.209788
ihd	357453	1	0 cigarettes per day	5 to 7.5 cigarettes per day	1.128171	0.099894
ihd	357456	0	0 cigarettes per day	1 to 10 cigarettes per day	0.693147	0.318878
ihd	357456	0	0 cigarettes per day	11 to 20 cigarettes per day	0.993252	0.302343
ihd	357456	0	0 cigarettes per day	21 to 31.5 cigarettes per day	1.064711	0.580577
ihd	357927	0	0 cigarettes per day	0 to 9 cigarettes per day	1.163151	0.310906
ihd	357927	0	0 cigarettes per day	11 to 16.5 cigarettes per day	2.208274	0.364431
ihd	357948	0	0 cigarettes per day	35 to 52.5 cigarettes per day	2.302585	0.242347
ihd	357948	0	0 cigarettes per day	25 to 34 cigarettes per day	2.116256	0.276617
ihd	357948	0	0 cigarettes per day	1 to 14 cigarettes per day	-0.10536	0.510204
ihd	357948	0	0 cigarettes per day	15 to 24 cigarettes per day	1.458615	1.281443
ihd	357952	0	0 cigarettes per day	1 to 14 cigarettes per day	0.470004	0.223214
ihd	357952	0	0 cigarettes per day	15 to 22.5 cigarettes per day	0.832909	0.255102
ihd	357956	1	0 cigarettes per day	1 to 24 cigarettes per day	1.547563	0.134529
ihd	357956	1	0 cigarettes per day	25 to 37.5 cigarettes per day	2.484907	0.158374
ihd	357959	0	0 cigarettes per day	1 to 14 cigarettes per day	1.187843	0.250212
ihd	357959	0	0 cigarettes per day	15 to 24 cigarettes per day	1.711995	0.383802
ihd	357959	0	0 cigarettes per day	1 to 14 cigarettes per day	0.378436	0.139787
ihd	357959	0	0 cigarettes per day	15 to 24 cigarettes per day	0.966984	0.148792
ihd	357959	0	0 cigarettes per day	25 to 37.5 cigarettes per day	1.927164	0.210958
ihd	358054	0	0 cigarettes per day	0 to 20 cigarettes per day	0.875469	0.487883
ihd	358054	0	0 cigarettes per day	21 to 31.5 cigarettes per day	2.352327	0.654139
ihd	358066	0	0 cigarettes per day	15 to 24 cigarettes per day	1.722767	0.22777
ihd	358066	0	0 cigarettes per day	25 to 37.5 cigarettes per day	1.686399	0.212585
ihd	358066	0	0 cigarettes per day	25 to 37.5 cigarettes per day	2.282382	0.351416
ihd	358066	0	0 cigarettes per day	0 to 14 cigarettes per day	0.875469	0.223214

ihd	358066	0	0 cigarettes per day	0 to 14 cigarettes per day	0.741937	0.218659
ihd	358066	0	0 cigarettes per day	15 to 24 cigarettes per day	1.163151	0.191327
ihd	359113	0	0 cigarettes per day	0 to 24 cigarettes per day	0.693147	1.211735
ihd	359114	0	0 cigarettes per day	25 to 34 cigarettes per day	0.832909	1.053682
ihd	359114	0	0 cigarettes per day	1 to 14 cigarettes per day	0.993252	0.897581
ihd	359114	0	0 cigarettes per day	15 to 24 cigarettes per day	1.064711	0.835679
ihd	359114	0	0 cigarettes per day	35 to 52.5 cigarettes per day	1.648659	0.466052
ihd	359115	0	0 cigarettes per day	1 to 4 cigarettes per day	0.875469	0.42517
ihd	359115	0	0 cigarettes per day	5 to 14 cigarettes per day	0.916291	0.193878
ihd	359115	0	0 cigarettes per day	15 to 24 cigarettes per day	1.098612	0.127551
ihd	359115	0	0 cigarettes per day	35 to 44 cigarettes per day	1.589235	0.171803
ihd	359115	0	0 cigarettes per day	25 to 34 cigarettes per day	1.629241	0.17507
ihd	359115	0	0 cigarettes per day	45 to 67.5 cigarettes per day	3.091042	0.31308
ihd	359320	0	0 cigarettes per day	35 to 40 cigarettes per day	1.481605	0.249304
ihd	359320	0	0 cigarettes per day	45 to 67.5 cigarettes per day	1.609438	0.265306
ihd	359320	0	0 cigarettes per day	0 to 24 cigarettes per day	0.916291	0.234694
ihd	359320	0	0 cigarettes per day	25 to 35 cigarettes per day	1.064711	0.255102
ihd	432350	0	0 cigarettes per day	1 to 14 cigarettes per day	0.24686	0.045783
ihd	432350	1	0 cigarettes per day	15 to 24 cigarettes per day	0.392042	0.049977
ihd	432350	1	0 cigarettes per day	25 to 37.5 cigarettes per day	0.392042	0.07239
ihd	462792	0	0 cigarettes per day	25 to 37.5 cigarettes per day	1.205971	0.08132
ihd	462792	0	0 cigarettes per day	1 to 14 cigarettes per day	0.631272	0.089044
ihd	462792	0	0 cigarettes per day	15 to 24 cigarettes per day	0.936093	0.073677
ihd	462792	0	0 cigarettes per day	15 to 24 cigarettes per day	0.524729	0.055909
ihd	462792	0	0 cigarettes per day	1 to 14 cigarettes per day	0.239017	0.066399
ihd	462792	0	0 cigarettes per day	25 to 37.5 cigarettes per day	0.667829	0.062467
ihd	501896	0	0 cigarettes per day	1 to 19 cigarettes per day	0.500775	0.085047
ihd	501896	0	0 cigarettes per day	20 to 30 cigarettes per day	0.604316	0.07097
ihd	502077	0	0 cigarettes per day	1 to 9 cigarettes per day	0.207014	0.068018
ihd	502077	1	0 cigarettes per day	10 to 19 cigarettes per day	0.24686	0.060543
ihd	502077	0	0 cigarettes per day	20 to 30 cigarettes per day	0.708036	0.06427
ihd	502134	0	0 cigarettes per day	4 to 14 cigarettes per day	0.751416	0.077903
ihd	502134	0	0 cigarettes per day	1 to 4 cigarettes per day	0.457425	0.223542
ihd	502134	0	0 cigarettes per day	15 to 22.5 cigarettes per day	0.871293	0.088181
kidney_cancer	164492	0	0 pack-years	1 to 19 pack-years	-0.10536	0.226757
kidney_cancer	164492	0	0 pack-years	1 to 19 pack-years	0.182322	0.276361
kidney_cancer	164492	0	0 pack-years	40 to 60 pack-years	0.262364	0.274725
kidney_cancer	164492	0	0 pack-years	20 to 39 pack-years	0.336472	0.309767
kidney_cancer	164492	0	0 pack-years	20 to 39 pack-years	0.336472	0.23688
kidney_cancer	164492	0	0 pack-years	40 to 60 pack-years	0.693147	0.293367
kidney_cancer	164580	1	0 pack-years	0 to 20 pack-years	0.850151	0.250657
kidney_cancer	164580	0	0 pack-years	20 to 30 pack-years	0.815365	0.240532
kidney_cancer	164580	0	0 pack-years	0 to 20 pack-years	0.48858	0.312869

kidney_cancer	164580	0	0 pack-years	20 to 30 pack-years	0.615186	0.367439
kidney_cancer	328339	0	0 pack-years	60 to 90 pack-years	1.305626	0.766689
kidney_cancer	328339	0	0 pack-years	0 to 19 pack-years	1.075002	1.961587
kidney_cancer	328339	0	0 pack-years	40 to 59 pack-years	0.609766	0.651619
kidney_cancer	328339	0	0 pack-years	0 to 19 pack-years	-0.38566	2.108343
kidney_cancer	328339	0	0 pack-years	20 to 39 pack-years	0.741937	0.596453
kidney_cancer	343155	0	0 pack-years	21 to 40 pack-years	0.09531	0.440631
kidney_cancer	343155	0	0 pack-years	0 to 20 pack-years	0.262364	0.529827
kidney_cancer	343155	0	0 pack-years	21 to 40 pack-years	0.336472	0.400875
kidney_cancer	343155	0	0 pack-years	41 to 61.5 pack-years	0.587787	0.340136
kidney_cancer	343155	0	0 pack-years	41 to 61.5 pack-years	0.832909	0.28394
kidney_cancer	343155	0	0 pack-years	0 to 20 pack-years	1.131402	0.534891
kidney_cancer	343580	0	0 pack-years	75.05 to 112.5 pack-years	-0.10536	0.326769
kidney_cancer	343580	0	0 pack-years	50.05 to 75 pack-years	0	0.280258
kidney_cancer	343580	0	0 pack-years	25.05 to 50 pack-years	0	0.250212
kidney_cancer	343580	0	0 pack-years	0.05 to 25 pack-years	0.182322	0.280258
kidney_cancer	358807	0	0 pack-years	0 to 15.9 pack-years	2.408745	1.552865
kidney_cancer	358807	1	0 pack-years	34.3 to 51.45 pack-years	1.962908	0.489065
kidney_cancer	358807	1	0 pack-years	16 to 34.2 pack-years	1.754404	0.69469
kidney_cancer	358807	1	0 pack-years	34.3 to 51.45 pack-years	1.691939	0.363626
kidney_cancer	358807	0	0 pack-years	34.3 to 51.45 pack-years	1.451614	0.510802
kidney_cancer	358807	0	0 pack-years	0 to 15.9 pack-years	1.313724	0.757077
kidney_cancer	358807	1	0 pack-years	16 to 34.2 pack-years	1.244155	0.396254
kidney_cancer	358807	0	0 pack-years	34.3 to 51.45 pack-years	1.205971	0.355157
kidney_cancer	358807	0	0 pack-years	34.3 to 51.45 pack-years	1.121678	0.302466
kidney_cancer	358807	0	0 pack-years	16 to 34.2 pack-years	1.054312	0.477316
kidney_cancer	358807	0	0 pack-years	0 to 15.9 pack-years	0.932164	1.019404
kidney_cancer	358807	0	0 pack-years	16 to 34.2 pack-years	0.904218	0.415186
kidney_cancer	358807	1	0 pack-years	0 to 15.9 pack-years	0.698135	0.171337
kidney_cancer	358807	0	0 pack-years	0 to 15.9 pack-years	0.662688	0.429992
kidney_cancer	358807	0	0 pack-years	0 to 15.9 pack-years	0.518794	1.228438
kidney_cancer	358807	0	0 pack-years	16 to 34.2 pack-years	0.476234	0.264609
kidney_cancer	358807	0	0 pack-years	16 to 34.2 pack-years	0.239017	0.343484
kidney_cancer	358807	1	0 pack-years	34.3 to 51.45 pack-years	1.085189	0.239589
kidney_cancer	358809	0	0 pack-years	40 to 60 pack-years	0.765468	0.434267
kidney_cancer	358809	0	0 pack-years	0 to 19 pack-years	0.336472	0.428207
kidney_cancer	358809	0	0 pack-years	20 to 30 pack-years	0.792993	0.702973
kidney_cancer	358809	0	0 pack-years	0 to 9 pack-years	-0.73397	0.935374
kidney_cancer	358809	0	0 pack-years	10 to 19 pack-years	-1.17118	0.929888
kidney_cancer	358809	0	0 pack-years	20 to 39 pack-years	0.067659	0.371924
kidney_cancer	358812	0	0 pack-years	10 to 19 pack-years	-0.35667	0.255102
kidney_cancer	358812	0	0 pack-years	30 to 39 pack-years	-0.10536	0.42517
kidney_cancer	358814	0	0 pack-years	1 to 20 pack-years	-0.22314	0.57398

kidney_cancer	358814	0	0 pack-years	21 to 31.5 pack-years	0.875469	0.457058
kidney_cancer	358814	0	0 pack-years	1 to 20 pack-years	0.955511	0.441523
kidney_cancer	358814	1	0 pack-years	21 to 31.5 pack-years	1.526056	0.421473
kidney_cancer	413175	0	0 pack-years	31 to 40 pack-years	0.182322	0.162242
kidney_cancer	413175	0	0 pack-years	21 to 30 pack-years	0.182322	0.146777
kidney_cancer	413175	0	0 pack-years	40 to 60 pack-years	0.336472	0.163738
kidney_cancer	413175	0	0 pack-years	1 to 10 pack-years	0.09531	0.142759
kidney_cancer	413175	0	0 pack-years	11 to 20 pack-years	0.182322	0.734171
kidney_cancer	502130	0	0 pack-years	0 to 10 pack-years	0.34359	0.422625
kidney_cancer	502130	0	0 pack-years	10 to 19 pack-years	-0.94161	0.595759
kidney_cancer	502130	0	0 pack-years	20 to 29 pack-years	0.019803	0.384296
kidney_cancer	502130	0	0 pack-years	30 to 45 pack-years	0.34359	0.295914
kidney_cancer	502132	0	0 pack-years	1 to 19 pack-years	0.131028	0.244305
kidney_cancer	502132	0	0 pack-years	20 to 39 pack-years	0.00995	0.201818
kidney_cancer	502132	0	0 pack-years	40 to 60 pack-years	0.34359	0.212596
kidney_cancer	502217	0	0 pack-years	1 to 10 pack-years	-0.16252	0.536769
kidney_cancer	502217	0	0 pack-years	10 to 19 pack-years	0.41211	0.357025
kidney_cancer	502217	0	0 pack-years	20 to 29 pack-years	0.322083	0.381503
kidney_cancer	502217	0	0 pack-years	30 to 45 pack-years	0.09531	0.443802
kidney_cancer	502427	0	0 pack-years	20 to 30 pack-years	0.329304	0.18203
kidney_cancer	502427	0	0 pack-years	10 to 19.9 pack-years	0.039221	0.273191
kidney_cancer	502427	0	0 pack-years	1 to 4.9 pack-years	-0.44629	0.261697
kidney_cancer	502427	0	0 pack-years	5 to 9.9 pack-years	-0.4943	0.357863
kidney_cancer	502441	0	0 pack-years	1 to 19 pack-years	-0.35667	0.392971
kidney_cancer	502441	0	0 pack-years	20 to 39 pack-years	0.182322	0.33145
kidney_cancer	502441	0	0 pack-years	40 to 69 pack-years	0.182322	0.353647
kidney_cancer	502441	0	0 pack-years	70 to 105 pack-years	0.641854	0.457081
kidney_cancer	502441	0	0 pack-years	1 to 19 pack-years	0	0.280258
kidney_cancer	502441	0	0 pack-years	20 to 39 pack-years	0.336472	0.344369
kidney_cancer	502441	0	0 pack-years	40 to 60 pack-years	0.405465	0.57161
kidney_cancer	502448	0	0 pack-years	1 to 17 pack-years	-0.27444	0.19344
kidney_cancer	502448	1	0 pack-years	31 to 45.5 pack-years	-0.16252	0.127983
kidney_cancer	502448	0	0 pack-years	17 to 31 pack-years	-0.05129	0.12544
kidney_cancer	502448	0	0 pack-years	1 to 17 pack-years	-0.16252	0.117027
kidney_cancer	502448	0	0 pack-years	31 to 45.5 pack-years	-0.16252	0.380843
kidney_cancer	502448	1	0 pack-years	31 to 45.5 pack-years	-0.11653	0.146538
kidney_cancer	502448	0	0 pack-years	17 to 31 pack-years	0	0.148049
kidney_cancer	502448	0	0 pack-years	1 to 17 pack-years	-0.06188	0.149946
kidney_cancer	502448	0	0 pack-years	17 to 31 pack-years	-0.01005	0.262349
kidney_cancer	502478	0	0 pack-years	28 to 42.2 pack-years	0.336472	0.139424
kidney_cancer	502478	0	0 pack-years	1 to 15.9 pack-years	0.09531	0.160359
kidney_cancer	502478	0	0 pack-years	16 to 27.9 pack-years	0.09531	0.160359
kidney_cancer	502478	0	0 pack-years	42.3 to 63.45 pack-years	0.693147	0.133482

kidney_cancer	502480	0	0 pack-years	11 to 20 pack-years	0.955511	0.608792
kidney_cancer	502480	0	0 pack-years	1 to 10 pack-years	1.029619	0.584544
kidney_cancer	502480	0	0 pack-years	20 to 30 pack-years	0.641854	0.440618
laryngeal_cancer	298142	0	0 pack-years	1 to 10 pack-years	0.641854	0.228249
laryngeal_cancer	298142	0	0 pack-years	11 to 20 pack-years	1.481605	0.220315
laryngeal_cancer	298142	0	0 pack-years	21 to 31.5 pack-years	1.791759	0.242347
laryngeal_cancer	298147	0	0 pack-years	0 to 9 pack-years	0.336472	0.747085
laryngeal_cancer	298147	0	0 pack-years	10 to 19 pack-years	1.064711	0.59817
laryngeal_cancer	298147	1	0 pack-years	20 to 29 pack-years	1.131402	0.617182
laryngeal_cancer	298147	0	0 pack-years	30 to 39 pack-years	2.734368	0.556586
laryngeal_cancer	298147	0	0 pack-years	40 to 60 pack-years	3.222868	0.517318
laryngeal_cancer	343375	0	0 pack-years	41 to 61.5 pack-years	2.791165	0.696444
laryngeal_cancer	343375	0	0 pack-years	21 to 40 pack-years	2.24071	0.635041
laryngeal_cancer	343375	0	0 pack-years	21 to 40 pack-years	2.186051	0.696515
laryngeal_cancer	343375	0	0 pack-years	41 to 61.5 pack-years	2.140066	0.627251
laryngeal_cancer	343375	0	0 pack-years	1 to 20 pack-years	2.04122	1.371588
laryngeal_cancer	343375	0	0 pack-years	1 to 20 pack-years	1.987874	0.69891
laryngeal_cancer	343375	1	0 pack-years	21 to 31.5 pack-years	0.875469	1.349915
laryngeal_cancer	343375	0	0 pack-years	1 to 20 pack-years	2.104134	0.609756
laryngeal_cancer	364113	0	0 pack-years	1 to 29 pack-years	1.950187	0.163294
laryngeal_cancer	364113	0	0 pack-years	30 to 39 pack-years	2.66026	0.190881
laryngeal_cancer	364113	0	0 pack-years	40 to 49 pack-years	2.821379	0.220177
laryngeal_cancer	364113	0	0 pack-years	60 to 90 pack-years	3.077312	0.306828
laryngeal_cancer	364113	0	0 pack-years	50 to 59 pack-years	3.144152	0.269397
laryngeal_cancer	502130	0	0 pack-years	20 to 29 pack-years	2.933325	0.677798
laryngeal_cancer	502130	1	0 pack-years	0 to 10 pack-years	2.515274	0.876388
laryngeal_cancer	502130	0	0 pack-years	10 to 19 pack-years	2.60343	0.717491
laryngeal_cancer	502130	0	0 pack-years	30 to 45 pack-years	3.125005	0.620322
lbp	259680	0	0 cigarettes per day	1 to 9 cigarettes per day	0.48858	0.311444
lbp	348202	1	0 cigarettes per day	11 to 20 cigarettes per day	-0.10536	0.226757
lbp	348202	0	0 cigarettes per day	21 to 31.5 cigarettes per day	0.09531	0.347866
lbp	348202	0	0 cigarettes per day	11 to 20 cigarettes per day	0.182322	0.510204
lbp	348202	0	0 cigarettes per day	1 to 10 cigarettes per day	0.182322	0.552721
lbp	348202	0	0 cigarettes per day	1 to 10 cigarettes per day	0.182322	0.233844
lbp	348202	0	0 cigarettes per day	21 to 31.5 cigarettes per day	1.704748	0.496289
lbp	348204	0	0 cigarettes per day	1 to 25 cigarettes per day	0.824175	0.37594
lbp	348204	0	0 cigarettes per day	25 to 37.5 cigarettes per day	1.329724	1.126363
lbp	348206	0	0 cigarettes per day	1 to 9 cigarettes per day	0.985817	0.377894
lbp	348206	0	0 cigarettes per day	10 to 15 cigarettes per day	0.943906	0.53899
lbp	348206	0	0 cigarettes per day	10 to 15 cigarettes per day	0.779325	0.471588
lbp	348206	0	0 cigarettes per day	1 to 9 cigarettes per day	0.871293	0.286056
lbp	348208	0	0 cigarettes per day	1 to 10 cigarettes per day	0.470004	0.165816
lbp	348208	0	0 cigarettes per day	11 to 20 cigarettes per day	0.756122	0.156894

lbp	348208	0	0 cigarettes per day	21 to 31.5 cigarettes per day	1.386294	0.852679
lbp	409803	0	0 cigarettes per day	21 to 30 cigarettes per day	0.029559	3.118313
lbp	409803	1	0 cigarettes per day	11 to 20 cigarettes per day	-0.31471	4.399811
lbp	409803	0	0 cigarettes per day	1 to 10 cigarettes per day	0.29267	2.396912
leukemia	328339	0	0 pack-years	40 to 59 pack-years	0.688135	0.893498
leukemia	328339	0	0 pack-years	20 to 39 pack-years	0.883768	0.729465
leukemia	328339	0	0 pack-years	0 to 19 pack-years	0.963174	1.040855
leukemia	328339	1	0 pack-years	20 to 39 pack-years	1.108563	1.877484
leukemia	328339	0	0 pack-years	61 to 91.5 pack-years	1.644805	0.971653
leukemia	343390	0	0 pack-years	10 to 19 pack-years	1.075002	1.229365
leukemia	343390	1	0 pack-years	0 to 9 pack-years	0.920283	2.031669
leukemia	343390	0	0 pack-years	20 to 29 pack-years	0.845868	1.579881
leukemia	343390	0	0 pack-years	20 to 29 pack-years	0.845868	1.557984
leukemia	343390	0	0 pack-years	31 to 46.5 pack-years	0.703098	0.386442
leukemia	343390	0	0 pack-years	31 to 46.5 pack-years	0.542324	0.364855
leukemia	343390	0	0 pack-years	20 to 29 pack-years	0.451076	1.298258
leukemia	343390	0	0 pack-years	0 to 9 pack-years	0.398776	0.58896
leukemia	343390	0	0 pack-years	31 to 46.5 pack-years	0.314811	0.657307
leukemia	343390	0	0 pack-years	10 to 19 pack-years	0.29267	0.969007
leukemia	343390	0	0 pack-years	31 to 46.5 pack-years	0.223144	1.032653
leukemia	343390	0	0 pack-years	20 to 29 pack-years	0.039221	0.537186
leukemia	343390	1	0 pack-years	0 to 9 pack-years	-0.46204	2.040816
leukemia	343390	1	0 pack-years	20 to 29 pack-years	-1.20397	2.142857
leukemia	343390	0	0 pack-years	10 to 19 pack-years	0.405465	0.52381
leukemia	502130	0	0 pack-years	10 to 19 pack-years	0.41871	0.38132
leukemia	502130	0	0 pack-years	30 to 45 pack-years	0.182322	0.398217
leukemia	502130	0	0 pack-years	20 to 29 pack-years	-0.11653	0.519266
leukemia	502130	0	0 pack-years	0 to 10 pack-years	0.307485	0.520517
leukemia	502130	0	0 pack-years	0 to 10 pack-years	-0.46204	0.727712
leukemia	502130	1	0 pack-years	20 to 29 pack-years	-1.60944	0.983983
leukemia	502130	0	0 pack-years	10 to 19 pack-years	0.350657	0.376078
leukemia	502130	0	0 pack-years	30 to 45 pack-years	-0.30111	0.462688
leukemia	502184	0	0 pack-years	1 to 9 pack-years	0.559616	0.393645
leukemia	502184	0	0 pack-years	10 to 19 pack-years	0.378436	0.517511
leukemia	502184	0	0 pack-years	20 to 30 pack-years	0.565314	0.411216
leukemia	502184	0	0 pack-years	1 to 9 pack-years	0.405465	0.353647
leukemia	502184	0	0 pack-years	10 to 19 pack-years	0.270027	0.419219
leukemia	502184	0	0 pack-years	20 to 30 pack-years	-0.17435	0.402385
leukemia	502395	0	0 pack-years	1 to 19 pack-years	-0.0202	0.283277
leukemia	502395	0	0 pack-years	20 to 30 pack-years	0.09531	0.307136
leukemia	502397	0	0 pack-years	1 to 20 pack-years	0.239017	0.152645
leukemia	502397	0	0 pack-years	21 to 31.5 pack-years	0.254642	0.155844
leukemia	502419	0	0 pack-years	11 to 20 pack-years	-0.10536	0.106644

leukemia	502419	0	0 pack-years	1 to 10 pack-years	0	0.080698
leukemia	502419	0	0 pack-years	20 to 30 pack-years	0.09531	0.093106
lip_oral_cavity_cancer	286841	0	0 pack-years	1 to 19 pack-years	0.182322	0.220663
lip_oral_cavity_cancer	286841	0	0 pack-years	20 to 39 pack-years	0.336472	0.280258
lip_oral_cavity_cancer	286841	0	0 pack-years	40 to 60 pack-years	0.262364	0.410571
lip_oral_cavity_cancer	343375	1	0 pack-years	1 to 20 pack-years	1.757858	0.340561
lip_oral_cavity_cancer	343375	1	0 pack-years	21 to 40 pack-years	2.163323	0.362394
lip_oral_cavity_cancer	343375	0	0 pack-years	41 to 61.5 pack-years	1.808289	0.399397
lip_oral_cavity_cancer	343432	0	0 pack-years	1 to 20 pack-years	0.270027	0.426194
lip_oral_cavity_cancer	343432	0	0 pack-years	21 to 31.5 pack-years	1.621366	0.535642
lip_oral_cavity_cancer	348052	0	0 pack-years	34 to 51 pack-years	1.131402	0.394997
lip_oral_cavity_cancer	348052	0	0 pack-years	34 to 51 pack-years	0.530628	0.45018
lip_oral_cavity_cancer	348052	0	0 pack-years	0 to 18 pack-years	0.916291	0.428571
lip_oral_cavity_cancer	359126	0	0 pack-years	0 to 19 pack-years	0.14842	0.134148
lip_oral_cavity_cancer	359126	1	0 pack-years	20 to 39 pack-years	0.277632	0.086967
lip_oral_cavity_cancer	359126	0	0 pack-years	40 to 60 pack-years	1.075002	0.165425
lip_oral_cavity_cancer	359128	0	0 pack-years	40 to 59 pack-years	0.928219	0.45878
lip_oral_cavity_cancer	359128	0	0 pack-years	60 to 90 pack-years	1.648659	0.48175
lip_oral_cavity_cancer	359128	0	0 pack-years	0 to 19 pack-years	0.81978	0.54167
lip_oral_cavity_cancer	359128	0	0 pack-years	40 to 59 pack-years	-1.13943	2.112564
lip_oral_cavity_cancer	359128	0	0 pack-years	0 to 19 pack-years	-0.26136	1.232441
lip_oral_cavity_cancer	359128	0	0 pack-years	20 to 39 pack-years	-1.30933	1.209373
lip_oral_cavity_cancer	359128	0	0 pack-years	20 to 39 pack-years	0.350657	0.45092
lip_oral_cavity_cancer	364113	1	0 pack-years	40 to 49 pack-years	1.957274	0.194569
lip_oral_cavity_cancer	502130	0	0 pack-years	0 to 10 pack-years	0.270027	0.601656
lip_oral_cavity_cancer	502130	0	0 pack-years	10 to 19 pack-years	0.792993	0.368691
lip_oral_cavity_cancer	502130	0	0 pack-years	20 to 29 pack-years	0.536493	0.434567
lip_oral_cavity_cancer	502130	0	0 pack-years	30 to 45 pack-years	1.585145	0.258849
lip_oral_cavity_cancer	502243	0	0 pack-years	1 to 20 pack-years	0.841567	0.377228
lip_oral_cavity_cancer	502243	0	0 pack-years	21 to 40 pack-years	1.677097	0.381556
lip_oral_cavity_cancer	502243	0	0 pack-years	40 to 60 pack-years	2.152924	0.364157
lip_oral_cavity_cancer	502352	0	0 pack-years	10 to 19 pack-years	0.058269	0.591991
lip_oral_cavity_cancer	502352	0	0 pack-years	1 to 9 pack-years	0.270027	0.822707
lip_oral_cavity_cancer	502352	0	0 pack-years	20 to 30 pack-years	1.678964	0.548627
liver_cancer	125938	0	0 pack-years	0 to 19 pack-years	0.182322	0.170068
liver_cancer	125938	1	0 pack-years	20 to 30 pack-years	4.154185	0.510605
liver_cancer	293871	0	0 pack-years	11 to 16.5 pack-years	-0.10536	0.255102
liver_cancer	293871	0	0 pack-years	0 to 10 pack-years	0.405465	0.272109
liver_cancer	328266	0	0 pack-years	0 to 19 pack-years	0.530628	0.285114
liver_cancer	328266	0	0 pack-years	20 to 40 pack-years	0.741937	0.279397
liver_cancer	328266	0	0 pack-years	41 to 61.5 pack-years	0.916291	0.336735
liver_cancer	328339	0	0 pack-years	60 to 90 pack-years	1.530395	1.797862
liver_cancer	328339	0	0 pack-years	40 to 59 pack-years	1.453953	0.73014

liver_cancer	328339	0	0 pack-years	20 to 39 pack-years	0.904218	0.467859
liver_cancer	328339	0	0 pack-years	60 to 90 pack-years	0.593327	0.243827
liver_cancer	328339	0	0 pack-years	0 to 19 pack-years	0.506818	0.235124
liver_cancer	328339	0	0 pack-years	20 to 39 pack-years	0.482426	0.165344
liver_cancer	328339	0	0 pack-years	40 to 59 pack-years	0.165514	0.196731
liver_cancer	328339	0	0 pack-years	0 to 19 pack-years	0	0.517857
liver_cancer	343515	1	0 pack-years	50 to 75 pack-years	-0.22314	3.891103
liver_cancer	343515	1	0 pack-years	1 to 24 pack-years	-0.10536	3.458759
liver_cancer	343515	0	0 pack-years	25 to 49 pack-years	0.955511	1.197263
liver_cancer	343580	0	0 pack-years	75.05 to 112.5 pack-years	-0.22314	0.560516
liver_cancer	343580	0	0 pack-years	50.05 to 75 pack-years	-0.35667	0.611708
liver_cancer	343580	0	0 pack-years	25.05 to 50 pack-years	-0.35667	0.470874
liver_cancer	343580	0	0 pack-years	0.05 to 25 pack-years	0.336472	0.517385
liver_cancer	343580	0	0 pack-years	25 to 50 pack-years	-0.35667	0.58309
liver_cancer	343580	0	0 pack-years	76 to 100 pack-years	-0.22314	0.765306
liver_cancer	343580	0	0 pack-years	50 to 75 pack-years	-0.35667	0.728863
liver_cancer	343580	0	0 pack-years	0.05 to 25 pack-years	0.336472	0.601312
liver_cancer	343586	0	0 pack-years	0 to 24 pack-years	0.693147	1.556441
liver_cancer	343586	0	0 pack-years	25 to 49 pack-years	1.013054	1.130313
liver_cancer	343586	0	0 pack-years	50 to 75 pack-years	1.064366	1.073778
liver_cancer	343586	0	0 pack-years	0 to 24 pack-years	1.48024	0.708439
liver_cancer	343588	0	0 pack-years	0 to 19 pack-years	0.131028	0.373702
liver_cancer	343588	0	0 pack-years	40 to 60 pack-years	0.086178	0.372121
liver_cancer	343588	0	0 pack-years	20 to 39 pack-years	0.086178	0.369781
liver_cancer	343590	1	0 pack-years	21 to 31.5 pack-years	-0.35667	0.291545
liver_cancer	343590	0	0 pack-years	1 to 20 pack-years	-0.10536	0.340136
liver_cancer	343632	0	0 pack-years	13 to 19.5 pack-years	0.587787	0.410998
liver_cancer	343632	0	0 pack-years	0.1 to 12.9 pack-years	0.875469	0.403912
liver_cancer	502130	0	0 pack-years	20 to 29 pack-years	0.470004	0.352047
liver_cancer	502130	0	0 pack-years	10 to 19 pack-years	0.131028	0.393657
liver_cancer	502130	0	0 pack-years	30 to 45 pack-years	-0.22314	0.423364
lri	173863	0	0 cigarettes per day	1 to 14 cigarettes per day	0.463734	1.707098
lri	173863	0	0 cigarettes per day	15 to 24 cigarettes per day	0.774727	1.250823
lri	173863	0	0 cigarettes per day	25 to 37.5 cigarettes per day	0.8671	1.140456
lri	236197	0	0 cigarettes per day	0 to 9 cigarettes per day	0.722706	1.317614
lri	236197	0	0 cigarettes per day	10 to 19 cigarettes per day	1.118415	0.887021
lri	236197	0	0 cigarettes per day	20 to 30 cigarettes per day	1.48614	0.614092
lri	298305	0	0 cigarettes per day	0 to 24 cigarettes per day	0.350657	0.269474
lri	298305	0	0 cigarettes per day	25 to 37.5 cigarettes per day	0.378436	0.396631
lri	298305	0	0 cigarettes per day	0 to 24 cigarettes per day	0.512824	0.172614
lri	298305	0	0 cigarettes per day	25 to 37.5 cigarettes per day	0.932164	0.320384
lri	355108	0	0 cigarettes per day	15 to 24 cigarettes per day	1.308333	0.413679
lri	355108	0	0 cigarettes per day	25 to 37.5 cigarettes per day	1.704748	0.482375

lri	355108	0	0 cigarettes per day	1 to 14 cigarettes per day	0.832909	0.332742
lri	355112	0	0 cigarettes per day	1 to 9 cigarettes per day	0.215111	0.357966
lri	355112	0	0 cigarettes per day	10 to 20 cigarettes per day	0.858662	0.291854
lri	355112	0	0 cigarettes per day	21 to 31.5 cigarettes per day	1.088562	0.368481
lri	355955	1	0 cigarettes per day	25 to 37.5 cigarettes per day	-1.13943	8.482143
lri	355955	0	0 cigarettes per day	1 to 14 cigarettes per day	0.371564	1.871921
lri	355955	0	0 cigarettes per day	15 to 24 cigarettes per day	1.098612	0.904762
lri	413804	0	0 cigarettes per day	10 to 19 cigarettes per day	0.604316	0.179772
lri	413804	0	0 cigarettes per day	10 to 19 cigarettes per day	0.604316	0.184008
lri	413804	1	0 cigarettes per day	0 to 9 cigarettes per day	0.667829	0.074568
lri	413804	0	0 cigarettes per day	20 to 30 cigarettes per day	1.193922	0.176252
lri	413804	0	0 cigarettes per day	1 to 10 cigarettes per day	0.667829	0.18052
lri	413804	0	0 cigarettes per day	20 to 30 cigarettes per day	1.193922	0.172995
lung_cancer	193948	0	0 pack-years	33.33 to 57.33 pack-years	0.774727	0.951049
lung_cancer	193948	0	0 pack-years	3.33 to 32.33 pack-years	0.95935	0.892368
lung_cancer	193948	0	0 pack-years	58.33 to 87.495 pack-years	1.547563	1.105623
lung_cancer	193987	0	0 pack-years	1 to 39 pack-years	0.65752	0.278894
lung_cancer	193987	0	0 pack-years	1 to 39 pack-years	1.319086	0.187575
lung_cancer	193987	1	0 pack-years	40 to 60 pack-years	1.690096	0.121432
lung_cancer	193987	1	0 pack-years	40 to 60 pack-years	2.259678	0.067903
lung_cancer	250095	0	0 pack-years	40 to 60 pack-years	1.976855	0.358627
lung_cancer	250095	1	0 pack-years	60 to 90 pack-years	1.490654	0.146511
lung_cancer	250095	1	0 pack-years	40 to 59 pack-years	1.050822	0.126659
lung_cancer	250095	0	0 pack-years	20 to 39 pack-years	1.366092	0.292196
lung_cancer	250095	0	0 pack-years	0 to 19 pack-years	0.559616	0.325073
lung_cancer	250095	0	0 pack-years	0 to 19 pack-years	0.14842	0.255102
lung_cancer	250095	1	0 pack-years	20 to 39 pack-years	0.741937	0.13241
lung_cancer	286844	0	0 pack-years	0.05 to 12.5 pack-years	0.587787	0.481859
lung_cancer	286844	0	0 pack-years	12.5 to 25 pack-years	2.140066	0.381152
lung_cancer	286844	0	0 pack-years	26 to 39 pack-years	4.213608	0.434353
lung_cancer	298142	0	0 pack-years	1 to 10 pack-years	0.587787	0.198413
lung_cancer	298142	0	0 pack-years	11 to 20 pack-years	1.458615	0.201709
lung_cancer	298142	0	0 pack-years	21 to 31.5 pack-years	1.916923	0.22509
lung_cancer	321342	1	0 pack-years	0 to 25 pack-years	2.451005	0.07917
lung_cancer	321342	0	0 pack-years	25 to 40 pack-years	3.025291	0.070586
lung_cancer	328215	0	0 pack-years	35 to 52.5 pack-years	1.979621	0.37913
lung_cancer	328215	0	0 pack-years	21 to 34 pack-years	1.211941	0.378098
lung_cancer	328215	0	0 pack-years	1 to 10 pack-years	0.41871	0.530344
lung_cancer	328215	0	0 pack-years	11 to 15 pack-years	0.512824	0.597275
lung_cancer	328215	0	0 pack-years	16 to 20 pack-years	0.916291	0.495918
lung_cancer	328266	0	0 pack-years	0 to 19 pack-years	0.832909	0.354925
lung_cancer	328266	0	0 pack-years	20 to 40 pack-years	1.504077	0.323129
lung_cancer	328266	0	0 pack-years	41 to 61.5 pack-years	1.667707	0.346554

lung_cancer	328909	0	0 pack-years	0 to 9 pack-years	1.499623	0.334821
lung_cancer	328909	0	0 pack-years	10 to 18 pack-years	1.913977	0.267895
lung_cancer	328909	0	0 pack-years	20 to 28 pack-years	2.428336	0.252628
lung_cancer	328909	0	0 pack-years	30 to 45 pack-years	2.821974	0.243265
lung_cancer	334460	0	0 pack-years	0 to 25 pack-years	2.885917	0.251685
lung_cancer	334460	0	0 pack-years	0 to 25 pack-years	2.800325	0.488803
lung_cancer	334460	0	0 pack-years	16 to 24 pack-years	2.651833	0.288924
lung_cancer	334460	0	0 pack-years	16 to 24 pack-years	2.329227	0.358435
lung_cancer	334460	0	0 pack-years	1 to 4 pack-years	0.48858	0.583761
lung_cancer	334460	0	0 pack-years	6 to 14 pack-years	1.479329	0.298684
lung_cancer	334460	0	0 pack-years	1 to 4 pack-years	0.746688	0.315553
lung_cancer	334460	0	0 pack-years	6 to 14 pack-years	1.83737	0.244947
lung_cancer	343580	0	0 pack-years	50.05 to 75 pack-years	2.778819	0.328431
lung_cancer	343580	0	0 pack-years	75.05 to 112.5 pack-years	3.332205	0.335417
lung_cancer	343580	0	0 pack-years	25.05 to 50 pack-years	2.292535	0.321637
lung_cancer	343580	0	0 pack-years	0.05 to 25 pack-years	1.481605	0.347782
lung_cancer	343580	0	0 pack-years	50 to 75 pack-years	2.778819	0.35334
lung_cancer	343580	0	0 pack-years	25 to 50 pack-years	2.292535	0.34529
lung_cancer	343580	0	0 pack-years	0.05 to 25 pack-years	1.481605	0.371058
lung_cancer	343580	0	0 pack-years	76 to 100 pack-years	3.332205	0.359876
lung_cancer	355961	0	0 pack-years	0 to 30 pack-years	1.84055	0.364431
lung_cancer	355961	0	0 pack-years	31 to 45 pack-years	2.197225	0.348639
lung_cancer	355961	0	0 pack-years	47 to 70.5 pack-years	3.148453	0.326268
lung_cancer	355963	0	0 pack-years	20 to 39 pack-years	2.24071	0.369084
lung_cancer	355963	0	0 pack-years	1 to 19 pack-years	1.064711	0.510204
lung_cancer	355963	0	0 pack-years	40 to 60 pack-years	2.867899	0.330473
lung_cancer	355965	0	0 pack-years	0 to 25 pack-years	1.458615	0.40935
lung_cancer	355965	0	0 pack-years	25 to 49.99 pack-years	2.282382	0.314973
lung_cancer	355965	0	0 pack-years	50 to 75 pack-years	3.148453	0.316414
lung_cancer	355970	0	0 pack-years	40 to 49 pack-years	3.478158	0.100781
lung_cancer	355970	0	0 pack-years	50 to 59 pack-years	3.835142	0.116256
lung_cancer	355970	0	0 pack-years	60 to 90 pack-years	3.246491	0.307711
lung_cancer	355970	0	0 pack-years	60 to 90 pack-years	3.864931	0.109635
lung_cancer	355970	0	0 pack-years	50 to 59 pack-years	2.884801	0.277904
lung_cancer	355970	0	0 pack-years	30 to 39 pack-years	3.202746	0.085034
lung_cancer	355970	0	0 pack-years	40 to 49 pack-years	2.639057	0.215015
lung_cancer	355970	0	0 pack-years	30 to 39 pack-years	2.557227	0.138427
lung_cancer	355970	1	0 pack-years	1 to 19 pack-years	2.186051	0.091722
lung_cancer	355970	0	0 pack-years	20 to 29 pack-years	1.987874	0.118815
lung_cancer	355970	0	0 pack-years	1 to 19 pack-years	1.252763	0.102041
lung_cancer	355970	0	0 pack-years	20 to 29 pack-years	2.839078	0.086526
lung_cancer	357351	0	0 pack-years	0 to 20 pack-years	1.743969	1.044491
lung_cancer	357351	0	0 pack-years	0 to 20 pack-years	1.989243	0.690974

lung_cancer	357351	0	0 pack-years	20 to 40 pack-years	2.119863	0.681395
lung_cancer	357351	0	0 pack-years	41 to 61.5 pack-years	2.784394	0.775706
lung_cancer	357351	0	0 pack-years	21 to 31.5 pack-years	2.995232	0.960429
lung_cancer	357367	0	0 pack-years	41 to 61.5 pack-years	2.642622	0.297044
lung_cancer	357367	0	0 pack-years	41 to 61.5 pack-years	2.427454	0.858296
lung_cancer	357367	0	0 pack-years	20 to 40 pack-years	2.273156	0.28479
lung_cancer	357367	0	0 pack-years	0 to 20 pack-years	0.57098	0.33293
lung_cancer	357367	0	0 pack-years	0 to 20 pack-years	1.329724	0.288171
lung_cancer	357367	0	0 pack-years	20 to 40 pack-years	1.65058	0.383388
lung_cancer	357400	0	0 pack-years	1 to 29 pack-years	1.335001	0.268528
lung_cancer	357400	0	0 pack-years	30 to 50 pack-years	2.028148	0.268528
lung_cancer	357400	0	0 pack-years	51 to 85 pack-years	2.541602	0.269163
lung_cancer	357400	0	0 pack-years	86 to 100 pack-years	2.701361	0.272223
lung_cancer	357402	0	0 pack-years	40 to 60 pack-years	1.678964	0.206081
lung_cancer	357402	0	0 pack-years	20 to 39.95 pack-years	1.272566	0.190076
lung_cancer	357402	0	0 pack-years	40 to 60 pack-years	1.713798	0.184317
lung_cancer	357402	0	0 pack-years	0 to 19 pack-years	0.65752	0.174474
lung_cancer	357402	0	0 pack-years	0 to 19 pack-years	0.609766	0.280058
lung_cancer	357402	0	0 pack-years	20 to 39.95 pack-years	1.205971	0.219968
lung_cancer	357404	0	0 pack-years	41 to 61.5 pack-years	2.928524	0.712103
lung_cancer	357404	0	0 pack-years	21 to 40 pack-years	2.721295	0.716635
lung_cancer	357404	0	0 pack-years	1 to 20 pack-years	1.064711	0.844476
lung_cancer	357406	0	0 pack-years	0 to 20 pack-years	0.300105	0.396825
lung_cancer	357406	0	0 pack-years	60 to 90 pack-years	1.280934	0.727041
lung_cancer	357406	0	0 pack-years	0 to 20 pack-years	1.373716	0.359726
lung_cancer	357406	0	0 pack-years	21 to 40 pack-years	1.435085	0.358965
lung_cancer	357406	0	0 pack-years	41 to 60 pack-years	1.931521	0.345682
lung_cancer	357406	0	0 pack-years	41 to 60 pack-years	2.000128	0.556806
lung_cancer	357406	0	0 pack-years	60 to 90 pack-years	2.046402	0.345409
lung_cancer	357406	0	0 pack-years	21 to 40 pack-years	2.195	0.537476
lung_cancer	357415	0	0 pack-years	33 to 49.5 pack-years	3.367296	0.139866
lung_cancer	357415	0	0 pack-years	33 to 49.5 pack-years	3.328627	0.179211
lung_cancer	357415	0	0 pack-years	19 to 32 pack-years	2.564949	0.125589
lung_cancer	357415	0	0 pack-years	19 to 32 pack-years	2.541602	0.178772
lung_cancer	357415	0	0 pack-years	0 to 8 pack-years	0.741937	0.218659
lung_cancer	357415	0	0 pack-years	9 to 18 pack-years	1.280934	0.198413
lung_cancer	357415	0	0 pack-years	0 to 8 pack-years	0.405465	0.153061
lung_cancer	357415	0	0 pack-years	9 to 18 pack-years	1.410987	0.143106
lung_cancer	357437	0	0 pack-years	90 to 100 pack-years	3.020425	1.029119
lung_cancer	357437	0	0 pack-years	0 to 44 pack-years	1.871802	0.255102
lung_cancer	357437	0	0 pack-years	45 to 89 pack-years	2.541602	0.253093
lung_cancer	357447	0	0 pack-years	20.25 to 40 pack-years	1.84055	0.465662
lung_cancer	357447	0	0 pack-years	40.25 to 60.375 pack-years	2.631889	0.405594

lung_cancer	357447	0	0 pack-years	0.25 to 20 pack-years	0.262364	0.804553
lung_cancer	357451	0	0 pack-years	1 to 32 pack-years	0.378436	0.232387
lung_cancer	357451	0	0 pack-years	33 to 53 pack-years	1.302913	0.193406
lung_cancer	357451	0	0 pack-years	54 to 81 pack-years	1.517323	0.189089
lung_cancer	357451	0	0 pack-years	82 to 100 pack-years	1.528228	0.188698
lung_cancer	357644	0	0 pack-years	30 to 39 pack-years	2.895359	0.217071
lung_cancer	357644	0	0 pack-years	40 to 60 pack-years	2.97604	0.201102
lung_cancer	357644	0	0 pack-years	20 to 29 pack-years	2.175887	0.161258
lung_cancer	357644	0	0 pack-years	0 to 20 pack-years	1.238374	0.119431
lung_cancer	357644	0	0 pack-years	31 to 39 pack-years	3.328985	0.114552
lung_cancer	357644	0	0 pack-years	21 to 30 pack-years	2.899772	0.113638
lung_cancer	357644	1	0 pack-years	0 to 20 pack-years	2.401525	0.111642
lung_cancer	357644	0	0 pack-years	40 to 60 pack-years	3.613078	0.108182
lung_cancer	357757	0	0 pack-years	60 to 90 pack-years	4.405499	0.753158
lung_cancer	357757	0	0 pack-years	0 to 29 pack-years	1.652497	0.446184
lung_cancer	357757	0	0 pack-years	0 to 29 pack-years	1.987874	0.310665
lung_cancer	357757	0	0 pack-years	30 to 59 pack-years	2.397895	0.392161
lung_cancer	357757	0	0 pack-years	60 to 90 pack-years	3.11795	0.465279
lung_cancer	357757	0	0 pack-years	30 to 59 pack-years	3.284664	0.34969
lung_cancer	357765	0	0 pack-years	51 to 60 pack-years	4.270676	0.517404
lung_cancer	357765	0	0 pack-years	41 to 50 pack-years	4.051785	0.469343
lung_cancer	357765	0	0 pack-years	61 to 91.5 pack-years	4.732684	0.602095
lung_cancer	357765	0	0 pack-years	31 to 40 pack-years	3.789177	0.334367
lung_cancer	357765	0	0 pack-years	26 to 30 pack-years	3.577669	0.423103
lung_cancer	357765	0	0 pack-years	21 to 25 pack-years	3.075929	0.375295
lung_cancer	357765	0	0 pack-years	16 to 20 pack-years	2.230014	0.35824
lung_cancer	357765	0	0 pack-years	11 to 15 pack-years	2.045109	0.372918
lung_cancer	357765	0	0 pack-years	6 to 10 pack-years	1.302913	0.393051
lung_cancer	357765	0	0 pack-years	1 to 5 pack-years	0.542324	0.456811
lung_cancer	357792	0	0 pack-years	1 to 9 pack-years	0.693147	0.204082
lung_cancer	357792	0	0 pack-years	10 to 19 pack-years	1.252763	0.182216
lung_cancer	357792	0	0 pack-years	20 to 29 pack-years	1.774952	0.177274
lung_cancer	357792	0	0 pack-years	30 to 39 pack-years	2.219203	0.169144
lung_cancer	357792	0	0 pack-years	40 to 60 pack-years	3.063391	0.158545
lung_cancer	357961	0	0 pack-years	40 to 60 pack-years	1.832581	0.65551
lung_cancer	357961	0	0 pack-years	20 to 39 pack-years	1.437463	0.66472
lung_cancer	357961	0	0 pack-years	1 to 19 pack-years	1.358409	0.667594
lung_cancer	357961	0	0 pack-years	40 to 60 pack-years	1.131402	0.27156
lung_cancer	357961	0	0 pack-years	1 to 19 pack-years	0.277632	0.351925
lung_cancer	357961	0	0 pack-years	20 to 39 pack-years	1.166271	0.208214
lung_cancer	358161	0	0 pack-years	1 to 20 pack-years	0.165514	0.462859
lung_cancer	358161	0	0 pack-years	21 to 31.5 pack-years	0.788457	0.396568
lung_cancer	358213	0	0 pack-years	1 to 39 pack-years	1.423108	8.003442

lung_cancer	358213	0	0 pack-years	40 to 60 pack-years	2.385086	3.058406
lung_cancer	358338	0	0 pack-years	1 to 20 pack-years	1.393766	0.208892
lung_cancer	358338	0	0 pack-years	20 to 50 pack-years	2.069391	0.190682
lung_cancer	358338	0	0 pack-years	51 to 76.5 pack-years	2.271094	0.192182
lung_cancer	358479	0	0 pack-years	40 to 60 pack-years	2.235269	2.001087
lung_cancer	358479	0	0 pack-years	1 to 40 pack-years	0.645007	1.290902
lung_cancer	358502	0	0 pack-years	0 to 19 pack-years	0.741937	0.327988
lung_cancer	358502	0	0 pack-years	20 to 39 pack-years	1.131402	0.213957
lung_cancer	358502	0	0 pack-years	40 to 59 pack-years	1.774952	0.203217
lung_cancer	358502	0	0 pack-years	60 to 90 pack-years	2.04122	0.228598
lung_cancer	358552	0	0 pack-years	20 to 38 pack-years	2.901422	0.220061
lung_cancer	358552	0	0 pack-years	40 to 60 pack-years	2.509599	0.29036
lung_cancer	358552	0	0 pack-years	40 to 60 pack-years	3.74242	0.213391
lung_cancer	358552	0	0 pack-years	1 to 18 pack-years	1.774952	0.250778
lung_cancer	358552	0	0 pack-years	1 to 18 pack-years	0.587787	0.255102
lung_cancer	358552	0	0 pack-years	20 to 38 pack-years	1.931521	0.232919
lung_cancer	358558	0	0 pack-years	0 to 34 pack-years	0.891998	0.531114
lung_cancer	358558	0	0 pack-years	35 to 52.5 pack-years	1.675226	0.471987
lung_cancer	358563	0	0 pack-years	0 to 18 pack-years	0.65752	0.611981
lung_cancer	358563	0	0 pack-years	20 to 38 pack-years	1.223775	0.427671
lung_cancer	358563	0	0 pack-years	61 to 91.5 pack-years	1.451614	0.538881
lung_cancer	358563	0	0 pack-years	40 to 58 pack-years	1.704748	0.434137
lung_cancer	358565	0	0 pack-years	20 to 28 pack-years	1.774952	0.242131
lung_cancer	358565	0	0 pack-years	0 to 9 pack-years	0.09531	0.20872
lung_cancer	358565	0	0 pack-years	10 to 18 pack-years	1.064711	0.246305
lung_cancer	358565	0	0 pack-years	30 to 45 pack-years	2.873565	0.227718
lung_cancer	358577	0	0 pack-years	50 to 58 pack-years	1.928619	0.217282
lung_cancer	358577	0	0 pack-years	61 to 91.5 pack-years	2.166765	0.187601
lung_cancer	358577	0	0 pack-years	40 to 48 pack-years	1.76815	0.181096
lung_cancer	358577	0	0 pack-years	1 to 18 pack-years	1.150572	0.219582
lung_cancer	358577	0	0 pack-years	20 to 28 pack-years	1.366092	0.192628
lung_cancer	358577	0	0 pack-years	30 to 38 pack-years	1.669592	0.175353
lung_cancer	358597	0	0 pack-years	0 to 20.3 pack-years	-1.27297	1.029519
lung_cancer	358597	0	0 pack-years	21.3 to 31.95 pack-years	2.489894	0.220835
lung_cancer	358717	0	0 pack-years	0 to 33 pack-years	1.860975	0.260656
lung_cancer	358717	0	0 pack-years	34 to 56 pack-years	2.424803	0.265487
lung_cancer	358717	0	0 pack-years	58 to 87 pack-years	3.044522	0.269679
lung_cancer	358794	1	0 pack-years	1 to 4 pack-years	1.345472	0.110943
lung_cancer	358794	1	0 pack-years	1 to 4 pack-years	1.745716	0.146917
lung_cancer	358794	1	0 pack-years	6 to 14 pack-years	2.539237	0.075906
lung_cancer	358794	1	0 pack-years	6 to 14 pack-years	2.605648	0.103058
lung_cancer	358794	1	0 pack-years	16 to 24 pack-years	3.173878	0.076744
lung_cancer	358794	1	0 pack-years	16 to 24 pack-years	3.304686	0.099924

lung_cancer	359451	1	0 pack-years	40 to 60 pack-years	1.247032	0.104093
lung_cancer	359451	1	0 pack-years	30 to 39 pack-years	1.033184	0.139807
lung_cancer	359451	0	0 pack-years	1 to 8 pack-years	0.231112	0.145773
lung_cancer	359451	0	0 pack-years	10 to 18 pack-years	0.636577	0.144423
lung_cancer	359451	0	0 pack-years	20 to 28 pack-years	0.955511	0.136381
lung_cancer	413177	0	0 pack-years	10.9 to 22.5 pack-years	1.808289	0.103435
lung_cancer	413177	0	0 pack-years	10.9 to 22.5 pack-years	1.722767	0.160774
lung_cancer	413177	0	0 pack-years	5.4 to 10.8 pack-years	1.098612	0.231048
lung_cancer	413177	0	0 pack-years	1 to 5.3 pack-years	0.693147	0.333758
lung_cancer	413177	1	0 pack-years	22.6 to 33.9 pack-years	0.693147	0.08797
lung_cancer	413177	1	0 pack-years	10.9 to 22.5 pack-years	0.470004	0.077903
lung_cancer	413177	0	0 pack-years	5.4 to 10.8 pack-years	0.262364	0.135364
lung_cancer	413177	0	0 pack-years	22.6 to 33.9 pack-years	2.517696	0.104276
lung_cancer	413177	0	0 pack-years	5.4 to 10.8 pack-years	1.223775	0.13994
lung_cancer	413177	0	0 pack-years	10.9 to 22.5 pack-years	3.353407	0.409372
lung_cancer	413177	0	0 pack-years	22.6 to 33.9 pack-years	2.424803	1.029894
lung_cancer	413177	0	0 pack-years	10.9 to 22.5 pack-years	2.370244	0.557347
lung_cancer	413177	0	0 pack-years	5.4 to 10.8 pack-years	2.00148	0.560516
lung_cancer	413177	0	0 pack-years	1 to 5.3 pack-years	2.151762	0.62701
lung_cancer	413177	0	0 pack-years	22.6 to 33.9 pack-years	0.182322	0.965354
lung_cancer	413177	0	0 pack-years	5.4 to 10.8 pack-years	0.741937	0.329333
lung_cancer	413177	0	0 pack-years	22.6 to 33.9 pack-years	2.60269	1.029143
lung_cancer	413177	0	0 pack-years	22.6 to 33.9 pack-years	2.406945	0.165411
lung_cancer	413177	0	0 pack-years	5.4 to 10.8 pack-years	3.321432	0.384998
lung_cancer	413177	0	0 pack-years	1 to 5.3 pack-years	0.955511	0.176823
lung_cancer	419667	0	0 pack-years	51 to 76.5 pack-years	3.218876	0.834694
lung_cancer	419667	0	0 pack-years	30 to 50 pack-years	2.639057	0.881924
lung_cancer	419667	0	0 pack-years	1 to 30 pack-years	1.916923	0.829082
lung_cancer	419669	0	0 pack-years	1 to 33 pack-years	0.641854	0.37594
lung_cancer	419669	0	0 pack-years	34 to 54 pack-years	1.871802	0.33752
lung_cancer	419669	0	0 pack-years	55 to 84 pack-years	2.674149	0.343068
lung_cancer	419669	0	0 pack-years	85 to 100 pack-years	2.778819	0.345418
lung_cancer	419671	0	0 pack-years	20 to 39 pack-years	-0.91629	2.933673
lung_cancer	419671	0	0 pack-years	1 to 19 pack-years	0.641854	0.308808
lung_cancer	419671	0	0 pack-years	20 to 39 pack-years	0.693147	0.369898
lung_cancer	419671	0	0 pack-years	1 to 19 pack-years	0.916291	0.520408
lung_cancer	419671	0	0 pack-years	40 to 60 pack-years	1.193922	0.494743
lung_cancer	419675	0	0 pack-years	40 to 60 pack-years	3.981549	0.238444
lung_cancer	419675	0	0 pack-years	20 to 39 pack-years	3.449988	0.95724
lung_cancer	419675	0	0 pack-years	20 to 39 pack-years	3.404525	0.231372
lung_cancer	419675	0	0 pack-years	20 to 39 pack-years	3.317816	0.561964
lung_cancer	419675	0	0 pack-years	40 to 60 pack-years	2.476538	0.437318
lung_cancer	419675	0	0 pack-years	40 to 60 pack-years	3.511545	0.794243

lung_cancer	419675	0	0 pack-years	0 to 19 pack-years	2.140066	0.231092
lung_cancer	419675	0	0 pack-years	0 to 19 pack-years	1.974081	0.60941
lung_cancer	419675	0	0 pack-years	0 to 19 pack-years	1.648659	0.539639
lung_cancer	419675	0	0 pack-years	20 to 39 pack-years	2.282382	0.304561
lung_cancer	419675	0	0 pack-years	0 to 19 pack-years	0.530628	0.22509
lung_cancer	419696	0	0 pack-years	1 to 29 pack-years	1.193922	0.448361
lung_cancer	419696	0	0 pack-years	30 to 44 pack-years	2.476538	0.454468
lung_cancer	419696	0	0 pack-years	45 to 67.5 pack-years	3.693867	0.467052
lung_cancer	419717	0	0 pack-years	21 to 30 pack-years	2.318458	0.274938
lung_cancer	419717	0	0 pack-years	31 to 46.5 pack-years	3.165053	0.258333
lung_cancer	419717	0	0 pack-years	1 to 10 pack-years	1.311032	0.251664
lung_cancer	419717	0	0 pack-years	11 to 20 pack-years	1.948763	0.263097
lung_cancer	419728	0	0 pack-years	1 to 19 pack-years	1.105257	0.268617
lung_cancer	419728	0	0 pack-years	20 to 29 pack-years	2.543176	0.350164
lung_cancer	419728	0	0 pack-years	30 to 45 pack-years	3.255015	0.314153
lung_cancer	426275	0	0 pack-years	80 to 100 pack-years	3.238678	0.113045
lung_cancer	426275	0	0 pack-years	80 to 100 pack-years	3.86073	0.102041
lung_cancer	426275	0	0 pack-years	60 to 79 pack-years	3.763523	0.09411
lung_cancer	426275	0	0 pack-years	60 to 79 pack-years	3.678829	0.108225
lung_cancer	426275	0	0 pack-years	80 to 100 pack-years	3.591818	0.103306
lung_cancer	426275	0	0 pack-years	40 to 59 pack-years	3.401197	0.072279
lung_cancer	426275	0	0 pack-years	60 to 79 pack-years	3.397858	0.104088
lung_cancer	426275	0	0 pack-years	40 to 59 pack-years	3.367296	0.097643
lung_cancer	426275	0	0 pack-years	40 to 59 pack-years	3.202746	0.099552
lung_cancer	426275	0	0 pack-years	80 to 100 pack-years	3.903991	0.104407
lung_cancer	426275	0	0 pack-years	60 to 79 pack-years	3.144152	0.100062
lung_cancer	426275	0	0 pack-years	0 to 19 pack-years	1.601406	0.115207
lung_cancer	426275	0	0 pack-years	20 to 39 pack-years	2.360854	0.082307
lung_cancer	426275	1	0 pack-years	0 to 19 pack-years	2.509599	0.129003
lung_cancer	426275	1	0 pack-years	0 to 19 pack-years	2.70805	0.078231
lung_cancer	426275	0	0 pack-years	20 to 39 pack-years	3.186353	0.112793
lung_cancer	426275	1	0 pack-years	0 to 19 pack-years	2.833213	0.117047
lung_cancer	426275	0	0 pack-years	40 to 59 pack-years	2.879198	0.078824
lung_cancer	426275	1	0 pack-years	20 to 39 pack-years	3.095578	0.07503
lung_cancer	426275	0	0 pack-years	20 to 39 pack-years	2.76001	0.109791
lung_cancer	450624	0	0 pack-years	15 to 30 pack-years	1.948763	0.176309
lung_cancer	450624	0	0 pack-years	1 to 15 pack-years	0.989541	0.171452
lung_cancer	450624	0	0 pack-years	31 to 46.5 pack-years	2.520113	0.186111
lung_cancer	450624	0	0 pack-years	15 to 30 pack-years	1.451614	0.258872
lung_cancer	450624	0	0 pack-years	1 to 15 pack-years	0.727549	0.244671
lung_cancer	450624	0	0 pack-years	31 to 46.5 pack-years	1.809927	0.278087
lung_cancer	499241	0	0 pack-years	1 to 5 pack-years	1.337629	0.169717
lung_cancer	499241	0	0 pack-years	6 to 15 pack-years	2.066863	0.16266

lung_cancer	499241	0	0 pack-years	16 to 22.5 pack-years	3.055886	0.160008
lung_cancer	502075	0	0 pack-years	0.1 to 9.9 pack-years	0.703098	0.133908
lung_cancer	502075	0	0 pack-years	10 to 19.9 pack-years	1.363537	0.099174
lung_cancer	502075	0	0 pack-years	20 to 29.9 pack-years	1.976855	0.089892
lung_cancer	502075	0	0 pack-years	30 to 39.9 pack-years	2.36368	0.088635
lung_cancer	502075	0	0 pack-years	40 to 60 pack-years	2.899221	0.079635
lung_cancer	502124	0	0 pack-years	26 to 35 pack-years	3.378611	0.175655
lung_cancer	502124	1	0 pack-years	16 to 25 pack-years	3.16167	0.115918
lung_cancer	502124	0	0 pack-years	35 to 52.5 pack-years	3.653511	0.208709
lung_cancer	502124	0	0 pack-years	1 to 5 pack-years	2.221375	0.298299
lung_cancer	502124	1	0 pack-years	6 to 15 pack-years	2.668616	0.123646
lung_cancer	502126	1	0 pack-years	1 to 29 pack-years	0.601361	0.103087
lung_cancer	502126	1	0 pack-years	30 to 49 pack-years	1.067259	0.127873
lung_cancer	502126	1	0 pack-years	50 to 75 pack-years	1.256869	0.163028
lung_cancer	502130	0	0 pack-years	0 to 10 pack-years	0.157004	0.309121
lung_cancer	502130	0	0 pack-years	10 to 19 pack-years	1.316408	0.14444
lung_cancer	502130	0	0 pack-years	20 to 29 pack-years	1.774952	0.127076
lung_cancer	502130	0	0 pack-years	30 to 45 pack-years	2.487404	0.094866
lung_cancer	502143	0	0 pack-years	2.5 to 11 pack-years	3.76584	1.017146
lung_cancer	502143	0	0 pack-years	12 to 18 pack-years	4.232607	1.024342
lung_cancer	502147	1	0 pack-years	40 to 60 pack-years	0.900161	0.078195
lung_cancer	502147	1	0 pack-years	1 to 39 pack-years	0.641854	0.091949
lung_cancer	502184	0	0 pack-years	1 to 9 pack-years	-0.44629	0.64545
lung_cancer	502184	0	0 pack-years	10 to 19 pack-years	2.009555	0.370232
lung_cancer	502184	0	0 pack-years	20 to 30 pack-years	2.998229	0.30923
lung_cancer	502184	0	0 pack-years	1 to 9 pack-years	0.559616	0.608183
lung_cancer	502184	0	0 pack-years	10 to 19 pack-years	0.277632	0.707293
lung_cancer	502184	0	0 pack-years	20 to 30 pack-years	2.318458	0.439651
lung_cancer	502190	0	0 pack-years	0 to 30 pack-years	1.249902	0.444242
lung_cancer	502190	0	0 pack-years	0 to 30 pack-years	1.181727	0.340722
lung_cancer	502190	0	0 pack-years	30 to 45 pack-years	1.742219	0.278195
lung_cancer	502190	0	0 pack-years	30 to 45 pack-years	2.346602	0.509685
lung_cancer	502192	0	0 pack-years	20 to 30 pack-years	2.285439	0.151108
lung_cancer	502192	1	0 pack-years	0 to 20 pack-years	3.030134	0.12301
lung_cancer	502202	0	0 pack-years	0 to 9 pack-years	-0.21072	0.187965
lung_cancer	502202	0	0 pack-years	10 to 19 pack-years	0.875469	0.18883
lung_cancer	502202	0	0 pack-years	20 to 29 pack-years	1.474763	0.19744
lung_cancer	502202	0	0 pack-years	30 to 45 pack-years	2.214846	0.160665
lung_cancer	502208	0	0 pack-years	1 to 9 pack-years	0.076035	0.239733
lung_cancer	502208	0	0 pack-years	10 to 20 pack-years	0.111541	0.245367
lung_cancer	502208	1	0 pack-years	21 to 30 pack-years	0.497132	0.182418
lung_cancer	502208	1	0 pack-years	31 to 40 pack-years	0.214305	0.278356
lung_cancer	502208	0	0 pack-years	41 to 50 pack-years	0.798858	0.27012

lung_cancer	502208	0	0 pack-years	51 to 60 pack-years	0.383219	0.357386
lung_cancer	502208	0	0 pack-years	61 to 91.5 pack-years	1.299374	0.304469
lung_cancer	502213	0	0 pack-years	41 to 61.5 pack-years	2.619554	0.127073
lung_cancer	502213	0	0 pack-years	20 to 40 pack-years	1.980049	0.134067
lung_cancer	502213	0	0 pack-years	1 to 19 pack-years	1.019317	0.151592
lung_cancer	502215	0	0 pack-years	1 to 39.9 pack-years	0.542324	0.189372
lung_cancer	502215	1	0 pack-years	40 to 60 pack-years	0.920283	0.198394
lung_cancer	502234	0	0 pack-years	1 to 19 pack-years	0.587787	0.404112
lung_cancer	502234	0	0 pack-years	20 to 39 pack-years	2.140066	0.41191
lung_cancer	502234	0	0 pack-years	40 to 60 pack-years	3.218876	0.437929
lung_cancer	502234	0	0 pack-years	1 to 19 pack-years	0.09531	0.831147
lung_cancer	502234	0	0 pack-years	20 to 39 pack-years	3.346389	1.49775
lung_cancer	502234	0	0 pack-years	40 to 60 pack-years	2.054124	1.505076
lung_cancer	502241	0	0 pack-years	49.5 to 74.25 pack-years	0.604316	0.668348
lung_cancer	502241	0	0 pack-years	49.6 to 74.4 pack-years	0.565314	0.574545
lung_cancer	502246	0	0 pack-years	40 to 60 pack-years	2.13771	0.275743
lung_cancer	502246	0	0 pack-years	1 to 35 pack-years	1.238374	0.283247
lung_cancer	502252	0	0 pack-years	1 to 39.9 pack-years	1.413423	0.289575
lung_cancer	502252	0	0 pack-years	40 to 60 pack-years	1.728109	0.272142
lung_cancer	502407	0	0 pack-years	1 to 20 pack-years	0.675645	0.1948
lung_cancer	502407	0	0 pack-years	21 to 40 pack-years	0.957433	0.178998
lung_cancer	502407	1	0 pack-years	41 to 60 pack-years	1.24973	0.171421
lung_cancer	502407	1	0 pack-years	61 to 91.5 pack-years	1.374551	0.166037
lung_cancer	502407	0	0 pack-years	1 to 5 pack-years	0.22594	0.100394
lung_cancer	502407	0	0 pack-years	5 to 19 pack-years	1.19189	0.073833
lung_cancer	502407	0	0 pack-years	20 to 30 pack-years	2.389139	0.056879
lung_cancer	502421	0	0 pack-years	1 to 19 pack-years	0.972179	0.061423
lung_cancer	502421	0	0 pack-years	1 to 19 pack-years	1.099545	0.06189
lung_cancer	502421	0	0 pack-years	1 to 19 pack-years	0.465682	0.073277
lung_cancer	502433	0	0 pack-years	1 to 19 pack-years	1.068497	0.275705
lung_cancer	502433	0	0 pack-years	20 to 30 pack-years	1.268917	0.323224
lung_cancer	502458	0	0 pack-years	0.05 to 25 pack-years	1.686399	0.353647
lung_cancer	502458	0	0 pack-years	25.05 to 50 pack-years	2.549445	0.336667
lung_cancer	502458	0	0 pack-years	50.05 to 75 pack-years	3.135494	0.34147
lung_cancer	502458	0	0 pack-years	75.05 to 112.5 pack-years	3.74242	0.346392
lung_cancer	502460	0	0 pack-years	1 to 19.9 pack-years	-0.09431	0.371826
lung_cancer	502460	0	0 pack-years	20 to 39 pack-years	1.223775	0.331843
lung_cancer	502460	0	0 pack-years	40 to 60 pack-years	1.560248	0.347486
lung_cancer	502482	1	0 pack-years	1 to 25 pack-years	0.245922	0.11096
lung_cancer	502482	1	0 pack-years	1 to 25 pack-years	0.435089	0.109771
macular_degeneration	261355	0	0 cigarettes per day	0 to 19 cigarettes per day	0.231112	0.400875
macular_degeneration	261355	0	0 cigarettes per day	20 to 30 cigarettes per day	0.900161	0.227103
macular_degeneration	359130	0	0 cigarettes per day	15 to 24 cigarettes per day	0.336472	0.291545

macular_degeneration	359130	0	0 cigarettes per day	1 to 14 cigarettes per day	0.470004	0.302934
macular_degeneration	359130	0	0 cigarettes per day	25 to 37.5 cigarettes per day	0.875469	0.276361
multiple_sclerosis	221241	0	0 cigarettes per day	20 to 80 cigarettes per day	0.802002	0.551596
multiple_sclerosis	221241	0	0 cigarettes per day	20 to 80 cigarettes per day	1.20896	0.47863
multiple_sclerosis	348023	0	0 cigarettes per day	16 to 24 cigarettes per day	0.693147	0.063776
multiple_sclerosis	348023	1	0 cigarettes per day	0 to 4 cigarettes per day	0.693147	0.204082
multiple_sclerosis	348023	0	0 cigarettes per day	16 to 24 cigarettes per day	0.587787	0.113379
multiple_sclerosis	348023	0	0 cigarettes per day	11 to 16.5 cigarettes per day	0.530628	0.07503
multiple_sclerosis	348023	0	0 cigarettes per day	5 to 10 cigarettes per day	0.530628	0.15006
multiple_sclerosis	348023	0	0 cigarettes per day	0 to 4 cigarettes per day	0.530628	0.255102
multiple_sclerosis	348023	0	0 cigarettes per day	16 to 24 cigarettes per day	0.405465	0.119048
multiple_sclerosis	348023	1	0 cigarettes per day	5 to 10 cigarettes per day	0.641854	0.120838
multiple_sclerosis	348023	0	0 cigarettes per day	11 to 16.5 cigarettes per day	0.336472	0.127551
multiple_sclerosis	348023	0	0 cigarettes per day	11 to 16.5 cigarettes per day	0.182322	0.127551
multiple_sclerosis	348023	0	0 cigarettes per day	5 to 10 cigarettes per day	0.09531	0.139147
multiple_sclerosis	348023	0	0 cigarettes per day	0 to 4 cigarettes per day	0.09531	0.185529
multiple_sclerosis	348023	0	0 cigarettes per day	0 to 4 cigarettes per day	0	0.178571
multiple_sclerosis	348023	0	0 cigarettes per day	5 to 10 cigarettes per day	0	0.127551
multiple_sclerosis	348023	0	0 cigarettes per day	16 to 24 cigarettes per day	0.405465	0.102041
multiple_sclerosis	350715	0	0 cigarettes per day	1 to 14 cigarettes per day	0.182322	0.212585
multiple_sclerosis	350715	0	0 cigarettes per day	16 to 24 cigarettes per day	0.336472	0.23688
multiple_sclerosis	350717	0	0 cigarettes per day	15 to 22.5 cigarettes per day	0.587787	0.396825
multiple_sclerosis	350717	0	0 cigarettes per day	1 to 14 cigarettes per day	0.470004	0.366709
multiple_sclerosis	369019	0	0 cigarettes per day	0 to 9 cigarettes per day	-0.35667	0.437318
multiple_sclerosis	369019	0	0 cigarettes per day	10 to 20 cigarettes per day	0.336472	0.291545
multiple_sclerosis	369019	0	0 cigarettes per day	20 to 40 cigarettes per day	0.641854	0.268528
multiple_sclerosis	369019	0	0 cigarettes per day	40 to 60 cigarettes per day	1.704748	0.746753
multiple_sclerosis	369022	0	0 cigarettes per day	14 to 21 cigarettes per day	-0.06188	0.645897
multiple_sclerosis	369022	0	0 cigarettes per day	0 to 13 cigarettes per day	-0.05129	0.725027
multiple_sclerosis	369022	0	0 cigarettes per day	0 to 13 cigarettes per day	0.019803	0.752801
multiple_sclerosis	369022	0	0 cigarettes per day	14 to 21 cigarettes per day	0.518794	0.580053
nasopharyngeal_cancer	328266	0	0 pack-years	41 to 61.5 pack-years	1.029619	1.685496
nasopharyngeal_cancer	328266	0	0 pack-years	0 to 20 pack-years	1.163151	1.100128
nasopharyngeal_cancer	328266	0	0 pack-years	20 to 40 pack-years	1.360977	1.03349
nasopharyngeal_cancer	328921	0	0 pack-years	0 to 9 pack-years	0.565314	0.933442
nasopharyngeal_cancer	328921	1	0 pack-years	10 to 15 pack-years	1.393766	0.714666
nasopharyngeal_cancer	328952	0	0 pack-years	0 to 30 pack-years	-0.16252	0.464476
nasopharyngeal_cancer	328952	0	0 pack-years	30 to 45 pack-years	0.756122	0.439987
nasopharyngeal_cancer	346030	0	0 pack-years	60 to 90 pack-years	1.589235	1.025614
nasopharyngeal_cancer	346030	0	0 pack-years	31 to 59 pack-years	1.252763	0.575802
nasopharyngeal_cancer	346030	0	0 pack-years	60 to 90 pack-years	1.131402	0.370309
nasopharyngeal_cancer	346030	0	0 pack-years	0 to 30 pack-years	0.262364	0.431711
nasopharyngeal_cancer	346030	0	0 pack-years	0 to 30 pack-years	-0.10536	0.340136

nasopharyngeal_cancer	346030	0	0 pack-years	31 to 59 pack-years	0.587787	0.354308
nasopharyngeal_cancer	346032	0	0 pack-years	60 to 90 pack-years	1.458615	0.646654
nasopharyngeal_cancer	346032	0	0 pack-years	1 to 4 pack-years	0.641854	0.416219
nasopharyngeal_cancer	346032	0	0 pack-years	35 to 59 pack-years	1.098612	0.467687
nasopharyngeal_cancer	346040	0	0 pack-years	30 to 44 pack-years	0.916291	0.377551
nasopharyngeal_cancer	346040	0	0 pack-years	45 to 67.5 pack-years	1.360977	0.379383
nasopharyngeal_cancer	346040	0	0 pack-years	0 to 14 pack-years	0.262364	0.353218
nasopharyngeal_cancer	346040	0	0 pack-years	15 to 29 pack-years	0.587787	0.354308
nasopharyngeal_cancer	346047	0	0 pack-years	0 to 19 pack-years	0.262364	0.235479
nasopharyngeal_cancer	346047	0	0 pack-years	20 to 30 pack-years	0.405465	0.255102
nasopharyngeal_cancer	346049	0	0 pack-years	20 to 39 pack-years	-0.07257	1.091727
nasopharyngeal_cancer	346049	0	0 pack-years	0 to 19 pack-years	0.182322	0.142432
nasopharyngeal_cancer	346049	0	0 pack-years	20 to 39 pack-years	0.277632	0.16427
nasopharyngeal_cancer	346049	0	0 pack-years	0 to 19 pack-years	0.29267	0.394076
nasopharyngeal_cancer	346049	0	0 pack-years	40 to 60 pack-years	0.48858	0.241017
nasopharyngeal_cancer	346049	1	0 pack-years	40 to 60 pack-years	0.770108	4.494992
nasopharyngeal_cancer	346056	1	0 pack-years	30 to 45 pack-years	2.667228	0.246776
nasopharyngeal_cancer	346056	0	0 pack-years	0 to 14 pack-years	0.262364	0.133438
nasopharyngeal_cancer	346056	1	0 pack-years	15 to 30 pack-years	2.38968	0.234759
nasopharyngeal_cancer	346071	0	0 pack-years	1 to 13 pack-years	0.494696	0.195993
nasopharyngeal_cancer	346071	0	0 pack-years	14 to 21 pack-years	0.57098	0.196011
nasopharyngeal_cancer	373803	0	0 pack-years	10 to 19 pack-years	0.122218	0.112877
nasopharyngeal_cancer	373803	0	0 pack-years	0 to 9 pack-years	0.165514	0.099447
nasopharyngeal_cancer	373803	0	0 pack-years	20 to 29 pack-years	0.207014	0.109922
nasopharyngeal_cancer	373803	0	0 pack-years	30 to 45 pack-years	0.438255	0.105332
nasopharyngeal_cancer	502223	0	0 pack-years	16 to 25 pack-years	0.223144	0.136612
nasopharyngeal_cancer	502223	0	0 pack-years	0 to 15 pack-years	0.00995	0.139424
nasopharyngeal_cancer	502223	0	0 pack-years	25 to 37.5 pack-years	0.683097	0.116035
other_pharynx_cancer	343375	0	0 pack-years	1 to 20 pack-years	1.686399	0.535456
other_pharynx_cancer	343375	0	0 pack-years	21 to 40 pack-years	1.740466	0.557515
other_pharynx_cancer	343375	0	0 pack-years	41 to 61.5 pack-years	2.014903	0.583107
other_pharynx_cancer	343434	0	0 pack-years	51 to 76.5 pack-years	1.029619	0.637755
other_pharynx_cancer	343434	0	0 pack-years	1 to 25 pack-years	0.641854	0.980129
other_pharynx_cancer	343434	0	0 pack-years	25 to 50 pack-years	1.410987	1.636386
other_pharynx_cancer	343434	0	0 pack-years	1 to 25 pack-years	0	0.688776
other_pharynx_cancer	343434	1	0 pack-years	51 to 76.5 pack-years	-0.10536	6.972789
other_pharynx_cancer	343434	0	0 pack-years	25 to 50 pack-years	0.336472	0.69242
other_pharynx_cancer	343450	0	0 pack-years	1 to 28 pack-years	1.252763	0.386297
other_pharynx_cancer	343450	0	0 pack-years	48 to 76 pack-years	1.902108	0.373134
other_pharynx_cancer	343450	1	0 pack-years	29 to 47 pack-years	2.186051	0.375487
other_pharynx_cancer	343450	1	0 pack-years	77 to 100 pack-years	2.587764	0.374022
other_pharynx_cancer	359128	0	0 pack-years	0 to 19 pack-years	2.491551	2.480487
other_pharynx_cancer	359128	0	0 pack-years	60 to 90 pack-years	3.040706	2.302625

other_pharynx_cancer	359128	0	0 pack-years	40 to 59 pack-years	2.695303	2.130731
other_pharynx_cancer	359128	0	0 pack-years	20 to 39 pack-years	1.86408	2.202183
other_pharynx_cancer	359128	0	0 pack-years	60 to 90 pack-years	1.373716	1.11147
other_pharynx_cancer	359128	0	0 pack-years	20 to 39 pack-years	1.350667	0.7012
other_pharynx_cancer	359128	0	0 pack-years	40 to 59 pack-years	0.672944	0.978759
other_pharynx_cancer	359128	0	0 pack-years	0 to 19 pack-years	-0.24846	2.282836
other_pharynx_cancer	364113	0	0 pack-years	1 to 29 pack-years	0.908259	0.102864
other_pharynx_cancer	364113	0	0 pack-years	30 to 39 pack-years	1.20896	0.144685
other_pharynx_cancer	364113	0	0 pack-years	40 to 49 pack-years	1.366092	0.175708
other_pharynx_cancer	364113	0	0 pack-years	60 to 90 pack-years	1.408545	0.243251
other_pharynx_cancer	364113	0	0 pack-years	50 to 59 pack-years	1.432701	0.213092
other_pharynx_cancer	439809	0	0 pack-years	1 to 19 pack-years	1.269761	0.33968
other_pharynx_cancer	439809	0	0 pack-years	20 to 39 pack-years	1.439835	0.302272
other_pharynx_cancer	439809	0	0 pack-years	40 to 60 pack-years	1.583094	0.298715
other_pharynx_cancer	439813	0	0 pack-years	0.05 to 40 pack-years	0.707543	0.489473
other_pharynx_cancer	439813	0	0 pack-years	40 to 60 pack-years	1.934849	0.526249
other_pharynx_cancer	502194	0	0 pack-years	1 to 10 pack-years	0.019803	0.832126
other_pharynx_cancer	502194	0	0 pack-years	10 to 19 pack-years	0.314811	0.350858
other_pharynx_cancer	502194	1	0 pack-years	20 to 30 pack-years	0.524729	0.251447
pancreatic_cancer	164536	0	0 pack-years	20 to 39 pack-years	0.425268	0.590236
pancreatic_cancer	164536	0	0 pack-years	0 to 19 pack-years	0.832909	0.50244
pancreatic_cancer	164536	0	0 pack-years	0 to 19 pack-years	0.97456	0.470735
pancreatic_cancer	164536	1	0 pack-years	40 to 60 pack-years	1.398717	0.456664
pancreatic_cancer	164536	1	0 pack-years	20 to 39 pack-years	1.570697	0.419513
pancreatic_cancer	164536	1	0 pack-years	40 to 60 pack-years	1.931521	0.85108
pancreatic_cancer	164547	0	0 pack-years	0 to 29 pack-years	0.405465	0.323129
pancreatic_cancer	343580	0	0 pack-years	50.05 to 75 pack-years	0.587787	0.390683
pancreatic_cancer	343580	0	0 pack-years	75.05 to 112.5 pack-years	0.641854	0.386823
pancreatic_cancer	343580	0	0 pack-years	0.05 to 25 pack-years	0.182322	0.420576
pancreatic_cancer	343580	0	0 pack-years	25.05 to 50 pack-years	0.530628	0.34646
pancreatic_cancer	346137	1	0 pack-years	40 to 60 pack-years	-0.56212	0.313283
pancreatic_cancer	346137	0	0 pack-years	0 to 19 pack-years	0	0.336735
pancreatic_cancer	346137	0	0 pack-years	20 to 39 pack-years	0.139762	0.301686
pancreatic_cancer	346137	0	0 pack-years	0 to 19 pack-years	0.357674	0.695733
pancreatic_cancer	346137	1	0 pack-years	40 to 60 pack-years	0.444686	1.439037
pancreatic_cancer	346137	0	0 pack-years	20 to 39 pack-years	0.875469	0.687713
pancreatic_cancer	346826	0	0 pack-years	11 to 25 pack-years	0.993252	0.349584
pancreatic_cancer	346826	0	0 pack-years	1 to 10 pack-years	0.262364	1.000785
pancreatic_cancer	346826	0	0 pack-years	51 to 76.5 pack-years	0.741937	0.315841
pancreatic_cancer	346826	1	0 pack-years	26 to 50 pack-years	1.029619	0.23688
pancreatic_cancer	346828	0	0 pack-years	20 to 30 pack-years	0.652325	0.289647
pancreatic_cancer	346828	0	0 pack-years	0 to 19 pack-years	0.131028	0.429646
pancreatic_cancer	346832	1	0 pack-years	1 to 8.5 pack-years	-1.60944	6.505102

pancreatic_cancer	346832	0	0 pack-years	1 to 14 pack-years	0.09531	0.672542
pancreatic_cancer	346832	0	0 pack-years	15 to 22.5 pack-years	0.832909	0.343833
pancreatic_cancer	346832	0	0 pack-years	9.5 to 14.25 pack-years	1.029619	0.446429
pancreatic_cancer	346870	1	0 pack-years	0 to 14 pack-years	-0.22314	0.28699
pancreatic_cancer	346870	0	0 pack-years	0 to 9 pack-years	0	0.382653
pancreatic_cancer	346870	0	0 pack-years	15 to 34 pack-years	0.405465	0.204082
pancreatic_cancer	346870	0	0 pack-years	10 to 15 pack-years	0.693147	0.357143
pancreatic_cancer	346870	0	0 pack-years	35 to 52.5 pack-years	0.875469	0.212585
pancreatic_cancer	346876	0	0 pack-years	60 to 90 pack-years	0.530628	0.495198
pancreatic_cancer	346876	0	0 pack-years	0 to 19 pack-years	0.693147	0.447704
pancreatic_cancer	346876	0	0 pack-years	40 to 59 pack-years	0.336472	0.340743
pancreatic_cancer	346876	0	0 pack-years	20 to 39 pack-years	0.530628	0.322629
pancreatic_cancer	347176	0	0 pack-years	0 to 19 pack-years	0.262364	0.156986
pancreatic_cancer	347176	0	0 pack-years	20 to 44 pack-years	0.641854	0.161117
pancreatic_cancer	347176	0	0 pack-years	45 to 67.5 pack-years	0.788457	0.173933
pancreatic_cancer	359135	0	0 pack-years	20 to 39 pack-years	0.615186	0.390237
pancreatic_cancer	359135	0	0 pack-years	0 to 19 pack-years	0.131028	0.304332
pancreatic_cancer	359135	0	0 pack-years	20 to 39 pack-years	0.398776	0.186618
pancreatic_cancer	359135	0	0 pack-years	40 to 60 pack-years	0.582216	0.171018
pancreatic_cancer	359135	0	0 pack-years	0 to 19 pack-years	0.783902	0.26908
pancreatic_cancer	359138	0	0 pack-years	15 to 24 pack-years	0.553885	0.250704
pancreatic_cancer	359139	0	0 pack-years	25 to 49 pack-years	0.891998	0.270785
pancreatic_cancer	359139	0	0 pack-years	50 to 75 pack-years	0.955511	0.275706
pancreatic_cancer	359139	0	0 pack-years	0 to 24 pack-years	0.553885	0.324009
pancreatic_cancer	359140	0	0 pack-years	10 to 19 pack-years	0.300105	0.472411
pancreatic_cancer	502128	0	0 pack-years	1 to 49.5 pack-years	0.86289	0.435114
pancreatic_cancer	502128	1	0 pack-years	49.51 to 74.25 pack-years	-0.12783	0.534552
pancreatic_cancer	502130	0	0 pack-years	0 to 10 pack-years	0.329304	0.343569
pancreatic_cancer	502130	0	0 pack-years	10 to 19 pack-years	0.405465	0.250904
pancreatic_cancer	502130	0	0 pack-years	20 to 29 pack-years	0.444686	0.257568
pancreatic_cancer	502130	0	0 pack-years	30 to 45 pack-years	0.65752	0.205769
pancreatic_cancer	502188	0	0 pack-years	14 to 32 pack-years	0.451076	0.108079
pancreatic_cancer	502188	1	0 pack-years	1 to 14 pack-years	-0.10536	0.11498
pancreatic_cancer	502188	0	0 pack-years	32 to 48 pack-years	0.405465	0.106925
pancreatic_cancer	502220	0	0 pack-years	0 to 20 pack-years	0.357674	0.095451
pancreatic_cancer	502220	0	0 pack-years	20 to 30 pack-years	0.565314	0.095148
pancreatic_cancer	502474	0	0 pack-years	41 to 61.5 pack-years	0.837248	0.457081
pancreatic_cancer	502474	0	0 pack-years	21 to 40 pack-years	0.955511	0.200474
pancreatic_cancer	502474	0	0 pack-years	21 to 40 pack-years	0.887891	0.245708
pancreatic_cancer	502474	0	0 pack-years	41 to 61.5 pack-years	0.904218	0.451178
pancreatic_cancer	502474	0	0 pack-years	1 to 20 pack-years	0.48858	0.173503
pancreatic_cancer	502474	0	0 pack-years	1 to 20 pack-years	0.512824	0.15406
pancreatic_cancer	502474	0	0 pack-years	21 to 40 pack-years	0.858662	0.227938

pancreatic_cancer	502474	0	0 pack-years	41 to 61.5 pack-years	1.043804	0.457081
pancreatic_cancer	502474	0	0 pack-years	1 to 20 pack-years	0.620576	0.156482
pancreatic_cancer	502474	0	0 pack-years	21 to 40 pack-years	0.932164	0.213736
pancreatic_cancer	502474	0	0 pack-years	41 to 61.5 pack-years	1.095273	0.452548
pancreatic_cancer	502474	0	0 pack-years	1 to 20 pack-years	0.512824	0.163849
pancreatic_cancer	502474	0	0 pack-years	21 to 40 pack-years	0.908259	0.221146
pancreatic_cancer	502474	0	0 pack-years	21 to 40 pack-years	0.947789	0.193557
pancreatic_cancer	502474	0	0 pack-years	1 to 20 pack-years	0.457425	0.185935
pancreatic_cancer	502474	0	0 pack-years	41 to 61.5 pack-years	0.828552	0.45481
pancreatic_cancer	502474	0	0 pack-years	1 to 20 pack-years	0.620576	0.144833
pancreatic_cancer	502474	0	0 pack-years	41 to 61.5 pack-years	0.587787	0.116758
pancreatic_cancer	502474	0	0 pack-years	21 to 40 pack-years	0.500775	0.12234
pancreatic_cancer	502474	0	0 pack-years	1 to 20 pack-years	0.350657	0.138677
pancreatic_cancer	502474	0	0 pack-years	21 to 40 pack-years	0.463734	0.103435
pancreatic_cancer	502474	0	0 pack-years	41 to 61.5 pack-years	0.609766	0.107399
pancreatic_cancer	502474	0	0 pack-years	1 to 20 pack-years	0.329304	0.139298
pancreatic_cancer	502474	0	0 pack-years	21 to 40 pack-years	0.444686	0.104104
pancreatic_cancer	502474	0	0 pack-years	41 to 61.5 pack-years	0.587787	0.109194
pancreatic_cancer	502474	0	0 pack-years	1 to 20 pack-years	0.336472	0.153733
pancreatic_cancer	502474	0	0 pack-years	21 to 40 pack-years	0.48858	0.11299
pancreatic_cancer	502474	0	0 pack-years	41 to 61.5 pack-years	0.615186	0.119682
pancreatic_cancer	502474	0	0 pack-years	1 to 20 pack-years	0.307485	0.151624
pancreatic_cancer	502474	0	0 pack-years	21 to 40 pack-years	0.470004	0.110585
pancreatic_cancer	502474	0	0 pack-years	1 to 20 pack-years	0.300105	0.154162
pancreatic_cancer	502474	0	0 pack-years	21 to 40 pack-years	0.451076	0.112712
pancreatic_cancer	502474	0	0 pack-years	41 to 61.5 pack-years	0.582216	0.120351
pancreatic_cancer	502474	0	0 pack-years	1 to 20 pack-years	0.300105	0.166081
pancreatic_cancer	502474	0	0 pack-years	41 to 61.5 pack-years	0.615186	0.153439
pancreatic_cancer	502474	0	0 pack-years	41 to 61.5 pack-years	1.054312	0.457807
parkinson	173863	0	0 cigarettes per day	15 to 24 cigarettes per day	-1.20397	1.862245
parkinson	173863	0	0 cigarettes per day	25 to 37.5 cigarettes per day	-0.10536	0.620748
parkinson	173863	0	0 cigarettes per day	1 to 14 cigarettes per day	0.09531	0.507885
parkinson	355955	1	0 cigarettes per day	1 to 14 cigarettes per day	0.587787	0.310374
parkinson	359151	0	0 cigarettes per day	21 to 31.5 cigarettes per day	-0.47804	0.50609
parkinson	359151	0	0 cigarettes per day	1 to 20 cigarettes per day	-0.43078	0.298273
parkinson	359152	0	0 cigarettes per day	1 to 14 cigarettes per day	-0.69315	0.383693
parkinson	359152	0	0 cigarettes per day	15 to 22.5 cigarettes per day	-1.20397	0.280258
parkinson	359154	1	0 cigarettes per day	12.7 to 19.2 cigarettes per day	-1.07881	0.210084
parkinson	359154	1	0 cigarettes per day	7 to 12.7 cigarettes per day	-0.99425	0.199945
parkinson	359154	0	0 cigarettes per day	19.2 to 60 cigarettes per day	-0.94161	0.196232
parkinson	359154	0	0 cigarettes per day	0 to 7 cigarettes per day	-0.63488	0.182903
parkinson	359157	0	0 cigarettes per day	1 to 10 cigarettes per day	-0.24846	0.30416
parkinson	359157	0	0 cigarettes per day	31 to 46.5 cigarettes per day	-0.26136	0.72555

parkinson	359157	0	0 cigarettes per day	11 to 20 cigarettes per day	-0.3285	0.276361
parkinson	359157	0	0 cigarettes per day	21 to 30 cigarettes per day	-0.40048	0.464514
parkinson	359157	0	0 cigarettes per day	21 to 30 cigarettes per day	-0.51919	0.287258
parkinson	359157	0	0 cigarettes per day	11 to 20 cigarettes per day	-0.73397	0.244473
parkinson	359157	0	0 cigarettes per day	1 to 10 cigarettes per day	-0.79851	0.328798
parkinson	359157	0	0 cigarettes per day	31 to 46.5 cigarettes per day	-1.20397	0.527211
parkinson	359158	1	0 cigarettes per day	21 to 31.5 cigarettes per day	0.815365	0.237042
parkinson	359158	1	0 cigarettes per day	0 to 19 cigarettes per day	1.695616	0.268208
parkinson	359159	0	0 cigarettes per day	30 to 45 cigarettes per day	-1.96611	1.767493
parkinson	359159	0	0 cigarettes per day	10 to 19 cigarettes per day	-0.44629	0.342793
parkinson	359159	0	0 cigarettes per day	30 to 45 cigarettes per day	-0.41552	0.200989
parkinson	359159	0	0 cigarettes per day	20 to 29 cigarettes per day	-0.35667	0.353499
parkinson	359159	0	0 cigarettes per day	20 to 29 cigarettes per day	-0.27444	0.1779
parkinson	359159	0	0 cigarettes per day	10 to 19 cigarettes per day	-0.13926	0.196458
parkinson	359159	0	0 cigarettes per day	0 to 9 cigarettes per day	-0.01005	0.21645
parkinson	359159	0	0 cigarettes per day	0 to 9 cigarettes per day	0.113329	0.23688
parkinson	359160	0	0 cigarettes per day	0 to 19 cigarettes per day	-0.38566	0.427671
parkinson	359160	0	0 cigarettes per day	21 to 31.5 cigarettes per day	-1.10866	0.347866
parkinson	359161	0	0 cigarettes per day	21 to 31.5 cigarettes per day	-2.20727	1.7718
parkinson	359161	0	0 cigarettes per day	0 to 19 cigarettes per day	-0.3285	0.279904
parkinson	359162	0	0 cigarettes per day	21 to 31.5 cigarettes per day	-1.83258	0.57398
parkinson	359162	0	0 cigarettes per day	11 to 20 cigarettes per day	-0.79851	1.547619
parkinson	359162	0	0 cigarettes per day	11 to 20 cigarettes per day	-0.52763	0.229159
parkinson	359162	0	0 cigarettes per day	1 to 10 cigarettes per day	-0.04082	0.236501
parkinson	359162	1	0 cigarettes per day	21 to 31.5 cigarettes per day	0.631272	0.419561
parkinson	359162	1	0 cigarettes per day	1 to 10 cigarettes per day	0.920283	0.2612
parkinson	359164	0	0 cigarettes per day	11 to 20 cigarettes per day	-0.35667	0.335277
parkinson	359164	0	0 cigarettes per day	1 to 10 cigarettes per day	-0.35667	0.517493
parkinson	359164	0	0 cigarettes per day	21 to 31.5 cigarettes per day	-1.04982	0.880466
parkinson	359307	0	0 cigarettes per day	20 to 39 cigarettes per day	-0.67334	0.370148
parkinson	359307	0	0 cigarettes per day	41 to 61.5 cigarettes per day	-0.34249	0.467088
parkinson	359307	0	0 cigarettes per day	0 to 19 cigarettes per day	-0.26136	0.404188
parkinson	502009	0	0 cigarettes per day	12 to 18 cigarettes per day	-0.61619	0.318323
parkinson	502009	0	0 cigarettes per day	1 to 12 cigarettes per day	-0.51083	0.450186
parkinson	502009	0	0 cigarettes per day	1 to 12 cigarettes per day	-0.91629	0.669894
parkinson	502009	0	0 cigarettes per day	12 to 18 cigarettes per day	-0.38566	0.203701
parkinson	502009	0	0 cigarettes per day	1 to 12 cigarettes per day	-0.09431	0.192527
parkinson	502009	0	0 cigarettes per day	12 to 18 cigarettes per day	-0.94161	0.366732
parkinson	502009	0	0 cigarettes per day	1 to 12 cigarettes per day	-0.23572	0.167712
parkinson	502009	0	0 cigarettes per day	12 to 18 cigarettes per day	-0.47804	0.405045
parkinson	502009	0	0 cigarettes per day	1 to 12 cigarettes per day	0.076961	0.327761
parkinson	502009	0	0 cigarettes per day	12 to 18 cigarettes per day	-0.63488	0.322172
parkinson	502009	0	0 cigarettes per day	12 to 18 cigarettes per day	-0.57982	0.151292

parkinson	502009	0	0 cigarettes per day	1 to 12 cigarettes per day	-0.18633	0.195885
parkinson	502009	0	0 cigarettes per day	1 to 12 cigarettes per day	-0.99425	0.4599
parkinson	502009	0	0 cigarettes per day	1 to 12 cigarettes per day	-0.03046	0.307617
parkinson	502009	0	0 cigarettes per day	12 to 18 cigarettes per day	-0.52763	0.381661
peptic_ulcer	262563	0	0 cigarettes per day	0 to 19 cigarettes per day	0.470004	0.255102
peptic_ulcer	328259	0	0 cigarettes per day	1 to 7 cigarettes per day	0.587787	0.365646
peptic_ulcer	328259	0	0 cigarettes per day	8 to 15 cigarettes per day	0.683097	0.407133
peptic_ulcer	328259	0	0 cigarettes per day	8 to 15 cigarettes per day	0.693147	0.628827
peptic_ulcer	328259	1	0 cigarettes per day	16 to 25 cigarettes per day	0.81978	1.853142
peptic_ulcer	328259	0	0 cigarettes per day	1 to 7 cigarettes per day	0.871293	0.376783
peptic_ulcer	328259	0	0 cigarettes per day	16 to 25 cigarettes per day	1.163151	0.460778
peptic_ulcer	328259	0	0 cigarettes per day	26 to 39 cigarettes per day	1.291984	1.033724
peptic_ulcer	338384	0	0 cigarettes per day	15 to 20 cigarettes per day	1.609438	0.642857
peptic_ulcer	338384	0	0 cigarettes per day	21 to 31.5 cigarettes per day	0.916291	0.612245
peptic_ulcer	338384	0	0 cigarettes per day	15 to 20 cigarettes per day	1.064711	0.492611
peptic_ulcer	338384	0	0 cigarettes per day	1 to 9 cigarettes per day	0.405465	0.493197
peptic_ulcer	338384	0	0 cigarettes per day	10 to 14 cigarettes per day	0.262364	0.510204
peptic_ulcer	338384	0	0 cigarettes per day	15 to 20 cigarettes per day	0.693147	0.395408
peptic_ulcer	349377	0	0 cigarettes per day	0 to 15 cigarettes per day	0.215111	0.357966
peptic_ulcer	349377	0	0 cigarettes per day	16 to 24 cigarettes per day	0.385262	0.452936
peptic_ulcer	349377	1	0 cigarettes per day	0 to 15 cigarettes per day	1.348073	0.277631
peptic_ulcer	349377	1	0 cigarettes per day	16 to 24 cigarettes per day	1.467874	0.298599
peptic_ulcer	349381	0	0 cigarettes per day	1 to 14 cigarettes per day	0.029559	0.616703
peptic_ulcer	349381	0	0 cigarettes per day	14 to 24 cigarettes per day	0.029559	0.537448
peptic_ulcer	349381	0	0 cigarettes per day	25 to 37.5 cigarettes per day	0.24686	0.49426
peptic_ulcer	349542	0	0 cigarettes per day	1 to 9 cigarettes per day	0.559616	0.370262
peptic_ulcer	349542	0	0 cigarettes per day	10 to 20 cigarettes per day	0.703098	0.239947
peptic_ulcer	349542	0	0 cigarettes per day	21 to 31.5 cigarettes per day	0.832909	0.353815
peptic_ulcer	359166	0	0 cigarettes per day	0 to 14 cigarettes per day	0.254642	0.395507
peptic_ulcer	359166	0	0 cigarettes per day	15 to 22.5 cigarettes per day	0.732368	0.381427
peripheral_artery_disease	350659	0	0 cigarettes per day	0 to 14 cigarettes per day	2.189416	0.310522
peripheral_artery_disease	350659	1	0 cigarettes per day	15 to 22.5 cigarettes per day	2.830268	0.239299
peripheral_artery_disease	359221	0	0 cigarettes per day	1 to 20 cigarettes per day	1.205971	0.306275
peripheral_artery_disease	359221	0	0 cigarettes per day	19 to 28.5 cigarettes per day	1.534714	0.294687
peripheral_artery_disease	359223	0	0 cigarettes per day	0 to 24 cigarettes per day	1.054312	0.329766
peripheral_artery_disease	359223	0	0 cigarettes per day	25 to 37.5 cigarettes per day	2.53449	0.485928
peripheral_artery_disease	369290	0	0 cigarettes per day	1 to 20 cigarettes per day	2.116256	1.05533
peripheral_artery_disease	369290	0	0 cigarettes per day	21 to 40 cigarettes per day	2.351375	1.148031
peripheral_artery_disease	369290	0	0 cigarettes per day	41 to 61.5 cigarettes per day	2.70805	1.605736
peripheral_artery_disease	462792	0	0 cigarettes per day	1 to 14 cigarettes per day	1.249902	0.11089
peripheral_artery_disease	462792	0	0 cigarettes per day	15 to 24 cigarettes per day	1.773256	0.089642
peripheral_artery_disease	462792	0	0 cigarettes per day	25 to 37.5 cigarettes per day	1.98238	0.102145
peripheral_artery_disease	501896	0	0 cigarettes per day	1 to 19 cigarettes per day	0.982078	0.157216

peripheral_artery_disease	501896	1	0 cigarettes per day	20 to 30 cigarettes per day	1.311032	0.1312
prostate_cancer	165518	0	0 cigarettes per day	0 to 30 cigarettes per day	0.262364	0.353218
prostate_cancer	165518	0	0 cigarettes per day	31 to 55 cigarettes per day	0.262364	0.392465
prostate_cancer	165518	0	0 cigarettes per day	56 to 84 cigarettes per day	0.693147	0.344388
prostate_cancer	165571	0	0 cigarettes per day	1 to 20 cigarettes per day	0.262364	0.392971
prostate_cancer	165571	0	0 cigarettes per day	20 to 30 cigarettes per day	0.470004	0.438176
prostate_cancer	165618	0	0 cigarettes per day	0 to 10 cigarettes per day	-0.69315	0.33145
prostate_cancer	165618	0	0 cigarettes per day	11 to 16.5 cigarettes per day	-0.51083	0.353647
prostate_cancer	328210	0	0 cigarettes per day	0 to 20 cigarettes per day	0.322083	0.201635
prostate_cancer	328210	0	0 cigarettes per day	20 to 30 cigarettes per day	-0.38566	0.332772
prostate_cancer	328339	0	0 cigarettes per day	0 to 19 cigarettes per day	-0.34249	0.643145
prostate_cancer	328339	0	0 cigarettes per day	60 to 90 cigarettes per day	0.277632	0.479283
prostate_cancer	328339	0	0 cigarettes per day	40 to 59 cigarettes per day	0.34359	0.28405
prostate_cancer	328339	0	0 cigarettes per day	20 to 39 cigarettes per day	0.457425	0.269633
prostate_cancer	343354	0	0 cigarettes per day	39 to 58.5 cigarettes per day	0.41211	0.117228
prostate_cancer	343354	0	0 cigarettes per day	10 to 20 cigarettes per day	0.139762	0.048527
prostate_cancer	343354	0	0 cigarettes per day	21 to 39 cigarettes per day	0.207014	0.06018
prostate_cancer	343354	0	0 cigarettes per day	15 to 22.5 cigarettes per day	0.165514	0.179878
prostate_cancer	343354	0	0 cigarettes per day	1 to 14 cigarettes per day	0.207014	0.195837
prostate_cancer	343354	0	0 cigarettes per day	1 to 9 cigarettes per day	0.10436	0.068744
prostate_cancer	347383	0	0 cigarettes per day	1 to 19 cigarettes per day	0.470004	0.361496
prostate_cancer	347383	0	0 cigarettes per day	20 to 29 cigarettes per day	0.530628	0.376507
prostate_cancer	347383	0	0 cigarettes per day	30 to 45 cigarettes per day	0.336472	0.611708
prostate_cancer	347385	0	0 cigarettes per day	0 to 20 cigarettes per day	0	0.250212
prostate_cancer	347385	0	0 cigarettes per day	20 to 30 cigarettes per day	0.641854	0.242112
prostate_cancer	347435	0	0 cigarettes per day	20 to 30 cigarettes per day	0.223144	0.11507
prostate_cancer	347435	0	0 cigarettes per day	20 to 20 cigarettes per day	0.322083	0.112547
prostate_cancer	347435	0	0 cigarettes per day	10 to 20 cigarettes per day	0.457425	0.127812
prostate_cancer	347435	0	0 cigarettes per day	0 to 10 cigarettes per day	0.285179	0.164576
prostate_cancer	347448	0	0 cigarettes per day	20 to 39 cigarettes per day	-0.04082	0.103635
prostate_cancer	347448	0	0 cigarettes per day	40 to 60 cigarettes per day	0.131028	0.105174
prostate_cancer	347448	0	0 cigarettes per day	0.25 to 19 cigarettes per day	0.182322	0.082908
prostate_cancer	347451	0	0 cigarettes per day	10 to 19 cigarettes per day	-0.16252	0.200322
prostate_cancer	347451	0	0 cigarettes per day	20 to 30 cigarettes per day	0.457425	0.263432
prostate_cancer	347451	0	0 cigarettes per day	10 to 19 cigarettes per day	0.10436	0.236647
prostate_cancer	347451	0	0 cigarettes per day	20 to 30 cigarettes per day	-0.05129	0.220229
prostate_cancer	347451	0	0 cigarettes per day	0 to 10 cigarettes per day	0.10436	0.257715
prostate_cancer	347451	0	0 cigarettes per day	0 to 10 cigarettes per day	0.113329	0.473908
prostate_cancer	347451	0	0 cigarettes per day	10 to 19 cigarettes per day	-0.05129	0.17872
prostate_cancer	347451	0	0 cigarettes per day	0 to 10 cigarettes per day	-0.65393	0.483321
prostate_cancer	347451	0	0 cigarettes per day	10 to 19 cigarettes per day	0.029559	0.278837
prostate_cancer	347451	0	0 cigarettes per day	20 to 30 cigarettes per day	0.00995	0.224964
prostate_cancer	347451	0	0 cigarettes per day	20 to 30 cigarettes per day	0.322083	0.311185

prostate_cancer	347451	0	0 cigarettes per day	0 to 10 cigarettes per day	0.113329	0.334233
prostate_cancer	347454	1	0 cigarettes per day	0 to 20 cigarettes per day	-0.09431	0.036708
prostate_cancer	347454	1	0 cigarettes per day	20 to 30 cigarettes per day	-0.28768	0.047126
prostate_cancer	347454	1	0 cigarettes per day	0 to 20 cigarettes per day	0.582216	0.174807
prostate_cancer	347454	0	0 cigarettes per day	20 to 30 cigarettes per day	0.431782	0.214337
prostate_cancer	347458	0	0 cigarettes per day	15 to 22.5 cigarettes per day	0.722706	0.349217
prostate_cancer	347458	0	0 cigarettes per day	15 to 22.5 cigarettes per day	0.593327	0.290337
prostate_cancer	347458	0	0 cigarettes per day	10 to 14 cigarettes per day	0.565314	0.36381
prostate_cancer	347458	0	0 cigarettes per day	1 to 9 cigarettes per day	0.131028	0.429646
prostate_cancer	347458	0	0 cigarettes per day	10 to 14 cigarettes per day	0.223144	0.336735
prostate_cancer	347458	0	0 cigarettes per day	1 to 9 cigarettes per day	0.223144	0.530612
prostate_cancer	347464	0	0 cigarettes per day	1 to 20 cigarettes per day	0.530628	0.397486
prostate_cancer	347464	0	0 cigarettes per day	20 to 30 cigarettes per day	0.641854	0.440618
prostate_cancer	356281	0	0 cigarettes per day	0 to 10 cigarettes per day	-0.24846	0.538606
prostate_cancer	356281	0	0 cigarettes per day	10 to 20 cigarettes per day	-0.35667	0.455611
prostate_cancer	356281	0	0 cigarettes per day	20 to 30 cigarettes per day	0.04879	0.470874
prostate_cancer	358689	1	0 cigarettes per day	20 to 30 cigarettes per day	-0.44629	0.203284
prostate_cancer	359183	1	0 cigarettes per day	20 to 30 cigarettes per day	-0.3285	0.070333
prostate_cancer	359183	0	0 cigarettes per day	0 to 10 cigarettes per day	-0.15082	0.056261
prostate_cancer	359183	1	0 cigarettes per day	10 to 19 cigarettes per day	-0.15082	0.05359
prostate_cancer	359185	0	0 cigarettes per day	1 to 14 cigarettes per day	-0.03046	0.055159
prostate_cancer	359185	1	0 cigarettes per day	15 to 24 cigarettes per day	-0.10536	0.059463
prostate_cancer	359185	1	0 cigarettes per day	25 to 37.5 cigarettes per day	-0.13926	0.09273
prostate_cancer	359185	0	0 cigarettes per day	1 to 14 cigarettes per day	0.173953	0.190452
prostate_cancer	359185	0	0 cigarettes per day	15 to 24 cigarettes per day	0.270027	0.191955
prostate_cancer	359185	0	0 cigarettes per day	25 to 37.5 cigarettes per day	0.593327	0.247613
prostate_cancer	359211	0	0 cigarettes per day	13 to 19.5 cigarettes per day	-0.12783	0.168593
prostate_cancer	359211	0	0 cigarettes per day	0 to 13 cigarettes per day	0.067659	0.188612
prostate_cancer	359212	0	0 cigarettes per day	26 to 39 cigarettes per day	0.215111	0.154296
prostate_cancer	359212	0	0 cigarettes per day	18 to 25 cigarettes per day	0.215111	0.154296
prostate_cancer	359212	0	0 cigarettes per day	1 to 10 cigarettes per day	-0.05129	0.161117
prostate_cancer	359212	0	0 cigarettes per day	11 to 17 cigarettes per day	-0.17435	0.191327
prostate_cancer	359215	0	0 cigarettes per day	0 to 19 cigarettes per day	-0.40048	0.159915
prostate_cancer	359215	1	0 cigarettes per day	20 to 39 cigarettes per day	-0.17435	0.097182
prostate_cancer	359218	0	0 cigarettes per day	1 to 30 cigarettes per day	0.336472	0.400875
prostate_cancer	359218	0	0 cigarettes per day	31 to 46.5 cigarettes per day	0.182322	0.255102
prostate_cancer	428283	0	0 cigarettes per day	60 to 90 cigarettes per day	0.277632	0.479283
prostate_cancer	428283	0	0 cigarettes per day	40 to 59 cigarettes per day	0.34359	0.28405
prostate_cancer	428283	0	0 cigarettes per day	0 to 19 cigarettes per day	-0.34249	0.643145
prostate_cancer	428283	0	0 cigarettes per day	20 to 39 cigarettes per day	0.457425	0.269633
rheumatoid_arthritis	261500	0	0 cigarettes per day	1 to 14 cigarettes per day	0.139762	0.173026
rheumatoid_arthritis	261500	0	0 cigarettes per day	15 to 24 cigarettes per day	0.392042	0.143064
rheumatoid_arthritis	261500	0	0 cigarettes per day	25 to 37.5 cigarettes per day	0.587787	0.15873

rheumatoid_arthritis	261510	0	0 cigarettes per day	15 to 22.5 cigarettes per day	0.09531	0.510204
rheumatoid_arthritis	261510	0	0 cigarettes per day	1 to 14 cigarettes per day	0.09531	0.185529
rheumatoid_arthritis	261510	1	0 cigarettes per day	15 to 22.5 cigarettes per day	1.386294	0.357143
rheumatoid_arthritis	261510	1	0 cigarettes per day	1 to 14 cigarettes per day	1.481605	0.359462
rheumatoid_arthritis	350653	0	0 cigarettes per day	15 to 22.5 cigarettes per day	0.378436	0.221904
rheumatoid_arthritis	350653	0	0 cigarettes per day	8 to 14 cigarettes per day	0.783902	0.199189
rheumatoid_arthritis	350653	1	0 cigarettes per day	1 to 7 cigarettes per day	0.837248	0.195468
rheumatoid_arthritis	353080	0	0 cigarettes per day	1 to 15 cigarettes per day	0.770108	0.393282
rheumatoid_arthritis	353080	0	0 cigarettes per day	17 to 25.5 cigarettes per day	-0.03046	0.489165
rheumatoid_arthritis	502082	0	0 cigarettes per day	1 to 10 cigarettes per day	-0.10536	0.770517
rheumatoid_arthritis	502082	0	0 cigarettes per day	11 to 20 cigarettes per day	0.916291	0.457081
rheumatoid_arthritis	502082	0	0 cigarettes per day	21 to 31.5 cigarettes per day	1.609438	0.515889
rheumatoid_arthritis	502082	0	0 cigarettes per day	1 to 10 cigarettes per day	0.336472	0.379611
rheumatoid_arthritis	502082	0	0 cigarettes per day	11 to 20 cigarettes per day	0.587787	0.247296
rheumatoid_arthritis	502082	0	0 cigarettes per day	21 to 31.5 cigarettes per day	0.741937	0.287248
rheumatoid_arthritis	502084	0	0 cigarettes per day	1 to 14 cigarettes per day	0.207014	0.11957
rheumatoid_arthritis	502084	0	0 cigarettes per day	15 to 24 cigarettes per day	0.470004	0.106038
rheumatoid_arthritis	502084	0	0 cigarettes per day	25 to 37.5 cigarettes per day	0.524729	0.134207
stomach_cancer	309835	0	0 pack-years	1 to 13 pack-years	-0.10536	0.296722
stomach_cancer	309835	1	0 pack-years	14 to 31 pack-years	0.832909	0.240934
stomach_cancer	309835	1	0 pack-years	32 to 54 pack-years	1.029619	0.228015
stomach_cancer	309835	0	0 pack-years	54 to 81 pack-years	0.916291	0.256511
stomach_cancer	309835	0	0 pack-years	1 to 13 pack-years	0.182322	0.20687
stomach_cancer	309835	0	0 pack-years	14 to 31 pack-years	0.405465	0.223334
stomach_cancer	309835	0	0 pack-years	32 to 54 pack-years	0.530628	0.197242
stomach_cancer	309835	0	0 pack-years	54 to 81 pack-years	0.741937	0.229792
stomach_cancer	328266	0	0 pack-years	41 to 61.5 pack-years	1.029619	0.362598
stomach_cancer	328266	0	0 pack-years	0 to 20 pack-years	0.262364	0.392971
stomach_cancer	328266	0	0 pack-years	20 to 40 pack-years	0.405465	0.379611
stomach_cancer	339695	0	0 pack-years	0.01 to 17.99 pack-years	0.262364	0.379477
stomach_cancer	339695	0	0 pack-years	18 to 32.99 pack-years	0.336472	0.390786
stomach_cancer	339695	0	0 pack-years	33 to 49.5 pack-years	0.832909	0.32106
stomach_cancer	339697	0	0 pack-years	10 to 19 pack-years	0.019803	0.37606
stomach_cancer	339697	0	0 pack-years	20 to 30 pack-years	0.239017	0.457081
stomach_cancer	339697	0	0 pack-years	1 to 9 pack-years	-0.16252	0.332502
stomach_cancer	339697	0	0 pack-years	40 to 60 pack-years	0.518794	0.16175
stomach_cancer	339697	0	0 pack-years	20 to 39 pack-years	0.431782	0.14871
stomach_cancer	339697	0	0 pack-years	10 to 19 pack-years	0.04879	0.17167
stomach_cancer	339697	0	0 pack-years	1 to 9 pack-years	0	0.176823
stomach_cancer	340513	0	0 pack-years	1 to 13 pack-years	0.470004	0.271046
stomach_cancer	340513	0	0 pack-years	14 to 25 pack-years	0.530628	0.270108
stomach_cancer	340513	0	0 pack-years	26 to 50 pack-years	0.741937	0.279397
stomach_cancer	340513	0	0 pack-years	51 to 76.5 pack-years	0.832909	0.266193

stomach_cancer	340528	1	0 pack-years	33 to 49.5 pack-years	1.131402	0.485517
stomach_cancer	340528	0	0 pack-years	33 to 49.5 pack-years	0.405465	0.272109
stomach_cancer	340528	0	0 pack-years	19 to 32 pack-years	0.405465	0.272109
stomach_cancer	340528	0	0 pack-years	1 to 18 pack-years	0.09531	0.556586
stomach_cancer	340528	0	0 pack-years	1 to 18 pack-years	-0.10536	0.283447
stomach_cancer	340528	0	0 pack-years	19 to 32 pack-years	0.182322	0.57398
stomach_cancer	340554	0	0 pack-years	41 to 61.5 pack-years	0.470004	0.302934
stomach_cancer	340554	1	0 pack-years	41 to 61.5 pack-years	-0.35667	0.400875
stomach_cancer	340554	0	0 pack-years	0 to 40 pack-years	-0.10536	0.311791
stomach_cancer	340554	0	0 pack-years	0 to 40 pack-years	0.405465	0.255102
stomach_cancer	340578	0	0 pack-years	30 to 45 pack-years	0.182322	0.191327
stomach_cancer	340578	0	0 pack-years	20 to 29 pack-years	0.09531	0.20872
stomach_cancer	340578	0	0 pack-years	10 to 19 pack-years	0	0.178571
stomach_cancer	340578	0	0 pack-years	0 to 9 pack-years	0	0.204082
stomach_cancer	340582	1	0 pack-years	40 to 59 pack-years	1.047319	0.145005
stomach_cancer	340582	0	0 pack-years	60 to 90 pack-years	1.043804	0.244323
stomach_cancer	340582	0	0 pack-years	60 to 90 pack-years	1.004302	0.241085
stomach_cancer	340582	1	0 pack-years	40 to 59 pack-years	0.989541	0.14794
stomach_cancer	340582	1	0 pack-years	20 to 39 pack-years	0.788457	0.169295
stomach_cancer	340582	0	0 pack-years	60 to 90 pack-years	0.760806	0.158545
stomach_cancer	340582	0	0 pack-years	60 to 90 pack-years	0.732368	0.161892
stomach_cancer	340582	1	0 pack-years	20 to 39 pack-years	0.678034	0.170931
stomach_cancer	340582	0	0 pack-years	0 to 19 pack-years	0.536493	0.241676
stomach_cancer	340582	0	0 pack-years	0 to 19 pack-years	0.444686	0.21422
stomach_cancer	340582	0	0 pack-years	0 to 19 pack-years	0.350657	0.24612
stomach_cancer	340582	0	0 pack-years	20 to 39 pack-years	0.231112	0.216634
stomach_cancer	340582	0	0 pack-years	20 to 39 pack-years	0.215111	0.216014
stomach_cancer	340582	0	0 pack-years	40 to 59 pack-years	0.182322	0.272109
stomach_cancer	340582	0	0 pack-years	40 to 59 pack-years	0.14842	0.268297
stomach_cancer	340582	0	0 pack-years	0 to 19 pack-years	0.476234	0.213905
stomach_cancer	343580	0	0 pack-years	75.05 to 112.5 pack-years	0.641854	0.304572
stomach_cancer	343580	0	0 pack-years	50.05 to 75 pack-years	0.530628	0.27161
stomach_cancer	343580	0	0 pack-years	0.05 to 25 pack-years	0.470004	0.289536
stomach_cancer	343580	0	0 pack-years	25.05 to 50 pack-years	0.470004	0.253381
stomach_cancer	347810	0	0 pack-years	1 to 19.95 pack-years	-0.75502	0.705601
stomach_cancer	347810	0	0 pack-years	1 to 19.95 pack-years	-0.73397	0.71216
stomach_cancer	347810	0	0 pack-years	40 to 60 pack-years	0.270027	0.140209
stomach_cancer	347810	0	0 pack-years	40 to 60 pack-years	0.285179	0.140018
stomach_cancer	347810	0	0 pack-years	20 to 39.95 pack-years	0.300105	0.139834
stomach_cancer	347810	0	0 pack-years	1 to 19.95 pack-years	0.307485	0.215711
stomach_cancer	347810	0	0 pack-years	20 to 39.95 pack-years	0.307485	0.140681
stomach_cancer	347810	0	0 pack-years	1 to 19.95 pack-years	0.322083	0.216282
stomach_cancer	347810	0	0 pack-years	20 to 39.95 pack-years	0.582216	0.464599

stomach_cancer	347810	0	0 pack-years	20 to 39.95 pack-years	0.615186	0.460563
stomach_cancer	502130	0	0 pack-years	20 to 29 pack-years	0.329304	0.316158
stomach_cancer	502130	0	0 pack-years	0 to 10 pack-years	-0.17435	0.510136
stomach_cancer	502130	0	0 pack-years	10 to 19 pack-years	0.157004	0.33183
stomach_cancer	502130	0	0 pack-years	30 to 45 pack-years	0.732368	0.232197
stomach_cancer	502141	0	0 pack-years	1 to 30 pack-years	0.099845	0.098402
stomach_cancer	502141	0	0 pack-years	31 to 45 pack-years	0.167377	0.097462
stroke	122237	0	0 cigarettes per day	21 to 31.5 cigarettes per day	0.182322	1.766582
stroke	122237	0	0 cigarettes per day	10 to 19 cigarettes per day	0.405465	1.413265
stroke	122237	0	0 cigarettes per day	1 to 9 cigarettes per day	0.641854	1.115736
stroke	122237	0	0 cigarettes per day	10 to 19 cigarettes per day	0.641854	1.115736
stroke	122237	0	0 cigarettes per day	21 to 31.5 cigarettes per day	0.875469	0.883291
stroke	122237	0	0 cigarettes per day	1 to 9 cigarettes per day	0.993252	0.785147
stroke	155207	0	0 cigarettes per day	25 to 37.5 cigarettes per day	1.000632	0.172569
stroke	155207	0	0 cigarettes per day	25 to 37.5 cigarettes per day	0.871293	0.129152
stroke	155207	0	0 cigarettes per day	15 to 24 cigarettes per day	0.951658	0.09357
stroke	155207	0	0 cigarettes per day	1 to 14 cigarettes per day	0.615186	0.100662
stroke	155207	0	0 cigarettes per day	1 to 14 cigarettes per day	0.277632	0.117888
stroke	155207	0	0 cigarettes per day	15 to 24 cigarettes per day	0.698135	0.175145
stroke	165539	0	0 cigarettes per day	1 to 9 cigarettes per day	0.09531	0.143785
stroke	165539	0	0 cigarettes per day	10 to 15 cigarettes per day	0.405465	0.098639
stroke	174238	0	0 cigarettes per day	1 to 14 cigarettes per day	0.314811	1.547371
stroke	174238	0	0 cigarettes per day	15 to 24 cigarettes per day	0.457425	1.341708
stroke	174238	0	0 cigarettes per day	25 to 37.5 cigarettes per day	0.641854	1.115736
stroke	193978	0	0 cigarettes per day	10 to 19 cigarettes per day	1.043804	0.397025
stroke	193978	0	0 cigarettes per day	20 to 30 cigarettes per day	1.302913	0.467918
stroke	193978	0	0 cigarettes per day	1 to 9 cigarettes per day	1.118415	0.521042
stroke	193978	0	0 cigarettes per day	10 to 19 cigarettes per day	1.004302	0.239217
stroke	193978	0	0 cigarettes per day	1 to 9 cigarettes per day	0.858662	0.432376
stroke	193978	0	0 cigarettes per day	20 to 30 cigarettes per day	0.97456	0.241625
stroke	193978	0	0 cigarettes per day	10 to 19 cigarettes per day	0.982078	0.312428
stroke	193978	0	0 cigarettes per day	20 to 30 cigarettes per day	0.756122	0.280253
stroke	193978	0	0 cigarettes per day	1 to 9 cigarettes per day	0.392042	1.018685
stroke	298316	0	0 cigarettes per day	0 to 20 cigarettes per day	1.410987	0.3111
stroke	298316	1	0 cigarettes per day	21 to 31.5 cigarettes per day	2.406945	0.457345
stroke	324092	0	0 cigarettes per day	1 to 19 cigarettes per day	0.530628	0.165066
stroke	324092	0	0 cigarettes per day	21 to 31.5 cigarettes per day	0.788457	0.162338
stroke	324092	0	0 cigarettes per day	1 to 19 cigarettes per day	0.470004	0.143495
stroke	328259	0	0 cigarettes per day	26 to 39 cigarettes per day	0.357674	0.390681
stroke	328259	0	0 cigarettes per day	16 to 25 cigarettes per day	-0.52763	0.453995
stroke	328259	0	0 cigarettes per day	1 to 7 cigarettes per day	-0.08338	0.119232
stroke	328259	0	0 cigarettes per day	1 to 7 cigarettes per day	-0.01005	0.108225
stroke	328259	1	0 cigarettes per day	16 to 25 cigarettes per day	0	0.183673

stroke	328259	1	0 cigarettes per day	8 to 15 cigarettes per day	0.04879	0.133625
stroke	328259	0	0 cigarettes per day	8 to 15 cigarettes per day	0.231112	0.141723
stroke	328460	0	0 cigarettes per day	0 to 9 cigarettes per day	0.765468	0.290698
stroke	328460	0	0 cigarettes per day	21 to 31.5 cigarettes per day	-0.04082	0.326849
stroke	328460	0	0 cigarettes per day	10 to 19 cigarettes per day	0.24686	0.277025
stroke	330677	0	0 cigarettes per day	1 to 9 cigarettes per day	0.182322	0.065901
stroke	330677	1	0 cigarettes per day	10 to 19 cigarettes per day	0.19062	0.082223
stroke	330677	1	0 cigarettes per day	10 to 19 cigarettes per day	0.198851	0.052275
stroke	330677	0	0 cigarettes per day	1 to 9 cigarettes per day	0.207014	0.05185
stroke	330677	1	0 cigarettes per day	21 to 31.5 cigarettes per day	0.314811	0.046551
stroke	330677	1	0 cigarettes per day	21 to 31.5 cigarettes per day	0.350657	0.100604
stroke	330932	0	0 cigarettes per day	1 to 9 cigarettes per day	0.34359	0.40165
stroke	330932	0	0 cigarettes per day	10 to 20 cigarettes per day	0.157004	0.97026
stroke	330932	0	0 cigarettes per day	22 to 33 cigarettes per day	0.500775	0.64162
stroke	330932	0	0 cigarettes per day	1 to 9 cigarettes per day	0.683097	0.315657
stroke	330932	0	0 cigarettes per day	10 to 20 cigarettes per day	0.815365	0.320571
stroke	330932	0	0 cigarettes per day	22 to 33 cigarettes per day	1.927164	0.979562
stroke	331371	0	0 cigarettes per day	1 to 19 cigarettes per day	0.565314	0.243506
stroke	331371	0	0 cigarettes per day	1 to 19 cigarettes per day	0.41871	0.181257
stroke	331371	0	0 cigarettes per day	20 to 30 cigarettes per day	1.153732	0.449044
stroke	331371	0	0 cigarettes per day	1 to 19 cigarettes per day	0.270027	0.477099
stroke	331371	0	0 cigarettes per day	20 to 30 cigarettes per day	0.24686	0.239158
stroke	331371	0	0 cigarettes per day	1 to 19 cigarettes per day	0.19062	0.375274
stroke	331371	1	0 cigarettes per day	20 to 30 cigarettes per day	-0.03046	0.194614
stroke	331371	0	0 cigarettes per day	20 to 30 cigarettes per day	-0.59784	1.846011
stroke	331705	0	0 cigarettes per day	0 to 9 cigarettes per day	0.14842	0.101161
stroke	331705	0	0 cigarettes per day	0 to 9 cigarettes per day	0.329304	0.067905
stroke	331710	0	0 cigarettes per day	1 to 20 cigarettes per day	0.350657	0.370078
stroke	331710	0	0 cigarettes per day	1 to 20 cigarettes per day	0.470004	0.299745
stroke	331710	0	0 cigarettes per day	22 to 33 cigarettes per day	0.774727	0.377363
stroke	331710	0	0 cigarettes per day	22 to 33 cigarettes per day	1.363537	0.764654
stroke	332100	0	0 cigarettes per day	1 to 19 cigarettes per day	0.131028	0.129789
stroke	332100	1	0 cigarettes per day	20 to 39 cigarettes per day	0.277632	0.106293
stroke	332100	0	0 cigarettes per day	40 to 60 cigarettes per day	0.29267	0.171337
stroke	332100	0	0 cigarettes per day	1 to 19 cigarettes per day	1.160021	0.530996
stroke	332100	0	0 cigarettes per day	40 to 60 cigarettes per day	1.319086	0.656172
stroke	332100	0	0 cigarettes per day	20 to 39 cigarettes per day	1.329724	0.471061
stroke	332104	0	0 cigarettes per day	1 to 19 cigarettes per day	0.647103	0.177636
stroke	332104	0	0 cigarettes per day	22 to 33 cigarettes per day	0.751416	0.179293
stroke	332104	0	0 cigarettes per day	20 to 20 cigarettes per day	0.576613	0.199209
stroke	334028	0	0 cigarettes per day	35 to 52.5 cigarettes per day	2.324347	0.546897
stroke	334028	0	0 cigarettes per day	25 to 34 cigarettes per day	2.073172	0.467527
stroke	334028	0	0 cigarettes per day	1 to 14 cigarettes per day	1.453953	0.47027

stroke	334028	0	0 cigarettes per day	15 to 24 cigarettes per day	1.391282	0.421363
stroke	334410	0	0 cigarettes per day	1 to 15 cigarettes per day	0.688633	0.146066
stroke	334410	0	0 cigarettes per day	36 to 45 cigarettes per day	0.896272	0.14814
stroke	334410	0	0 cigarettes per day	16 to 25 cigarettes per day	0.903598	0.111405
stroke	334410	0	0 cigarettes per day	26 to 35 cigarettes per day	1.039863	0.126612
stroke	334731	0	0 cigarettes per day	25 to 37.5 cigarettes per day	0.565314	0.140596
stroke	334731	0	0 cigarettes per day	15 to 24 cigarettes per day	0.392042	0.162024
stroke	334731	0	0 cigarettes per day	25 to 37.5 cigarettes per day	0.322083	0.166371
stroke	334731	0	0 cigarettes per day	1 to 14 cigarettes per day	0.223144	0.17551
stroke	334731	0	0 cigarettes per day	1 to 14 cigarettes per day	0.076961	0.179516
stroke	334731	0	0 cigarettes per day	15 to 24 cigarettes per day	0.307485	0.161315
stroke	334814	0	0 cigarettes per day	1 to 10 cigarettes per day	-0.40048	0.932836
stroke	334814	0	0 cigarettes per day	22 to 33 cigarettes per day	0.792993	0.487118
stroke	334814	0	0 cigarettes per day	11 to 20 cigarettes per day	0.900161	0.413763
stroke	334967	0	0 cigarettes per day	21 to 31.5 cigarettes per day	0.948299	0.456084
stroke	334967	0	0 cigarettes per day	10 to 19 cigarettes per day	0.848286	0.466922
stroke	334967	0	0 cigarettes per day	1 to 9 cigarettes per day	0.144714	0.646966
stroke	335266	1	0 cigarettes per day	21 to 31.5 cigarettes per day	0.24686	0.109614
stroke	335266	1	0 cigarettes per day	10 to 20 cigarettes per day	0.039221	0.117739
stroke	335266	0	0 cigarettes per day	0 to 9 cigarettes per day	0.039221	0.117739
stroke	335914	0	0 cigarettes per day	10 to 19 cigarettes per day	0.34359	0.14293
stroke	335914	0	0 cigarettes per day	21 to 31.5 cigarettes per day	0.357674	0.137363
stroke	335914	0	0 cigarettes per day	1 to 9 cigarettes per day	0.672944	0.1692
stroke	335914	0	0 cigarettes per day	1 to 9 cigarettes per day	0.756122	0.259893
stroke	335914	0	0 cigarettes per day	10 to 19 cigarettes per day	0.81978	0.244988
stroke	335914	0	0 cigarettes per day	21 to 31.5 cigarettes per day	1.047319	0.266738
stroke	336216	0	0 cigarettes per day	40 to 60 cigarettes per day	0.737164	0.126941
stroke	336216	0	0 cigarettes per day	20 to 39 cigarettes per day	0.71295	0.078782
stroke	336216	0	0 cigarettes per day	20 to 39 cigarettes per day	0.732368	0.127551
stroke	336216	0	0 cigarettes per day	10 to 19 cigarettes per day	0.746688	0.099139
stroke	336216	0	0 cigarettes per day	0 to 9 cigarettes per day	0.698135	0.126916
stroke	336216	0	0 cigarettes per day	40 to 60 cigarettes per day	0.943906	0.178671
stroke	336216	0	0 cigarettes per day	20 to 39 cigarettes per day	0.993252	0.074641
stroke	336216	0	0 cigarettes per day	40 to 60 cigarettes per day	1.05779	0.307363
stroke	336216	0	0 cigarettes per day	20 to 39 cigarettes per day	0.81093	0.14059
stroke	336216	0	0 cigarettes per day	20 to 39 cigarettes per day	0.683097	0.085034
stroke	336216	0	0 cigarettes per day	10 to 19 cigarettes per day	0.615186	0.118588
stroke	336216	0	0 cigarettes per day	10 to 19 cigarettes per day	0.542324	0.115686
stroke	336216	0	0 cigarettes per day	0 to 9 cigarettes per day	0.198851	0.094095
stroke	336216	0	0 cigarettes per day	0 to 9 cigarettes per day	0.254642	0.142383
stroke	336216	1	0 cigarettes per day	10 to 19 cigarettes per day	0.277632	0.079236
stroke	336216	0	0 cigarettes per day	40 to 60 cigarettes per day	0.34359	0.416124
stroke	336216	0	0 cigarettes per day	0 to 9 cigarettes per day	0.00995	0.103556

stroke	336216	0	0 cigarettes per day	0 to 9 cigarettes per day	0.431782	0.139147
stroke	336216	0	0 cigarettes per day	40 to 60 cigarettes per day	0.438255	0.128374
stroke	336216	0	0 cigarettes per day	10 to 19 cigarettes per day	0.530628	0.090036
stroke	336216	1	0 cigarettes per day	20 to 39 cigarettes per day	0.371564	0.059817
stroke	336319	0	0 cigarettes per day	25 to 37.5 cigarettes per day	-1.20397	1.794218
stroke	336319	0	0 cigarettes per day	15 to 24 cigarettes per day	0.173953	0.621677
stroke	336319	0	0 cigarettes per day	15 to 24 cigarettes per day	0.29267	0.426439
stroke	336319	0	0 cigarettes per day	1 to 14 cigarettes per day	0.378436	0.318004
stroke	336319	0	0 cigarettes per day	1 to 14 cigarettes per day	0.86289	0.256178
stroke	336319	0	0 cigarettes per day	25 to 37.5 cigarettes per day	0.871293	0.49953
stroke	336801	0	0 cigarettes per day	22 to 33 cigarettes per day	0.788457	0.31308
stroke	336801	0	0 cigarettes per day	20 to 20 cigarettes per day	0.788457	0.231911
stroke	336801	0	0 cigarettes per day	22 to 33 cigarettes per day	0.470004	0.111607
stroke	336801	1	0 cigarettes per day	20 to 20 cigarettes per day	0.336472	0.080175
stroke	336801	0	0 cigarettes per day	1 to 19 cigarettes per day	0.300105	0.066138
stroke	336801	0	0 cigarettes per day	1 to 19 cigarettes per day	0.300105	0.132275
stroke	343297	0	0 cigarettes per day	1 to 20 cigarettes per day	0.875469	0.212585
stroke	343297	0	0 cigarettes per day	20 to 20 cigarettes per day	0.955511	0.24529
stroke	343297	0	0 cigarettes per day	22 to 33 cigarettes per day	1.252763	0.182216
stroke	343300	0	0 cigarettes per day	21 to 31.5 cigarettes per day	2.230014	0.650099
stroke	343300	1	0 cigarettes per day	21 to 31.5 cigarettes per day	2.054124	0.385924
stroke	343300	0	0 cigarettes per day	21 to 31.5 cigarettes per day	1.824549	0.576037
stroke	343300	1	0 cigarettes per day	0 to 20 cigarettes per day	1.435085	0.291545
stroke	343300	0	0 cigarettes per day	0 to 20 cigarettes per day	1.131402	0.518433
stroke	343300	0	0 cigarettes per day	0 to 20 cigarettes per day	1.629241	0.390156
stroke	343306	0	0 cigarettes per day	1 to 14 cigarettes per day	0.81978	0.148341
stroke	343306	0	0 cigarettes per day	25 to 34 cigarettes per day	1.07841	0.189157
stroke	343306	0	0 cigarettes per day	35 to 52.5 cigarettes per day	1.081805	0.258561
stroke	343306	1	0 cigarettes per day	15 to 24 cigarettes per day	1.172482	0.124787
stroke	343582	0	0 cigarettes per day	1 to 14 cigarettes per day	0.457425	1.341708
stroke	343582	0	0 cigarettes per day	15 to 24 cigarettes per day	0.559616	1.21137
stroke	343582	0	0 cigarettes per day	25 to 37.5 cigarettes per day	0.392042	1.432363
stroke	343582	0	0 cigarettes per day	25 to 37.5 cigarettes per day	0.788457	0.96359
stroke	343582	0	0 cigarettes per day	15 to 24 cigarettes per day	0.582216	1.184301
stroke	343582	0	0 cigarettes per day	25 to 37.5 cigarettes per day	0.385262	1.442107
stroke	343582	0	0 cigarettes per day	25 to 37.5 cigarettes per day	0.285179	1.593908
stroke	343582	0	0 cigarettes per day	1 to 14 cigarettes per day	0.19062	1.751982
stroke	343582	0	0 cigarettes per day	1 to 14 cigarettes per day	0.14842	1.827498
stroke	343582	0	0 cigarettes per day	15 to 24 cigarettes per day	0.04879	2.01895
stroke	343582	0	0 cigarettes per day	1 to 14 cigarettes per day	-0.01005	2.141311
stroke	343582	0	0 cigarettes per day	15 to 24 cigarettes per day	-0.23572	2.683415
stroke	343647	0	0 cigarettes per day	15 to 24 cigarettes per day	0.991271	0.433303
stroke	343647	0	0 cigarettes per day	26 to 39 cigarettes per day	0.794684	0.487908

stroke	343647	0	0 cigarettes per day	26 to 39 cigarettes per day	0.788457	0.595431
stroke	343647	0	0 cigarettes per day	15 to 24 cigarettes per day	0.336472	0.625911
stroke	343647	0	0 cigarettes per day	1 to 14 cigarettes per day	0.587787	0.62585
stroke	343647	0	0 cigarettes per day	15 to 24 cigarettes per day	0.296068	0.260877
stroke	343647	0	0 cigarettes per day	1 to 14 cigarettes per day	0.23679	0.497853
stroke	343647	0	0 cigarettes per day	1 to 14 cigarettes per day	-0.05337	0.227917
stroke	343647	0	0 cigarettes per day	26 to 39 cigarettes per day	0.331925	0.381652
stroke	344308	0	0 cigarettes per day	1 to 19 cigarettes per day	0.620576	0.314077
stroke	344308	0	0 cigarettes per day	20 to 30 cigarettes per day	0.996949	0.201446
stroke	344315	0	0 cigarettes per day	41 to 61.5 cigarettes per day	2.208274	0.639157
stroke	344315	0	0 cigarettes per day	1 to 10 cigarettes per day	0.788457	0.20872
stroke	344315	0	0 cigarettes per day	11 to 20 cigarettes per day	0.916291	0.22449
stroke	344315	0	0 cigarettes per day	21 to 39 cigarettes per day	1.458615	0.486474
stroke	344318	0	0 cigarettes per day	15 to 22.5 cigarettes per day	1.23256	0.356994
stroke	344318	0	0 cigarettes per day	0 to 14 cigarettes per day	0.65752	0.564397
stroke	344318	0	0 cigarettes per day	0 to 14 cigarettes per day	0.722706	0.568407
stroke	344340	0	0 cigarettes per day	0 to 19 cigarettes per day	1.199965	0.638523
stroke	344340	0	0 cigarettes per day	1 to 20 cigarettes per day	1.199965	0.638523
stroke	344340	0	0 cigarettes per day	20 to 30 cigarettes per day	1.813195	0.345823
stroke	344340	0	0 cigarettes per day	21 to 31.5 cigarettes per day	1.813195	0.345823
stroke	344343	0	0 cigarettes per day	1 to 9 cigarettes per day	0.262364	0.255102
stroke	344343	0	0 cigarettes per day	10 to 20 cigarettes per day	0.587787	0.170068
stroke	344343	0	0 cigarettes per day	22 to 33 cigarettes per day	0.788457	0.405844
stroke	344345	0	0 cigarettes per day	16 to 24 cigarettes per day	0.182322	1.766582
stroke	344345	0	0 cigarettes per day	1 to 15 cigarettes per day	0.09531	1.92718
stroke	344345	0	0 cigarettes per day	1 to 15 cigarettes per day	0.587787	1.177721
stroke	344345	0	0 cigarettes per day	16 to 24 cigarettes per day	0.916291	0.847959
stroke	344359	0	0 cigarettes per day	1 to 14 cigarettes per day	0.916291	0.22449
stroke	344359	0	0 cigarettes per day	14 to 25 cigarettes per day	1.064711	0.193526
stroke	344359	0	0 cigarettes per day	26 to 39 cigarettes per day	1.335001	0.194683
stroke	344360	0	0 cigarettes per day	40 to 40 cigarettes per day	0.641854	1.115736
stroke	344360	0	0 cigarettes per day	30 to 30 cigarettes per day	0.512824	1.2694
stroke	344360	0	0 cigarettes per day	40 to 40 cigarettes per day	0.678034	1.07609
stroke	344360	0	0 cigarettes per day	30 to 30 cigarettes per day	0.482426	1.308579
stroke	344360	0	0 cigarettes per day	20 to 20 cigarettes per day	0.322083	1.536158
stroke	344360	0	0 cigarettes per day	10 to 10 cigarettes per day	0.173953	1.781427
stroke	344360	0	0 cigarettes per day	10 to 10 cigarettes per day	0.157004	1.811879
stroke	344360	0	0 cigarettes per day	20 to 20 cigarettes per day	0.336472	1.514213
stroke	344362	0	0 cigarettes per day	40 to 60 cigarettes per day	-0.21072	2.617158
stroke	344362	0	0 cigarettes per day	10 to 39 cigarettes per day	-0.16252	2.493998
stroke	344362	0	0 cigarettes per day	1 to 9 cigarettes per day	0.254642	1.643332
stroke	350743	0	0 cigarettes per day	20 to 30 cigarettes per day	1.686399	0.316515
stroke	350743	0	0 cigarettes per day	21 to 31.5 cigarettes per day	1.686399	0.316515

stroke	350743	0	0 cigarettes per day	1 to 20 cigarettes per day	1.193922	0.255102
stroke	350743	0	0 cigarettes per day	1 to 20 cigarettes per day	1.193922	0.255102
stroke	350747	0	0 cigarettes per day	1 to 19 cigarettes per day	0.806476	0.321155
stroke	350747	0	0 cigarettes per day	0 to 19 cigarettes per day	0.806476	0.321155
stroke	350747	0	0 cigarettes per day	21 to 31.5 cigarettes per day	1.000632	0.39297
stroke	350747	0	0 cigarettes per day	21 to 31.5 cigarettes per day	1.000632	0.39297
stroke	350749	0	0 cigarettes per day	21 to 31.5 cigarettes per day	1.223775	0.554472
stroke	350749	0	0 cigarettes per day	0 to 19 cigarettes per day	0.593327	1.878735
stroke	350753	1	0 cigarettes per day	11 to 20 cigarettes per day	1.358409	0.20395
stroke	350753	0	0 cigarettes per day	21 to 30 cigarettes per day	1.015231	0.262496
stroke	350753	1	0 cigarettes per day	21 to 30 cigarettes per day	2.122262	0.435048
stroke	350753	0	0 cigarettes per day	31 to 46.5 cigarettes per day	1.291984	0.393166
stroke	350753	0	0 cigarettes per day	1 to 10 cigarettes per day	0.65752	0.265676
stroke	350753	0	0 cigarettes per day	11 to 20 cigarettes per day	0.756122	0.197614
stroke	350753	1	0 cigarettes per day	1 to 10 cigarettes per day	1.081805	0.185922
stroke	350757	0	0 cigarettes per day	1 to 9 cigarettes per day	0.582216	0.490252
stroke	350757	0	0 cigarettes per day	10 to 15 cigarettes per day	1.18479	0.409567
stroke	356591	0	0 cigarettes per day	1 to 9 cigarettes per day	0.451076	0.043839
stroke	356591	0	0 cigarettes per day	10 to 19 cigarettes per day	0.615186	0.034433
stroke	356591	0	0 cigarettes per day	20 to 30 cigarettes per day	0.81093	0.036181
stroke	356591	1	0 cigarettes per day	1 to 9 cigarettes per day	0.530628	0.036074
stroke	356591	0	0 cigarettes per day	10 to 19 cigarettes per day	0.530628	0.035863
stroke	356591	0	0 cigarettes per day	20 to 30 cigarettes per day	0.722706	0.034559
stroke	357417	1	0 cigarettes per day	6 to 14 cigarettes per day	1.360977	0.234171
stroke	357417	1	0 cigarettes per day	0 to 5 cigarettes per day	1.272566	0.291545
stroke	357417	1	0 cigarettes per day	15 to 22.5 cigarettes per day	1.536867	0.176103
stroke	357431	0	0 cigarettes per day	21 to 31.5 cigarettes per day	1.029619	0.273324
stroke	357431	0	0 cigarettes per day	21 to 31.5 cigarettes per day	1.570697	0.270482
stroke	357431	0	0 cigarettes per day	1 to 10 cigarettes per day	0.751416	0.493358
stroke	357431	0	0 cigarettes per day	1 to 10 cigarettes per day	0.582216	0.57006
stroke	357431	0	0 cigarettes per day	11 to 20 cigarettes per day	0.620576	0.342879
stroke	357431	0	0 cigarettes per day	11 to 20 cigarettes per day	0.451076	0.357468
stroke	357435	0	0 cigarettes per day	11 to 20 cigarettes per day	0.451076	0.357468
stroke	357435	0	0 cigarettes per day	1 to 10 cigarettes per day	0.582216	0.57006
stroke	357435	0	0 cigarettes per day	11 to 20 cigarettes per day	0.620576	0.342879
stroke	357435	0	0 cigarettes per day	1 to 10 cigarettes per day	0.751416	0.493358
stroke	357435	0	0 cigarettes per day	21 to 31.5 cigarettes per day	1.029619	0.273324
stroke	357435	0	0 cigarettes per day	21 to 31.5 cigarettes per day	1.570697	0.270482
stroke	357445	0	0 cigarettes per day	21 to 31.5 cigarettes per day	1.258461	0.564558
stroke	357445	0	0 cigarettes per day	1 to 20 cigarettes per day	0.688135	0.352528
stroke	358462	0	0 cigarettes per day	20 to 39 cigarettes per day	-0.13926	0.39878
stroke	358462	0	0 cigarettes per day	0 to 19 cigarettes per day	-0.05129	0.373255
stroke	358462	0	0 cigarettes per day	40 to 60 cigarettes per day	1.214913	0.887937

stroke	358466	0	0 cigarettes per day	1 to 10 cigarettes per day	0.19062	0.198178
stroke	358466	0	0 cigarettes per day	41 to 61.5 cigarettes per day	1.656321	0.609519
stroke	358470	0	0 cigarettes per day	0 to 20 cigarettes per day	0.512824	0.168031
stroke	358470	0	0 cigarettes per day	21 to 31.5 cigarettes per day	0.652325	0.220557
stroke	358476	0	0 cigarettes per day	11 to 20 cigarettes per day	0.765468	0.552919
stroke	358476	1	0 cigarettes per day	21 to 31.5 cigarettes per day	1.991976	0.36647
stroke	358476	0	0 cigarettes per day	11 to 20 cigarettes per day	1.272566	0.589522
stroke	358476	0	0 cigarettes per day	21 to 31.5 cigarettes per day	0.683097	0.410998
stroke	358476	0	0 cigarettes per day	0 to 10 cigarettes per day	0.058269	0.700327
stroke	358476	0	0 cigarettes per day	0 to 10 cigarettes per day	0.157004	0.481859
stroke	358506	0	0 cigarettes per day	0 to 20 cigarettes per day	1.193922	0.270563
stroke	358506	0	0 cigarettes per day	21 to 31.5 cigarettes per day	1.722767	0.305211
stroke	358540	0	0 cigarettes per day	1 to 14 cigarettes per day	0.741937	0.170068
stroke	358540	0	0 cigarettes per day	16 to 24 cigarettes per day	0.916291	0.204082
stroke	358652	0	0 cigarettes per day	1 to 9 cigarettes per day	0.371564	0.395848
stroke	412060	0	0 cigarettes per day	21 to 31.5 cigarettes per day	1.363537	0.764654
stroke	412060	0	0 cigarettes per day	1 to 20 cigarettes per day	0.350657	0.370078
stroke	412060	0	0 cigarettes per day	1 to 20 cigarettes per day	0.470004	0.299745
stroke	412060	0	0 cigarettes per day	21 to 31.5 cigarettes per day	0.774727	0.377363
stroke	429295	0	0 cigarettes per day	10 to 15 cigarettes per day	0.955511	0.224581
stroke	429295	0	0 cigarettes per day	1 to 9 cigarettes per day	0.832909	0.230323
stroke	429295	0	0 cigarettes per day	1 to 9 cigarettes per day	1.163151	0.271146
stroke	429295	0	0 cigarettes per day	10 to 15 cigarettes per day	0.955511	0.291351
stroke	432350	0	0 cigarettes per day	25 to 37.5 cigarettes per day	0.425268	0.125159
stroke	432350	1	0 cigarettes per day	15 to 24 cigarettes per day	0.329304	0.091428
stroke	432350	0	0 cigarettes per day	1 to 14 cigarettes per day	0.329304	0.080262
stroke	432350	1	0 cigarettes per day	15 to 24 cigarettes per day	0.019803	0.054696
stroke	432350	1	0 cigarettes per day	1 to 14 cigarettes per day	0.10436	0.048189
stroke	432350	1	0 cigarettes per day	25 to 37.5 cigarettes per day	0.086178	0.072042
stroke	501860	0	0 cigarettes per day	20 to 30 cigarettes per day	1.10194	0.328646
stroke	501860	0	0 cigarettes per day	10 to 19 cigarettes per day	1.381282	0.249557
stroke	501860	0	0 cigarettes per day	1 to 9 cigarettes per day	1.040277	0.279219
stroke	501896	0	0 cigarettes per day	1 to 19 cigarettes per day	0.307485	0.105723
stroke	501896	1	0 cigarettes per day	20 to 30 cigarettes per day	0.29267	0.096445
stroke	501942	0	0 cigarettes per day	30 to 45 cigarettes per day	0.636577	0.285711
stroke	501942	0	0 cigarettes per day	1 to 14 cigarettes per day	-0.10536	0.283508
stroke	501942	0	0 cigarettes per day	30 to 45 cigarettes per day	0.307485	0.362073
stroke	501942	0	0 cigarettes per day	15 to 29 cigarettes per day	0.198851	0.240374
stroke	501942	0	0 cigarettes per day	15 to 29 cigarettes per day	0.122218	0.215161
stroke	501942	0	0 cigarettes per day	30 to 45 cigarettes per day	1.226712	0.525455
stroke	501942	0	0 cigarettes per day	15 to 29 cigarettes per day	-0.75502	0.578746
stroke	501942	0	0 cigarettes per day	1 to 14 cigarettes per day	-0.13926	0.617004
stroke	501942	0	0 cigarettes per day	1 to 14 cigarettes per day	-0.09431	0.325585

stroke	501943	0	0 cigarettes per day	1 to 9 cigarettes per day	0.506818	0.0796
stroke	501943	0	0 cigarettes per day	10 to 20 cigarettes per day	0.587787	0.050907
stroke	501943	0	0 cigarettes per day	21 to 31.5 cigarettes per day	0.806476	0.048793
stroke	502077	0	0 cigarettes per day	20 to 30 cigarettes per day	0.500775	0.0796
stroke	502077	1	0 cigarettes per day	10 to 19 cigarettes per day	0.322083	0.075523
stroke	502077	0	0 cigarettes per day	1 to 9 cigarettes per day	0.139762	0.07844
stroke	502248	0	0 cigarettes per day	1 to 19 cigarettes per day	0.832909	0.266879
stroke	502248	0	0 cigarettes per day	20 to 30 cigarettes per day	0.963174	0.330772
stroke	502250	0	0 cigarettes per day	1 to 9 cigarettes per day	0.300105	0.44859
stroke	502250	0	0 cigarettes per day	1 to 9 cigarettes per day	0.215111	0.4069
stroke	502250	0	0 cigarettes per day	20 to 30 cigarettes per day	0.350657	0.257522
stroke	502250	0	0 cigarettes per day	10 to 19 cigarettes per day	0.314811	0.275971
stroke	502250	0	0 cigarettes per day	20 to 30 cigarettes per day	1.108563	0.116837
stroke	502250	0	0 cigarettes per day	10 to 19 cigarettes per day	0.553885	0.250512
stroke	502250	0	0 cigarettes per day	1 to 9 cigarettes per day	0.19062	0.209689
stroke	502250	1	0 cigarettes per day	20 to 30 cigarettes per day	1.071584	0.079189
stroke	502250	0	0 cigarettes per day	10 to 19 cigarettes per day	0.604316	0.105141
stroke	502250	0	0 cigarettes per day	1 to 9 cigarettes per day	0.41871	0.186361
stroke	502250	0	0 cigarettes per day	10 to 19 cigarettes per day	0.708036	0.115731
stroke	502250	0	0 cigarettes per day	20 to 30 cigarettes per day	0.518794	0.317141
tb	173863	0	0 cigarettes per day	1 to 14 cigarettes per day	0.559616	1.697376
tb	173863	0	0 cigarettes per day	15 to 24 cigarettes per day	0.81093	1.320181
tb	173863	0	0 cigarettes per day	25 to 37.5 cigarettes per day	1.609438	0.594082
tb	236195	1	0 cigarettes per day	1 to 19 cigarettes per day	0.00995	2.940998
tb	236195	0	0 cigarettes per day	20 to 20 cigarettes per day	0.207014	2.414966
tb	236195	0	0 cigarettes per day	1 to 19 cigarettes per day	0.215111	2.39549
tb	236195	0	0 cigarettes per day	20 to 20 cigarettes per day	0.392042	2.007033
tb	236195	0	0 cigarettes per day	22 to 33 cigarettes per day	0.451076	1.89198
tb	236195	0	0 cigarettes per day	22 to 33 cigarettes per day	0.708036	1.463255
tb	298335	0	0 cigarettes per day	0 to 4 cigarettes per day	-0.10536	0.340136
tb	298335	0	0 cigarettes per day	5 to 7.5 cigarettes per day	0.262364	0.333595
tb	298340	0	0 cigarettes per day	5 to 15 cigarettes per day	0.182322	0.297619
tb	298340	1	0 cigarettes per day	16 to 29 cigarettes per day	0.336472	0.291545
tb	298340	0	0 cigarettes per day	30 to 45 cigarettes per day	0.470004	0.57398
tb	298340	0	0 cigarettes per day	0 to 4 cigarettes per day	0.587787	0.198413
tb	327906	0	0 cigarettes per day	0 to 9 cigarettes per day	1.308333	0.59294
tb	327906	0	0 cigarettes per day	10 to 15 cigarettes per day	0.609766	0.596162
tb	327949	0	0 cigarettes per day	5 to 15 cigarettes per day	0.14842	0.628958
tb	327949	0	0 cigarettes per day	0 to 5 cigarettes per day	0.625938	0.795318
tb	327949	0	0 cigarettes per day	15 to 25 cigarettes per day	0.760806	0.454177
tb	327949	0	0 cigarettes per day	26 to 39 cigarettes per day	1.638997	0.665742
tb	327956	1	0 cigarettes per day	20 to 30 cigarettes per day	0.198851	0.148461
tb	327956	0	0 cigarettes per day	1 to 9 cigarettes per day	0.019803	0.332633

tb	327956	0	0 cigarettes per day	10 to 19 cigarettes per day	0.09531	0.435993
tb	327956	0	0 cigarettes per day	1 to 9 cigarettes per day	0.157004	0.139543
tb	327956	1	0 cigarettes per day	10 to 19 cigarettes per day	0.215111	0.131666
tb	327956	0	0 cigarettes per day	20 to 30 cigarettes per day	0.285179	0.726945
tb	343383	0	0 cigarettes per day	1 to 10 cigarettes per day	0.631272	0.343482
tb	343383	0	0 cigarettes per day	11 to 16.5 cigarettes per day	0.625938	0.360486
tb	343582	0	0 cigarettes per day	25 to 37.5 cigarettes per day	1.652497	0.569044
tb	343582	0	0 cigarettes per day	25 to 37.5 cigarettes per day	1.181727	0.911168
tb	343582	0	0 cigarettes per day	25 to 37.5 cigarettes per day	1.890095	0.448702
tb	343582	0	0 cigarettes per day	15 to 24 cigarettes per day	1.075002	1.013791
tb	343582	0	0 cigarettes per day	15 to 24 cigarettes per day	0.350657	2.091837
tb	343582	0	0 cigarettes per day	1 to 14 cigarettes per day	0.270027	2.267487
tb	343582	0	0 cigarettes per day	1 to 14 cigarettes per day	0.029559	2.883891
tb	343582	0	0 cigarettes per day	1 to 14 cigarettes per day	0.019803	2.912165
tb	343582	0	0 cigarettes per day	1 to 14 cigarettes per day	0.86289	1.253337
tb	348044	0	0 cigarettes per day	1 to 10 cigarettes per day	0.582216	0.628492
tb	348044	0	0 cigarettes per day	11 to 16.5 cigarettes per day	1.381282	0.726849
tb	348046	0	0 cigarettes per day	1 to 10 cigarettes per day	0.559616	1.697376
tb	348046	0	0 cigarettes per day	11 to 20 cigarettes per day	1.153732	0.937037
tb	348046	0	0 cigarettes per day	21 to 31.5 cigarettes per day	1.302913	0.807176
tb	348053	0	0 cigarettes per day	15 to 22.5 cigarettes per day	1.671473	0.558347
tb	348053	0	0 cigarettes per day	5 to 9 cigarettes per day	0.371564	2.048557
tb	348053	0	0 cigarettes per day	10 to 14 cigarettes per day	0.828552	1.297121
tb	348053	0	0 cigarettes per day	15 to 22.5 cigarettes per day	1.029619	1.06086
tb	348053	0	0 cigarettes per day	5 to 9 cigarettes per day	1.283708	0.822828
tb	348053	1	0 cigarettes per day	10 to 14 cigarettes per day	1.625311	0.584726
tb	359225	0	0 cigarettes per day	21 to 31.5 cigarettes per day	1.000632	0.469875
tb	359225	0	0 cigarettes per day	1 to 10 cigarettes per day	-0.15082	0.542833
tb	359225	0	0 cigarettes per day	11 to 20 cigarettes per day	0.883768	0.502825
tb	359228	0	0 cigarettes per day	5 to 10 cigarettes per day	0.34359	2.106672
tb	359228	0	0 cigarettes per day	11 to 15 cigarettes per day	0.48858	1.822336
tb	359228	0	0 cigarettes per day	16 to 20 cigarettes per day	0.548121	1.716999
tb	359228	0	0 cigarettes per day	21 to 25 cigarettes per day	1.648659	0.571232
tb	359228	0	0 cigarettes per day	26 to 39 cigarettes per day	1.648659	0.571232
tb	359230	1	0 cigarettes per day	1 to 10 cigarettes per day	0.879627	0.168304
tb	359230	0	0 cigarettes per day	11 to 16.5 cigarettes per day	1.018847	0.142747
tb	359273	0	0 cigarettes per day	0 to 10 cigarettes per day	0.262364	0.237441
tb	359273	0	0 cigarettes per day	11 to 16.5 cigarettes per day	0.593327	0.248055
tb	369826	0	0 cigarettes per day	30 to 45 cigarettes per day	0.182322	0.786565
tb	501926	0	0 cigarettes per day	1 to 12 cigarettes per day	0.57098	0.087541
tb	501926	0	0 cigarettes per day	13 to 19.5 cigarettes per day	0.802002	0.077343
tb	501936	0	0 cigarettes per day	1 to 10 cigarettes per day	0.943906	0.317461
tb	501936	1	0 cigarettes per day	11 to 20 cigarettes per day	2.506342	0.383595

Section 9: GATHER and PRISMA checklists

Section 9.1: PRISMA

Table 9: PRISMA Manuscript Checklist

Section and Topic	Item #	Checklist item	Location where item is reported
**TITLE**			
Title	1	Identify the report as a systematic review.	Following a request by the Journal, systematic review does not appear in the title, but it is in the abstract and first page of the manuscript.
**ABSTRACT**			
Abstract	2	See the PRISMA 2020 for Abstracts checklist.	Page 2, "Abstract", See PRISMA abstract checklist below
**INTRODUCTION**			
Rationale	3	Describe the rationale for the review in the context of existing knowledge.	Main text in the first and second paragraphs
Objectives	4	Provide an explicit statement of the objective(s) or question(s) the review addresses.	Main text (second paragraph)
**METHODS**			
Eligibility criteria	5	Specify the inclusion and exclusion criteria for the review and how studies were grouped for the syntheses.	Main text (Methods Overview and Systematic review of literature and data extraction sections); SI Section 1; SI Section 5
Information sources	6	Specify all databases, registers, websites, organisations, reference lists and other sources searched or consulted to identify studies. Specify the date when each source was last searched or consulted.	Main text (Methods Systematic review of literature and data extraction section); SI Section 1.1
Search strategy	7	Present the full search strategies for all databases, registers and websites, including any filters and limits used.	SI Section 1.1
Selection process	8	Specify the methods used to decide whether a study met the inclusion criteria of the review, including how many reviewers screened each record and each report retrieved, whether they worked independently, and if applicable, details of automation tools used in the process.	Main text (Methods Systematic review of literature and data extraction section); SI Section 1.1
Data collection process	9	Specify the methods used to collect data from reports, including how many reviewers collected data from each report, whether they worked independently, any processes for obtaining or confirming data from study investigators, and if applicable, details of automation tools used in the process.	Main text (Methods Systematic review of literature and data extraction section); SI Section 1.4
Data items	10a	List and define all outcomes for which data were sought. Specify whether all results that were compatible with each outcome domain in each study were sought (e.g. for all measures, time points, analyses), and if not, the methods used to decide which results to collect.	Main text (Methods Overview); SI Section 1.3
	10b	List and define all other variables for which data were sought (e.g. participant and intervention characteristics, funding sources).	SI Section 5.1; SI Section 1.4

		Describe any assumptions made about any missing or unclear information.	
Study risk of bias assessment	11	Specify the methods used to assess risk of bias in the included studies, including details of the tool(s) used, how many reviewers assessed each study and whether they worked independently, and if applicable, details of automation tools used in the process.	Main text (Methods Modeling dose-response relative risk of smoking on the selected health outcomes section); SI Section 5.2; SI Section 3
Effect measures	12	Specify for each outcome the effect measure(s) (e.g. risk ratio, mean difference) used in the synthesis or presentation of results.	Main test (Methods Estimating the mean risk function and the burden of proof risk function section)
Synthesis methods	13a	Describe the processes used to decide which studies were eligible for each synthesis (e.g. tabulating the study intervention characteristics and comparing against the planned groups for each synthesis (item #5)).	Main text (Methods Estimating the age-specific risk function for CVD outcomes); SI Section 2.1; SI Section 5
	13b	Describe any methods required to prepare the data for presentation or synthesis, such as handling of missing summary statistics, or data conversions.	Main text (Methods); SI Section 5.1
	13c	Describe any methods used to tabulate or visually display results of individual studies and syntheses.	Main text (Figure captions)
	13d	Describe any methods used to synthesize results and provide a rationale for the choice(s). If meta-analysis was performed, describe the model(s), method(s) to identify the presence and extent of statistical heterogeneity, and software package(s) used.	Main text (Methods); Main text (Code availability)
	13e	Describe any methods used to explore possible causes of heterogeneity among study results (e.g. subgroup analysis, meta-regression).	Main text (Methods Modeling dose-response relative risk of smoking on the selected health outcomes); SI Section 5.1
	13f	Describe any sensitivity analyses conducted to assess robustness of the synthesized results.	Main text (Model validation); SI Section 6
Reporting bias assessment	14	Describe any methods used to assess risk of bias due to missing results in a synthesis (arising from reporting biases).	Main text (Methods Modeling dose-response relative risk of smoking on the selected health outcomes); SI Section 5.1
Certainty assessment	15	Describe any methods used to assess certainty (or confidence) in the body of evidence for an outcome.	Main text (Methods Modeling dose-response relative risk of smoking on the selected health outcomes); SI Section 5.2
**RESULTS**			
Study selection	16a	Describe the results of the search and selection process, from the number of records identified in the search to the number of studies included in the review, ideally using a flow diagram.	SI Section 1.5
	16b	Cite studies that might appear to meet the inclusion criteria, but which were excluded, and explain why they were excluded.	N/A
Study characteristics	17	Cite each included study and present its characteristics.	SI Section 2
Risk of bias in studies	18	Present assessments of risk of bias for each included study.	SI Section 3
Results of individual studies	19	For all outcomes, present, for each study: (a) summary statistics for each group (where appropriate) and (b) an effect estimate and its precision (e.g. confidence/credible interval), ideally using structured tables or plots.	SI Section 8

Results of syntheses	20a	For each synthesis, briefly summarise the characteristics and risk of bias among contributing studies.	SI Section 5.2
	20b	Present results of all statistical syntheses conducted. If meta-analysis was done, present for each the summary estimate and its precision (e.g. confidence/credible interval) and measures of statistical heterogeneity. If comparing groups, describe the direction of the effect.	Main text (Results); Main text (Figures); SI Section 5.2; SI Section 4
	20c	Present results of all investigations of possible causes of heterogeneity among study results.	N/A
	20d	Present results of all sensitivity analyses conducted to assess the robustness of the synthesized results.	SI Section 6
Reporting biases	21	Present assessments of risk of bias due to missing results (arising from reporting biases) for each synthesis assessed.	SI Section 6
Certainty of evidence	22	Present assessments of certainty (or confidence) in the body of evidence for each outcome assessed.	Main text (Results)
**DISCUSSION**			
Discussion	23a	Provide a general interpretation of the results in the context of other evidence.	Main text (Discussion first and second paragraph)
	23b	Discuss any limitations of the evidence included in the review.	Main text (Discussion limitations paragraph)
	23c	Discuss any limitations of the review processes used.	Main text (Discussion limitations paragraph)
	23d	Discuss implications of the results for practice, policy, and future research.	Main text (Discussion fourth paragraph)
**OTHER INFORMATION**			
Registration and protocol	24a	Provide registration information for the review, including register name and registration number, or state that the review was not registered.	The Global Burden of Disease, Injuries, and Risk Factors study has been registered through UW IRB (#9060). This systematic review is encompassed in this larger study.
	24b	Indicate where the review protocol can be accessed, or state that a protocol was not prepared.	A protocol specifically for this review update was not prepared.
	24c	Describe and explain any amendments to information provided at registration or in the protocol.	N/A
Support	25	Describe sources of financial or non-financial support for the review, and the role of the funders or sponsors in the review.	Main text (Acknowledgements)
Competing interests	26	Declare any competing interests of review authors.	Main text (Competing interests)
Availability of data, code and other materials	27	Report which of the following are publicly available and where they can be found: template data collection forms; data extracted from included studies; data used for all analyses; analytic code; any other materials used in the review.	SI Section 1.4; SI Section 8; Main text (Data availability); Main text (Code availability)

Table 10: PRISMA Abstract Checklist

Section and Topic	Item #	Checklist item	Reported (Yes/No)

**TITLE**			
Title	1	Identify the report as a systematic review.	Following a request by the Journal, systematic review does not appear in the title, but it is in the abstract and first page of the manuscript.
**BACKGROUND**			
Objectives	2	Provide an explicit statement of the main objective(s) or question(s) the review addresses.	Yes
**METHODS**			
Eligibility criteria	3	Specify the inclusion and exclusion criteria for the review.	Not in abstract due to word count limitations, but provided in the main text and supplementary information
Information sources	4	Specify the information sources (e.g. databases, registers) used to identify studies and the date when each was last searched.	Not in abstract due to word count limitations, but provided in the main text and supplementary information
Risk of bias	5	Specify the methods used to assess risk of bias in the included studies.	Not in abstract due to word count limitations, but provided in the main text and supplementary information
Synthesis of results	6	Specify the methods used to present and synthesise results.	Yes
**RESULTS**			
Included studies	7	Give the total number of included studies and participants and summarise relevant characteristics of studies.	Not in abstract due to word count limitations, but provided in the main text and supplementary information
Synthesis of results	8	Present results for main outcomes, preferably indicating the number of included studies and participants for each. If meta-analysis was done, report the summary estimate and confidence/credible interval. If comparing groups, indicate the direction of the effect (i.e. which group is favoured).	Yes, though number of included studies, participants, and summary estimates are only provided in the main text and supplemental information
**DISCUSSION**			
Limitations of evidence	9	Provide a brief summary of the limitations of the evidence included in the review (e.g. study risk of bias, inconsistency and imprecision).	Not in abstract due to word count limitations, but provided in the main text and supplementary information
Interpretation	10	Provide a general interpretation of the results and important implications.	Yes
**OTHER**			
Funding	11	Specify the primary source of funding for the review.	Not in abstract due to word count limitations, but provided in the main text
Registration	12	Provide the register name and registration number.	Not in abstract, but provided in the supplemental information

Section 9.2: GATHER

Table 11: GATHER Checklist

Item #	Checklist item	Reported on page #
**Objectives and funding**		
**1**	Define the indicator(s), populations (including age, sex, and geographic entities), and time period(s) for which estimates were made.	SI Section 5.1 (Scope of the systematic literature review)
**2**	List the funding sources for the work.	Main text (Acknowledgements section)
**Data Inputs**		
For all data inputs from multiple sources that are synthesized as part of the study:		
**3**	Describe how the data were identified and how the data were accessed.	Main text (step 1 in methods); SI Section 1.5 (PRISMA 2020 flow diagrams)
**4**	Specify the inclusion and exclusion criteria. Identify all ad-hoc exclusions.	Main text (Systematic review of literature and data extraction in Methods); SI Section 1.5 (PRISMA 2020 flow diagrams)
**5**	Provide information on all included data sources and their main characteristics. For each data source used, report reference information or contact name/institution, population represented, data collection method, year(s) of data collection, sex and age range, diagnostic criteria or measurement method, and sample size, as relevant.	SI Section 2.1; http://vizhub.healthdata.org/burden-of-proof/; data sources and citations for each risk-outcome pair can be downloaded using the "download" button on each risk curve page)
**6**	Identify and describe any categories of input data that have potentially important biases (e.g., based on characteristics listed in item 5).	Main text (Modelling dose-response relative risk of smoking on selected health outcomes in Methods); SI Section 3, Section 8
For data inputs that contribute to the analysis but were not synthesized as part of the study:		
**7**	Describe and give sources for any other data inputs.	NA
For all data inputs:		
**8**	Provide all data inputs in a file format from which data can be efficiently extracted (e.g., a spreadsheet rather than a PDF), including all relevant meta-data listed in item 5. For any data inputs that cannot be shared because of ethical or legal reasons, such as third-party ownership, provide a contact name or the name of the institution that retains the right to the data.	http://vizhub.healthdata.org/burden-of-proof/ (data sources and citations for each risk-outcome pair can be downloaded using the "download" button on each risk curve page)
**Data analysis**		
**9**	Provide a conceptual overview of the data analysis method. A diagram may be helpful.	Main text (Methods); SI Section 5.2 (Detailed methods for estimating the dose-response risk curves of the 36 health outcomes)
**10**	Provide a detailed description of all steps of the analysis, including mathematical formulae. This description should cover, as relevant, data cleaning, data pre-processing, data adjustments and weighting of data sources, and mathematical or statistical model(s).	Main text (Methods); SI Section 5.2 (Detailed methods for estimating the dose-response risk curves of the 36 health outcomes)
**11**	Describe how candidate models were evaluated and how the final model(s) were selected.	Main text (Model validation); SI Section 5.2, Section 4
**12**	Provide the results of an evaluation of model performance, if done, as well as the results of any relevant sensitivity analysis.	Main text (Model validation); SI Section 4, Section 5.2, Section 6
**13**	Describe methods for calculating uncertainty of the estimates. State which sources of uncertainty were, and were not, accounted for in the uncertainty analysis.	Main text (Estimating the mean risk function and the burden of proof risk function in Methods)

**14**	State how analytic or statistical source code used to generate estimates can be accessed.	https://github.com/ihmeuw-msca/burden-of-proof
**Results and Discussion**		
**15**	Provide published estimates in a file format from which data can be efficiently extracted.	http://vizhub.healthdata.org/burden-of-proof/
**16**	Report a quantitative measure of the uncertainty of the estimates (e.g. uncertainty intervals).	UIs given in all main text figures and SI figures; SI Section 4; http://vizhub.healthdata.org/burden-of-proof/
**17**	Interpret results in light of existing evidence. If updating a previous set of estimates, describe the reasons for changes in estimates.	Main text (Discussion)
**18**	Discuss limitations of the estimates. Include a discussion of any modelling assumptions or data limitations that affect interpretation of the estimates.	Main text (Limitations section in discussion)
