# Supplementary material for: Health effects associated with smoking: a Burden of Proof study
Source: Nat Med. 2022 Oct 10;28(10):2045–55. doi: 10.1038/s41591-022-01978-x (PMC9556318; doi:10.1038/s41591-022-01978-x)
Supplement: Supplementary file 2 — Reporting summary [file 41591_2022_1978_MOESM2_ESM.pdf]

nature portfolio

Corresponding author(s): Xiaochen Dai

Last updated by author(s): Jul 27, 2022

Reporting Summary

Nature Portfolio wishes to improve the reproducibility of the work that we publish. This form provides structure for consistency and transparency in reporting. For further information on Nature Portfolio policies, see our Editorial Policies and the Editorial Policy Checklist.

Statistics

For all statistical analyses, confirm that the following items are present in the figure legend, table legend, main text, or Methods section.

n/a	Confirmed	
☐	☒	The exact sample size (*n*) for each experimental group/condition, given as a discrete number and unit of measurement
☐	☒	A statement on whether measurements were taken from distinct samples or whether the same sample was measured repeatedly
☐	☒	The statistical test(s) used AND whether they are one- or two-sided<br>*Only common tests should be described solely by name; describe more complex techniques in the Methods section.*
☐	☒	A description of all covariates tested
☐	☒	A description of any assumptions or corrections, such as tests of normality and adjustment for multiple comparisons
☐	☒	A full description of the statistical parameters including central tendency (e.g. means) or other basic estimates (e.g. regression coefficient) AND variation (e.g. standard deviation) or associated estimates of uncertainty (e.g. confidence intervals)
☐	☒	For null hypothesis testing, the test statistic (e.g. *F*, *t*, *r*) with confidence intervals, effect sizes, degrees of freedom and *P* value noted<br>*Give P values as exact values whenever suitable.*
☐	☒	For Bayesian analysis, information on the choice of priors and Markov chain Monte Carlo settings
☒	☐	For hierarchical and complex designs, identification of the appropriate level for tests and full reporting of outcomes
☐	☒	Estimates of effect sizes (e.g. Cohen's *d*, Pearson's *r*), indicating how they were calculated

Our web collection on statistics for biologists contains articles on many of the points above.

Software and code

Policy information about availability of computer code

Data collection	No primary data collection was carried out for this analysis.
Data analysis	All codes used for these analyses are publicly available online (https://github.com/ihmeuw-msca/burden-of-proof). This includes codes for the meta-regression engine, the model specification interface, the data processing, and risk-specific custom code, as appropriate. Analyses were carried out using R version 3.6.1 and 4.0.5, Python version 3.8, and Stata version 17.<br><br>To validate key aspects of the meta-regression model used in this analysis, the following packages were used, as described in Zheng et al: metafor (R package available for download at https://www.jstatsoft.org/article/view/v036i03) and dosmesreta (R package available for download at https://www.jstatsoft.org/article/view/v072c01).

For manuscripts utilizing custom algorithms or software that are central to the research but not yet described in published literature, software must be made available to editors and reviewers. We strongly encourage code deposition in a community repository (e.g. GitHub). See the Nature Portfolio guidelines for submitting code & software for further information.

Data

Policy information about availability of data

All manuscripts must include a data availability statement. This statement should provide the following information, where applicable:

- Accession codes, unique identifiers, or web links for publicly available datasets
- A description of any restrictions on data availability
- For clinical datasets or third party data, please ensure that the statement adheres to our policy

The findings from this study are supported by data available in the published literature from PubMed database. Study sources and citations for each risk-outcome pair can be downloaded using the “download” button on each risk curve page at https://vizhub.healthdata.org/burden-of-proof/. Study characteristics and citations for all input data used in the analyses are also provided in the Supplementary Table 3. Also see Supplementary Table 2 for a template of the data collection form.

Human research participants

Policy information about studies involving human research participants and Sex and Gender in Research.

Reporting on sex and gender	No primary data collection was carried out for this analysis, so the study does not involve human research participants. As stated in the methods overview, our estimates are not specific to or disaggregated by specific populations, including by sex. That said, relative risk of prostate cancer was only estimated for males and cervical and breast cancer were only estimated for females because of the sex-specific nature of these outcomes. Because estimates were not disaggregated by sex, we included all available data regardless of how or if the input study collected and reported data by sex or gender. We did not perform sex- or gender-based analyses due to limitations in and scarcity of the underlying data. There is no evidence to suggest that relative risk of an outcome due to smoking varies between males and females.
Population characteristics	No primary data collection was carried out for this analysis, so the study does not involve human research participants. The analysis evaluated the evidence on the association between smoking and 36 disease endpoints.
Recruitment	No primary data collection was carried out for this analysis, so we did not recruit participants.
Ethics oversight	This study was approved by the University of Washington IRB Committee (study #9060).

Note that full information on the approval of the study protocol must also be provided in the manuscript.

Field-specific reporting

Please select the one below that is the best fit for your research. If you are not sure, read the appropriate sections before making your selection.

☒ Life sciences ☐ Behavioural & social sciences ☐ Ecological, evolutionary & environmental sciences

For a reference copy of the document with all sections, see nature.com/documents/nr-reporting-summary-flat.pdf

Life sciences study design

All studies must disclose on these points even when the disclosure is negative.

Sample size	The study was a secondary analysis of existing data involving systematic reviews and meta-analyses. No sample size calculation was performed for this meta-analysis; all available data meeting inclusion criteria were included. As reported in the results section, we reviewed 21,108 studies published in PubMed; and 793 studies met our inclusion criteria and thus were included in the analysis.
Data exclusions	Studies were excluded if they did not meet our inclusion criteria. As described in Supplementary Information file, studies were excluded based on the following exclusion criteria: were an aggregate study: meta-analysis or pooled cohort; had the wrong study type: not a cohort study or a case-control study; were a duplicate study: cohort reported in paper was also reported elsewhere; had unmeasurable exposure: reported smoking consumption without details; had no measure of interest: reported RR for change in smoking or doesn’t report RR; did not have outcome of interest: reported on all-cause-mortality or an outcome outside of the 36 studied in this paper; were not in English.
Replication	This is a meta-analysis of existing studies with many years of cohort and other data. The data and code have all been made available. It could theoretically be replicated.
Randomization	This analysis is a meta-analysis of existing studies and thus, there were no experimental groups.
Blinding	N/A. Blinding was not relevant to this study, as it was a meta-analysis of existing data and we did not collect primary data.

Reporting for specific materials, systems and methods

We require information from authors about some types of materials, experimental systems and methods used in many studies. Here, indicate whether each material, system or method listed is relevant to your study. If you are not sure if a list item applies to your research, read the appropriate section before selecting a response.

Materials & experimental systems

n/a	Involved in the study
☒	☐ Antibodies
☒	☐ Eukaryotic cell lines
☒	☐ Palaeontology and archaeology
☒	☐ Animals and other organisms
☒	☐ Clinical data
☒	☐ Dual use research of concern

Methods

n/a	Involved in the study
☒	☐ ChIP-seq
☒	☐ Flow cytometry
☒	☐ MRI-based neuroimaging
